# Using digital traces to build prospective and real-time county-level early warning systems to anticipate COVID-19 outbreaks in the United States

**DOI:** 10.1126/sciadv.abq0199

**Published:** 2023-01-18

**Authors:** Lucas M. Stolerman, Leonardo Clemente, Canelle Poirier, Kris V. Parag, Atreyee Majumder, Serge Masyn, Bernd Resch, Mauricio Santillana

**Affiliations:** ^1^ Computational Health Informatics Program, Boston Children’s Hospital, Boston, MA, USA.; ^2^ Department of Pediatrics, Harvard Medical School, Boston, MA, USA.; ^3^ Department of Mathematics, Oklahoma State University, Stillwater, OK, USA.; ^4^ Machine Intelligence Group for the Betterment of Health and the Environment, Network Science Institute, Northeastern University, Boston, MA, USA.; ^5^ NIHR Health Protection Research Unit, Behavioural Science and Evaluation, University of Bristol, Bristol, UK.; ^6^ Global Public Health, Janssen R&D, Beerse, Belgium.; ^7^ Department of Geoinformatics - Z-GIS, University of Salzburg, Salzburg, Austria.; ^8^ Center for Geographic Analysis, Harvard University, Cambridge, MA, USA.; ^9^ Harvard University, T.H. Chan School of Public Health, Boston, MA, USA.

## Abstract

Coronavirus disease 2019 (COVID-19) continues to affect the world, and the design of strategies to curb disease outbreaks requires close monitoring of their trajectories. We present machine learning methods that leverage internet-based digital traces to anticipate sharp increases in COVID-19 activity in U.S. counties. In a complementary direction to the efforts led by the Centers for Disease Control and Prevention (CDC), our models are designed to detect the time when an uptrend in COVID-19 activity will occur. Motivated by the need for finer spatial resolution epidemiological insights, we build upon previous efforts conceived at the state level. Our methods—tested in an out-of-sample manner, as events were unfolding, in 97 counties representative of multiple population sizes across the United States—frequently anticipated increases in COVID-19 activity 1 to 6 weeks before local outbreaks, defined when the effective reproduction number *R_t_* becomes larger than 1 for a period of 2 weeks.

## Teaser

Changes in human behavior, as captured by Internet digital traces, anticipate the onset of COVID-19 outbreaks.

## INTRODUCTION

With more than 6 million deaths worldwide as of August 2022, the coronavirus disease 2019 (COVID-19) pandemic has become a global catastrophic event (*1*). The United States alone has reported more than 90 million infections and more than 1 million deaths (*2*). While COVID-19 vaccination strategies have been deployed in the United States since the early months of 2021, the proportion of fully vaccinated individuals is still low, at around 64%. With the emergence of new variants of severe acute respiratory syndrome coronavirus 2 (SARS-CoV-2)—the virus responsible for infecting people with COVID-19—such as Omicron, the observed waning of immunity conferred by vaccines (*3*), and the fact that many nonpharmaceutical interventions (NPIs), such as mask mandates and social distancing, have become less frequently practiced, the United States is still highly vulnerable to the effects of the COVID-19 outbreaks (*4*). Thus, our best line of defense against uncontrolled outbreaks remains to be vaccinated and to adjust our social behavior when sharp increases of infections are first detected (*5*, *6*). In the context of designing timely and appropriate public health responses to slow down infections and eventual deaths, robust real-time indicators of COVID-19 activity are of great importance, as they guide authorities in their decision-making processes.

Tracking COVID-19 in real time with reliable data sources remains a challenge despite many initiatives led by hospitals, local health authorities, and the research community (*7*). For instance, polymerase chain reaction (PCR) COVID-19 test results are typically delayed by multiple days and reported with days or weeks of delay. Testing availability may substantially affect the recorded number of positive COVID-19 cases, which may suggest that changes in COVID-19 activity reflect testing volumes rather than the underlying proportions of infections in the population (*1*, *8*). Furthermore, the reliability, consistency, and, in general, the quality of reported COVID-19 data—such as confirmed cases, hospitalization, and deaths—vary highly from country to country (and within countries) frequently due to disparities in economic resources locally allocated to monitor and respond to the pandemic (*7*).

Statistical models have been proposed to address delays in data collection and ascertainment biases retrospectively and in real time (*9*–*12*). Computational mechanistic [susceptible-infected-recovered (SIR)] models, on the other hand, have been used to reconstruct the spatiotemporal patterns of the spread of COVID-19 retrospectively and to forecast likely COVID-19 cases and deaths to occur in the near future (*13*–*19*). Many studies characterizing the quality and accuracy of forecasts have emerged from COVID-19 initiatives coordinated by the U.S. Centers for Disease Control and Prevention (CDC) (*20*). Those models are usually based on mechanistic SIR-like systems, sometimes with added inference frameworks (*21*, *22*). Despite their ability to explore potential “what if” scenarios and their accuracy during periods where the epidemic curves have been monotonically increasing or decreasing, most of these forecasting models have not been very consistent or reliable in anticipating sharp changes in disease activity (*23*).

Several studies have also shown the potential utility of “digital” (or internet-based) data sources as a complementary way to track (and/or confirm) changes in disease activity at the population level (*24*–*31*). In the past, many approaches explored valuable information from search engines (*29*, *32*–*35*), Twitter microblogs (*36*–*38*), and electronic health records (*39*–*41*) for real-time estimates of disease incidence and characterized the limitations of those nontraditional data sources in the context of influenza (*42*, *43*). In the past 2 years, statistical and machine learning approaches have explored how to incorporate disease-related internet search data to track and forecast COVID-19 activity (*44*–*46*), with some limitations documented (*47*). The logic behind using disease-related “digital data” to monitor disease activity is that user-generated digital traces may capture changes in human behavior (human mobility, situational awareness, increases in certain clinical treatments, population-level topic interests, social media trending content) that may have an impact on disease transmission and/or may reflect increases in symptomatic infections (*48*, *49*).

Kogan *et al*. (*50*) explored the effectiveness of Google Trends, Twitter microblogs, clinician searches, anonymized human mobility from mobile phones records, and smart thermometers to anticipate increases and decreases in COVID-19 activity at the state level, as reported by health care systems. By combining multiple data streams, they proposed a Bayesian indicator capable of predicting an impending COVID-19 outbreak with several weeks of anticipation in near real time. Their methods were successful when tested in a retrospective fashion and during the first half of the year 2020. However, at the time of their study, Kogan and colleagues (*50*) did not have enough data to perform out-of-sample validation tests, which is now possible given the higher number of COVID-19 outbreaks. Moreover, Kogan *et al*. (*50*) did not explore the feasibility of using their approaches at finer spatial resolutions, such as the county level, where the signal-to-noise ratio in aggregated digital data streams may be compromised and where most outbreak control strategies are implemented in the United States.

Here, we present a framework to deploy a prospective real-time machine learning–based early warning system (EWS) to anticipate or confirm COVID-19 outbreaks at the county level in the United States. Our choice of counties was based on a list of locations identified by our collaborators at Johnson & Johnson as potentially suitable locations (with a range of population sizes) to deploy clinical trials for their vaccine. In addition, we included the most highly populated counties in the country leading to a total of 97 counties included in our analysis. Our systems leverage the predictive power of both individual internet-based data sources and their combined consensus. We quantify their predictive performance in a prospective out-of-sample way from January 2020 to January 2022, including the most recent periods when the highly contagious Omicron variant was detected. By implementing event detection algorithms on each of these internet-based time series and using machine learning strategies to combine this information, we anticipate the onset of local COVID-19 outbreaks—defined as the time when the local effective reproductive number, *R_t_*, becomes larger than 1 in a given region (*51*). In comparison to the current state-of-the-art models on COVID-19 prediction led by the CDC’s COVID-19 Forecasting Hub Consortium (*23*, *52*–*54*), our methods do not aim to predict the number of cases or deaths, but rather were designed to detect sharp increases in COVID-19 activity, a task that the current CDC models have continuously failed to accomplish as stated by Cramer *et al*. (*52*): “Most forecasts [within the CDC’s COVID-19 Forecast Hub Consortium] have failed to reliably predict rapid changes in the trends of reported cases and hospitalizations. Due to this limitation, they should not be relied upon for decisions about the possibility or timing of rapid changes in trends.” Our approach aims to fill this gap, providing an EWS that specifically focuses on predicting rapid increases in the trends of reported COVID-19 cases.

## RESULTS

We analyzed COVID-19 activity in 97 U.S. counties across the United States between 1 January 2020 and 1 January 2022. First, we identify weeks when the local reproductive number (commonly denoted by *R_t_*) was higher than 1 [with 95% confidence interval as described in (*55*)], suggesting that the local number of secondary COVID-19 infections was larger than 1 per index case. We labeled each first week when the local *R_t_* transitioned from a value smaller than 1 to one above 1 as outbreak onset for each location (see Materials and Methods for details). Interchangeably, we also refer to these outbreak onsets as events here. We identified 464 outbreak onsets at the county level. From this total, 367 events were used to test our methods out of sample, after using the first outbreak onset of each location as initial training data. In this work, we use a dynamic training approach, which is retraining the EWS every time a new outbreak event is observed to reassess the predictive power of all used Google search terms. More specifically, we use all the data up to a given outbreak as our training set and prepare for the upcoming prediction. This technique has been used successfully to address the limitations of Google Flu Trends (*24*, *42*, *49*, *56*). We replicated this analysis for the 50 U.S. states, where we identified 252 outbreak onsets at the state level (a total of 202 out-of-sample outbreak onsets).

We obtained COVID-19–related digital streams for the same time period with the goal of identifying, for example, moments in time when (i) COVID-19–related internet searches, such as fever or anosmia, showed sharp increases—perhaps signaling a population-wide increase of symptomatic infections; (ii) clinicians were looking for dosage information for specific medications to control fever or other COVID-19 symptoms; or (iii) Twitter users expressed that they or their family/friends may have caught COVID-19, among other signatures. We then explored the ability of our methods to extract information from these data sources (individually and as a consensus) to anticipate outbreak onsets for each geographical scale.

Our results are summarized in Figs. 1 and 2 for the county and state levels, respectively. By dynamically training our machine learning methods to recognize temporal patterns that precede increases in COVID-19 activity, we tested their ability to anticipate outbreak onsets. Specifically, we quantified how early they could anticipate unseen outbreaks (referred to as earliness) and the number of times they anticipated, synchronously identified, or lately confirmed a subsequently observed outbreak (referred to as an early, synchronous, or late warning). We also quantified the number of times our methods triggered an alarm, but no outbreak onset was subsequently observed (referred to as a false alarm), and the number of missed outbreaks when no alarm preceded an outbreak onset.

**Fig. 1.**
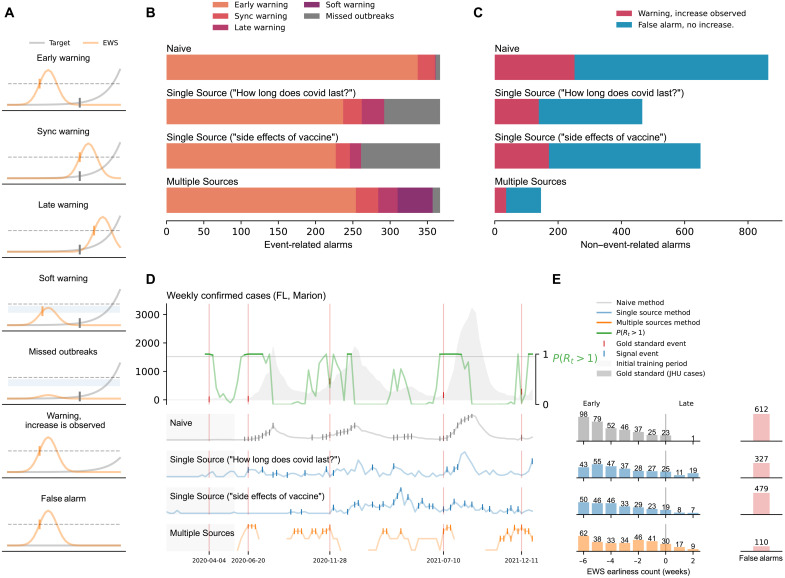
A summary of our results at the county level. (**A**) Graphical representation of the different outcomes observed in our methods: early warnings, synchronous warnings, late warnings, soft warnings, missed outbreaks, warnings with increased activity, and false alarms. (**B**) Summary of the outbreak onset events. Horizontal bars are colored, from orange to purple, depending on the event class. (**C**) False alarms for the Naive, Single Source, and Multiple Source methods. The Multiple Source method produced the lowest amount of false alarms (110). (**D**) Probability of resurgence *P*(*R_t_* > 1) and different events generated by the Naive, Single Source (Google Trends “How long does covid last?” and “side effects of vaccine”), and Multiple Source methods. (**E**) Earliness of the alarms triggered by each method. Bars represent the number of alarms within the out-of-sample time window.

**Fig. 2.**
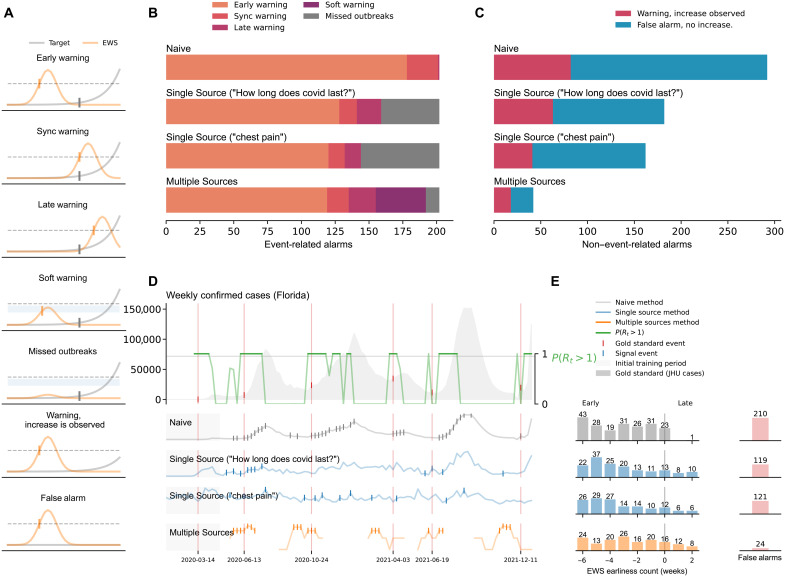
A summary of our results at the state level. (**A**) Graphical representation of the different outcomes observed in our methods: early warnings, synchronous warnings, late warnings, soft warnings, missed outbreaks, warnings with increased activity, and false alarms. (**B**) Summary of the outbreak onset events. Horizontal bars are colored, from orange to purple, depending on the event class. (**C**) False alarms for the Naive, Single Source, and Multiple Source methods. The Multiple Source method produced the lowest amount of false alarms (24). (**D**) Probability of resurgence *P*(*R_t_* > 1) and different events generated by the Naive, Single Source (Google Trends “How long does covid last?” and “chest pain”), and Multiple Source methods. (**E**) Earliness of the alarms triggered by each method. Bars represent the number of alarms within the out-of-sample time window.

Specifically, we defined an early warning whenever an alarm was triggered up to 6 weeks before the outbreak onsets. The choice of a 6-week window was made to plausibly relate a digital trend change with a potential subsequent infection. For example, if a person uses Google to search for COVID-19–related information due to a likely symptomatic infection, that person’s COVID-19 infection may be confirmed in the following week or two, if they are admitted to a health care facility or tested by a provider. Alternatively, such person may search for COVID symptoms not in response to their own symptoms but to someone else’s within their close contact network (that may eventually infect them). In that case, the lag between that internet search and an eventual confirmed infection may be longer, perhaps up to 4 to 6 weeks. We also defined synchronous warning and late warning whenever an alarm was triggered on the same date or up to 2 weeks later than the identified outbreak onset, respectively.

In both Figs. 1 and 2, panel (A) serves as a graphical representation of the different outcomes observed in our EWSs, namely, when the system leads to a successful early, synchronous, or late warning; a false alarm; and a missed outbreak. Two other scenarios were characterized: one that explores when the system may have suggested that a full outbreak would occur but only a mild increase in COVID-19 activity was subsequently observed, labeled as warning, increase is observed, and another labeled as a soft warning, which is observed when the system almost triggered an alarm and an increase of COVID-19 activity was subsequently seen—this could signal an improper model calibration, perhaps a consequence of the small number of events to train the models in a given location. For simplicity, the COVID-19 cases reported by Johns Hopkins University (JHU) are shown in gray, and an early warning indicator is shown in light orange. The horizontal dotted black line represents an EWS’s decision boundary, i.e., a threshold value used to activate an outbreak alarm (see Materials and Methods).

### County-level performance

Figure 1B displays a summary count of all the outbreak onsets observed at the county level. Each horizontal bar is colored, from orange to purple, depending on the event class previously described. In this work, we developed two different machine learning methods: (i) The Single Source method that explores the predictive power of individual digital sources by detecting increases in the search volume of a given term and (ii) the Multiple Source method that incorporates many different signals, optimizing on their best individual performances, to produce a single output (see Materials and Methods). We compared the predictive performance of our methods against an intuitive baseline, which we refer to as the Naive method. The Naive method predicts that an outbreak will happen whenever there is an increase in the number of confirmed COVID-19 cases, i.e., if the COVID-19 cases increase on week *t* compared to week *t* − 1, the Naive method triggers an alarm at week *t*.

#### Early warnings

The alarms produced by the Naive method resulted in early warnings for 337 of the 367 total events (92%) and displayed 23 synchronous warnings and one late warning in the remaining 19 events (6%). The best signal in the Single Source method (Google search term “How long does covid last?”) identified 237 early warnings (65%), 25 synchronous warnings (7%), and 30 (8%) late warnings. Here, we use the term “best signal” to reference those digital traces with a higher number of early warnings across the 97 counties in our dataset. We obtained a comparable performance for the Google search term “side effects of vaccine,” with 227 early warnings (62%), 19 synchronous warnings (5%), and no late warnings. The Multiple Source method identified 254 early warnings (69%), 30 synchronous warnings (8%), and 26 late warnings (7%).

#### Soft warnings and missed outbreaks

The Naive method missed six events (2%), and the Single Source method for the two displayed Google Trends (“How long does covid last?” and “side effects of vaccine”) missed 75 and 106 events (20 and 29%), respectively. For the Multiple Source method, 47 of the 57 remaining events were soft warnings (13% of the total events) and 10 were missed outbreaks (3% of the total events).

#### False alarms

Figure 1C summarizes the false alarms for the different methods in our analysis. The Naive method registered 617 false alarms (about 1.7 times the number of observed outbreaks). In comparison, the Single Source method led to 374 false alarms for the term “How long does covid last?” and 479 false alarms for the term “side effects of vaccine” (1 and 1.3 times the number of outbreaks, respectively). The Multiple Source method produced the lowest amount with only 110 false alarms (0.3 times the number of outbreaks), being the best model in this aspect. The Naive method also exhibited the highest number of “warnings with an increase” observed (252 registered events), followed by the Single Source method (139 events for “How long does covid last?” and 171 for “side effects of vaccine”) and the Multiple Source method with 36 events. In terms of false discovery rates (FDRs), the Naive method exhibited a 0.63 rate approximately. Our intuitive Single Source method alone dropped the number of false alarms significantly (leading to a 0.56 FDR, i.e., one in two alarms are false) while still producing early or at least synchronous warnings on 237 for Google Trends term “How long does covid last?”. From a decision-making perspective, having fewer false alarms is critical to reducing an unnecessary burden of resources—alarm fatigue—and workforce (*57*, *58*). Moreover, a system with many false alarms may lead to distrust within the end-user community. Our Multiple Source method markedly decreased the number of false alarms (110, or a 0.28 FDR, which suggest that only about one in three alarms are false) while displaying successful early and synchronous warnings in 78% of observed outbreaks. See Table 1 for FDR scores for all methods at the county level.

**Table 1.** FDR for the Multiple Source method at the county and state levels. Each row shows the FDR with the inclusion of different warning signals (early, sync, or late).

	Multiple Source (county level)	Multiple Source (state level)
Early	0.30	0.17
Early + sync	0.28	0.15
Early + sync + late	0.28	0.15

Figure 1D shows a graphical representation of the probability of resurgence *P*(*R_t_* > 1), i.e., the probability that the effective reproductive number is higher than 1 given the data, along with the weekly confirmed COVID-19 cases (gray-filled curve in the top), and three representative signals for the Naive, Single Source, and Multiple Source methods for the county of Marion (FL). We chose to depict this specific county as an example where our methods performed well (but similar visualization for all counties and states can be found in the Supplementary Materials). Technically, this panel shows the conditional probability $P(R_{t}>1|I_{1}^{t})$ where *I*_1_ denotes the initially infected population. For notational simplicity, we write *P*(*R_t_* > 1) throughout the manuscript. The outbreak onsets were thus defined when *P*(*R_t_* > 1) > 0.95 and marked with red vertical lines that extend across the five horizontal panels in Fig. 1D containing the COVID-19 case counts and the three early warning methods. Triggered alarms are displayed as vertical tick marks for each method (Naive, Single Source, and Multiple Source methods in gray, blue, and yellow, respectively).

#### Earliness

Figure 1E shows the earliness (in weeks) for the alarms triggered by each method. The bars represent a count of the number of activated alarms within the out-of-sample time window (between 6 weeks before and 2 weeks after the outbreak onset). Triggered alarms that did not precede any increment in the activity of confirmed COVID-19 cases within the 6-week observational window were considered false alarms (displayed in red). We observed that most early warning activation counts of the Naive and Single Source methods fell within the 4- and 6-week early range (68% Naive and 61 and 62% for the Single Source). The Multiple Source method’s highest count (62 alarms) fell within the 6-week early mark (26%). The rest of the activations were spread across the 5- to 1-week early mark, with a higher number of activations as we reached the sync warning mark.

#### Omicron-attributable outbreaks

A total of 62 outbreak onsets were observed at the county level after 1 December 2021. The Single Source method correctly identified 35 and 43 early warnings (56 and 69%) for the Google Trends terms “How long does covid last?” and “side effects of vaccine,” respectively. The Multiple Source method anticipated 54 (87%) outbreak onsets.

### State-level performance

Figure 2 summarizes the state-level results.

#### Early warnings

The alarms produced by the Naive method preceded COVID-19 outbreak onsets in 178 of the 202 total events (88%) and displayed a synchronous warning in 23 (11%) events and only one late warning. The best signal in the Single Source method (Google Trends “How long does covid last?”) identified 128 early warnings (63%), 13 synchronous warnings (6%), and 18 late warnings (9%). Comparable results were found for the Google Trends term “chest pain” with 120 early warnings (59%), 12 synchronous warnings (6%), and 12 late warnings (6%). On the other hand, the Multiple Source method produced 119 early warnings (59%), 16 synchronous warnings (8%), and 20 late warnings (10%).

#### Soft warnings and missed outbreaks

The Naive method had no missed events, and the Single Source method for the two displayed Google Trends (“How long does covid last?” and “chest pain”) missed 43 and 58 events (21 and 29%), respectively. For the Multiple Source method, the remaining 47 events were characterized by 37 soft warnings and 10 missed outbreaks, representing 18 and 5% of the total events.

#### False alarms

The Naive method led to 271 false alarms (Fig. 2C), about 1.3 times the number of observed outbreaks. The Single Source method produced 171 false alarms for the Google Trends term “How long does covid last?” and 151 false alarms for the term “chest pain” (about 0.84 and 0.74 times the number of outbreaks, respectively). The Multiple Source method led to 24 false alarms (about 0.11 times the number of outbreaks), the lowest of all methods. For “warnings with an increase” observed, the Naive method exhibited 74 events, followed by the Single Source method with 82 and 63 events for the two best signals, and the Multiple Source method with only 18 events. At the state level, the Naive method also exhibited the highest ability to identify outbreaks at the cost of having a 0.51 FDR, i.e., one in two alarms were false. As in the county-level analysis, our methods maintained a high rate of early warnings with a substantial decrease in the number of false alarms. Our Multiple Source method successfully identified 135 (early/sync) of the 202 outbreak events for 50 states, with only 24 false alarms (0.15 FDR), i.e., about one in six alarms are false. See also table S8 for FDR scores for all methods at the state level. Table 1 summarizes the FDR scores of the Multiple Source method at the county and state levels.

Figure 2D shows a graphical representation of the probability of resurgence *P*(*R_t_* > 1) and weekly confirmed COVID-19 cases in Florida. The top signals for the Single Source method and the output of the Multiple Source method are also shown, with tick marks representing the triggered alarms for each method. In Fig. 2E, we observe that the Naive method’s highest early warning counts fell within the −6-, −3-, and −1-week mark (approximately 59% of total early warning rates). The highest counts for the Single Source method fall between the 4- and 6-week early mark (65 and 69% for the best signals, respectively). The majority of early warning activations of the Multiple Source methods fall between 3- and 6-week early range (83 or 69%).

#### Omicron-attributable outbreaks

We observed 19 state-level outbreak onsets occurring after 1 December 2021. The Single Source methodology preceded 13 and 12 (68 and 63%, accordingly) outbreak onsets. The Multiple Source method preceded 18 of the 19 (95%).

### Geographical county-level analysis

Given the lower number of false alarms and the number early warning rates of the Multiple Source method, we investigated this method more extensively. Figure 3 shows a detailed breakdown of the performance for each county in our dataset. We implemented a *k*-means clustering approach to group the counties based on their COVID-19 weekly activity. We separated each set of selected locations into three different groups: the set of counties that experienced their first outbreak at the beginning of 2020 (blue), the set of counties that experienced their first outbreak during summer 2020 (yellow), and a set of counties that experienced their first major outbreak after the summer of 2020 (green). Figure 3A shows the geographical location of the selected counties across the U.S. map, where the colors represent each cluster. The clustering analysis highlights how the COVID-19 outbreak dynamics seem heavily dependent on the geographical location. Counties in cluster 1 were mainly located in the northeast part of the country, while counties in clusters 2 and 3 were scattered throughout the south and north/central regions, respectively.

**Fig. 3.**
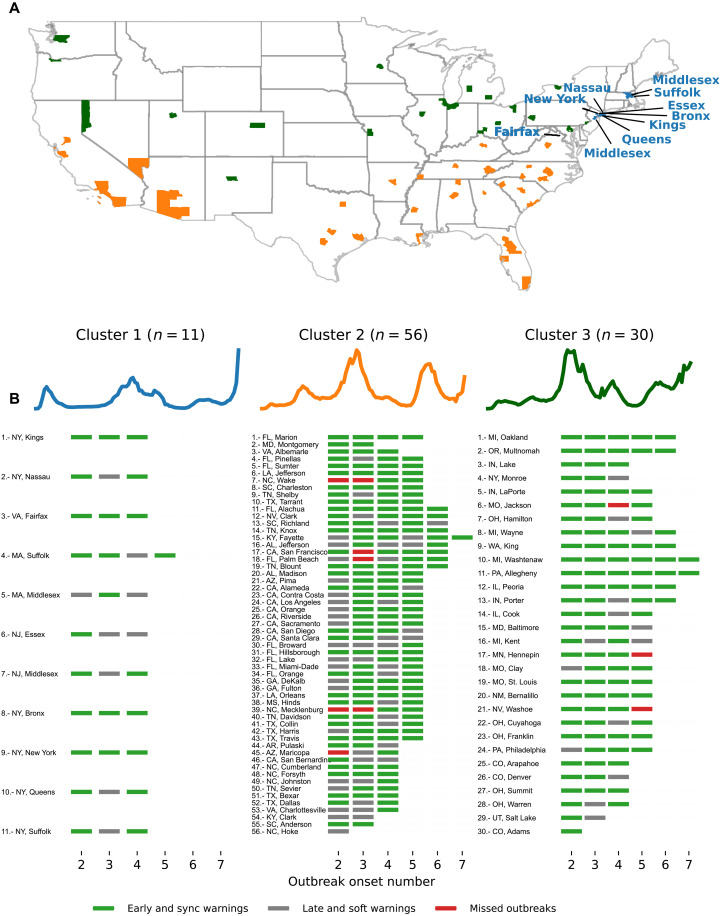
Clustering analysis at the county level based on confirmed COVID-19 case trajectories. (**A**) The geographical map color codes each location based on their cluster. A total of three clusters described groups of counties that experienced their first outbreak onset early in 2020 (blue), during summer (yellow), and late in 2020 (green). (**B**) Blue, magenta, and gray markers correspond to the performance of the Multiple Source method for each out-of-sample outbreak onset. For example, the first location in cluster 1, Kings (NY), experienced three out-of-sample events: All of them were either early or synchronous warnings.

Figure 3B shows a list of counties under the colored time series representing each cluster center. Along with its name and corresponding number, we display a set of tick marks at the bottom representing the out-of-sample outbreak onset (varying from 2 to 7). For each outbreak onset, we trained our method on the previous onsets. For this reason, onset 1 is used for training only and was not included in the analysis. We then show the performance of the Multiple Source method predicting the out-of-sample outbreak using the following color scheme: green for early and sync warnings, red for missed outbreaks, and gray for soft and late warnings. In general, counties in cluster 1 experienced at most four onset events, while counties in clusters 2 and 3 experienced up to seven outbreak onsets (we refer to our Materials and Methods for a precise definition of outbreak onsets). In cluster 1 (11 counties), 25 early/sync and 9 soft/late events were observed. For cluster 2 (56 counties), we observed 156 early/sync events, 51 soft/late events, and 7 missed outbreaks. The counties that experienced missed outbreaks in cluster 2 were San Francisco, Palm Beach, Maricopa, Mecklenburg, and Wake. For cluster 3 (30 counties), 103 early/sync events, 12 soft/late, and 3 missed outbreaks were observed. Missed events for cluster 3 occurred in Jackson, Hennepin, and Washoe. Normalizing the number of missed events by the number of counties in each cluster, the performance of our models was poorest in cluster 2, corresponding to regions in the south of the United States, where the number of onsets of outbreaks has been higher.

### Geographical state-level analysis

By extending our analysis to the state level (Fig. 4), we observe that cluster 1 (consisting of 13 states) experienced 38 early/sync warnings, 11 soft/late warnings, and 5 missed events (located in Maine, New Jersey, and Pennsylvania). Cluster 2 (20 states) experienced 51 early/sync warnings, 28 soft/late warnings, and 3 missed events (Washington, Arkansas, and California). Cluster 3 (17 states) had 49 early/sync warnings, 18 soft/late, and 2 missed events (Kansas and Minnesota) .

**Fig. 4.**
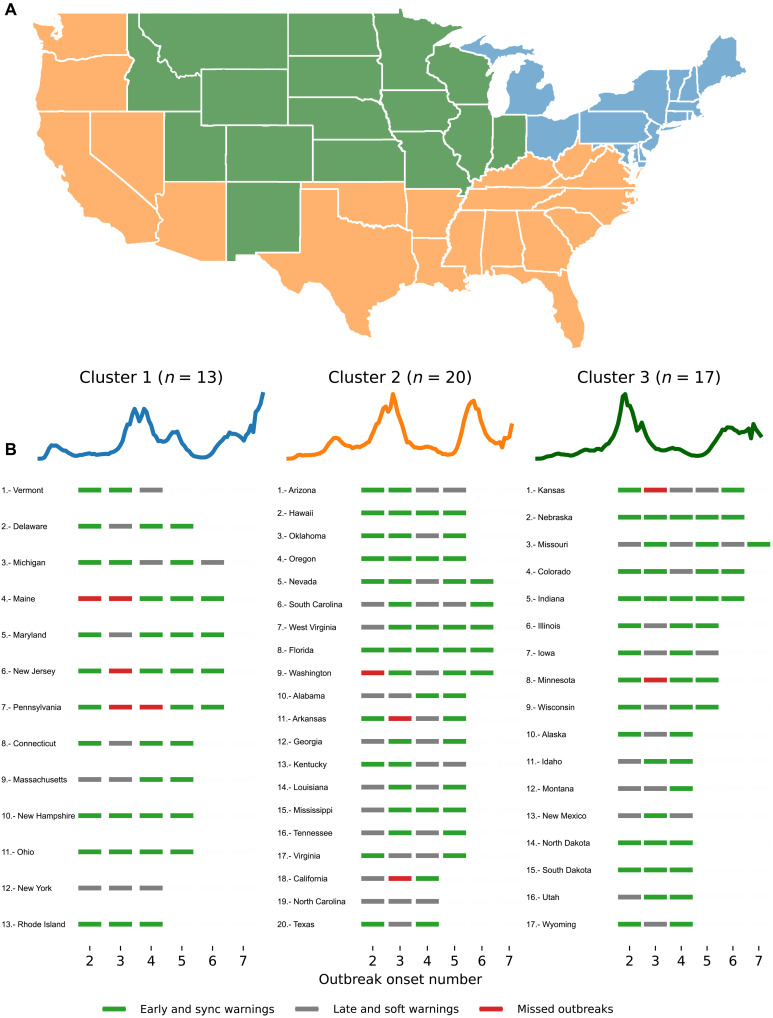
Clustering analysis at the state level based on COVID-19–confirmed case trajectories. (**A**) The geographical map color codes each location based on their cluster. A total of three clusters describe groups of states that experienced their first outbreak onset early in 2020 (cluster 1 in blue), during summer (cluster 2 in yellow), and late in 2020 (cluster 3 in green). (**B**) The set of blue, magenta, and gray markers corresponds to the performance of the Multiple Source method for each out-of-sample outbreak onset.

### Feature importance analysis

Our Multiple Source method dynamically selected the most predictive internet-based data streams and historical epidemiological information—both at the state and county levels—for each location to produce future-looking early warnings. We analyzed which data streams were most informative in our EWSs across time, individually, and across locations (see Materials and Methods for more details). Tables 2 and 3 show the top 10 most frequently digital and historical proxies selected by the Multiple Source method, across all locations, to predict each out-of-sample outbreak onset at the county and state levels. In both cases, the number of out-of-sample outbreak onsets varied from 2 to 7. Moreover, many counties experienced at least two onsets, while fewer counties experienced more than six onsets. Hence, we display six columns with the corresponding number (*n*) of locations for the analysis. County level: Although still available to 54 Google search proxies, the Multiple Source method consistently picked up COVID-19–related terms such as “covid,” “covid-19,” and “covid symptoms” in the first positions, followed by officially confirmed case counts from JHU. State level: We found similar performances for the Multiple Source method at the state level, with COVID-19–related Google Trends leading the list of most selected streams for onsets 2 and 3. After onset 4, officially confirmed cases from JHU also appeared as a highly selected source. Other relevant terms in the top 10 list included the Google Trends terms “acute bronchitis,” “cough,” and “chest pain.”

**Table 2.** Model-selected terms from data streams (ordered by frequency from top to bottom) of the Multiple Source method at the county level. The number of instances that a data stream was selected (values within parentheses) was reduced over time, given that some locations experienced fewer outbreak events than others. GT, Google Trends.

Onset 2 (*n* = 97)	Onset 3 (*n* = 95)	Onset 4 (*n* = 91)	Onset 5 (*n* = 64)	Onset 6 (*n* = 17)	Onset 7 (*n* = 3)
(GT) covid (95)	(GT) covid symptoms (65)	(GT) covid symptoms (61)	(GT) covid symptoms (42)	(GT) covid (10)	(GT) Fever (3)
(GT) covid-19 (89)	(GT) covid-19 (54)	(GT) covid (60)	(GT) covid (34)	(GT) covid-19 (10)	(GT) covid symptoms (2)
(GT) covid symptoms (74)	(GT) covid (53)	Confirmed cases (45)	Confirmed cases (33)	(GT) covid symptoms (8)	Confirmed cases (2)
Confirmed cases (52)	Confirmed cases (34)	(GT) covid-19 (44)	(GT) covid-19 (29)	Confirmed cases (8)	(GT) covid-19 (2)
(GT) chest pain (50)	(GT) Ageusia (28)	(GT) Ageusia (29)	(GT) Ageusia (23)	(GT) Fever (7)	(GT) Phlegm (2)
(GT) quarantine (42)	(GT) chest pain (25)	(GT) chest pain (27)	(GT) Anosmia (20)	(GT) Ageusia (6)	(GT) Asthma (1)
(GT) how long does covid last (33)	(GT) fever (21)	(GT) Anosmia (23)	(GT) chest pain (14)	(GT) Anosmia (5)	(GT) covid (1)
(GT) covid-19 who (24)	(GT) Anosmia (19)	(GT) quarantine (18)	(GT) Fever (13)	(GT) Cough (4)	(GT) Abdominal pain (1)
(GT) chest tightness (20)	(GT) quarantine (17)	(GT) fever (14)	(GT) quarantine (11)	(GT) quarantine (4)	(GT) Bronchitis (1)
(GT) loss taste (15)	(GT) Acute bronchitis (16)	(GT) Acute bronchitis (14)	(GT) Chills (9)	(GT) fever (4)	(GT) quarantine (1)

**Table 3.** Model-selected terms from data streams (ordered by frequency from top to bottom) of the Multiple Source method at state level. The number of instances that a data stream was selected (values within parentheses) was reduced over time, given that some locations experienced less outbreak events than others.

Onset 2 (*n* = 50)	Onset 3 (*n* = 50)	Onset 4 (*n* = 50)	Onset 5 (*n* = 36)	Onset 6 (*n* = 15)	Onset 7 (*n* = 1)
(GT) covid (50)	(GT) covid symptoms (28)	(GT) covid symptoms (27)	(GT) covid symptoms (26)	(GT) covid symptoms (10)	(GT) Nasal congestion (1)
(GT) covid-19 (49)	(GT) covid (21)	Confirmed cases (18)	(GT) covid (14)	Confirmed cases (9)	(GT) Pneumonia (1)
(GT) covid symptoms (37)	(GT) covid-19 (21)	(GT) covid (17)	(GT) Chest pain (13)	(GT) Cough (6)	(GT) Throat irritation (1)
(GT) chest pain (30)	(GT) chest pain (18)	(GT) Acute bronchitis (16)	Confirmed cases (11)	(GT) Hyperventilation (6)	(GT) nose bleed (1)
Confirmed cases (27)	(GT) fever (17)	(GT) Ageusia (15)	(GT) Cough (10)	(GT) covid (6)	(GT) Chest pain (1)
(GT) quarantine (20)	(GT) Acute bronchitis (16)	(GT) Nasal congestion (14)	(GT) Nasal congestion (9)	(GT) Fever (5)	(GT) Cough (1)
(GT) how long does covid last (18)	Confirmed cases (15)	(GT) fever (14)	(GT) Asthma (9)	(GT) Asthma (5)	
(GT) fever (12)	(GT) Ageusia (12)	(GT) Asthma (13)	(GT) covid-19 (9)	(GT) Chest pain (5)	
(GT) Acute bronchitis (11)	(GT) Anosmia (11)	(GT) covid-19 (13)	(GT) Hyperventilation (9)	twitter_state (3)	
(GT) chest tightness (10)	(GT) Chest pain (10)	(GT) Chest pain (12)	(GT) Bronchitis (9)	(GT) chest pain (3)	

## DISCUSSION

We have presented a set of methods that can be deployed in real time and in prospective mode to anticipate the onset of COVID-19 outbreaks in the United States at the county level. Our proposed methods leverage information from multiple internet-based data sources, commonly called digital traces, as they are collected when humans navigate the internet and serve as proxies of human behavior. The EWS framework presented here extends previous work—conducted retrospectively and at the state level by Kogan *et al*. (*50*)—to the county level, a geopolitical spatial resolution where most outbreak mitigation strategies are designed and deployed in the United States. Specifically, our methods were designed to anticipate sharp increases in COVID-19 transmission, as identified by changes in the effective reproduction number (*R_t_*), an outbreak indicator preferred by the community of epidemiologists (*12*, *55*, *59*, *60*). As an additional step, we compared the performance of our EWS to anticipate outbreaks as detected by the county-level CDC community transmission levels (available at: https://data.cdc.gov/Public-Health-Surveillance/United-States-COVID-19-County-Level-of-Community-T/nra9-vzzn). To achieve this, we first compared the timing of the onset of outbreaks using our event detection approach, based on the values of the effective reproductive number *R_t_*, with outbreaks identified when community activity was high in multiple days within a week as labeled by the CDC COVID-19 indicators. We found that the majority of outbreak onsets (87%) were identified by our *R_t_*-based method either synchronously or multiple weeks before the CDC indicators (fig. S188B). Hence, and given that our EWS leveraging multiple sources anticipates an important number of outbreaks weeks before the activations in the effective reproduction number *R_t_*, our EWS may be able to anticipate events as labeled by the CDC indicators even more weeks in advance.

We developed two methods that incorporate single or multiple digital signals, namely, (i) a Single Source method, which locally identifies the magnitude and the number of uptrends in the digital signals that precede outbreaks, and (ii) a Multiple Source method that dynamically selects a subset of the strongest predictive data streams available at each location historically and combines them prospectively into a single indicator that quantifies the likelihood of occurrence of an outbreak in the following weeks. Both methods are data-driven techniques that continuously incorporate newly available data, making them adaptive and responsive to the frequently changing trends of an emerging disease such as COVID-19.

Both single and Multiple Source methods successfully anticipated most outbreak events between January 2020 and January 2022 for the 97 U.S. counties in our dataset. To compare our methods with a baseline system, we define a Naive method that triggers an alarm whenever there is an increase in COVID-19 cases. As expected, the Naive method had the highest early warning rates, since there was at least one increase in the COVID-19 case counts at least 6 weeks before the outbreak onset events. However, the Naive method leads to a significantly high number of undesirable false alarms—it produced 612 false alarms for about 367 actual events, that is, two of the three alarms produced by this approach are false (assuming that all events were identified by the Naive method). Tables S1 to S4 summarize all early percentage rates for the single and Multiple Source methods at both county and state levels.

At the county level, the single (for the two performing proxies) and Multiple Source methods mainly activated 1 to 6 weeks before *R_t_*. At the state level, most early warnings for the single and Multiple Source methods preceded the outbreak onset events in 4 to 6 weeks. This finding can be contrasted with previous work by Kogan *et al*. (*50*) where a 2- to 3-week anticipation was found. However, note that Kogan *et al*. (*50*) considered a different target quantity to be anticipated by digital traces, namely, when exponential growth in confirmed cases and deaths is observed, not when outbreaks start—in our case, defined as when the reproduction number *R_t_* is higher than 1.

Notably, the performance of our EWSs at the state level was comparable to the county level, as shown in Figs. 1 and 2. This result is important, given that the signal-to-noise ratio in digital data sources tends to decrease as we zoom in to finer spatial resolutions, and thus extracting meaningful signals tends to be more challenging. The spatial resolution dependency of the signal-to-noise ration has been documented in multiple studies that have attempted to extend the use of digital streams to monitor disease activity at finer spatial resolutions and lower population densities (*30*, *31*, *61*, *62*). Our methods seem to overcome this challenge by showing comparable ability and earliness to identify the onset of outbreaks for county and state levels. Moreover, our methods’ predictive performance did not show any dependency on total population or population density across counties, as shown in fig. S288.

The predictive performance of our methods varied across counties, indicating the challenge of accurately detecting COVID-19 outbreaks ahead of time on such a fine spatial resolution. After incorporating knowledge of multiple outbreaks, our ability to anticipate the Omicron variant attributed outbreaks (after 1 December 2021) using our Multiple Source models improved from 66% (200 of 305)—in the time period before Omicron—to 87% (54 of 62) at the county level, and from 55 (101 of 183) to 95% (18 of 19) at the state level. Additional to the time analysis, we performed a geographical analysis by using a *k*-means algorithm on the normalized disease activity curves of each location over time and obtained three different COVID-19 activity clusters for the 97 counties and 50 states analyzed in our validation experiment. Our results showed COVID-19 trajectories that represented meaningful geographic regions (north east for early 2020 outbreaks, south for summer, and north for post-summer outbreaks) generated by the clustering algorithm. As presented in Figs. 1 and 2, although the clustering technique resulted in a meaningful set of clusters, our Multiple Source model performance did not seem to change between them.

The purely data-driven aspect of our Multiple Source method led to significant differences in the most selected predictive features as time progressed. In the first COVID-19 waves, Google Trends and COVID-19 cases (at both county and state levels) were mainly selected. A possible explanation for this alternation of most selected signals might be that Google searches might have lost their early correlation power, as increased awareness of the symptoms COVID-19 was likely in later waves. This preliminary evidence of variability in the chosen signals/features may point to the dynamic nature of how COVID-19 was initially perceived and investigated by the population, as well as the ever-changing trends in COVID-19 outbreaks.

Our Multiple Source method rarely completely missed an outbreak. Instead, we found that the early warning indicator frequently displayed that something was about to happen even when the indicator did not cross the decision threshold. As mentioned before, we refer to this scenario as a “soft warning” for two reasons. First, a low number of events (for a given location) is not enough to properly calibrate a local predictive system. Second, the changing nature of human behavior and the SARS-CoV-2 virus challenges any prediction system. In these cases, one can always argue that the preferred epidemiological approach (when the effective reproductive number, *R_t_*, is larger than 1) would eventually identify these sharp increases in COVID-19 activity. A soft warning can thus be seen as a “yellow light” that may inform a public health official of a significant possibility of an outbreak. It is a way to produce meaningful information when our EWS signal approaches the trained threshold without necessarily crossing it. From a decision-making perspective, a soft warning is substantially different than a missed outbreak, where the EWS output completely fails to identify an upcoming outbreak event. In addition, to characterize the predictive performance of our models, we considered synchronous and late warnings to be different from completely missed outbreaks. Decision-makers frequently need as much reassurance as possible regarding the inevitability of an impending outbreak to impose socially and economically expensive NPIs to curb the effects of outbreaks. Synchronous and late warnings may prove important as complementary and confirmatory signals that may enable decision-makers to enact preventive measures in a timely fashion. Given the exponential growth nature of new outbreaks, having additional confirmation of an impending outbreak may help save lives. In addition, we find that our systems sometimes suggested that an outbreak would occur, but only a slight increase in cases was subsequently observed. We have referred to these instances as “warning, increased observed” in Figs. 1 and 2. Again, these findings should not be interpreted as a failure but a calibration issue that may be mitigated with more observations in a given location. Alternatively, these results raise the hypothesis that our methods might be more accurate on preceding COVID-19 outbreaks with higher incidence, given that *R_t_* is better inferred at larger case numbers (*63*). Future studies could address this question at length.

Our present study has multiple limitations. First, our county-level analysis was conducted in a subset of 97 counties, not in all 3006. We selected a subset of U.S. counties based on the local health care capacity to conduct clinical trials and independently of their population size or population density. We also considered all populous counties with more than 1 million inhabitants, totaling 97 counties. Although our results demonstrate success in the feasibility of deploying early warning methodologies in nonrural, highly populated counties, future studies can explore our single and Multiple Source methods in a larger subset of or all the 3006 U.S. counties, which would allow us to explore our methods’ generalizability across all U.S. geographies. Our current findings do not suggest that the ability to anticipate outbreaks depends on population size or density, as shown in fig. S288. However, our internet search–based methods may struggle to perform well in areas with poor literacy rates and limited access to internet resources, frequently areas that may also suffer from poor health care systems. A possible solution for this challenge may include using state-level EWSs to guide county-level outbreak decision-making. Exploring the accuracy of state-level alarms as guidance for finer spatial resolutions could be an interesting topic for future studies. Our method to determine outbreak events based on the effective reproduction number (*R_t_*) has limitations once *R_t_* estimates from raw case data are known to be biased when case ascertainment rates are unstable. Future studies could evaluate the performance of our method in comparison with different methodologies for outbreak detection, such as change point algorithms, case rate thresholds, or growth rates. Note, however, that each one of those methods has its own limitations, as discussed at length in recent studies (*64*–*66*). Similarly, different Naive models could be considered in this work. We could argue that using two consecutive weeks with an increase in case counts would yield a better baseline system for comparison. In a different direction, we could even use the current CDC community levels (COVID Data Tracker available at https://covid.cdc.gov/covid-data-tracker) as the backbone of a Naive method. In this work, we opted for a Naive method with the earliest activation possible, even at the cost of higher false alarms. Future studies could explore our method’s performance compared to alternative baselines, such as those mentioned above.

From a methodological viewpoint, our Single Source method has shown good predictive power and earliness in anticipating the outbreak onset events. Future studies could refine our analysis by exploring other nuances of the digital time series, such as uptrend magnitude or downtrends associated with decreased COVID-19 activities, as done in our own team’s previous work (*50*). From the event identification perspective using *R_t_*, as shown in (*67*), downticks in *R_t_* are much easier to detect and less problematic to estimate, usually because the incidence is higher at the start of a downtick. For digital traces, however, we have seen that it is possible to do this in (*50*), but it was not the focus of this manuscript. Future work should be focused on achieving this. It would probably involve drops in internet searches for symptoms or a rise in terms related to negative tests, end of quarantines, and other terms related to a “back to normal” sentiment. Likewise, the Multiple Source method was designed to identify an outbreak onsets but no other properties, such as magnitude and timing. Further studies could investigate the relationship between those features and the digital signals to build more sophisticated EWSs. Future efforts could also explore other connections between our results and the probabilistic estimation of *R_t_* adopted in this study, such as cumulative probability lower bounds for the false alarm rates, among others. Moreover, if EWSs, such as the one proposed here, are implemented in practice successfully and lead to the timely implementation of preventive interventions, it would be important to quantify the degree to which a timely response may lead to large enough social behavior changes capable of proving future predictions inaccurate. Note also that digital traces usually exhibit specific statistical properties, such as lack of first- and second-order stationarity, that make results hard to interpret and the application of statistical methods potentially inappropriate (*68*). Another important limitation of our work is the current lack of determinants of success for model performance. In other words, finding the characteristics of counties where our methodology is successful is a challenging problem. Our clustering analysis revealed three geographically distinct regions at state and county levels. We did note that cluster 2 contained multiple counties with bad performance (more missed alerts were seen in this cluster, as shown in Fig. 3). Moreover, counties with different population sizes performed similarly in terms of early and synchronous rates (fig. S288). To the best of our current knowledge, the quality of digital signals (signal versus noise, for instance) and the reliability of the epidemiological data are likely the main predictive factors. Future studies could address this question in more detail. From a machine learning perspective, our methods would likely benefit from learning from more outbreaks in a given location (*69*, *70*). These increased datasets will probably improve the robustness and performance of our analysis if the underlying relationship between predictors (internet-based data streams) and outbreaks maintains temporal coherence.

## MATERIALS AND METHODS

In the following sections, we present the data sources that have been used for our study, along with a detailed explanation of our methods. In this work, we collected several data sources from January 2020 to January 2022 for 97 counties that are potential locations for vaccination trials or have a population of at least 1 million inhabitants.

### Data sources

In this section, we present and describe the epidemiological COVID-19 reports, COVID-19 and health-related searches from Google Trends application programming interface (API), UpToDate trends, and Twitter microblogs.

#### Official COVID-19 reports

We collected daily COVID-19 case counts from the JHU database (*71*). The serial interval (time from symptomatic primary infection to symptomatic secondary infection) was obtained from Ferguson *et al*. (*72*). For each U.S. county, we obtained aggregated weekly time series covering the period between 1 January 2020 and 1 January 2022.

#### UpToDate trends

UpToDate is a software package developed by UpToDate Inc., a company in the Wolters Kluwer Health division (*73*). UpToDate is used by physicians and health centers as a tool to search for medical resources. Unlike Google Trends, all information provided in the UpToDate database is edited by experts using rigorous standards. This study used state-level COVID-19–related UpToDate searches from January 2020 to March 2021. We note that UpToDate stopped providing data after that period and hence could not evaluate the signal performance from April to December 2021.

#### Google Trends

We used the Google Trends API to obtain daily COVID-19–related search terms. For the Single Source method, we chose the following COVID-19–related terms: “Covid,” “Covid19,” “How long does covid last?,” “covid symptoms,” and “Covid 19 WHO.” To account for common COVID-19 symptoms, we also selected Google Trends “fever” and “chest pain.” To account for searches related to vaccination, we also chose the Google Trends terms “after covid vaccine,” “side effects of covid vaccine,” and “effects of covid vaccine.” For the Multiple Source method, we extended the Google Search term pool available by incorporating a subset of terms from a publicly available dataset of health symptoms by Google. The filtering method consisted in keeping COVID-19–related symptoms. A list of all terms can be found in table S9.

#### Twitter API

The Twitter data were harvested by an automated crawler connecting to Twitter’s APIs in a fully automated fashion. The geo-crawler software collects georeferenced social media posts, i.e., tweets with an explicit geospatial reference.

The geo-crawler requests data from two Twitter endpoints: the REST and streaming APIs. The REST API offers several API functionalities to access tweets, including the “search/tweets” end point that enables the collection of tweets from the last 7 days in a moving window. This requires a stringently designed collection procedure to harvest all provided tweets within the fast-moving time window of the API with a minimal number of requests. In contrast, the streaming API provides a real-time data stream that can be filtered using multiple parameters, including a post’s language, location, or user IDs.

The geo-crawler software requests tweets that include location information as either a point coordinate from the mobile device used for tweeting (e.g., GPS) or a rectangular outline based on a geocoded place name. The combination of the REST and streaming APIs makes crawling robust against interruptions or backend issues that inevitably lead to data holes. For example, if data from the streaming API cannot be stored in time, the missing data can be retrieved via the partly redundant REST API.

All tweets used for this study are located in the United States. To filter the data for COVID-19–relevant tweets, we used a simple keyword list as shown in Table 4. We opted for keyword-based filtering because of high performance, filtering in near real time, and its simplicity compared to machine learning–based semantic analysis methods. While a machine learning–based semantic clustering method like guided latent Dirichlet allocation may generate more comprehensive results (e.g., by identifying co-occurring and unknown terms), controlling the ratio between false positives (FPs) and false negatives (FNs) requires extensive experimental work and expert knowledge, which is typically a strong limitation when dealing with large datasets.

**Table 4.** Search term list for Twitter.

covid, corona, epidemic, flu, influenza, face mask, spread, virus, infection, fever, panic buying, state of emergency, masks, quarantine, sars, 2019-ncov

#### Apple mobility

Apple mobility data were generated by counting the number of requests made to Apple Maps for directions in selected locations. Data sent from users’ devices to Apple Maps service are associated with random, rotating identifiers, so Apple does not profile users’ information. Data availability in a particular location is based on several factors, including minimum thresholds for direction requests per day. Data were obtained at www.apple.com/covid19/mobility on 19 January 2022. As of April 2022, this link does not contain updated data. Available mobility data can be accessed in our repository (https://doi.org/10.7910/DVN/TKCJGL).

### Addressing delays in data

Our data streams experience specific availability delays. For example, the most recent values from Google Searches are available up to 36 hours before the current date. In addition, epidemiological reports suffer from backfilling and reporting delays due to postprocessing. Thus, for Google searches, data reported at time *t* were shifted to time *t* + 2 to address the 36-hour delay. Similarly, epidemiological data reported at time *t* were shifted to time *t* + 7. Last, UpToDate data at time *t* were shifted to time *t* + 1.

### Estimating the effective reproductive number *R_t_* and defining outbreak onsets in COVID-19 activity

The effective reproductive number *R_t_* is defined as the expected number of secondary cases generated by a primary case infected at time *t*. It can be used as a near real-time indicator to track trends and changes during an outbreak or to measure the impacts of public health interventions. When *R_t_* > 1, we can expect epidemic growth, whereas when *R_t_* < 1, the epidemic decreases. However, estimating *R_t_* in near real time can be challenging due to delays in reporting cases and under-ascertainment. While the latter is difficult to correct for general bias in all *R_t_* estimation methods, we overcome reporting delays by using cases by date of onset (*55*, *74*).

The most popular approach for near real-time estimation of *R_t_* is the EpiEstim method introduced by Cori *et al*. (*63*). This method, while powerful, can suffer from edge effects and unstable inference during periods of low incidence (*55*). A recent approach from Parag *et al*. (*55*) (EpiFilter) circumvents some of these issues by applying Bayesian smoothing theory to improve estimate robustness (or minimize noise), especially in low-incidence periods and between outbreaks. Because early warning signals are desired in exactly such settings, we use this approach as a ground truth signal for outbreak onset.

In this work, we aimed to anticipate sharp increases in COVID-19 activity, using reported by JHU’s confirmatory cases to determine outbreak onset events when the effective reproduction number (*R_t_*) was probabilistically higher than 1. To this end, we first defined the concept of outbreak onset. In this work, outbreak onsets marked the beginning of an exponential surge for a location’s JHU’s COVID-19–confirmed case signal based on the probability *P*(*R_t_* > 1). For details on how this probability is calculated, we refer the reader to the recent work from Parag and Donnelly (*75*). We labeled a given date as the start of an outbreak onset whenever *P*(*R_t_* > 1) crossed the 0.95 threshold for at least two consecutive weeks. An event was considered finished after *P*(*R_t_* > 1) < 0.05 (consecutive events that happened within at most 1-month gap were considered a single event). Figure 5 exhibits a visual explanation of our outbreak onset definition for the COVID-19–confirmed case activity of Marion county, Florida. We used this definition to identify the onsets that occurred within each of the U.S. locations selected for experimentation (97 counties and 50 states) and tested our ability to anticipate such dates using alternative sources of information. It is important to highlight that, for an outbreak to be identified, the value of the effective reproduction number (*R_t_*) must be above 1 with 95% confidence interval [i.e., *P*(*R_t_* > 1) > 0.95] for at least 2 weeks. For that reason, the Naive method will typically lead to early warnings (Fig. 1B)—a first uptrend being followed by an eventual outbreak event—given that the threshold *P*(*R_t_* > 1) > 0.95 may be reached (in times *t* − 1 and *t* − 2) even without an uptrend in COVID-19 cases at week *t*. Rarely, the Naive method will lead to synchronous warnings.

**Fig. 5.**
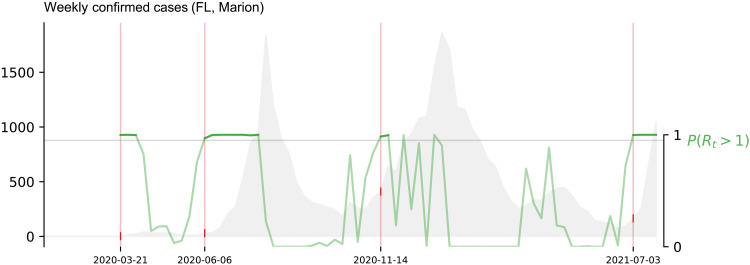
Defining outbreak onsets The value of *P*(*R_t_* > 1) (green line) is calculated for the weekly volume of COVID-19–confirmed cases (gray) in Marion county (FL). A successive increase of COVID-19–confirmed case activity is labeled as an outbreak if *P*(*R_t_* > 1) > 0.95 for 2 weeks or more. Marked events are enclosed within a rectangle.

### Single Source method

As a way to evaluate the predictive power of individual signals, we developed the Single Source method that explores the volume increase of available digital data to generate early warnings of COVID-19 activity. In Fig. 6 (A and B), we illustrate the two possible alarm events. Given a 6-week time window, spanning both digital and COVID-19 cases data, a threshold activation is defined if the digital signal crosses a given threshold τ (Fig. 6A). Figure 6B shows a different kind of alarm, where a number α of increases happen within the 6-week moving window (α = 3 in the example). In this case, we define an α-week trend activation. A true positive (TP) occurs when an alarm in the digital signal (either threshold or α-week trend) precedes the outbreak onset event within the 6-week moving window. Figure 6C illustrates other possible outcomes in the Single Source method. We only show threshold activations for simplicity. An FP occurs when the digital signal activates (either through threshold or α-week trend) but no outbreak onset event occurs. Conversely, an FN may occur when an event occurs but no alarm is triggered by the individual signal. Last, a true negative (TN) takes place when no alarms in the individual signal or outbreak onset events occur within the 6-week moving window.

**Fig. 6.**
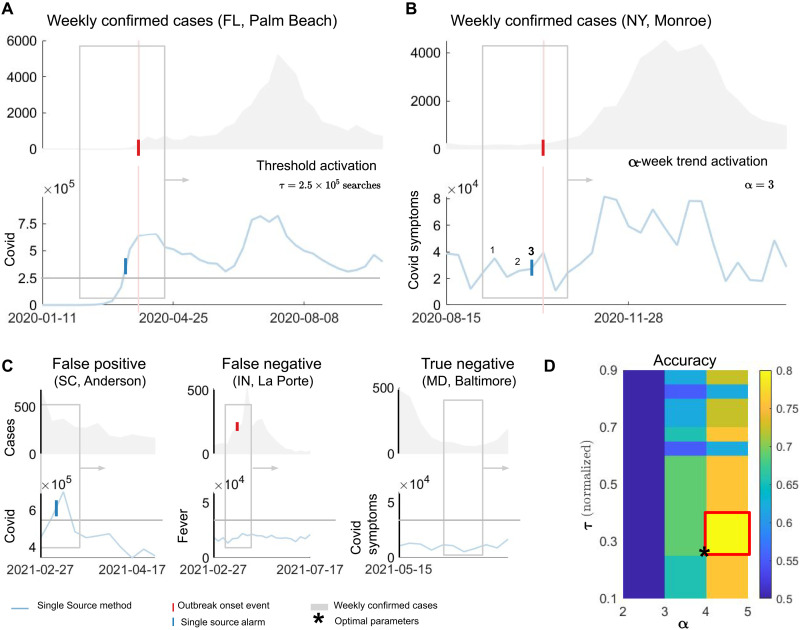
An EWS for COVID-19 based on single sources (Single Source method). A moving window spans both predictor (Google Trends for the term COVID) and COVID-19 cases. A threshold activation occurs when a predictor signal crosses the value τ. In the example, τ = 2.5 × 10^5^ Google searches for Palm Beach County (FL). The blue tick denotes the week of crossing. The red vertical tick and line denote the week of the outbreak onset event. (**B**) If a number α of increases happen in the predictor signal, an α-week trend activation takes place. In the example, α = 3 triggers an alarm for Monroe (NY) county preceding the outbreak onset event. Both (**A**) and (B) represent true positive (TP) events. (**C**) Definitions of false positive (FP), false negative (FN), and true negative (TN) events. (**D**) For the training step, we evaluate the performance of our Single Source method for different thresholds τ (normalized by the maximum of the signal on the training period) and α values for the α-week trend activation. A colormap with the training accuracy shows the highest rate for τ and α. The example illustrates optimal parameters τ and α as the lowest values such that accuracy is maximized, indicated by an asterisk in the lower leftmost corner of the red rectangle.

For the training step, we choose multiple threshold values, which are normalized by the maximum of the digital signal in the training period, resulting in a scale from 0.1 to 0.9. We also select possible values for α in the α-week trend activation ranging between two and five signal uptrends within the 6-week moving window. As the window progresses in time, we compute the number of TPs, FPs, FNs, and TNs. With that information, we obtain performance metrics such as accuracy as depicted in Fig. 6D. From the collection of τ and α maximizing performance metrics (red rectangle in Fig. 6D), we choose those minimum parameter values (black star) to promote the earliness of our prediction method. In our simulations, we chose to simultaneously optimize accuracy ($\frac{\mathrm{TP}+\mathrm{TN}}{P+N}$), precision ($\frac{\mathrm{TP}}{P}$), and the negative predictive values ($\frac{\mathrm{TN}}{N}$), where *P* = TP + FP and *N* = TN + FN. By doing this, we arrive at the minimum optimal τ and α maximizing those three quantities at the same time. If there are no such parameter values, we optimize over precision only.

### Multiple Source method

Here, we describe our Multiple Source method. Given that our earlier work had shown the feasibility of implementing digital streams as alternative proxies to track state-level COVID-19 activity (*50*), we hypothesized that a county-level EWS could provide a higher-resolution picture of the pandemic at near real time. In what follows, we define the variables of our EWS and provide a detailed explanation for each step of our analysis.

### Definitions

To describe our Multiple Source method in detail, in this section, we start defining the following variables.

*y*: Target signal to track using our EWS methodologies. In this work, *y* is given by the time series {*P*(*R_t_* > 1)}_*t* > 0_. We define *y_t_* as the value of *y* at week *t*.

*X* = {*x_i_*, *i* = 1,2, …, *N*}: The set of all alternative proxies, i.e., official epidemiological reports, Google Search volumes, social network activity, Twitter microblogs, among others. We refer the reader to our data sources section for a detailed description of these datasets. We define *x*_*i*, *t*_ as the value of *x_i_* at week *t*.

*t*_e_: Event onset date and can be used to represent outbreak onset events (denoted by *t*_e, y_) generated by the time series {*P*(*R_t_* > 1)}_*t* > 0_ or proxy events (denoted by *t*_e,X*_i_*_) generated by the lambda approach.

*M* = EWS(*y*, *X*): An EWS output as a function of all available data from both target and proxies. We define *M_t_* as the value of *M* at week *t*.

### Lambda approach: Capturing increasing activity trends in digital proxies

To define and label proxy events, we used a measure of a proxy trend, henceforth called λ. Here, we emphasize that λ was calculated for our proxy variables, i.e., the Google Trends signals, Twitter, and other digital traces. However, we derived this measure from the classic SIR epidemic model equations

$$\begin{array}{rl} \dot{S} & =-\beta SI\\ \dot{I} & =\beta SI-\gamma I \end{array}$$ (1)

where *S* and *I* represent the populations of susceptible and infected individuals and *r* = *N* − *S* − *I* represents the recovered class in a constant population of size *N*. For an inter-outbreak period, where the number of infected individuals *I* is very low, the susceptible pool of individuals *S* can be assumed as a constant parameter *S* = S^*^. In this case, the SIR system of equations reduces to

$$\begin{array}{rl} \dot{S} & =0\\ \dot{I} & =(\beta S^{*}-\gamma )I \end{array}$$ (2)

and the solution to the equation for the number of infected individuals *I* is then an exponential function with the intrinsic growth rate

$$\lambda =\beta S^{*}-\gamma$$ (3)

which is directly proportional to the size of the susceptibility pool. In the context of epidemiological models, λ can be thought of as an indicator of the susceptible population that is easily interpretable: If λ > 1, then *I* increases exponentially. Moreover, λ can be estimated by linear regression, as the coefficient of a 1-lag autoregressive model with no intercept *I*_*t*_ = λ*I*_*t*−1_. In this work, we use a 3-week time window to estimate the value of λ.

#### *Implementing* λ *in practice*

For each week *t*, we calculated the value of λ*_t_* using the most recent data available. Retrospectively, a proxy event was defined as a period where the value λ*_t_* > 1 for at least 2 weeks. If two time periods satisfied such conditions but were only separated at most 4 weeks apart, they were merged into a single, bigger period.

### Implementing the Multiple Source method

Our EWS *M* was designed to track COVID-19 outbreak events by iteratively identifying proxy signals *x_i_* that have experienced events preceding a target (*y*) events, and combining them into a single output signal *M_t_*, where 0 ≤ *M_t_* < 1 for all *t* > 0. This output signal *M_t_* can be interpreted as an indicator for *y* experiencing an outbreak in the near future (up to 6 weeks). Given *y* and *X* up to week *t* for a specific location, we implement our method as follows

#### Data preparation

We begin by identifying all the outbreak events for *y* and the proxy events in our dataset *X*. These events are used to establish a performance ranking for each proxy *x_i_* via the following labeling rules (applied individually to each proxy).

TP: If a proxy event *t*_e,*x_i_*_ precedes an outbreak onset event *t*_e,*y*_ by at most 6 weeks, then we label *t*_e,*x_i_*_ as TP.

FP: If a proxy event *t*_e,***x****_i_*_ occurs but there is no outbreak onset event happening in the next 6 weeks, then we label that proxy’s event as a FP, also referred as false alarm.

FN: If an outbreak onset event *t*_e,*y*_ occurs but no proxy event occurs at most 6 weeks retrospectively, then we identify that as a FN.

The number of TP, FP, and FN is then calculated for each proxy *x_i_*. With the aim of focusing specifically in the detection of outbreak events and avoiding a highly imbalanced dataset, we do not optimize for TNs events, since every week where the activity was not an outbreak onset or within the outbreak period (as marked by *R_t_*) would be a potential TN.

#### Feature selection

A performance ranking is created based on the TP, FP, and FN values of each proxy. Given that our main objective is to identify outbreak events for *t*_*e*,*y*_ earlier in time, we prioritize signals maximizing TPs while minimizing FPs. A subset of six proxies χ = {*x*_1_, *x*_2_, ⋯, *x*_6_} with highest TP and lowest FP is then selected to be used as the input for our EWS. We set the number of proxies to 6 to allow all six sources of information (epidemiological, Google trends, UpToDate, neighboring activity, Apple mobility, and Twitter data) to contribute to the Multiple Source method. In the case more than six are fit to be used within our EWS, those six proxies are then selected randomly.

#### Combining selected proxies into an EWS

The value *M_t_* of our EWS is given by the expression

$$M_{t}=2S(n_{e,t})-1$$

where

$$n_{e,t}=\sum_{x_{i}\in \chi }g(x_{i},t)$$

and

$$g(x_{i},t)=\left\{ \begin{array}{cc} 1 & \;\mathrm{if}\;t-k\leq t_{e,x_{i}}\leq t\\ 0 & \;\mathrm{otherwise} \end{array}\right.$$

is the number of proxy events that have occurred in the past *k* = 3 weeks, and

$$S(x)=\frac{1}{1+e^{-x}}$$

is the well-known sigmoid function. Our choice of *k* = 3 weeks (the number of weeks to retrospectively look for activations within a selected proxy) is to restrict the influence of an individual predictor to the output of the EWS to a maximum of 3 weeks. Intuitively, the quantity *M_t_* changes between 0 and 0.9951 (∼1) depending on the number of proxy events (from zero to six) within the past *k* weeks before the week *t*.

#### Thresholding

Although each proxy *x_i_* has been selected on the basis of the premise that its events have successfully tracked our target *y* during training (and thus, even a low value *M_t_* may convey some relevant information about an incoming event), there may be some instances when not all proxies in the set χ have activated before an outbreak onset event. Similarly, as we compute the value of *M_t_* every week, there may be some instances when a proxy (for example, Google search activity spiking due to non–COVID-related events) triggers an alarm, and thus increasing the value of *M_t_*. On the basis of these possibilities, and with the purpose of having a more practical way of interpreting *M_t_*,we define a decision threshold τ, which we use to map *M_t_* into a “yes/no” methodology. If *M_t_* > τ, then we interpret it as our EWS is expecting an outbreak onset event happening in the near future. In practice, we find this threshold by computing the performance of our EWS as a function of the threshold τ (similar to a receiver operating characteristic curve) and selecting the threshold that maximizes a metric of interest (precision, for example).

Given that *M_t_* is calculated every week using a *k*-week moving window, our EWS events consist in a subset of weeks in which *M_t_* > τ (this is to be contrasted to a *x_i_* event, which consists only of a single week). On the basis of this behavior, we define a different set of event labels, which we use to compute the performance of our EWS. We label our events in the following way

TP: If *M_t_* > τ prior *t*_e,*y*_ within a retrospective window of *w* weeks.

Strict FP: If *M_t_* > τ, but no outbreak onset event *t*_e,*y*_ is observed in the following *w* weeks.

Relaxed FP (RFP): Given a set of *m* subsequent weeks where *M_t_* > τ, we count all dates as a single misfire (in comparison to a strict misfire, which would *m* misfires instead of 1).

FN: If *t*_e,**y**_ occurs but *M_t_* > τ is not observed retrospectively within a *w* week window.

We also define

$$m_{1}=\frac{\mathrm{TP}}{\mathrm{TP}+\mathrm{FP}+\mathrm{FN}}$$ (4)

and

$$m_{2}=\frac{\mathrm{TP}}{\mathrm{TP}+\mathrm{FN}+\mathrm{RFP}}$$ (5)

Given that the *M_t_* values are inherently connected overtime (if *M_t_* > τ, then it may take some weeks to deactivate), optimizing for *m*_1_ usually leads to high threshold values as a way to avoid a high number of strict FPs (i.e., keeping *M_t_* > τ a high number of weeks). Although this is desirable in practice (having a system that only activates when it is certain that an event is going to happen, and below the threshold otherwise), *m*_1_ may cause our EWS to overfit given (i) the very low number of events scenario may not convey enough information to find an adequate threshold and (ii) if *M_t_* > 0 for the first occasion at week *t*, then it takes at least *k* weeks to change its value. On the other hand, optimizing for *m*_2_ encourages the selection of lower threshold values as it does not penalize *M_t_* > τ being true for a long period. Nonetheless, *m*_2_ may go as low as a threshold of τ = 0 if there are no FNs or if the number of RFPs is the same below a certain threshold value. We thus opt for optimizing the averaged sum of *m*_1_ and *m*_2_. Precisely, our optimal threshold τ_opt_ is given by

$$\tau_{\mathrm{opt}}={\mathrm{argmax}}_{\tau \in_{\left[0,1\right]}}\left(\frac{m_{1}+m_{2}}{2}\right)$$

where both *m*_1_ and *m*_2_ is given by Eqs. 4 and 5 depending on τ.

#### Out-of-sample experiment description

For a given location with outbreak onset events *E* = {*e*_1_, *e*_2_, , …, *e_n_*}, we used *e*_1_ as the first event for training, with the aim to predict *e*_2_. As a next step, we incorporate *e*_2_ into the training dataset, and thus use both *e*_1_ and *e*_2_ to train our EWS, and predict the following event *e*_3_. We repeated this procedure until the last event *e_n_*. We also defined the out-of-sample period as the time interval between the week when the last outbreak event in the training dataset ended and the week when the out-of-sample outbreak onset occurred.

We counted the number of times when a method triggered an alarm based on the following labels

Early warnings: When the EWS triggered an alarm preceding the outbreak onset event with at most 6 weeks in advance.

Synchronous warnings: When the EWS triggered an alarm on the same date as the outbreak onset.

Late warnings: Events where the EWS triggered an alarm up to 2 weeks later than the outbreak onset event.

For the Multiple Source method, we considered a soft warnings when the output of the EWS increased at least 70% of the decision threshold and the outbreak onset event was successfully observed 6 weeks after.

Missed outbreaks: When an outbreak onset event was observed, but no alarm was registered within the observation window.

In terms of false alarms, we differentiated between two different scenarios:

1) False alarm, no increase: occurred when the system triggered an alarm, but no event and no increase in COVID-19 cases were observed in the following 6 weeks.

2) Warning, increase observed: occurred when the EWS triggered an alarm and was followed by an increase in COVID-19 cases.

Last, to assess our model performance in terms of FDR, we defined

$$\mathrm{FDR}=\frac{\text{False alarms}}{\text{False alarms}+\mathrm{Early},\;\text{Sync, and Late warnings}}$$

Tables S7 and S8 exhibit all FDR values at the state and county levels, also including FDR for different warning signals in the denominator of the fraction.

### Clustering COVID-19 trajectories per county

With the purpose to observe any difference in the performance of our methodologies as a function of the time they experienced different outbreaks, we implemented a *k*-means algorithm over the weekly COVID-19 official reports of each location. We opted to generate three separate groups (clusters) *g_i_*, *i* = 1,2, and 3, to separate counties that experienced their first outbreak in early 2020, summer, and late 2020.

In this context, *k*-means is used as an unsupervised learning methodology, which groups a set of *n* time series (each time series is an *m*-dimensional vector, and each dimension represents a weekly official COVID-19 reports) into *k* clusters. The clusters represent the mean COVID-19 trajectory of the members within the cluster. These are found by iteratively performing the following steps (after initializing each cluster’s location *g_i_* with a random or educated guess of the cluster means)

1) Calculate the distance between each COVID-19 cases time series ${\vec{x}}_{j}$ to each cluster *g_i_*

$$d_{j,i}=\sqrt{\sum_{k=0}^{k=m}\;{\left(x_{j,k}-g_{i,k}\right)}^{2}}$$

2) Assign each time series ${\vec{x}}_{j}$ a membership to the cluster with closest distance (lowest *d*_*j*,*i*_).

3) Update the cluster’s location ${\vec{g}}_{i}$ by calculating the mean over all the members within the cluster.

Each time series is normalized before the clustering procedure to allow for similarity in the trajectories, regardless of the volume of tests reported.

## References

[1] M. Lipsitch, M. Santillana, Enhancing situational awareness to prevent infectious disease outbreaks from becoming catastrophic. Curr. Top Microbiol. Immunol. 424, 59–74 (2019). 31292726 10.1007/82_2019_172

[2] Worldometer, www.worldometers.info/coronavirus [accessed 28 August 2023].

[3] Y. Goldberg, M. Mandel, Y. M. Bar-On, O. Bodenheimer, L. Freedman, E. J. Haas, R. Milo, S. Alroy-Preis, N. Ash, A. Huppert, Waning immunity after the bnt162b2 vaccine in Israel. N. Engl. J. Med. 385, e85 (2021). 34706170 10.1056/NEJMoa2114228 PMC8609604

[4] S. M. Kissler, C. Tedijanto, E. Goldstein, Y. H. Grad, M. Lipsitch, Projecting the transmission dynamics of sars-cov-2 through the postpandemic period. Science 368, 860–868 (2020). 32291278 10.1126/science.abb5793 PMC7164482

[5] M. Dashtbali, M. Mirzaie, A compartmental model that predicts the effect of social distancing and vaccination on controlling covid-19. Sci. Rep. 11, 1–11 (2021). 33854079 10.1038/s41598-021-86873-0 PMC8046797

[6] B. Buonomo, R. D. Marca, Effects of information-induced behavioural changes during the covid-19 lockdowns: The case of italy. R. Soc. Open Sci. 7, 201635 (2020). 33204488 10.1098/rsos.201635 PMC7657925

[7] The COVID Tracking Project, https://covidtracking.com/analysis-updates/three-covid-19-data-problems [accessed 9 December 2021].

[8] J. Kaashoek, M. Santillana, Covid-19 positive cases, evidence on the time evolution of the epidemic or an indicator of local testing capabilities? A case study in the United States (April 10, 2020); 10.2139/ssrn.3574849.

[9] T. Jombart, S. Ghozzi, D. Schumacher, T. J. Taylor, Q. J. Leclerc, M. Jit, S. Flasche, F. Greaves, T. Ward, R. M. Eggo, E. Nightingale, S. Meakin, O. J. Brady; Centre for Mathematical Modelling of Infectious Diseases COVID-19 Working Group, G. F. Medley, M. Höhle, W. J. Edmunds, Real-time monitoring of covid-19 dynamics using automated trend fitting and anomaly detection. Philos. Trans. R. Soc. B Biol. Sci. 376, 20200266 (2021). 10.1098/rstb.2020.0266 PMC8165581 34053271

[10] E. Gutierrez, A. Rubli, T. Tavares, Delays in death reports and their implications for tracking the evolution of COVID-19 (2020); https://ssrn.com/abstract=3645304.

[11] F. S. Lu, A. T. Nguyen, N. B. Link, M. Molina, J. T. Davis, M. Chinazzi, X. Xiong, A. Vespignani, M. Lipsitch, M. Santillana, Estimating the cumulative incidence of covid-19 in the United States using influenza surveillance, virologic testing, and mortality data: Four complementary approaches. PLOS Comput. Biol. 17, e1008994 (2021). 34138845 10.1371/journal.pcbi.1008994 PMC8241061

[12] P. M. De Salazar, F. Lu, J. A. Hay, D. Gómez-Barroso, P. Fernández-Navarro, E. Martínez, J. Astray-Mochales, R. Amillategui, A. García-Fulgueiras, M. D. Chirlaque, A. Sánchez-Migallón, A. Larrauri, M. J. Sierra, M. Lipsitch, F. Simón, M. Santillana, M. A. Hernán, Near real-time surveillance of the SARS-CoV-2 epidemic with incomplete data. *PLOS Comput. Biol.* **18**, e1009964 (2022). 10.1371/journal.pcbi.1009964 PMC9004750 35358171

[13] IHME COVID-19 forecasting team, Modeling covid-19 scenarios for the United States. Nat. Med. 27, 94–105 (2020). 33097835 10.1038/s41591-020-1132-9 PMC7806509

[14] M. Monod, A. Blenkinsop, X. Xi, D. Hebert, S. Bershan, S. Tietze, M. Baguelin, V. C. Bradley, Y. Chen, H. Coupland, S. Filippi, J. Ish-Horowicz, M. M. Manus, T. Mellan, A. Gandy, M. Hutchinson, H. J. T. Unwin, S. L. van Elsland, M. A. C. Vollmer, S. Weber, H. Zhu, A. Bezancon, N. M. Ferguson, S. Mishra, S. Flaxman, S. Bhatt, O. Ratmann; Imperial College COVID-19 Response Team, Age groups that sustain resurging covid-19 epidemics in the United States. Science 371, eabe8372 (2021). 33531384 10.1126/science.abe8372 PMC8101272

[15] F. D. Rossa, D. Salzano, A. Di Meglio, F. De Lellis, M. Coraggio, C. Calabrese, A. Guarino, R. Cardona-Rivera, P. De Lellis, D. Liuzza, F. L. Iudice, G. Russo, M. di Bernardo, A network model of italy shows that intermittent regional strategies can alleviate the covid-19 epidemic. Nat. Commun. 11, 1–9 (2020). 33037190 10.1038/s41467-020-18827-5 PMC7547104

[16] A. Vespignani, H. Tian, C. Dye, J. O. Lloyd-Smith, R. M. Eggo, M. Shrestha, S. V. Scarpino, B. Gutierrez, M. U. G. Kraemer, J. Wu, K. Leung, G. M. Leung, Modelling covid-19. Nat. Rev. Phys. Ther. 2, 279–281 (2020). 10.1038/s42254-020-0178-4 PMC7201389 33728401

[17] S. Lai, N. W. Ruktanonchai, L. Zhou, O. Prosper, W. Luo, J. R. Floyd, A. Wesolowski, M. Santillana, C. Zhang, X. Du, H. Yu, A. J. Tatem, Effect of non-pharmaceutical interventions to contain covid-19 in China. Nature 585, 410–413 (2020). 32365354 10.1038/s41586-020-2293-x PMC7116778

[18] J. T. Davis, M. Chinazzi, N. Perra, K. Mu, A. P. Y. Piontti, M. Ajelli, N. E. Dean, C. Gioannini, M. Litvinova, S. Merler, L. Rossi, K. Sun, X. Xiong, I. M. Longini Jr., M. E. Halloran, C. Viboud, A. Vespignani, Cryptic transmission of sars-cov-2 and the first covid-19 wave. Nature 600, 127–132 (2021). 34695837 10.1038/s41586-021-04130-w PMC8636257

[19] S. Chang, E. Pierson, P. W. Koh, J. Gerardin, B. Redbird, D. Grusky, J. Leskovec, Mobility network models of covid-19 explain inequities and inform reopening. Nature 589, 82–87 (2021). 33171481 10.1038/s41586-020-2923-3

[20] U.S. CDC, Forecasts of total covid-19 deaths; www.cdc.gov/coronavirus/2019-ncov/science/forecasting/forecasting-us.html [accessed 10 December 2021].

[21] R. E. Baker, W. Yang, G. A. Vecchi, C. Jessica, E. Metcalf, B. T. Grenfell, Assessing the influence of climate on wintertime sars-cov-2 outbreaks. Nat. Commun. 12, 1–7 (2021). 33558479 10.1038/s41467-021-20991-1 PMC7870658

[22] S. Flaxman, S. Mishra, A. Gandy, H. J. T. Unwin, T. A. Mellan, H. Coupland, C. Whittaker, H. Zhu, T. Berah, J. W. Eaton, M. Monod; Imperial College COVID-19 Response Team, A. C. Ghani, C. A. Donnelly, S. Riley, M. A. C. Vollmer, N. M. Ferguson, L. C. Okell, S. Bhatt, Estimating the effects of non-pharmaceutical interventions on covid-19 in Europe. Nature 584, 257–261 (2020). 32512579 10.1038/s41586-020-2405-7

[23] E. L. Ray, N. Wattanachit, J. Niemi, A. H. Kanji, K. House, E. Y. Cramer, J. Bracher, A. Zheng, T. K. Yamana, X. Xiong, S. Woody, Y. Wang, L. Wang, R. L. Walraven, V. Tomar, K. Sherratt, D. Sheldon, R. C. Reiner Jr., B. Aditya Prakash, D. Osthus, M. L. Li, E. C. Lee, U. Koyluoglu, P. Keskinocak, Y. Gu, Q. Gu, G. E. George, G. España, S. Corsetti, J. Chhatwal, S. Cavany, H. Biegel, M. Ben-Nun, J. Walker, R. Slayton, V. Lopez, M. Biggerstaff, M. A. Johansson, N. G. Reich, Ensemble forecasts of coronavirus disease 2019 (covid-19) in the us. medRXiv 2020.08.19.20177493 (2020). 10.1101/2020.08.19.20177493.

[24] S. Yang, M. Santillana, S. C. Kou, Accurate estimation of influenza epidemics using Google search data via argo. Proc. Natl. Acad. Sci. U.S.A. 112, 14473–14478 (2015). 26553980 10.1073/pnas.1515373112 PMC4664296

[25] S. F. McGough, J. S. Brownstein, J. B. Hawkins, M. Santillana, Forecasting zika incidence in the 2016 latin america outbreak combining traditional disease surveillance with search, social media, and news report data. PLOS Negl. Trop. Dis. 11, e0005295 (2017). 28085877 10.1371/journal.pntd.0005295 PMC5268704

[26] M. Santillana, A. T. Nguyen, M. Dredze, M. J. Paul, E. O. Nsoesie, J. S. Brownstein, Combining search, social media, and traditional data sources to improve influenza surveillance. PLOS Comput. Biol. 11, e1004513 (2015). 26513245 10.1371/journal.pcbi.1004513 PMC4626021

[27] A. F. Dugas, M. Jalalpour, Y. Gel, S. Levin, F. Torcaso, T. Igusa, R. E. Rothman, Influenza forecasting with Google flu trends. PLOS ONE 8, e56176 (2013). 23457520 10.1371/journal.pone.0056176 PMC3572967

[28] K. Lee, A. Agrawal, A. Choudhary, Forecasting influenza levels using real-time social media streams, in *Proceedings of the 2017 IEEE International Conference on Healthcare Informatics (ICHI)* (IEEE, 2017), pp. 409–414.

[29] E. L. Aiken, S. F. McGough, M. S. Majumder, G. Wachtel, A. T. Nguyen, C. Viboud, M. Santillana, Real-time estimation of disease activity in emerging outbreaks using internet search information. PLOS Comput. Biol. 16, e1008117 (2020). 32804932 10.1371/journal.pcbi.1008117 PMC7451983

[30] F. S. Lu, S. Hou, K. Baltrusaitis, M. Shah, J. Leskovec, R. Sosic, J. Hawkins, J. Brownstein, G. Conidi, J. Gunn, J. Gray, A. Zink, M. Santillana, Accurate influenza monitoring and forecasting using novel internet data streams: A case study in the boston metropolis. JMIR Public Health Surveill. 4, e4 (2018). 29317382 10.2196/publichealth.8950 PMC5780615

[31] F. S. Lu, M. W. Hattab, C. L. Clemente, M. Biggerstaff, M. Santillana, Improved state-level influenza nowcasting in the United States leveraging internet-based data and network approaches. Nat. Commun. 10, 1–10 (2019). 30635558 10.1038/s41467-018-08082-0 PMC6329822

[32] Q. Yuan, E. O. Nsoesie, B. Lv, G. Peng, R. Chunara, J. S. Brownstein, Monitoring influenza epidemics in China with search query from baidu. PLOS ONE 8, e64323 (2013). 23750192 10.1371/journal.pone.0064323 PMC3667820

[33] R. T. Gluskin, M. A. Johansson, M. Santillana, J. S. Brownstein, Evaluation of internet-based dengue query data: Google dengue trends. PLOS Negl. Trop. Dis. 8, e2713 (2014). 24587465 10.1371/journal.pntd.0002713 PMC3937307

[34] B. M. Althouse, Y. Y. Ng, D. A. Cummings, Prediction of dengue incidence using search query surveillance. PLOS Negl. Trop. Dis. 5, e1258 (2011). 21829744 10.1371/journal.pntd.0001258 PMC3149016

[35] J. Ginsberg, M. H. Mohebbi, R. S. Patel, L. Brammer, M. S. Smolinski, L. Brilliant, Detecting influenza epidemics using search engine query data. Nature 457, 1012–1014 (2009). 19020500 10.1038/nature07634

[36] R. Nagar, Q. Yuan, C. C. Freifeld, M. Santillana, A. Nojima, R. Chunara, J. S. Brownstein, A case study of the new york city 2012-2013 influenza season with daily geocoded twitter data from temporal and spatiotemporal perspectives. J. Med. Internet Res. 16, e236 (2014). 25331122 10.2196/jmir.3416 PMC4259880

[37] E. Aramaki, S. Maskawa, M. Morita, Twitter catches the flu: Detecting influenza epidemics using twitter, in *Proceedings of the 2011 Conference on Empirical Methods in Natural Language Processing* (Association for Computational Linguistics, Stroudsburg, PA, 2011), pp. 1568–1576.

[38] M. J. Paul, M. Dredze, D. Broniatowski, Twitter improves influenza forecasting. PLOS Curr. 6, (2014). 10.1371/currents.outbreaks.90b9ed0f59bae4ccaa683a39865d9117 PMC4234396 25642377

[39] S. Yang, M. Santillana, J. S. Brownstein, J. Gray, S. Richardson, S. C. Kou, Using electronic health records and internet search information for accurate influenza forecasting. BMC Infect. Dis. 17, 1–9 (2017). 28482810 10.1186/s12879-017-2424-7 PMC5423019

[40] P. Rangarajan, S. K. Mody, M. Marathe, Forecasting dengue and influenza incidences using a sparse representation of Google trends, electronic health records, and time series data. PLOS Comput. Biol. 15, e1007518 (2019). 31751346 10.1371/journal.pcbi.1007518 PMC6894887

[41] M. Santillana, A. T. Nguyen, T. Louie, A. Zink, J. Gray, I. Sung, J. S. Brownstein, Cloud-based electronic health records for real-time, region-specific influenza surveillance. Sci. Rep. 6, 1–8 (2016). 27165494 10.1038/srep25732 PMC4863169

[42] M. Santillana, D. W. Zhang, B. M. Althouse, J. W. Ayers, What can digital disease detection learn from (an external revision to) Google flu trends? Am. J. Prev. Med. 47, 341–347 (2014). 24997572 10.1016/j.amepre.2014.05.020

[43] D. Lazer, R. Kennedy, G. King, A. Vespignani, The parable of Google flu: Traps in big data analysis. Science 343, 1203–1205 (2014). 24626916 10.1126/science.1248506

[44] V. Lampos, M. S. Majumder, E. Yom-Tov, M. Edelstein, S. Moura, Y. Hamada, M. X. Rangaka, R. A. McKendry, I. J. Cox, Tracking covid-19 using online search. NPJ Digit. Med. 4, 1–11 (2021). 33558607 10.1038/s41746-021-00384-w PMC7870878

[45] D. Liu, L. Clemente, C. Poirier, X. Ding, M. Chinazzi, J. Davis, A. Vespignani, M. Santillana, Real-time forecasting of the covid-19 outbreak in chinese provinces: Machine learning approach using novel digital data and estimates from mechanistic models. J. Med. Internet Res. 22, e20285 (2020). 32730217 10.2196/20285 PMC7459435

[46] T. Lu, B. Y. Reis, Internet search patterns reveal clinical course of covid-19 disease progression and pandemic spread across 32 countries. NPJ Digit. Med. 4, 1–9 (2021). 33574582 10.1038/s41746-021-00396-6 PMC7878474

[47] A. I. Bento, T. Nguyen, C. Wing, F. Lozano-Rojas, Y.-Y. Ahn, K. Simon, Evidence from internet search data shows information-seeking responses to news of local covid-19 cases. Proc. Natl. Acad. Sci. U.S.A. 117, 11220–11222 (2020). 32366658 10.1073/pnas.2005335117 PMC7260988

[48] M. Salathé, L. Bengtsson, T. J. Bodnar, D. D. Brewer, J. S. Brownstein, C. Buckee, E. M. Campbell, C. Cattuto, S. Khandelwal, P. L. Mabry, A. Vespignani, Digital epidemiology. PLOS Comput. Biol. 2012, e1002616 (2012). 10.1371/journal.pcbi.1002616 PMC3406005 22844241

[49] M. Santillana, Perspectives on the future of internet search engines and biosurveillance systems. Clin. Infect. Dis. 64, ciw660 (2016). 10.1093/cid/ciw660 27678086

[50] N. E. Kogan, L. Clemente, P. Liautaud, J. Kaashoek, N. B. Link, A. T. Nguyen, F. S. Lu, P. Huybers, B. Resch, C. Havas, A. Petutschnig, J. Davis, M. Chinazzi, B. Mustafa, W. P. Hanage, A. Vespignani, M. Santillana, An early warning approach to monitor covid-19 activity with multiple digital traces in near real time. Sci. Adv. 7, eabd6989 (2021). 33674304 10.1126/sciadv.abd6989 PMC7935356

[51] K. V. Parag, C. A. Donnelly, Using information theory to optimise epidemic models for real-time prediction and estimation. PLOS Comput. Biol. 16, e1007990 (2020). 32609732 10.1371/journal.pcbi.1007990 PMC7360089

[52] E. Y. Cramer, E. L. Ray, V. K. Lopez, J. Bracher, A. Brennen, A. J. C. Rivadeneira, A. Gerding, T. Gneiting, K. H. House, Y. Huang, D. Jayawardena, A. H. Kanji, A. Khandelwal, K. Le, A. Mühlemann, J. Niemi, A. Shah, A. Stark, Y. Wang, N. Wattanachit, M. W. Zorn, Y. Gu, S. Jain, N. Bannur, A. Deva, M. Kulkarni, S. Merugu, A. Raval, S. Shingi, A. Tiwari, J. White, N. F. Abernethy, S. Woody, M. Dahan, S. Fox, K. Gaither, M. Lachmann, L. A. Meyers, J. G. Scott, M. Tec, A. Srivastava, G. E. George, J. C. Cegan, I. D. Dettwiller, W. P. England, M. W. Farthing, R. H. Hunter, B. Lafferty, I. Linkov, M. L. Mayo, M. D. Parno, M. A. Rowland, B. D. Trump, Y. Zhang-James, S. Chen, S. V. Faraone, J. Hess, C. P. Morley, A. Salekin, D. Wang, S. M. Corsetti, T. M. Baer, M. C. Eisenberg, K. Falb, Y. Huang, E. T. Martin, E. M. Cauley, R. L. Myers, T. Schwarz, D. Sheldon, G. C. Gibson, R. Yu, L. Gao, Y. Ma, D. Wu, X. Yan, X. Jin, Y.-X. Wang, Y. Q. Chen, L. Guo, Y. Zhao, Q. Gu, J. Chen, L. Wang, P. Xu, W. Zhang, D. Zou, H. Biegel, J. Lega, S. M. Connell, V. P. Nagraj, S. L. Guertin, C. Hulme-Lowe, S. D. Turner, Y. Shi, X. Ban, R. Walraven, Q.-J. Hong, S. Kong, A. van de Walle, J. A. Turtle, M. Ben-Nun, S. Riley, P. Riley, U. Koyluoglu, D. D. Roches, P. Forli, B. Hamory, C. Kyriakides, H. Leis, J. Milliken, M. Moloney, J. Morgan, N. Nirgudkar, G. Ozcan, N. Piwonka, M. Ravi, C. Schrader, E. Shakhnovich, D. Siegel, R. Spatz, C. Stiefeling, B. Wilkinson, A. Wong, S. Cavany, G. España, S. Moore, R. Oidtman, A. Perkins, D. Kraus, A. Kraus, Z. Gao, J. Bian, W. Cao, J. L. Ferres, C. Li, T.-Y. Liu, X. Xie, S. Zhang, S. Zheng, A. Vespignani, M. Chinazzi, J. T. Davis, K. Mu, A. P. Y. Piontti, X. Xiong, A. Zheng, J. Baek, V. Farias, A. Georgescu, R. Levi, D. Sinha, J. Wilde, G. Perakis, M. A. Bennouna, D. Nze-Ndong, D. Singhvi, I. Spantidakis, L. Thayaparan, A. Tsiourvas, A. Sarker, A. Jadbabaie, D. Shah, N. D. Penna, L. A. Celi, S. Sundar, R. Wolfinger, D. Osthus, L. Castro, G. Fairchild, I. Michaud, D. Karlen, M. Kinsey, L. C. Mullany, K. Rainwater-Lovett, L. Shin, K. Tallaksen, S. Wilson, E. C. Lee, J. Dent, K. H. Grantz, A. L. Hill, J. Kaminsky, K. Kaminsky, L. T. Keegan, S. A. Lauer, J. C. Lemaitre, J. Lessler, H. R. Meredith, J. Perez-Saez, S. Shah, C. P. Smith, S. A. Truelove, J. Wills, M. Marshall, L. Gardner, K. Nixon, J. C. Burant, L. Wang, L. Gao, Z. Gu, M. Kim, X. Li, G. Wang, Y. Wang, S. Yu, R. C. Reiner, R. Barber, E. Gakidou, S. I. Hay, S. Lim, C. Murray, D. Pigott, H. L. Gurung, P. Baccam, S. A. Stage, B. T. Suchoski, B. A. Prakash, B. Adhikari, J. Cui, A. Rodríguez, A. Tabassum, J. Xie, P. Keskinocak, J. Asplund, A. Baxter, B. E. Oruc, N. Serban, S. O. Arik, M. Dusenberry, A. Epshteyn, E. Kanal, L. T. Le, C.-L. Li, T. Pfister, D. Sava, R. Sinha, T. Tsai, N. Yoder, J. Yoon, L. Zhang, S. Abbott, N. I. Bosse, S. Funk, J. Hellewell, S. R. Meakin, K. Sherratt, M. Zhou, R. Kalantari, T. K. Yamana, S. Pei, J. Shaman, M. L. Li, D. Bertsimas, O. S. Lami, S. Soni, H. T. Bouardi, T. Ayer, M. Adee, J. Chhatwal, O. O. Dalgic, M. A. Ladd, B. P. Linas, P. Mueller, J. Xiao, Y. Wang, Q. Wang, S. Xie, D. Zeng, A. Green, J. Bien, L. Brooks, A. J. Hu, M. Jahja, D. M. Donald, B. Narasimhan, C. Politsch, S. Rajanala, A. Rumack, N. Simon, R. J. Tibshirani, R. Tibshirani, V. Ventura, L. Wasserman, E. B. O’Dea, J. M. Drake, R. Pagano, Q. T. Tran, L. S. T. Ho, H. Huynh, J. W. Walker, R. B. Slayton, M. A. Johansson, M. Biggerstaff, N. G. Reich, Evaluation of individual and ensemble probabilistic forecasts of covid-19 mortality in the United States. Proc. Natl. Acad. Sci. U.S.A. 119, e2113561119 (2022). 35394862 10.1073/pnas.2113561119 PMC9169655

[53] E. L. Ray, L. C. Brooks, J. Bien, M. Biggerstaff, N. I. Bosse, J. Bracher, E. Y. Cramer, S. Funk, A. Gerding, M. A. Johansson, A. Rumack, Y. Wang, M. Zorn, R. J. Tibshirani, N. G. Reich, Comparing trained and untrained probabilistic ensemble forecasts of covid-19 cases and deaths in the United States. arXiv:2201.12387 [stat.ME] (28 January 2022). 10.1016/j.ijforecast.2022.06.005 PMC9247236 35791416

[54] E. Y. Cramer, Y. Huang, Y. Wang, E. L. Ray, M. Cornell, J. Bracher, A. Brennen, A. J. Castero Rivadeneira, A. Gerding, K. House, D. Jayawardena, A. H. Kanji, A. Khandelwal, K. Le, J. Niemi, A. Stark, A. Shah, N. Wattanchit; M. W. Zorn, Nicholas G Reich on behalf of the US COVID-19 Forecast Hub Consortium, The United States covid-19 forecast hub dataset. medRxiv 2021.11.04.21265886 (2021). 10.1101/2021.11.04.21265886.

[55] K. V. Parag, Improved estimation of time-varying reproduction numbers at low case incidence and between epidemic waves. PLOS Comput. Biol. 17, 1–23 (2021). 10.1371/journal.pcbi.1009347 PMC8448340 34492011

[56] S. Yang, S. C. Kou, F. Lu, J. S. Brownstein, N. Brooke, M. Santillana, Advances in using internet searches to track dengue. PLOS Comput. Biol. 13, e1005607 (2017). 28727821 10.1371/journal.pcbi.1005607 PMC5519005

[57] A. B. Lawson, K. Kleinman, *Spatial and Syndromic Surveillance for Public Health* (John Wiley & Sons, 2005).

[58] E. Surkova, V. Nikolayevskyy, F. Drobniewski, False-positive covid-19 results: Hidden problems and costs. Lancet Respir. Med. 8, 1167–1168 (2020). 33007240 10.1016/S2213-2600(20)30453-7 PMC7524437

[59] H. J. T. Unwin, S. Mishra, V. C. Bradley, A. Gandy, T. A. Mellan, H. Coupland, J. Ish-Horowicz, M. A. C. Vollmer, C. Whittaker, S. L. Filippi, X. Xi, M. Monod, O. Ratmann, M. Hutchinson, F. Valka, H. Zhu, I. Hawryluk, P. Milton, K. E. C. Ainslie, M. Baguelin, A. Boonyasiri, N. F. Brazeau, L. Cattarino, Z. Cucunuba, G. Cuomo-Dannenburg, I. Dorigatti, O. D. Eales, J. W. Eaton, S. L. van Elsland, R. G. F. John, K. A. M. Gaythorpe, W. Green, W. Hinsley, B. Jeffrey, E. Knock, D. J. Laydon, J. Lees, G. Nedjati-Gilani, P. Nouvellet, L. Okell, K. V. Parag, I. Siveroni, H. A. Thompson, P. Walker, C. E. Walters, O. J. Watson, L. K. Whittles, A. C. Ghani, N. M. Ferguson, S. Riley, C. A. Donnelly, S. Bhatt, S. Flaxman, State-level tracking of covid-19 in the United States. Nat. Commun. 11, 1–9 (2020). 33273462 10.1038/s41467-020-19652-6 PMC7712910

[60] B. J. Cowling, M. S. Lau, L.-M. Ho, S.-K. Chuang, T. Tsang, S.-H. Liu, P.-Y. Leung, S.-V. Lo, E. H. Lau, The effective reproduction number of pandemic influenza: Prospective estimation. Epidemiology 21, 842–846 (2010). 20805752 10.1097/EDE.0b013e3181f20977 PMC3084966

[61] K. Baltrusaitis, J. S. Brownstein, S. V. Scarpino, E. Bakota, A. W. Crawley, G. Conidi, J. Gunn, J. Gray, A. Zink, M. Santillana, Comparison of crowd-sourced, electronic health records based, and traditional health-care based influenza-tracking systems at multiple spatial resolutions in the United States of America. BMC Infect. Dis. 18, 1–8 (2018). 30111305 10.1186/s12879-018-3322-3 PMC6094455

[62] E. L. Aiken, A. T. Nguyen, C. Viboud, M. Santillana, Toward the use of neural networks for influenza prediction at multiple spatial resolutions. Sci. Adv. 7, eabb1237 (2021). 34134985 10.1126/sciadv.abb1237 PMC8208709

[63] A. Cori, N. M. Ferguson, C. Fraser, S. Cauchemez, A new framework and software to estimate time-varying reproduction numbers during epidemics. Am. J. Epidemiol. 178, 1505–1512 (2013). 24043437 10.1093/aje/kwt133 PMC3816335

[64] K. V. Parag, R. N. Thompson, C. A. Donnelly, Are epidemic growth rates more informative than reproduction numbers? medRxiv 2021.04.15.21255565 (2021). 10.1101/2021.04.15.21255565. PMC9347870 35942192

[65] F. Dablander, H. Heesterbeek, D. Borsboom, J. M. Drake, Overlapping timescales obscure early warning signals of the second covid-19 wave. Proc. R. Soc. B 289, 20211809 (2022). 10.1098/rspb.2021.1809 PMC8825995 35135355

[66] D. A. O’brien, C. F. Clements, Early warning signal reliability varies with covid-19 waves. Biol. Lett. 17, 20210487 (2021). 34875183 10.1098/rsbl.2021.0487 PMC8651412

[67] K. V. Parag, C. A. Donnelly, Fundamental limits on inferring epidemic resurgence in real time using effective reproduction numbers. PLOS Comput. Biol. 18, e1010004 (2022). 35404936 10.1371/journal.pcbi.1010004 PMC9022826

[68] A. Rovetta, Reliability of Google trends: Analysis of the limits and potential of web infoveillance during covid-19 pandemic and for future research. Front. Res. Metr. Anal. 6, 28 (2021). 10.3389/frma.2021.670226 PMC8186442 34113751

[69] S. F. Ardabili, A. Mosavi, P. Ghamisi, F. Ferdinand, A. R. Varkonyi-Koczy, U. Reuter, T. Rabczuk, P. M. Atkinson, Covid-19 outbreak prediction with machine learning. Algorithms 13, 249 (2020).

[70] Y. Mohamadou, A. Halidou, P. T. Kapen, A review of mathematical modeling, artificial intelligence and datasets used in the study, prediction and management of covid-19. Appl. Intell. 50, 3913–3925 (2020). 10.1007/s10489-020-01770-9 PMC7335662 34764546

[71] E. Dong, H. Du, L. Gardner, An interactive web-based dashboard to track covid-19 in real time. Lancet Infect. Dis. 20, 533–534 (2020). 32087114 10.1016/S1473-3099(20)30120-1 PMC7159018

[72] N. M. Ferguson, D. Laydon, G. Nedjati-Gilani, N. Imai, K. Ainslie, M. Baguelin, S. Bhatia, A. Boonyasiri, Z. Cucunubá, G. Cuomo-Dannenburg, A. Dighe, I. Dorigatti, H. Fu, K. Gaythorpe, W. Green, A. Hamlet, W. Hinsley, L. C. Okell, S. van Elsland, H. Thompson, R. Verity, E. Volz, H. Wang, Y. Wang, P. G. Walker, C. Walters, P. Winskill, C. Whittaker, C. A. Donnelly, S. Riley, A. C. Ghani, Impact of non-pharmaceutical interventions (npis) to reduce covid-19 mortality and healthcare demand. J. R. Soc. Med. , 1–20 (2020).

[73] D. W. Marion, J. F. Dashe, Pacing the diaphragm: Patient selection, evaluation, implantation, and complications. UpToDate, Waltham, MA [accessed 4 January 2018].

[74] K. M. Gostic, L. McGough, E. B. Baskerville, S. Abbott, K. Joshi, C. Tedijanto, R. Kahn, R. Niehus, J. A. Hay, P. M. De Salazar, J. Hellewell, S. Meakin, J. D. Munday, N. I. Bosse, K. Sherrat, R. N. Thompson, L. F. White, J. S. Huisman, J. Scire, S. Bonhoeffer, T. Stadler, J. Wallinga, S. Funk, M. Lipsitch, S. Cobey, Practical considerations for measuring the effective reproductive number *Rt*. PLOS Comput. Biol. 16, 1–21 (2020). 10.1371/journal.pcbi.1008409 PMC7728287 33301457

[75] K. V. Parag, C. A. Donnelly, Fundamental limits on inferring epidemic resurgence in real time. medRxiv 2021.09.08.21263270 (2021). 10.1101/2021.09.08.21263270. PMC9022826 35404936

