# Supplementary material for: Using digital traces to build prospective and real-time county-level early warning systems to anticipate COVID-19 outbreaks in the United States
Source: Sci Adv. 2023 Jan 18;9(3):eabq0199. doi: 10.1126/sciadv.abq0199 (PMC9848273; doi:10.1126/sciadv.abq0199)
Supplement: Supplementary file 1 — Figs. S1 to S286 Tables S1 to S9 [file sciadv.abq0199_sm.pdf]

Science Advances
AAAS

Supplementary Materials for

Using digital traces to build prospective and real-time county-level early warning systems to anticipate COVID-19 outbreaks in the United States

Lucas M. Stolerman *et al.*

Corresponding author: Mauricio Santillana, m.santillana@northeastern.edu

Sci. Adv. **9**, eabq0199 (2023)
DOI: 10.1126/sciadv.abq0199

This PDF file includes:

Figs. S1 to S286
Tables S1 to S9

Figures S1-S49.
The probability of resurgence P(Rt > 1) (in green) and the different events generated by the Naive (gray), Single Source (blue) and Multiple Source methods (orange) at both county and state level.

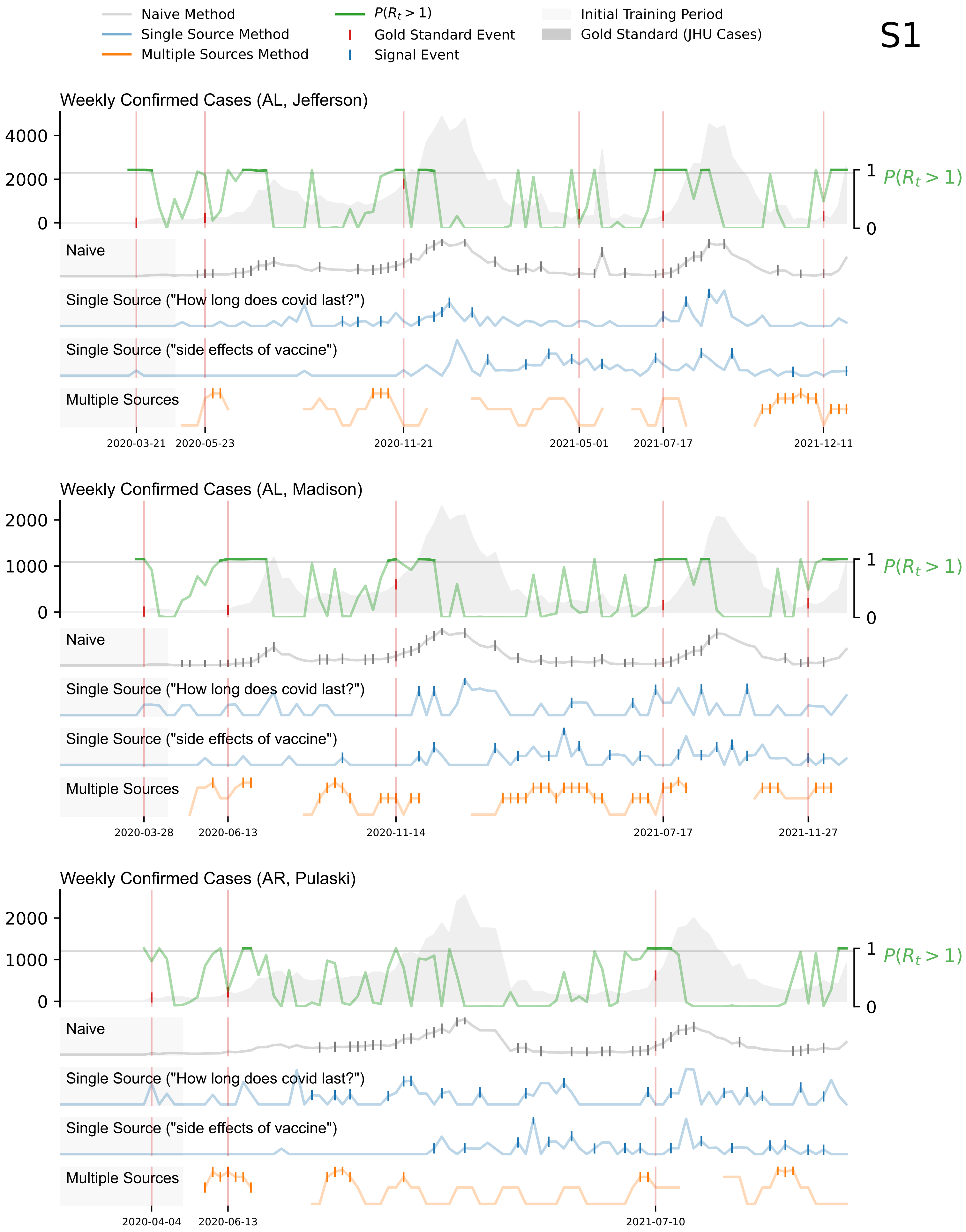
S1
Naive Method
Single Source Method
Multiple Sources Method
$P(R_t > 1)$
Gold Standard Event
Signal Event
Initial Training Period
Gold Standard (JHU Cases)
Weekly Confirmed Cases (AL, Jefferson)
Naive
Single Source ("How long does covid last?")
Single Source ("side effects of vaccine")
Multiple Sources
2020-03-21
2020-05-23
2020-11-21
2021-05-01
2021-07-17
2021-12-11
Weekly Confirmed Cases (AL, Madison)
Naive
Single Source ("How long does covid last?")
Single Source ("side effects of vaccine")
Multiple Sources
2020-03-28
2020-06-13
2020-11-14
2021-07-17
2021-11-27
Weekly Confirmed Cases (AR, Pulaski)
Naive
Single Source ("How long does covid last?")
Single Source ("side effects of vaccine")
Multiple Sources
2020-04-04
2020-06-13
2021-07-10

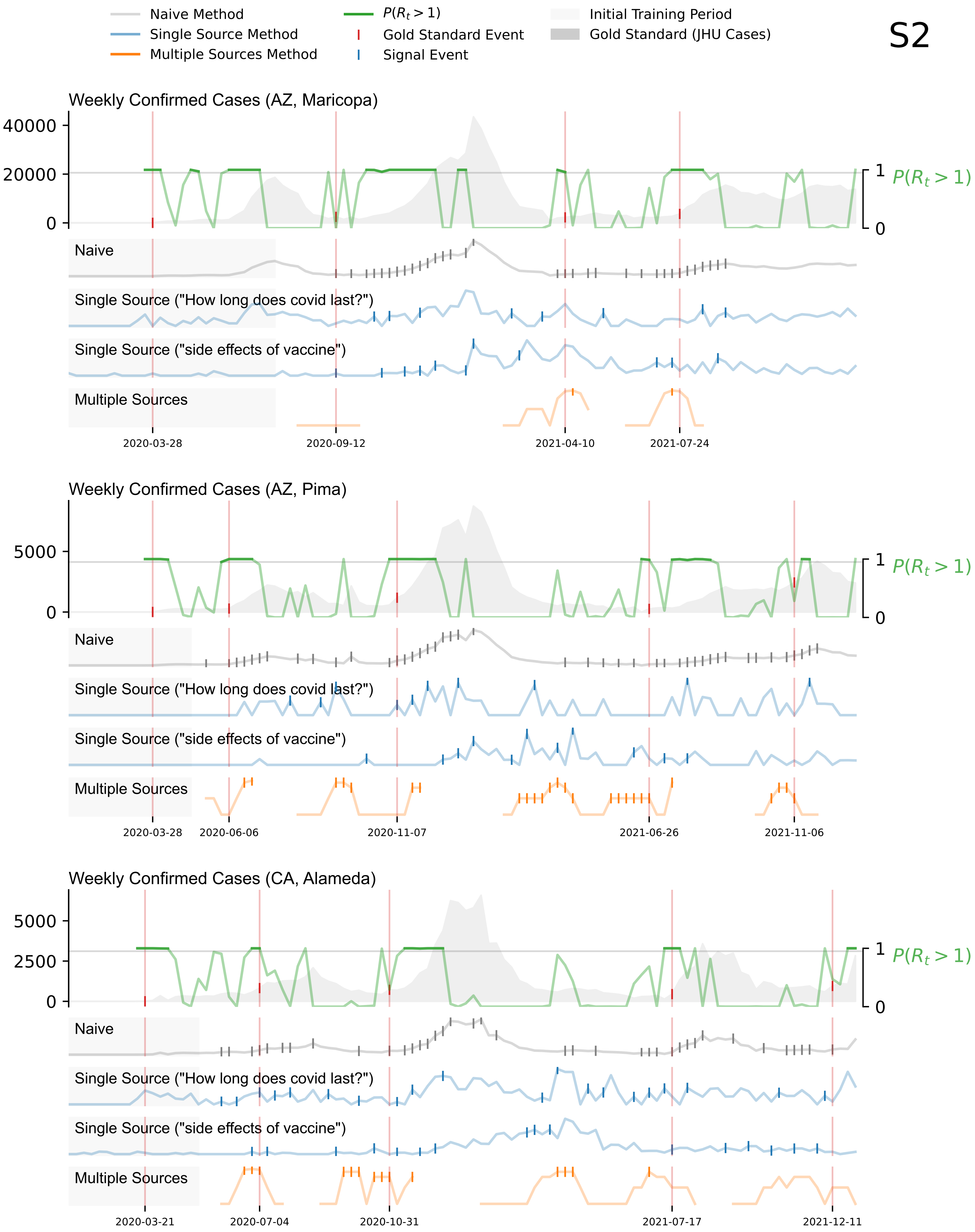

S2
Naive Method
Single Source Method
Multiple Sources Method
$P(R_t > 1)$
Gold Standard Event
Signal Event
Initial Training Period
Gold Standard (JHU Cases)
Weekly Confirmed Cases (AZ, Maricopa)
40000
20000
0
1
0
$P(R_t > 1)$
Naive
Single Source ("How long does covid last?")
Single Source ("side effects of vaccine")
Multiple Sources
2020-03-28
2020-09-12
2021-04-10
2021-07-24
Weekly Confirmed Cases (AZ, Pima)
5000
0
1
0
$P(R_t > 1)$
Naive
Single Source ("How long does covid last?")
Single Source ("side effects of vaccine")
Multiple Sources
2020-03-28
2020-06-06
2020-11-07
2021-06-26
2021-11-06
Weekly Confirmed Cases (CA, Alameda)
5000
2500
0
1
0
$P(R_t > 1)$
Naive
Single Source ("How long does covid last?")
Single Source ("side effects of vaccine")
Multiple Sources
2020-03-21
2020-07-04
2020-10-31
2021-07-17
2021-12-11

S3

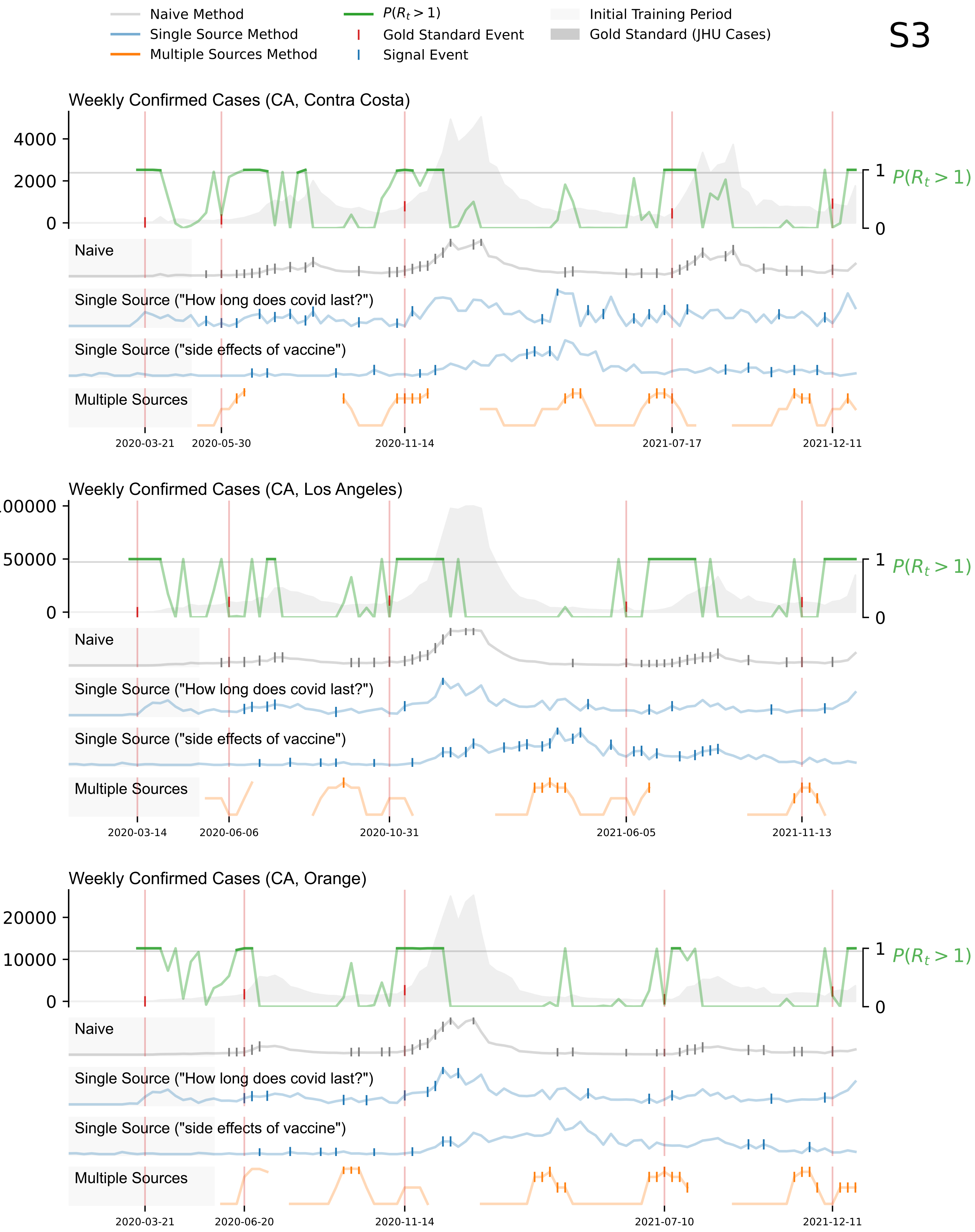

Naive Method
Single Source Method
Multiple Sources Method
$P(R_t > 1)$
Gold Standard Event
Signal Event
Initial Training Period
Gold Standard (JHU Cases)
Weekly Confirmed Cases (CA, Contra Costa)
4000
2000
0
1
0
Naive
Single Source ("How long does covid last?")
Single Source ("side effects of vaccine")
Multiple Sources
2020-03-21
2020-05-30
2020-11-14
2021-07-17
2021-12-11
Weekly Confirmed Cases (CA, Los Angeles)
50000
0
2020-03-14
2020-06-06
2020-10-31
2021-06-05
2021-11-13
Weekly Confirmed Cases (CA, Orange)
20000
10000
2020-03-21
2020-06-20
2020-11-14
2021-07-10
2021-12-11

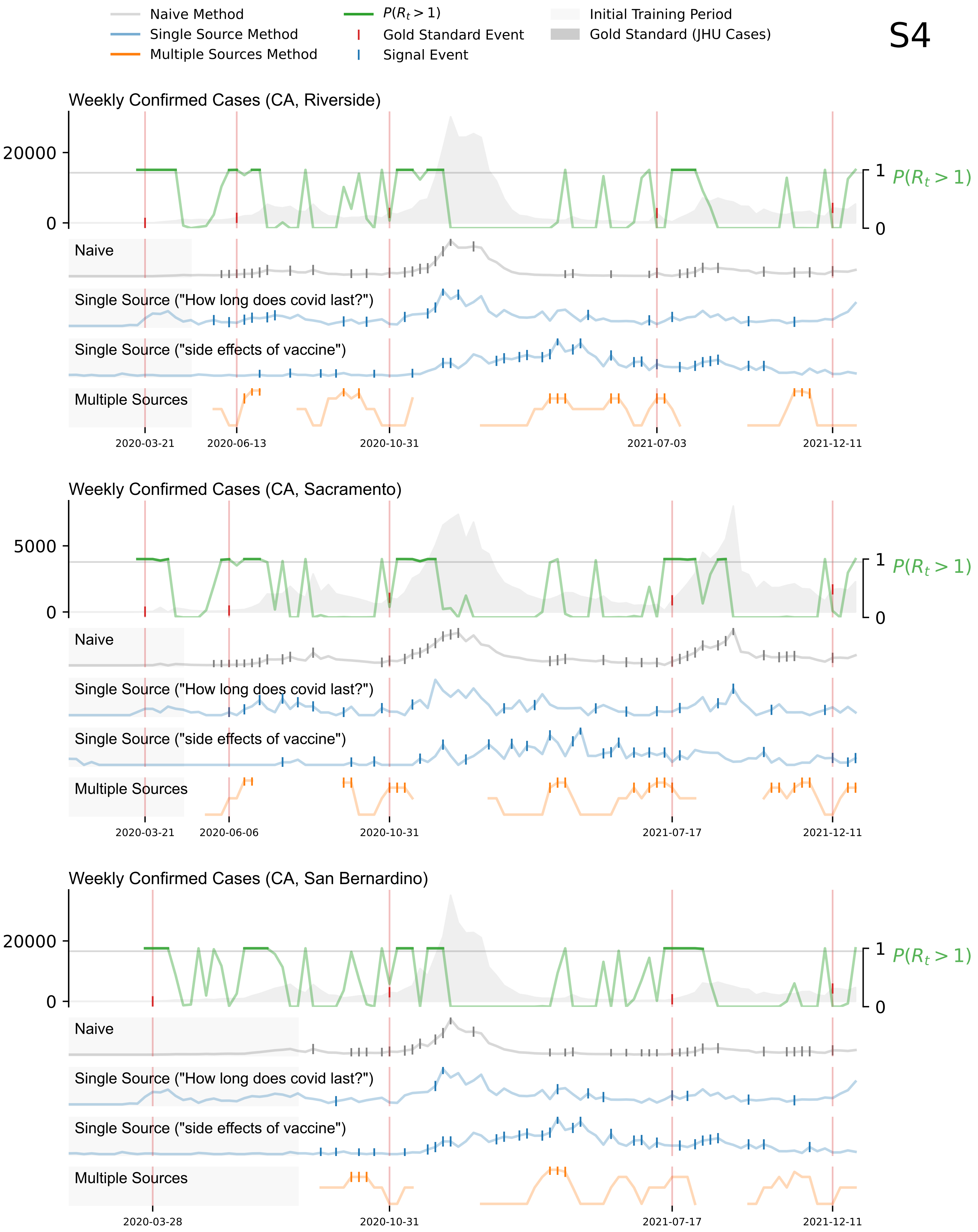

Naive Method
Single Source Method
Multiple Sources Method
$P(R_t > 1)$
Gold Standard Event
Signal Event
Initial Training Period
Gold Standard (JHU Cases)
S4
Weekly Confirmed Cases (CA, Riverside)
20000
0
1
0
$P(R_t > 1)$
Naive
Single Source ("How long does covid last?")
Single Source ("side effects of vaccine")
Multiple Sources
2020-03-21
2020-06-13
2020-10-31
2021-07-03
2021-12-11
Weekly Confirmed Cases (CA, Sacramento)
5000
0
1
0
$P(R_t > 1)$
Naive
Single Source ("How long does covid last?")
Single Source ("side effects of vaccine")
Multiple Sources
2020-03-21
2020-06-06
2020-10-31
2021-07-17
2021-12-11
Weekly Confirmed Cases (CA, San Bernardino)
20000
0
1
0
$P(R_t > 1)$
Naive
Single Source ("How long does covid last?")
Single Source ("side effects of vaccine")
Multiple Sources
2020-03-28
2020-10-31
2021-07-17
2021-12-11

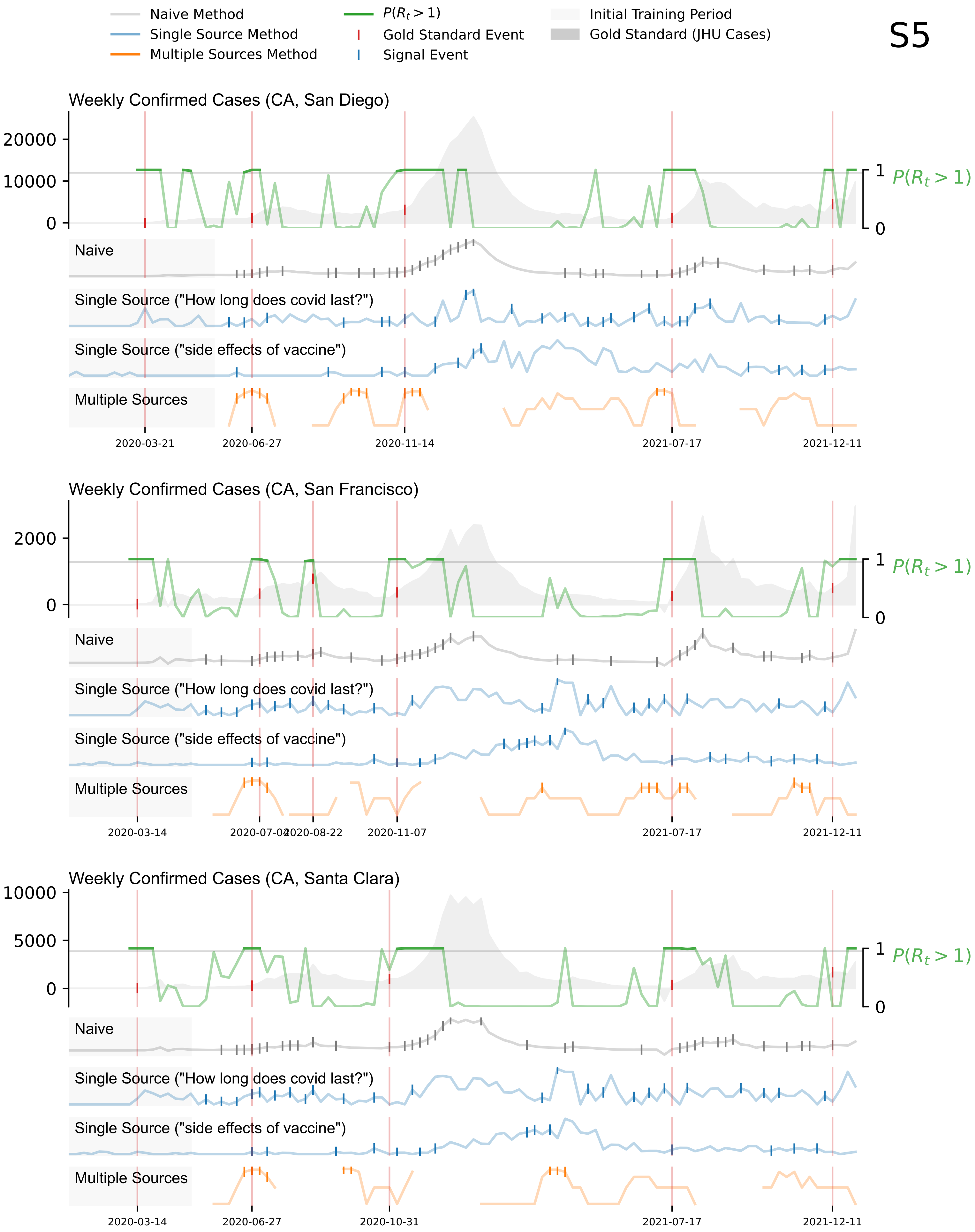

Naive Method
Single Source Method
Multiple Sources Method
$P(R_t > 1)$
Gold Standard Event
Signal Event
Initial Training Period
Gold Standard (JHU Cases)
S5
Weekly Confirmed Cases (CA, San Diego)
20000
10000
0
1
0
$P(R_t > 1)$
Naive
Single Source ("How long does covid last?")
Single Source ("side effects of vaccine")
Multiple Sources
2020-03-21
2020-06-27
2020-11-14
2021-07-17
2021-12-11
Weekly Confirmed Cases (CA, San Francisco)
2000
0
2020-03-14
2020-07-04
2020-08-22
2020-11-07
Weekly Confirmed Cases (CA, Santa Clara)
10000
5000
2020-10-31

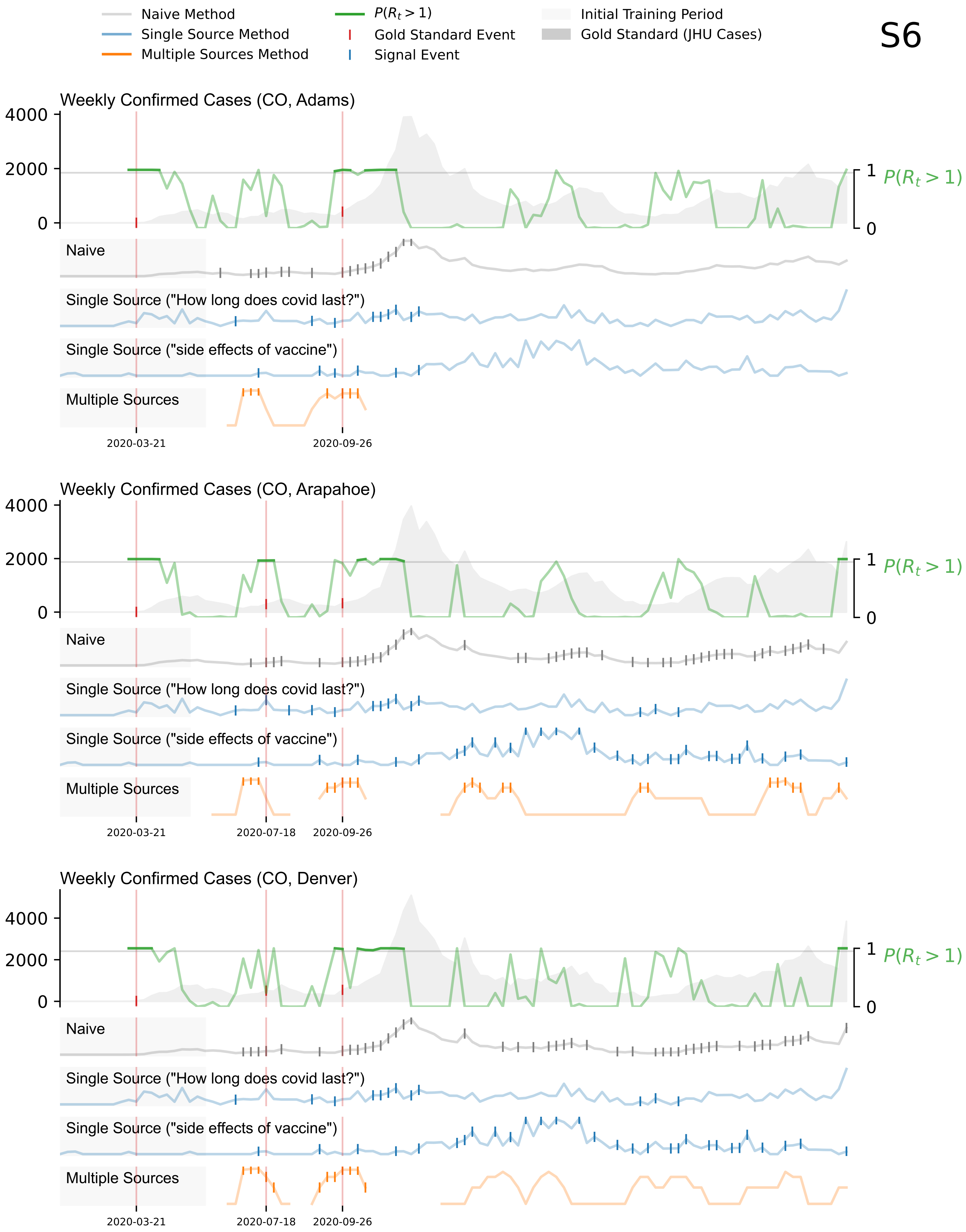

Naive Method
Single Source Method
Multiple Sources Method
$P(R_t > 1)$
Gold Standard Event
Signal Event
Initial Training Period
Gold Standard (JHU Cases)
S6
Weekly Confirmed Cases (CO, Adams)
4000
2000
0
1
0
$P(R_t > 1)$
Naive
Single Source ("How long does covid last?")
Single Source ("side effects of vaccine")
Multiple Sources
2020-03-21
2020-09-26
Weekly Confirmed Cases (CO, Arapahoe)
4000
2000
0
1
0
$P(R_t > 1)$
Naive
Single Source ("How long does covid last?")
Single Source ("side effects of vaccine")
Multiple Sources
2020-03-21
2020-07-18
2020-09-26
Weekly Confirmed Cases (CO, Denver)
4000
2000
0
1
0
$P(R_t > 1)$
Naive
Single Source ("How long does covid last?")
Single Source ("side effects of vaccine")
Multiple Sources
2020-03-21
2020-07-18
2020-09-26

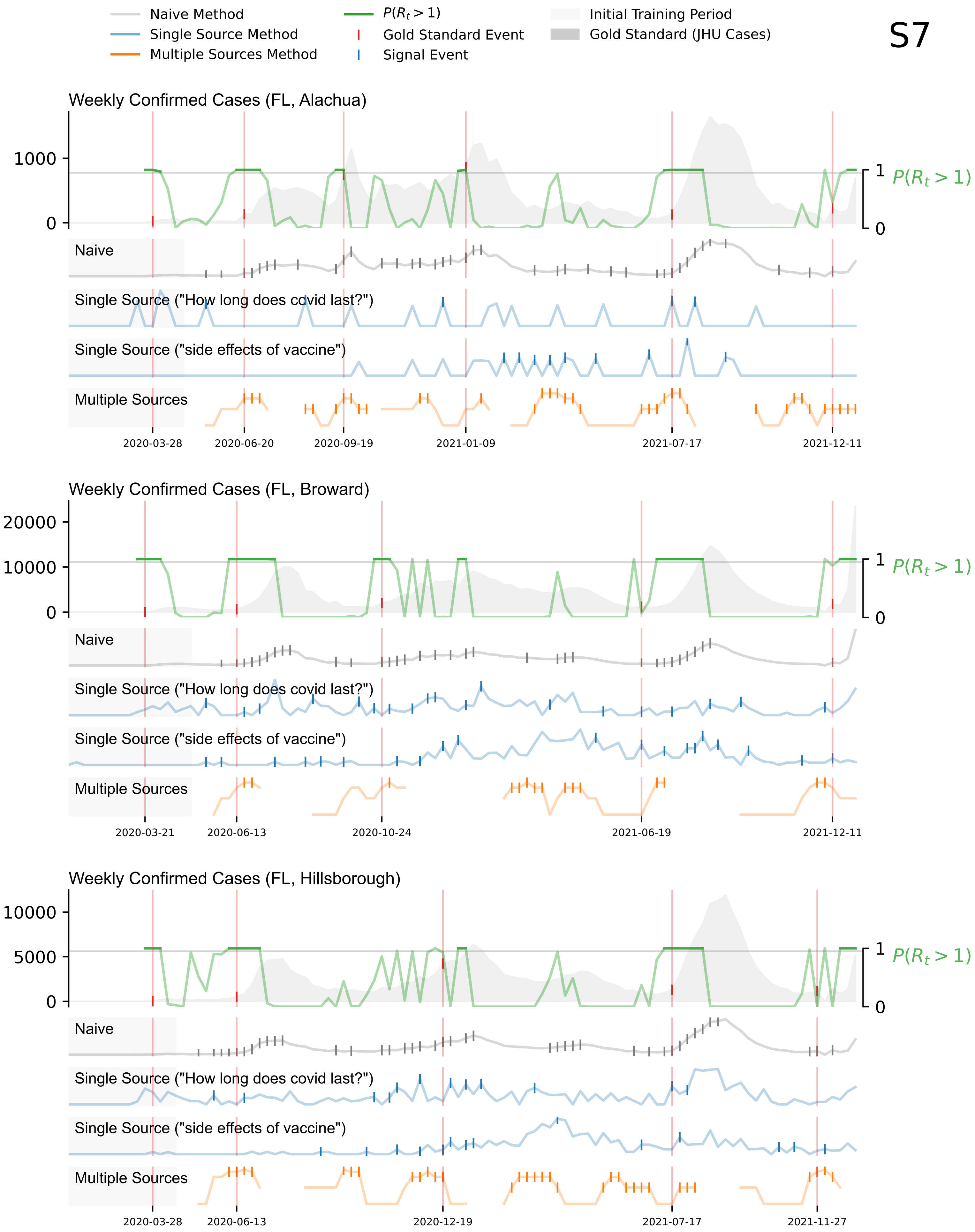

Naive Method
Single Source Method
Multiple Sources Method
$P(R_t > 1)$
Gold Standard Event
Signal Event
Initial Training Period
Gold Standard (JHU Cases)
S7
Weekly Confirmed Cases (FL, Alachua)
1000
0
1
$P(R_t > 1)$
0
Naive
Single Source ("How long does covid last?")
Single Source ("side effects of vaccine")
Multiple Sources
2020-03-28
2020-06-20
2020-09-19
2021-01-09
2021-07-17
2021-12-11
Weekly Confirmed Cases (FL, Broward)
20000
10000
0
1
$P(R_t > 1)$
0
Naive
Single Source ("How long does covid last?")
Single Source ("side effects of vaccine")
Multiple Sources
2020-03-21
2020-06-13
2020-10-24
2021-06-19
2021-12-11
Weekly Confirmed Cases (FL, Hillsborough)
10000
5000
0
1
$P(R_t > 1)$
0
Naive
Single Source ("How long does covid last?")
Single Source ("side effects of vaccine")
Multiple Sources
2020-03-28
2020-06-13
2020-12-19
2021-07-17
2021-11-27

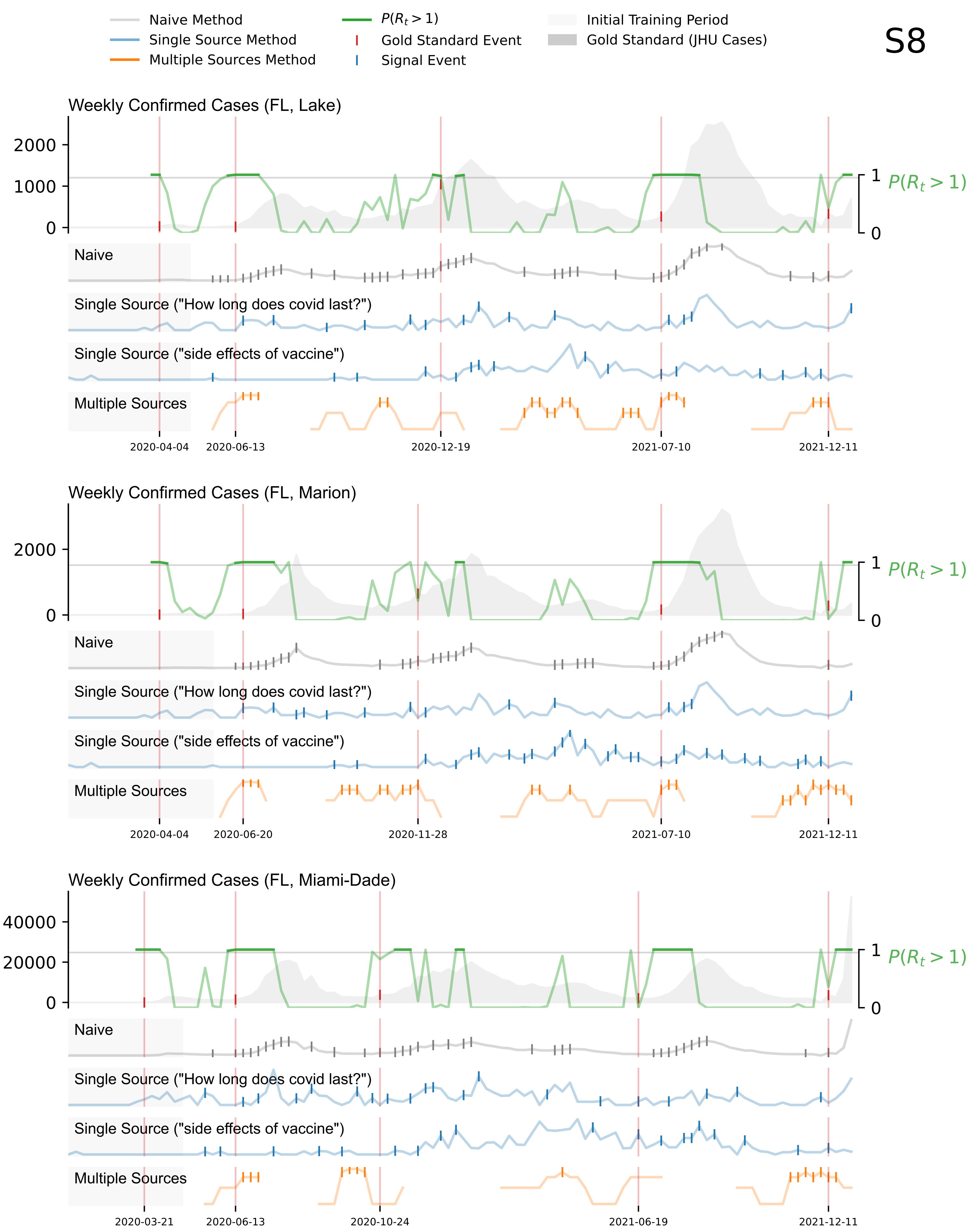

S8
Naive Method
Single Source Method
Multiple Sources Method
$P(R_t > 1)$
Gold Standard Event
Signal Event
Initial Training Period
Gold Standard (JHU Cases)
Weekly Confirmed Cases (FL, Lake)
2000
1000
0
1
0
$P(R_t > 1)$
Naive
Single Source ("How long does covid last?")
Single Source ("side effects of vaccine")
Multiple Sources
2020-04-04
2020-06-13
2020-12-19
2021-07-10
2021-12-11
Weekly Confirmed Cases (FL, Marion)
2000
0
1
0
$P(R_t > 1)$
Naive
Single Source ("How long does covid last?")
Single Source ("side effects of vaccine")
Multiple Sources
2020-04-04
2020-06-20
2020-11-28
2021-07-10
2021-12-11
Weekly Confirmed Cases (FL, Miami-Dade)
40000
20000
0
1
0
$P(R_t > 1)$
Naive
Single Source ("How long does covid last?")
Single Source ("side effects of vaccine")
Multiple Sources
2020-03-21
2020-06-13
2020-10-24
2021-06-19
2021-12-11

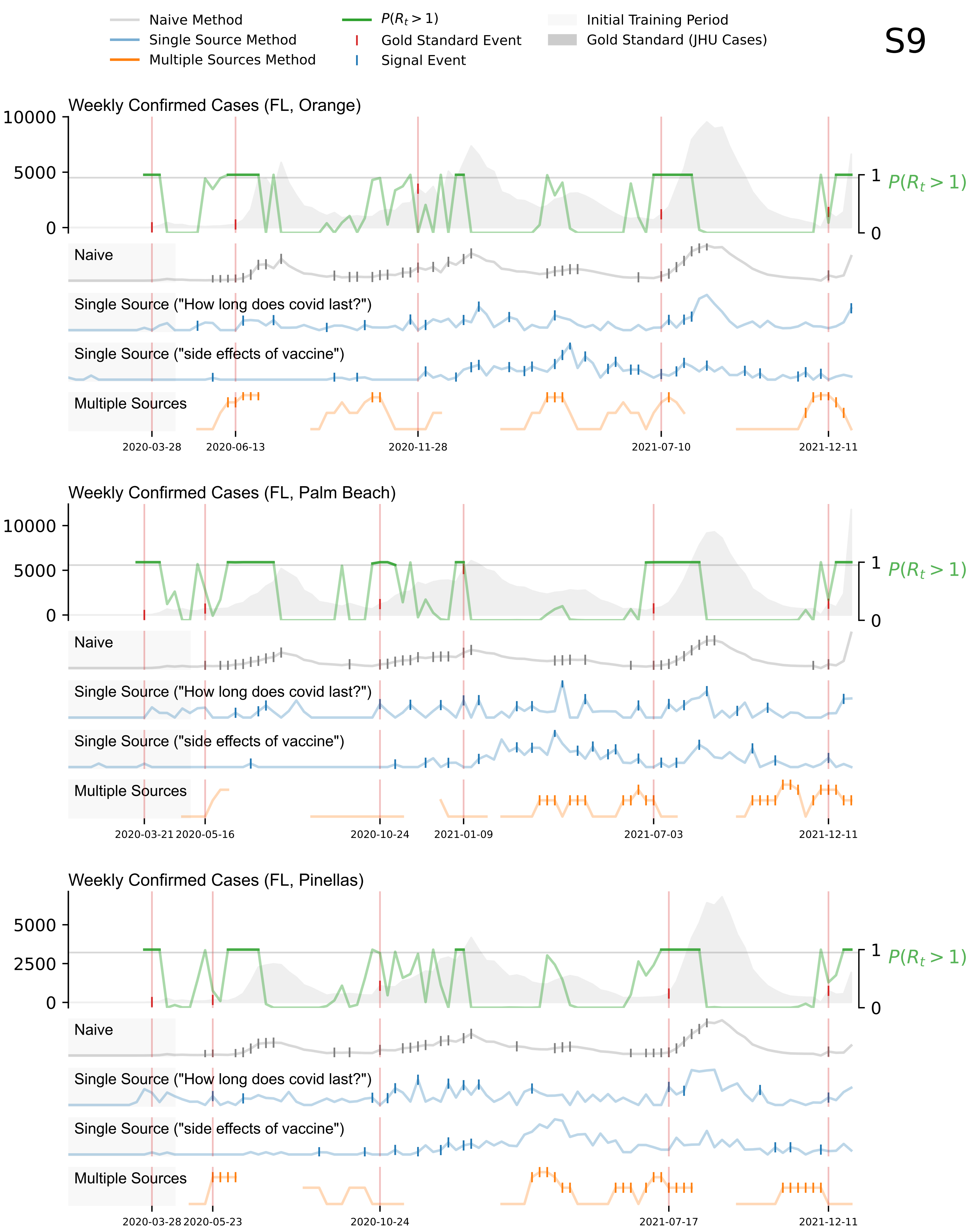

S9
Naive Method
Single Source Method
Multiple Sources Method
$P(R_t > 1)$
Gold Standard Event
Signal Event
Initial Training Period
Gold Standard (JHU Cases)
Weekly Confirmed Cases (FL, Orange)
Naive
Single Source ("How long does covid last?")
Single Source ("side effects of vaccine")
Multiple Sources
2020-03-28
2020-06-13
2020-11-28
2021-07-10
2021-12-11
Weekly Confirmed Cases (FL, Palm Beach)
2020-03-21
2020-05-16
2020-10-24
2021-01-09
2021-07-03
2021-12-11
Weekly Confirmed Cases (FL, Pinellas)
2020-03-28
2020-05-23
2020-10-24
2021-07-17
2021-12-11

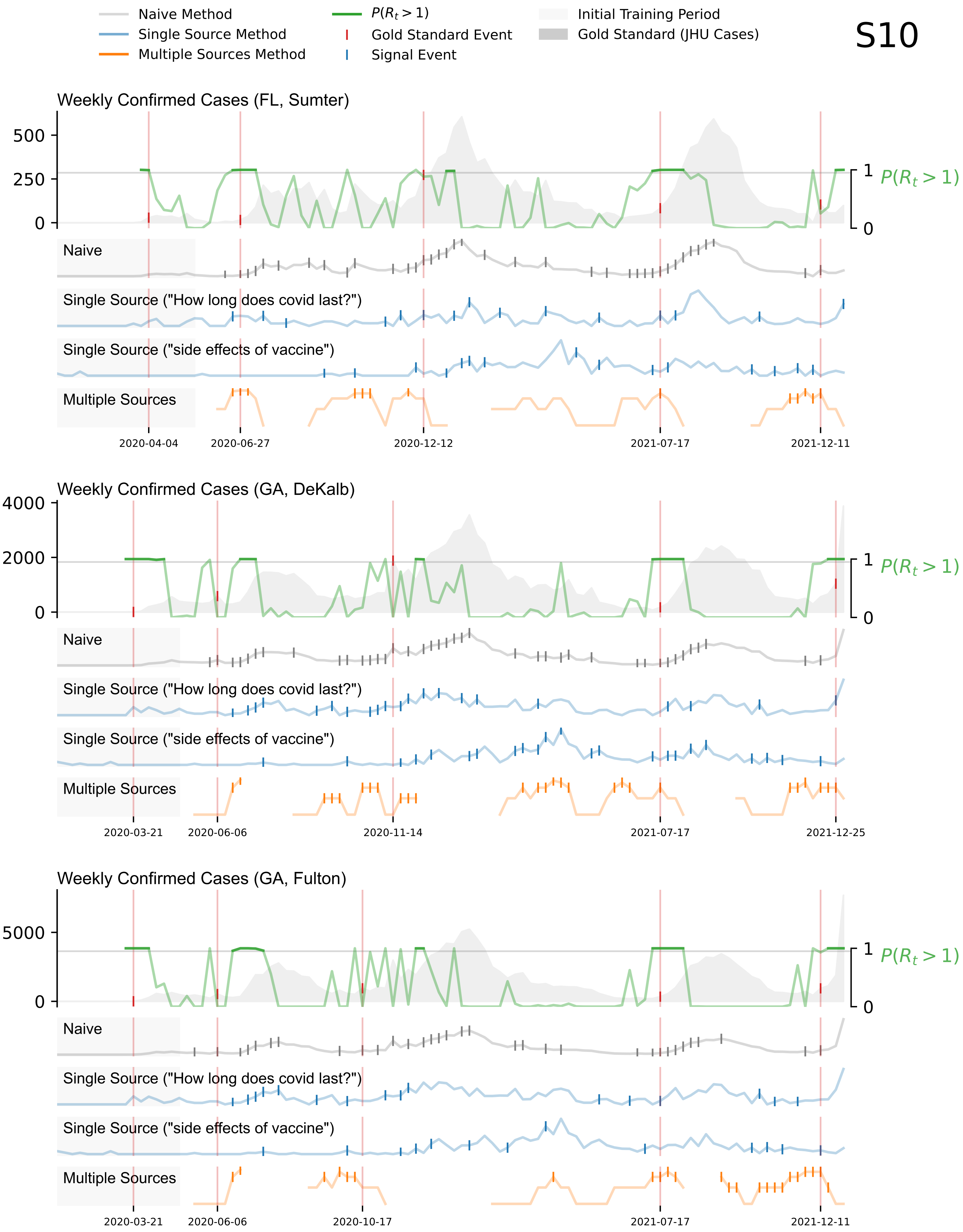

Naive Method
Single Source Method
Multiple Sources Method
$P(R_t > 1)$
Gold Standard Event
Signal Event
Initial Training Period
Gold Standard (JHU Cases)
S10
Weekly Confirmed Cases (FL, Sumter)
Naive
Single Source ("How long does covid last?")
Single Source ("side effects of vaccine")
Multiple Sources
2020-04-04
2020-06-27
2020-12-12
2021-07-17
2021-12-11
Weekly Confirmed Cases (GA, DeKalb)
Naive
Single Source ("How long does covid last?")
Single Source ("side effects of vaccine")
Multiple Sources
2020-03-21
2020-06-06
2020-11-14
2021-07-17
2021-12-25
Weekly Confirmed Cases (GA, Fulton)
Naive
Single Source ("How long does covid last?")
Single Source ("side effects of vaccine")
Multiple Sources
2020-03-21
2020-06-06
2020-10-17
2021-07-17
2021-12-11

S11

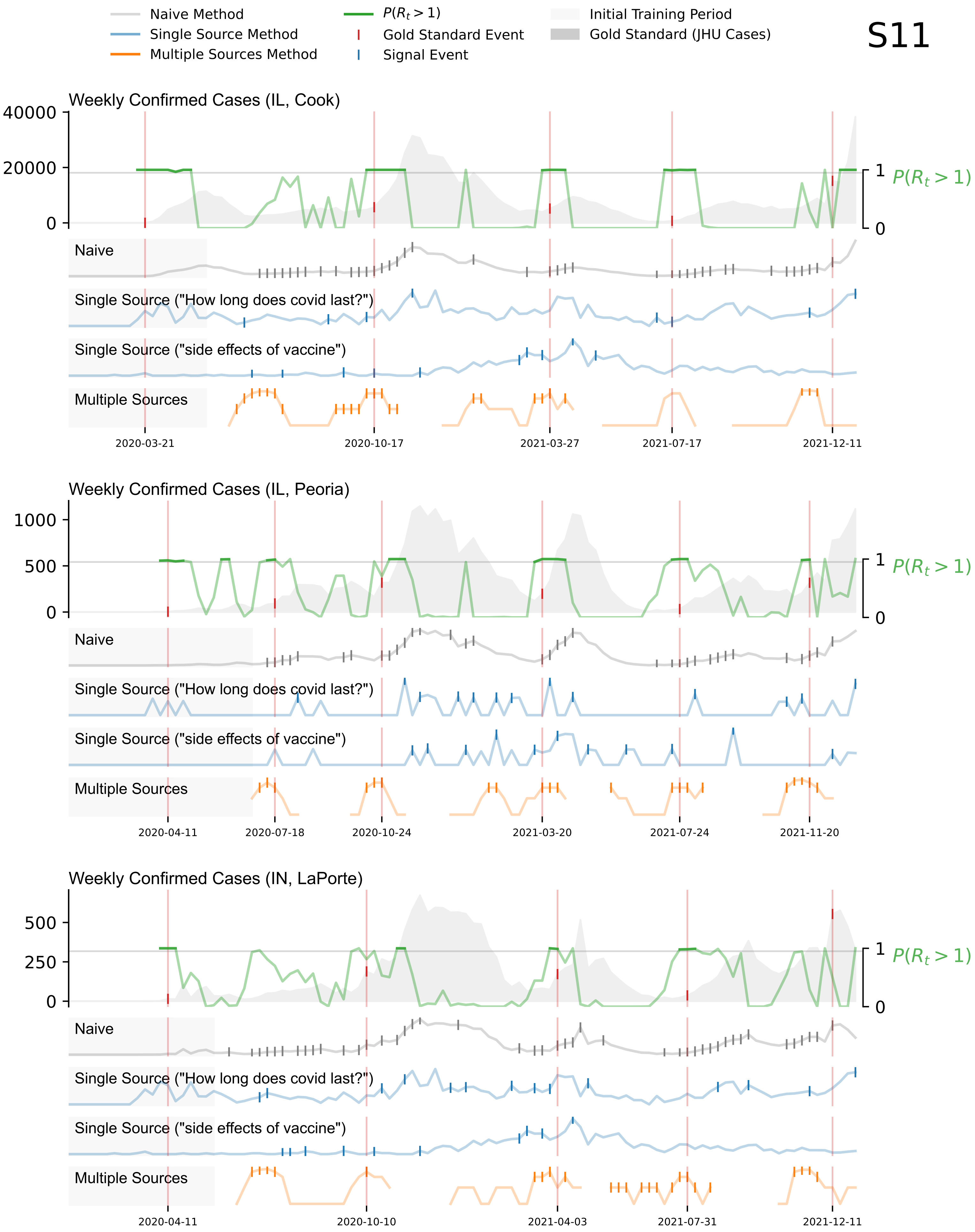

Naive Method
Single Source Method
Multiple Sources Method
$P(R_t > 1)$
Gold Standard Event
Signal Event
Initial Training Period
Gold Standard (JHU Cases)
Weekly Confirmed Cases (IL, Cook)
40000
20000
0
1
0
$P(R_t > 1)$
Naive
Single Source ("How long does covid last?")
Single Source ("side effects of vaccine")
Multiple Sources
2020-03-21
2020-10-17
2021-03-27
2021-07-17
2021-12-11
Weekly Confirmed Cases (IL, Peoria)
1000
500
0
1
0
$P(R_t > 1)$
Naive
Single Source ("How long does covid last?")
Single Source ("side effects of vaccine")
Multiple Sources
2020-04-11
2020-07-18
2020-10-24
2021-03-20
2021-07-24
2021-11-20
Weekly Confirmed Cases (IN, LaPorte)
500
250
0
1
0
$P(R_t > 1)$
Naive
Single Source ("How long does covid last?")
Single Source ("side effects of vaccine")
Multiple Sources
2020-04-11
2020-10-10
2021-04-03
2021-07-31
2021-12-11

S12

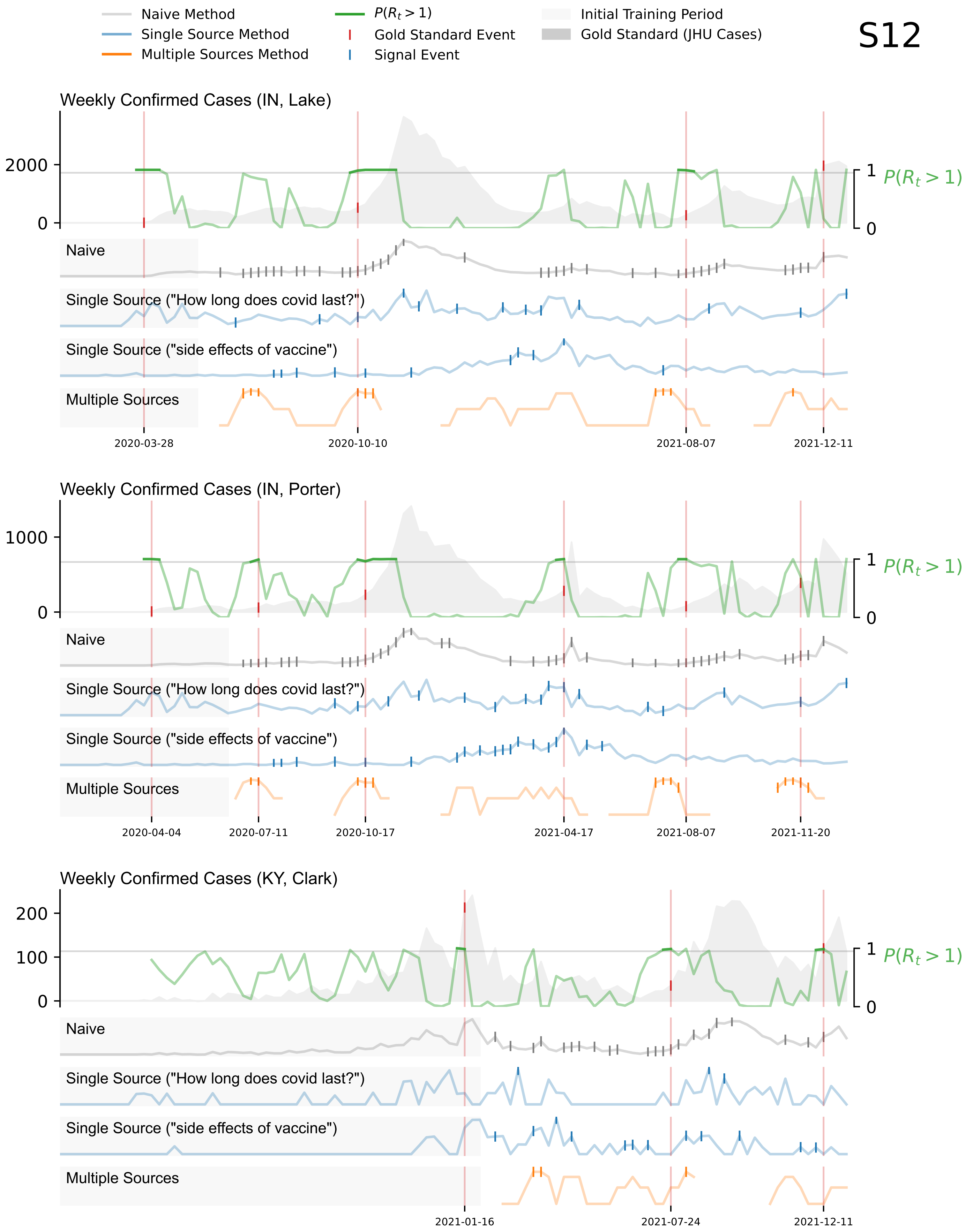

Naive Method
Single Source Method
Multiple Sources Method
$P(R_t > 1)$
Gold Standard Event
Signal Event
Initial Training Period
Gold Standard (JHU Cases)
Weekly Confirmed Cases (IN, Lake)
2000
0
1
0
$P(R_t > 1)$
Naive
Single Source ("How long does covid last?")
Single Source ("side effects of vaccine")
Multiple Sources
2020-03-28
2020-10-10
2021-08-07
2021-12-11
Weekly Confirmed Cases (IN, Porter)
1000
0
1
0
$P(R_t > 1)$
Naive
Single Source ("How long does covid last?")
Single Source ("side effects of vaccine")
Multiple Sources
2020-04-04
2020-07-11
2020-10-17
2021-04-17
2021-08-07
2021-11-20
Weekly Confirmed Cases (KY, Clark)
200
100
0
1
0
$P(R_t > 1)$
Naive
Single Source ("How long does covid last?")
Single Source ("side effects of vaccine")
Multiple Sources
2021-01-16
2021-07-24
2021-12-11

S13

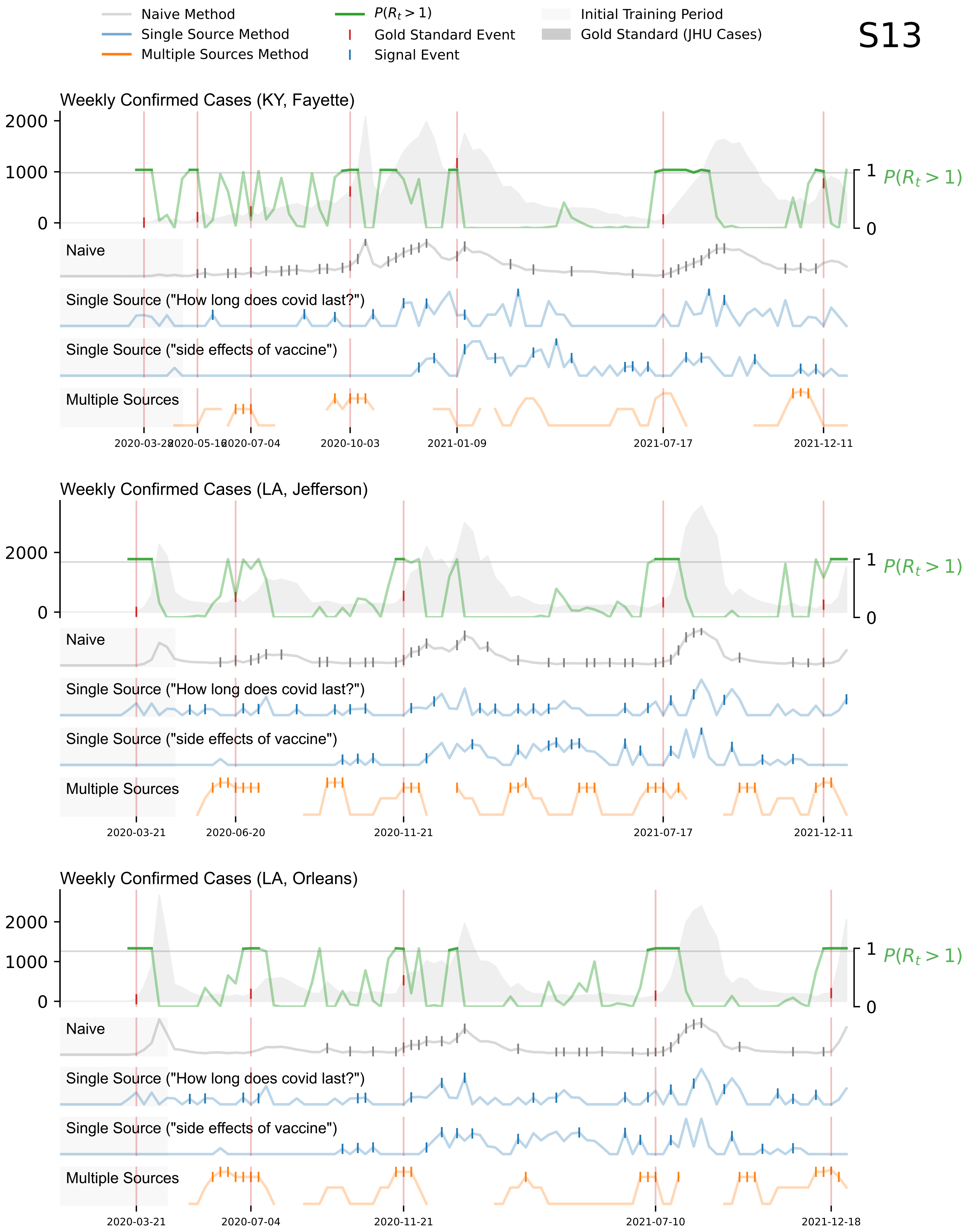
Naive Method
Single Source Method
Multiple Sources Method
$P(R_t > 1)$
Gold Standard Event
Signal Event
Initial Training Period
Gold Standard (JHU Cases)
Weekly Confirmed Cases (KY, Fayette)
Weekly Confirmed Cases (LA, Jefferson)
Weekly Confirmed Cases (LA, Orleans)
Naive
Single Source ("How long does covid last?")
Single Source ("side effects of vaccine")
Multiple Sources
2020-03-28
2020-05-16
2020-07-04
2020-10-03
2021-01-09
2021-07-17
2021-12-11
2020-03-21
2020-06-20
2020-11-21
2021-07-10
2021-12-18

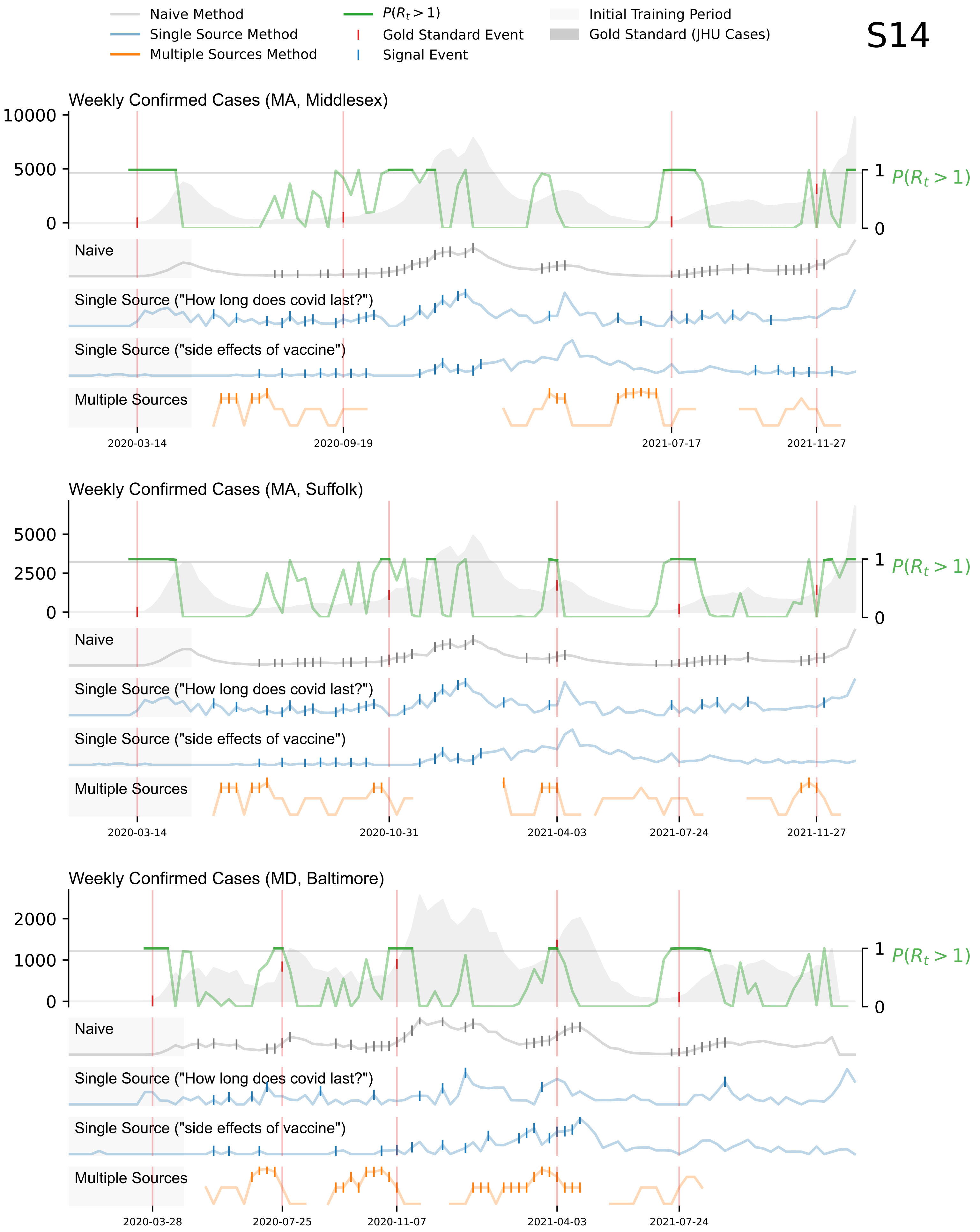

Naive Method
Single Source Method
Multiple Sources Method
$P(R_t > 1)$
Gold Standard Event
Signal Event
Initial Training Period
Gold Standard (JHU Cases)
S14
Weekly Confirmed Cases (MA, Middlesex)
10000
5000
0
1
0
$P(R_t > 1)$
Naive
Single Source ("How long does covid last?")
Single Source ("side effects of vaccine")
Multiple Sources
2020-03-14
2020-09-19
2021-07-17
2021-11-27
Weekly Confirmed Cases (MA, Suffolk)
5000
2500
0
1
0
$P(R_t > 1)$
Naive
Single Source ("How long does covid last?")
Single Source ("side effects of vaccine")
Multiple Sources
2020-03-14
2020-10-31
2021-04-03
2021-07-24
2021-11-27
Weekly Confirmed Cases (MD, Baltimore)
2000
1000
0
1
0
$P(R_t > 1)$
Naive
Single Source ("How long does covid last?")
Single Source ("side effects of vaccine")
Multiple Sources
2020-03-28
2020-07-25
2020-11-07
2021-04-03
2021-07-24

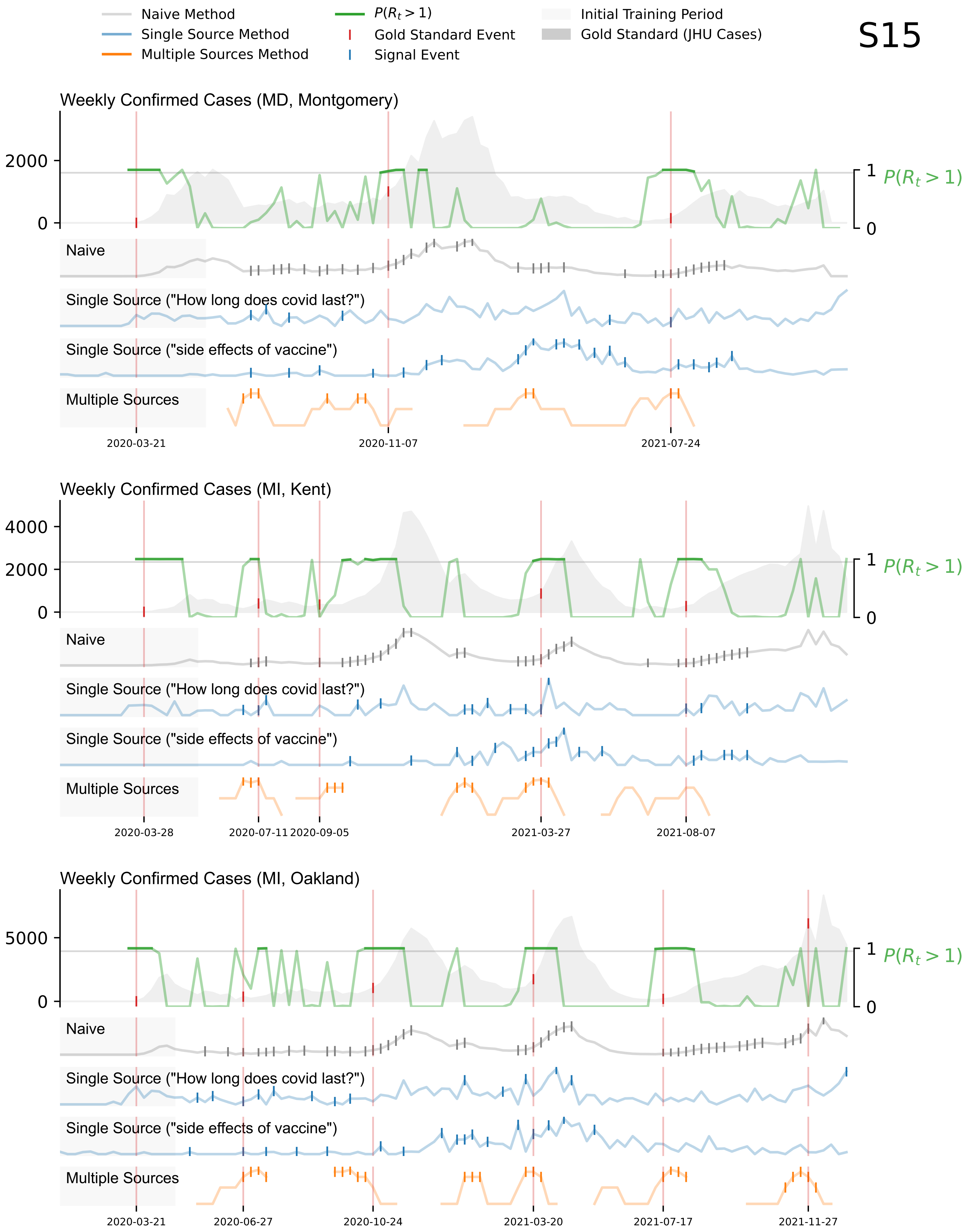

Naive Method
Single Source Method
Multiple Sources Method
$P(R_t > 1)$
Gold Standard Event
Signal Event
Initial Training Period
Gold Standard (JHU Cases)
S15
Weekly Confirmed Cases (MD, Montgomery)
Naive
Single Source ("How long does covid last?")
Single Source ("side effects of vaccine")
Multiple Sources
2020-03-21
2020-11-07
2021-07-24
Weekly Confirmed Cases (MI, Kent)
2020-03-28
2020-07-11
2020-09-05
2021-03-27
2021-08-07
Weekly Confirmed Cases (MI, Oakland)
2020-03-21
2020-06-27
2020-10-24
2021-03-20
2021-07-17
2021-11-27

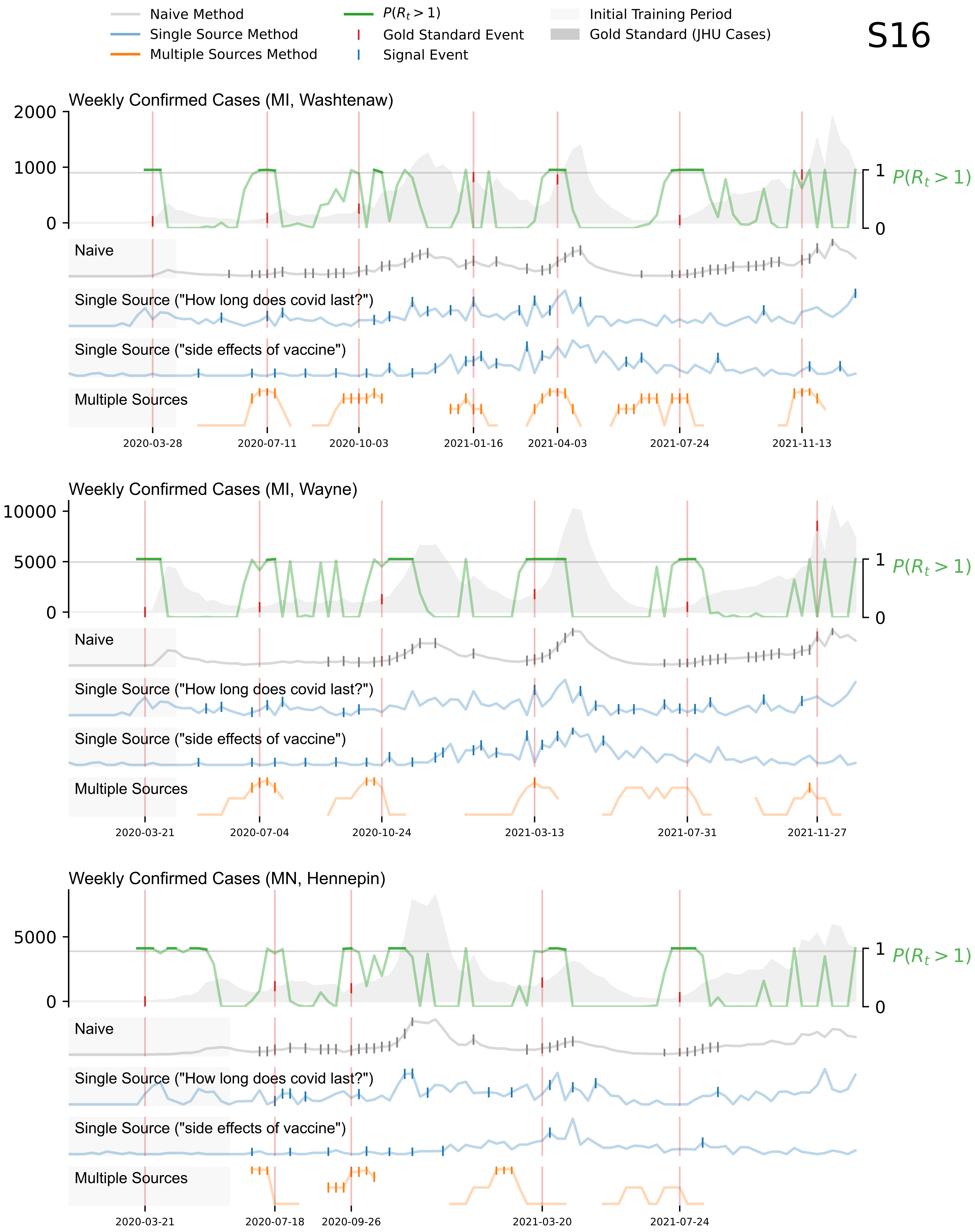

S16
Naive Method
Single Source Method
Multiple Sources Method
$P(R_t > 1)$
Gold Standard Event
Signal Event
Initial Training Period
Gold Standard (JHU Cases)
Weekly Confirmed Cases (MI, Washtenaw)
2000
1000
0
1
0
$P(R_t > 1)$
Naive
Single Source ("How long does covid last?")
Single Source ("side effects of vaccine")
Multiple Sources
2020-03-28
2020-07-11
2020-10-03
2021-01-16
2021-04-03
2021-07-24
2021-11-13
Weekly Confirmed Cases (MI, Wayne)
10000
5000
0
1
0
$P(R_t > 1)$
Naive
Single Source ("How long does covid last?")
Single Source ("side effects of vaccine")
Multiple Sources
2020-03-21
2020-07-04
2020-10-24
2021-03-13
2021-07-31
2021-11-27
Weekly Confirmed Cases (MN, Hennepin)
5000
0
1
0
$P(R_t > 1)$
Naive
Single Source ("How long does covid last?")
Single Source ("side effects of vaccine")
Multiple Sources
2020-03-21
2020-07-18
2020-09-26
2021-03-20
2021-07-24

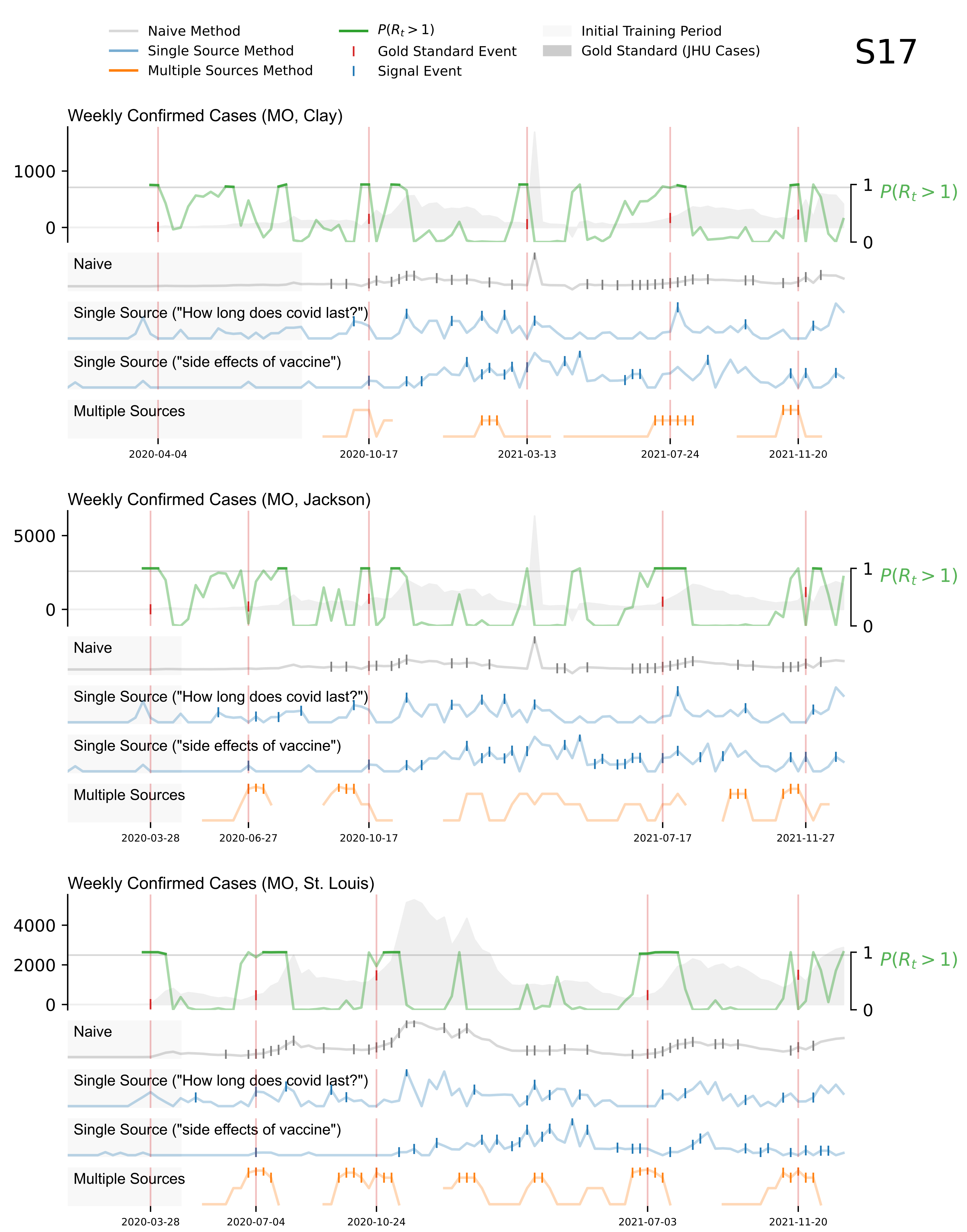

S17
Naive Method
Single Source Method
Multiple Sources Method
$P(R_t > 1)$
Gold Standard Event
Signal Event
Initial Training Period
Gold Standard (JHU Cases)
Weekly Confirmed Cases (MO, Clay)
1000
0
1
0
Naive
Single Source ("How long does covid last?")
Single Source ("side effects of vaccine")
Multiple Sources
2020-04-04
2020-10-17
2021-03-13
2021-07-24
2021-11-20
Weekly Confirmed Cases (MO, Jackson)
5000
0
2020-03-28
2020-06-27
2020-10-17
2021-07-17
2021-11-27
Weekly Confirmed Cases (MO, St. Louis)
4000
2000
0
2020-03-28
2020-07-04
2020-10-24
2021-07-03
2021-11-20

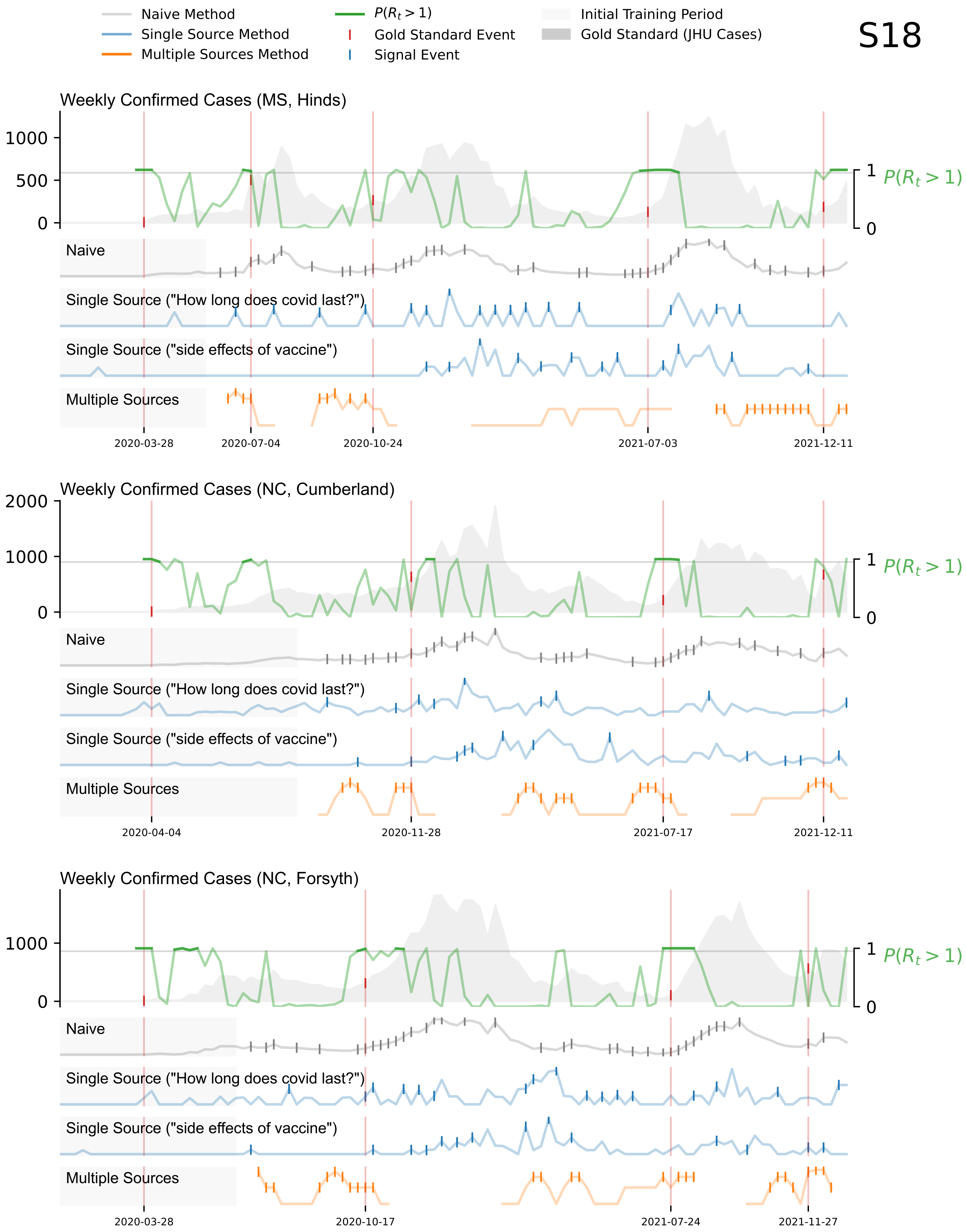

S18
Naive Method
Single Source Method
Multiple Sources Method
$P(R_t > 1)$
Gold Standard Event
Signal Event
Initial Training Period
Gold Standard (JHU Cases)
Weekly Confirmed Cases (MS, Hinds)
1000
500
0
1
0
$P(R_t > 1)$
Naive
Single Source ("How long does covid last?")
Single Source ("side effects of vaccine")
Multiple Sources
2020-03-28
2020-07-04
2020-10-24
2021-07-03
2021-12-11
Weekly Confirmed Cases (NC, Cumberland)
2000
1000
0
1
0
$P(R_t > 1)$
Naive
Single Source ("How long does covid last?")
Single Source ("side effects of vaccine")
Multiple Sources
2020-04-04
2020-11-28
2021-07-17
2021-12-11
Weekly Confirmed Cases (NC, Forsyth)
1000
0
1
0
$P(R_t > 1)$
Naive
Single Source ("How long does covid last?")
Single Source ("side effects of vaccine")
Multiple Sources
2020-03-28
2020-10-17
2021-07-24
2021-11-27

S19

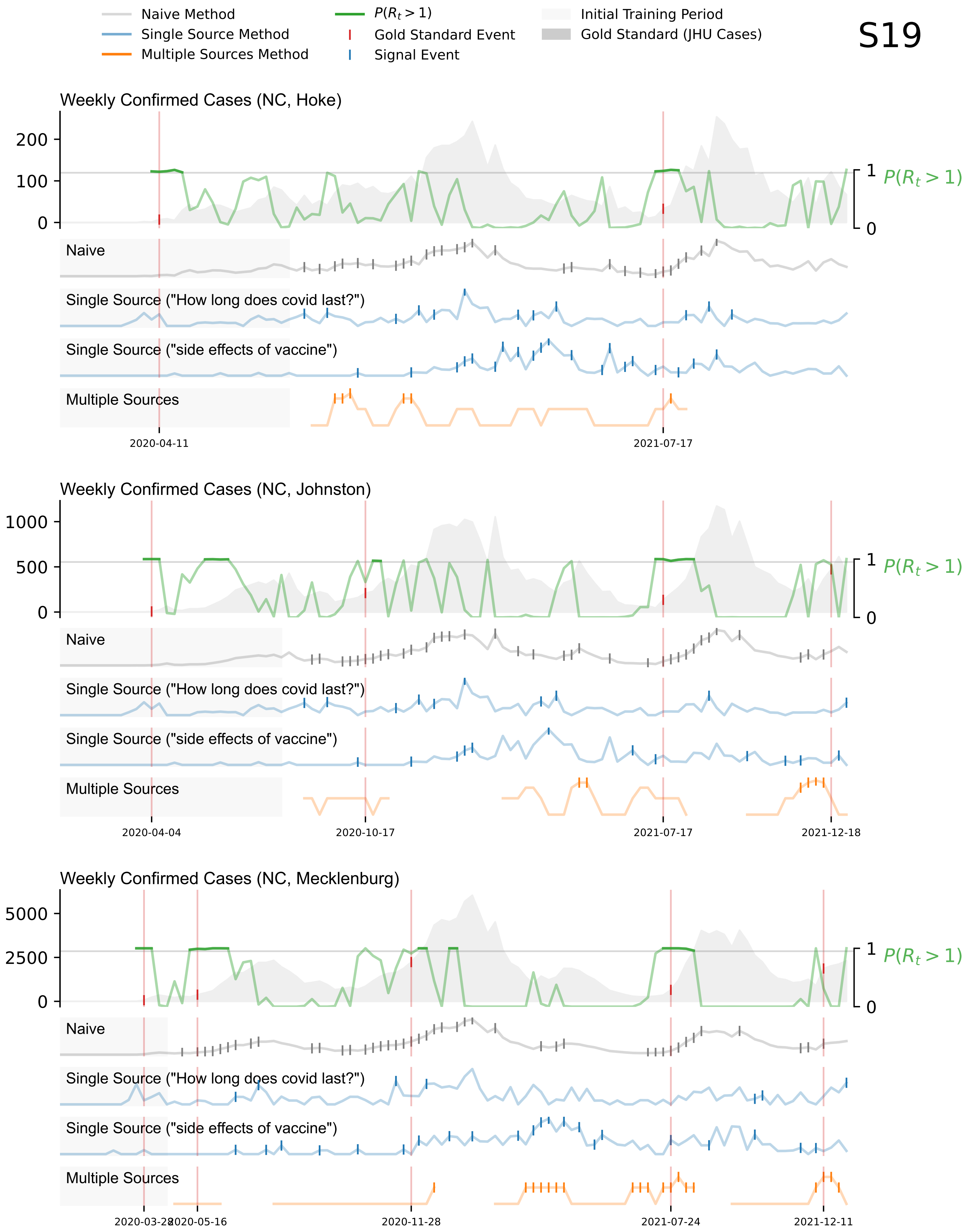

Naive Method
Single Source Method
Multiple Sources Method
$P(R_t > 1)$
Gold Standard Event
Signal Event
Initial Training Period
Gold Standard (JHU Cases)
Weekly Confirmed Cases (NC, Hoke)
200
100
0
1
0
$P(R_t > 1)$
Naive
Single Source ("How long does covid last?")
Single Source ("side effects of vaccine")
Multiple Sources
2020-04-11
2021-07-17
Weekly Confirmed Cases (NC, Johnston)
1000
500
0
1
0
$P(R_t > 1)$
Naive
Single Source ("How long does covid last?")
Single Source ("side effects of vaccine")
Multiple Sources
2020-04-04
2020-10-17
2021-07-17
2021-12-18
Weekly Confirmed Cases (NC, Mecklenburg)
5000
2500
0
1
0
$P(R_t > 1)$
Naive
Single Source ("How long does covid last?")
Single Source ("side effects of vaccine")
Multiple Sources
2020-03-28
2020-05-16
2020-11-28
2021-07-24
2021-12-11

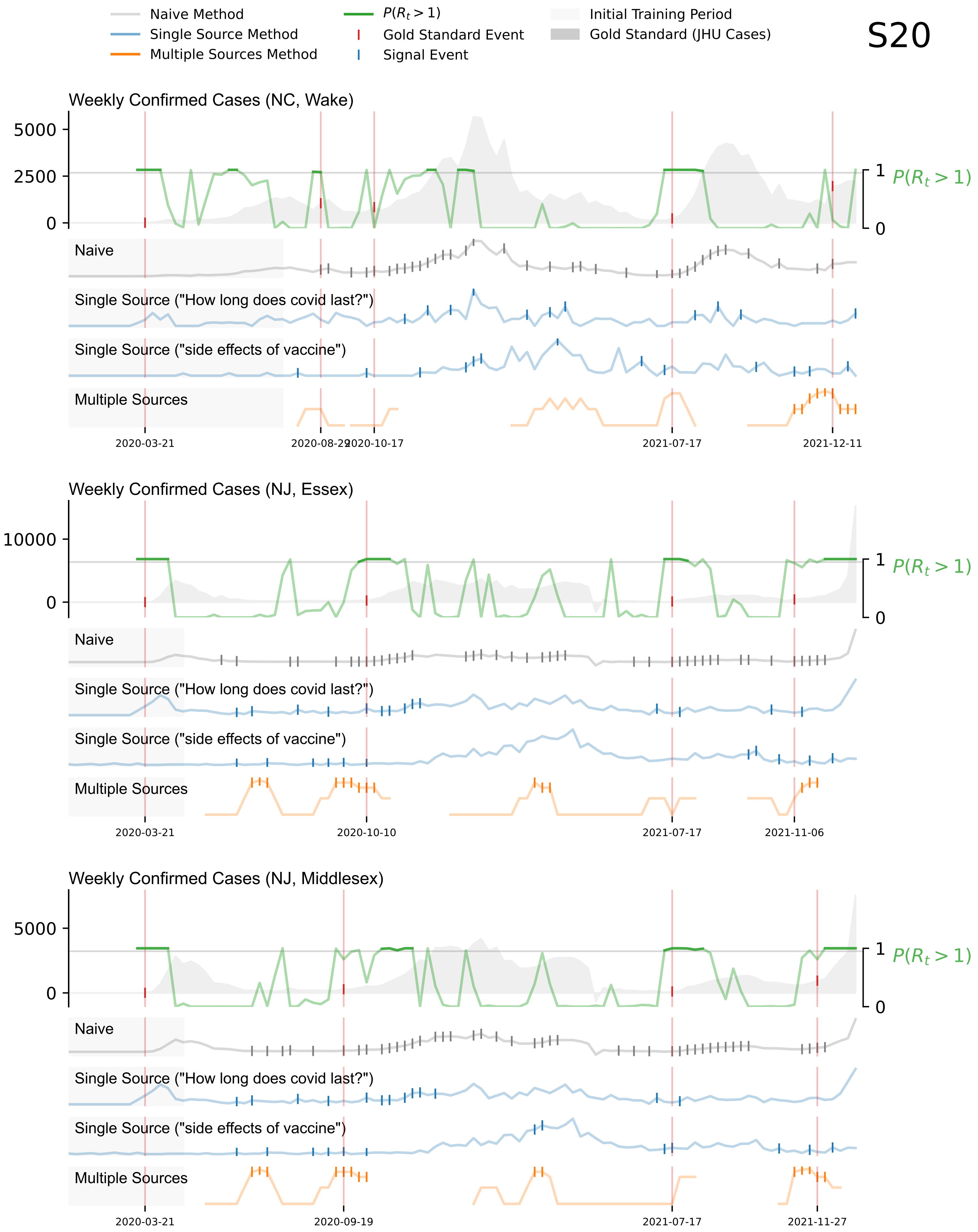
Naive Method
Single Source Method
Multiple Sources Method
$P(R_t > 1)$
Gold Standard Event
Signal Event
Initial Training Period
Gold Standard (JHU Cases)
S20
Weekly Confirmed Cases (NC, Wake)
5000
2500
0
1
0
$P(R_t > 1)$
Naive
Single Source ("How long does covid last?")
Single Source ("side effects of vaccine")
Multiple Sources
2020-03-21
2020-08-29
2020-10-17
2021-07-17
2021-12-11
Weekly Confirmed Cases (NJ, Essex)
10000
0
1
0
$P(R_t > 1)$
Naive
Single Source ("How long does covid last?")
Single Source ("side effects of vaccine")
Multiple Sources
2020-03-21
2020-10-10
2021-07-17
2021-11-06
Weekly Confirmed Cases (NJ, Middlesex)
5000
0
1
0
$P(R_t > 1)$
Naive
Single Source ("How long does covid last?")
Single Source ("side effects of vaccine")
Multiple Sources
2020-03-21
2020-09-19
2021-07-17
2021-11-27

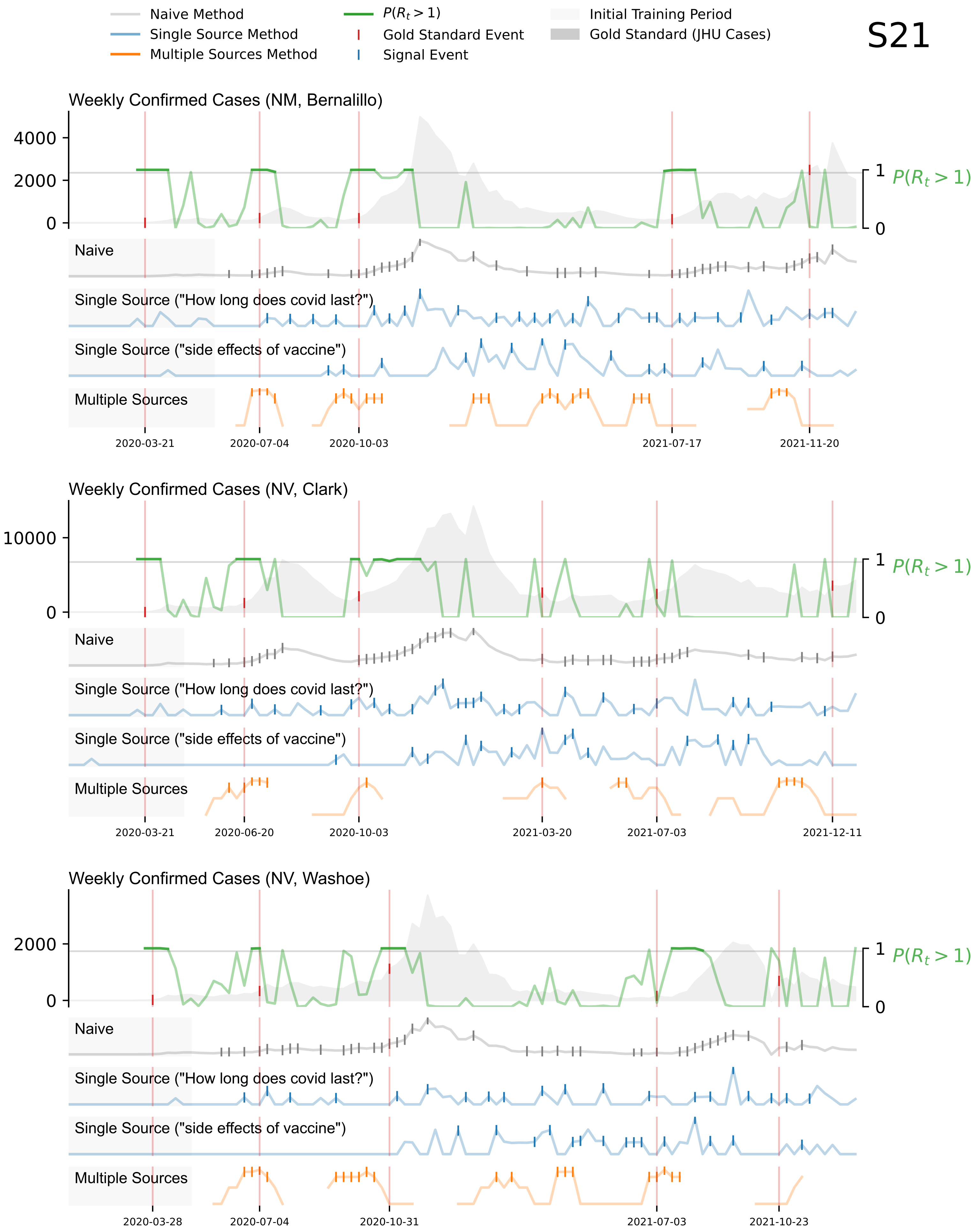

Naive Method
Single Source Method
Multiple Sources Method
$P(R_t > 1)$
Gold Standard Event
Signal Event
Initial Training Period
Gold Standard (JHU Cases)
S21
Weekly Confirmed Cases (NM, Bernalillo)
4000
2000
0
1
0
$P(R_t > 1)$
Naive
Single Source ("How long does covid last?")
Single Source ("side effects of vaccine")
Multiple Sources
2020-03-21
2020-07-04
2020-10-03
2021-07-17
2021-11-20
Weekly Confirmed Cases (NV, Clark)
10000
0
Naive
Single Source ("How long does covid last?")
Single Source ("side effects of vaccine")
Multiple Sources
2020-03-21
2020-06-20
2020-10-03
2021-03-20
2021-07-03
2021-12-11
Weekly Confirmed Cases (NV, Washoe)
2000
0
Naive
Single Source ("How long does covid last?")
Single Source ("side effects of vaccine")
Multiple Sources
2020-03-28
2020-07-04
2020-10-31
2021-07-03
2021-10-23

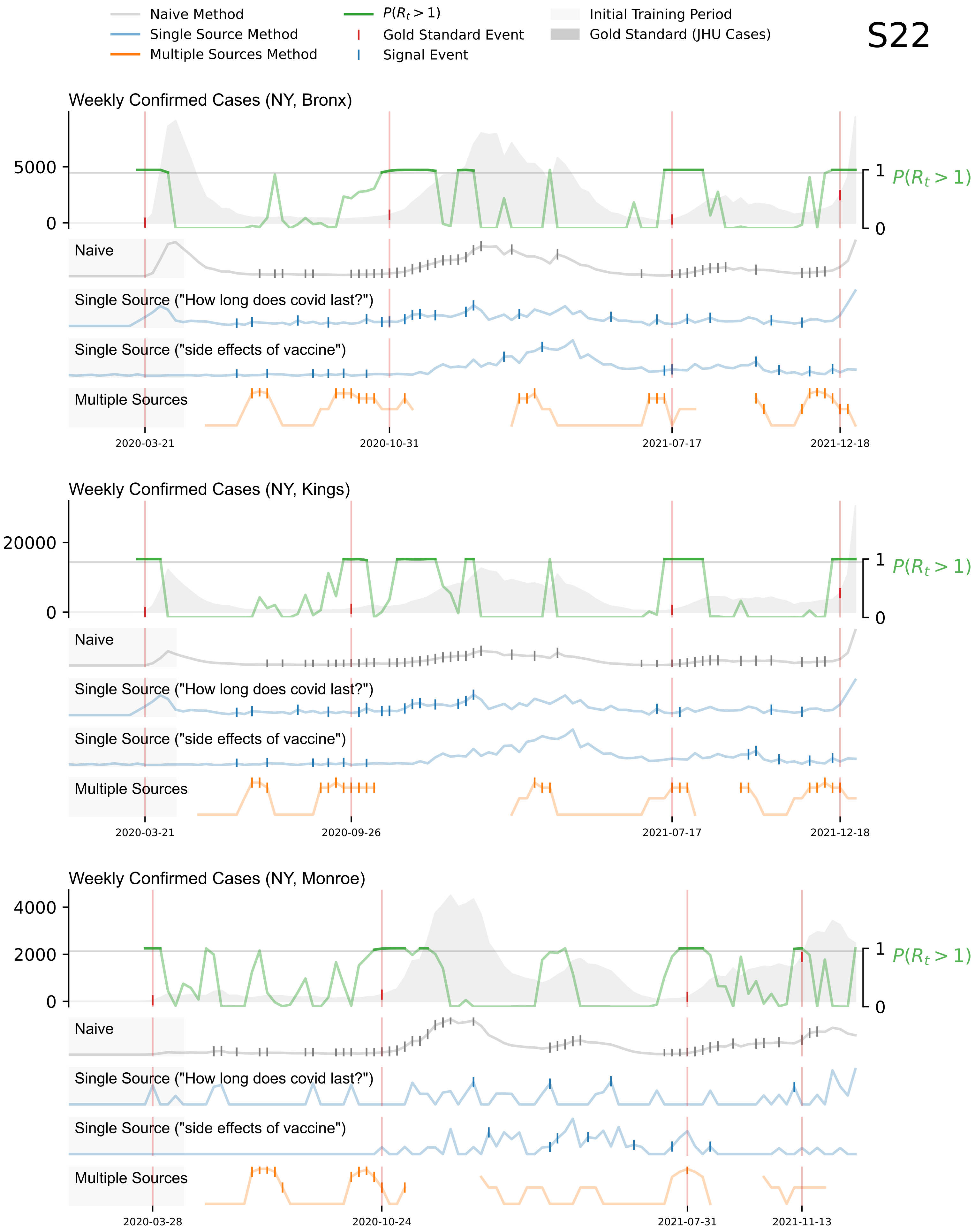

Naive Method
Single Source Method
Multiple Sources Method
$P(R_t > 1)$
Gold Standard Event
Signal Event
Initial Training Period
Gold Standard (JHU Cases)
S22
Weekly Confirmed Cases (NY, Bronx)
5000
0
1
0
$P(R_t > 1)$
Naive
Single Source ("How long does covid last?")
Single Source ("side effects of vaccine")
Multiple Sources
2020-03-21
2020-10-31
2021-07-17
2021-12-18
Weekly Confirmed Cases (NY, Kings)
20000
0
1
0
$P(R_t > 1)$
Naive
Single Source ("How long does covid last?")
Single Source ("side effects of vaccine")
Multiple Sources
2020-03-21
2020-09-26
2021-07-17
2021-12-18
Weekly Confirmed Cases (NY, Monroe)
4000
2000
0
1
0
$P(R_t > 1)$
Naive
Single Source ("How long does covid last?")
Single Source ("side effects of vaccine")
Multiple Sources
2020-03-28
2020-10-24
2021-07-31
2021-11-13

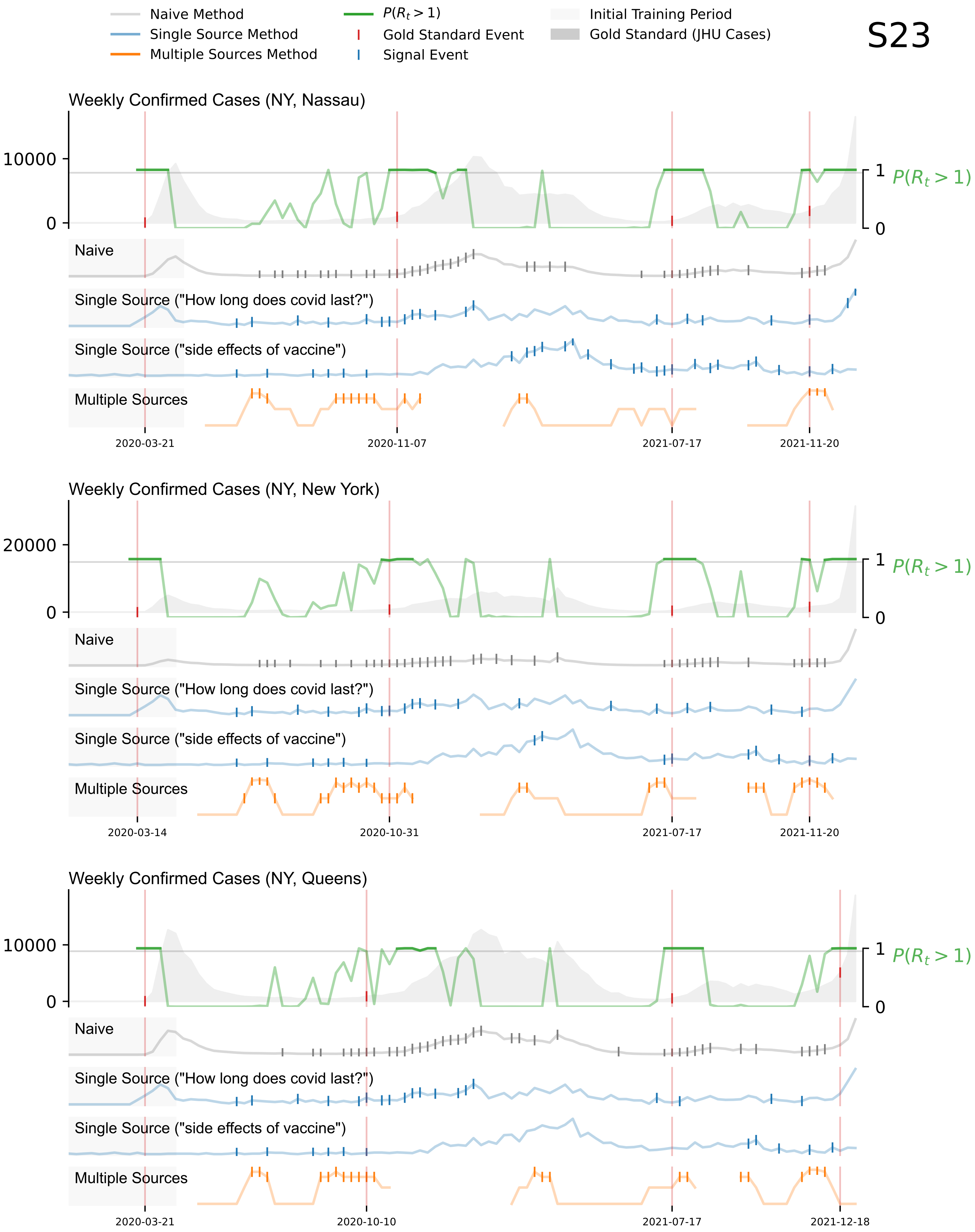
Naive Method
Single Source Method
Multiple Sources Method
$P(R_t > 1)$
Gold Standard Event
Signal Event
Initial Training Period
Gold Standard (JHU Cases)
S23
Weekly Confirmed Cases (NY, Nassau)
10000
0
1
0
Naive
Single Source ("How long does covid last?")
Single Source ("side effects of vaccine")
Multiple Sources
2020-03-21
2020-11-07
2021-07-17
2021-11-20
Weekly Confirmed Cases (NY, New York)
20000
0
2020-03-14
2020-10-31
2021-07-17
2021-11-20
Weekly Confirmed Cases (NY, Queens)
10000
0
2020-03-21
2020-10-10
2021-07-17
2021-12-18

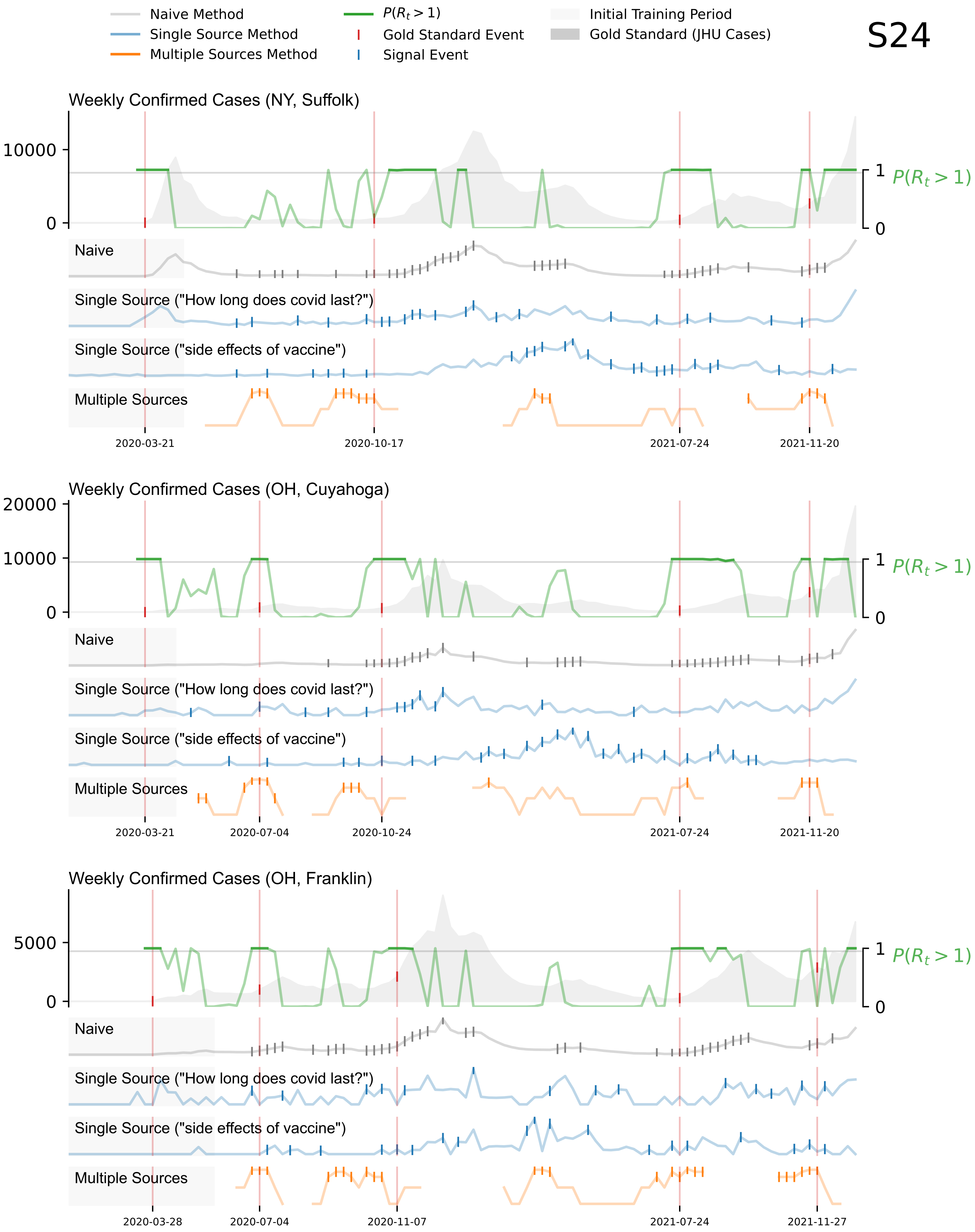

Naive Method
Single Source Method
Multiple Sources Method
$P(R_t > 1)$
Gold Standard Event
Signal Event
Initial Training Period
Gold Standard (JHU Cases)
S24
Weekly Confirmed Cases (NY, Suffolk)
10000
0
1
0
Naive
Single Source ("How long does covid last?")
Single Source ("side effects of vaccine")
Multiple Sources
2020-03-21
2020-10-17
2021-07-24
2021-11-20
Weekly Confirmed Cases (OH, Cuyahoga)
20000
10000
0
Naive
Single Source ("How long does covid last?")
Single Source ("side effects of vaccine")
Multiple Sources
2020-03-21
2020-07-04
2020-10-24
2021-07-24
2021-11-20
Weekly Confirmed Cases (OH, Franklin)
5000
0
Naive
Single Source ("How long does covid last?")
Single Source ("side effects of vaccine")
Multiple Sources
2020-03-28
2020-07-04
2020-11-07
2021-07-24
2021-11-27

S25

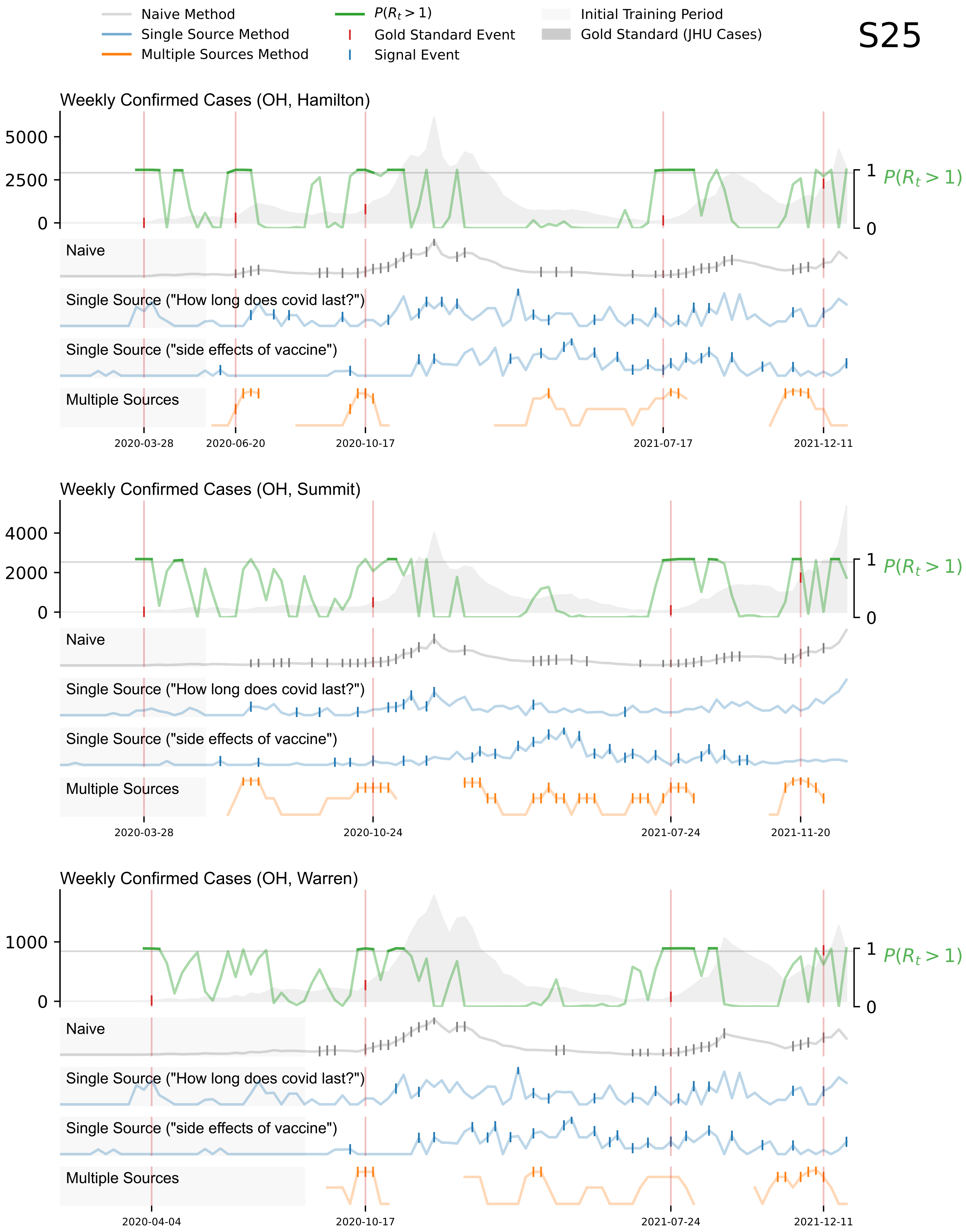

Naive Method
Single Source Method
Multiple Sources Method
$P(R_t > 1)$
Gold Standard Event
Signal Event
Initial Training Period
Gold Standard (JHU Cases)
Weekly Confirmed Cases (OH, Hamilton)
5000
2500
0
1
0
$P(R_t > 1)$
Naive
Single Source ("How long does covid last?")
Single Source ("side effects of vaccine")
Multiple Sources
2020-03-28
2020-06-20
2020-10-17
2021-07-17
2021-12-11
Weekly Confirmed Cases (OH, Summit)
4000
2000
0
1
0
$P(R_t > 1)$
Naive
Single Source ("How long does covid last?")
Single Source ("side effects of vaccine")
Multiple Sources
2020-03-28
2020-10-24
2021-07-24
2021-11-20
Weekly Confirmed Cases (OH, Warren)
1000
0
1
0
$P(R_t > 1)$
Naive
Single Source ("How long does covid last?")
Single Source ("side effects of vaccine")
Multiple Sources
2020-04-04
2020-10-17
2021-07-24
2021-12-11

S26

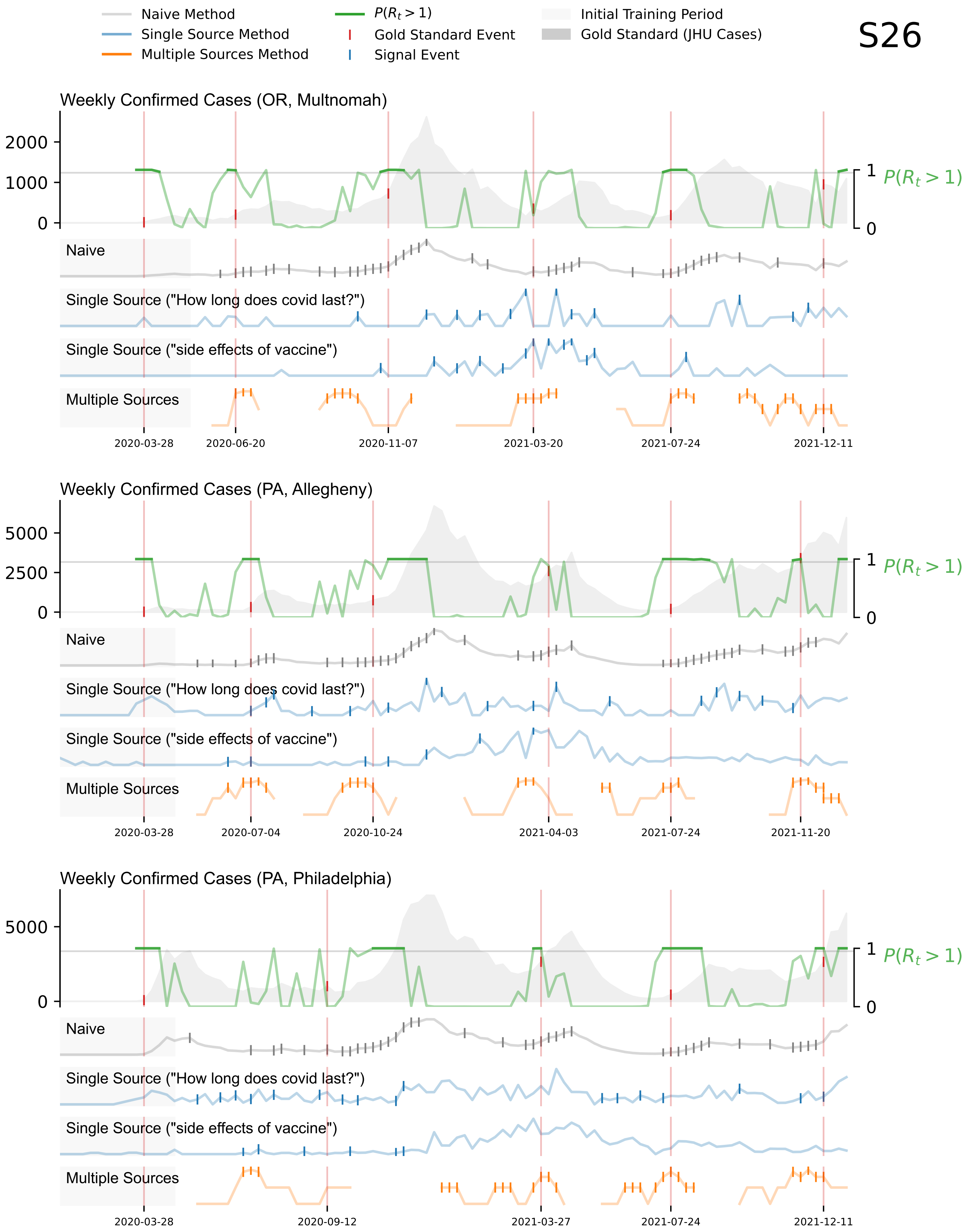

Naive Method
Single Source Method
Multiple Sources Method
P(R_t > 1)
Gold Standard Event
Signal Event
Initial Training Period
Gold Standard (JHU Cases)
Weekly Confirmed Cases (OR, Multnomah)
2000
1000
0
1
0
P(R_t > 1)
Naive
Single Source ("How long does covid last?")
Single Source ("side effects of vaccine")
Multiple Sources
2020-03-28
2020-06-20
2020-11-07
2021-03-20
2021-07-24
2021-12-11
Weekly Confirmed Cases (PA, Allegheny)
5000
2500
0
1
0
P(R_t > 1)
Naive
Single Source ("How long does covid last?")
Single Source ("side effects of vaccine")
Multiple Sources
2020-03-28
2020-07-04
2020-10-24
2021-04-03
2021-07-24
2021-11-20
Weekly Confirmed Cases (PA, Philadelphia)
5000
0
1
0
P(R_t > 1)
Naive
Single Source ("How long does covid last?")
Single Source ("side effects of vaccine")
Multiple Sources
2020-03-28
2020-09-12
2021-03-27
2021-07-24
2021-12-11

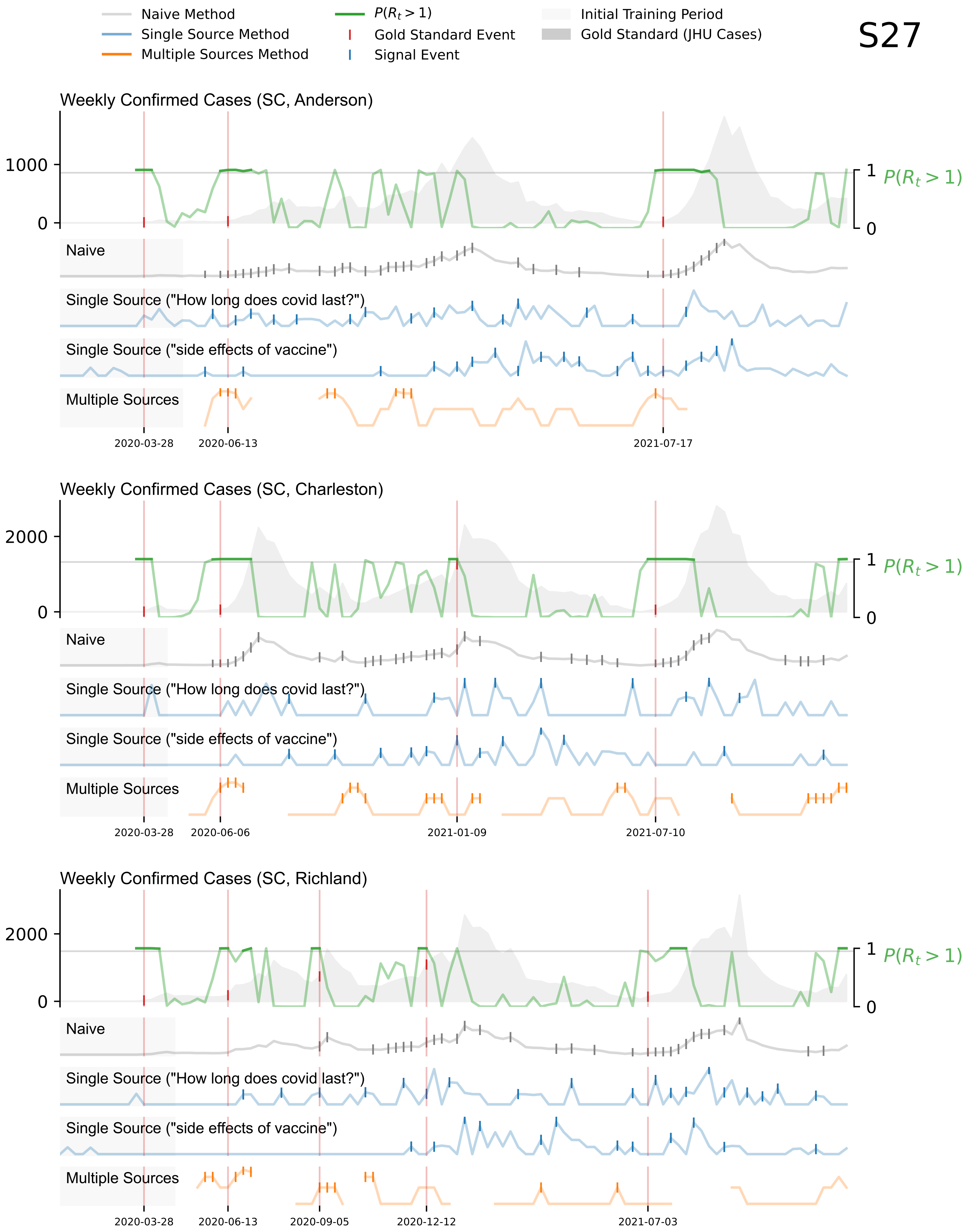

Naive Method
Single Source Method
Multiple Sources Method
$P(R_t > 1)$
Gold Standard Event
Signal Event
Initial Training Period
Gold Standard (JHU Cases)
S27
Weekly Confirmed Cases (SC, Anderson)
1000
0
1
0
$P(R_t > 1)$
Naive
Single Source ("How long does covid last?")
Single Source ("side effects of vaccine")
Multiple Sources
2020-03-28
2020-06-13
2021-07-17
Weekly Confirmed Cases (SC, Charleston)
2000
0
Naive
Single Source ("How long does covid last?")
Single Source ("side effects of vaccine")
Multiple Sources
2020-03-28
2020-06-06
2021-01-09
2021-07-10
Weekly Confirmed Cases (SC, Richland)
2000
0
Naive
Single Source ("How long does covid last?")
Single Source ("side effects of vaccine")
Multiple Sources
2020-03-28
2020-06-13
2020-09-05
2020-12-12
2021-07-03

S28

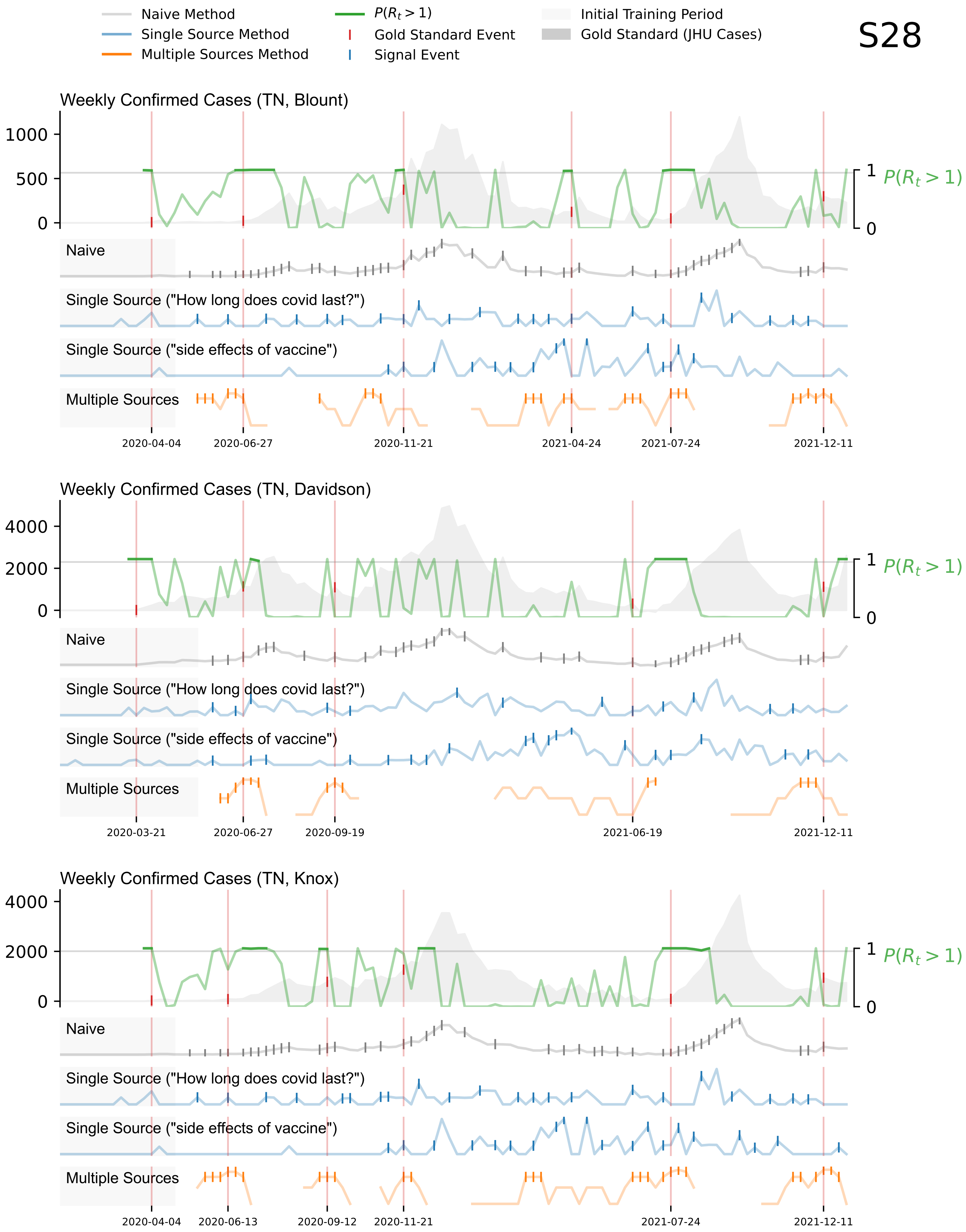

Naive Method
Single Source Method
Multiple Sources Method
$P(R_t > 1)$
Gold Standard Event
Signal Event
Initial Training Period
Gold Standard (JHU Cases)
Weekly Confirmed Cases (TN, Blount)
1000
500
0
1
0
$P(R_t > 1)$
Naive
Single Source ("How long does covid last?")
Single Source ("side effects of vaccine")
Multiple Sources
2020-04-04
2020-06-27
2020-11-21
2021-04-24
2021-07-24
2021-12-11
Weekly Confirmed Cases (TN, Davidson)
4000
2000
0
1
0
$P(R_t > 1)$
Naive
Single Source ("How long does covid last?")
Single Source ("side effects of vaccine")
Multiple Sources
2020-03-21
2020-06-27
2020-09-19
2021-06-19
2021-12-11
Weekly Confirmed Cases (TN, Knox)
4000
2000
0
1
0
$P(R_t > 1)$
Naive
Single Source ("How long does covid last?")
Single Source ("side effects of vaccine")
Multiple Sources
2020-04-04
2020-06-13
2020-09-12
2020-11-21
2021-07-24
2021-12-11

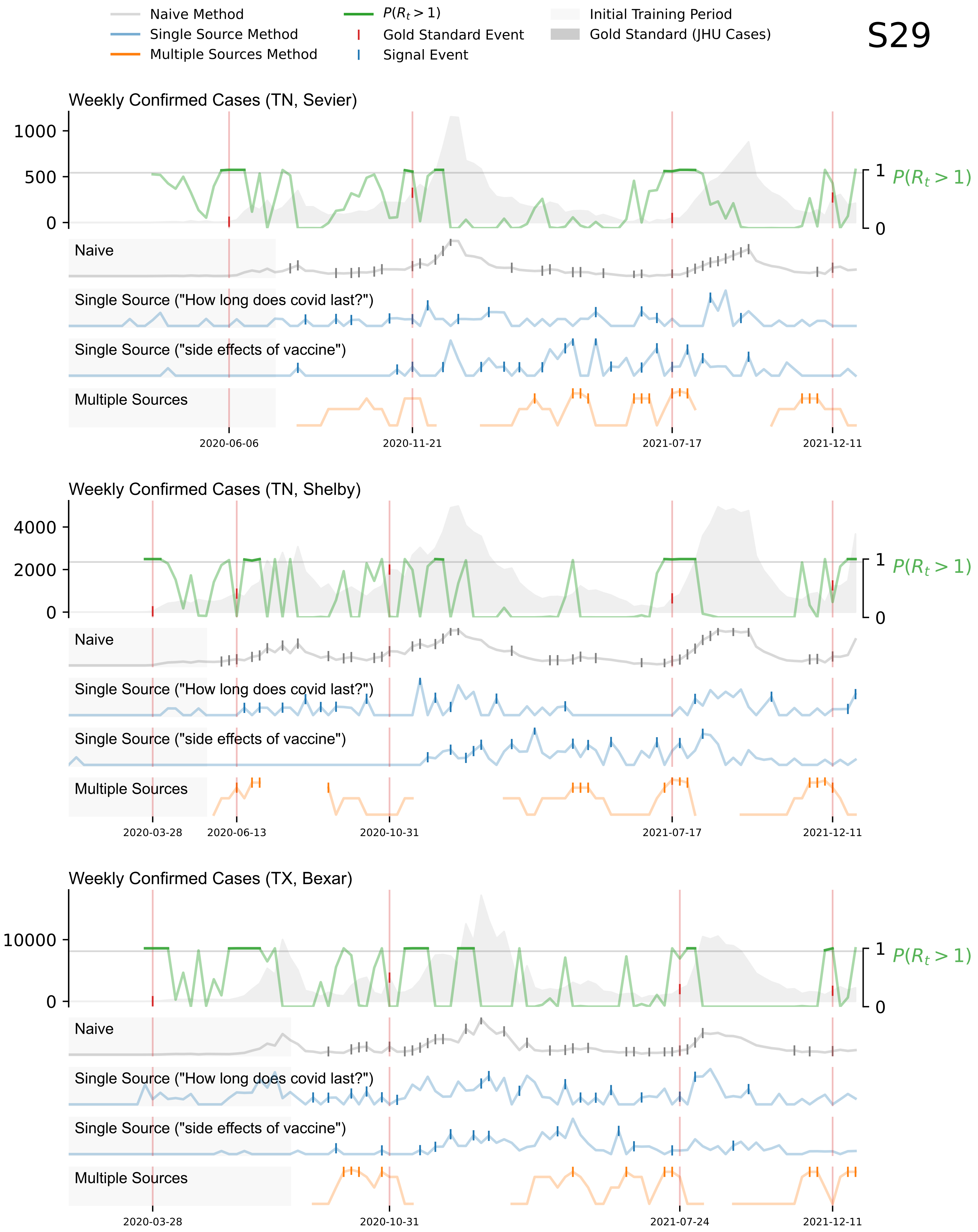

S29
Naive Method
Single Source Method
Multiple Sources Method
$P(R_t > 1)$
Gold Standard Event
Signal Event
Initial Training Period
Gold Standard (JHU Cases)
Weekly Confirmed Cases (TN, Sevier)
1000
500
0
1
0
$P(R_t > 1)$
Naive
Single Source ("How long does covid last?")
Single Source ("side effects of vaccine")
Multiple Sources
2020-06-06
2020-11-21
2021-07-17
2021-12-11
Weekly Confirmed Cases (TN, Shelby)
4000
2000
0
1
0
$P(R_t > 1)$
Naive
Single Source ("How long does covid last?")
Single Source ("side effects of vaccine")
Multiple Sources
2020-03-28
2020-06-13
2020-10-31
2021-07-17
2021-12-11
Weekly Confirmed Cases (TX, Bexar)
10000
0
1
0
$P(R_t > 1)$
Naive
Single Source ("How long does covid last?")
Single Source ("side effects of vaccine")
Multiple Sources
2020-03-28
2020-10-31
2021-07-24
2021-12-11

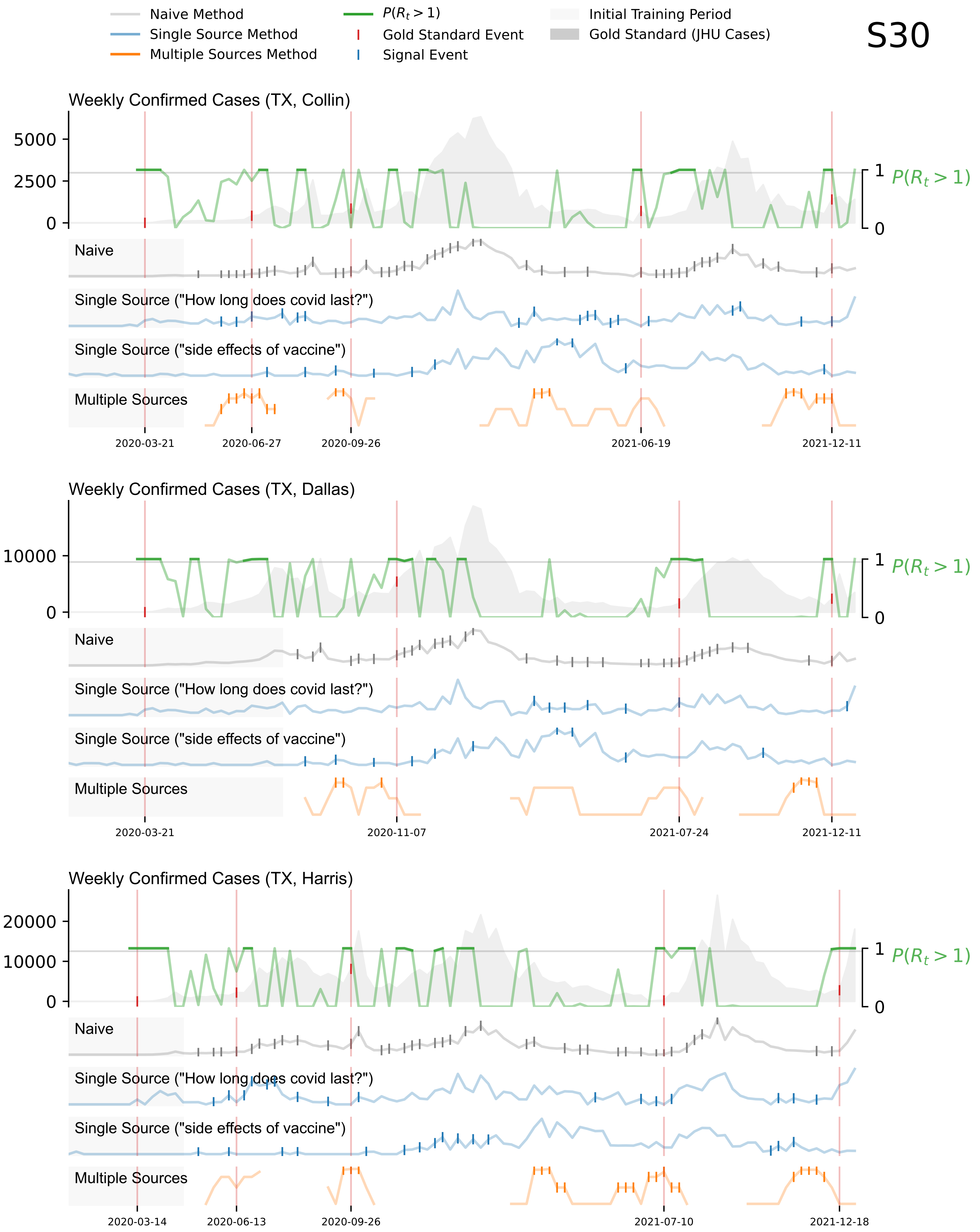
Naive Method
Single Source Method
Multiple Sources Method
$P(R_t > 1)$
Gold Standard Event
Signal Event
Initial Training Period
Gold Standard (JHU Cases)
S30
Weekly Confirmed Cases (TX, Collin)
5000
2500
0
1
0
$P(R_t > 1)$
Naive
Single Source ("How long does covid last?")
Single Source ("side effects of vaccine")
Multiple Sources
2020-03-21
2020-06-27
2020-09-26
2021-06-19
2021-12-11
Weekly Confirmed Cases (TX, Dallas)
10000
0
1
0
$P(R_t > 1)$
Naive
Single Source ("How long does covid last?")
Single Source ("side effects of vaccine")
Multiple Sources
2020-03-21
2020-11-07
2021-07-24
2021-12-11
Weekly Confirmed Cases (TX, Harris)
20000
10000
0
1
0
$P(R_t > 1)$
Naive
Single Source ("How long does covid last?")
Single Source ("side effects of vaccine")
Multiple Sources
2020-03-14
2020-06-13
2020-09-26
2021-07-10
2021-12-18

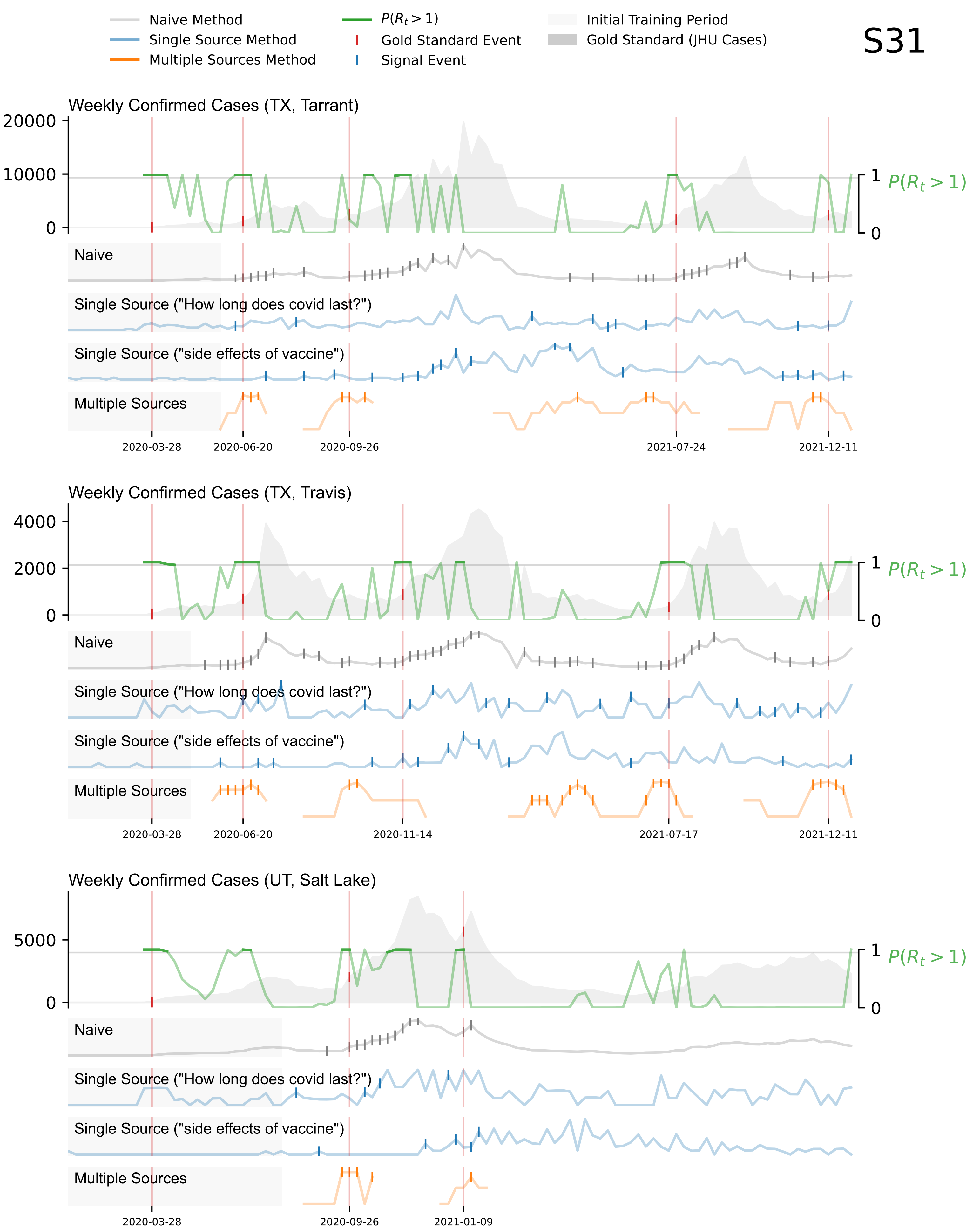

Naive Method
Single Source Method
Multiple Sources Method
$P(R_t > 1)$
Gold Standard Event
Signal Event
Initial Training Period
Gold Standard (JHU Cases)
S31
Weekly Confirmed Cases (TX, Tarrant)
20000
10000
0
1
0
Naive
Single Source ("How long does covid last?")
Single Source ("side effects of vaccine")
Multiple Sources
2020-03-28
2020-06-20
2020-09-26
2021-07-24
2021-12-11
Weekly Confirmed Cases (TX, Travis)
4000
2000
0
2020-03-28
2020-06-20
2020-11-14
2021-07-17
2021-12-11
Weekly Confirmed Cases (UT, Salt Lake)
5000
0
2020-03-28
2020-09-26
2021-01-09

S32

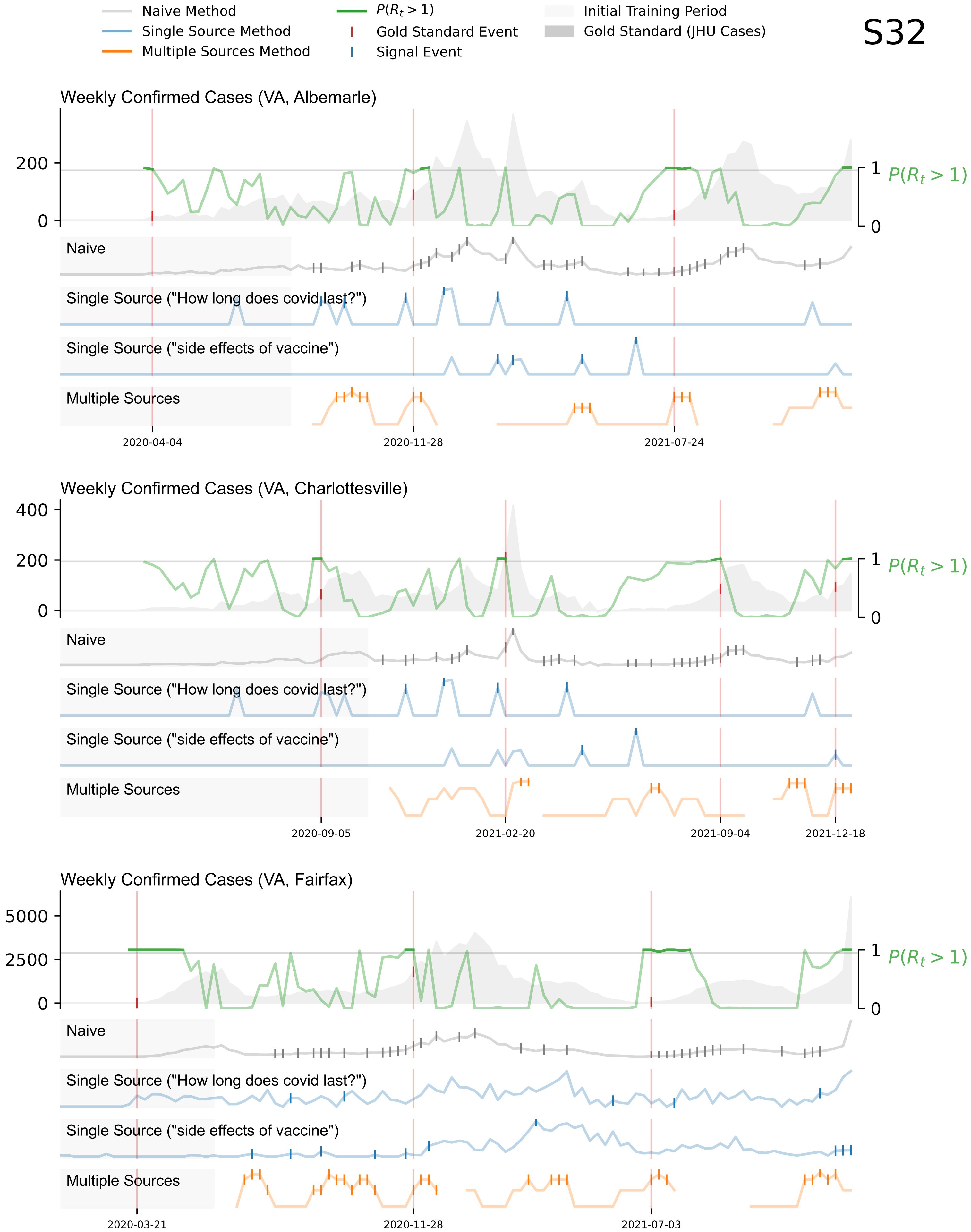
Naive Method
Single Source Method
Multiple Sources Method
$P(R_t > 1)$
Gold Standard Event
Signal Event
Initial Training Period
Gold Standard (JHU Cases)
Weekly Confirmed Cases (VA, Albemarle)
200
0
1
0
$P(R_t > 1)$
Naive
Single Source ("How long does covid last?")
Single Source ("side effects of vaccine")
Multiple Sources
2020-04-04
2020-11-28
2021-07-24
Weekly Confirmed Cases (VA, Charlottesville)
400
200
0
1
0
$P(R_t > 1)$
Naive
Single Source ("How long does covid last?")
Single Source ("side effects of vaccine")
Multiple Sources
2020-09-05
2021-02-20
2021-09-04
2021-12-18
Weekly Confirmed Cases (VA, Fairfax)
5000
2500
0
1
0
$P(R_t > 1)$
Naive
Single Source ("How long does covid last?")
Single Source ("side effects of vaccine")
Multiple Sources
2020-03-21
2020-11-28
2021-07-03

Figures S1-S49.
The probability of resurgence P(Rt > 1) (in green) and the different events generated by the Naive (gray), Single Source (blue) and Multiple Source methods (orange) at both county and state level.

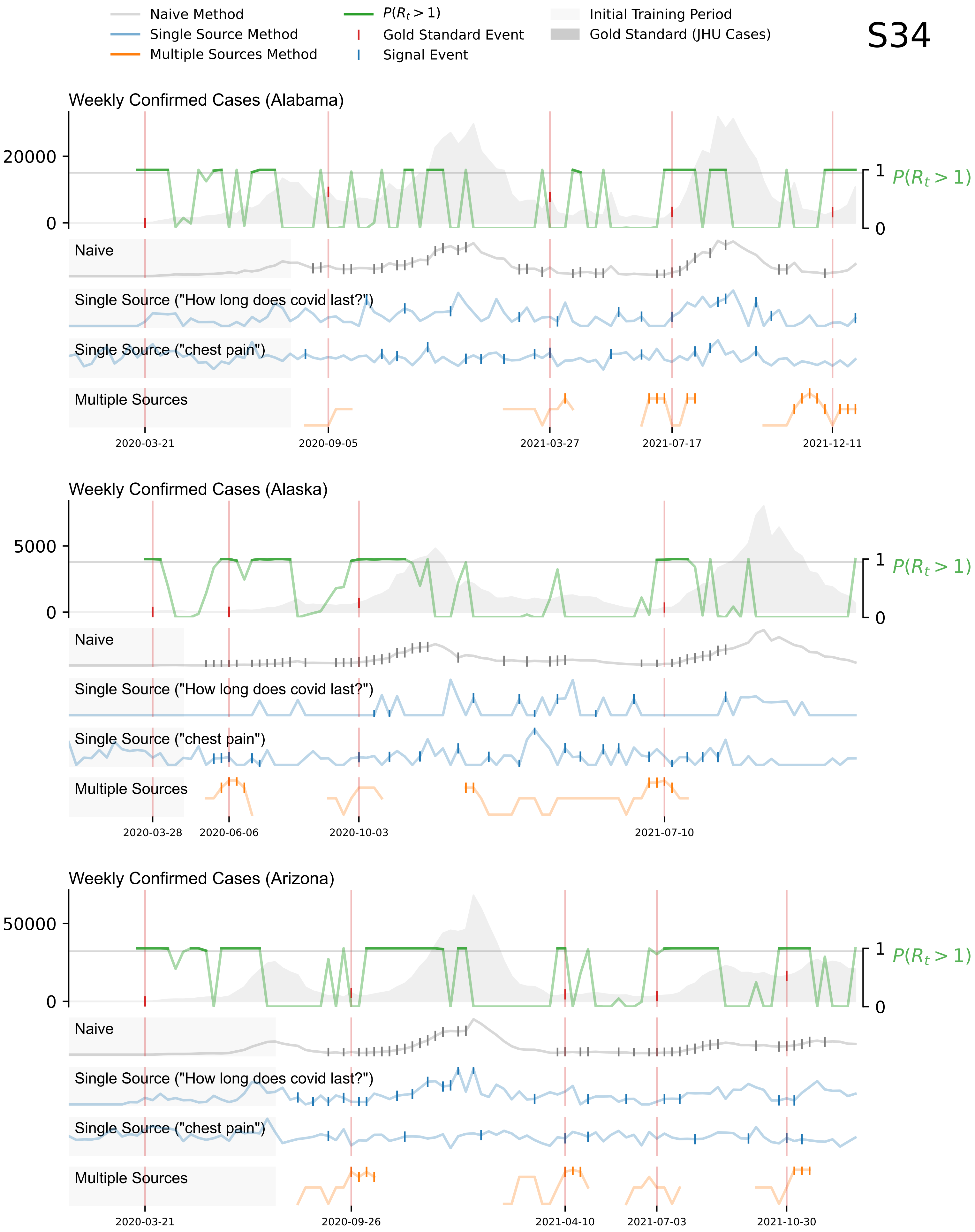

S34
Naive Method
Single Source Method
Multiple Sources Method
$P(R_t > 1)$
Gold Standard Event
Signal Event
Initial Training Period
Gold Standard (JHU Cases)
Weekly Confirmed Cases (Alabama)
20000
0
1
0
$P(R_t > 1)$
Naive
Single Source ("How long does covid last?")
Single Source ("chest pain")
Multiple Sources
2020-03-21
2020-09-05
2021-03-27
2021-07-17
2021-12-11
Weekly Confirmed Cases (Alaska)
5000
0
1
0
$P(R_t > 1)$
Naive
Single Source ("How long does covid last?")
Single Source ("chest pain")
Multiple Sources
2020-03-28
2020-06-06
2020-10-03
2021-07-10
Weekly Confirmed Cases (Arizona)
50000
0
1
0
$P(R_t > 1)$
Naive
Single Source ("How long does covid last?")
Single Source ("chest pain")
Multiple Sources
2020-03-21
2020-09-26
2021-04-10
2021-07-03
2021-10-30

S35

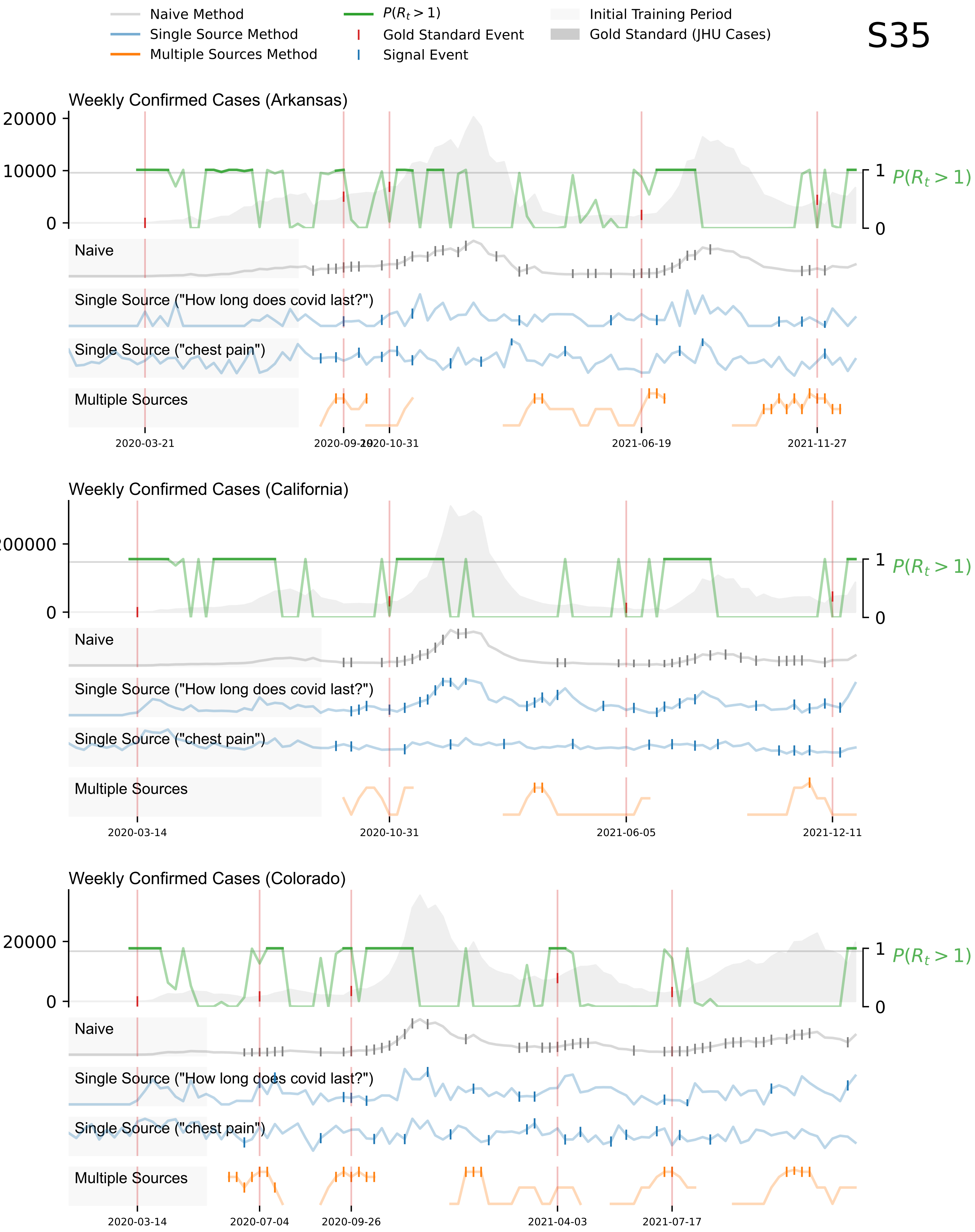
Naive Method
Single Source Method
Multiple Sources Method
$P(R_t > 1)$
Gold Standard Event
Signal Event
Initial Training Period
Gold Standard (JHU Cases)
Weekly Confirmed Cases (Arkansas)
20000
10000
0
1
0
$P(R_t > 1)$
Naive
Single Source ("How long does covid last?")
Single Source ("chest pain")
Multiple Sources
2020-03-21
2020-09-192020-10-31
2021-06-19
2021-11-27
Weekly Confirmed Cases (California)
200000
0
1
0
$P(R_t > 1)$
Naive
Single Source ("How long does covid last?")
Single Source ("chest pain")
Multiple Sources
2020-03-14
2020-10-31
2021-06-05
2021-12-11
Weekly Confirmed Cases (Colorado)
20000
0
1
0
$P(R_t > 1)$
Naive
Single Source ("How long does covid last?")
Single Source ("chest pain")
Multiple Sources
2020-03-14
2020-07-04
2020-09-26
2021-04-03
2021-07-17

S36

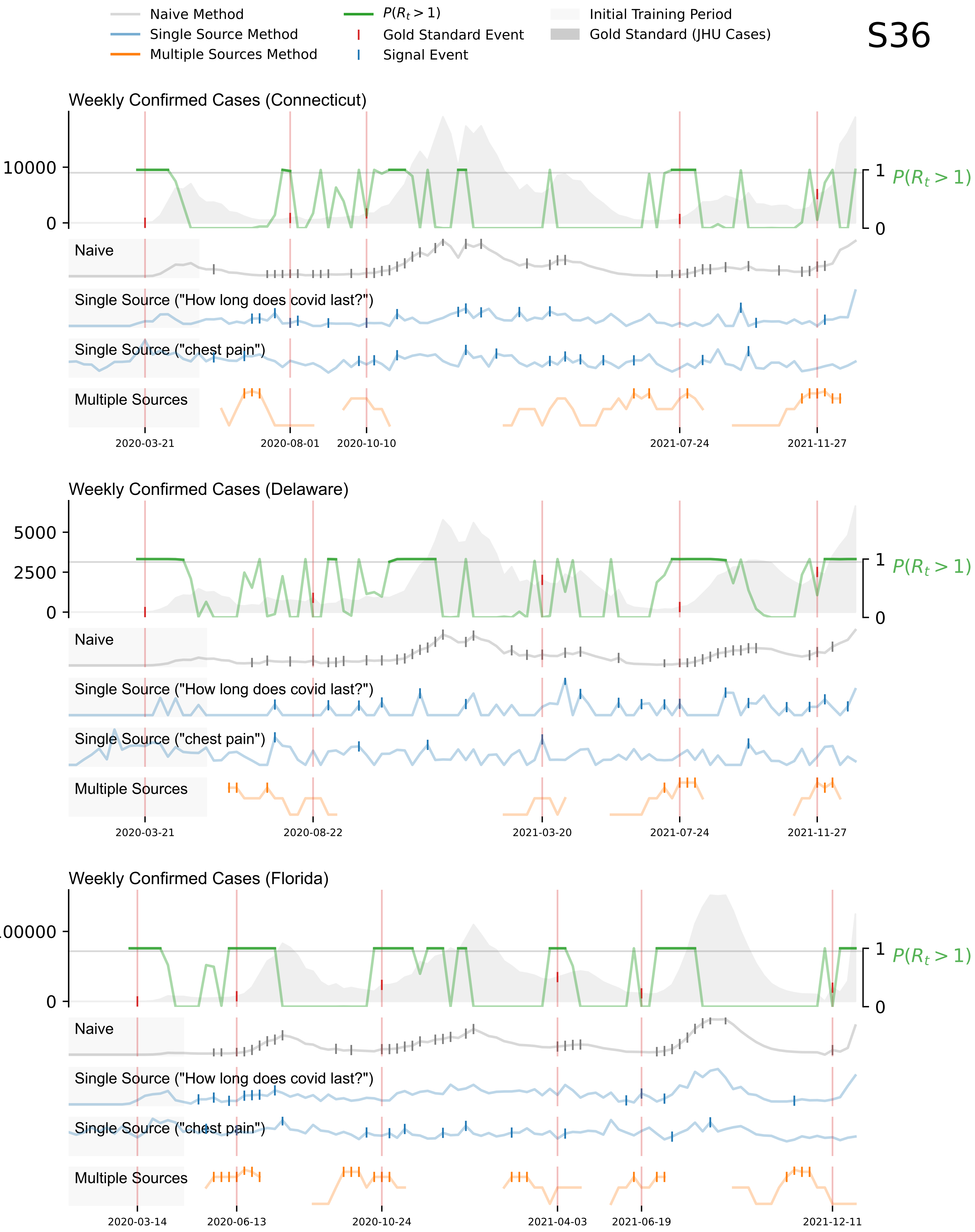
Naive Method
Single Source Method
Multiple Sources Method
$P(R_t > 1)$
Gold Standard Event
Signal Event
Initial Training Period
Gold Standard (JHU Cases)
Weekly Confirmed Cases (Connecticut)
10000
0
1
0
$P(R_t > 1)$
Naive
Single Source ("How long does covid last?")
Single Source ("chest pain")
Multiple Sources
2020-03-21
2020-08-01
2020-10-10
2021-07-24
2021-11-27
Weekly Confirmed Cases (Delaware)
5000
2500
0
1
0
$P(R_t > 1)$
Naive
Single Source ("How long does covid last?")
Single Source ("chest pain")
Multiple Sources
2020-03-21
2020-08-22
2021-03-20
2021-07-24
2021-11-27
Weekly Confirmed Cases (Florida)
100000
0
1
0
$P(R_t > 1)$
Naive
Single Source ("How long does covid last?")
Single Source ("chest pain")
Multiple Sources
2020-03-14
2020-06-13
2020-10-24
2021-04-03
2021-06-19
2021-12-11

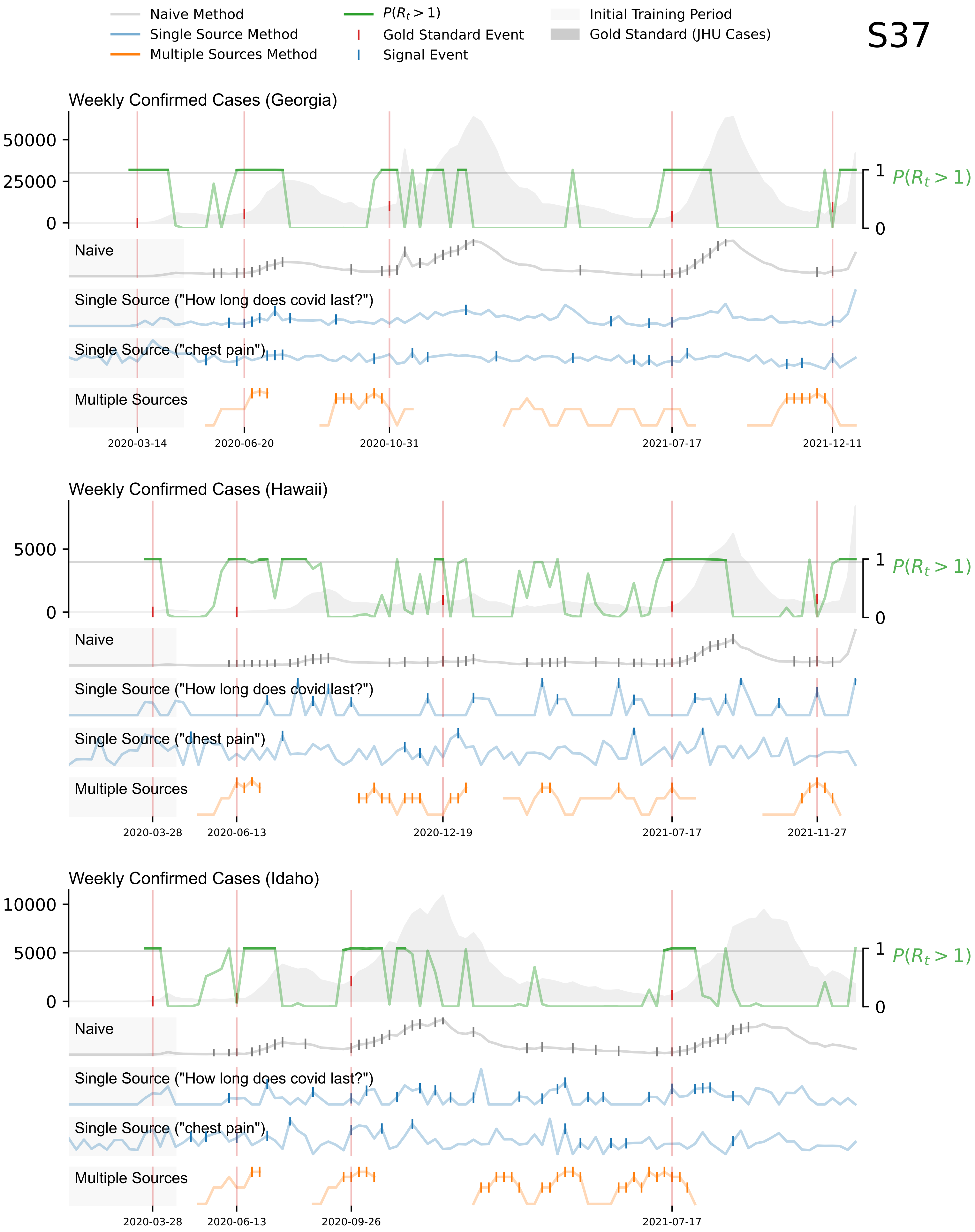

S37
Naive Method
Single Source Method
Multiple Sources Method
$P(R_t > 1)$
Gold Standard Event
Signal Event
Initial Training Period
Gold Standard (JHU Cases)
Weekly Confirmed Cases (Georgia)
50000
25000
0
1
0
Naive
Single Source ("How long does covid last?")
Single Source ("chest pain")
Multiple Sources
2020-03-14
2020-06-20
2020-10-31
2021-07-17
2021-12-11
Weekly Confirmed Cases (Hawaii)
5000
0
2020-03-28
2020-06-13
2020-12-19
2021-07-17
2021-11-27
Weekly Confirmed Cases (Idaho)
10000
5000
0
2020-03-28
2020-06-13
2020-09-26
2021-07-17

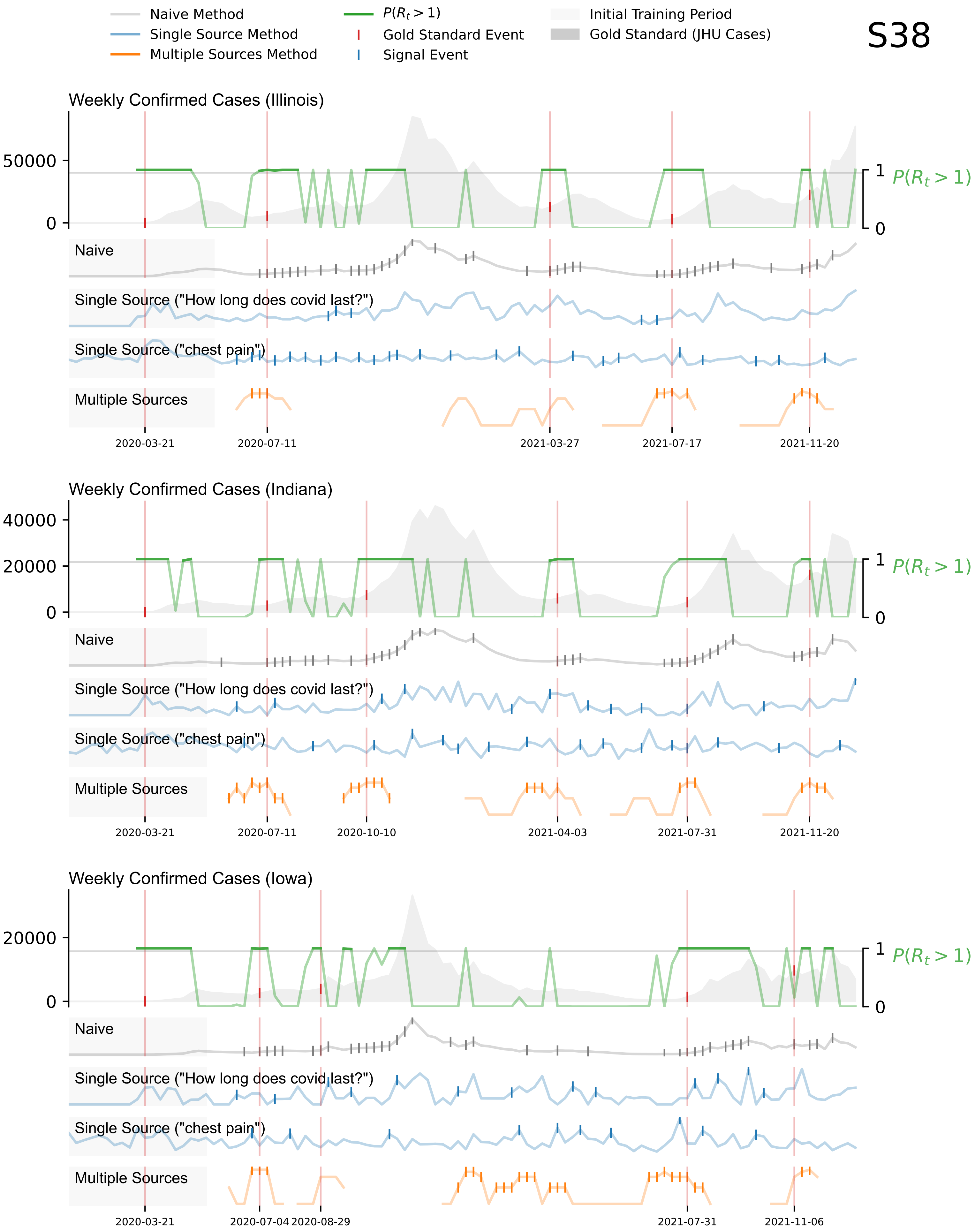

Naive Method
Single Source Method
Multiple Sources Method
$P(R_t > 1)$
Gold Standard Event
Signal Event
Initial Training Period
Gold Standard (JHU Cases)
S38
Weekly Confirmed Cases (Illinois)
50000
0
1
0
Naive
Single Source ("How long does covid last?")
Single Source ("chest pain")
Multiple Sources
2020-03-21
2020-07-11
2021-03-27
2021-07-17
2021-11-20
Weekly Confirmed Cases (Indiana)
40000
20000
0
2020-03-21
2020-07-11
2020-10-10
2021-04-03
2021-07-31
2021-11-20
Weekly Confirmed Cases (Iowa)
20000
0
2020-03-21
2020-07-04
2020-08-29
2021-07-31
2021-11-06

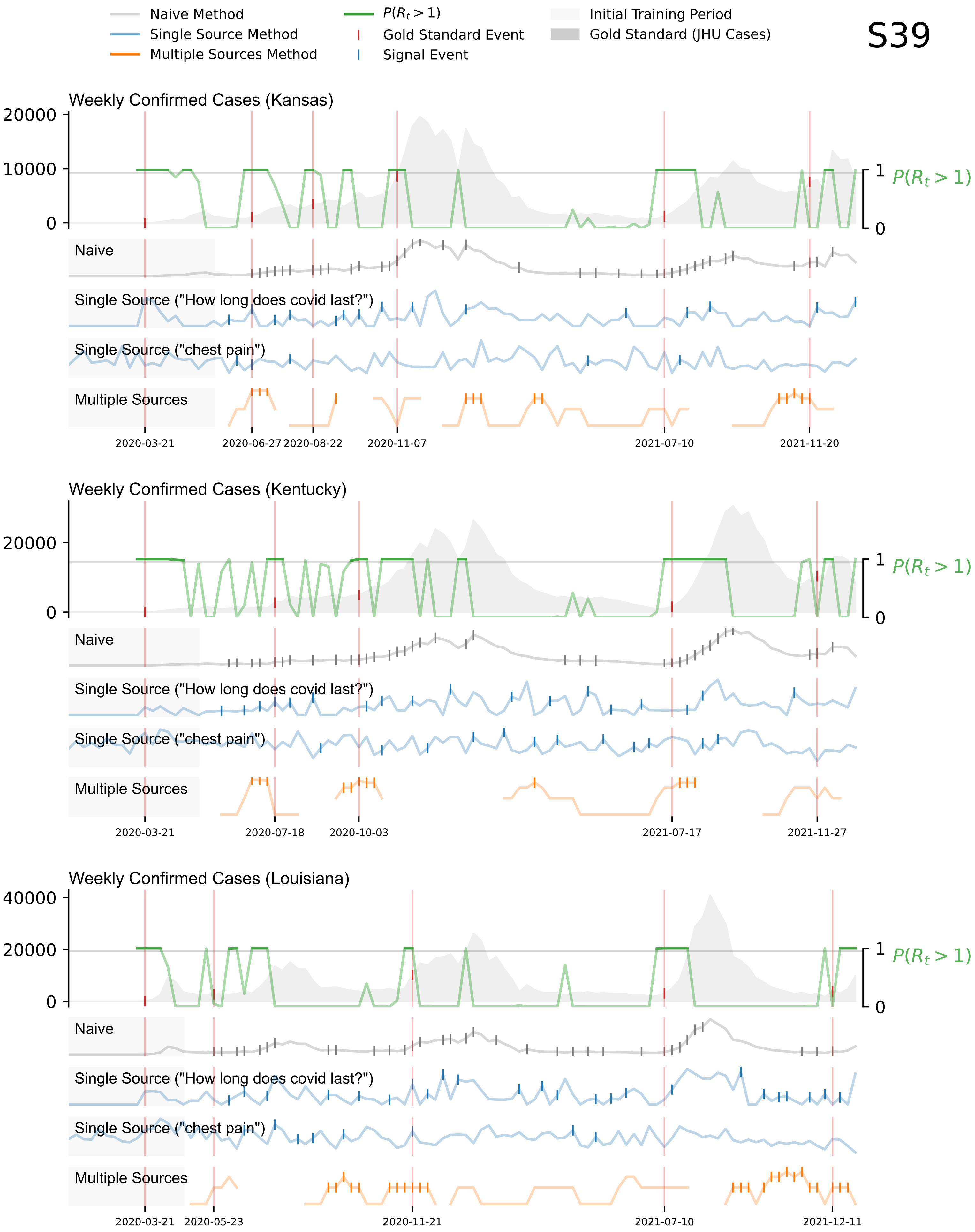

Naive Method
Single Source Method
Multiple Sources Method
$P(R_t > 1)$
Gold Standard Event
Signal Event
Initial Training Period
Gold Standard (JHU Cases)
S39
Weekly Confirmed Cases (Kansas)
20000
10000
0
1
0
$P(R_t > 1)$
Naive
Single Source ("How long does covid last?")
Single Source ("chest pain")
Multiple Sources
2020-03-21
2020-06-27
2020-08-22
2020-11-07
2021-07-10
2021-11-20
Weekly Confirmed Cases (Kentucky)
20000
0
1
0
$P(R_t > 1)$
Naive
Single Source ("How long does covid last?")
Single Source ("chest pain")
Multiple Sources
2020-03-21
2020-07-18
2020-10-03
2021-07-17
2021-11-27
Weekly Confirmed Cases (Louisiana)
40000
20000
0
1
0
$P(R_t > 1)$
Naive
Single Source ("How long does covid last?")
Single Source ("chest pain")
Multiple Sources
2020-03-21
2020-05-23
2020-11-21
2021-07-10
2021-12-11

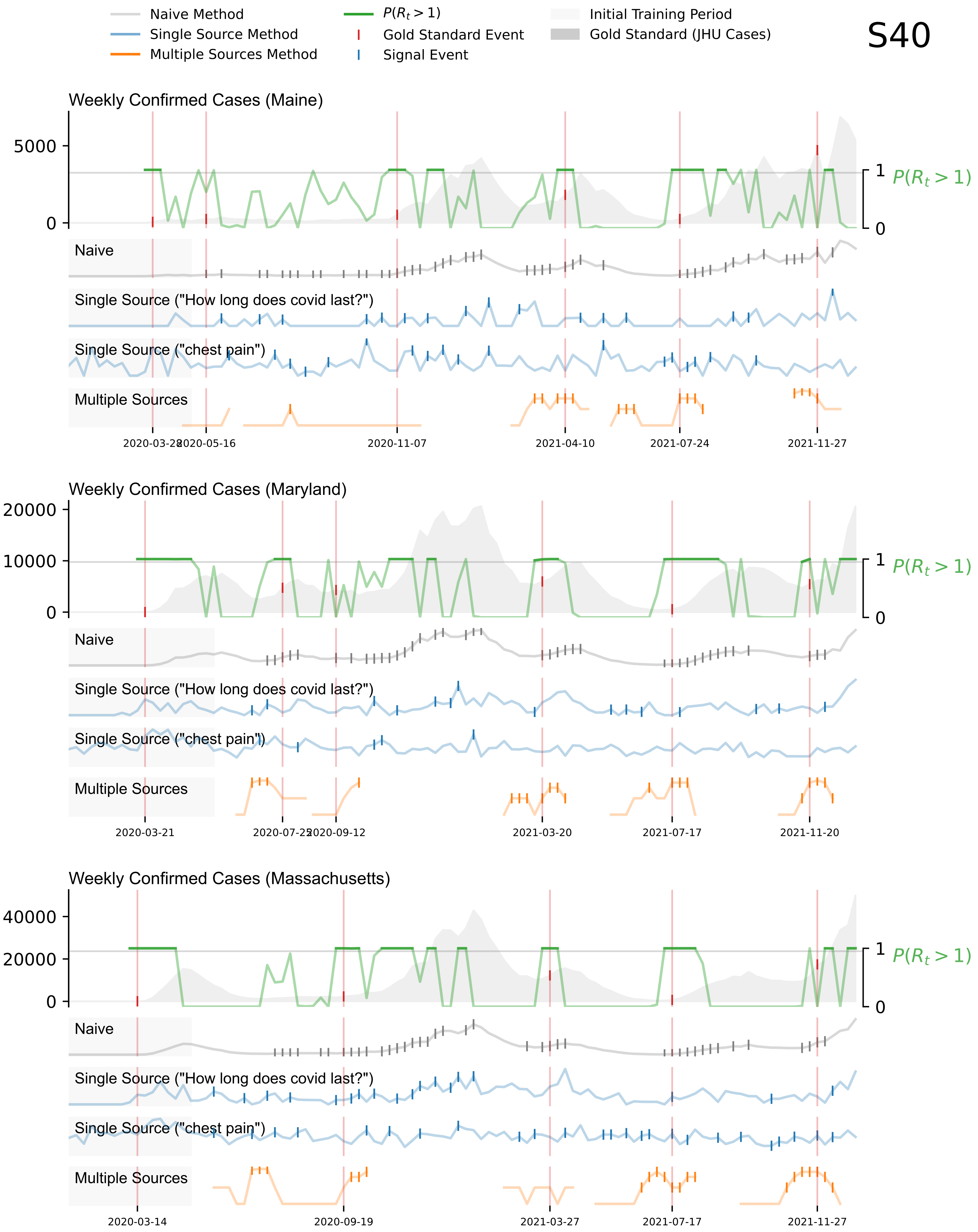

Naive Method
Single Source Method
Multiple Sources Method
$P(R_t > 1)$
Gold Standard Event
Signal Event
Initial Training Period
Gold Standard (JHU Cases)
S40
Weekly Confirmed Cases (Maine)
5000
0
1
0
$P(R_t > 1)$
Naive
Single Source ("How long does covid last?")
Single Source ("chest pain")
Multiple Sources
2020-03-28
2020-05-16
2020-11-07
2021-04-10
2021-07-24
2021-11-27
Weekly Confirmed Cases (Maryland)
20000
10000
0
Naive
Single Source ("How long does covid last?")
Single Source ("chest pain")
Multiple Sources
2020-03-21
2020-07-25
2020-09-12
2021-03-20
2021-07-17
2021-11-20
Weekly Confirmed Cases (Massachusetts)
40000
20000
0
Naive
Single Source ("How long does covid last?")
Single Source ("chest pain")
Multiple Sources
2020-03-14
2020-09-19
2021-03-27
2021-07-17
2021-11-27

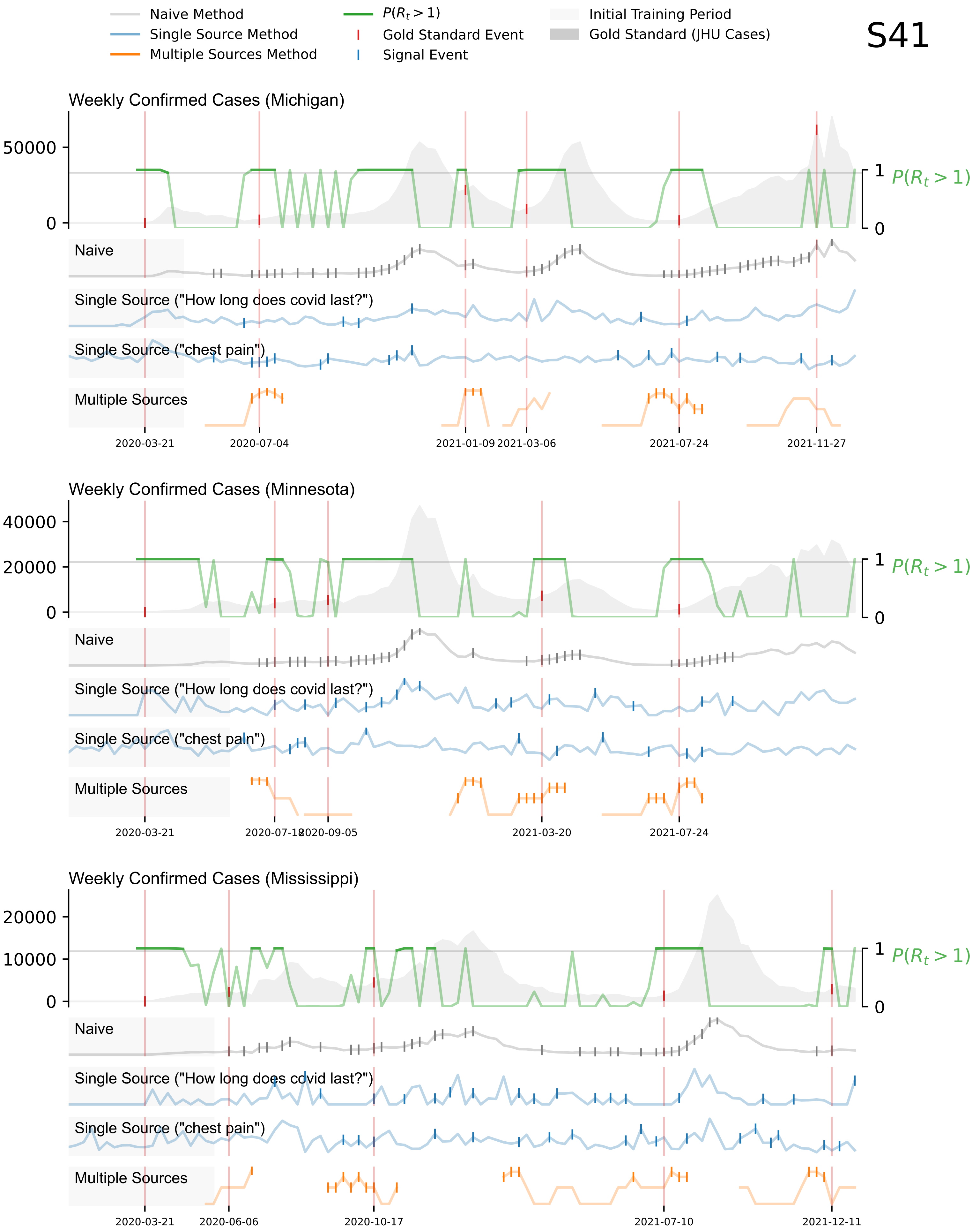

Naive Method
Single Source Method
Multiple Sources Method
$P(R_t > 1)$
Gold Standard Event
Signal Event
Initial Training Period
Gold Standard (JHU Cases)
S41
Weekly Confirmed Cases (Michigan)
50000
0
1
0
$P(R_t > 1)$
Naive
Single Source ("How long does covid last?")
Single Source ("chest pain")
Multiple Sources
2020-03-21
2020-07-04
2021-01-09
2021-03-06
2021-07-24
2021-11-27
Weekly Confirmed Cases (Minnesota)
40000
20000
0
1
0
$P(R_t > 1)$
Naive
Single Source ("How long does covid last?")
Single Source ("chest pain")
Multiple Sources
2020-03-21
2020-07-18
2020-09-05
2021-03-20
2021-07-24
Weekly Confirmed Cases (Mississippi)
20000
10000
0
1
0
$P(R_t > 1)$
Naive
Single Source ("How long does covid last?")
Single Source ("chest pain")
Multiple Sources
2020-03-21
2020-06-06
2020-10-17
2021-07-10
2021-12-11

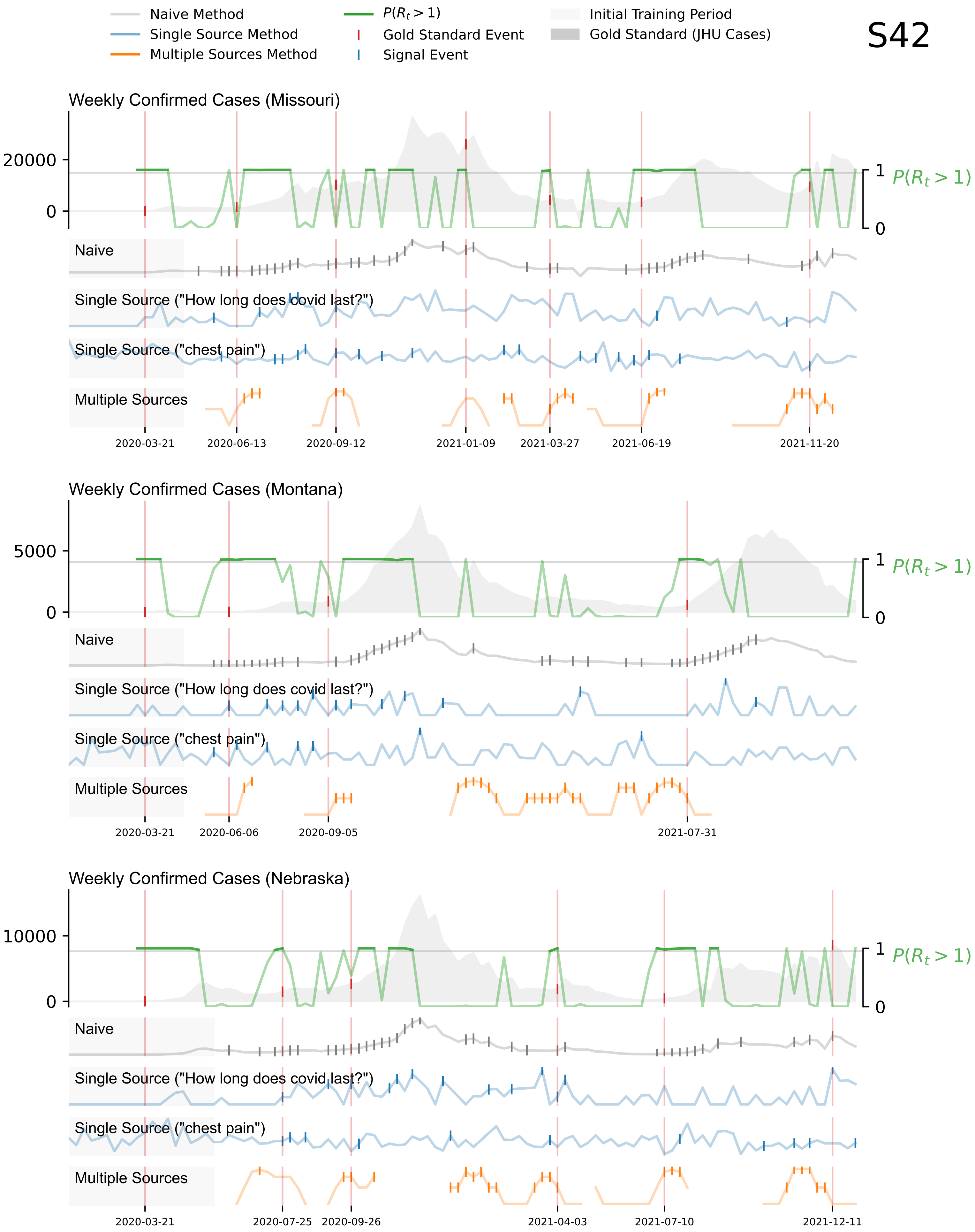

Naive Method
Single Source Method
Multiple Sources Method
$P(R_t > 1)$
Gold Standard Event
Signal Event
Initial Training Period
Gold Standard (JHU Cases)
S42
Weekly Confirmed Cases (Missouri)
20000
0
1
0
$P(R_t > 1)$
Naive
Single Source ("How long does covid last?")
Single Source ("chest pain")
Multiple Sources
2020-03-21
2020-06-13
2020-09-12
2021-01-09
2021-03-27
2021-06-19
2021-11-20
Weekly Confirmed Cases (Montana)
5000
0
1
0
$P(R_t > 1)$
Naive
Single Source ("How long does covid last?")
Single Source ("chest pain")
Multiple Sources
2020-03-21
2020-06-06
2020-09-05
2021-07-31
Weekly Confirmed Cases (Nebraska)
10000
0
1
0
$P(R_t > 1)$
Naive
Single Source ("How long does covid last?")
Single Source ("chest pain")
Multiple Sources
2020-03-21
2020-07-25
2020-09-26
2021-04-03
2021-07-10
2021-12-11

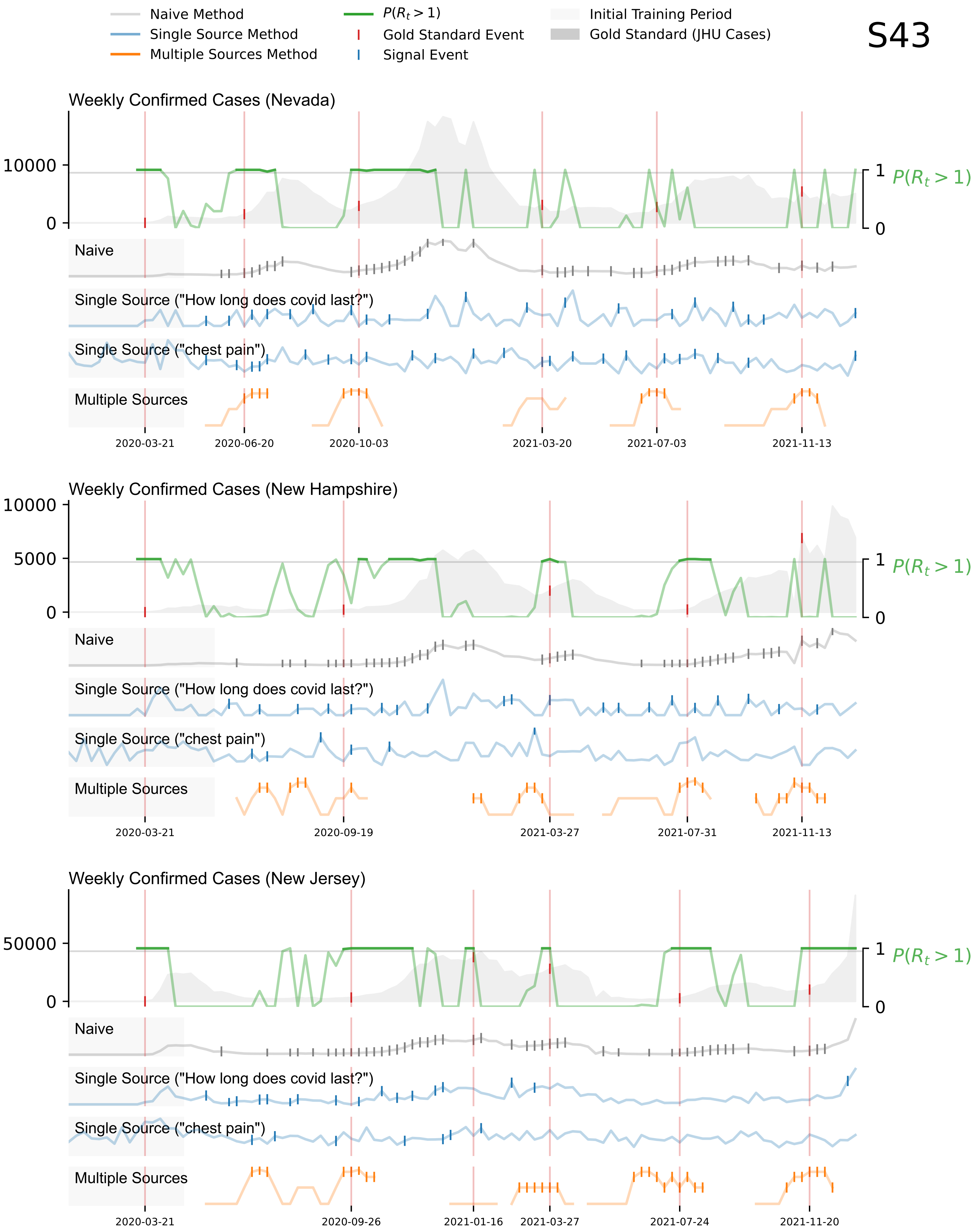

Naive Method
Single Source Method
Multiple Sources Method
$P(R_t > 1)$
Gold Standard Event
Signal Event
Initial Training Period
Gold Standard (JHU Cases)
S43
Weekly Confirmed Cases (Nevada)
10000
0
1
0
$P(R_t > 1)$
Naive
Single Source ("How long does covid last?")
Single Source ("chest pain")
Multiple Sources
2020-03-21
2020-06-20
2020-10-03
2021-03-20
2021-07-03
2021-11-13
Weekly Confirmed Cases (New Hampshire)
10000
5000
0
1
0
$P(R_t > 1)$
Naive
Single Source ("How long does covid last?")
Single Source ("chest pain")
Multiple Sources
2020-03-21
2020-09-19
2021-03-27
2021-07-31
2021-11-13
Weekly Confirmed Cases (New Jersey)
50000
0
1
0
$P(R_t > 1)$
Naive
Single Source ("How long does covid last?")
Single Source ("chest pain")
Multiple Sources
2020-03-21
2020-09-26
2021-01-16
2021-03-27
2021-07-24
2021-11-20

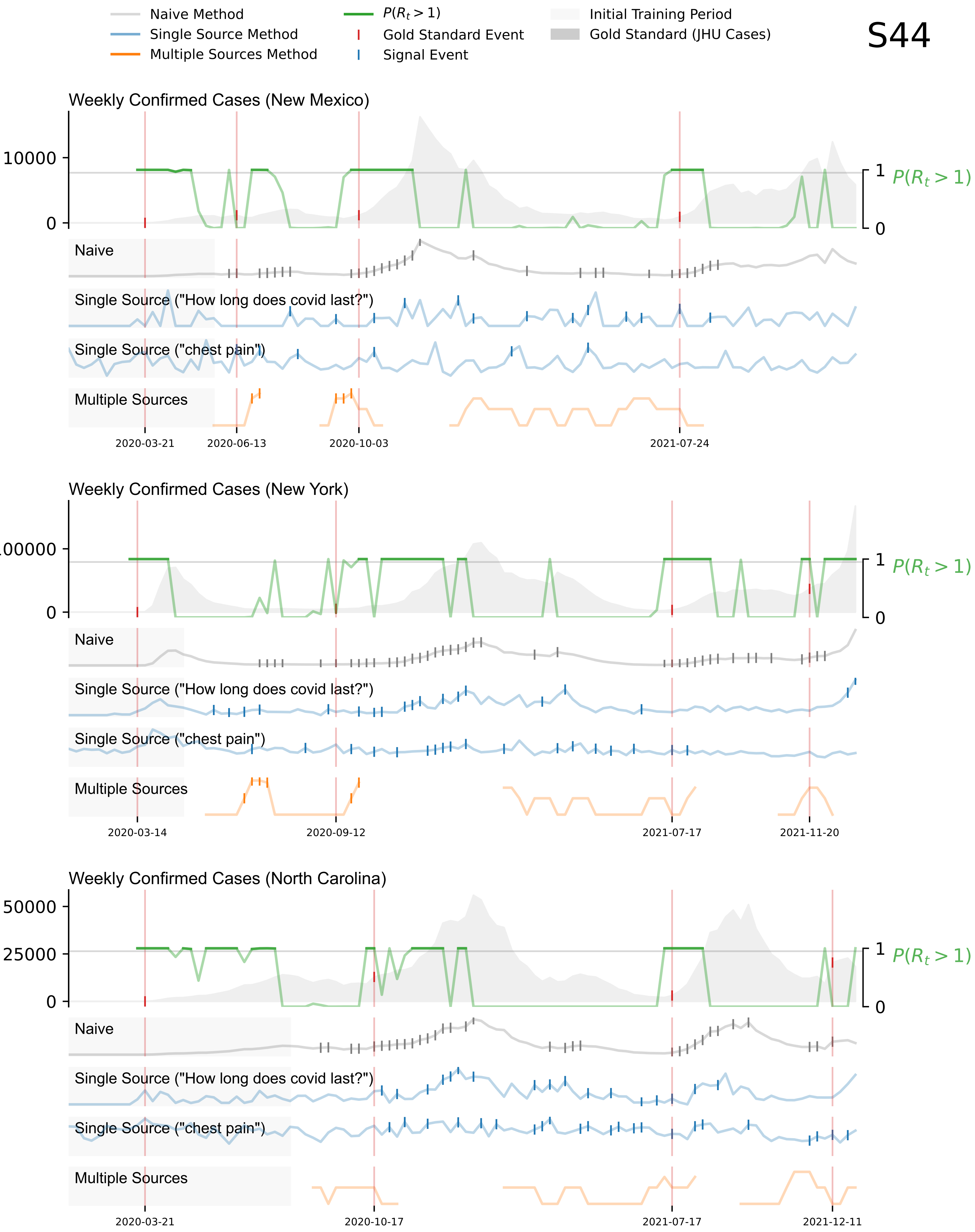

Naive Method
Single Source Method
Multiple Sources Method
$P(R_t > 1)$
Gold Standard Event
Signal Event
Initial Training Period
Gold Standard (JHU Cases)
S44
Weekly Confirmed Cases (New Mexico)
10000
0
1
0
$P(R_t > 1)$
Naive
Single Source ("How long does covid last?")
Single Source ("chest pain")
Multiple Sources
2020-03-21
2020-06-13
2020-10-03
2021-07-24
Weekly Confirmed Cases (New York)
100000
0
Naive
Single Source ("How long does covid last?")
Single Source ("chest pain")
Multiple Sources
2020-03-14
2020-09-12
2021-07-17
2021-11-20
Weekly Confirmed Cases (North Carolina)
50000
25000
0
Naive
Single Source ("How long does covid last?")
Single Source ("chest pain")
Multiple Sources
2020-03-21
2020-10-17
2021-07-17
2021-12-11

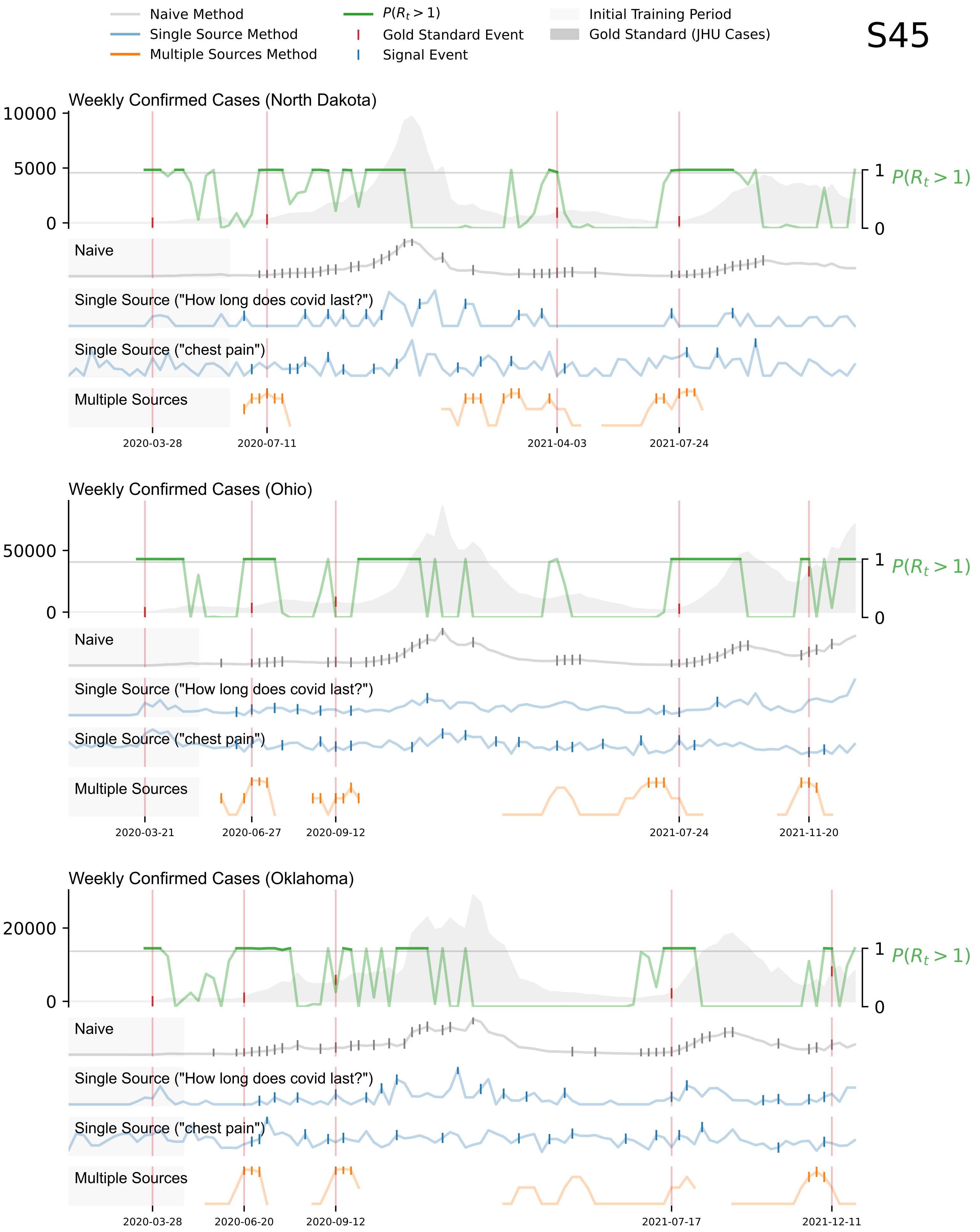

S45
Naive Method
Single Source Method
Multiple Sources Method
$P(R_t > 1)$
Gold Standard Event
Signal Event
Initial Training Period
Gold Standard (JHU Cases)
Weekly Confirmed Cases (North Dakota)
10000
5000
0
1
0
Naive
Single Source ("How long does covid last?")
Single Source ("chest pain")
Multiple Sources
2020-03-28
2020-07-11
2021-04-03
2021-07-24
Weekly Confirmed Cases (Ohio)
50000
0
Naive
Single Source ("How long does covid last?")
Single Source ("chest pain")
Multiple Sources
2020-03-21
2020-06-27
2020-09-12
2021-07-24
2021-11-20
Weekly Confirmed Cases (Oklahoma)
20000
0
Naive
Single Source ("How long does covid last?")
Single Source ("chest pain")
Multiple Sources
2020-03-28
2020-06-20
2020-09-12
2021-07-17
2021-12-11

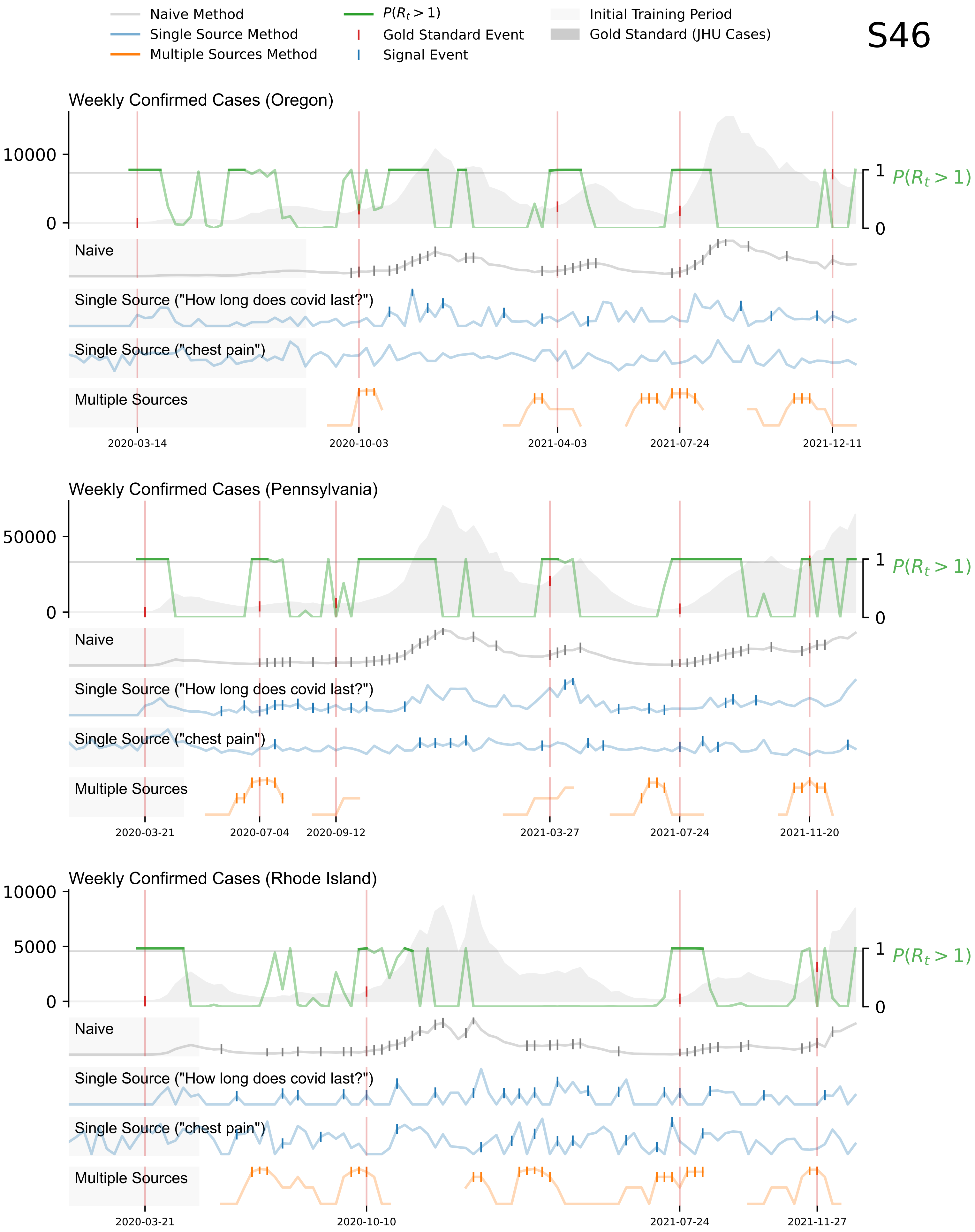

S46
Naive Method
Single Source Method
Multiple Sources Method
$P(R_t > 1)$
Gold Standard Event
Signal Event
Initial Training Period
Gold Standard (JHU Cases)
Weekly Confirmed Cases (Oregon)
10000
0
1
0
$P(R_t > 1)$
Naive
Single Source ("How long does covid last?")
Single Source ("chest pain")
Multiple Sources
2020-03-14
2020-10-03
2021-04-03
2021-07-24
2021-12-11
Weekly Confirmed Cases (Pennsylvania)
50000
0
1
0
$P(R_t > 1)$
Naive
Single Source ("How long does covid last?")
Single Source ("chest pain")
Multiple Sources
2020-03-21
2020-07-04
2020-09-12
2021-03-27
2021-07-24
2021-11-20
Weekly Confirmed Cases (Rhode Island)
10000
5000
0
1
0
$P(R_t > 1)$
Naive
Single Source ("How long does covid last?")
Single Source ("chest pain")
Multiple Sources
2020-03-21
2020-10-10
2021-07-24
2021-11-27

S47

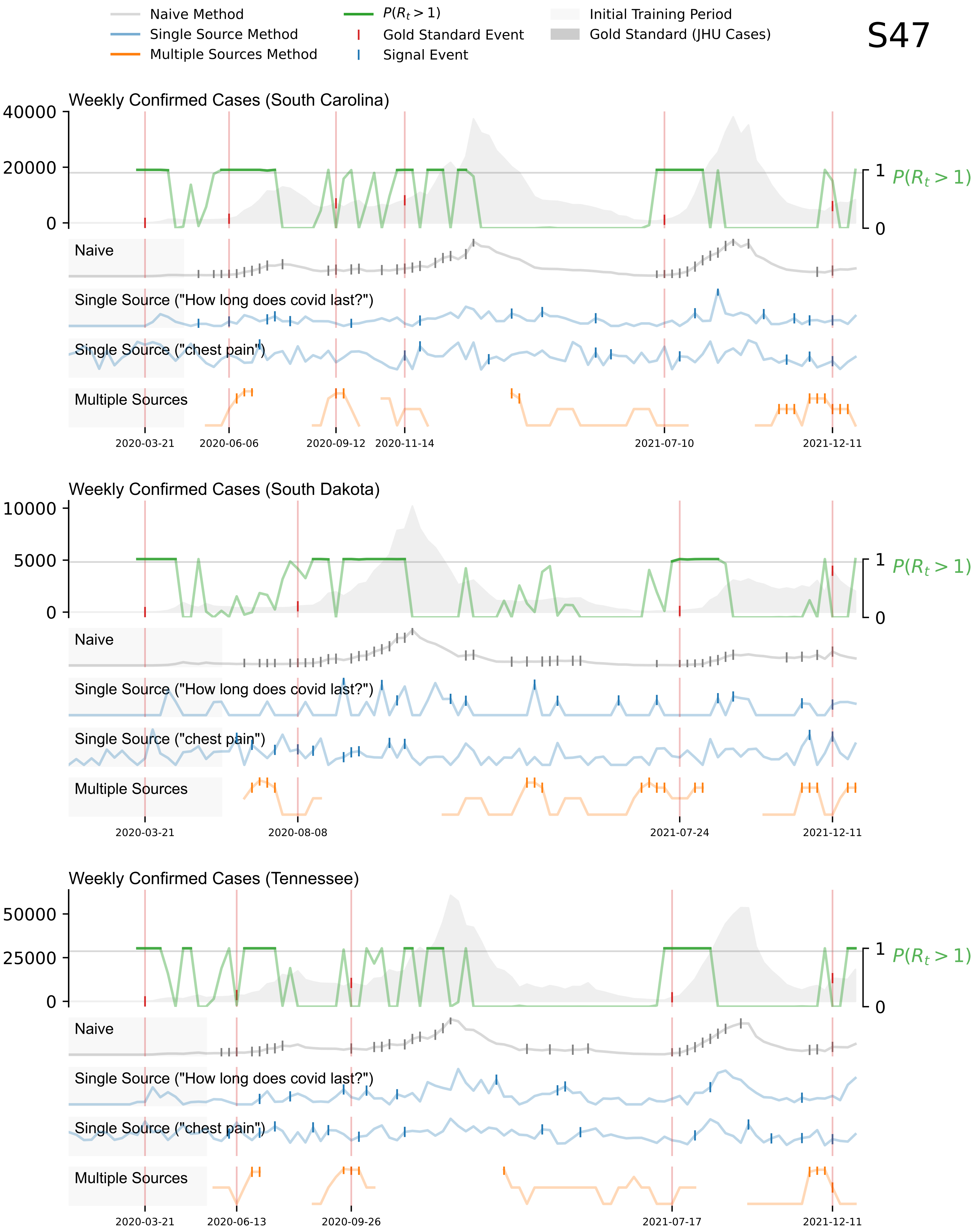

Naive Method
Single Source Method
Multiple Sources Method
$P(R_t > 1)$
Gold Standard Event
Signal Event
Initial Training Period
Gold Standard (JHU Cases)
Weekly Confirmed Cases (South Carolina)
40000
20000
0
1
0
$P(R_t > 1)$
Naive
Single Source ("How long does covid last?")
Single Source ("chest pain")
Multiple Sources
2020-03-21
2020-06-06
2020-09-12
2020-11-14
2021-07-10
2021-12-11
Weekly Confirmed Cases (South Dakota)
10000
5000
0
Naive
Single Source ("How long does covid last?")
Single Source ("chest pain")
Multiple Sources
2020-03-21
2020-08-08
2021-07-24
2021-12-11
Weekly Confirmed Cases (Tennessee)
50000
25000
0
Naive
Single Source ("How long does covid last?")
Single Source ("chest pain")
Multiple Sources
2020-03-21
2020-06-13
2020-09-26
2021-07-17
2021-12-11

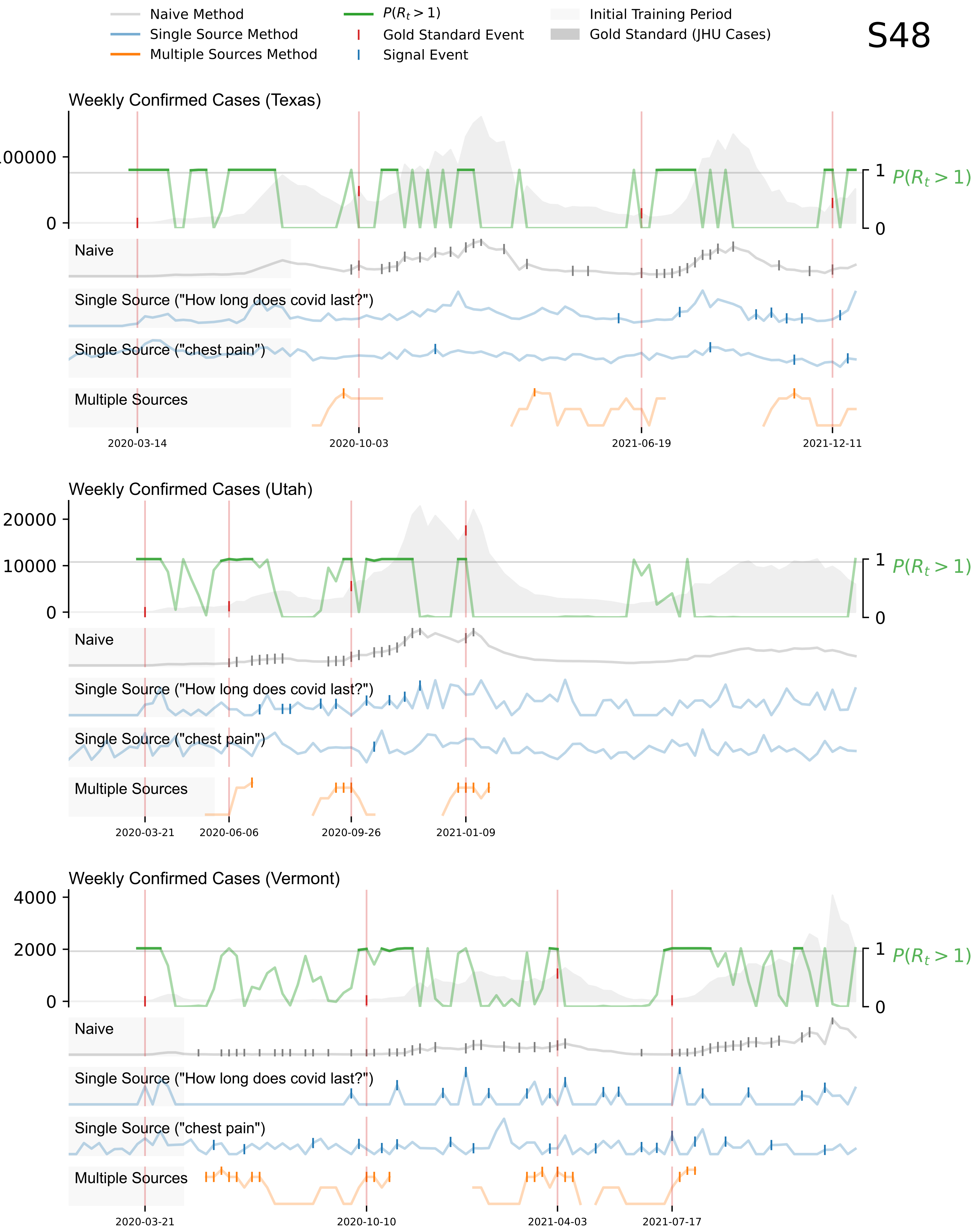

Naive Method
Single Source Method
Multiple Sources Method
$P(R_t > 1)$
Gold Standard Event
Signal Event
Initial Training Period
Gold Standard (JHU Cases)
S48
Weekly Confirmed Cases (Texas)
100000
0
1
0
$P(R_t > 1)$
Naive
Single Source ("How long does covid last?")
Single Source ("chest pain")
Multiple Sources
2020-03-14
2020-10-03
2021-06-19
2021-12-11
Weekly Confirmed Cases (Utah)
20000
10000
0
2020-03-21
2020-06-06
2020-09-26
2021-01-09
Weekly Confirmed Cases (Vermont)
4000
2000
0
2020-03-21
2020-10-10
2021-04-03
2021-07-17

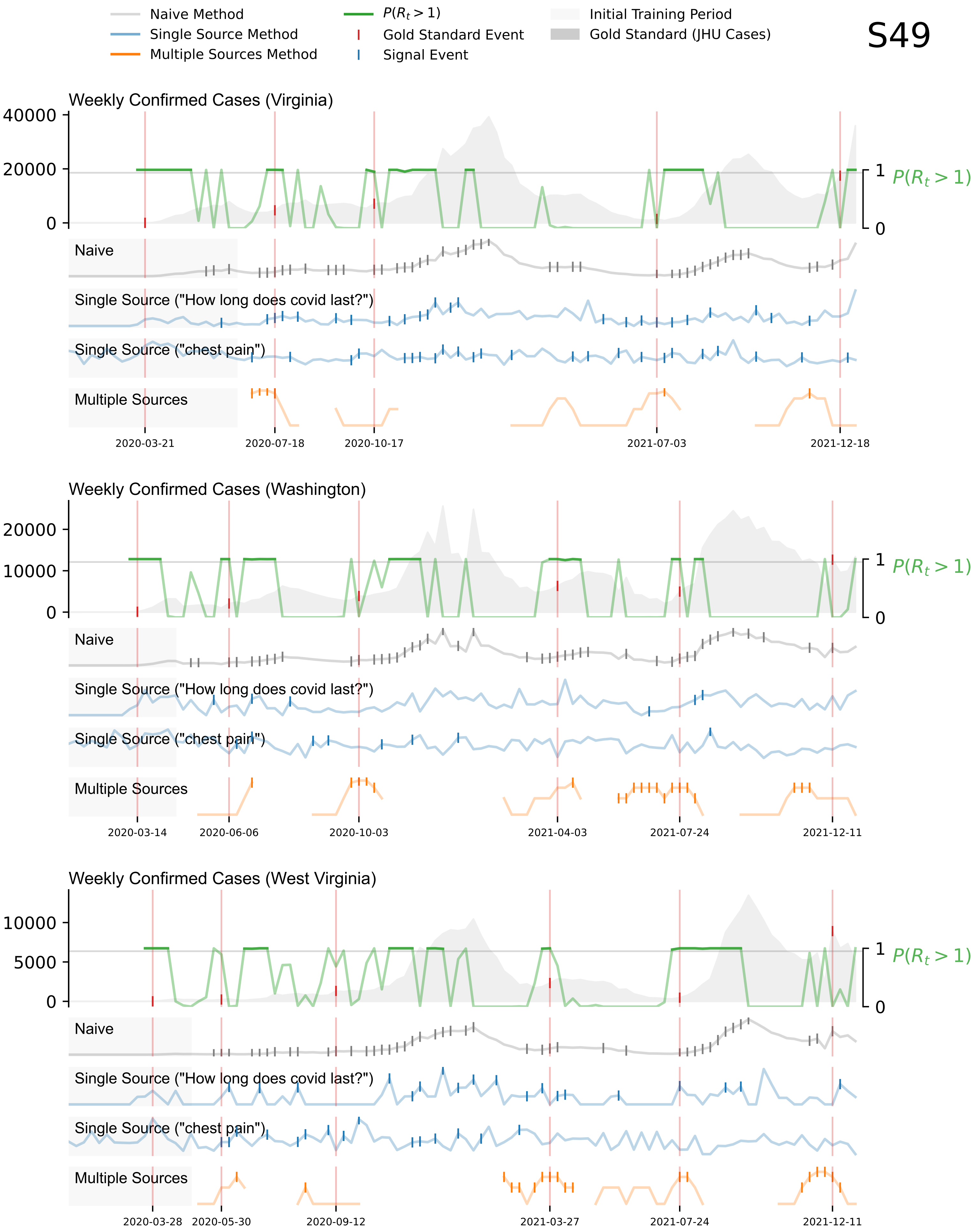

Naive Method
Single Source Method
Multiple Sources Method
$P(R_t > 1)$
Gold Standard Event
Signal Event
Initial Training Period
Gold Standard (JHU Cases)
S49
Weekly Confirmed Cases (Virginia)
40000
20000
0
1
0
$P(R_t > 1)$
Naive
Single Source ("How long does covid last?")
Single Source ("chest pain")
Multiple Sources
2020-03-21
2020-07-18
2020-10-17
2021-07-03
2021-12-18
Weekly Confirmed Cases (Washington)
20000
10000
0
Naive
Single Source ("How long does covid last?")
Single Source ("chest pain")
Multiple Sources
2020-03-14
2020-06-06
2020-10-03
2021-04-03
2021-07-24
2021-12-11
Weekly Confirmed Cases (West Virginia)
10000
5000
0
Naive
Single Source ("How long does covid last?")
Single Source ("chest pain")
Multiple Sources
2020-03-28
2020-05-30
2020-09-12
2021-03-27
2021-07-24
2021-12-11

Figures S50-S139.
Visualization of the multiple sources model as a function of time. The model's output (orange) is compared against the onsets (vertical gray tick marks) of the COVID-19 reported cases (gray-filled curve). As more information becomes available, the EWS model is re-trained to select the proxies that best fit the training data available (represented by the blue shading on each Figure). A list of the selected proxies for each model, along with a performance table are also presented.

Proxies selected by the Multiple Sources method

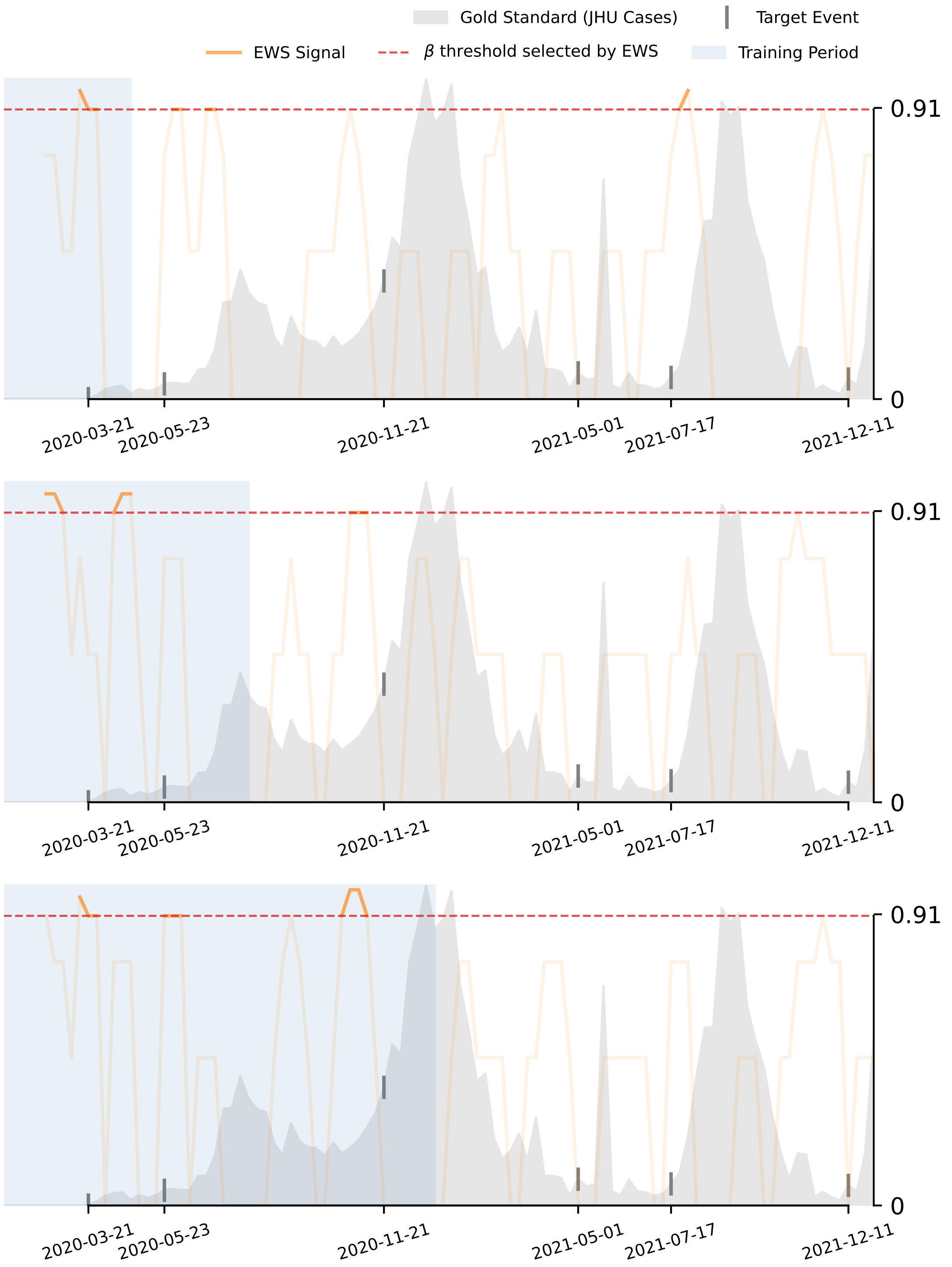

Model 1

(GT) covid
(GT) covid-19
(GT) covid symptoms
(GT) quarantine
(GT) Acute bronchitis
(GT) Bronchitis

Model 2

(GT) covid
(GT) covid-19
(GT) Abdominal pain
(GT) Asphyxia
(GT) Dizziness
(GT) Vertigo

Model 3

(GT) covid
(GT) covid-19
(GT) Abdominal pain
(GT) Asphyxia
(GT) fever
(GT) covid symptoms

Performance as a function of β

Model 1

	Training			Out of sample		
β	tp	fp	fn	tp	fp	fn
1.00	0	0	1	0	0	5
0.99	0	0	1	0	0	5
0.96	0	0	1	0	1	4
0.91	1	0	0	2	7	1
0.76	1	1	0	4	13	1
0.46	1	2	0	5	30	0
0.00	1	2	0	5	56	0

Model 2

β	tp	fp	fn	tp	fp	fn
1.00	0	0	2	0	0	4
0.99	0	0	2	0	0	4
0.96	0	0	2	0	0	4
0.91	2	2	0	2	0	2
0.76	2	3	0	2	8	1
0.46	2	5	0	4	29	0
0.00	2	6	0	4	46	0

Model 3

β	tp	fp	fn	tp	fp	fn
1.00	0	0	3	0	0	3
0.99	0	0	3	0	0	3
0.96	1	0	2	0	0	3
0.91	2	0	1	1	0	2
0.76	3	6	0	3	4	0
0.46	3	10	0	3	19	0
0.00	3	16	0	3	31	0

Proxies selected by the Multiple Sources method

Performance as a function of β

Model 1

(GT) covid
(GT) covid-19
(GT) chest pain
(GT) covid symptoms
(GT) Chest pain
(GT) Wheeze

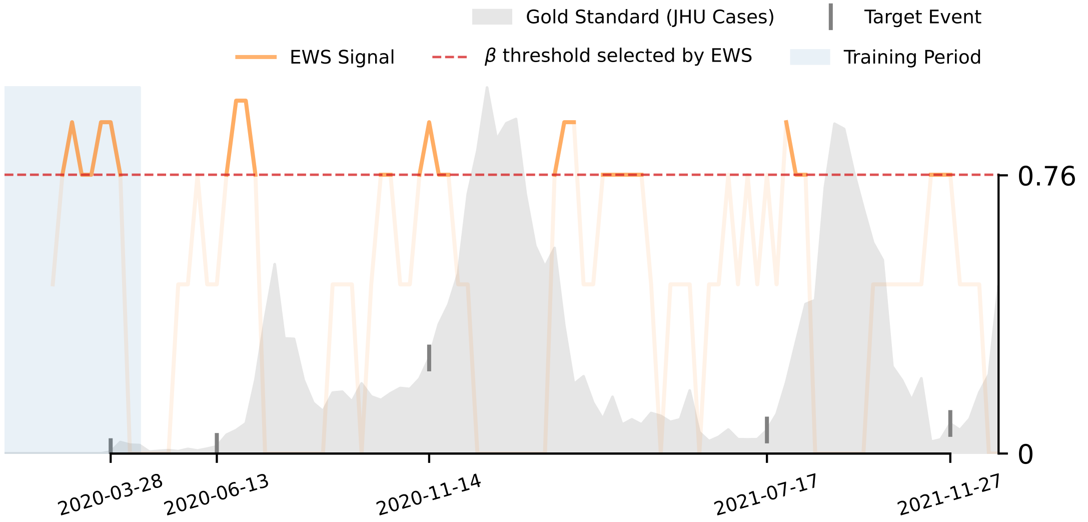

β	Training tp	Training fp	Training fn	Out of sample tp	Out of sample fp	Out of sample fn
1.00	0	0	1	0	0	4
0.99	0	0	1	0	0	4
0.96	0	0	1	0	2	3
0.91	0	0	1	1	5	1
0.76	1	0	0	4	17	0
0.46	1	2	0	4	34	0
0.00	1	3	0	4	61	0

Model 2

(GT) chest pain
(GT) Chest pain
(GT) Chills

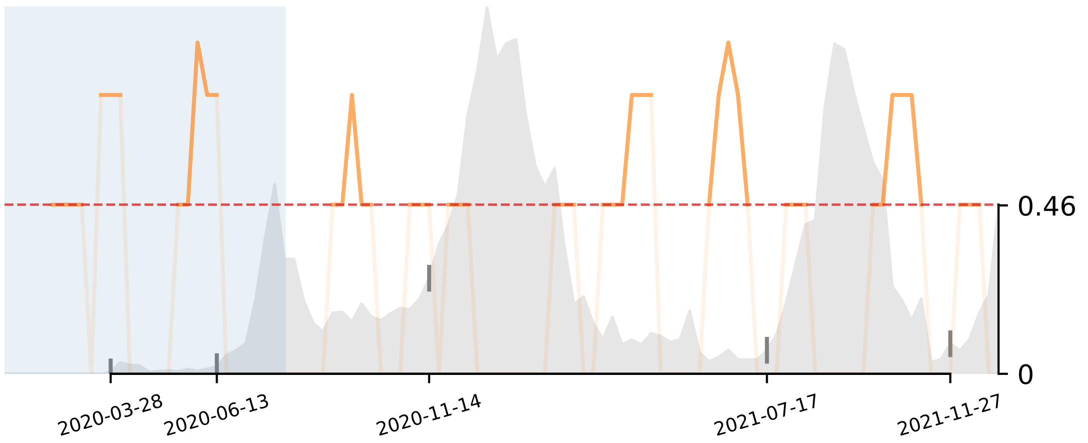

β	Training tp	Training fp	Training fn	Out of sample tp	Out of sample fp	Out of sample fn
0.91	0	0	2	1	0	2
0.76	1	0	1	2	4	1
0.46	2	1	0	3	24	0
0.00	2	3	0	3	53	0

Model 3

(GT) chest pain
(GT) covid
(GT) covid-19
(GT) covid symptoms
(GT) Diarrhea
(GT) Otitis

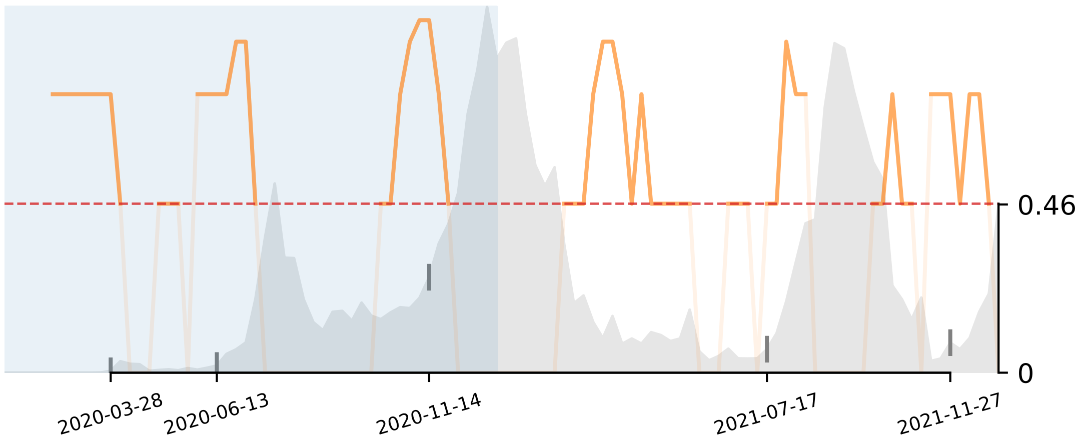

β	Training tp	Training fp	Training fn	Out of sample tp	Out of sample fp	Out of sample fn
1.00	0	0	3	0	0	2
0.99	0	0	3	0	0	2
0.96	0	0	3	0	0	2
0.91	1	0	2	0	3	1
0.76	1	2	2	1	10	0
0.46	3	6	0	2	24	0
0.00	3	12	0	2	38	0

Proxies selected by the Multiple Sources method

Model 1

(GT) covid
(GT) covid-19
(GT) chest pain
(GT) covid symptoms
(GT) fever
(GT) Anxiety

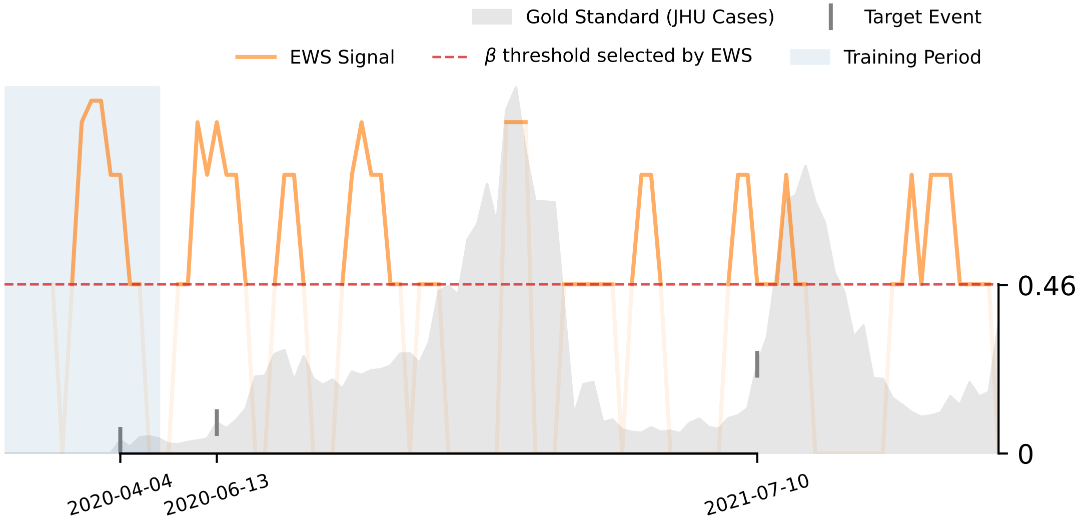

Performance as a function of β

β	Training tp	Training fp	Training fn	Out of sample tp	Out of sample fp	Out of sample fn
1.00	0	0	1	0	0	2
0.99	0	0	1	0	0	2
0.96	0	0	1	0	0	2
0.91	1	0	0	1	4	1
0.76	1	0	0	2	18	0
0.46	1	0	0	2	46	0
0.00	1	4	0	2	74	0

Model 2

(GT) covid
(GT) covid symptoms
(GT) fever
(GT) Anxiety
(GT) Diarrhea

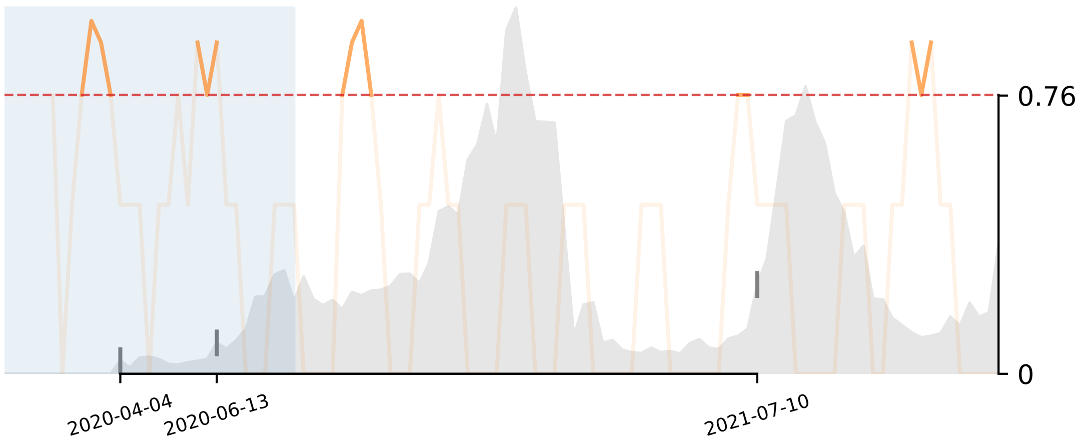

β	Training tp	Training fp	Training fn	Out of sample tp	Out of sample fp	Out of sample fn
0.99	0	0	2	0	0	1
0.96	0	0	2	0	1	1
0.91	1	0	1	0	4	1
0.76	2	0	0	1	8	0
0.46	2	1	0	1	32	0
0.00	2	11	0	1	66	0

Model 3

(GT) covid
(GT) covid symptoms
(GT) Nasal congestion
(GT) Anxiety
(GT) Diarrhea
(GT) fever

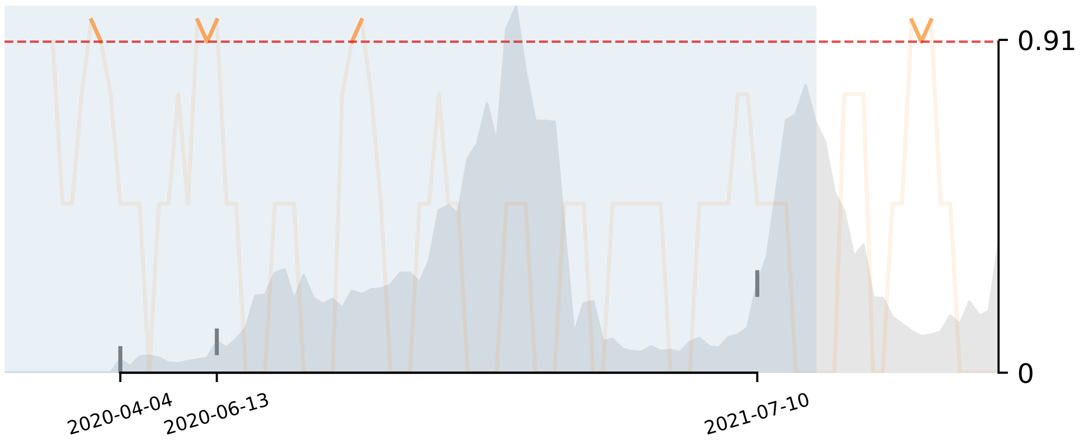

β	Training tp	Training fp	Training fn	Out of sample tp	Out of sample fp	Out of sample fn
1.00	0	0	3	0	0	0
0.99	0	0	3	0	0	0
0.96	0	0	3	0	2	0
0.91	2	1	1	0	3	0
0.76	2	3	1	0	6	0
0.46	3	6	0	0	10	0
0.00	3	39	0	0	19	0

Proxies selected by the Multiple Sources method

Model 1

(GT) loss of smell
(GT) chest tightness
(GT) covid-19 who
(GT) sars-cov-2
(GT) covid
(GT) covid-19

Model 2

(GT) covid symptoms
(GT) Acute bronchitis
(GT) Bronchitis
(GT) Cough
(GT) Phlegm
(GT) Chest pain

Model 3

(GT) covid symptoms
(GT) Acute bronchitis
(GT) Bronchitis
(GT) Cough
(GT) Phlegm
(GT) Chills

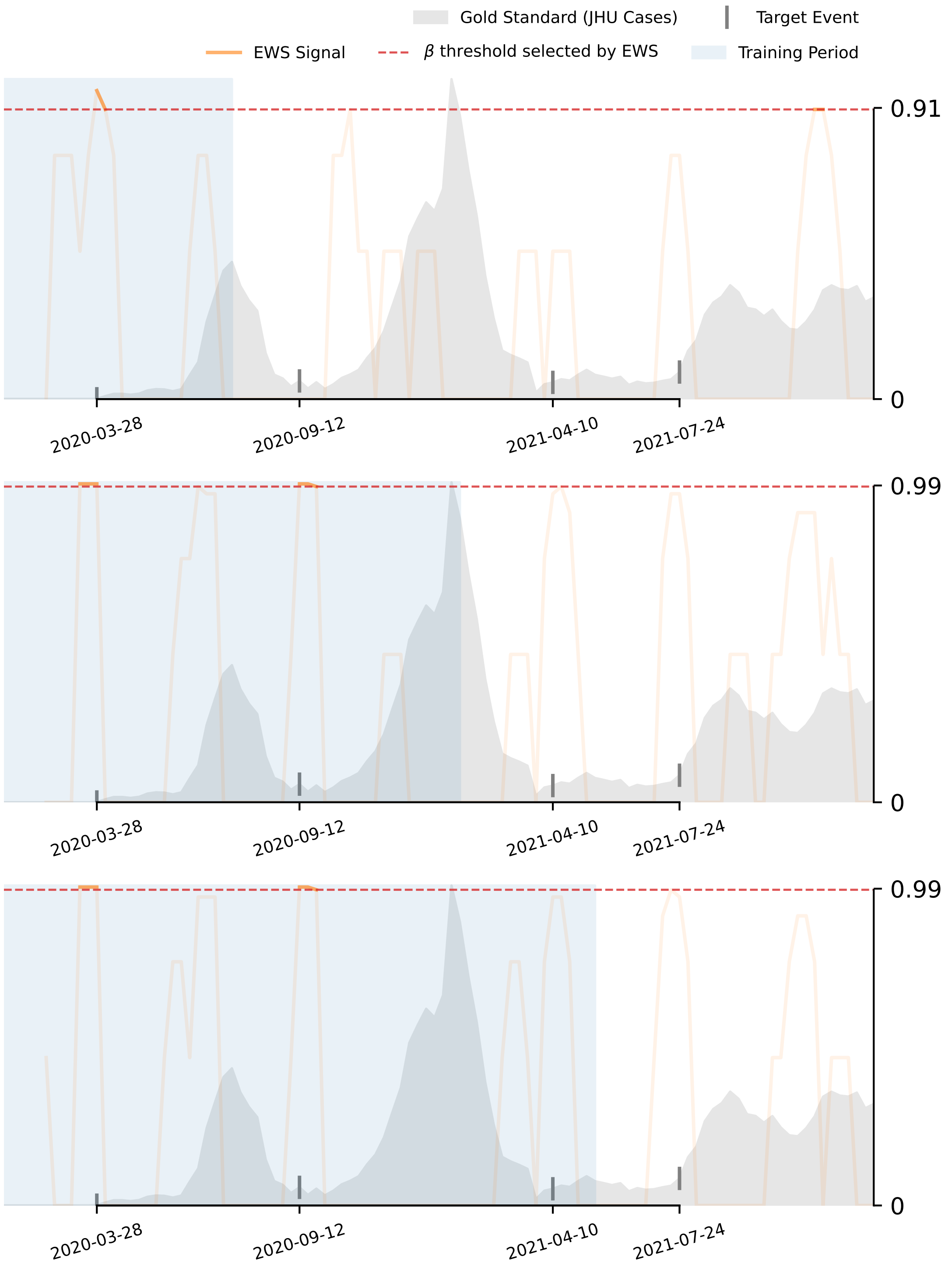

Performance as a function of β

Model 1

β	Training tp	Training fp	Training fn	Out of sample tp	Out of sample fp	Out of sample fn
1.00	0	0	1	0	0	3
0.99	0	0	1	0	0	3
0.96	0	0	1	0	0	3
0.91	1	0	0	0	3	3
0.76	1	1	0	1	7	2
0.46	1	5	0	2	20	1
0.00	1	7	0	3	55	0

Model 2

β	Training tp	Training fp	Training fn	Out of sample tp	Out of sample fp	Out of sample fn
1.00	0	0	2	0	0	2
0.99	2	1	0	0	1	1
0.96	2	3	0	2	1	0
0.91	2	5	0	2	5	0
0.76	2	5	0	2	8	0
0.46	2	7	0	2	17	0
0.00	2	11	0	2	35	0

Model 3

β	Training tp	Training fp	Training fn	Out of sample tp	Out of sample fp	Out of sample fn
1.00	0	0	3	0	0	1
0.99	2	1	1	1	0	0
0.96	2	2	1	1	0	0
0.91	3	6	0	1	2	0
0.76	3	6	0	1	5	0
0.46	3	10	0	1	10	0
0.00	3	14	0	1	26	0

Proxies selected by the Multiple Sources method

Performance as a function of β

Model 1

(GT) covid
(GT) covid-19
(GT) chest pain
(GT) covid symptoms
(GT) quarantine
(GT) Acute bronchitis

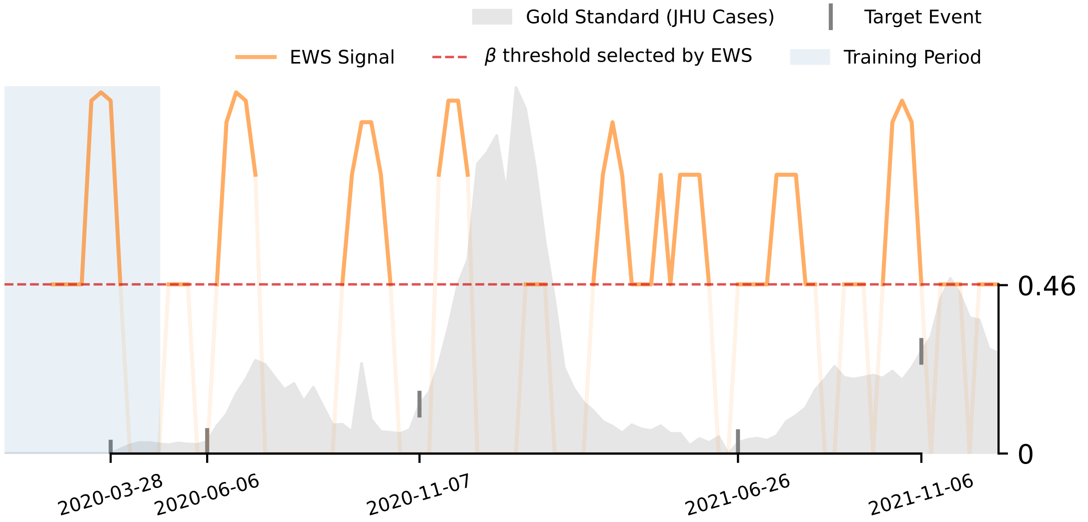

	Training			Out of sample		
β	tp	fp	fn	tp	fp	fn
1.00	0	0	1	0	0	4
0.99	0	0	1	0	1	4
0.96	1	0	0	1	4	3
0.91	1	0	0	2	6	1
0.76	1	0	0	3	16	0
0.46	1	0	0	4	39	0
0.00	1	3	0	4	61	0

Model 2

(GT) Ageusia
(GT) Chills
(GT) Nasal congestion
Apple Mobility (driving)
Apple Mobility (transit)
(GT) covid symptoms

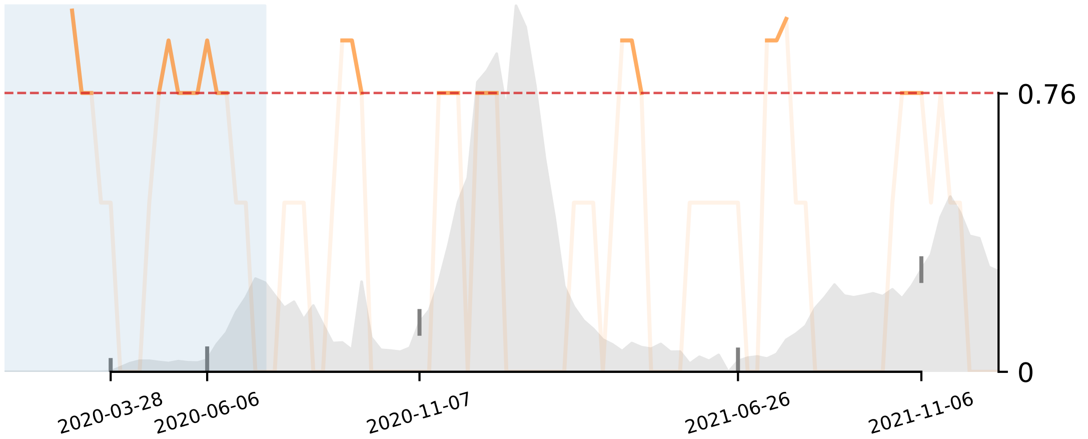

	Training			Out of sample		
β	tp	fp	fn	tp	fp	fn
1.00	0	0	2	0	0	3
0.99	0	0	2	0	0	3
0.96	1	0	1	0	1	3
0.91	1	0	1	0	7	3
0.76	2	0	0	2	15	1
0.46	2	3	0	3	28	0
0.00	2	6	0	3	55	0

Model 3

(GT) Ageusia
(GT) Nasal congestion
Apple Mobility (driving)
up2date
(GT) covid-19
(GT) covid symptoms

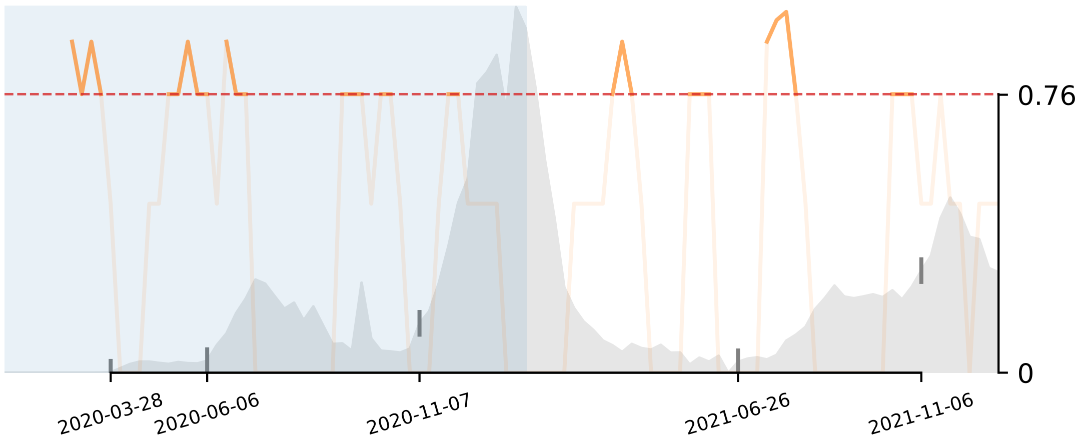

	Training			Out of sample		
β	tp	fp	fn	tp	fp	fn
1.00	0	0	3	0	0	2
0.99	0	0	3	0	1	2
0.96	0	0	3	0	2	2
0.91	0	0	3	0	4	2
0.76	2	1	1	2	8	0
0.46	3	8	0	2	20	0
0.00	3	17	0	2	35	0

Proxies selected by the Multiple Sources method

Model 1

(GT) covid
(GT) how long does covid last
(GT) covid-19
(GT) chest pain
(GT) covid symptoms
(GT) chest tightness

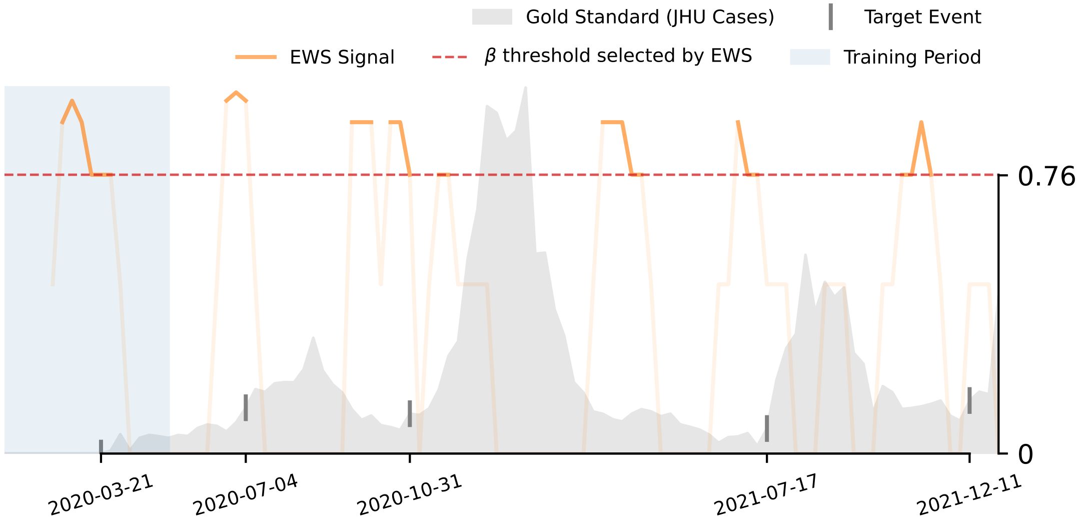

Performance as a function of β

β	Training tp	Training fp	Training fn	Out of sample tp	Out of sample fp	Out of sample fn
1.00	0	0	1	0	0	4
0.99	0	0	1	1	0	3
0.96	0	0	1	1	0	3
0.91	1	0	0	4	3	0
0.76	1	0	0	4	8	0
0.46	1	1	0	4	25	0
0.00	1	3	0	4	58	0

Model 2

(GT) covid
(GT) how long does covid last
(GT) covid-19
(GT) chest pain
(GT) covid symptoms
(GT) quarantine

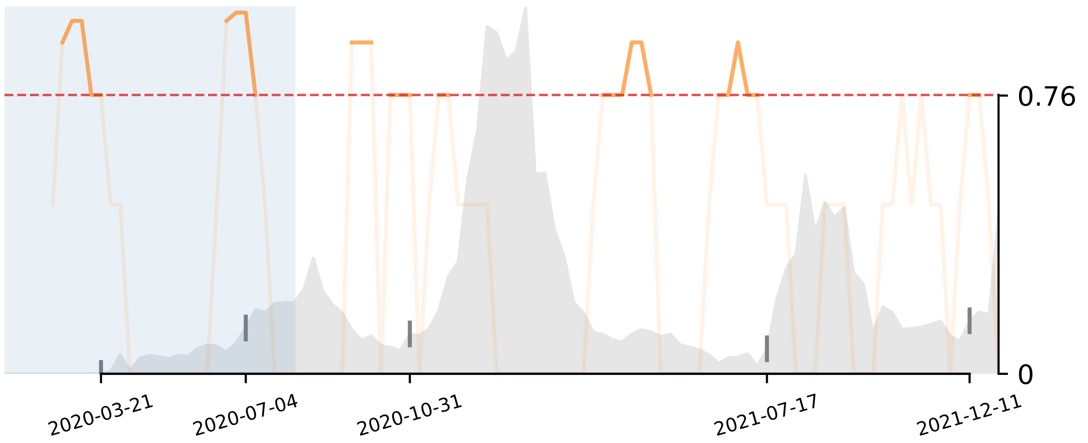

β	Training tp	Training fp	Training fn	Out of sample tp	Out of sample fp	Out of sample fn
1.00	0	0	2	0	0	3
0.99	0	0	2	0	0	3
0.96	1	0	1	0	0	3
0.91	2	0	0	2	2	1
0.76	2	0	0	3	10	0
0.46	2	1	0	3	24	0
0.00	2	5	0	3	52	0

Model 3

(GT) chest pain
(GT) covid symptoms
(GT) Acute bronchitis
(GT) Sore throat
(GT) Throat irritation
(GT) covid

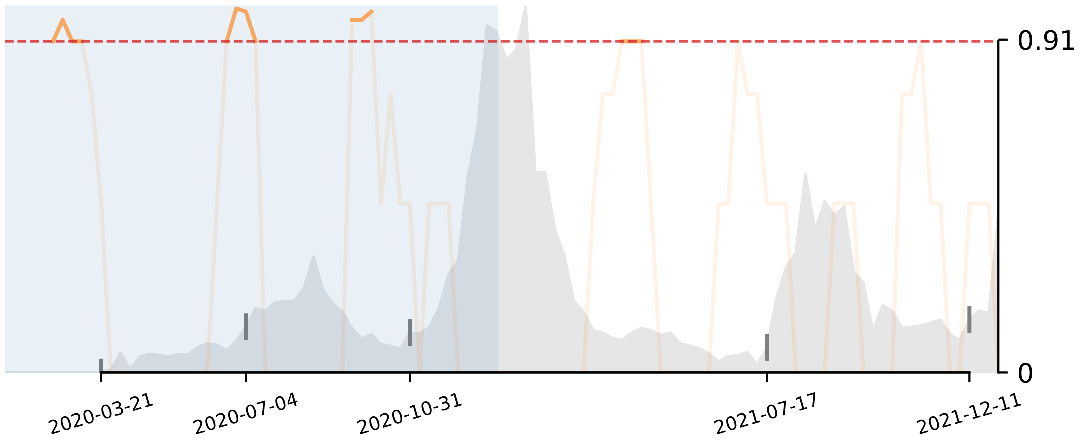

β	Training tp	Training fp	Training fn	Out of sample tp	Out of sample fp	Out of sample fn
1.00	0	0	3	0	0	2
0.99	1	0	2	0	0	2
0.96	2	0	1	0	0	2
0.91	3	2	0	2	3	0
0.76	3	4	0	2	6	0
0.46	3	4	0	2	15	0
0.00	3	7	0	2	38	0

Proxies selected by the Multiple Sources method

Model 1

(GT) covid
(GT) how long does covid last
(GT) covid-19
(GT) chest pain
(GT) covid symptoms
(GT) chest tightness

Model 2

(GT) fever
(GT) Ageusia
(GT) Chronic pain
(GT) Diarrhea
(GT) Fatigue
(GT) Otitis

Model 3

(GT) Ageusia
(GT) Asthma
(GT) loss of smell
(GT) chest tightness
Apple Mobility (transit)
(GT) chest pain

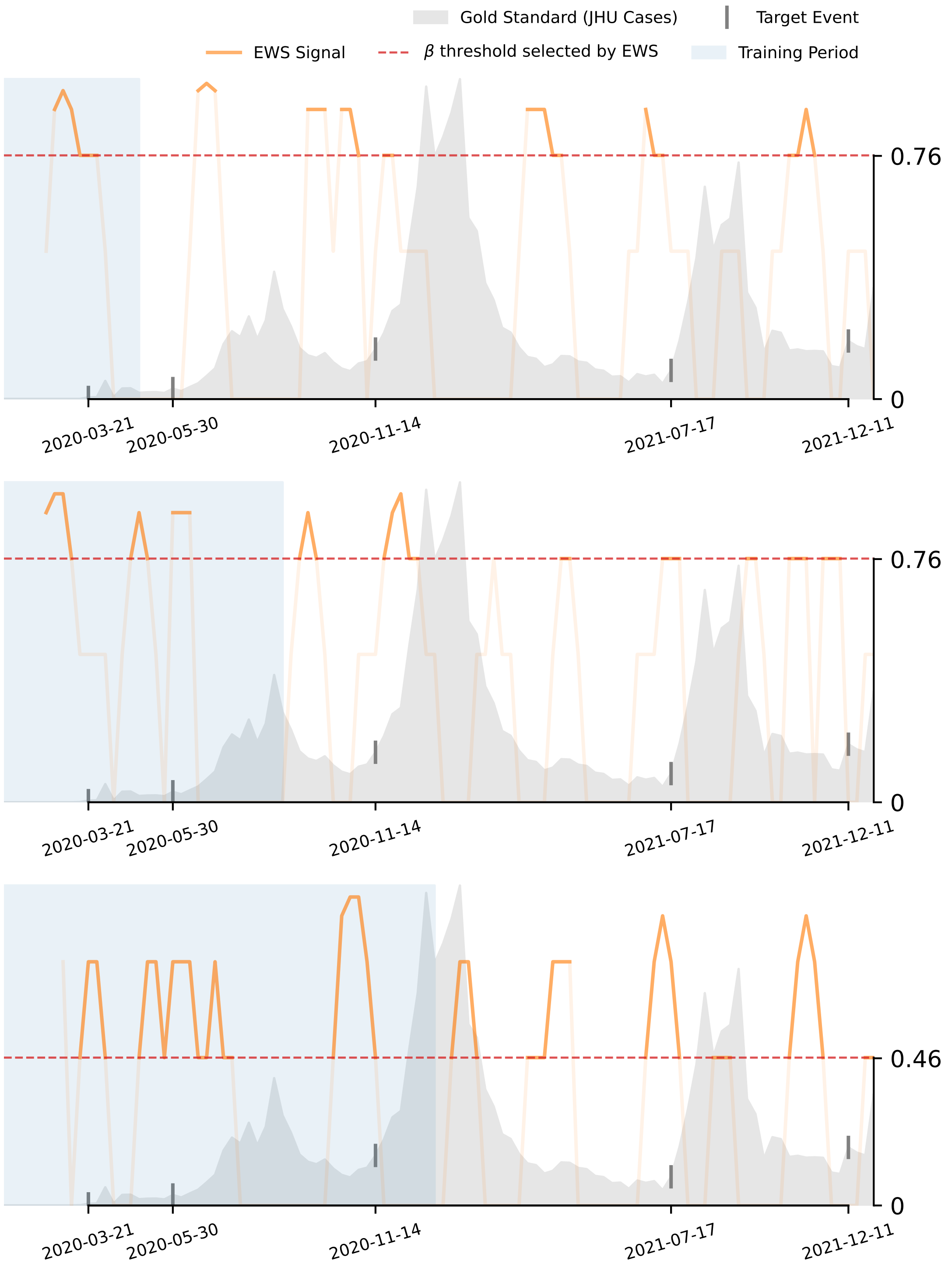

Performance as a function of β

Model 1

β	Training tp	Training fp	Training fn	Out of sample tp	Out of sample fp	Out of sample fn
1.00	0	0	1	0	0	4
0.99	0	0	1	0	1	4
0.96	0	0	1	0	3	4
0.91	1	0	0	3	8	1
0.76	1	0	0	3	13	1
0.46	1	1	0	3	30	0
0.00	1	3	0	4	62	0

Model 2

β	Training tp	Training fp	Training fn	Out of sample tp	Out of sample fp	Out of sample fn
1.00	0	0	2	0	0	3
0.99	0	0	2	0	0	3
0.96	0	0	2	0	1	3
0.91	1	0	1	0	3	2
0.76	2	3	0	2	15	0
0.46	2	4	0	3	28	0
0.00	2	7	0	3	49	0

Model 3

β	Training tp	Training fp	Training fn	Out of sample tp	Out of sample fp	Out of sample fn
1.00	0	0	3	0	0	2
0.99	0	0	3	0	0	2
0.96	0	0	3	0	0	2
0.91	1	0	2	2	0	0
0.76	1	0	2	2	5	0
0.46	3	4	0	2	17	0
0.00	3	10	0	2	38	0

Proxies selected by the Multiple Sources method

Model 1

(GT) covid
(GT) covid-19
(GT) loss taste
(GT) covid symptoms
(GT) quarantine
(GT) covid-19 who

Model 2

(GT) covid symptoms
(GT) Chronic pain
(GT) Diarrhea
Apple Mobility (transit)
(GT) nose bleed
(GT) Asphyxia

Model 3

(GT) covid symptoms
(GT) nose bleed
(GT) Asphyxia
(GT) loss of smell
(GT) covid
(GT) quarantine

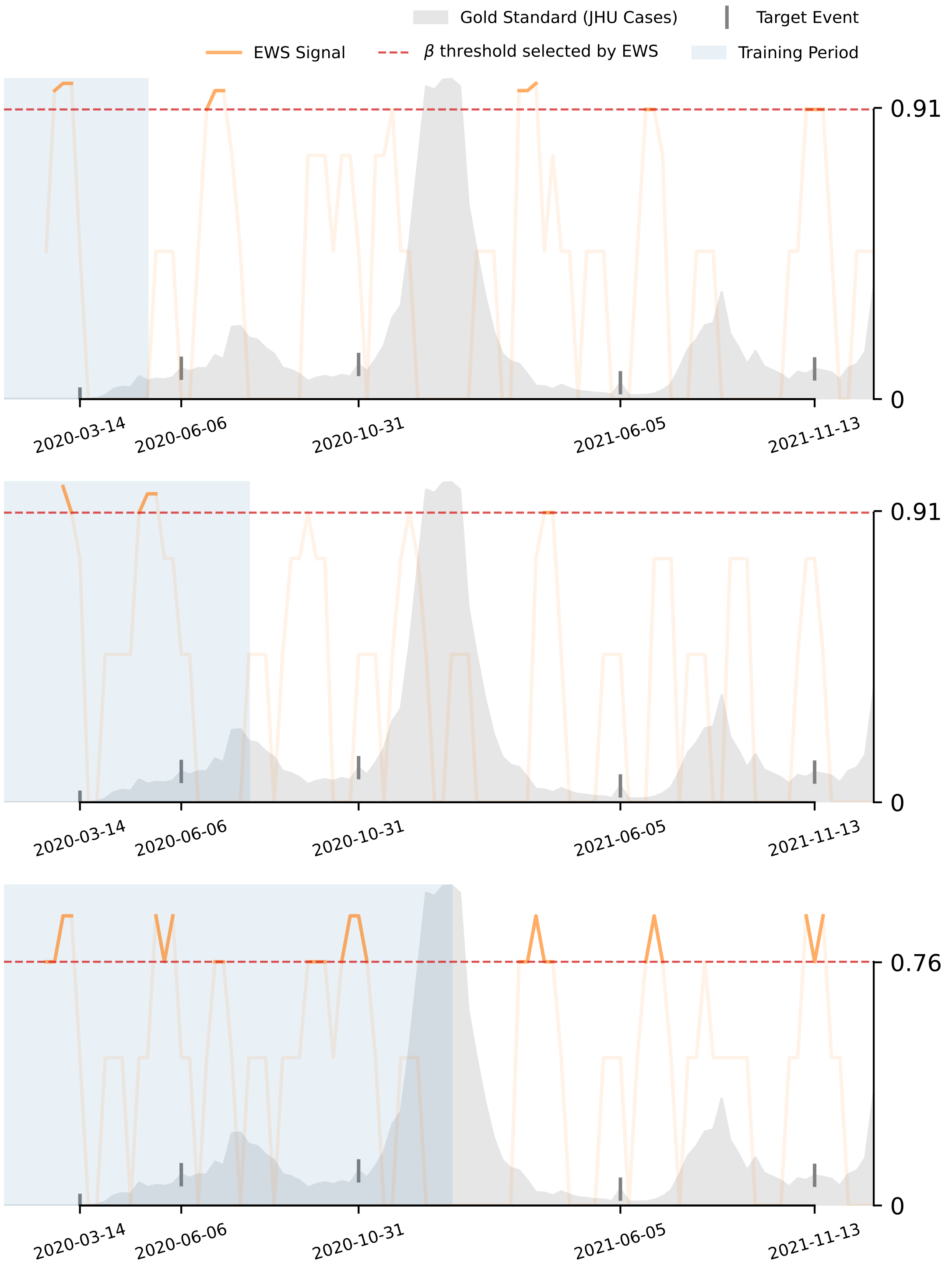

Performance as a function of β

Model 1

β	Training tp	Training fp	Training fn	Out of sample tp	Out of sample fp	Out of sample fn
1.00	0	0	1	0	0	4
0.99	0	0	1	0	1	4
0.96	1	0	0	0	5	4
0.91	1	0	0	1	10	3
0.76	1	0	0	2	15	2
0.46	1	0	0	4	32	0
0.00	1	0	0	4	61	0

Model 2

β	Training tp	Training fp	Training fn	Out of sample tp	Out of sample fp	Out of sample fn
1.00	0	0	2	0	0	3
0.99	0	0	2	0	0	3
0.96	1	0	1	0	0	3
0.91	2	0	0	1	3	2
0.76	2	1	0	2	14	1
0.46	2	1	0	3	29	0
0.00	2	7	0	3	53	0

Model 3

β	Training tp	Training fp	Training fn	Out of sample tp	Out of sample fp	Out of sample fn
1.00	0	0	3	0	0	2
0.99	0	0	3	0	0	2
0.96	0	0	3	0	0	2
0.91	0	0	3	1	3	1
0.76	3	0	0	1	10	1
0.46	3	5	0	2	22	0
0.00	3	22	0	2	36	0

Proxies selected by the Multiple Sources method

Model 1

(GT) covid
(GT) how long does covid last
(GT) covid-19
(GT) loss taste
(GT) chest pain
(GT) chest tightness

Model 2

(GT) how long does covid last
(GT) covid-19
(GT) covid symptoms
(GT) Acute bronchitis
(GT) Ageusia
(GT) Anosmia

Model 3

(GT) covid-19
(GT) loss of smell
(GT) covid
(GT) how long does covid last
(GT) covid symptoms
(GT) covid-19 who

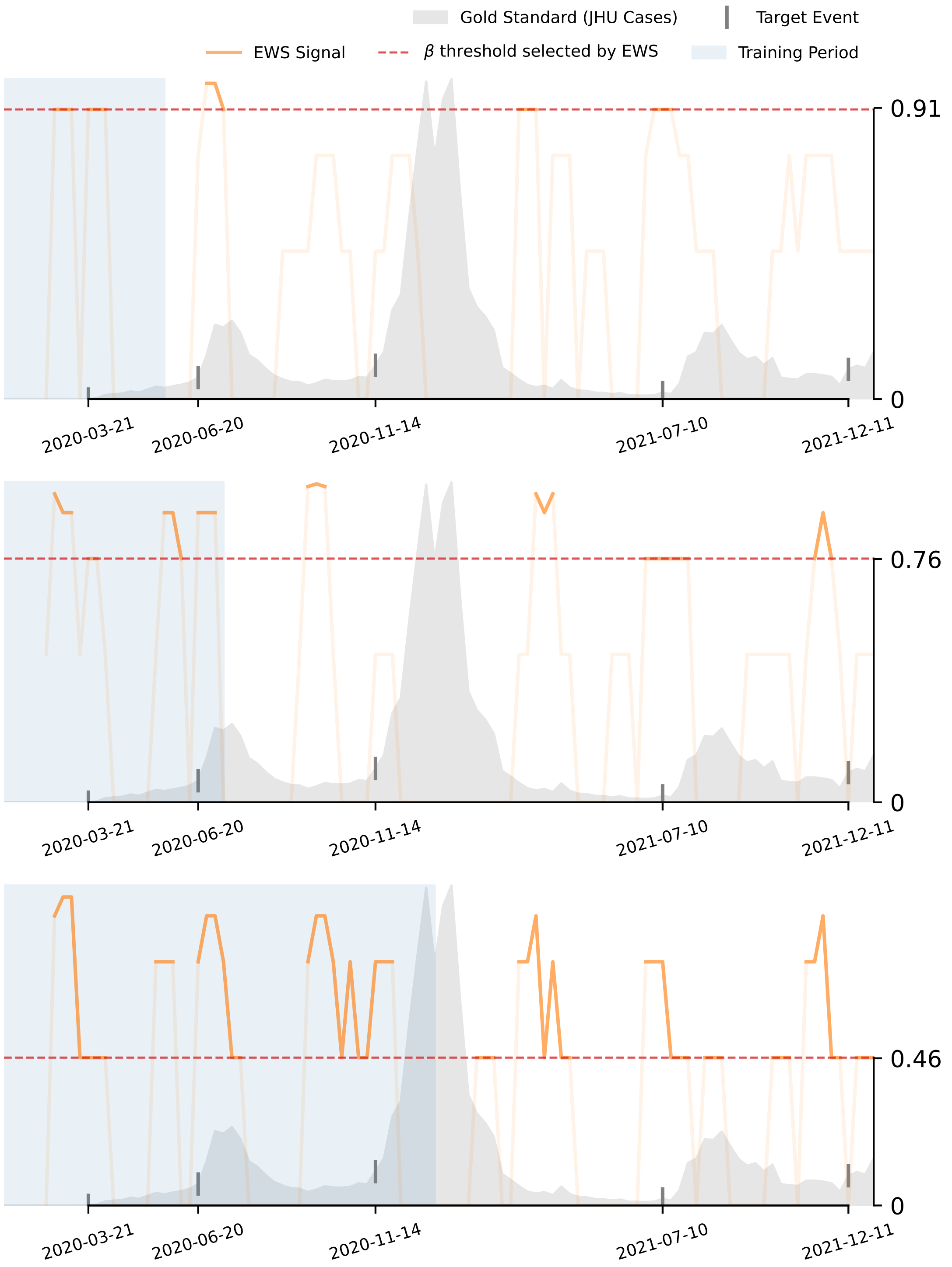

Performance as a function of β

Model 1

β	Training tp	Training fp	Training fn	Out of sample tp	Out of sample fp	Out of sample fn
1.00	0	0	1	0	0	4
0.99	0	0	1	0	2	3
0.96	0	0	1	0	2	3
0.91	0	0	1	1	7	2
0.76	1	2	0	4	17	0
0.46	1	2	0	4	34	0
0.00	1	2	0	4	59	0

Model 2

β	Training tp	Training fp	Training fn	Out of sample tp	Out of sample fp	Out of sample fn
1.00	0	0	2	0	1	3
0.99	0	0	2	1	2	2
0.96	0	0	2	1	4	2
0.91	1	0	1	2	5	1
0.76	2	2	0	3	8	0
0.46	2	3	0	3	24	0
0.00	2	6	0	3	56	0

Model 3

β	Training tp	Training fp	Training fn	Out of sample tp	Out of sample fp	Out of sample fn
1.00	0	0	3	0	0	2
0.99	0	0	3	0	0	2
0.96	0	0	3	0	0	2
0.91	1	0	2	1	1	1
0.76	1	4	2	2	4	0
0.46	3	10	0	2	22	0
0.00	3	14	0	2	38	0

Proxies selected by the Multiple Sources method

Performance as a function of β

Model 1

(GT) covid
(GT) how long does covid last
(GT) covid-19
(GT) loss taste
(GT) chest pain
(GT) covid symptoms

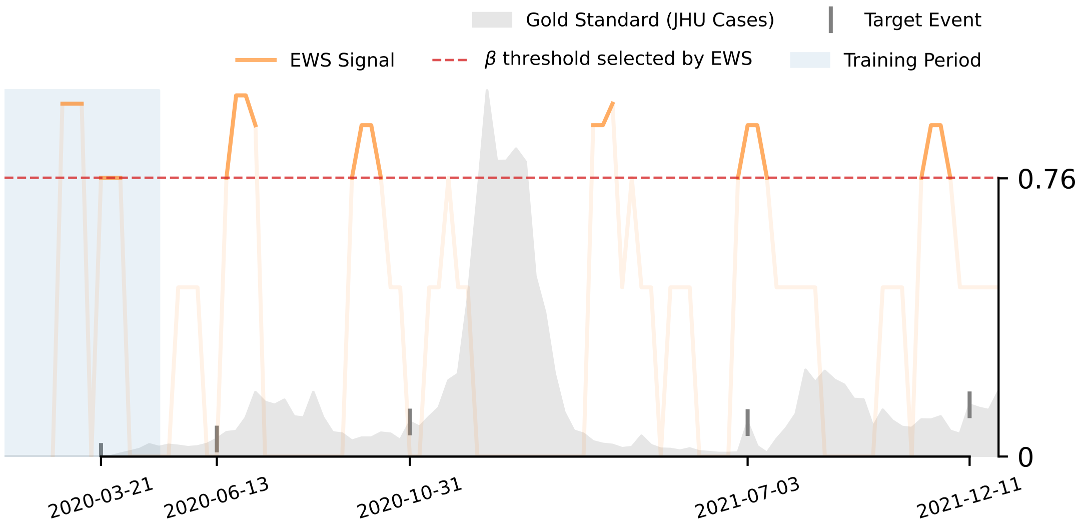

β	Training tp	Training fp	Training fn	Out of sample tp	Out of sample fp	Out of sample fn
1.00	0	0	1	0	0	4
0.99	0	0	1	0	2	3
0.96	0	0	1	0	3	3
0.91	1	0	0	3	7	0
0.76	1	0	0	3	11	0
0.46	1	2	0	4	31	0
0.00	1	2	0	4	60	0

Model 2

(GT) covid symptoms
(GT) nose bleed
(GT) Ageusia
(GT) Chills
(GT) Diarrhea
(GT) Fatigue

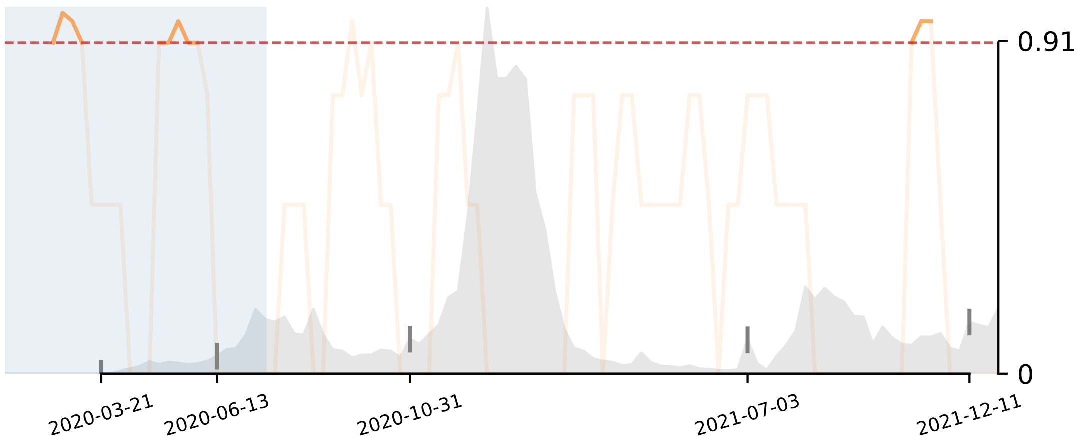

β	Training tp	Training fp	Training fn	Out of sample tp	Out of sample fp	Out of sample fn
1.00	0	0	2	0	0	3
0.99	0	0	2	0	0	3
0.96	1	0	1	2	0	1
0.91	2	0	0	2	1	1
0.76	2	3	0	3	12	0
0.46	2	3	0	3	27	0
0.00	2	5	0	3	55	0

Model 3

(GT) covid symptoms
(GT) Ageusia
(GT) nose bleed
(GT) Chest pain
(GT) loss of smell
Apple Mobility (transit)

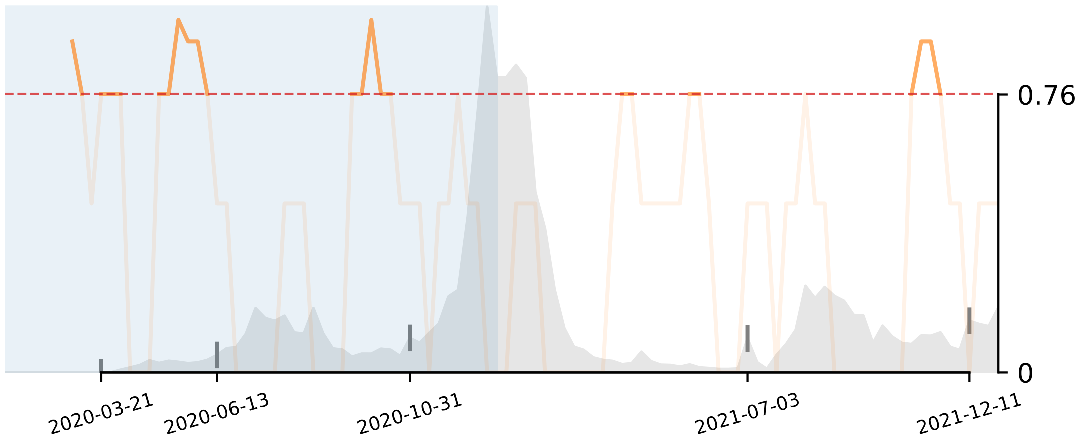

β	Training tp	Training fp	Training fn	Out of sample tp	Out of sample fp	Out of sample fn
1.00	0	0	3	0	0	2
0.99	0	0	3	0	0	2
0.96	0	0	3	0	0	2
0.91	2	0	1	1	0	1
0.76	3	0	0	2	3	0
0.46	3	7	0	2	21	0
0.00	3	16	0	2	38	0

CA, Sacramento

Proxies selected by the Multiple Sources method

Model 1

(GT) covid
(GT) how long does covid last
(GT) covid-19
(GT) chest pain
(GT) covid symptoms
(GT) quarantine

Model 2

(GT) fever
(GT) Burning Chest Pain
(GT) Otitis
Apple Mobility (transit)
(GT) Nasal congestion
(GT) Wheeze

Model 3

(GT) Burning Chest Pain
Apple Mobility (transit)
(GT) covid
(GT) covid symptoms
(GT) quarantine
(GT) fever

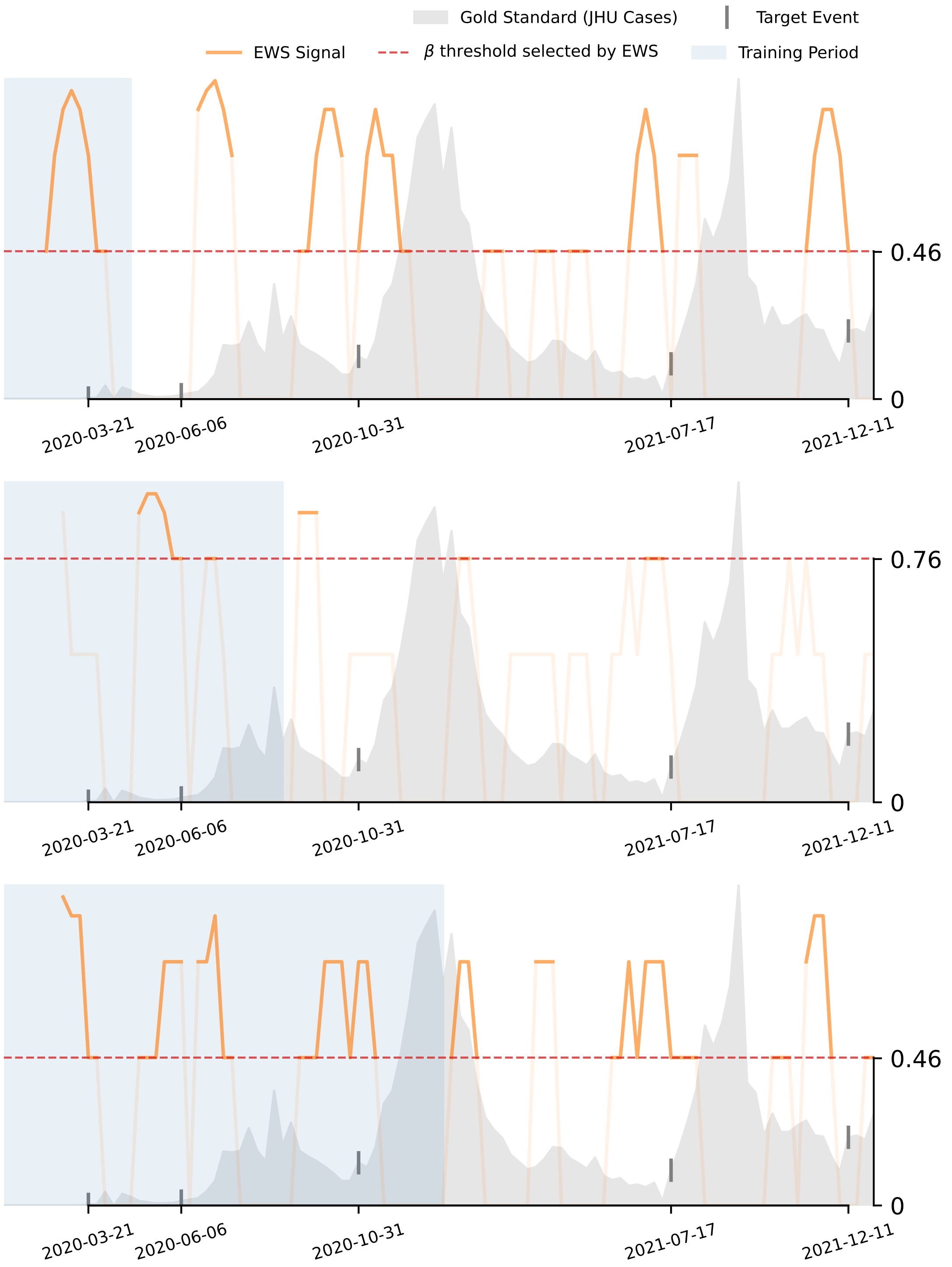

Performance as a function of β

Model 1

β	Training tp	Training fp	Training fn	Out of sample tp	Out of sample fp	Out of sample fn
1.00	0	0	1	0	1	4
0.99	0	0	1	0	1	4
0.96	0	0	1	0	2	4
0.91	1	0	0	3	5	0
0.76	1	0	0	3	12	0
0.46	1	0	0	3	24	0
0.00	1	3	0	4	61	0

Model 2

β	Training tp	Training fp	Training fn	Out of sample tp	Out of sample fp	Out of sample fn
1.00	0	0	2	0	0	3
0.99	0	0	2	0	0	3
0.96	0	0	2	0	0	3
0.91	1	0	1	1	1	2
0.76	2	1	0	3	4	0
0.46	2	3	0	3	24	0
0.00	2	6	0	3	49	0

Model 3

β	Training tp	Training fp	Training fn	Out of sample tp	Out of sample fp	Out of sample fn
1.00	0	0	3	0	0	2
0.99	0	0	3	0	0	2
0.96	0	0	3	0	0	2
0.91	1	0	2	1	0	1
0.76	1	1	2	2	5	0
0.46	3	4	0	2	16	0
0.00	3	12	0	2	37	0

CA, San Bernardino

Proxies selected by the Multiple Sources method

Model 1

(GT) chest tightness
(GT) covid
(GT) how long does covid last
(GT) covid-19
(GT) loss taste
(GT) chest pain

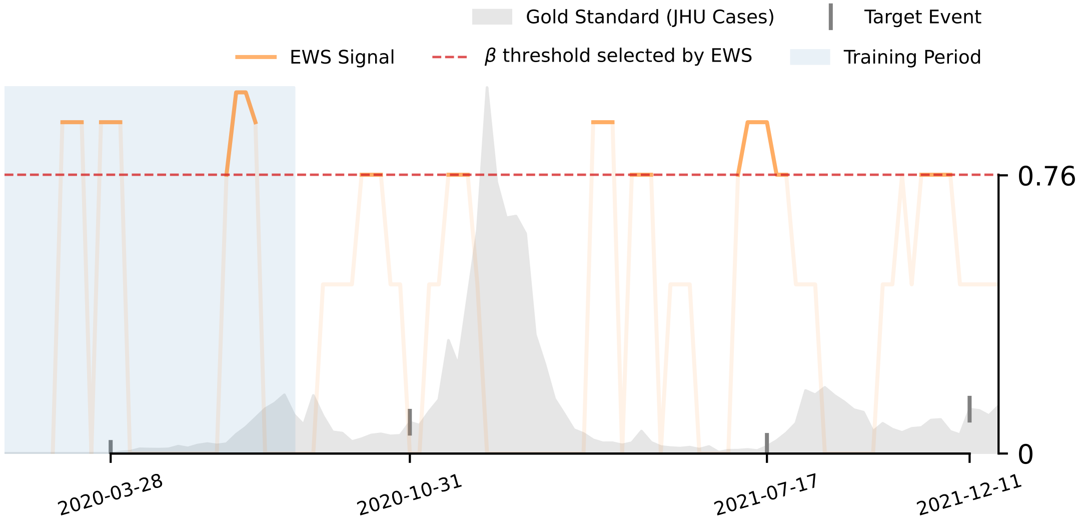

Performance as a function of β

	Training			Out of sample		
β	tp	fp	fn	tp	fp	fn
1.00	0	0	1	0	0	3
0.99	0	0	1	0	0	3
0.96	0	2	1	0	0	3
0.91	0	2	1	1	3	2
0.76	1	5	0	3	12	0
0.46	1	6	0	3	29	0
0.00	1	6	0	3	52	0

Model 2

(GT) covid
(GT) how long does covid last
(GT) covid symptoms
(GT) Ageusia
(GT) Anosmia
(GT) Bronchitis

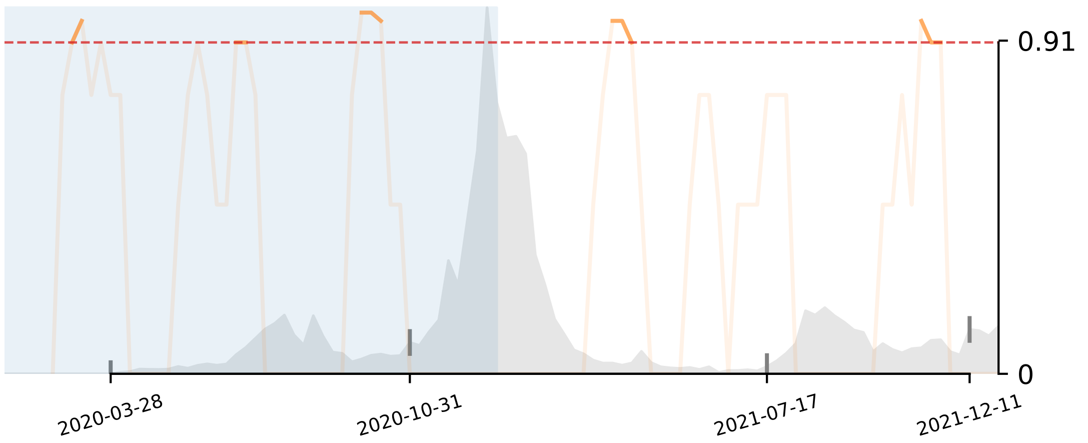

	Training			Out of sample		
β	tp	fp	fn	tp	fp	fn
1.00	0	0	2	0	0	2
0.99	0	0	2	0	0	2
0.96	1	1	1	1	2	1
0.91	2	1	0	1	3	1
0.76	2	4	0	2	8	0
0.46	2	10	0	2	13	0
0.00	2	13	0	2	38	0

Model 3

(GT) how long does covid last
(GT) covid
(GT) sars-cov-2
(GT) Fever
(GT) covid-19
(GT) Anosmia

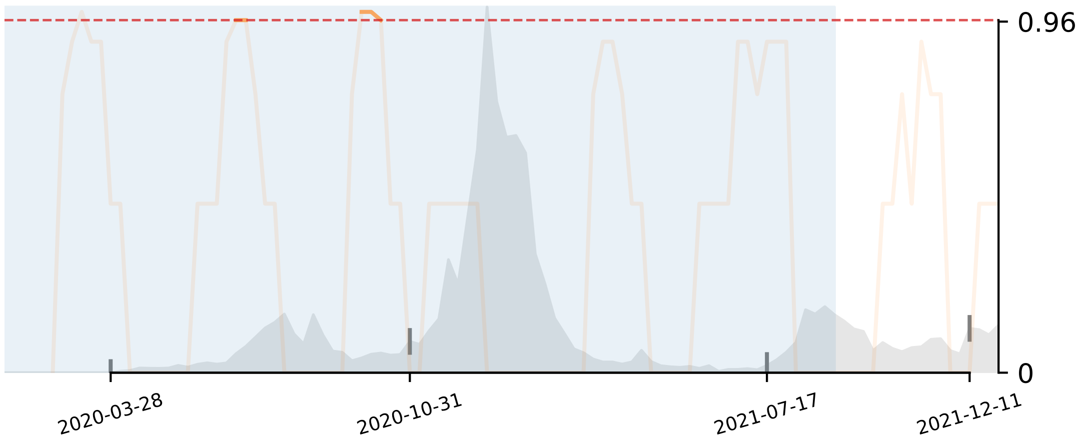

	Training			Out of sample		
β	tp	fp	fn	tp	fp	fn
1.00	0	0	3	0	0	1
0.99	0	0	3	0	0	1
0.96	2	1	1	0	0	1
0.91	2	3	1	1	0	0
0.76	3	8	0	1	1	0
0.46	3	13	0	1	6	0
0.00	3	30	0	1	10	0

Proxies selected by the Multiple Sources method

Model 1

(GT) covid
(GT) covid-19
(GT) covid symptoms
(GT) Acute bronchitis
(GT) Ageusia
(GT) Anosmia

Model 2

(GT) covid
(GT) covid-19
(GT) covid symptoms
(GT) sars-cov-2
(GT) Acute bronchitis
(GT) Ageusia

Model 3

(GT) covid-19
(GT) covid
(GT) covid symptoms
(GT) Fever
(GT) Wheeze
(GT) Asphyxia

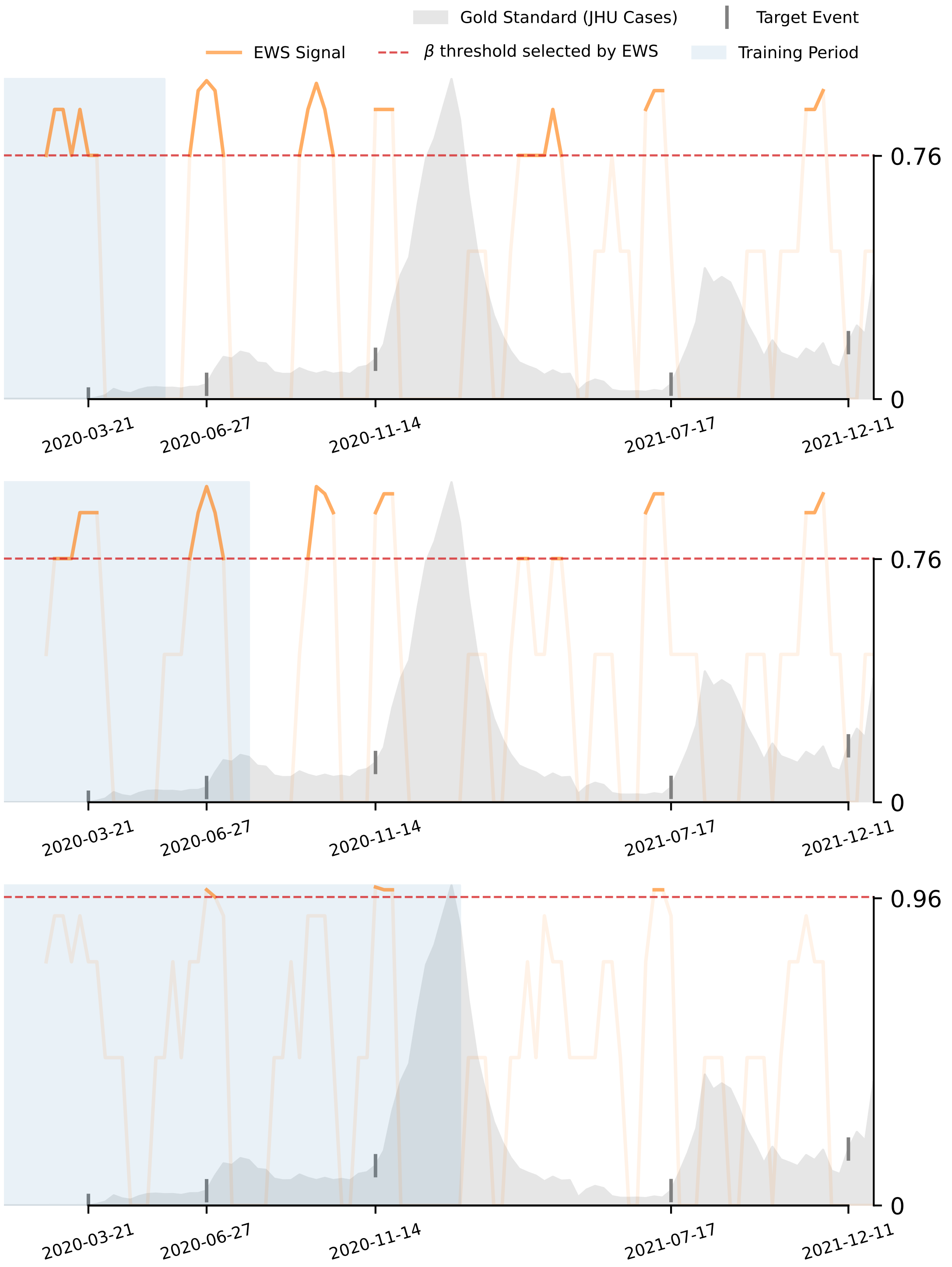

Performance as a function of β

Model 1

β	Training tp	Training fp	Training fn	Out of sample tp	Out of sample fp	Out of sample fn
1.00	0	0	1	1	0	3
0.99	0	0	1	1	1	3
0.96	0	0	1	3	2	1
0.91	0	0	1	4	6	0
0.76	1	0	0	4	14	0
0.46	1	2	0	4	28	0
0.00	1	2	0	4	58	0

Model 2

β	Training tp	Training fp	Training fn	Out of sample tp	Out of sample fp	Out of sample fn
1.00	0	0	2	0	0	3
0.99	0	0	2	0	1	3
0.96	1	0	1	3	3	0
0.91	1	0	1	3	3	0
0.76	2	2	0	3	8	0
0.46	2	3	0	3	30	0
0.00	2	6	0	3	53	0

Model 3

β	Training tp	Training fp	Training fn	Out of sample tp	Out of sample fp	Out of sample fn
1.00	0	0	3	0	0	2
0.99	1	0	2	1	0	1
0.96	2	2	1	1	0	1
0.91	2	3	1	2	1	0
0.76	3	7	0	2	7	0
0.46	3	10	0	2	24	0
0.00	3	19	0	2	35	0

Proxies selected by the Multiple Sources method

Performance as a function of β

Model 1

(GT) covid
(GT) covid-19
(GT) chest pain
(GT) covid symptoms
(GT) chest tightness
(GT) quarantine

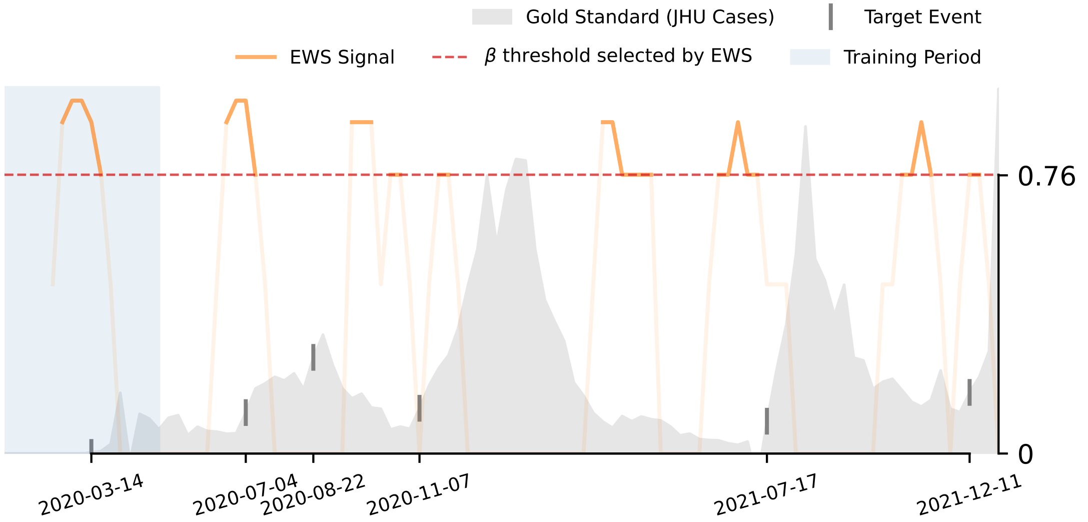

β	Training tp	Training fp	Training fn	Out of sample tp	Out of sample fp	Out of sample fn
1.00	0	0	1	0	0	5
0.99	0	0	1	0	0	5
0.96	0	0	1	1	0	4
0.91	1	0	0	4	3	1
0.76	1	0	0	5	11	0
0.46	1	1	0	5	19	0
0.00	1	2	0	5	52	0

Model 2

(GT) covid
(GT) covid-19
(GT) chest pain
(GT) covid symptoms
(GT) quarantine
(GT) fever

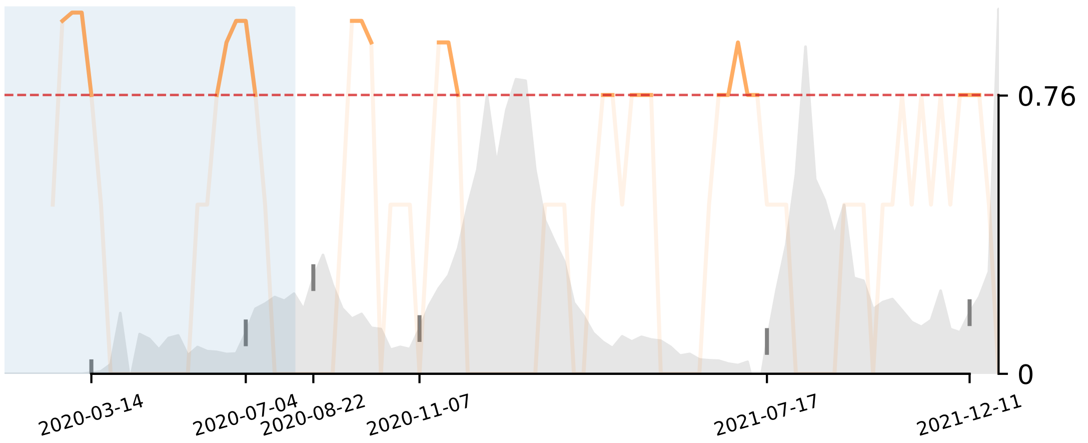

β	Training tp	Training fp	Training fn	Out of sample tp	Out of sample fp	Out of sample fn
1.00	0	0	2	0	0	4
0.99	0	0	2	0	0	4
0.96	1	0	1	1	1	3
0.91	2	0	0	2	3	2
0.76	2	0	0	3	11	1
0.46	2	1	0	3	26	1
0.00	2	4	0	4	50	0

Model 3

(GT) Asthma
(GT) covid
(GT) covid-19
(GT) chest pain
(GT) covid symptoms
(GT) quarantine

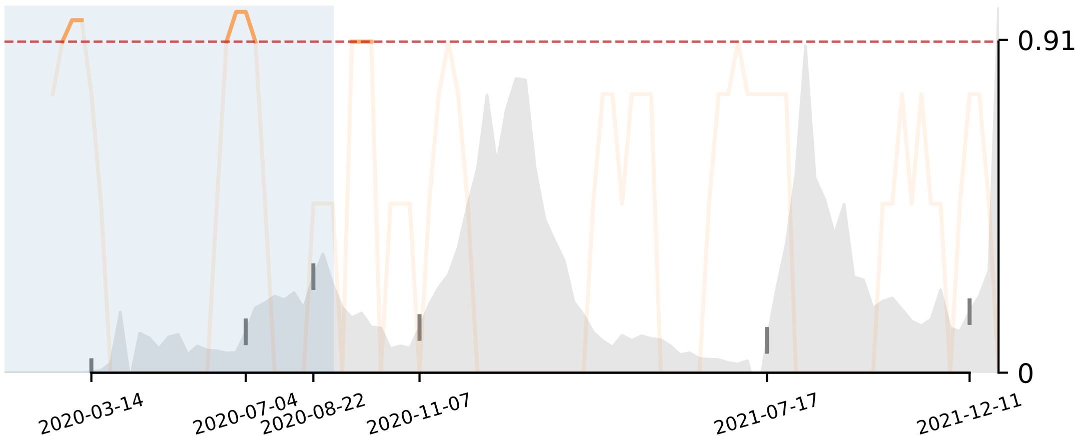

β	Training tp	Training fp	Training fn	Out of sample tp	Out of sample fp	Out of sample fn
1.00	0	0	3	0	0	3
0.99	0	0	3	0	0	3
0.96	1	0	2	0	0	3
0.91	2	0	1	2	2	1
0.76	2	1	1	3	13	0
0.46	2	1	1	3	20	0
0.00	3	5	0	3	48	0

CA, Santa Clara

Proxies selected by the Multiple Sources method

Model 1

(GT) covid
(GT) covid-19
(GT) chest pain
(GT) covid symptoms
(GT) chest tightness
(GT) quarantine

Model 2

(GT) covid
(GT) covid-19
(GT) chest pain
(GT) covid symptoms
(GT) fever
(GT) Acute bronchitis

Model 3

(GT) chest pain
(GT) covid symptoms
(GT) Anosmia
(GT) Fever
(GT) Otitis
up2date

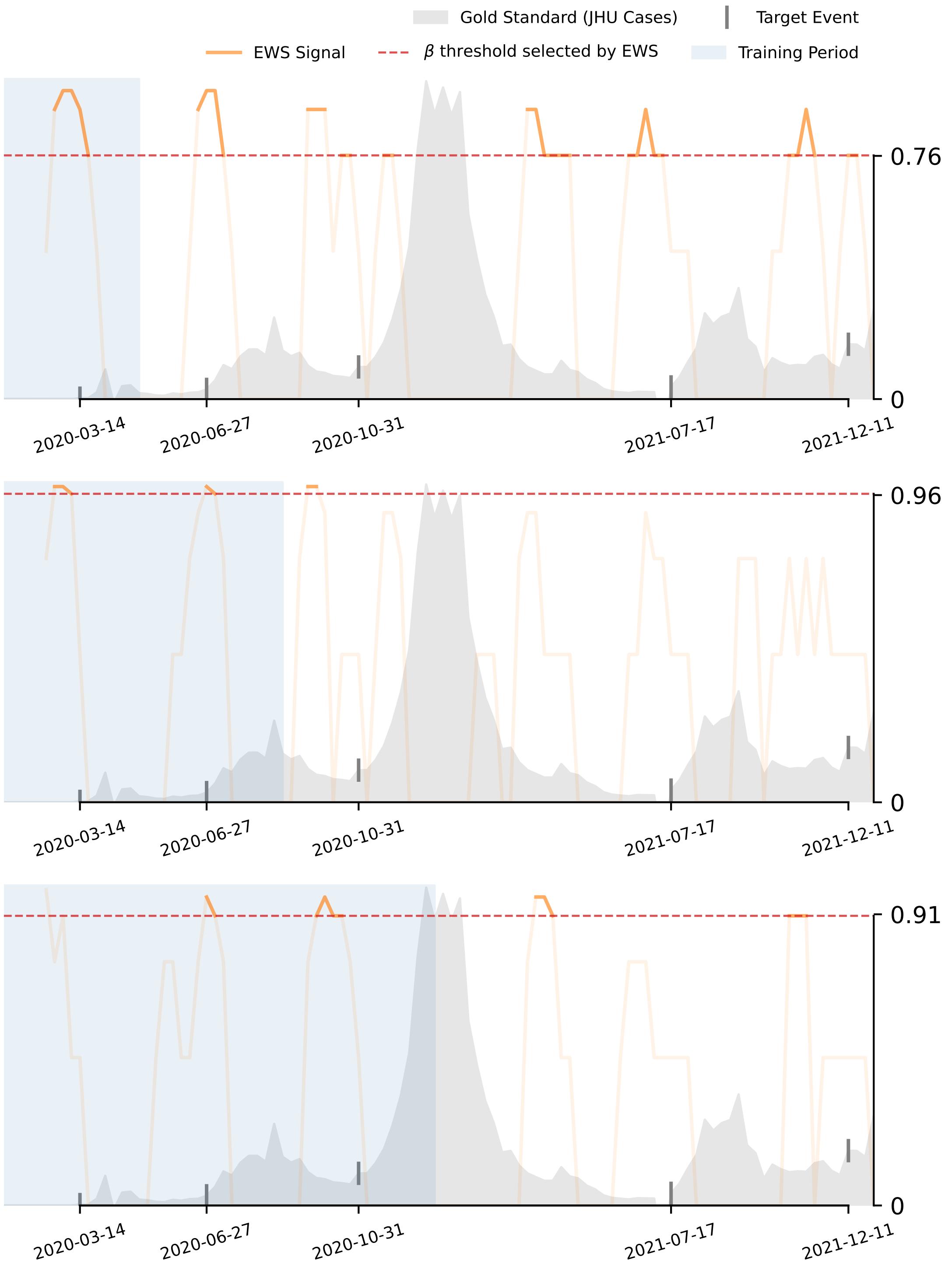

Performance as a function of β

Model 1

β	Training tp	Training fp	Training fn	Out of sample tp	Out of sample fp	Out of sample fn
1.00	0	0	1	0	0	4
0.99	0	0	1	0	0	4
0.96	0	0	1	1	1	3
0.91	1	0	0	4	3	0
0.76	1	0	0	4	12	0
0.46	1	1	0	4	21	0
0.00	1	2	0	4	59	0

Model 2

β	Training tp	Training fp	Training fn	Out of sample tp	Out of sample fp	Out of sample fn
1.00	0	0	2	0	0	3
0.99	0	0	2	1	0	2
0.96	2	0	0	1	0	2
0.91	2	1	0	2	4	1
0.76	2	1	0	3	11	0
0.46	2	2	0	3	25	0
0.00	2	2	0	3	49	0

Model 3

β	Training tp	Training fp	Training fn	Out of sample tp	Out of sample fp	Out of sample fn
1.00	0	0	3	0	0	2
0.99	0	0	3	0	0	2
0.96	1	0	2	0	2	2
0.91	3	0	0	1	4	1
0.76	3	2	0	2	5	0
0.46	3	5	0	2	11	0
0.00	3	6	0	2	38	0

Proxies selected by the Multiple Sources method

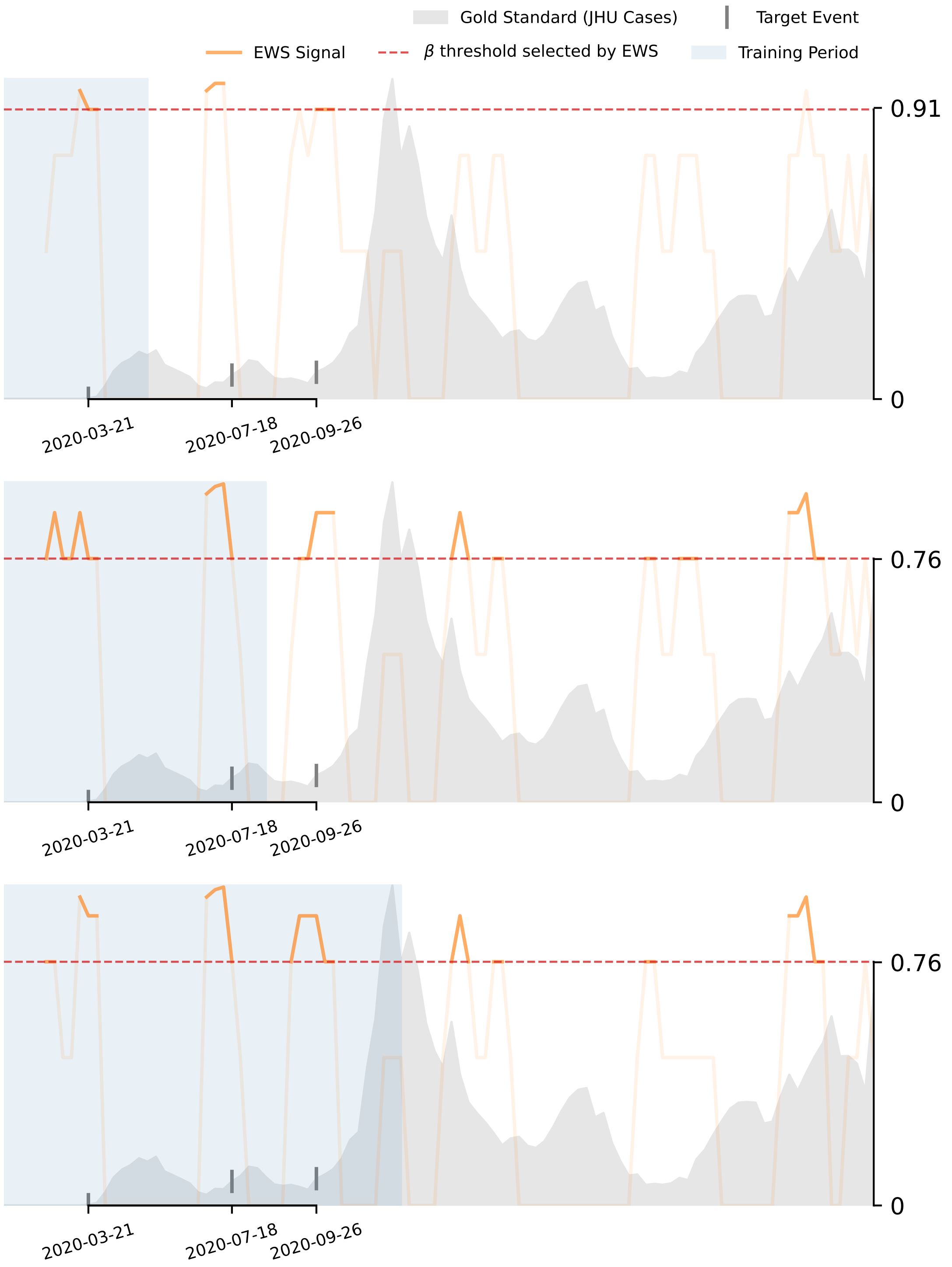

Performance as a function of β

Model 1

(GT) covid
(GT) how long does covid last
(GT) covid-19
(GT) covid symptoms
(GT) quarantine
(GT) sars-cov-2

	Training			Out of sample		
β	tp	fp	fn	tp	fp	fn
1.00	0	0	1	0	0	2
0.99	0	0	1	1	0	1
0.96	0	0	1	1	1	1
0.91	1	0	0	2	3	0
0.76	1	1	0	2	18	0
0.46	1	1	0	2	38	0
0.00	1	2	0	2	72	0

Model 2

(GT) covid
(GT) how long does covid last
(GT) covid-19
(GT) covid symptoms
(GT) quarantine
(GT) Acute bronchitis

β	tp	fp	fn	tp	fp	fn
1.00	0	0	2	0	0	1
0.99	1	0	1	0	0	1
0.96	1	0	1	0	1	1
0.91	1	0	1	1	6	0
0.76	2	0	0	1	19	0
0.46	2	2	0	1	37	0
0.00	2	3	0	1	66	0

Model 3

(GT) covid
(GT) how long does covid last
(GT) covid symptoms
(GT) Bronchitis
(GT) quarantine
(GT) Acute bronchitis

β	tp	fp	fn	tp	fp	fn
1.00	0	0	3	0	0	0
0.99	1	0	2	0	0	0
0.96	1	0	2	0	1	0
0.91	2	0	1	0	4	0
0.76	3	1	0	0	13	0
0.46	3	4	0	0	29	0
0.00	3	8	0	0	56	0

Proxies selected by the Multiple Sources method

Model 1

(GT) covid
(GT) how long does covid last
(GT) covid-19
(GT) covid symptoms
(GT) quarantine
(GT) sars-cov-2

Model 2

(GT) covid
(GT) how long does covid last
(GT) covid-19
(GT) covid symptoms
(GT) quarantine
(GT) Acute bronchitis

Model 3

(GT) covid
(GT) how long does covid last
(GT) covid symptoms
(GT) Acute bronchitis
(GT) Ageusia
(GT) Anosmia

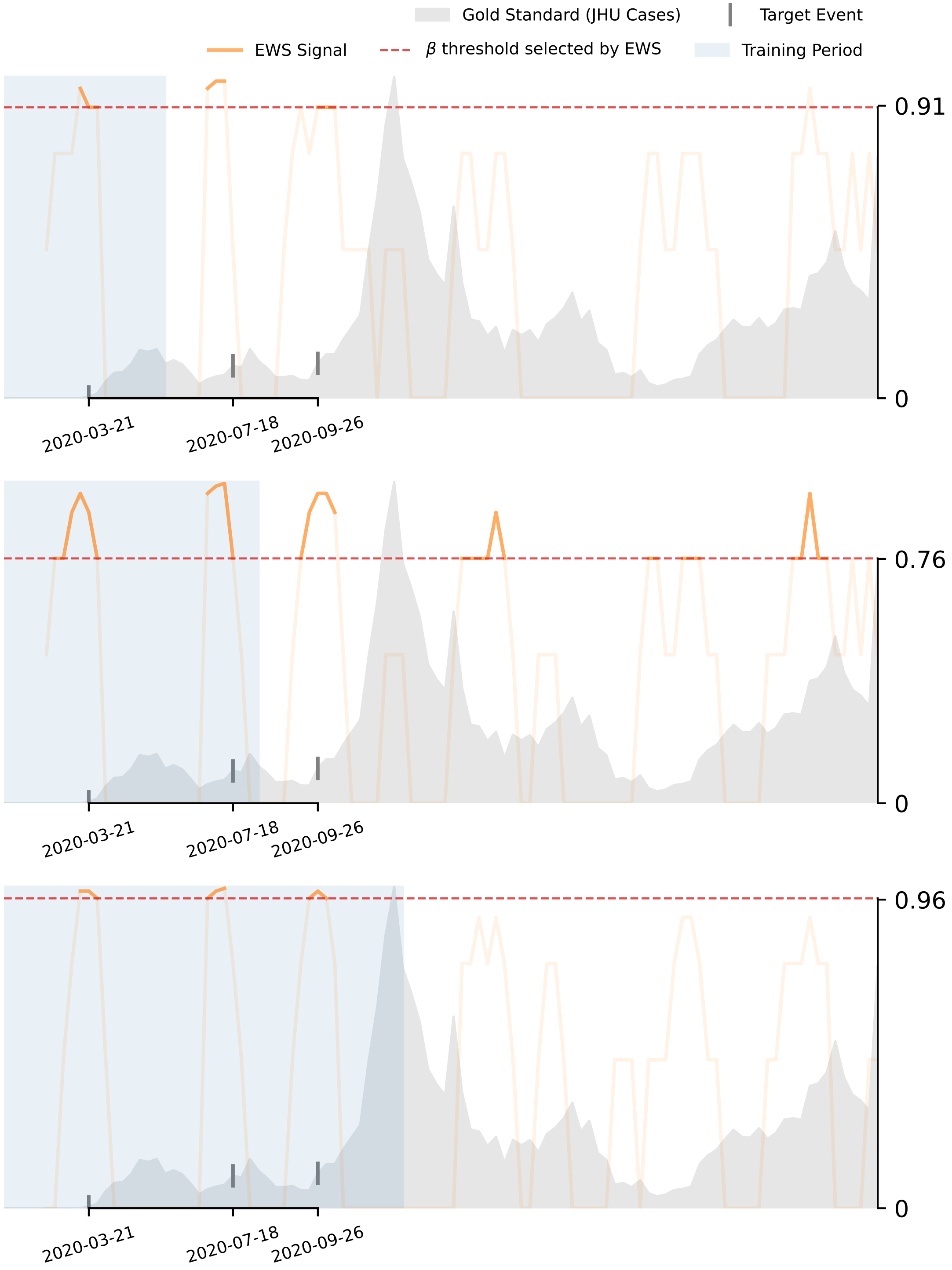

Performance as a function of β

Model 1

β	Training tp	Training fp	Training fn	Out of sample tp	Out of sample fp	Out of sample fn
1.00	0	0	1	0	0	2
0.99	0	0	1	1	0	1
0.96	0	0	1	1	1	1
0.91	1	0	0	2	3	0
0.76	1	1	0	2	18	0
0.46	1	1	0	2	38	0
0.00	1	2	0	2	70	0

Model 2

β	Training tp	Training fp	Training fn	Out of sample tp	Out of sample fp	Out of sample fn
1.00	0	0	2	0	0	1
0.99	1	0	1	0	0	1
0.96	1	0	1	1	2	0
0.91	2	0	0	1	4	0
0.76	2	0	0	1	20	0
0.46	2	1	0	1	41	0
0.00	2	3	0	1	66	0

Model 3

β	Training tp	Training fp	Training fn	Out of sample tp	Out of sample fp	Out of sample fn
1.00	0	0	3	0	0	0
0.99	1	0	2	0	0	0
0.96	3	0	0	0	0	0
0.91	3	2	0	0	5	0
0.76	3	2	0	0	18	0
0.46	3	3	0	0	33	0
0.00	3	5	0	0	56	0

Proxies selected by the Multiple Sources method

Performance as a function of β

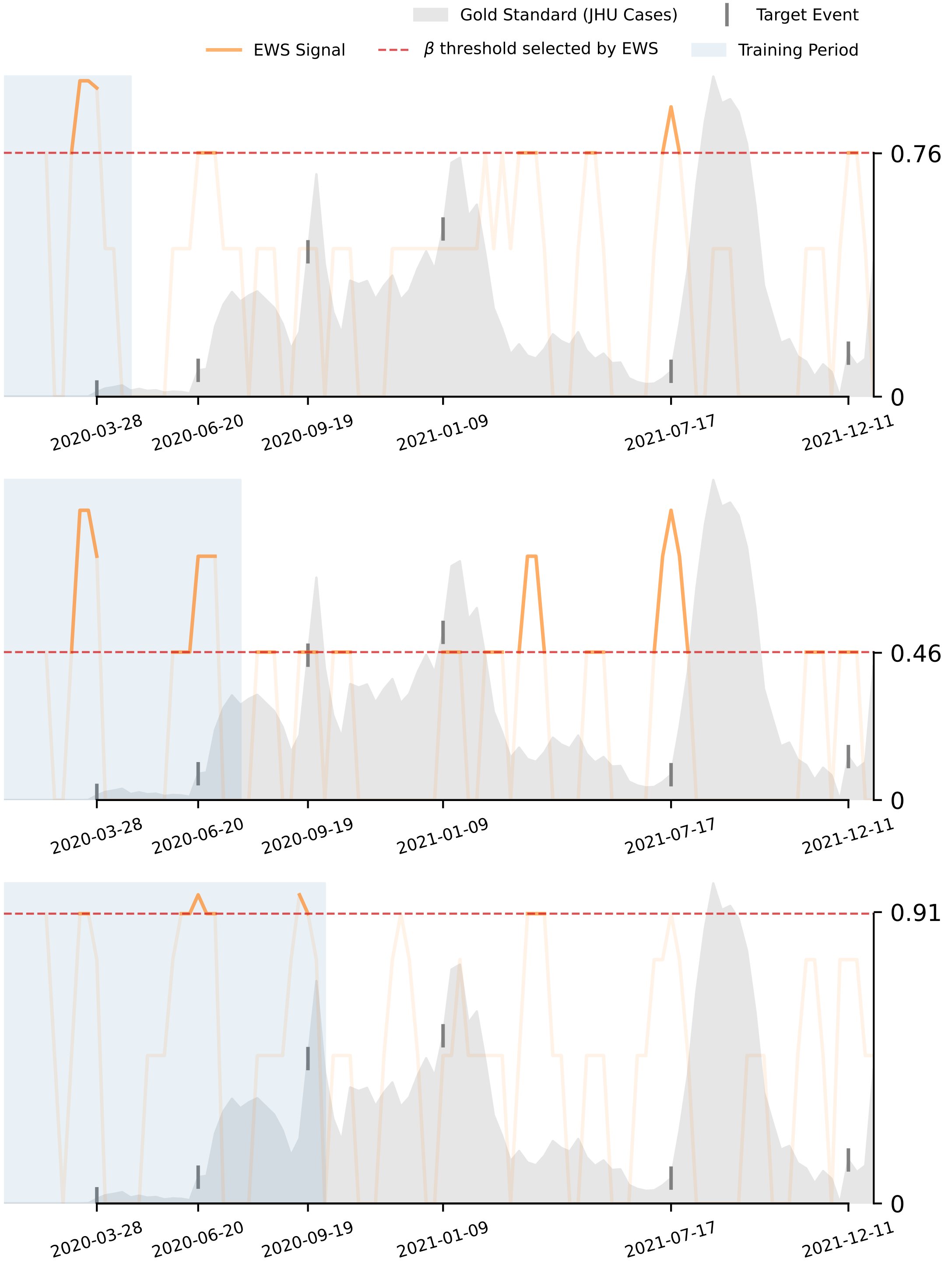

Model 1

(GT) covid
(GT) covid-19
(GT) quarantine
(GT) Wheeze
(GT) Anxiety
(GT) Cough

β	Training tp	Training fp	Training fn	Out of sample tp	Out of sample fp	Out of sample fn
1.00	0	0	1	0	0	5
0.99	0	0	1	0	0	5
0.96	1	0	0	0	0	5
0.91	1	0	0	1	0	4
0.76	1	0	0	3	11	2
0.46	1	1	0	5	32	0
0.00	1	3	0	5	53	0

Model 2

(GT) covid
(GT) covid-19
(GT) Cough

β	Training tp	Training fp	Training fn	Out of sample tp	Out of sample fp	Out of sample fn
0.91	0	0	2	1	0	3
0.76	1	0	1	1	3	3
0.46	2	2	0	4	19	0
0.00	2	3	0	4	47	0

Model 3

(GT) covid-19
(GT) Cough
(GT) covid
(GT) covid symptoms
(GT) Chest pain
(GT) Fever

β	Training tp	Training fp	Training fn	Out of sample tp	Out of sample fp	Out of sample fn
1.00	0	0	3	0	0	3
0.99	0	0	3	0	0	3
0.96	0	0	3	0	0	3
0.91	2	0	1	2	3	1
0.76	3	3	0	3	6	0
0.46	3	4	0	3	28	0
0.00	3	9	0	3	44	0

Proxies selected by the Multiple Sources method

Model 1

(GT) covid
(GT) how long does covid last
(GT) covid-19
(GT) chest pain
(GT) covid symptoms
(GT) chest tightness

Model 2

(GT) covid symptoms
(GT) covid-19 who
(GT) Ageusia
(GT) Diarrhea
(GT) Otitis

Model 3

(GT) covid symptoms
(GT) Ageusia
(GT) covid-19 who
(GT) chest pain
(GT) covid
(GT) covid-19

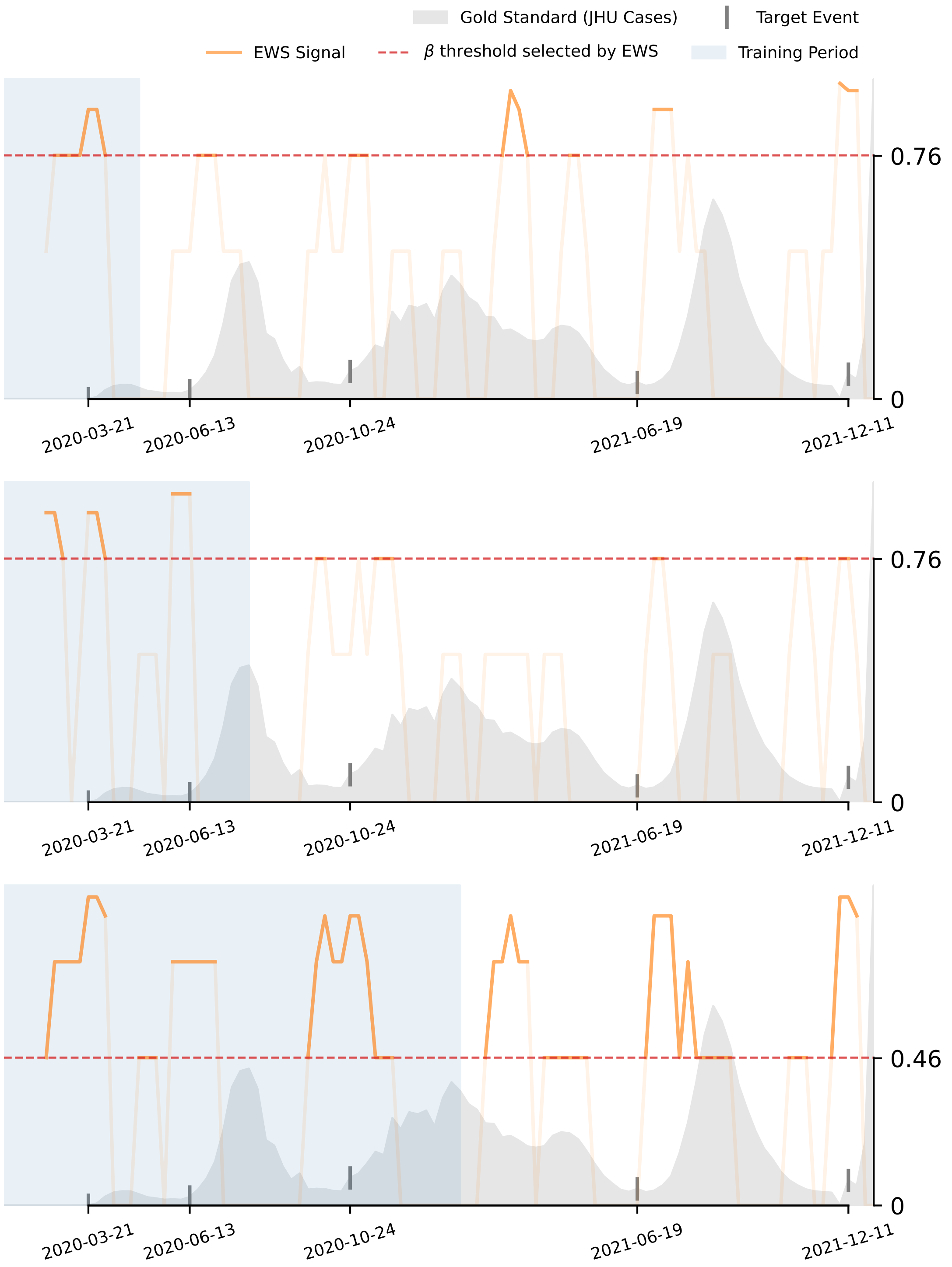

Performance as a function of β

Model 1

β	Training tp	Training fp	Training fn	Out of sample tp	Out of sample fp	Out of sample fn
1.00	0	0	1	0	0	4
0.99	0	0	1	1	0	3
0.96	0	0	1	1	2	3
0.91	0	0	1	1	6	2
0.76	1	1	0	2	16	0
0.46	1	2	0	4	32	0
0.00	1	3	0	4	60	0

Model 2

β	Training tp	Training fp	Training fn	Out of sample tp	Out of sample fp	Out of sample fn
0.99	0	0	2	0	0	3
0.96	0	0	2	0	0	3
0.91	1	0	1	0	0	3
0.76	2	2	0	2	6	0
0.46	2	3	0	2	27	0
0.00	2	5	0	3	53	0

Model 3

β	Training tp	Training fp	Training fn	Out of sample tp	Out of sample fp	Out of sample fn
1.00	0	0	3	0	0	2
0.99	0	0	3	0	0	2
0.96	0	0	3	1	0	1
0.91	1	1	2	1	5	0
0.76	2	3	1	1	10	0
0.46	3	7	0	2	24	0
0.00	3	14	0	2	35	0

Proxies selected by the Multiple Sources method

Model 1

(GT) covid
(GT) covid-19
(GT) covid symptoms
(GT) fever
(GT) Ageusia
(GT) Anosmia

Model 2

(GT) covid-19
(GT) covid symptoms
(GT) fever
(GT) Ageusia
(GT) Anosmia
(GT) Asphyxia

Model 3

(GT) covid-19
(GT) covid symptoms
(GT) fever
(GT) Asphyxia
(GT) nose bleed
(GT) loss taste

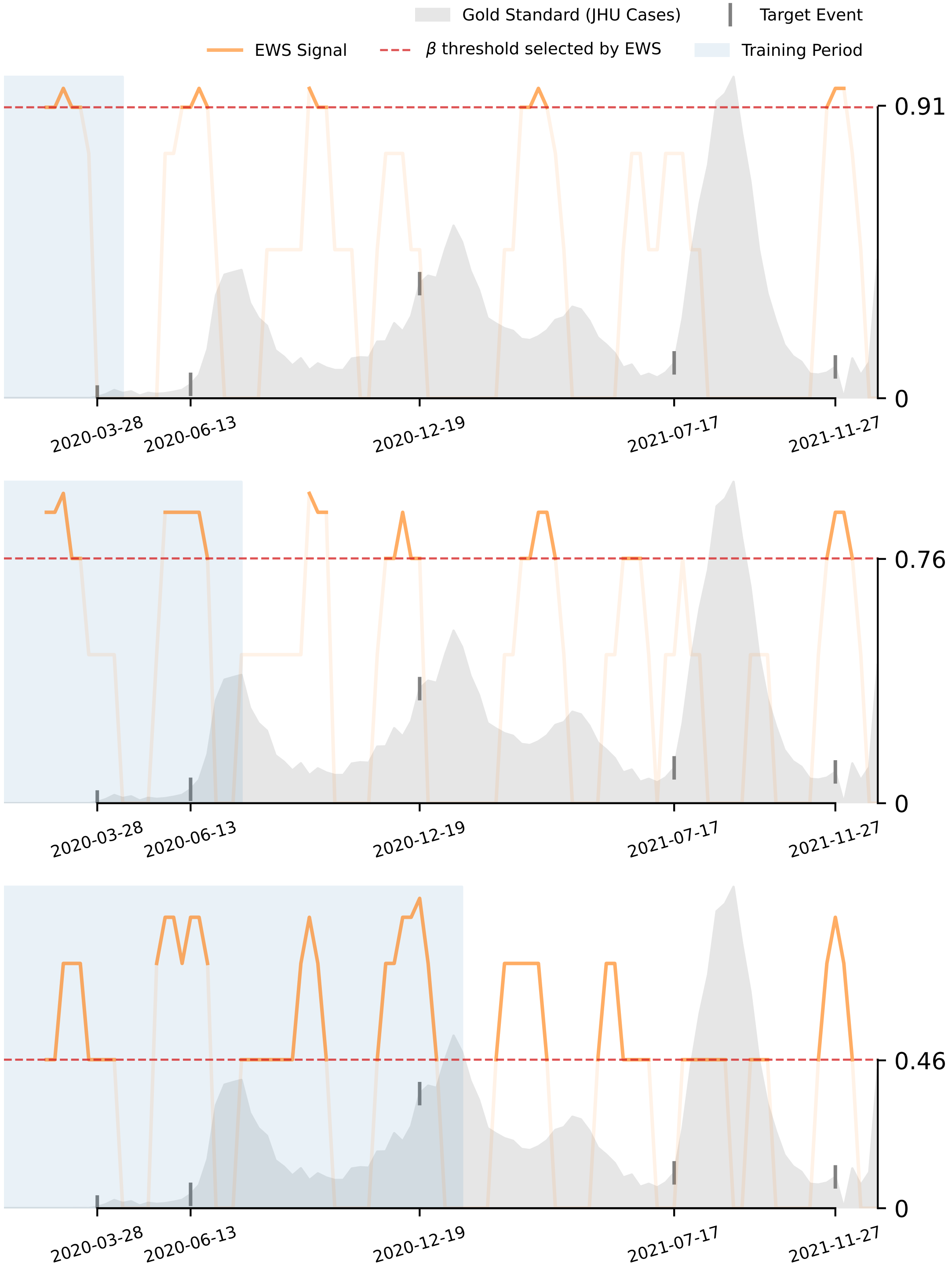

Performance as a function of β

Model 1

β	Training tp	Training fp	Training fn	Out of sample tp	Out of sample fp	Out of sample fn
1.00	0	0	1	0	0	4
0.99	0	0	1	0	0	4
0.96	0	0	1	1	4	2
0.91	1	0	0	2	10	2
0.76	1	2	0	4	13	0
0.46	1	2	0	4	28	0
0.00	1	2	0	4	61	0

Model 2

β	Training tp	Training fp	Training fn	Out of sample tp	Out of sample fp	Out of sample fn
1.00	0	0	2	0	0	3
0.99	0	0	2	0	0	3
0.96	0	0	2	0	1	3
0.91	1	0	1	2	6	1
0.76	2	3	0	3	11	0
0.46	2	4	0	3	29	0
0.00	2	7	0	3	54	0

Model 3

β	Training tp	Training fp	Training fn	Out of sample tp	Out of sample fp	Out of sample fn
1.00	0	0	3	0	0	2
0.99	0	0	3	0	0	2
0.96	0	0	3	0	0	2
0.91	1	0	2	1	0	1
0.76	2	2	1	1	8	1
0.46	3	6	0	2	21	0
0.00	3	20	0	2	35	0

Proxies selected by the Multiple Sources method

Model 1

(GT) covid
(GT) covid-19
(GT) chest pain
(GT) covid symptoms
(GT) quarantine
(GT) covid-19 who

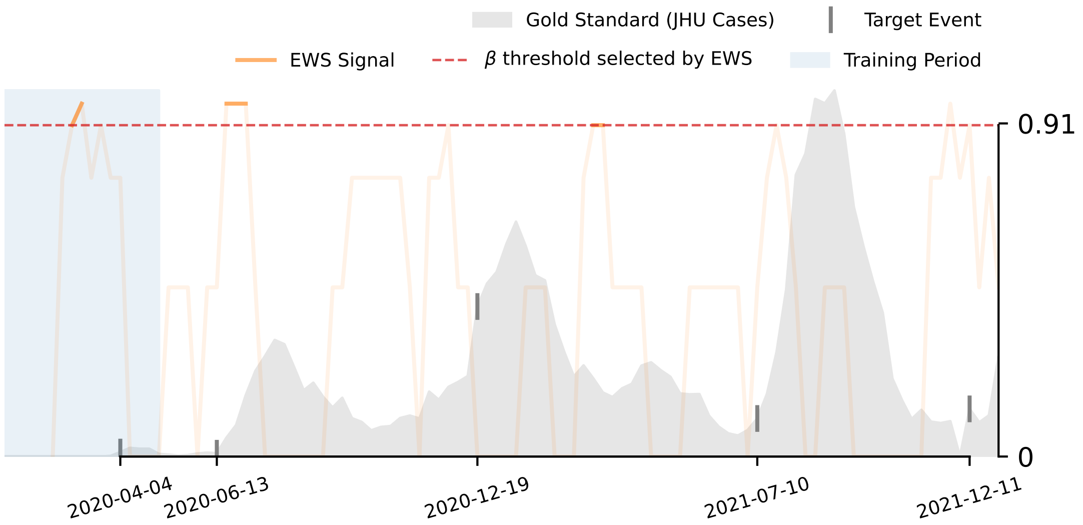

Performance as a function of β

β	Training tp	Training fp	Training fn	Out of sample tp	Out of sample fp	Out of sample fn
1.00	0	0	1	0	0	4
0.99	0	0	1	0	0	4
0.96	0	0	1	1	3	2
0.91	1	0	0	2	6	0
0.76	1	1	0	2	16	0
0.46	1	2	0	4	34	0
0.00	1	2	0	4	60	0

Model 2

(GT) chest pain
(GT) covid-19 who
(GT) Cardiac arrest

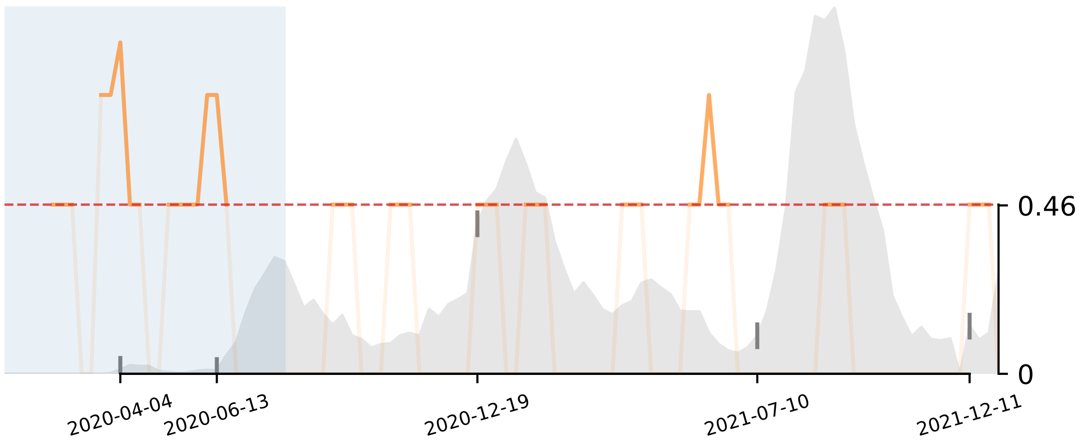

β	Training tp	Training fp	Training fn	Out of sample tp	Out of sample fp	Out of sample fn
0.91	0	0	2	0	0	3
0.76	1	0	1	1	0	2
0.46	2	0	0	3	20	0
0.00	2	7	0	3	53	0

Model 3

(GT) Cardiac arrest
(GT) how long does covid last
(GT) covid
(GT) covid-19 who
(GT) covid-19
(GT) chest pain

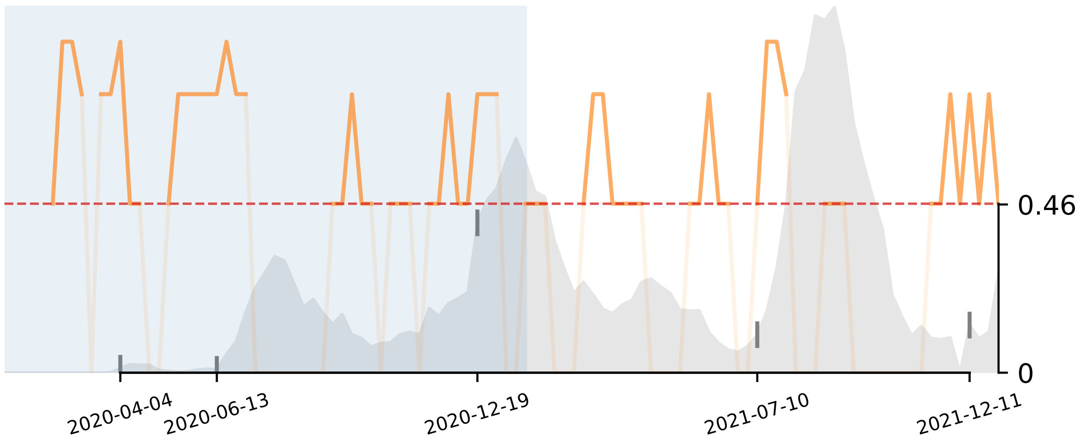

β	Training tp	Training fp	Training fn	Out of sample tp	Out of sample fp	Out of sample fn
1.00	0	0	3	0	0	2
0.99	0	0	3	0	0	2
0.96	0	0	3	0	0	2
0.91	0	0	3	0	2	1
0.76	1	3	2	2	6	0
0.46	3	8	0	2	19	0
0.00	3	21	0	2	35	0

Proxies selected by the Multiple Sources method

Model 1

(GT) covid
(GT) covid-19
(GT) chest pain
(GT) covid symptoms
(GT) quarantine
(GT) nose bleed

Model 2

(GT) covid
(GT) covid-19
(GT) chest pain
(GT) covid symptoms
(GT) covid-19 who
(GT) Dizziness

Model 3

(GT) covid
(GT) covid-19
(GT) chest pain
(GT) covid symptoms
(GT) Dizziness
(GT) covid-19 who

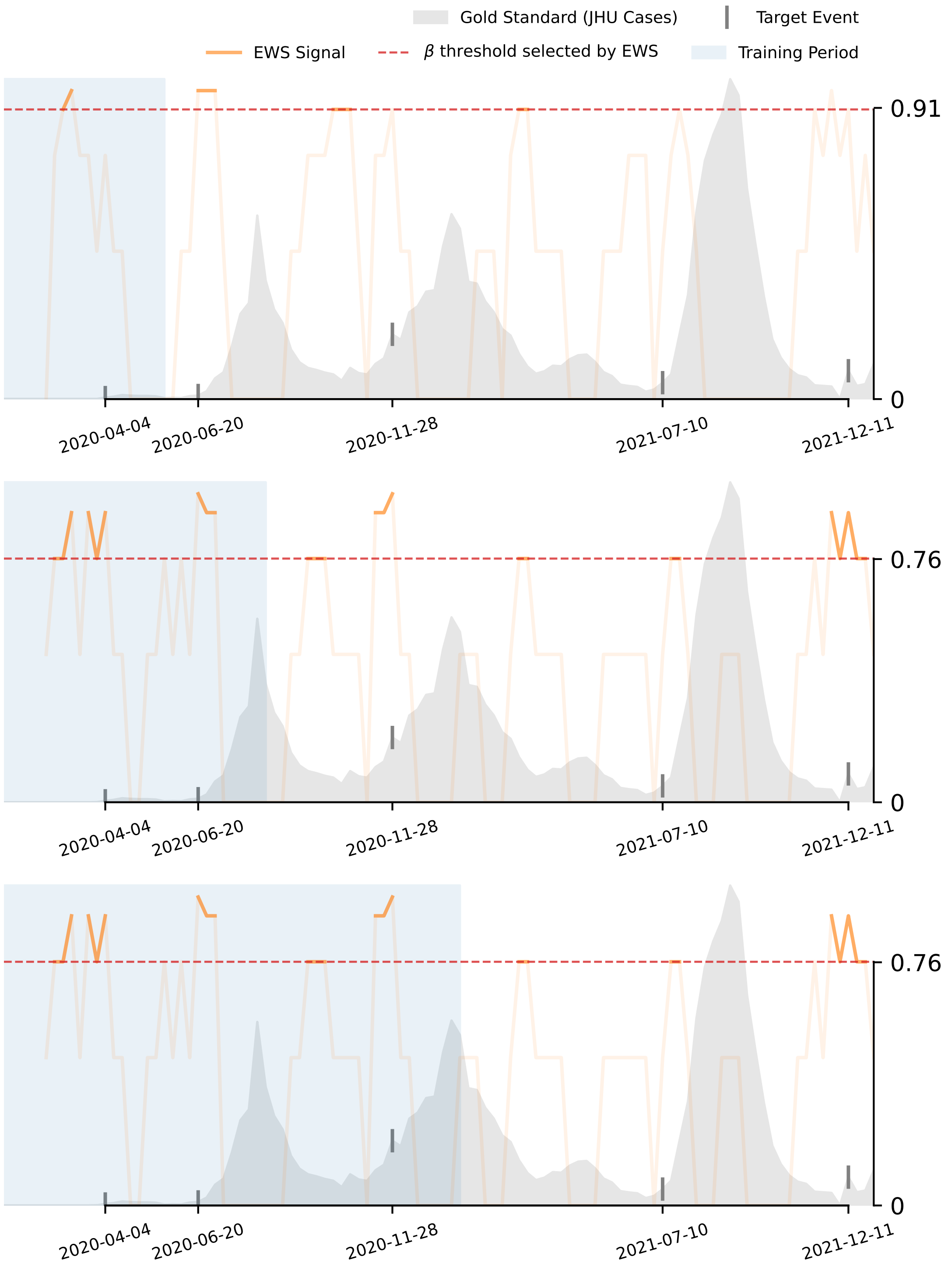

Performance as a function of β

Model 1

β	Training tp	Training fp	Training fn	Out of sample tp	Out of sample fp	Out of sample fn
1.00	0	0	1	0	0	4
0.99	0	0	1	0	0	4
0.96	0	0	1	2	2	2
0.91	1	0	0	3	6	0
0.76	1	1	0	4	13	0
0.46	1	2	0	4	29	0
0.00	1	4	0	4	59	0

Model 2

β	Training tp	Training fp	Training fn	Out of sample tp	Out of sample fp	Out of sample fn
1.00	0	0	2	0	0	3
0.99	0	0	2	0	0	3
0.96	0	0	2	1	0	2
0.91	1	0	1	2	0	1
0.76	2	2	0	2	9	0
0.46	2	4	0	3	28	0
0.00	2	9	0	3	51	0

Model 3

β	Training tp	Training fp	Training fn	Out of sample tp	Out of sample fp	Out of sample fn
1.00	0	0	3	0	0	2
0.99	0	0	3	0	0	2
0.96	0	0	3	0	0	2
0.91	2	0	1	1	0	1
0.76	3	2	0	1	6	0
0.46	3	7	0	2	19	0
0.00	3	20	0	2	35	0

FL, Miami-Dade

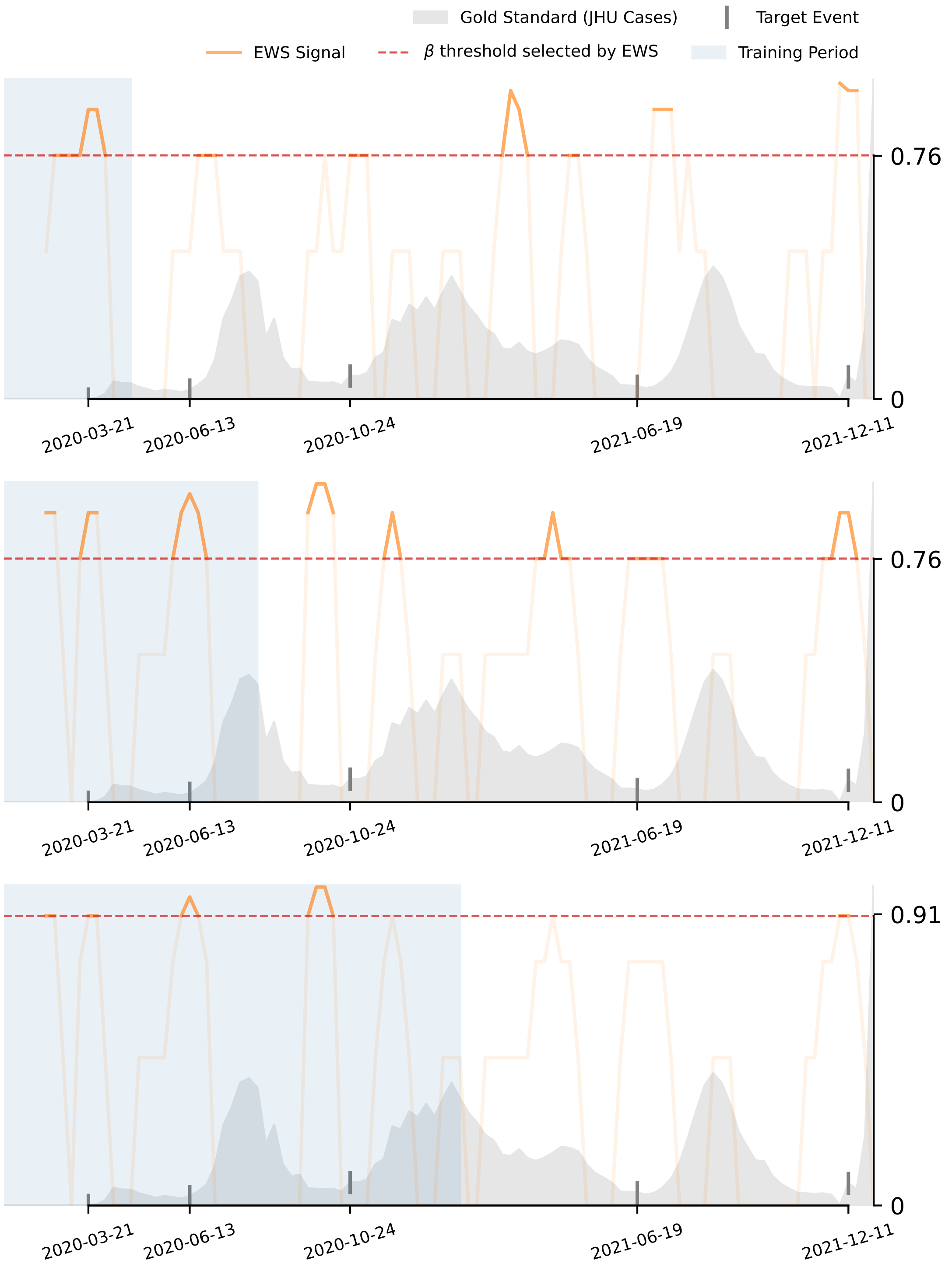

Proxies selected by the Multiple Sources method

Performance as a function of β

Model 1

(GT) covid
(GT) how long does covid last
(GT) covid-19
(GT) chest pain
(GT) covid symptoms
(GT) chest tightness

β	Training tp	Training fp	Training fn	Out of sample tp	Out of sample fp	Out of sample fn
1.00	0	0	1	0	0	4
0.99	0	0	1	1	0	3
0.96	0	0	1	1	2	3
0.91	0	0	1	1	6	2
0.76	1	1	0	2	16	0
0.46	1	2	0	4	32	0
0.00	1	3	0	4	60	0

Model 2

(GT) covid symptoms
(GT) covid-19 who
(GT) Ageusia
(GT) Anosmia
(GT) Chest pain
(GT) Fatigue

β	Training tp	Training fp	Training fn	Out of sample tp	Out of sample fp	Out of sample fn
1.00	0	0	2	1	0	2
0.99	0	0	2	1	0	2
0.96	0	0	2	1	0	2
0.91	1	0	1	2	2	1
0.76	2	3	0	3	12	0
0.46	2	4	0	3	29	0
0.00	2	7	0	3	52	0

Model 3

(GT) covid symptoms
(GT) Ageusia
(GT) Anosmia
(GT) covid-19 who
(GT) Chest pain
(GT) Fatigue

β	Training tp	Training fp	Training fn	Out of sample tp	Out of sample fp	Out of sample fn
1.00	0	0	3	0	0	2
0.99	1	0	2	0	0	2
0.96	1	0	2	0	0	2
0.91	2	0	1	1	1	1
0.76	3	5	0	2	9	0
0.46	3	8	0	2	21	0
0.00	3	16	0	2	35	0

Proxies selected by the Multiple Sources method

Model 1

(GT) covid
(GT) covid-19
(GT) chest pain
(GT) covid symptoms
(GT) quarantine
(GT) covid-19 who

Model 2

(GT) chest pain
(GT) covid-19 who
(GT) Ageusia
(GT) Anosmia
(GT) Hyperventilation

Model 3

(GT) chest pain
(GT) covid
(GT) covid-19 who
(GT) Ageusia
(GT) Anosmia
(GT) covid-19

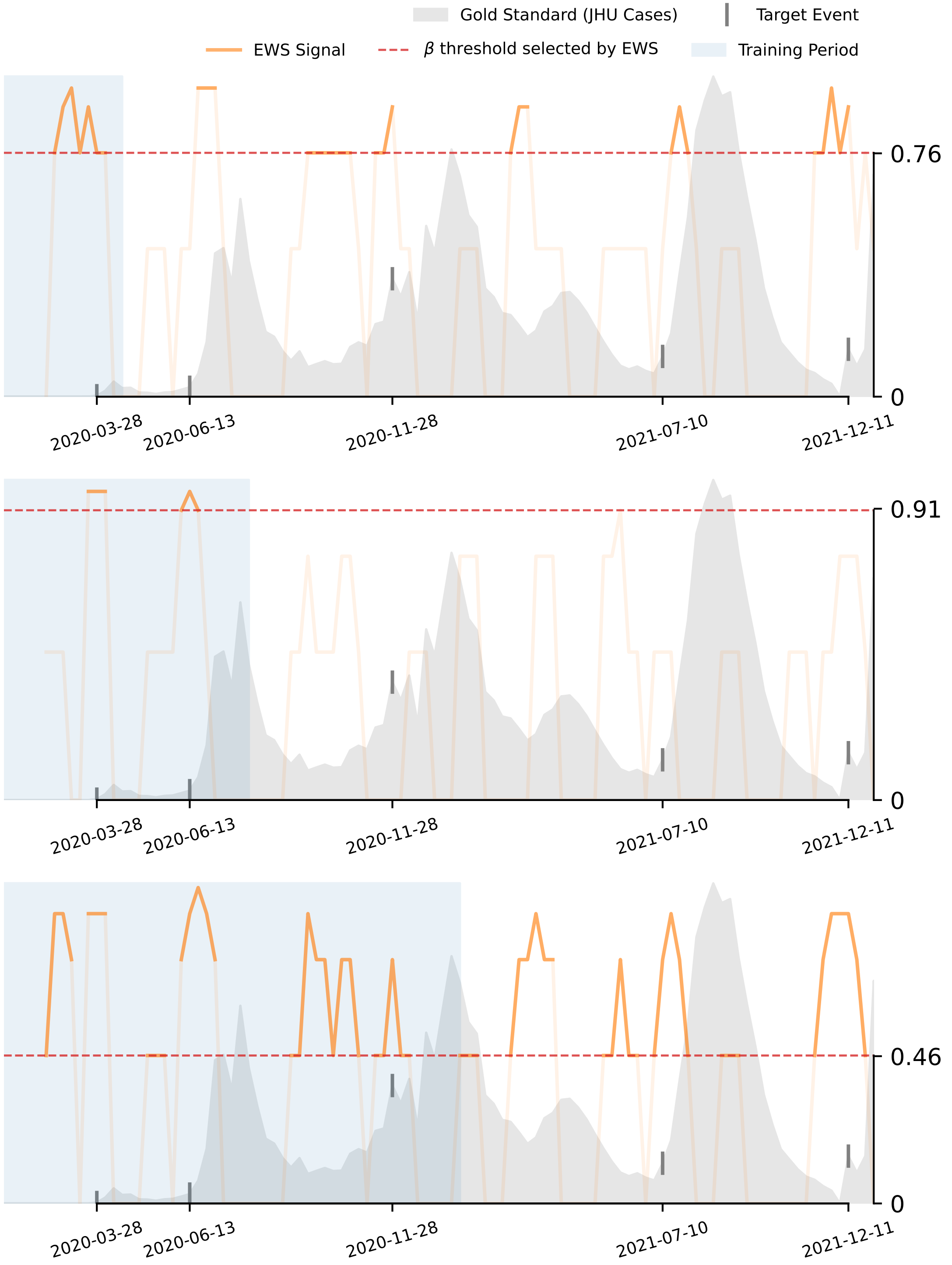

Performance as a function of β

Model 1

β	Training tp	Training fp	Training fn	Out of sample tp	Out of sample fp	Out of sample fn
1.00	0	0	1	0	0	4
0.99	0	0	1	0	0	4
0.96	0	0	1	1	3	2
0.91	1	0	0	2	6	0
0.76	1	0	0	2	14	0
0.46	1	2	0	4	33	0
0.00	1	2	0	4	61	0

Model 2

β	Training tp	Training fp	Training fn	Out of sample tp	Out of sample fp	Out of sample fn
0.99	0	0	2	0	0	3
0.96	0	0	2	0	0	3
0.91	2	1	0	1	0	2
0.76	2	2	0	3	9	0
0.46	2	2	0	3	23	0
0.00	2	6	0	3	53	0

Model 3

β	Training tp	Training fp	Training fn	Out of sample tp	Out of sample fp	Out of sample fn
1.00	0	0	3	0	0	2
0.99	0	0	3	0	0	2
0.96	0	1	3	0	0	2
0.91	0	1	3	1	2	0
0.76	2	5	1	2	8	0
0.46	3	10	0	2	17	0
0.00	3	18	0	2	35	0

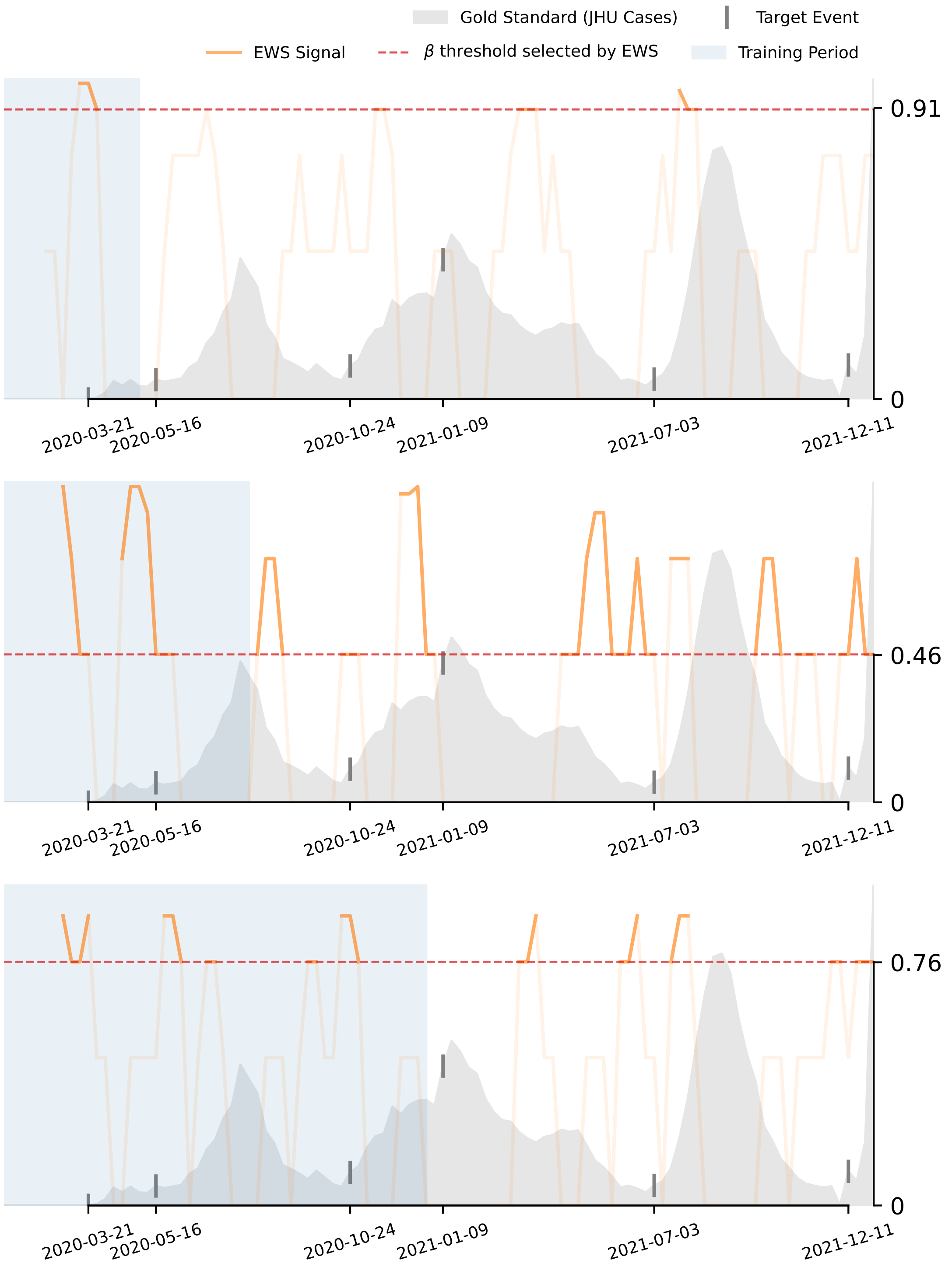

Proxies selected by the Multiple Sources method

Performance as a function of β

Model 1

(GT) covid
(GT) covid-19
(GT) chest pain
(GT) covid symptoms
(GT) chest tightness
(GT) quarantine

	Training			Out of sample		
β	tp	fp	fn	tp	fp	fn
1.00	0	0	1	0	0	5
0.99	0	0	1	0	0	5
0.96	1	0	0	0	1	5
0.91	1	0	0	0	9	5
0.76	1	1	0	3	19	0
0.46	1	1	0	4	36	0
0.00	1	2	0	5	57	0

Model 2

(GT) fever
(GT) Chronic pain
(GT) Fatigue
Apple Mobility (driving)
Apple Mobility (walking)
Apple Mobility (transit)

β	tp	fp	fn	tp	fp	fn
1.00	0	0	2	0	0	4
0.99	0	0	2	1	0	3
0.96	2	0	0	1	0	3
0.91	2	0	0	2	1	2
0.76	2	0	0	2	10	1
0.46	2	0	0	4	20	0
0.00	2	2	0	4	46	0

Model 3

Apple Mobility (walking)
(GT) covid-19
(GT) fever
(GT) Acute bronchitis
(GT) Ageusia
(GT) Anosmia

β	tp	fp	fn	tp	fp	fn
1.00	0	0	3	0	0	3
0.99	0	0	3	0	0	3
0.96	0	0	3	0	0	3
0.91	0	0	3	1	3	2
0.76	2	2	1	2	9	1
0.46	2	7	1	2	17	1
0.00	3	18	0	3	37	0

Proxies selected by the Multiple Sources method

Model 1

(GT) covid
(GT) covid-19
(GT) covid symptoms
(GT) fever
(GT) Acute bronchitis
(GT) Anosmia

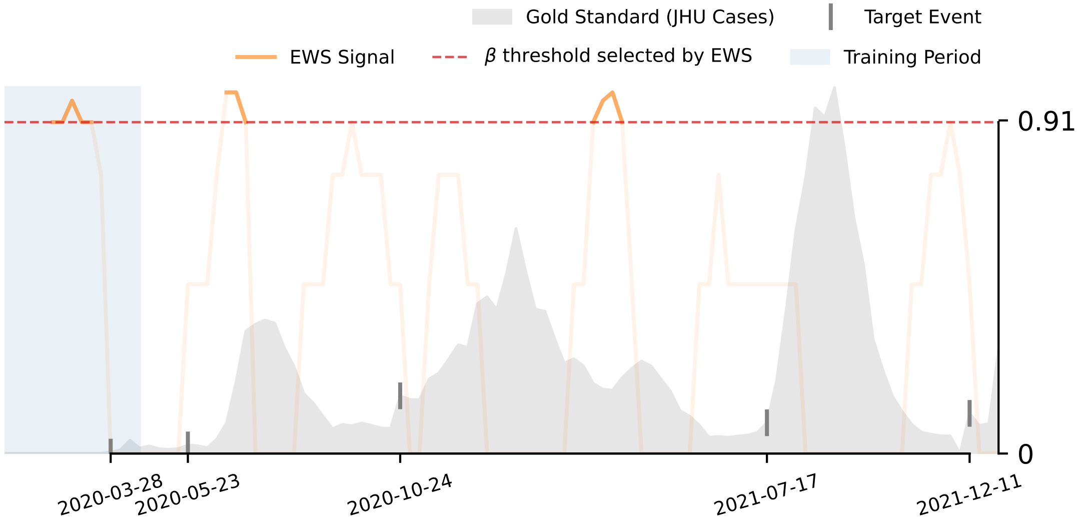

Performance as a function of β

β	Training tp	Training fp	Training fn	Out of sample tp	Out of sample fp	Out of sample fn
1.00	0	0	1	0	0	4
0.99	0	0	1	0	3	4
0.96	0	0	1	0	4	4
0.91	1	0	0	2	7	2
0.76	1	2	0	3	12	1
0.46	1	2	0	4	27	0
0.00	1	2	0	4	63	0

Model 2

(GT) fever
Apple Mobility (driving)
Apple Mobility (walking)
Apple Mobility (transit)
(GT) Otitis

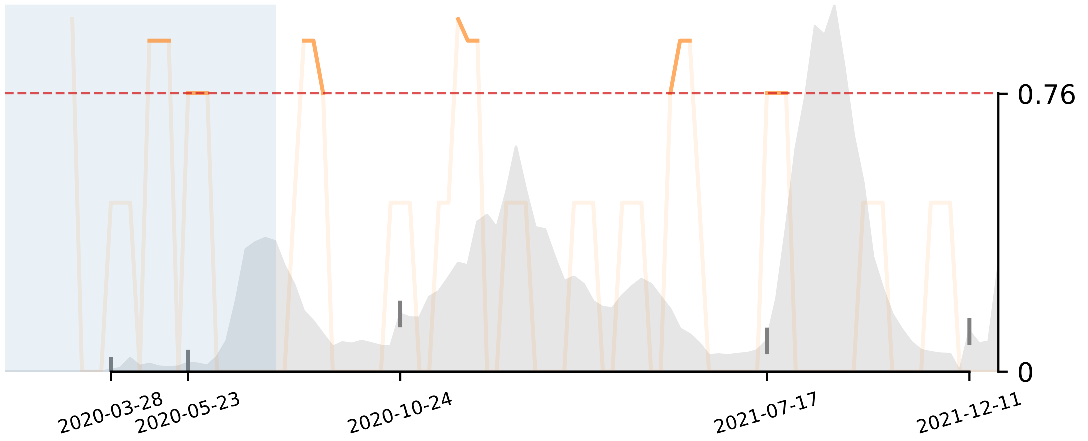

β	Training tp	Training fp	Training fn	Out of sample tp	Out of sample fp	Out of sample fn
0.99	0	0	2	0	0	3
0.96	0	0	2	0	1	3
0.91	1	0	1	0	7	3
0.76	2	0	0	1	11	2
0.46	2	2	0	3	28	0
0.00	2	4	0	3	54	0

Model 3

Apple Mobility (walking)
(GT) covid
(GT) Anosmia
(GT) Dizziness
(GT) Vertigo
Apple Mobility (driving)

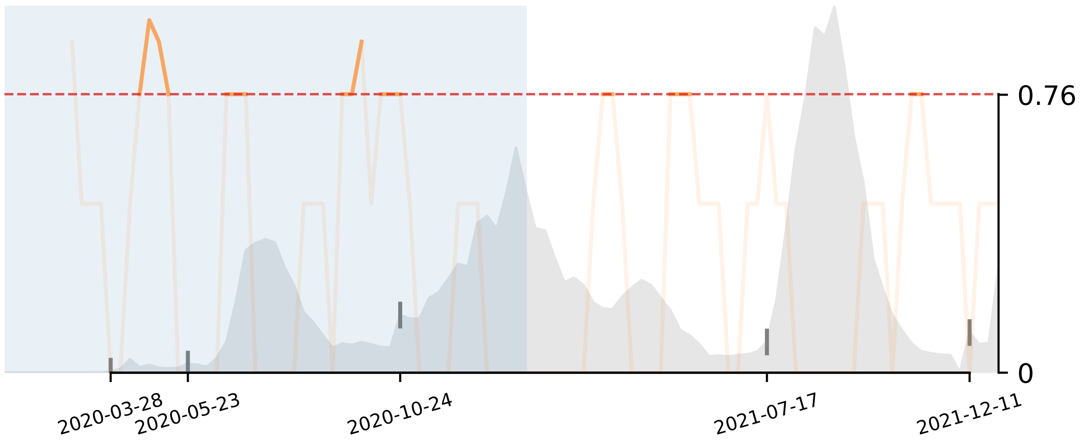

β	Training tp	Training fp	Training fn	Out of sample tp	Out of sample fp	Out of sample fn
1.00	0	0	3	0	0	2
0.99	0	0	3	0	0	2
0.96	0	0	3	0	0	2
0.91	1	0	2	0	0	2
0.76	3	0	0	2	5	0
0.46	3	6	0	2	17	0
0.00	3	14	0	2	35	0

Proxies selected by the Multiple Sources method

Model 1

(GT) covid
(GT) covid-19
(GT) chest pain
(GT) covid symptoms
(GT) quarantine
(GT) nose bleed

Model 2

(GT) covid
(GT) covid-19
(GT) chest pain
(GT) covid symptoms
(GT) quarantine
(GT) Otitis

Model 3

(GT) covid
(GT) covid-19
(GT) covid symptoms
(GT) Otitis
(GT) Pneumonia
(GT) quarantine

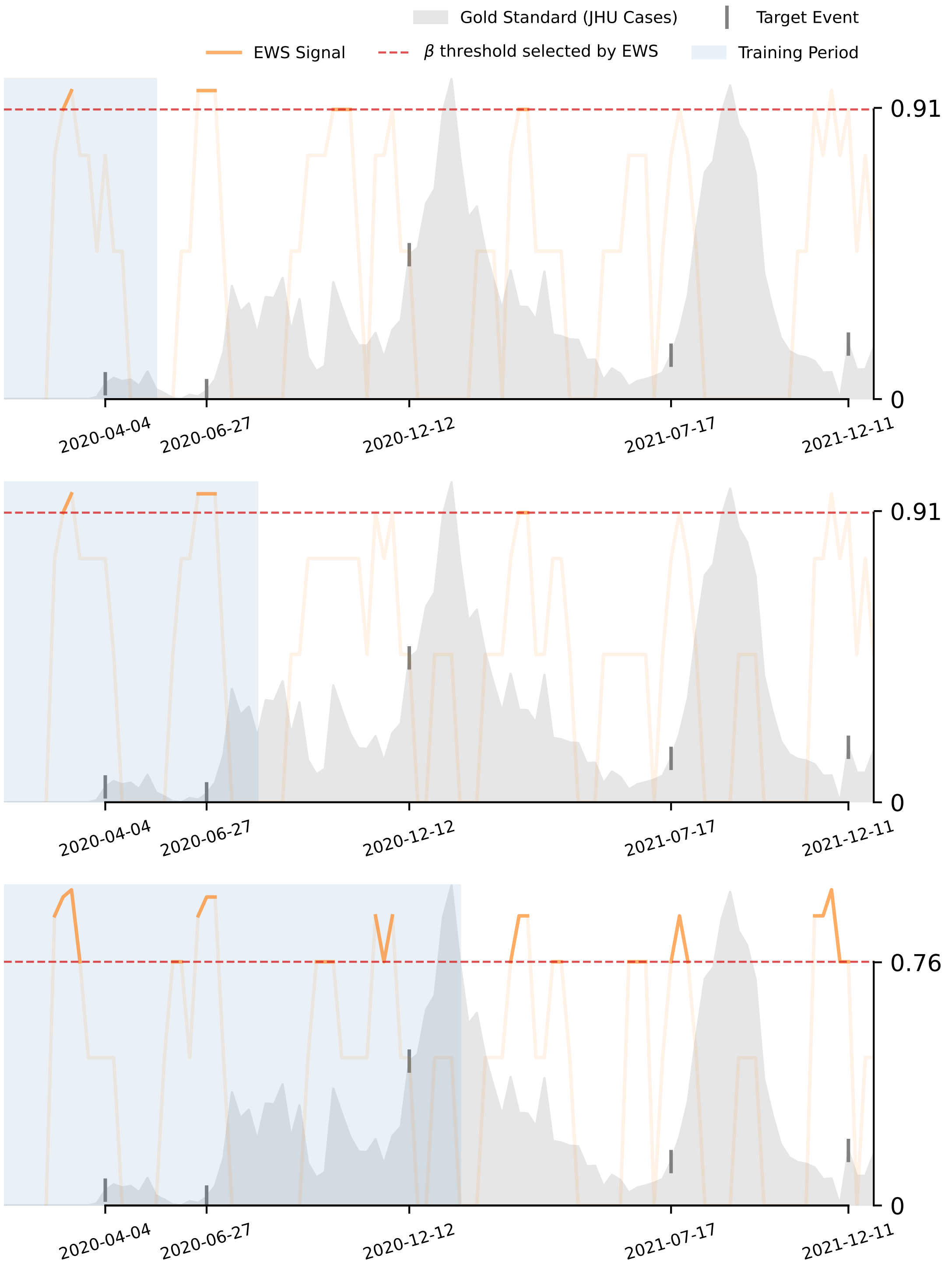

Performance as a function of β

Model 1

β	Training tp	Training fp	Training fn	Out of sample tp	Out of sample fp	Out of sample fn
1.00	0	0	1	0	0	4
0.99	0	0	1	0	0	4
0.96	0	0	1	2	1	2
0.91	1	0	0	3	7	0
0.76	1	1	0	4	13	0
0.46	1	2	0	4	28	0
0.00	1	4	0	4	58	0

Model 2

β	Training tp	Training fp	Training fn	Out of sample tp	Out of sample fp	Out of sample fn
1.00	0	0	2	0	0	3
0.99	0	0	2	0	0	3
0.96	0	0	2	1	0	2
0.91	2	1	0	2	3	0
0.76	2	2	0	3	14	0
0.46	2	3	0	3	33	0
0.00	2	5	0	3	52	0

Model 3

β	Training tp	Training fp	Training fn	Out of sample tp	Out of sample fp	Out of sample fn
1.00	0	0	3	0	0	2
0.99	0	0	3	1	0	1
0.96	1	0	2	1	0	1
0.91	2	2	1	1	3	0
0.76	3	3	0	2	7	0
0.46	3	6	0	2	19	0
0.00	3	17	0	2	35	0

GA, DeKalb

Proxies selected by the Multiple Sources method

Model 1

(GT) covid
(GT) covid-19
(GT) chest pain
(GT) covid symptoms
(GT) chest tightness
(GT) covid-19 who

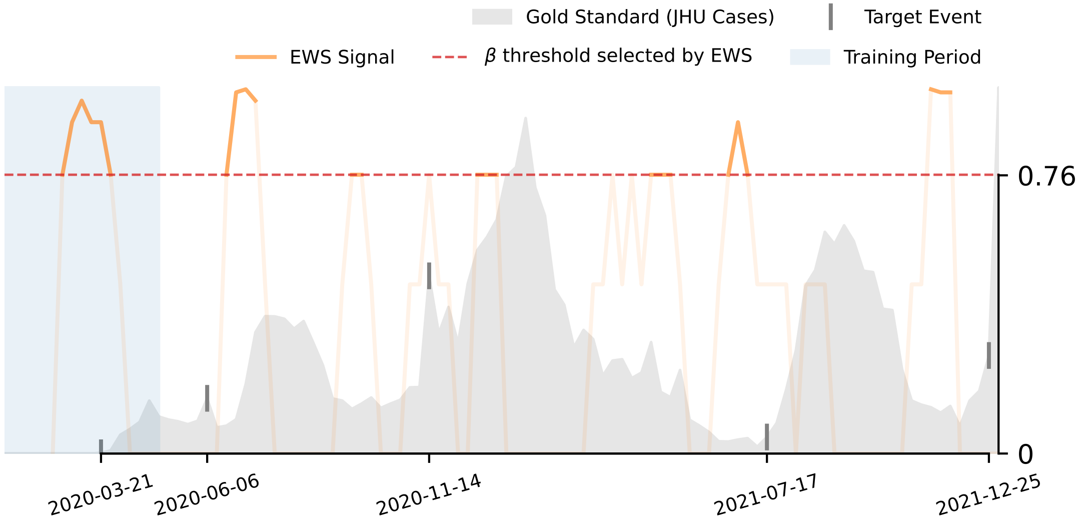

Performance as a function of β

β	Training tp	Training fp	Training fn	Out of sample tp	Out of sample fp	Out of sample fn
1.00	0	0	1	1	1	3
0.99	0	0	1	1	2	3
0.96	0	0	1	1	3	3
0.91	1	0	0	2	3	2
0.76	1	0	0	3	14	0
0.46	1	1	0	3	30	0
0.00	1	2	0	4	61	0

Model 2

(GT) Ageusia
(GT) Chills
(GT) Otitis
Apple Mobility (transit)

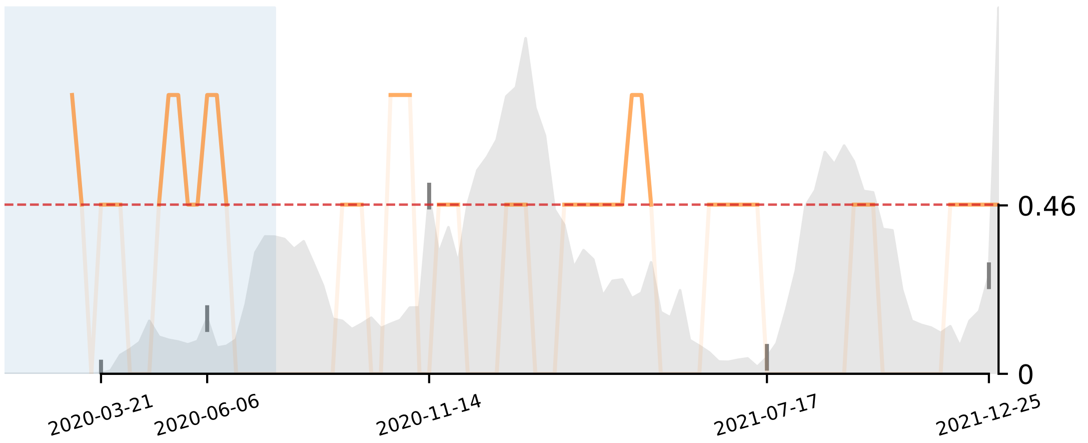

β	Training tp	Training fp	Training fn	Out of sample tp	Out of sample fp	Out of sample fn
0.96	0	0	2	0	0	3
0.91	0	0	2	0	0	3
0.76	0	0	2	1	2	2
0.46	2	1	0	3	23	0
0.00	2	5	0	3	54	0

Model 3

(GT) Ageusia
(GT) Otitis
(GT) how long does covid last
Apple Mobility (transit)
(GT) covid
(GT) covid-19

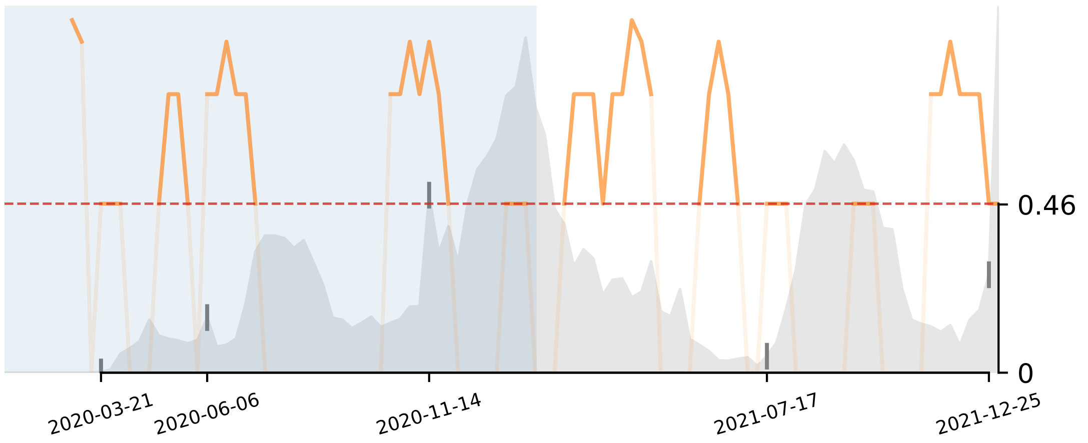

β	Training tp	Training fp	Training fn	Out of sample tp	Out of sample fp	Out of sample fn
1.00	0	0	3	0	0	2
0.99	0	0	3	0	0	2
0.96	0	0	3	0	1	2
0.91	1	0	2	2	2	0
0.76	2	1	1	2	8	0
0.46	3	5	0	2	17	0
0.00	3	13	0	2	34	0

Proxies selected by the Multiple Sources method

Model 1

(GT) covid
(GT) covid-19
(GT) chest pain
(GT) covid symptoms
(GT) chest tightness
(GT) covid-19 who

Model 2

(GT) Ageusia
(GT) Anosmia
(GT) Diarrhea
(GT) Fatigue
(GT) Otitis
Apple Mobility (transit)

Model 3

(GT) Ageusia
(GT) Anosmia
(GT) Diarrhea
(GT) Otitis
(GT) Hyperventilation
Apple Mobility (transit)

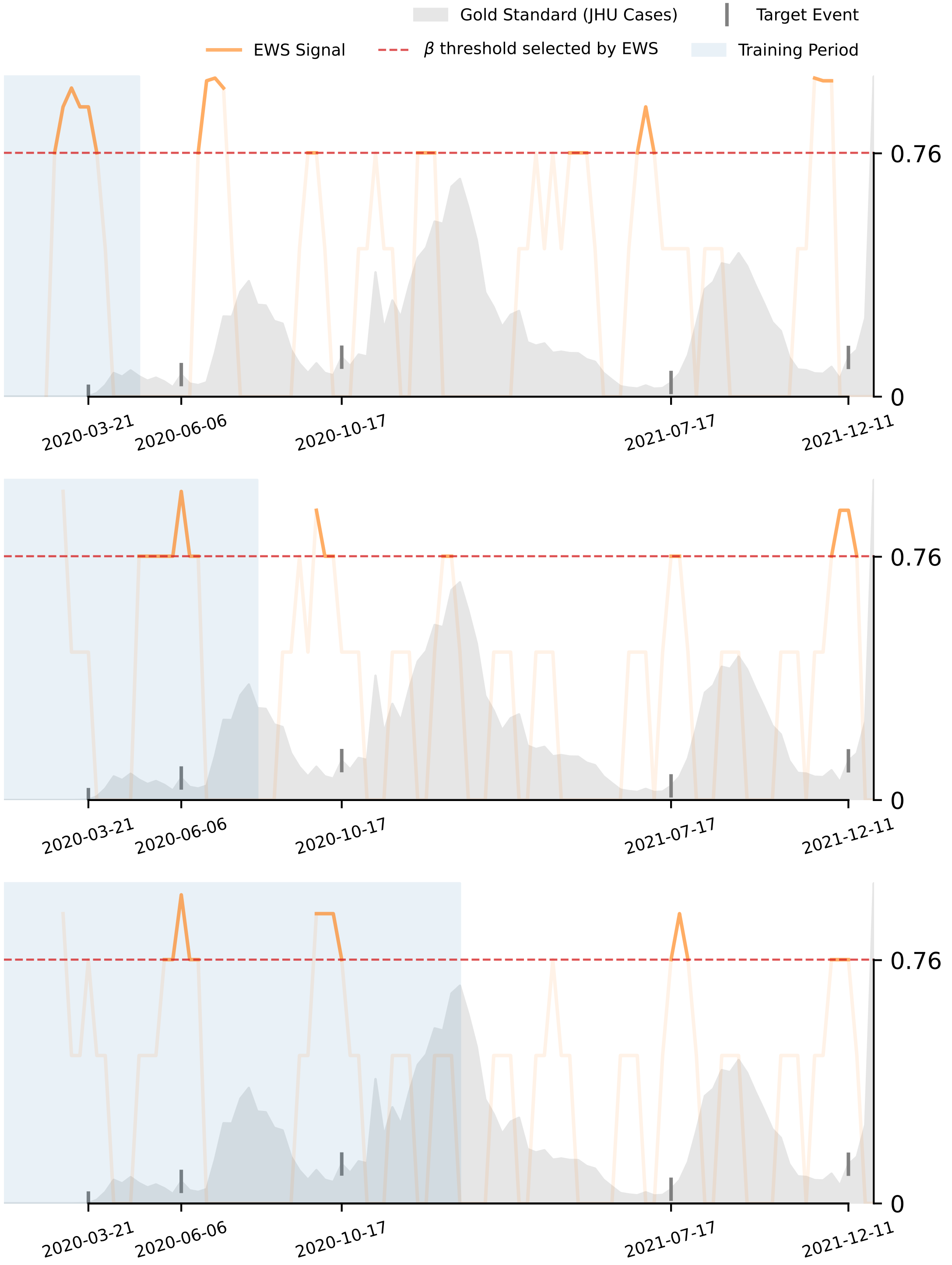

Performance as a function of β

Model 1

β	Training tp	Training fp	Training fn	Out of sample tp	Out of sample fp	Out of sample fn
1.00	0	0	1	1	1	3
0.99	0	0	1	1	2	3
0.96	0	0	1	1	3	3
0.91	1	0	0	2	3	2
0.76	1	0	0	3	13	0
0.46	1	1	0	3	28	0
0.00	1	2	0	4	61	0

Model 2

β	Training tp	Training fp	Training fn	Out of sample tp	Out of sample fp	Out of sample fn
1.00	0	0	2	0	0	3
0.99	0	0	2	0	0	3
0.96	0	0	2	0	0	3
0.91	2	0	0	2	0	1
0.76	2	0	0	3	4	0
0.46	2	3	0	3	24	0
0.00	2	3	0	3	52	0

Model 3

β	Training tp	Training fp	Training fn	Out of sample tp	Out of sample fp	Out of sample fn
1.00	0	0	3	0	0	2
0.99	0	0	3	0	0	2
0.96	0	0	3	0	0	2
0.91	1	0	2	0	1	1
0.76	3	0	0	2	3	0
0.46	3	2	0	2	17	0
0.00	3	14	0	2	35	0

Proxies selected by the Multiple Sources method

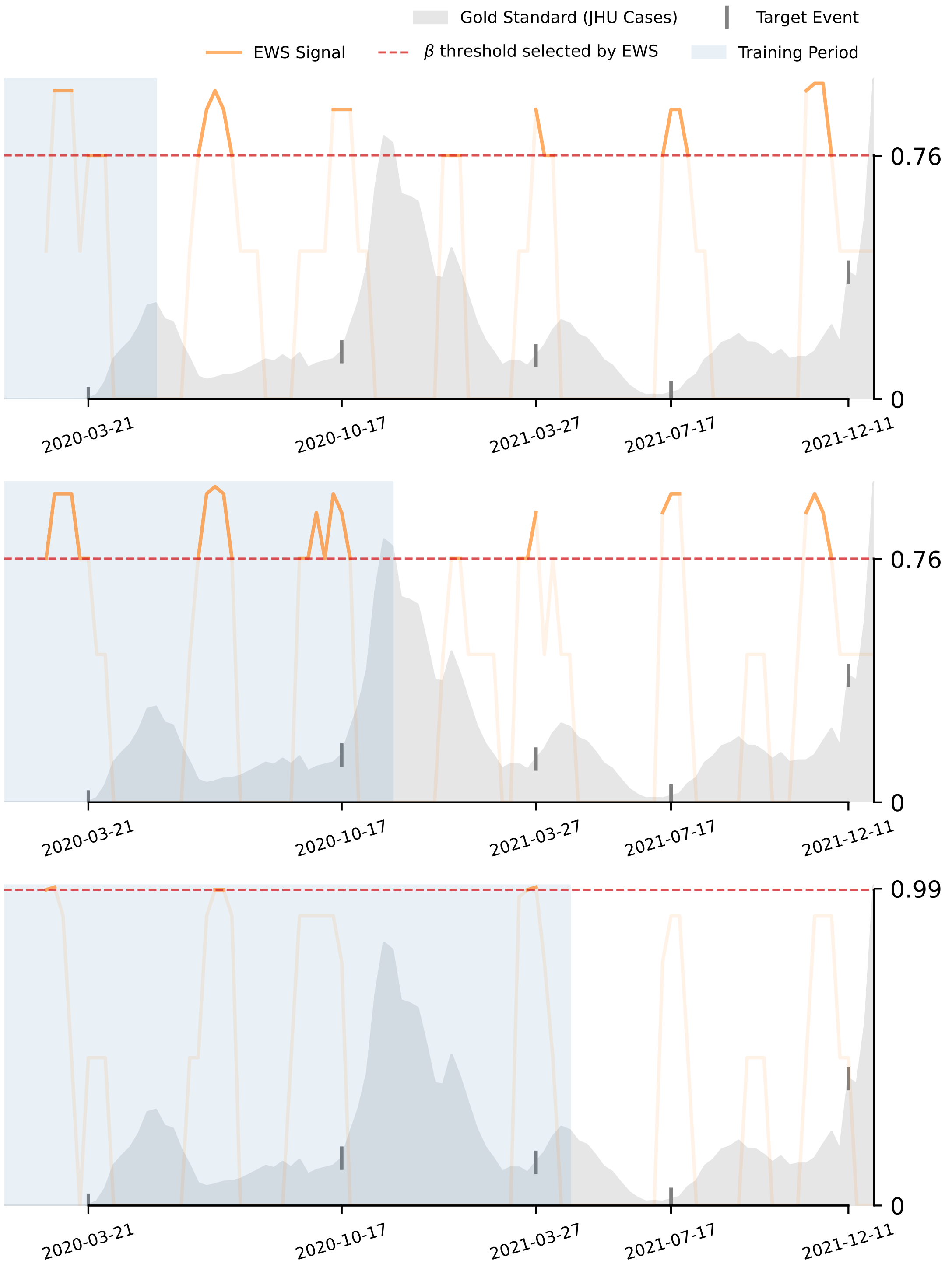

Performance as a function of β

Model 1

(GT) covid
(GT) how long does covid last
(GT) covid-19
(GT) chest pain
(GT) covid symptoms
(GT) chest tightness

β	Training tp	Training fp	Training fn	Out of sample tp	Out of sample fp	Out of sample fn
1.00	0	0	1	0	0	4
0.99	0	0	1	1	0	3
0.96	0	0	1	1	1	3
0.91	1	0	0	4	5	0
0.76	1	0	0	4	13	0
0.46	1	2	0	4	24	0
0.00	1	3	0	4	57	0

Model 2

(GT) covid
(GT) covid-19
(GT) chest pain
(GT) covid symptoms
(GT) quarantine
(GT) Acute bronchitis

β	Training tp	Training fp	Training fn	Out of sample tp	Out of sample fp	Out of sample fn
1.00	0	0	2	0	0	3
0.99	0	0	2	0	0	3
0.96	0	1	2	2	1	1
0.91	2	3	0	3	1	0
0.76	2	3	0	3	4	0
0.46	2	8	0	3	17	0
0.00	2	11	0	3	36	0

Model 3

(GT) chest pain
(GT) covid symptoms
(GT) Ageusia
(GT) Anosmia
(GT) Bronchitis
(GT) Cough

β	Training tp	Training fp	Training fn	Out of sample tp	Out of sample fp	Out of sample fn
1.00	0	0	3	0	0	2
0.99	2	0	1	0	0	2
0.96	2	3	1	0	0	2
0.91	2	3	1	2	1	0
0.76	3	6	0	2	1	0
0.46	3	7	0	2	5	0
0.00	3	13	0	2	22	0

Proxies selected by the Multiple Sources method

Model 1

(GT) covid-19
(GT) covid symptoms
(GT) Abdominal pain
(GT) covid
(GT) Vertigo
(GT) Anxiety

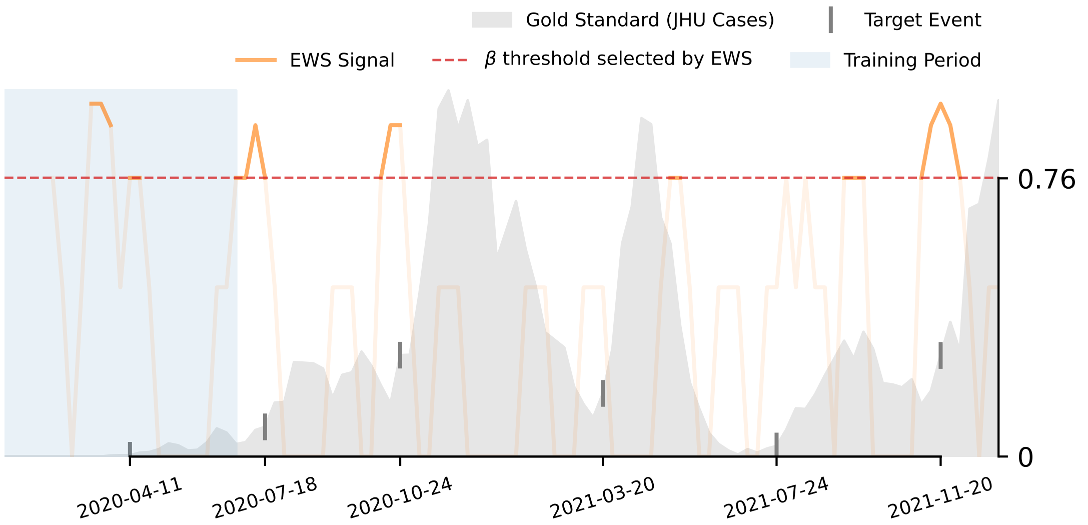

Performance as a function of β

β	Training tp	Training fp	Training fn	Out of sample tp	Out of sample fp	Out of sample fn
1.00	0	0	1	0	0	5
0.99	0	0	1	0	0	5
0.96	0	0	1	1	0	4
0.91	1	0	0	3	1	2
0.76	1	0	0	3	9	1
0.46	1	3	0	5	25	0
0.00	1	8	0	5	48	0

Model 2

(GT) covid
(GT) covid-19
(GT) covid symptoms
(GT) Anxiety
(GT) Chronic pain

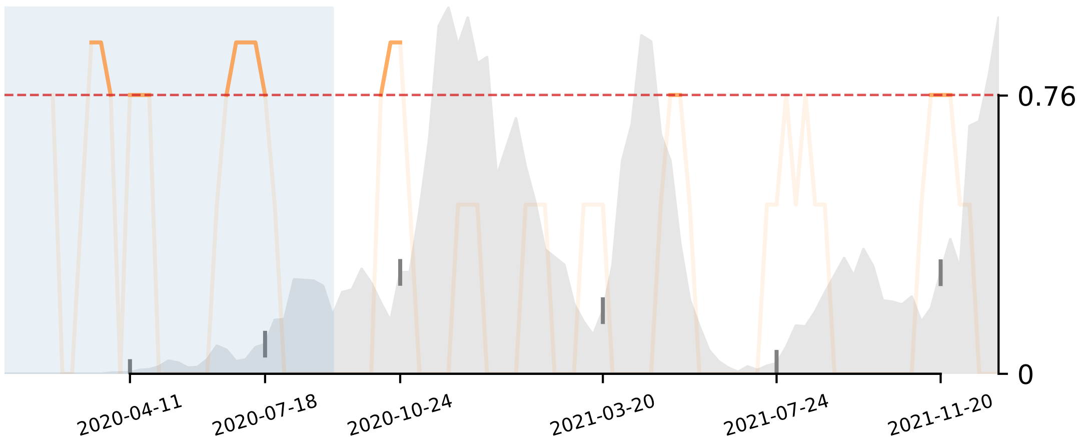

β	Training tp	Training fp	Training fn	Out of sample tp	Out of sample fp	Out of sample fn
0.99	0	0	2	0	0	4
0.96	0	0	2	0	0	4
0.91	0	0	2	1	0	3
0.76	2	0	0	2	5	1
0.46	2	3	0	4	18	0
0.00	2	6	0	4	41	0

Model 3

(GT) covid
(GT) covid-19
(GT) covid symptoms
(GT) Anxiety
(GT) Cough
(GT) Phlegm

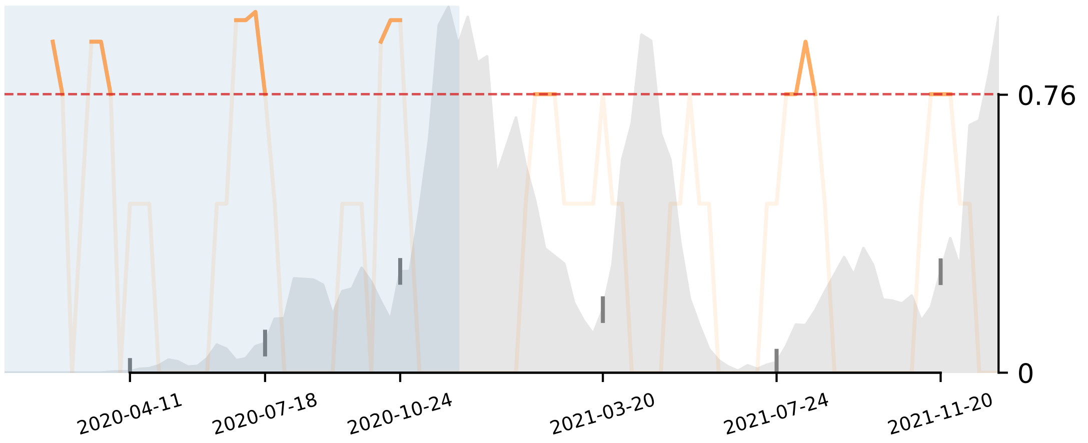

β	Training tp	Training fp	Training fn	Out of sample tp	Out of sample fp	Out of sample fn
1.00	0	0	3	0	0	3
0.99	0	0	3	0	0	3
0.96	1	0	2	0	0	3
0.91	2	0	1	0	1	3
0.76	3	1	0	2	7	0
0.46	3	2	0	3	17	0
0.00	3	10	0	3	35	0

Proxies selected by the Multiple Sources method

Model 1

(GT) covid
(GT) how long does covid last
(GT) quarantine
(GT) covid-19 who
(GT) fever
(GT) Chronic pain

Model 2

(GT) covid
(GT) quarantine
(GT) Otitis
(GT) chest pain

Model 3

(GT) chest pain
(GT) Otitis
(GT) covid
(GT) loss smell
(GT) loss of smell
(GT) covid symptoms

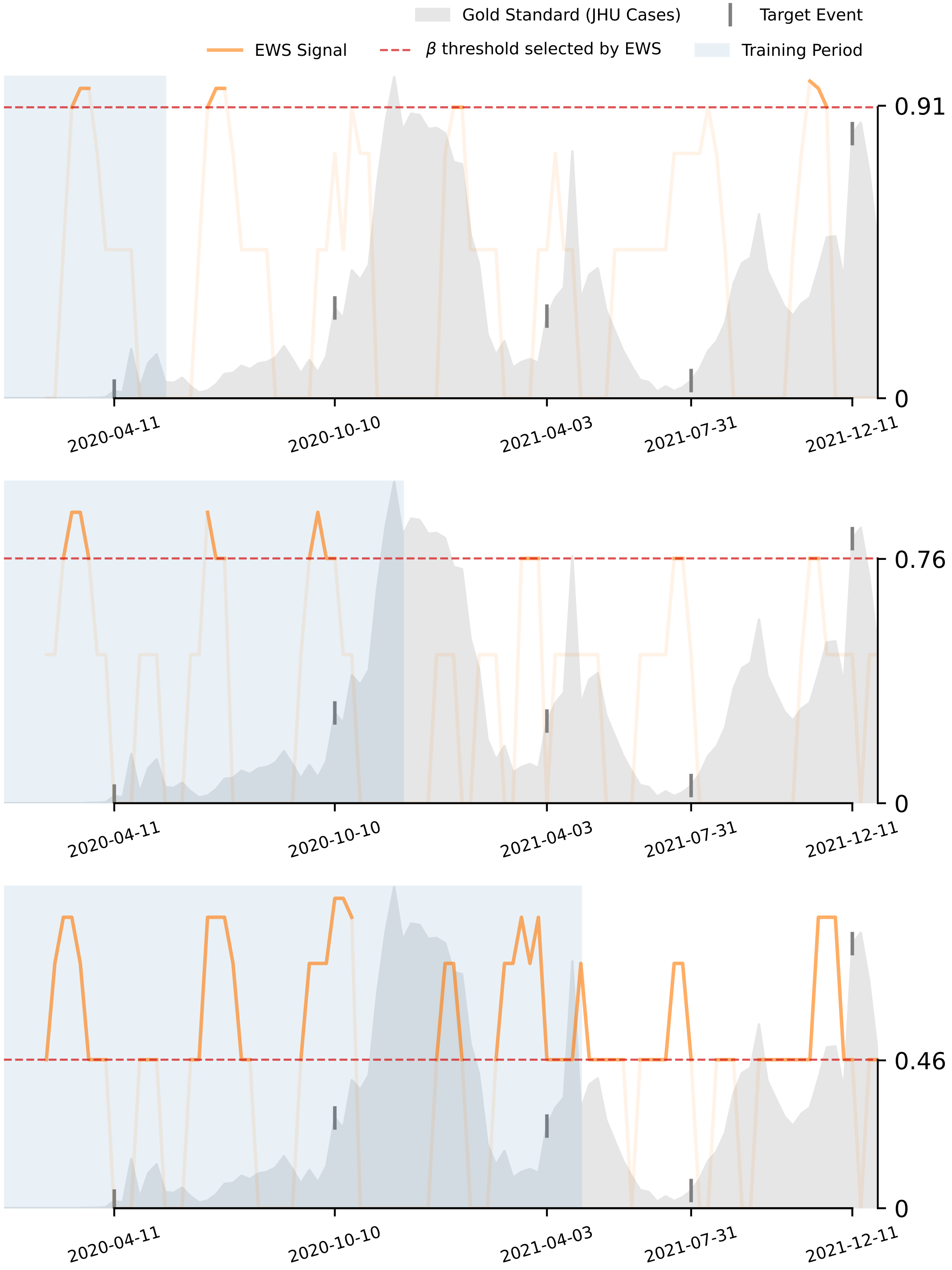

Performance as a function of β

Model 1

β	Training tp	Training fp	Training fn	Out of sample tp	Out of sample fp	Out of sample fn
1.00	0	0	1	0	0	4
0.99	0	0	1	1	0	3
0.96	0	0	1	1	2	3
0.91	1	0	0	1	7	1
0.76	1	1	0	3	14	0
0.46	1	1	0	4	30	0
0.00	1	4	0	4	56	0

Model 2

β	Training tp	Training fp	Training fn	Out of sample tp	Out of sample fp	Out of sample fn
0.96	0	0	2	0	0	3
0.91	0	0	2	0	0	3
0.76	2	2	0	3	0	0
0.46	2	5	0	3	13	0
0.00	2	14	0	3	35	0

Model 3

β	Training tp	Training fp	Training fn	Out of sample tp	Out of sample fp	Out of sample fn
1.00	0	0	3	0	0	2
0.99	0	0	3	0	0	2
0.96	0	0	3	0	0	2
0.91	1	1	2	1	0	1
0.76	2	7	1	2	0	0
0.46	3	13	0	2	15	0
0.00	3	27	0	2	21	0

IN, Lake

Proxies selected by the Multiple Sources method

Model 1

(GT) covid
(GT) how long does covid last
(GT) covid-19
(GT) covid symptoms
(GT) chest tightness
(GT) quarantine

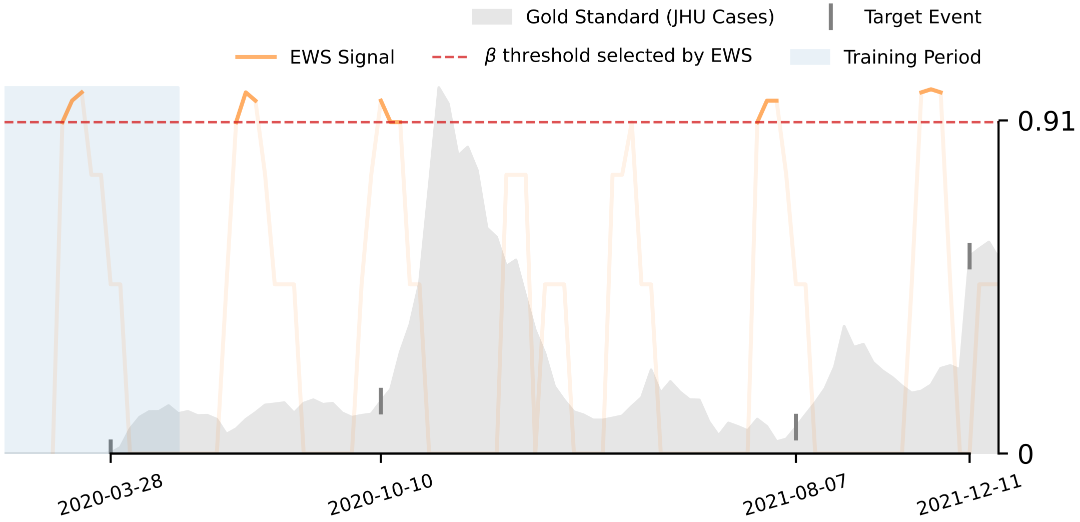

Performance as a function of β

β	Training tp	Training fp	Training fn	Out of sample tp	Out of sample fp	Out of sample fn
1.00	0	0	1	1	0	2
0.99	0	0	1	1	1	2
0.96	1	0	0	3	2	0
0.91	1	0	0	3	6	0
0.76	1	1	0	3	12	0
0.46	1	1	0	3	27	0
0.00	1	2	0	3	64	0

Model 2

(GT) Wheeze
(GT) covid
(GT) covid-19
(GT) covid symptoms
(GT) quarantine
(GT) Acute bronchitis

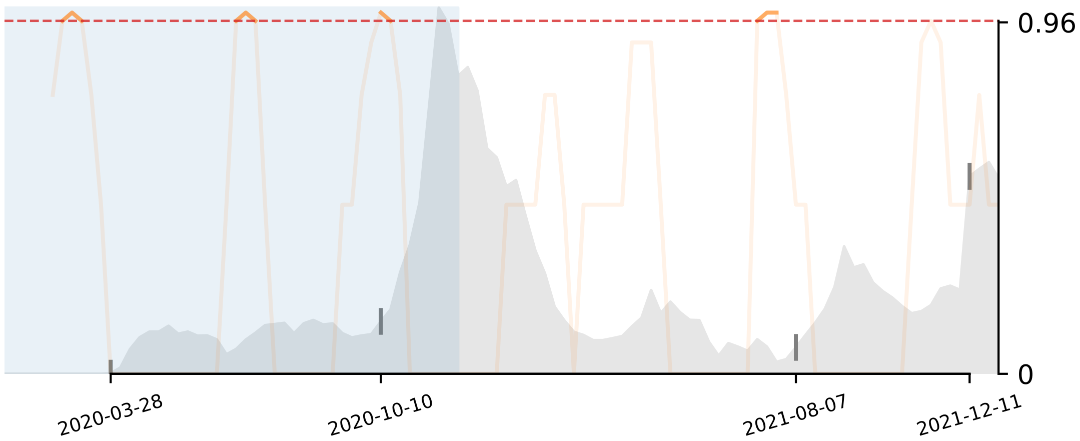

β	Training tp	Training fp	Training fn	Out of sample tp	Out of sample fp	Out of sample fn
1.00	0	0	2	0	0	2
0.99	0	0	2	1	0	1
0.96	2	1	0	2	0	0
0.91	2	5	0	2	3	0
0.76	2	5	0	2	6	0
0.46	2	7	0	2	20	0
0.00	2	9	0	2	42	0

Model 3

(GT) covid
(GT) covid-19
(GT) covid symptoms
(GT) Wheeze
(GT) quarantine
(GT) Acute bronchitis

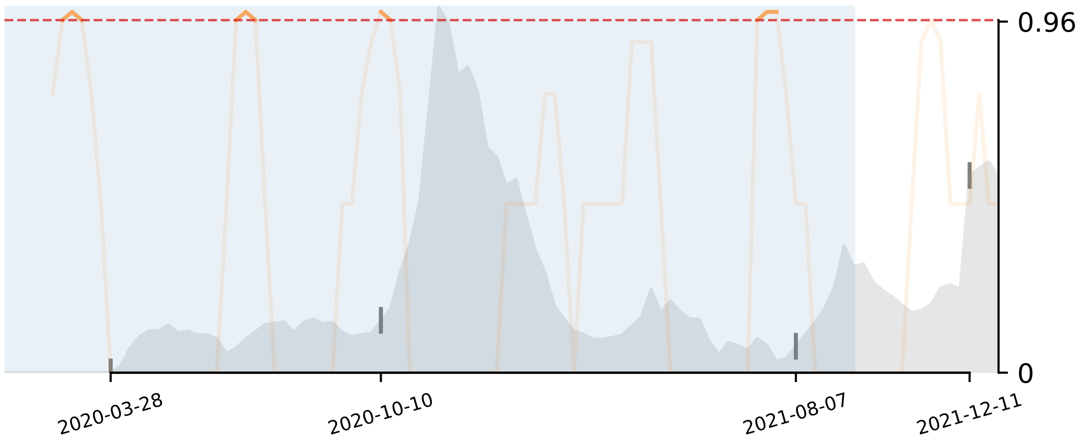

β	Training tp	Training fp	Training fn	Out of sample tp	Out of sample fp	Out of sample fn
1.00	0	0	3	0	0	1
0.99	0	0	3	0	0	1
0.96	3	1	0	1	0	0
0.91	3	5	0	1	0	0
0.76	3	8	0	1	1	0
0.46	3	12	0	1	3	0
0.00	3	26	0	1	8	0

Proxies selected by the Multiple Sources method

Model 1

(GT) covid
(GT) how long does covid last
(GT) covid-19
(GT) covid symptoms
(GT) chest tightness
(GT) quarantine

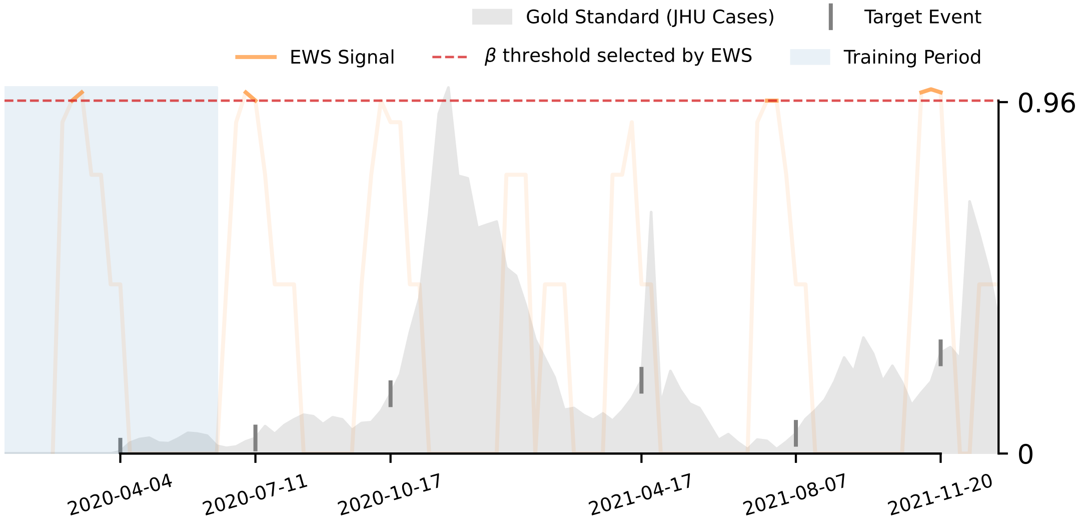

Performance as a function of β

β	Training tp	Training fp	Training fn	Out of sample tp	Out of sample fp	Out of sample fn
1.00	0	0	1	1	0	4
0.99	0	0	1	2	0	3
0.96	1	0	0	4	0	1
0.91	1	1	0	5	1	0
0.76	1	2	0	5	5	0
0.46	1	2	0	5	19	0
0.00	1	2	0	5	49	0

Model 2

(GT) covid
(GT) how long does covid last
(GT) covid-19
(GT) covid symptoms
(GT) quarantine
(GT) covid-19 who

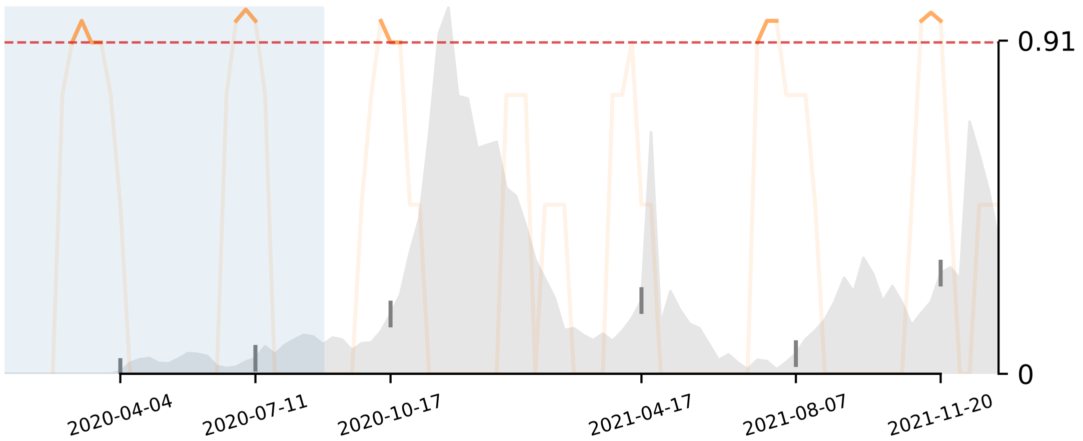

β	Training tp	Training fp	Training fn	Out of sample tp	Out of sample fp	Out of sample fn
1.00	0	0	2	0	0	4
0.99	1	0	1	1	0	3
0.96	1	0	1	3	0	1
0.91	2	0	0	4	1	0
0.76	2	1	0	4	5	0
0.46	2	3	0	4	16	0
0.00	2	3	0	4	42	0

Model 3

(GT) covid
(GT) covid-19
(GT) covid symptoms
(GT) quarantine
(GT) chest pain
(GT) loss smell

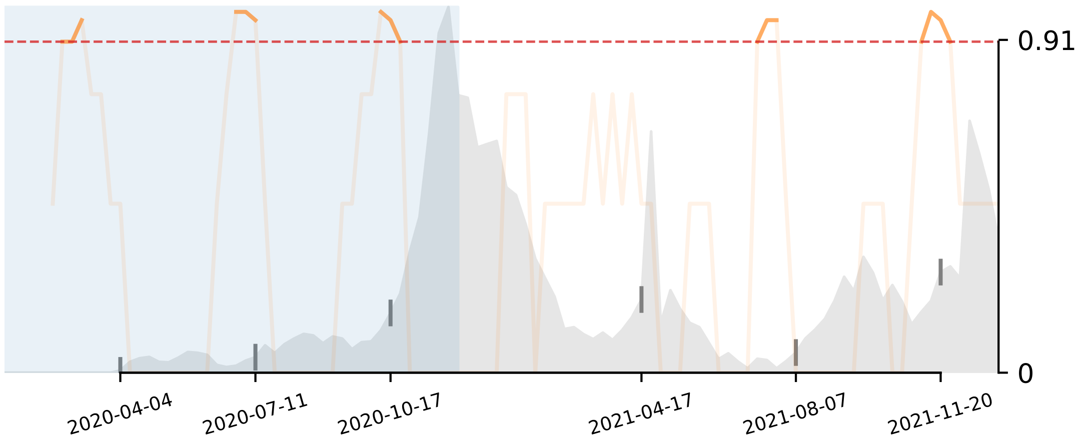

β	Training tp	Training fp	Training fn	Out of sample tp	Out of sample fp	Out of sample fn
1.00	0	0	3	0	0	3
0.99	0	0	3	1	0	2
0.96	2	0	1	2	0	1
0.91	3	0	0	2	1	1
0.76	3	3	0	3	4	0
0.46	3	3	0	3	19	0
0.00	3	6	0	3	35	0

Proxies selected by the Multiple Sources method

Model 1

(GT) covid
(GT) covid-19
(GT) covid symptoms
(GT) quarantine
(GT) nose bleed
(GT) fever

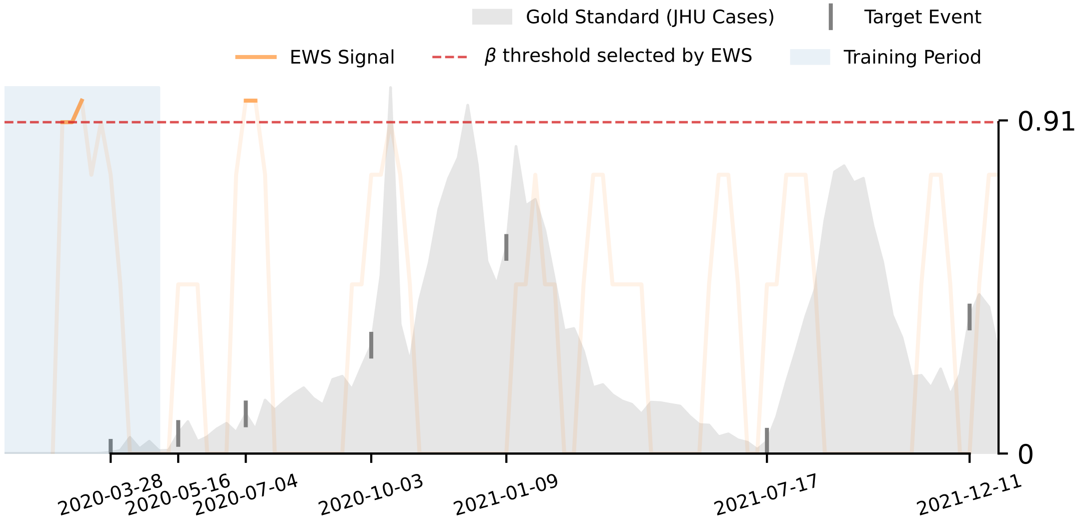

Performance as a function of β

β	Training tp	Training fp	Training fn	Out of sample tp	Out of sample fp	Out of sample fn
1.00	0	0	1	0	0	6
0.99	0	0	1	0	0	6
0.96	0	0	1	1	1	5
0.91	1	0	0	1	2	4
0.76	1	1	0	4	13	2
0.46	1	1	0	5	26	0
0.00	1	2	0	6	50	0

Model 2

(GT) fever
(GT) Abdominal pain
Apple Mobility (driving)
Apple Mobility (walking)
Apple Mobility (transit)

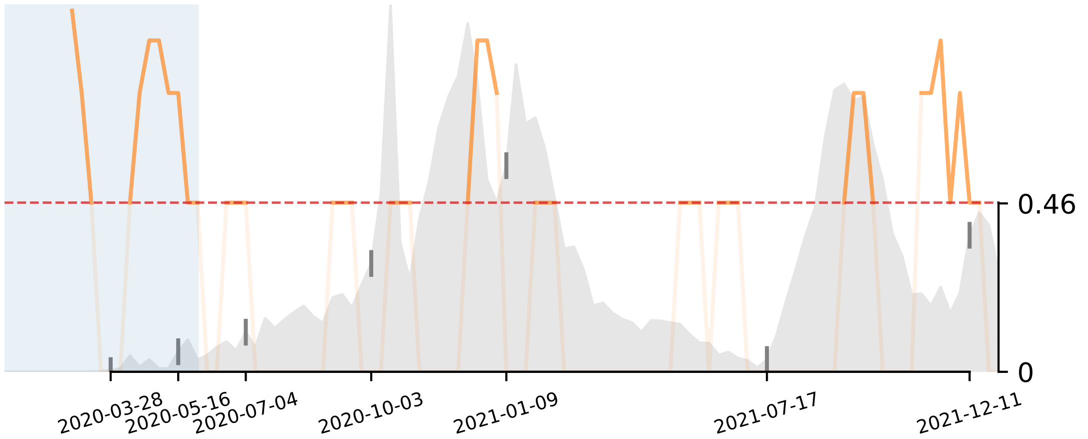

β	Training tp	Training fp	Training fn	Out of sample tp	Out of sample fp	Out of sample fn
0.99	0	0	2	0	0	5
0.96	1	0	1	0	0	5
0.91	1	0	1	2	0	3
0.76	2	0	0	2	2	3
0.46	2	0	0	5	14	0
0.00	2	3	0	5	50	0

Model 3

(GT) Abdominal pain
(GT) covid
(GT) covid-19
(GT) covid symptoms
(GT) quarantine
(GT) fever

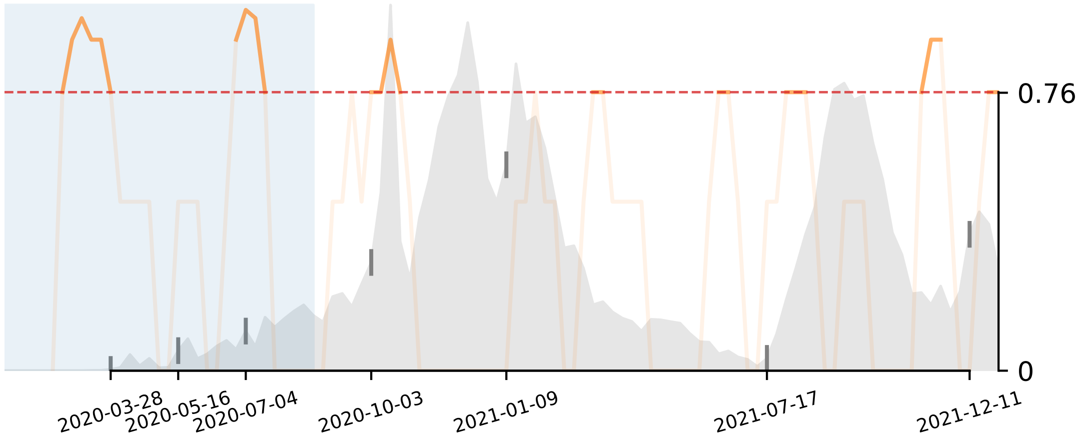

β	Training tp	Training fp	Training fn	Out of sample tp	Out of sample fp	Out of sample fn
1.00	0	0	3	0	0	4
0.99	0	0	3	0	0	4
0.96	1	0	2	0	0	4
0.91	2	1	1	1	1	2
0.76	2	1	1	3	11	1
0.46	2	3	1	3	27	0
0.00	3	7	0	4	44	0

LA, Jefferson

Proxies selected by the Multiple Sources method

Model 1

(GT) covid
(GT) covid-19
(GT) chest pain
(GT) covid symptoms
(GT) quarantine
(GT) fever

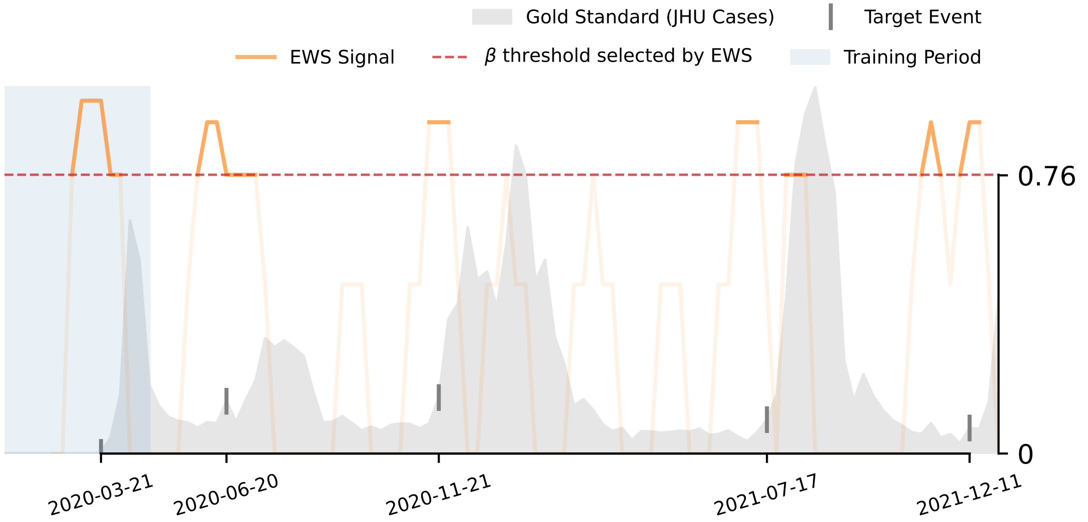

Performance as a function of β

β	Training tp	Training fp	Training fn	Out of sample tp	Out of sample fp	Out of sample fn
1.00	0	0	1	0	0	4
0.99	0	0	1	0	0	4
0.96	0	0	1	0	0	4
0.91	1	0	0	4	2	0
0.76	1	0	0	4	10	0
0.46	1	2	0	4	27	0
0.00	1	2	0	4	60	0

Model 2

(GT) covid-19
(GT) chest pain
(GT) covid symptoms
(GT) fever
(GT) Diarrhea
(GT) Otitis

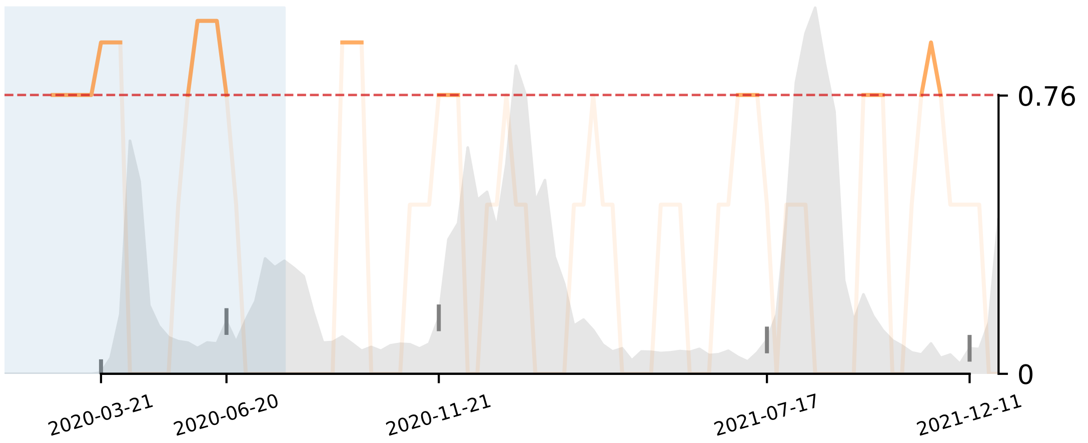

β	Training tp	Training fp	Training fn	Out of sample tp	Out of sample fp	Out of sample fn
1.00	0	0	2	0	0	3
0.99	0	0	2	0	0	3
0.96	0	0	2	0	0	3
0.91	1	0	1	1	3	2
0.76	2	2	0	3	10	0
0.46	2	3	0	3	25	0
0.00	2	5	0	3	53	0

Model 3

(GT) covid-19
(GT) fever
(GT) Otitis
(GT) Chills
(GT) chest pain
(GT) covid

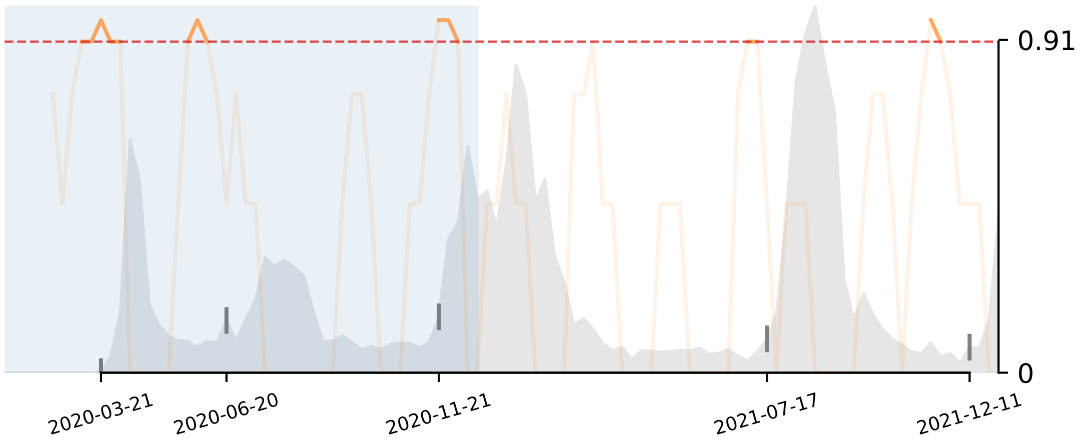

β	Training tp	Training fp	Training fn	Out of sample tp	Out of sample fp	Out of sample fn
1.00	0	0	3	0	0	2
0.99	0	0	3	0	0	2
0.96	0	0	3	1	0	1
0.91	3	1	0	2	1	0
0.76	3	4	0	2	6	0
0.46	3	8	0	2	21	0
0.00	3	13	0	2	40	0

Proxies selected by the Multiple Sources method

Performance as a function of β

Model 1

(GT) covid
(GT) covid-19
(GT) chest pain
(GT) covid symptoms
(GT) quarantine
(GT) fever

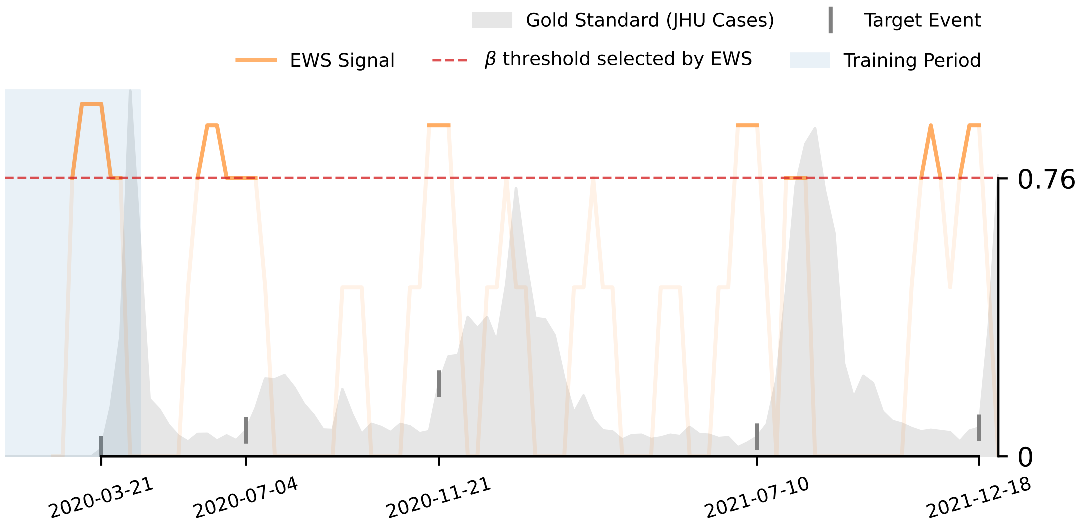

β	Training tp	Training fp	Training fn	Out of sample tp	Out of sample fp	Out of sample fn
1.00	0	0	1	0	0	4
0.99	0	0	1	0	0	4
0.96	0	0	1	0	0	4
0.91	1	0	0	4	1	0
0.76	1	0	0	4	7	0
0.46	1	2	0	4	26	0
0.00	1	2	0	4	61	0

Model 2

(GT) covid
(GT) covid-19
(GT) chest pain
(GT) covid symptoms
(GT) quarantine
(GT) fever

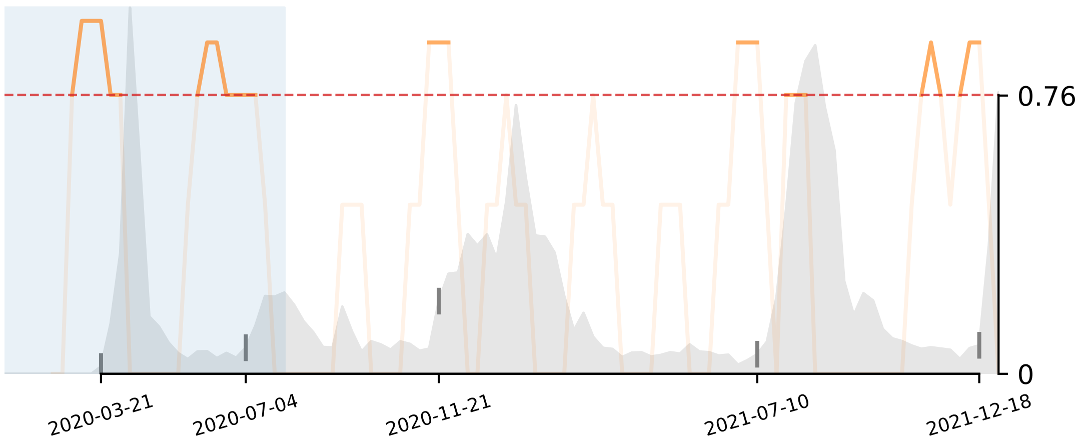

β	Training tp	Training fp	Training fn	Out of sample tp	Out of sample fp	Out of sample fn
1.00	0	0	2	0	0	3
0.99	0	0	2	0	0	3
0.96	0	0	2	0	0	3
0.91	1	0	1	3	1	0
0.76	2	0	0	3	6	0
0.46	2	4	0	3	24	0
0.00	2	6	0	3	53	0

Model 3

(GT) covid
(GT) covid-19
(GT) quarantine
(GT) fever
(GT) Abdominal pain
(GT) Diarrhea

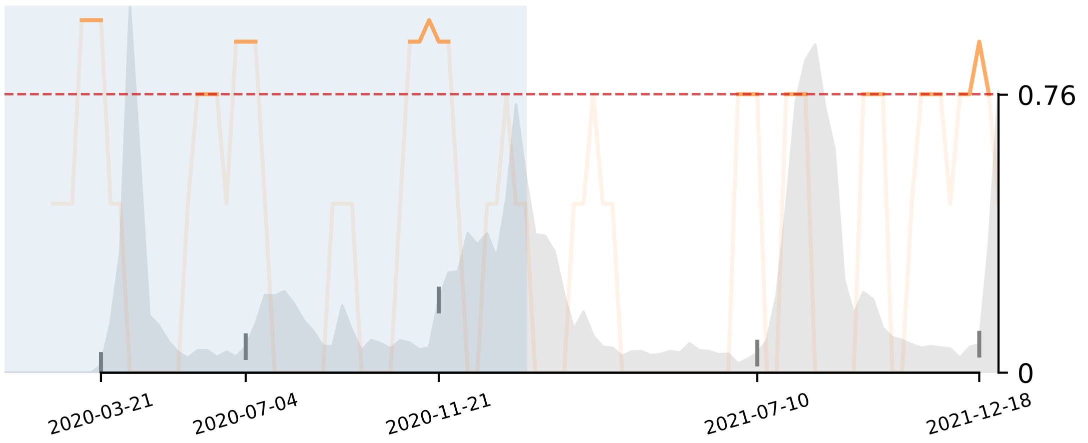

β	Training tp	Training fp	Training fn	Out of sample tp	Out of sample fp	Out of sample fn
1.00	0	0	3	0	0	2
0.99	0	0	3	0	0	2
0.96	0	0	3	0	0	2
0.91	2	0	1	1	0	1
0.76	3	2	0	2	8	0
0.46	3	4	0	2	14	0
0.00	3	17	0	2	35	0

Proxies selected by the Multiple Sources method

Performance as a function of β

Model 1

(GT) covid
(GT) covid-19
(GT) chest pain
(GT) covid symptoms
(GT) quarantine
(GT) covid-19 who

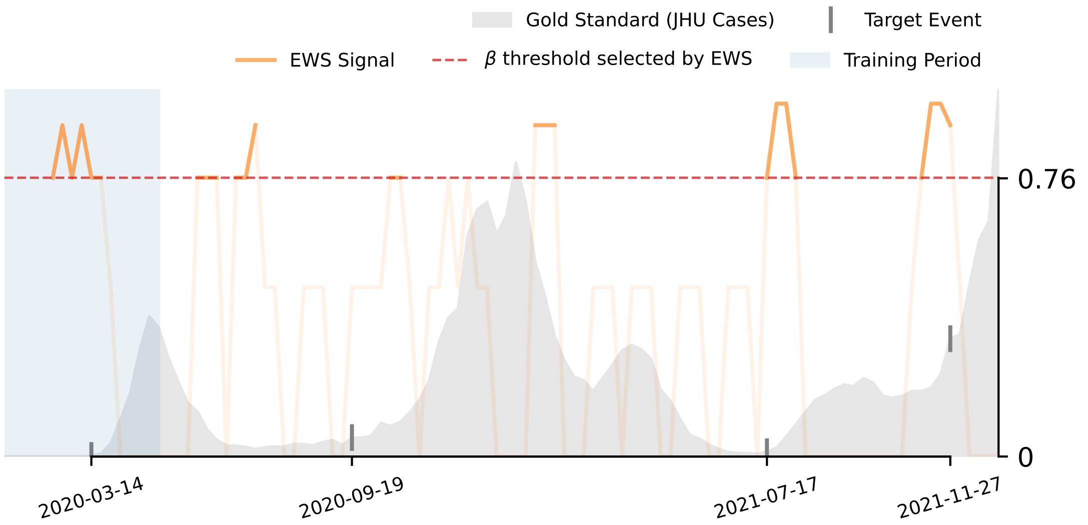

	Training			Out of sample		
β	tp	fp	fn	tp	fp	fn
1.00	0	0	1	0	0	3
0.99	0	0	1	0	0	3
0.96	0	0	1	1	2	1
0.91	0	0	1	1	6	1
0.76	1	0	0	2	16	1
0.46	1	1	0	3	37	0
0.00	1	2	0	3	66	0

Model 2

(GT) Viral pneumonia
(GT) covid-19
(GT) Anosmia
(GT) Burning Chest Pain
(GT) Cardiac arrest
(GT) Hyperventilation

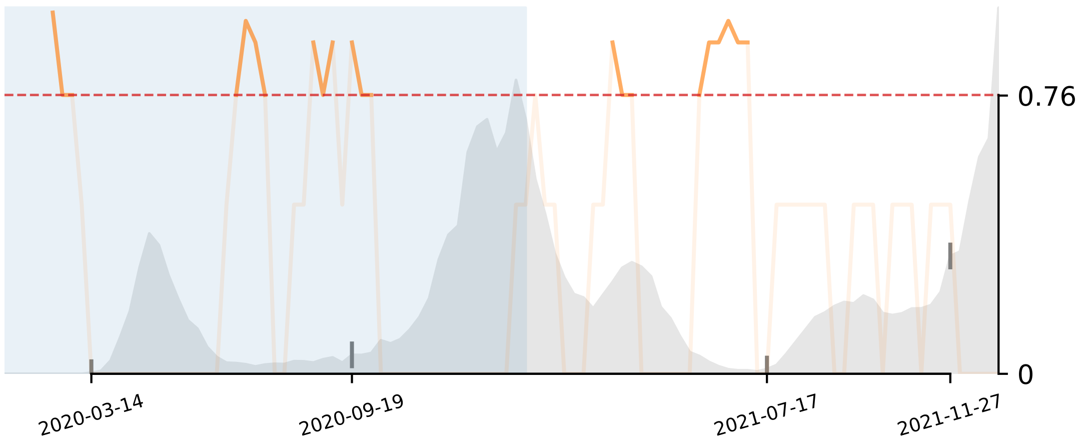

	Training			Out of sample		
β	tp	fp	fn	tp	fp	fn
1.00	0	0	2	0	0	2
0.99	0	0	2	0	0	2
0.96	1	0	1	1	0	1
0.91	1	1	1	1	1	1
0.76	2	2	0	1	5	1
0.46	2	6	0	2	18	0
0.00	2	11	0	2	35	0

Model 3

(GT) Burning Chest Pain
(GT) Viral pneumonia
(GT) covid-19
(GT) Asphyxia
(GT) Hyperventilation
(GT) chest pain

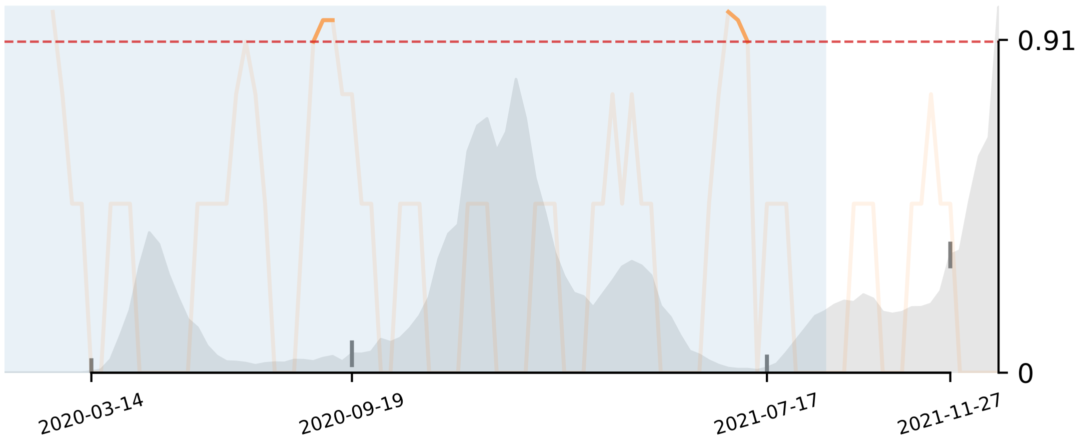

	Training			Out of sample		
β	tp	fp	fn	tp	fp	fn
1.00	0	0	3	0	0	1
0.99	0	0	3	0	0	1
0.96	2	0	1	0	0	1
0.91	3	0	0	0	0	1
0.76	3	1	0	1	0	0
0.46	3	6	0	1	3	0
0.00	3	34	0	1	11	0

Proxies selected by the Multiple Sources method

Model 1

(GT) covid
(GT) covid-19
(GT) chest pain
(GT) covid symptoms
(GT) quarantine
(GT) covid-19 who

Model 2

(GT) covid
(GT) covid-19
(GT) quarantine
(GT) Abdominal pain
(GT) Ageusia
(GT) Anosmia

Model 3

(GT) Ageusia
(GT) Anosmia
(GT) Fever
(GT) Vertigo
(GT) loss smell
(GT) loss of smell

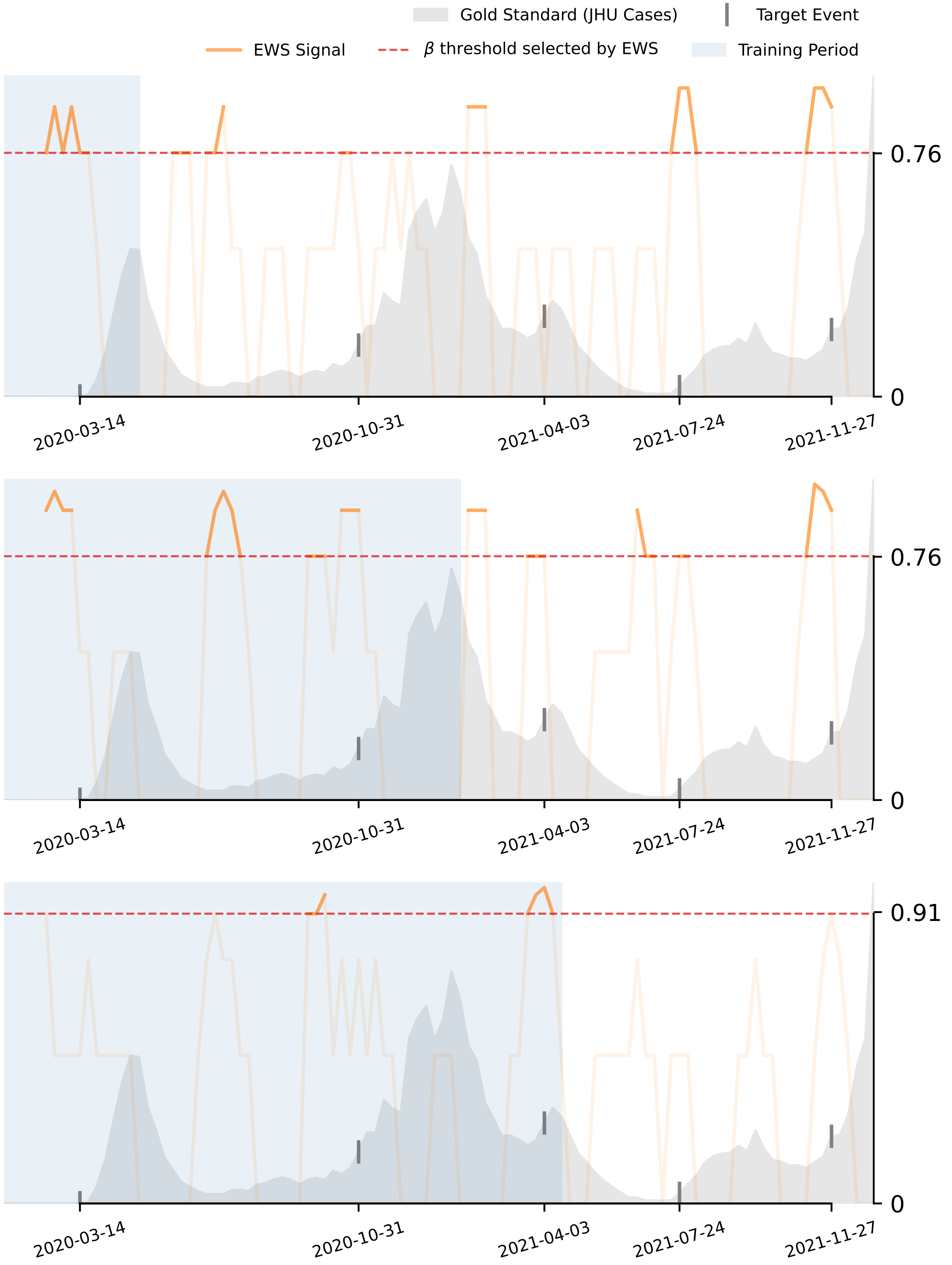

Performance as a function of β

Model 1

β	Training tp	Training fp	Training fn	Out of sample tp	Out of sample fp	Out of sample fn
1.00	0	0	1	0	0	4
0.99	0	0	1	0	0	4
0.96	0	0	1	2	1	2
0.91	0	0	1	2	5	2
0.76	1	0	0	3	13	1
0.46	1	1	0	4	30	0
0.00	1	2	0	4	59	0

Model 2

β	Training tp	Training fp	Training fn	Out of sample tp	Out of sample fp	Out of sample fn
1.00	0	0	2	0	0	3
0.99	0	0	2	1	0	2
0.96	0	0	2	1	0	2
0.91	1	1	1	2	3	1
0.76	2	3	0	3	4	0
0.46	2	7	0	3	9	0
0.00	2	14	0	3	28	0

Model 3

β	Training tp	Training fp	Training fn	Out of sample tp	Out of sample fp	Out of sample fn
1.00	0	0	3	0	0	2
0.99	0	0	3	0	0	2
0.96	1	0	2	0	0	2
0.91	2	0	1	1	0	1
0.76	3	4	0	2	2	0
0.46	3	9	0	2	12	0
0.00	3	24	0	2	23	0

Proxies selected by the Multiple Sources method

Model 1

(GT) covid
(GT) covid-19
(GT) chest pain
(GT) covid symptoms
(GT) quarantine
(GT) covid-19 who

Model 2

(GT) covid
(GT) covid-19
(GT) chest pain
(GT) Acute bronchitis
(GT) Ageusia
(GT) Bronchitis

Model 3

(GT) covid
(GT) chest pain
(GT) Acute bronchitis
(GT) Ageusia
(GT) Phlegm
(GT) quarantine

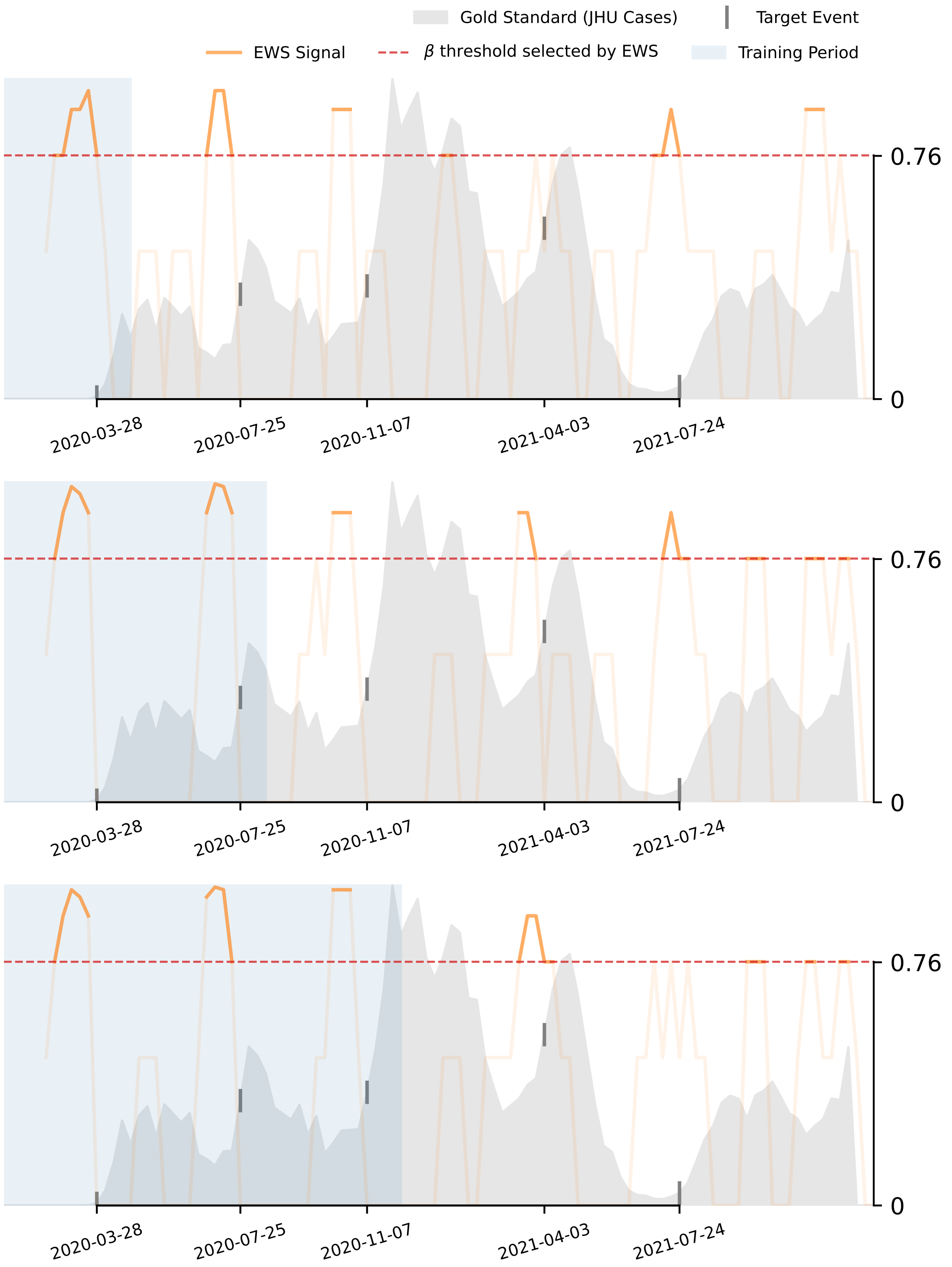

Performance as a function of β

Model 1

β	Training tp	Training fp	Training fn	Out of sample tp	Out of sample fp	Out of sample fn
1.00	0	0	1	0	0	4
0.99	0	0	1	0	0	4
0.96	0	0	1	1	0	3
0.91	1	0	0	3	3	1
0.76	1	0	0	4	7	0
0.46	1	1	0	4	35	0
0.00	1	3	0	4	60	0

Model 2

β	Training tp	Training fp	Training fn	Out of sample tp	Out of sample fp	Out of sample fn
1.00	0	0	2	0	0	3
0.99	1	0	1	0	0	3
0.96	2	0	0	0	0	3
0.91	2	0	0	3	0	0
0.76	2	0	0	3	9	0
0.46	2	1	0	3	25	0
0.00	2	3	0	3	51	0

Model 3

β	Training tp	Training fp	Training fn	Out of sample tp	Out of sample fp	Out of sample fn
1.00	0	0	3	0	0	2
0.99	1	0	2	0	0	2
0.96	3	0	0	0	0	2
0.91	3	0	0	1	0	1
0.76	3	0	0	2	9	0
0.46	3	1	0	2	21	0
0.00	3	8	0	2	42	0

Proxies selected by the Multiple Sources method

Model 1

(GT) covid
(GT) how long does covid last
(GT) covid-19
(GT) chest pain
(GT) covid symptoms
(GT) quarantine

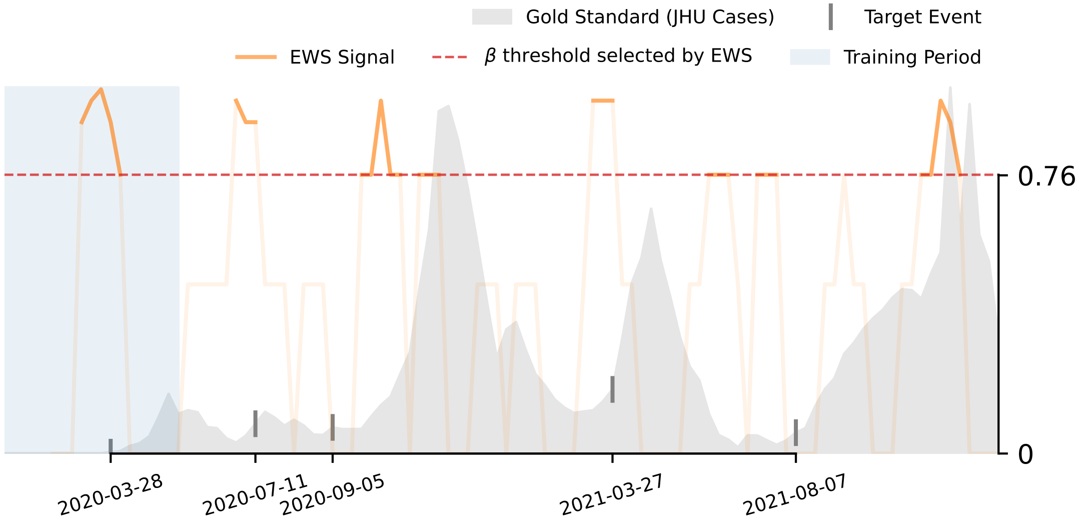

Performance as a function of β

β	Training tp	Training fp	Training fn	Out of sample tp	Out of sample fp	Out of sample fn
1.00	0	0	1	0	0	4
0.99	1	0	0	0	0	4
0.96	1	0	0	2	2	2
0.91	1	0	0	2	3	2
0.76	1	0	0	3	17	1
0.46	1	1	0	4	35	0
0.00	1	1	0	4	57	0

Model 2

(GT) covid
(GT) covid-19
(GT) chest pain
(GT) covid symptoms
(GT) Anosmia
(GT) Asthma

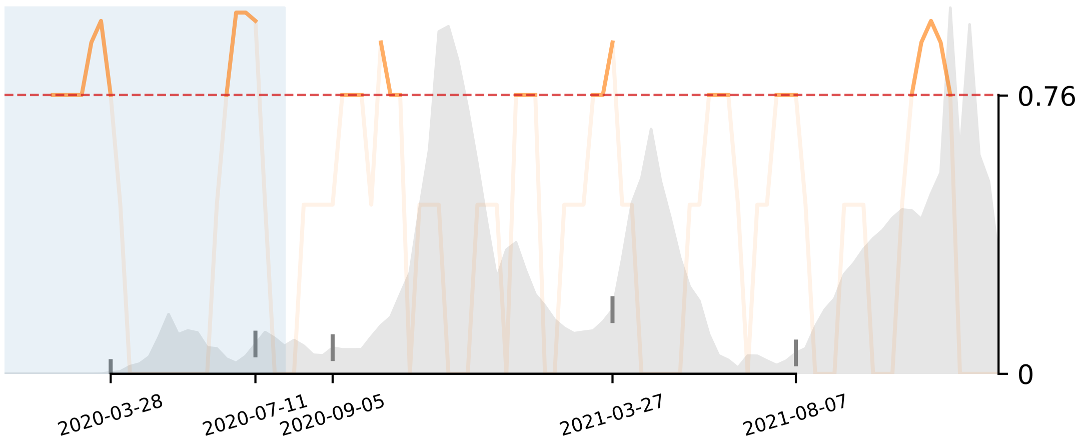

β	Training tp	Training fp	Training fn	Out of sample tp	Out of sample fp	Out of sample fn
1.00	0	0	2	0	0	3
0.99	0	0	2	0	0	3
0.96	1	0	1	0	1	3
0.91	2	0	0	1	4	2
0.76	2	0	0	2	17	0
0.46	2	2	0	3	33	0
0.00	2	4	0	3	55	0

Model 3

(GT) Anosmia
(GT) chest pain
(GT) Nasal congestion
(GT) Wheeze
(GT) covid
(GT) covid-19

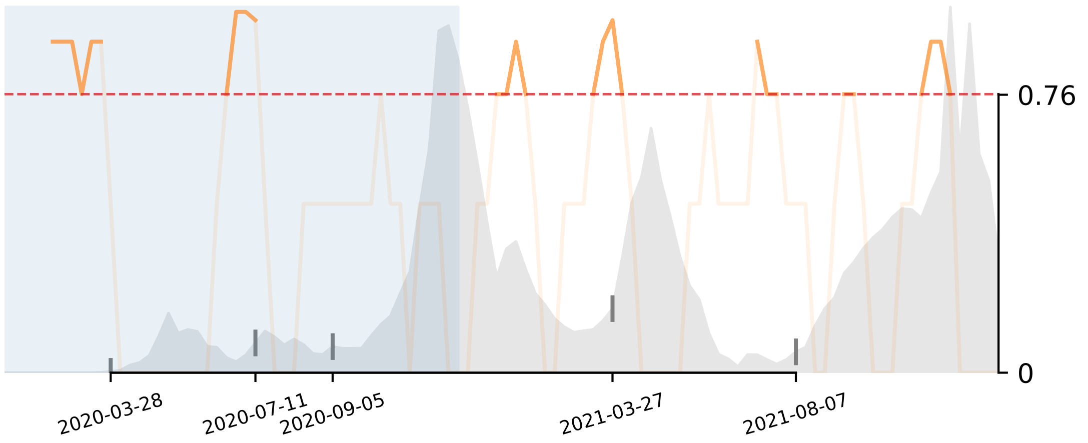

β	Training tp	Training fp	Training fn	Out of sample tp	Out of sample fp	Out of sample fn
1.00	0	0	3	0	0	2
0.99	0	0	3	0	0	2
0.96	1	0	2	1	0	1
0.91	1	0	2	2	3	0
0.76	2	2	1	2	12	0
0.46	2	3	1	2	25	0
0.00	3	13	0	2	42	0

MI, Oakland

Proxies selected by the Multiple Sources method

Model 1

(GT) covid
(GT) covid-19
(GT) chest pain
(GT) covid symptoms
(GT) quarantine
(GT) covid-19 who

Model 2

(GT) covid
(GT) covid-19
(GT) chest pain
(GT) Ageusia
(GT) Anosmia
(GT) Asthma

Model 3

(GT) covid
(GT) covid-19
(GT) Ageusia
(GT) Anosmia
(GT) Asthma
(GT) Chills

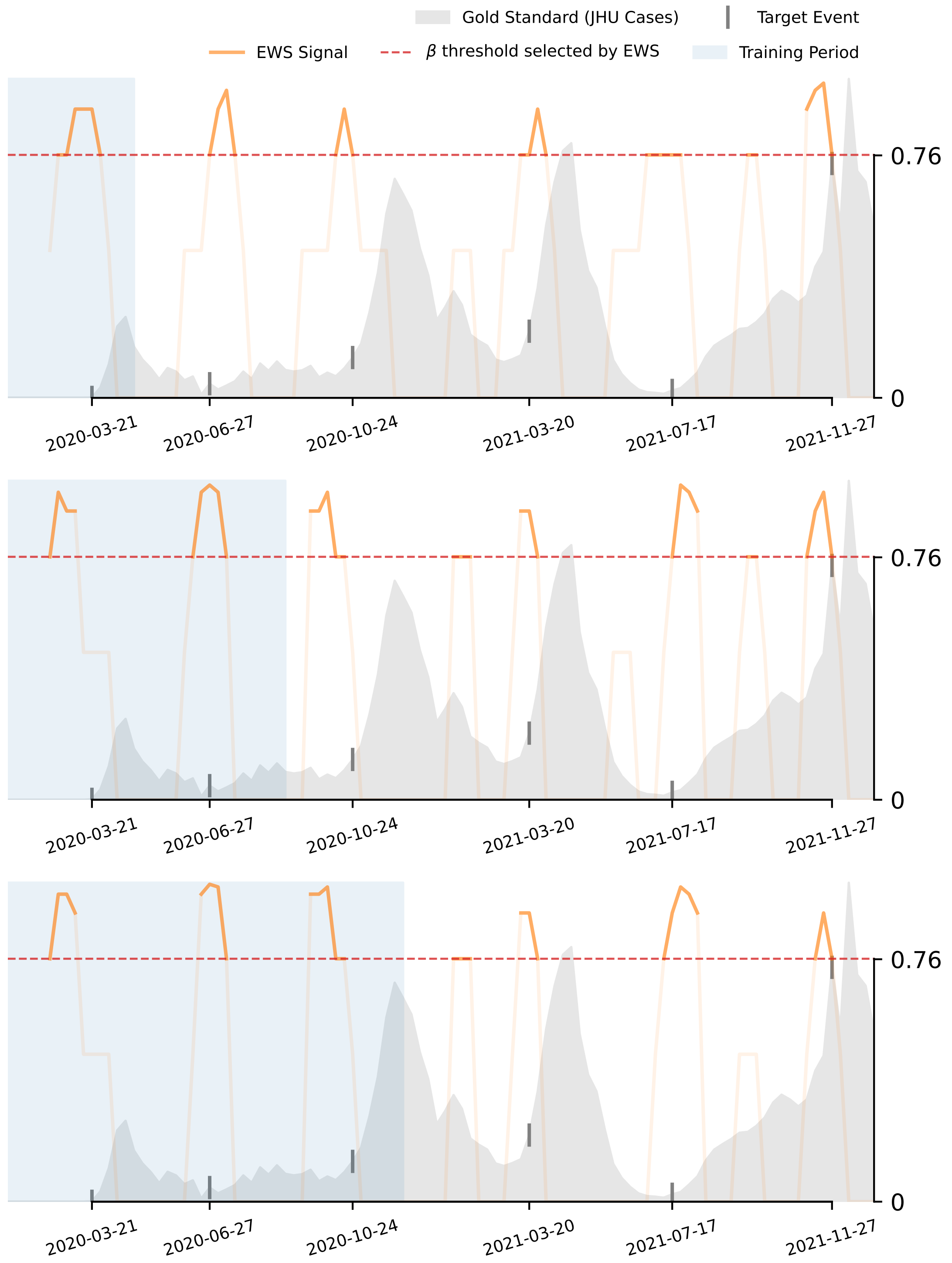

Performance as a function of β

Model 1

β	Training tp	Training fp	Training fn	Out of sample tp	Out of sample fp	Out of sample fn
1.00	0	0	1	0	0	5
0.99	0	0	1	1	0	4
0.96	0	0	1	1	1	3
0.91	0	0	1	2	3	1
0.76	1	0	0	5	8	0
0.46	1	1	0	5	22	0
0.00	1	3	0	5	53	0

Model 2

β	Training tp	Training fp	Training fn	Out of sample tp	Out of sample fp	Out of sample fn
1.00	0	0	2	0	0	4
0.99	0	0	2	0	1	3
0.96	1	0	1	2	2	1
0.91	2	1	0	3	3	0
0.76	2	1	0	4	9	0
0.46	2	3	0	4	13	0
0.00	2	5	0	4	42	0

Model 3

β	Training tp	Training fp	Training fn	Out of sample tp	Out of sample fp	Out of sample fn
1.00	0	0	3	0	0	3
0.99	1	0	2	0	1	2
0.96	2	1	1	0	2	2
0.91	3	2	0	3	3	0
0.76	3	2	0	3	7	0
0.46	3	4	0	3	11	0
0.00	3	6	0	3	35	0

Proxies selected by the Multiple Sources method

Model 1

(GT) covid
(GT) covid-19
(GT) covid symptoms
(GT) quarantine
(GT) covid-19 who
(GT) Nasal congestion

Model 2

(GT) covid
(GT) covid-19
(GT) covid symptoms
(GT) quarantine
(GT) Nasal congestion
(GT) Phlegm

Model 3

(GT) covid-19
(GT) covid symptoms
(GT) loss smell
(GT) Nasal congestion
(GT) Phlegm
(GT) covid

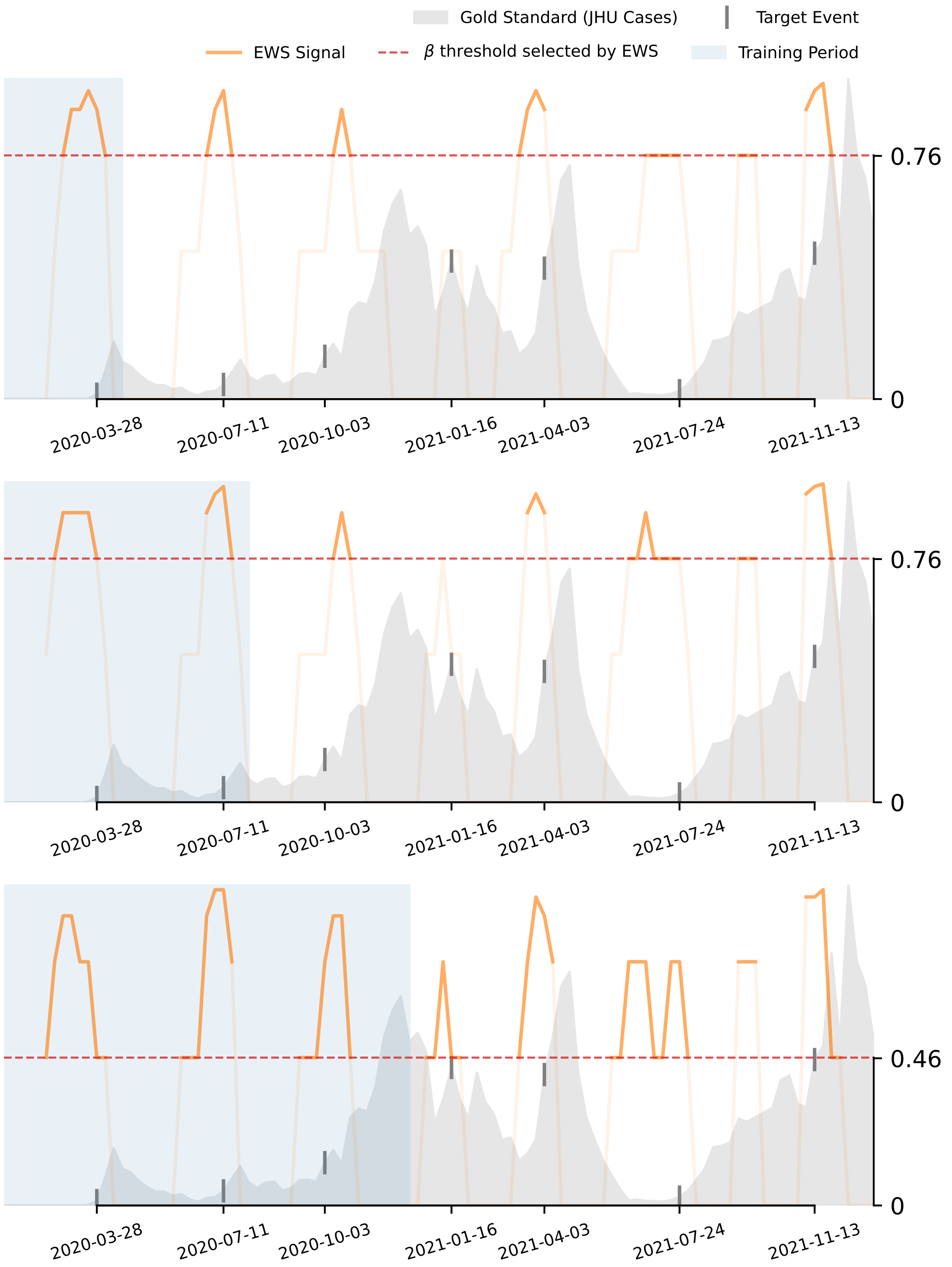

Performance as a function of β

Model 1

β	Training tp	Training fp	Training fn	Out of sample tp	Out of sample fp	Out of sample fn
1.00	0	0	1	0	0	6
0.99	0	0	1	0	1	5
0.96	0	0	1	3	1	3
0.91	1	0	0	3	2	2
0.76	1	0	0	4	9	1
0.46	1	1	0	6	20	0
0.00	1	2	0	6	47	0

Model 2

β	Training tp	Training fp	Training fn	Out of sample tp	Out of sample fp	Out of sample fn
1.00	0	0	2	0	1	4
0.99	0	0	2	1	1	4
0.96	1	0	1	2	1	3
0.91	1	0	1	3	2	1
0.76	2	0	0	4	8	0
0.46	2	2	0	5	15	0
0.00	2	6	0	5	39	0

Model 3

β	Training tp	Training fp	Training fn	Out of sample tp	Out of sample fp	Out of sample fn
1.00	0	0	3	0	0	4
0.99	0	0	3	0	1	3
0.96	1	0	2	2	1	2
0.91	1	0	2	2	1	2
0.76	2	2	1	4	5	0
0.46	3	4	0	4	11	0
0.00	3	8	0	4	29	0

MI, Wayne

Proxies selected by the Multiple Sources method

Model 1

(GT) covid
(GT) covid-19
(GT) chest pain
(GT) covid symptoms
(GT) quarantine
(GT) covid-19 who

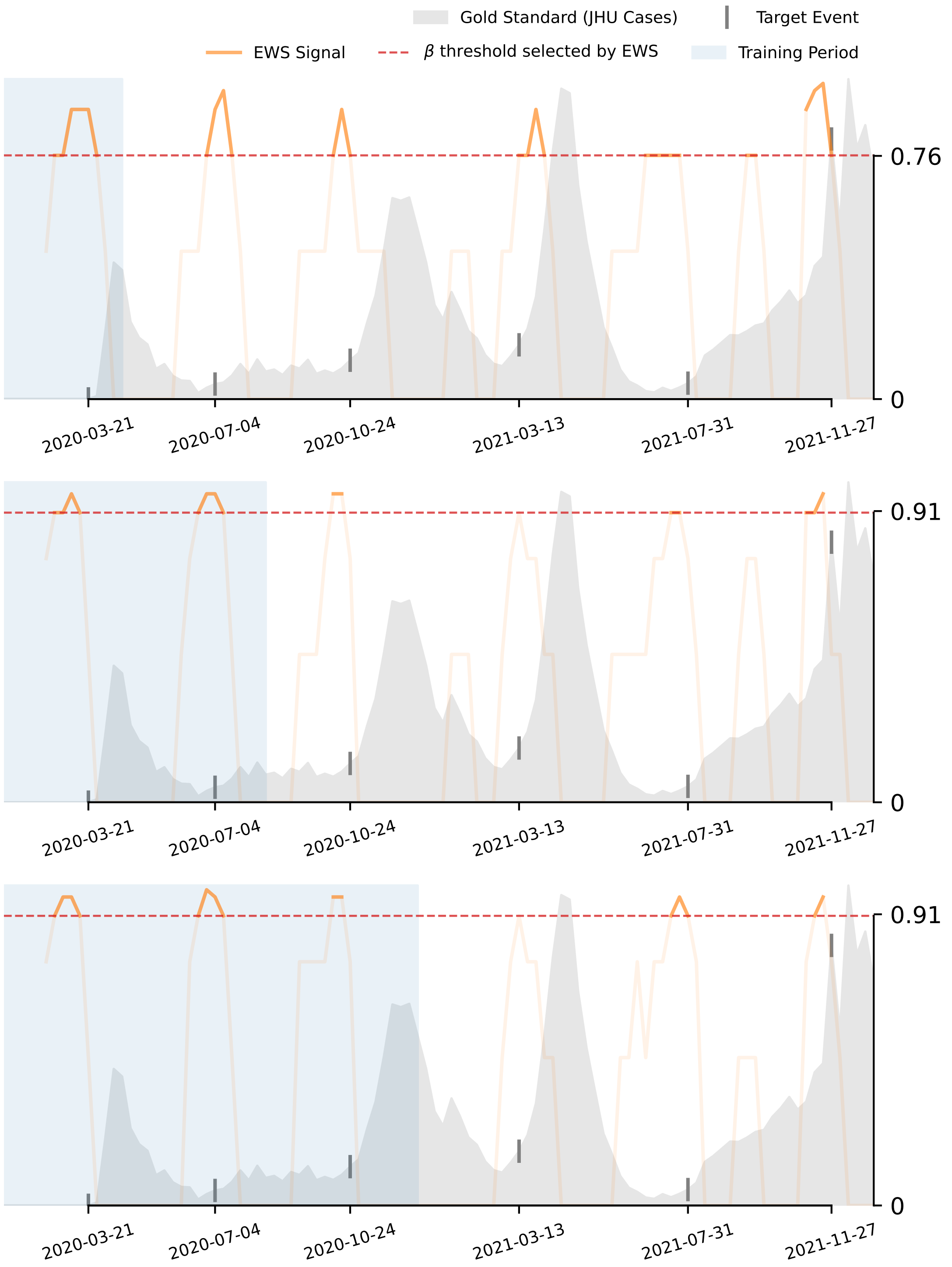

Performance as a function of β

β	Training tp	Training fp	Training fn	Out of sample tp	Out of sample fp	Out of sample fn
1.00	0	0	1	0	0	5
0.99	0	0	1	1	0	4
0.96	0	0	1	1	1	3
0.91	0	0	1	3	2	1
0.76	1	0	0	5	7	0
0.46	1	1	0	5	21	0
0.00	1	3	0	5	54	0

Model 2

(GT) covid
(GT) covid-19
(GT) chest pain
(GT) covid symptoms
(GT) Ageusia
(GT) Anosmia

β	tp	fp	fn	tp	fp	fn
1.00	0	0	2	0	0	4
0.99	0	0	2	0	0	4
0.96	0	0	2	2	0	2
0.91	2	0	0	4	0	0
0.76	2	1	0	4	4	0
0.46	2	2	0	4	15	0
0.00	2	3	0	4	44	0

Model 3

(GT) covid
(GT) covid-19
(GT) covid symptoms
(GT) Ageusia
(GT) Anosmia
(GT) Asthma

β	tp	fp	fn	tp	fp	fn
1.00	0	0	3	0	0	3
0.99	0	0	3	0	0	3
0.96	1	0	2	2	0	1
0.91	3	0	0	3	0	0
0.76	3	1	0	3	3	0
0.46	3	4	0	3	11	0
0.00	3	5	0	3	33	0

Proxies selected by the Multiple Sources method

Model 1

(GT) covid
(GT) covid-19
(GT) covid symptoms
(GT) chest tightness
(GT) fever
(GT) Acute bronchitis

Model 2

(GT) covid
(GT) covid-19
(GT) covid symptoms
(GT) fever
(GT) Acute bronchitis
(GT) Anosmia

Model 3

(GT) covid symptoms
(GT) Acute bronchitis
(GT) Anosmia
(GT) Bronchitis
(GT) Cough
(GT) Phlegm

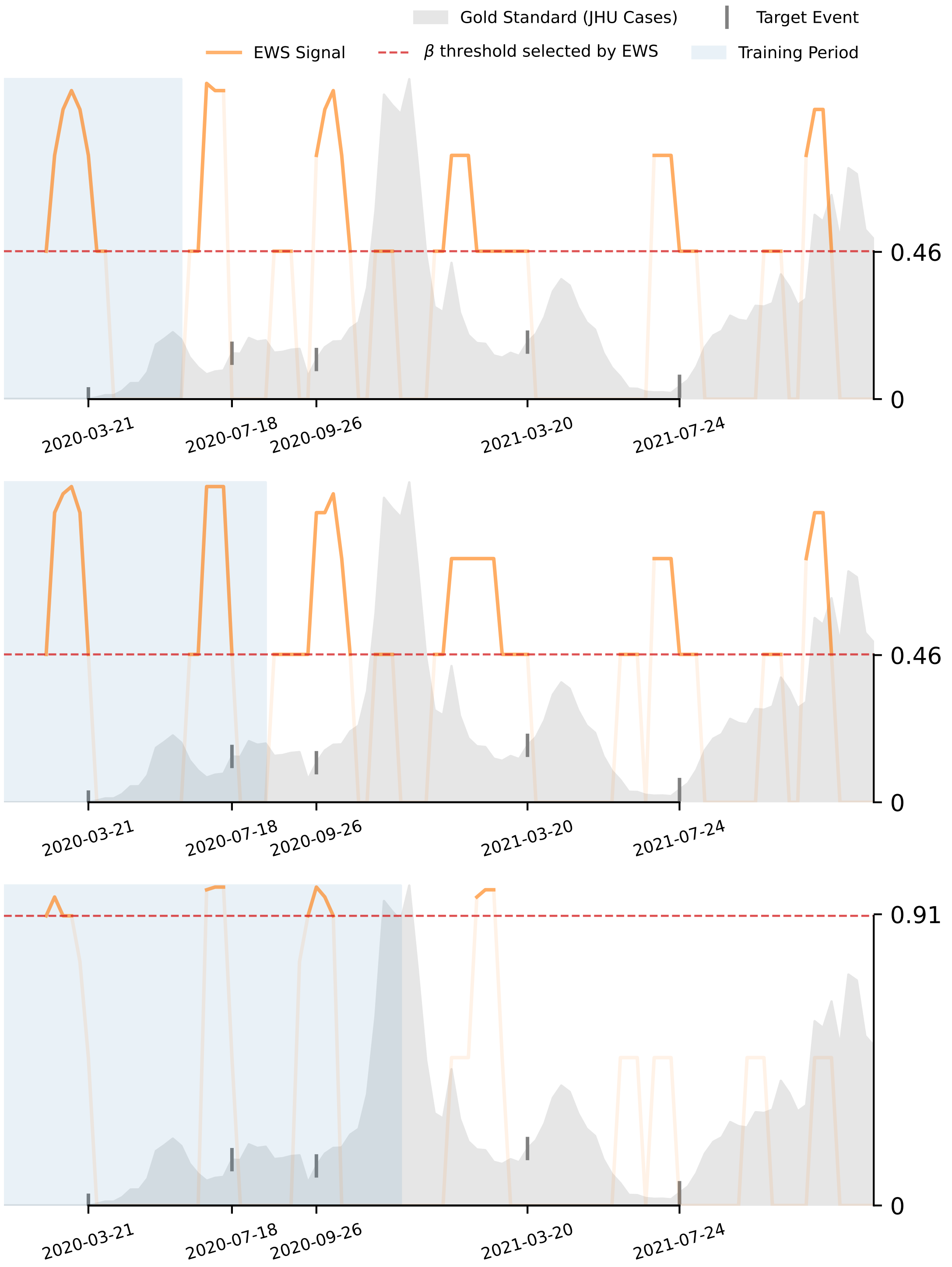

Performance as a function of β

Model 1

	Training			Out of sample		
β	tp	fp	fn	tp	fp	fn
1.00	0	0	1	0	0	4
0.99	0	0	1	1	0	3
0.96	0	0	1	1	1	2
0.91	1	0	0	1	4	2
0.76	1	0	0	3	9	1
0.46	1	0	0	4	21	0
0.00	1	3	0	4	55	0

Model 2

	Training			Out of sample		
β	tp	fp	fn	tp	fp	fn
1.00	0	0	2	0	0	3
0.99	0	0	2	0	0	3
0.96	2	0	0	0	1	2
0.91	2	0	0	1	4	2
0.76	2	0	0	3	9	0
0.46	2	0	0	3	22	0
0.00	2	2	0	3	52	0

Model 3

	Training			Out of sample		
β	tp	fp	fn	tp	fp	fn
1.00	0	0	3	0	0	2
0.99	2	0	1	1	0	1
0.96	2	0	1	1	0	1
0.91	3	1	0	1	0	1
0.76	3	3	0	1	0	1
0.46	3	3	0	2	10	0
0.00	3	3	0	2	42	0

Proxies selected by the Multiple Sources method

Model 1

(GT) covid-19 who
(GT) covid
(GT) covid-19
(GT) covid symptoms
(GT) Bronchitis
(GT) Cardiac arrest

Model 2

(GT) covid-19
(GT) covid

Model 3

(GT) covid-19
(GT) covid
(GT) Cough
(GT) Fever
(GT) Otitis
(GT) Sore throat

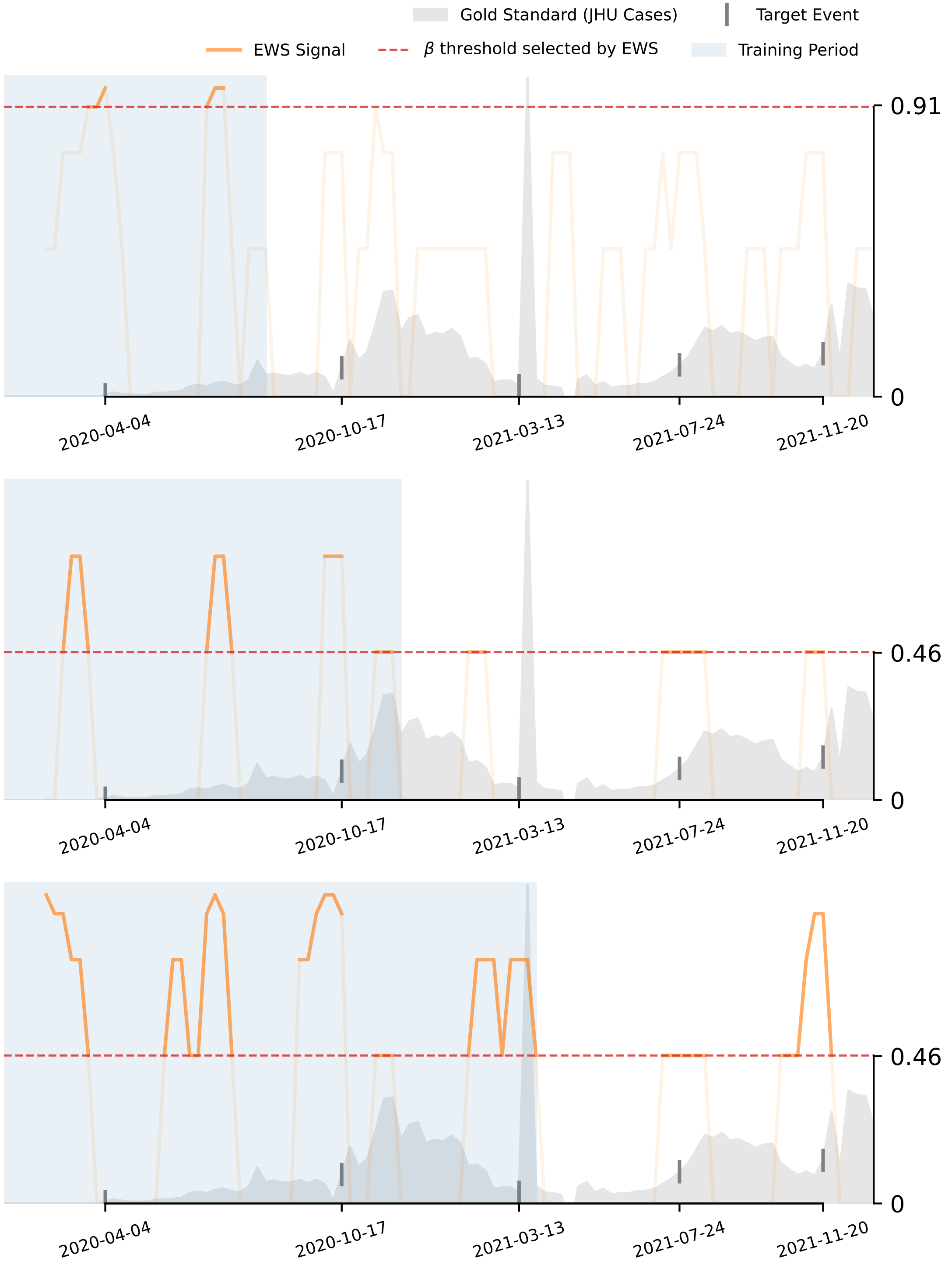

Performance as a function of β

Model 1

β	Training tp	Training fp	Training fn	Out of sample tp	Out of sample fp	Out of sample fn
1.00	0	0	1	0	0	4
0.99	0	0	1	0	0	4
0.96	0	0	1	0	0	4
0.91	1	2	0	0	1	4
0.76	1	3	0	3	8	1
0.46	1	5	0	4	26	0
0.00	1	12	0	4	44	0

Model 2

β	Training tp	Training fp	Training fn	Out of sample tp	Out of sample fp	Out of sample fn
0.76	0	0	2	0	0	3
0.46	2	2	0	3	3	0
0.00	2	8	0	3	35	0

Model 3

β	Training tp	Training fp	Training fn	Out of sample tp	Out of sample fp	Out of sample fn
1.00	0	0	3	0	0	2
0.99	0	0	3	0	0	2
0.96	0	0	3	0	0	2
0.91	1	2	2	1	0	1
0.76	1	6	2	1	0	1
0.46	3	11	0	2	4	0
0.00	3	20	0	2	26	0

Proxies selected by the Multiple Sources method

Model 1

(GT) covid
(GT) covid-19
(GT) chest pain
(GT) covid symptoms
(GT) covid-19 who
(GT) Acute bronchitis

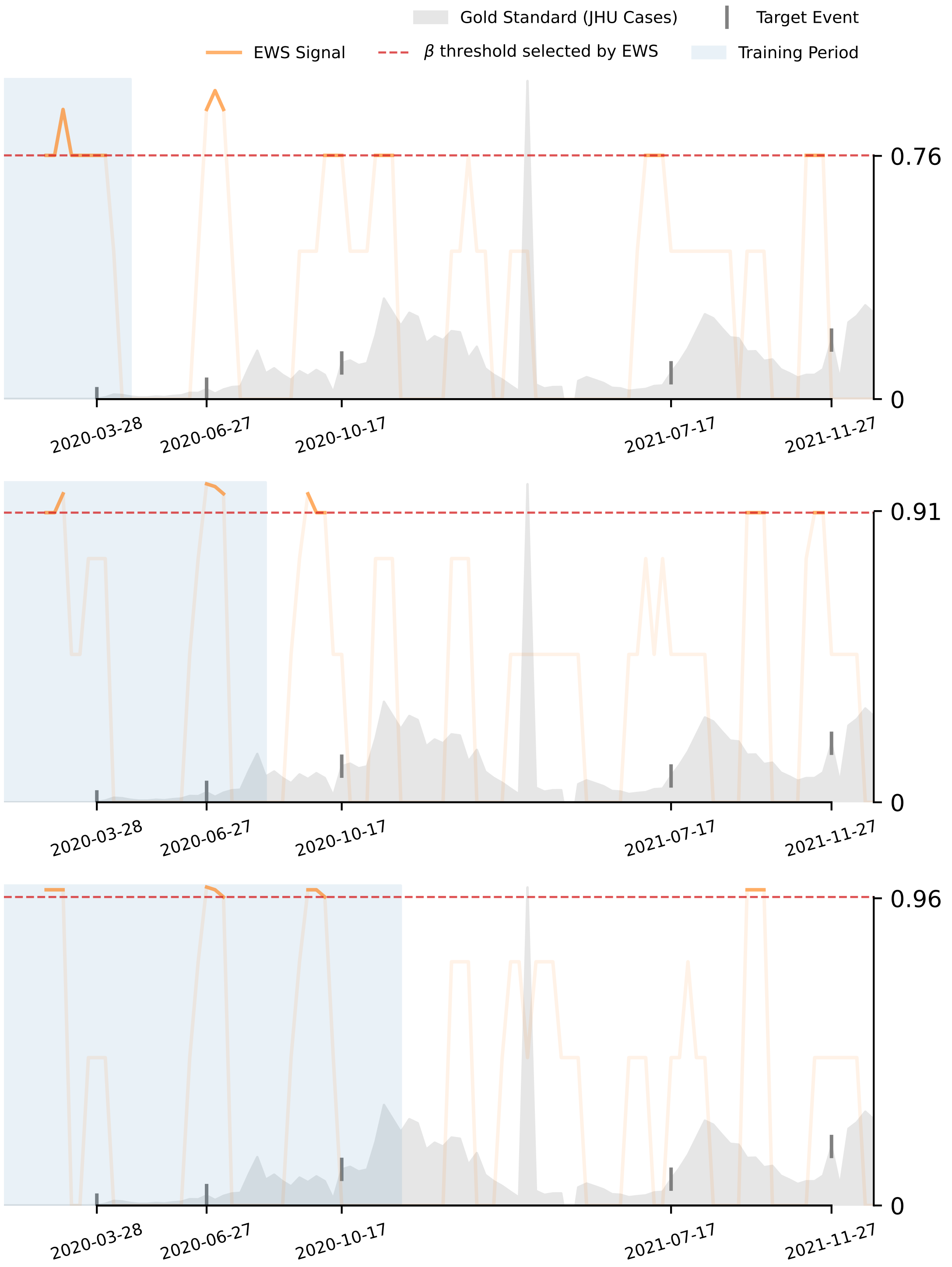

Performance as a function of β

Model 1

β	Training tp	Training fp	Training fn	Out of sample tp	Out of sample fp	Out of sample fn
1.00	0	0	1	0	0	4
0.99	0	0	1	0	0	4
0.96	0	0	1	0	1	3
0.91	0	0	1	1	2	3
0.76	1	0	0	4	6	0
0.46	1	3	0	4	27	0
0.00	1	4	0	4	60	0

Model 2

(GT) covid
(GT) covid symptoms
(GT) Acute bronchitis
(GT) Asthma
(GT) Bronchitis
(GT) Chest pain

β	Training tp	Training fp	Training fn	Out of sample tp	Out of sample fp	Out of sample fn
1.00	0	0	2	0	0	3
0.99	1	0	1	0	0	3
0.96	1	1	1	1	0	2
0.91	2	2	0	2	3	1
0.76	2	4	0	3	9	0
0.46	2	5	0	3	25	0
0.00	2	5	0	3	51	0

Model 3

(GT) Acute bronchitis
(GT) Asthma
(GT) Bronchitis
(GT) Chest pain
(GT) Cough
(GT) Phlegm

β	Training tp	Training fp	Training fn	Out of sample tp	Out of sample fp	Out of sample fn
1.00	0	0	3	0	0	2
0.99	1	0	2	0	3	2
0.96	3	3	0	0	3	2
0.91	3	4	0	0	3	2
0.76	3	4	0	0	12	1
0.46	3	5	0	2	23	0
0.00	3	7	0	2	42	0

MO, St. Louis

Proxies selected by the Multiple Sources method

Model 1

(GT) covid
(GT) how long does covid last
(GT) covid-19
(GT) chest pain
(GT) covid symptoms
(GT) Ageusia

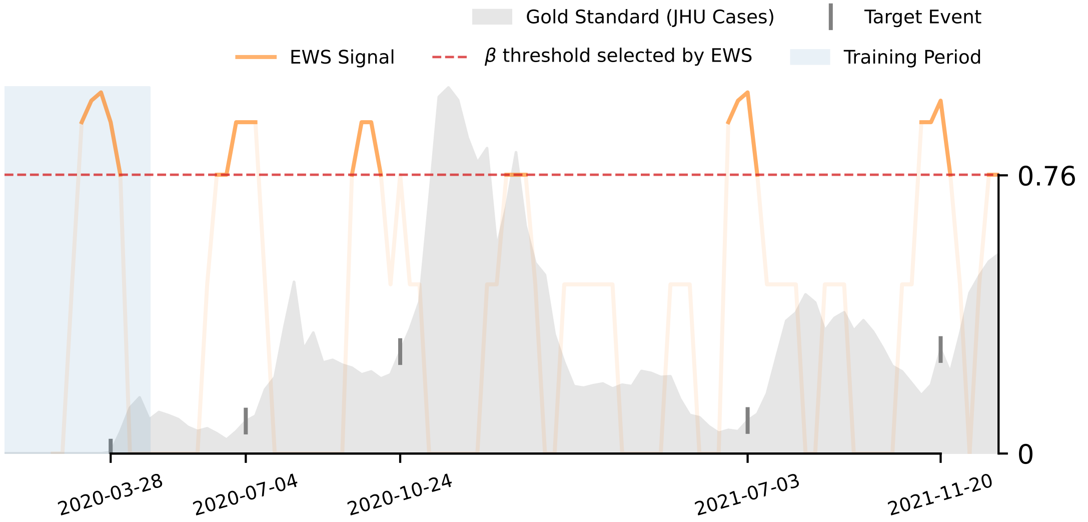

Performance as a function of β

β	Training tp	Training fp	Training fn	Out of sample tp	Out of sample fp	Out of sample fn
1.00	0	0	1	0	0	4
0.99	0	0	1	1	0	3
0.96	1	0	0	2	0	2
0.91	1	0	0	4	1	0
0.76	1	0	0	4	8	0
0.46	1	1	0	4	31	0
0.00	1	1	0	4	60	0

Model 2

(GT) covid
(GT) covid-19
(GT) chest pain
(GT) covid symptoms
(GT) Ageusia
(GT) Phlegm

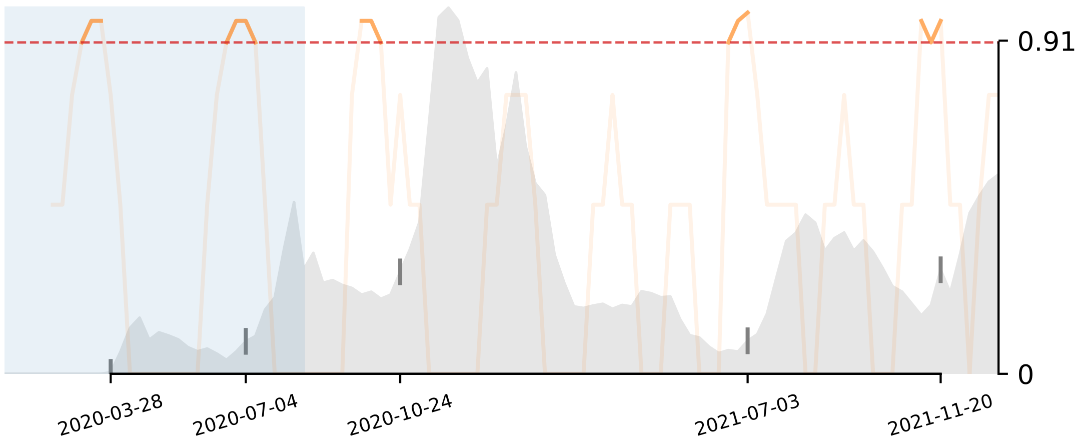

β	Training tp	Training fp	Training fn	Out of sample tp	Out of sample fp	Out of sample fn
1.00	0	0	2	0	0	3
0.99	0	0	2	1	0	2
0.96	0	0	2	3	0	0
0.91	2	0	0	3	0	0
0.76	2	1	0	3	8	0
0.46	2	1	0	3	30	0
0.00	2	5	0	3	51	0

Model 3

(GT) covid
(GT) covid-19
(GT) chest pain
(GT) covid symptoms
(GT) Ageusia
(GT) Phlegm

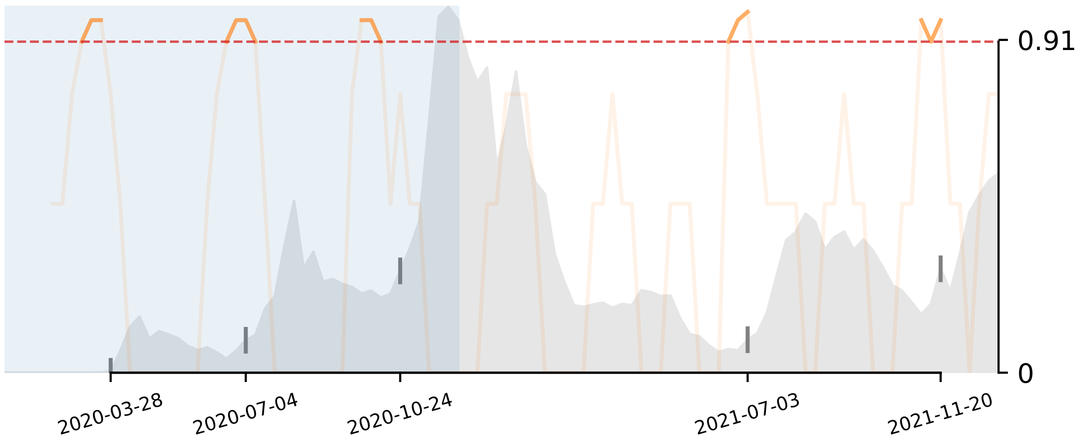

β	Training tp	Training fp	Training fn	Out of sample tp	Out of sample fp	Out of sample fn
1.00	0	0	3	0	0	2
0.99	0	0	3	1	0	1
0.96	0	0	3	2	0	0
0.91	3	0	0	2	0	0
0.76	3	1	0	2	8	0
0.46	3	2	0	2	28	0
0.00	3	8	0	2	42	0

Proxies selected by the Multiple Sources method

Model 1

(GT) covid
(GT) covid-19
(GT) covid symptoms
(GT) Chest pain
(GT) Dizziness
(GT) Fatigue

Model 2

(GT) covid-19
(GT) covid symptoms
(GT) Abdominal pain
(GT) Chest pain
(GT) Fatigue
(GT) Pneumonia

Model 3

(GT) Pneumonia
up2date
(GT) covid-19
(GT) Abdominal pain
(GT) covid
(GT) Chest pain

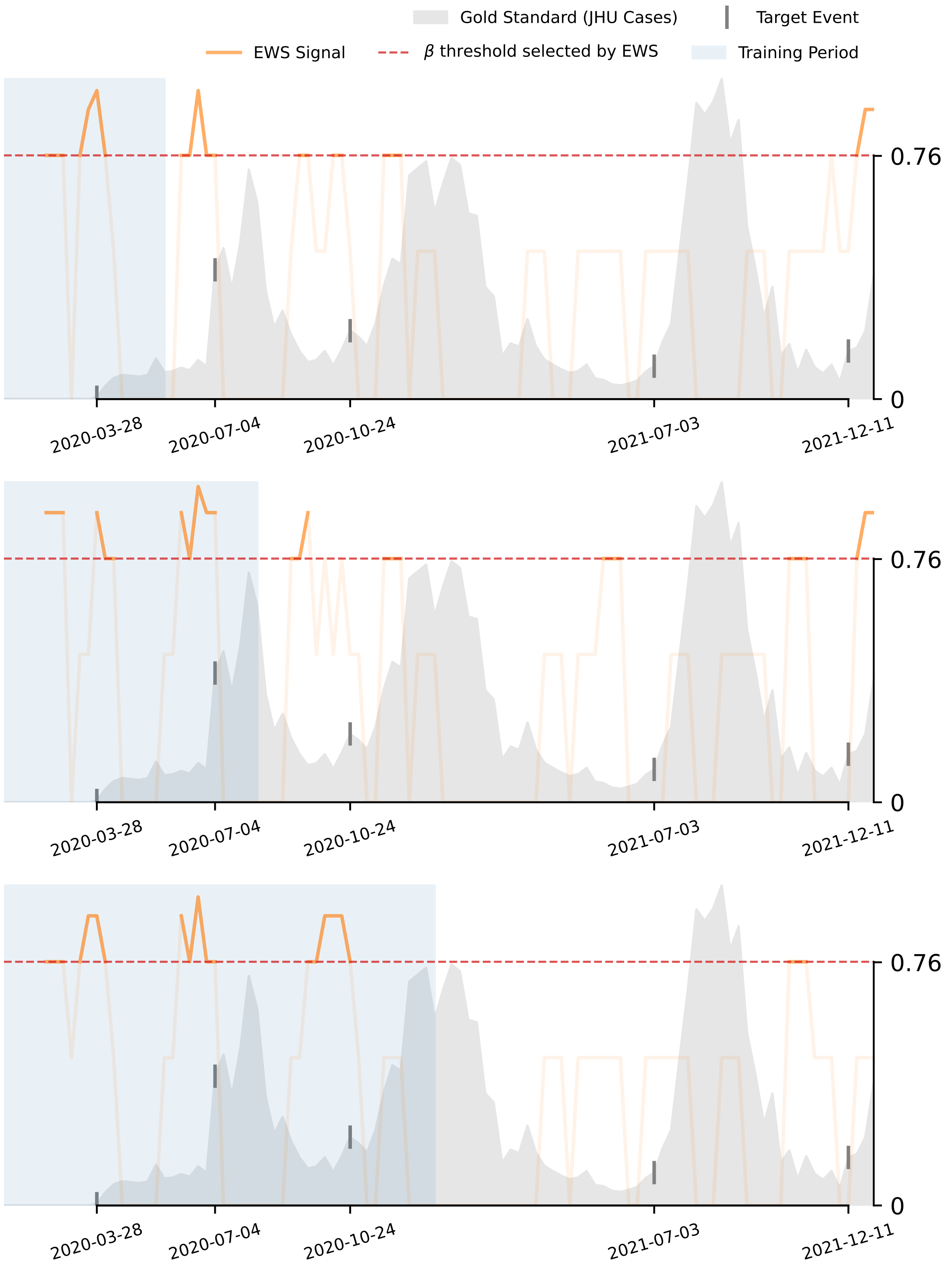

Performance as a function of β

Model 1

β	Training tp	Training fp	Training fn	Out of sample tp	Out of sample fp	Out of sample fn
1.00	0	0	1	0	0	4
0.99	0	0	1	0	0	4
0.96	0	0	1	1	0	3
0.91	1	0	0	1	2	2
0.76	1	0	0	3	6	1
0.46	1	3	0	4	24	0
0.00	1	4	0	4	57	0

Model 2

β	Training tp	Training fp	Training fn	Out of sample tp	Out of sample fp	Out of sample fn
1.00	0	0	2	0	0	3
0.99	0	0	2	0	0	3
0.96	1	0	1	0	0	3
0.91	1	0	1	1	2	1
0.76	2	2	0	3	8	0
0.46	2	4	0	3	27	0
0.00	2	6	0	3	52	0

Model 3

β	Training tp	Training fp	Training fn	Out of sample tp	Out of sample fp	Out of sample fn
1.00	0	0	3	0	0	2
0.99	0	0	3	0	0	2
0.96	0	0	3	0	0	2
0.91	1	0	2	0	0	2
0.76	3	0	0	1	1	1
0.46	3	4	0	2	17	0
0.00	3	13	0	2	38	0

Proxies selected by the Multiple Sources method

Model 1

(GT) chest tightness
(GT) covid-19 who
(GT) covid
(GT) loss smell
(GT) covid-19
(GT) covid symptoms

Model 2

(GT) covid
(GT) how long does covid last
(GT) covid-19

Model 3

(GT) covid
(GT) how long does covid last
(GT) covid-19
(GT) covid symptoms
(GT) Vomiting
(GT) Fever

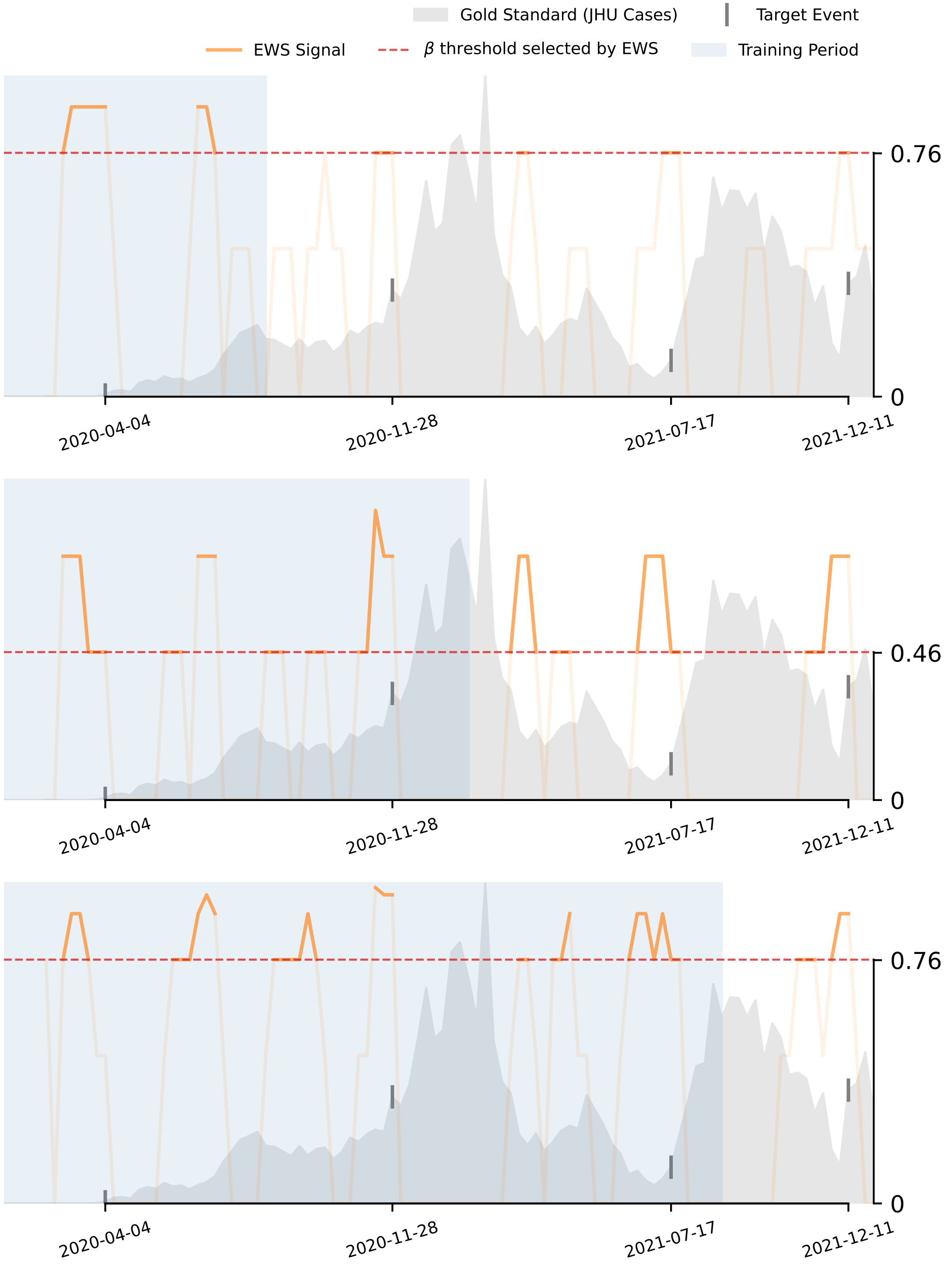

Performance as a function of β

Model 1

β	Training tp	Training fp	Training fn	Out of sample tp	Out of sample fp	Out of sample fn
1.00	0	0	1	0	0	3
0.99	0	0	1	0	0	3
0.96	0	0	1	0	0	3
0.91	0	0	1	0	0	3
0.76	1	2	0	3	4	0
0.46	1	4	0	3	21	0
0.00	1	9	0	3	51	0

Model 2

β	Training tp	Training fp	Training fn	Out of sample tp	Out of sample fp	Out of sample fn
0.91	0	0	2	0	0	2
0.76	1	0	1	2	2	0
0.46	2	4	0	2	8	0
0.00	2	13	0	2	34	0

Model 3

β	Training tp	Training fp	Training fn	Out of sample tp	Out of sample fp	Out of sample fn
1.00	0	0	3	0	0	1
0.99	0	0	3	0	0	1
0.96	1	0	2	0	0	1
0.91	1	1	2	1	0	0
0.76	3	5	0	1	0	0
0.46	3	21	0	1	3	0
0.00	3	30	0	1	11	0

NC, Forsyth

100

Proxies selected by the Multiple Sources method

Model 1

(GT) covid
(GT) Bronchitis
(GT) Throat irritation
(GT) Wheeze
(GT) covid-19
(GT) covid symptoms

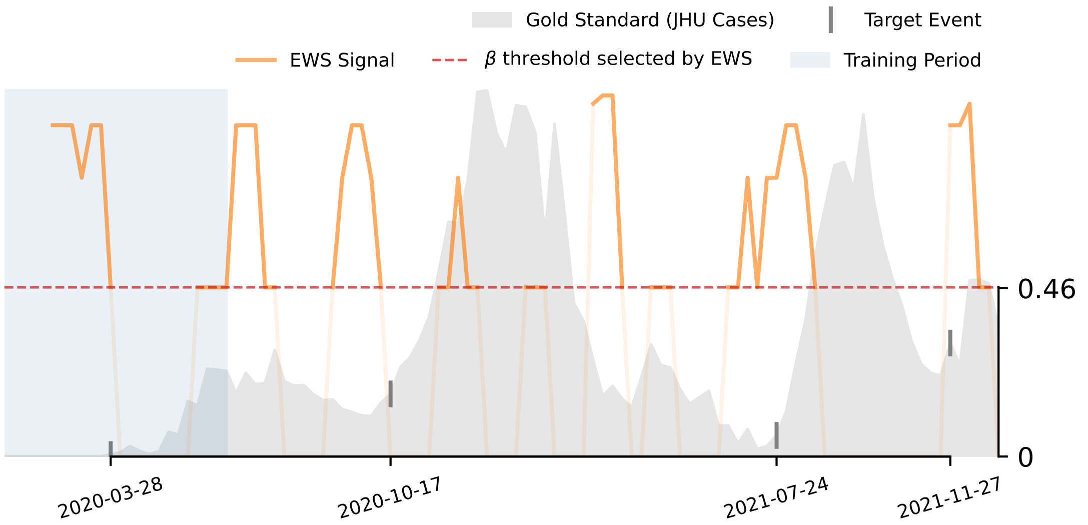

Performance as a function of β

β	Training tp	Training fp	Training fn	Out of sample tp	Out of sample fp	Out of sample fn
1.00	0	0	1	0	0	3
0.99	0	0	1	0	2	3
0.96	0	0	1	0	4	2
0.91	0	0	1	2	10	0
0.76	1	2	0	3	12	0
0.46	1	2	0	3	28	0
0.00	1	6	0	3	59	0

Model 2

(GT) Throat irritation
(GT) covid
(GT) covid symptoms
(GT) Abdominal pain
(GT) Asthma
(GT) Dizziness

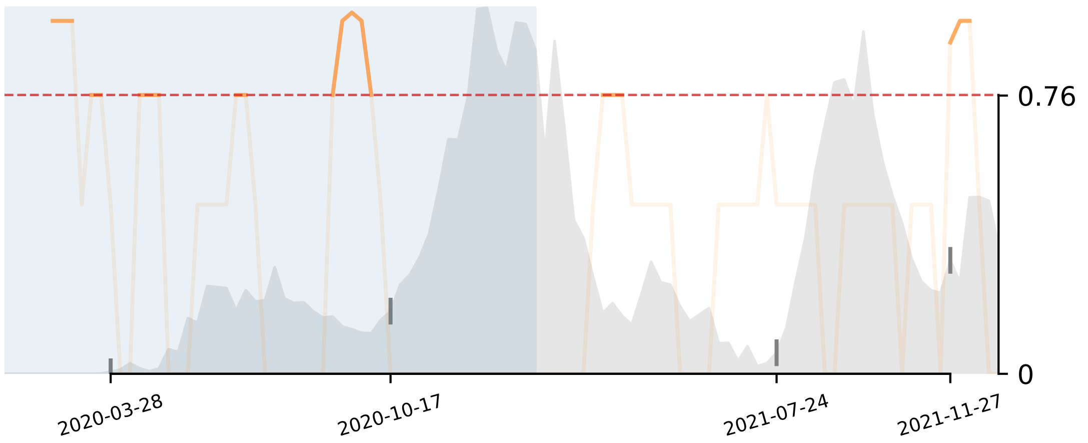

β	Training tp	Training fp	Training fn	Out of sample tp	Out of sample fp	Out of sample fn
1.00	0	0	2	0	0	2
0.99	0	0	2	0	0	2
0.96	1	0	1	0	2	1
0.91	2	3	0	1	2	1
0.76	2	3	0	2	5	0
0.46	2	9	0	2	21	0
0.00	2	14	0	2	34	0

Model 3

(GT) Asthma
(GT) covid
(GT) covid symptoms
(GT) quarantine
(GT) Abdominal pain
(GT) Dizziness

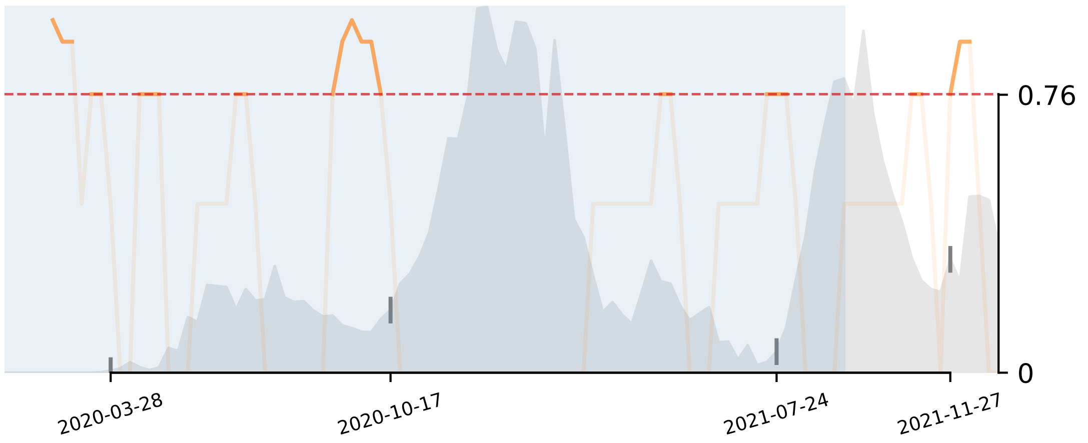

β	Training tp	Training fp	Training fn	Out of sample tp	Out of sample fp	Out of sample fn
1.00	0	0	3	0	0	1
0.99	0	0	3	0	0	1
0.96	0	0	3	0	0	1
0.91	1	1	2	0	2	0
0.76	2	3	1	1	2	0
0.46	3	12	0	1	7	0
0.00	3	29	0	1	9	0

NC, Johnston

Proxies selected by the Multiple Sources method

Model 1

(GT) chest tightness
(GT) covid-19 who
(GT) covid
(GT) how long does covid last
(GT) loss smell
(GT) covid-19

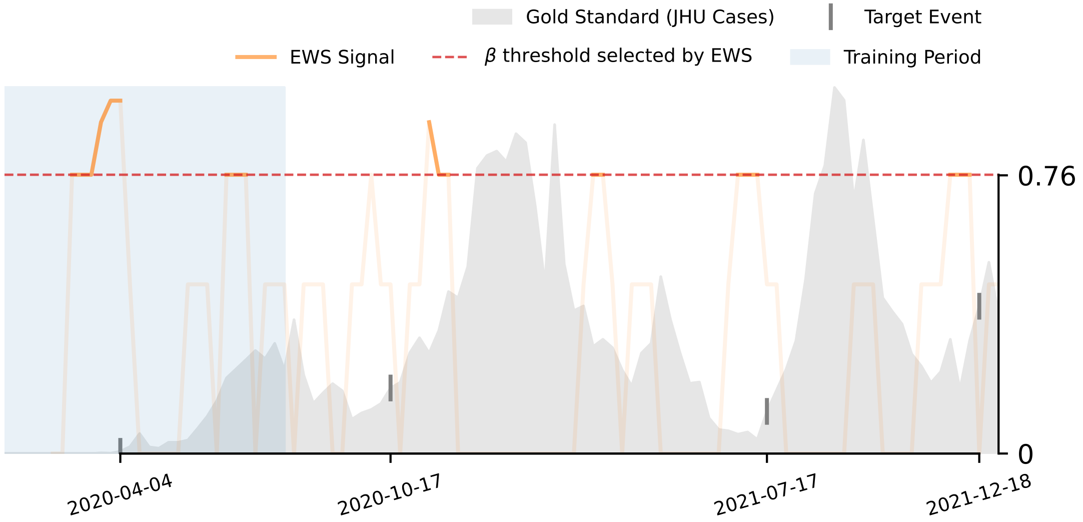

Performance as a function of β

β	Training tp	Training fp	Training fn	Out of sample tp	Out of sample fp	Out of sample fn
1.00	0	0	1	0	0	3
0.99	0	0	1	0	0	3
0.96	0	0	1	0	0	3
0.91	1	0	0	0	1	3
0.76	1	0	0	3	5	0
0.46	1	4	0	3	21	0
0.00	1	11	0	3	53	0

Model 2

(GT) covid-19 who
(GT) covid-19
(GT) fever
(GT) Diarrhea
(GT) Chest pain

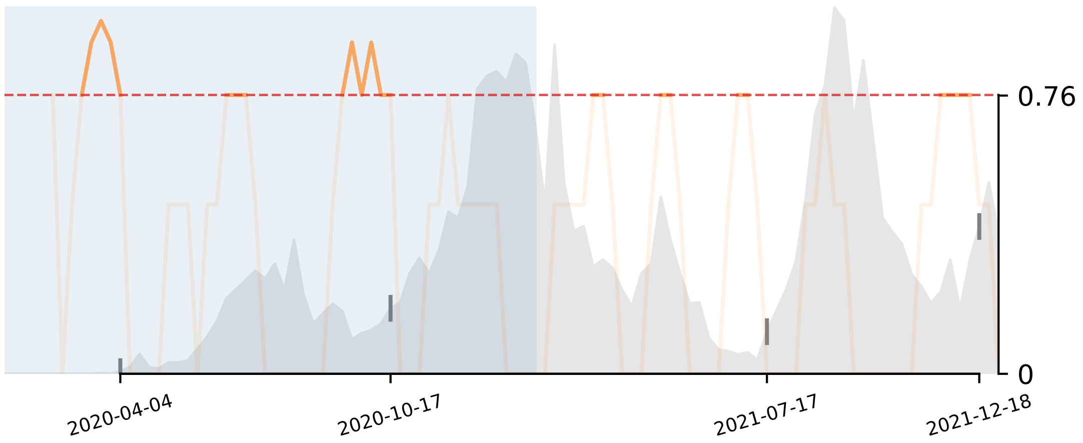

β	Training tp	Training fp	Training fn	Out of sample tp	Out of sample fp	Out of sample fn
0.99	0	0	2	0	0	2
0.96	0	0	2	0	0	2
0.91	1	0	1	0	0	2
0.76	2	0	0	2	5	0
0.46	2	6	0	2	17	0
0.00	2	21	0	2	34	0

Model 3

(GT) covid-19
(GT) fever
(GT) covid-19 who
(GT) Shortness of breath
(GT) covid
(GT) covid symptoms

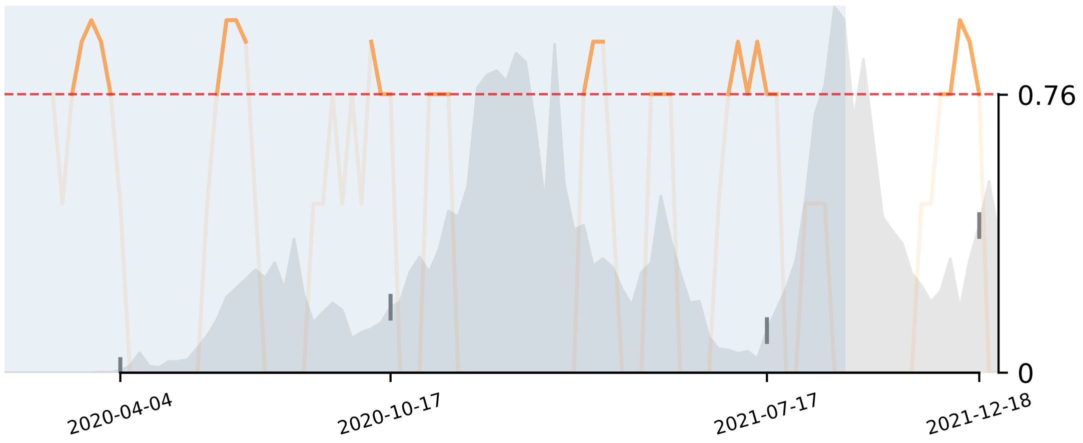

β	Training tp	Training fp	Training fn	Out of sample tp	Out of sample fp	Out of sample fn
1.00	0	0	3	0	0	1
0.99	0	0	3	0	0	1
0.96	0	0	3	1	0	0
0.91	1	2	2	1	0	0
0.76	3	5	0	1	0	0
0.46	3	17	0	1	0	0
0.00	3	28	0	1	9	0

NC, Mecklenburg

Proxies selected by the Multiple Sources method

Model 1

(GT) covid
(GT) covid-19
(GT) covid symptoms
(GT) quarantine
(GT) Burning Chest Pain
(GT) Chronic pain

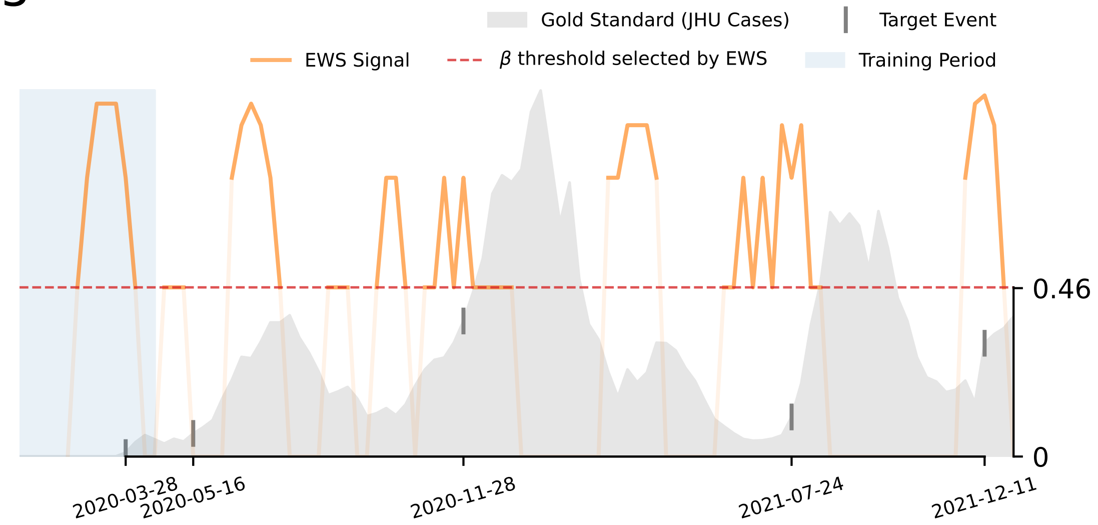

Performance as a function of β

β	Training tp	Training fp	Training fn	Out of sample tp	Out of sample fp	Out of sample fn
1.00	0	0	1	0	0	4
0.99	0	0	1	1	0	3
0.96	0	0	1	1	1	3
0.91	1	0	0	2	8	2
0.76	1	0	0	3	15	1
0.46	1	0	0	4	29	0
0.00	1	2	0	4	64	0

Model 2

(GT) Chronic pain
Apple Mobility (driving)
Apple Mobility (walking)
Apple Mobility (transit)

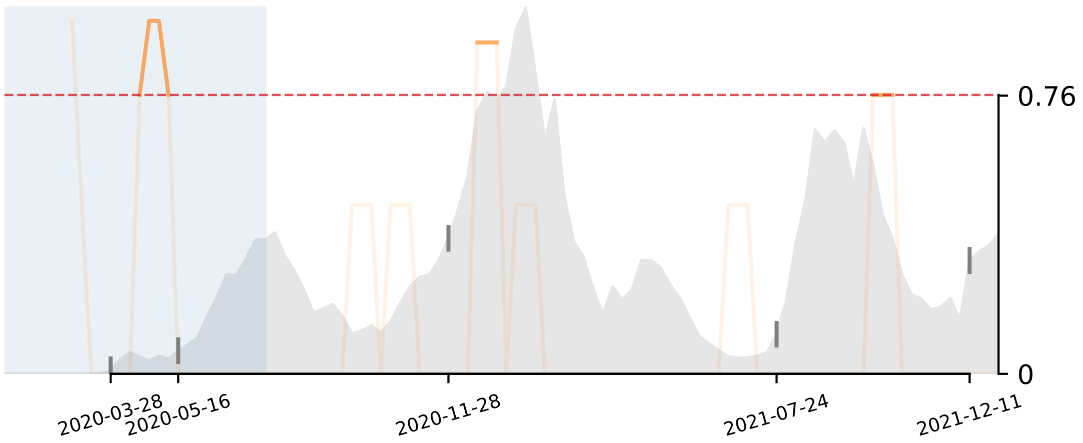

β	Training tp	Training fp	Training fn	Out of sample tp	Out of sample fp	Out of sample fn
0.96	0	0	2	0	0	3
0.91	2	0	0	0	3	3
0.76	2	0	0	0	6	3
0.46	2	0	0	2	12	1
0.00	2	0	0	3	55	0

Model 3

Apple Mobility (walking)
(GT) covid
(GT) loss smell
(GT) quarantine
(GT) Abdominal pain
(GT) Chronic pain

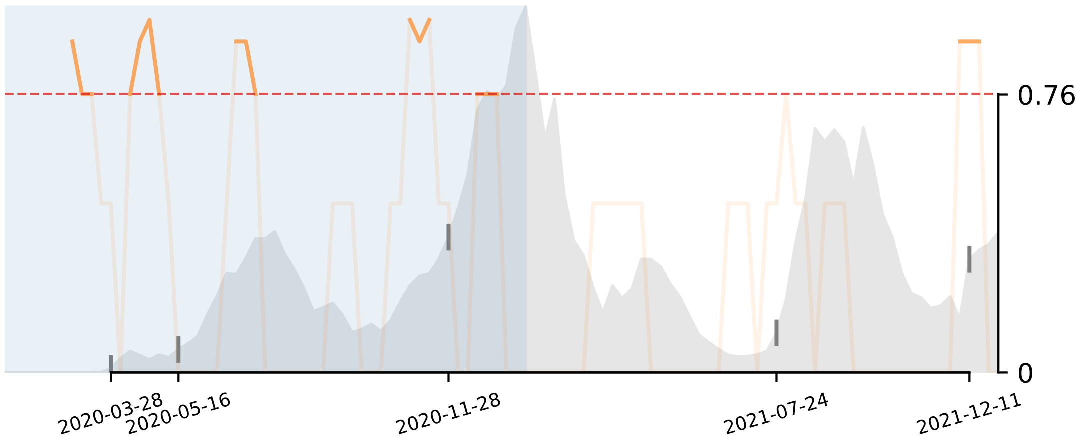

β	Training tp	Training fp	Training fn	Out of sample tp	Out of sample fp	Out of sample fn
1.00	0	0	3	0	0	2
0.99	0	0	3	0	0	2
0.96	0	0	3	0	0	2
0.91	2	0	1	1	1	1
0.76	3	2	0	1	2	0
0.46	3	7	0	2	13	0
0.00	3	13	0	2	35	0

Proxies selected by the Multiple Sources method

Model 1

(GT) chest tightness
(GT) covid-19 who
(GT) Hyperventilation
(GT) covid
(GT) how long does covid last
(GT) covid-19

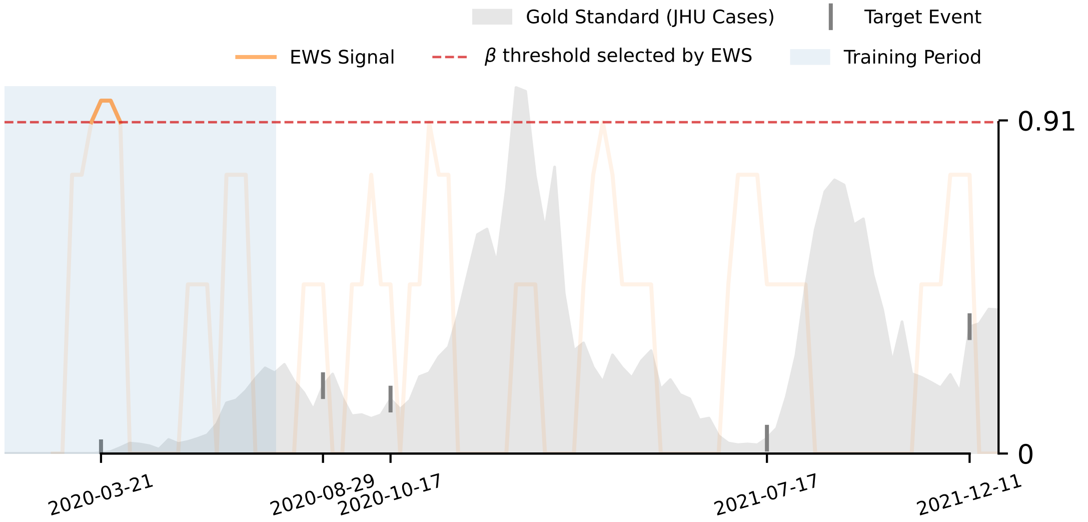

Performance as a function of β

β	Training tp	Training fp	Training fn	Out of sample tp	Out of sample fp	Out of sample fn
1.00	0	0	1	0	0	4
0.99	0	0	1	0	0	4
0.96	0	0	1	0	0	4
0.91	1	1	0	0	2	4
0.76	1	2	0	3	6	1
0.46	1	5	0	4	20	0
0.00	1	8	0	4	49	0

Model 2

(GT) how long does covid last
(GT) chest pain
(GT) covid symptoms
(GT) Burning Chest Pain
(GT) Chills

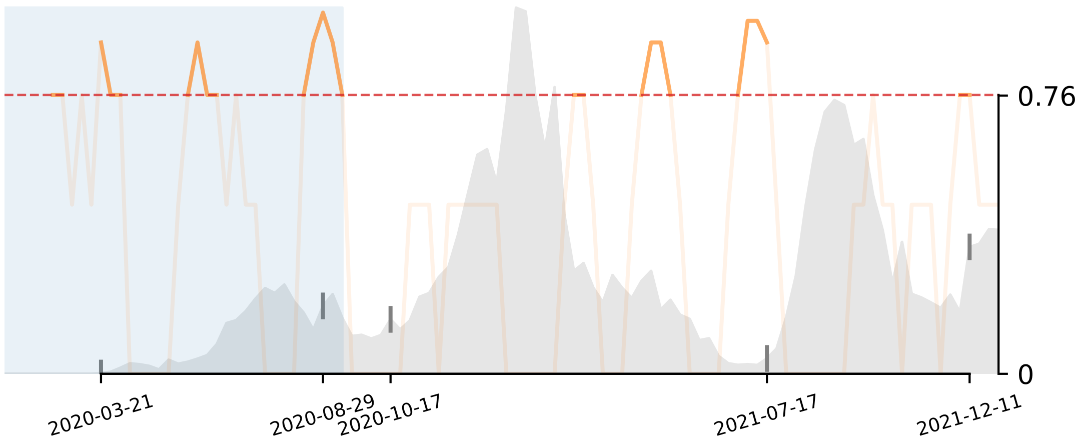

β	Training tp	Training fp	Training fn	Out of sample tp	Out of sample fp	Out of sample fn
0.99	0	0	2	0	0	3
0.96	1	0	1	1	0	2
0.91	1	0	1	1	2	2
0.76	2	2	0	2	7	1
0.46	2	10	0	2	28	0
0.00	2	14	0	3	49	0

Model 3

(GT) covid-19 who
(GT) covid symptoms
(GT) Acute bronchitis
(GT) Ageusia
(GT) Nasal congestion
(GT) Phlegm

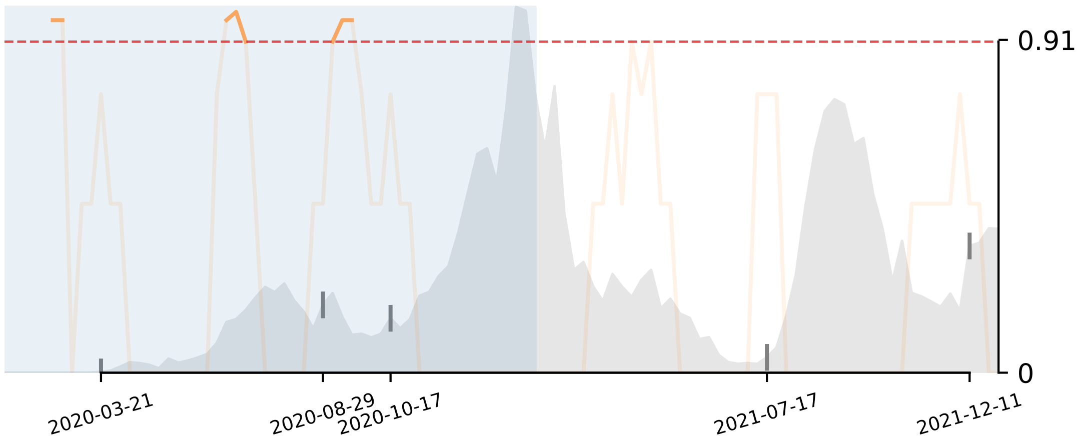

β	Training tp	Training fp	Training fn	Out of sample tp	Out of sample fp	Out of sample fn
1.00	0	0	3	0	0	2
0.99	0	0	3	0	0	2
0.96	0	1	3	0	0	2
0.91	2	4	1	0	2	2
0.76	2	6	1	2	5	0
0.46	2	7	1	2	11	0
0.00	3	12	0	2	34	0

Proxies selected by the Multiple Sources method

Model 1

(GT) covid
(GT) how long does covid last
(GT) loss smell
(GT) covid-19
(GT) loss taste
(GT) chest pain

Model 2

(GT) how long does covid last
(GT) nose bleed
(GT) loss smell
(GT) covid-19
(GT) covid symptoms
(GT) sars-cov-2

Model 3

(GT) covid
(GT) covid symptoms
(GT) Ageusia
(GT) chest pain
(GT) Chest pain
(GT) Hyperventilation

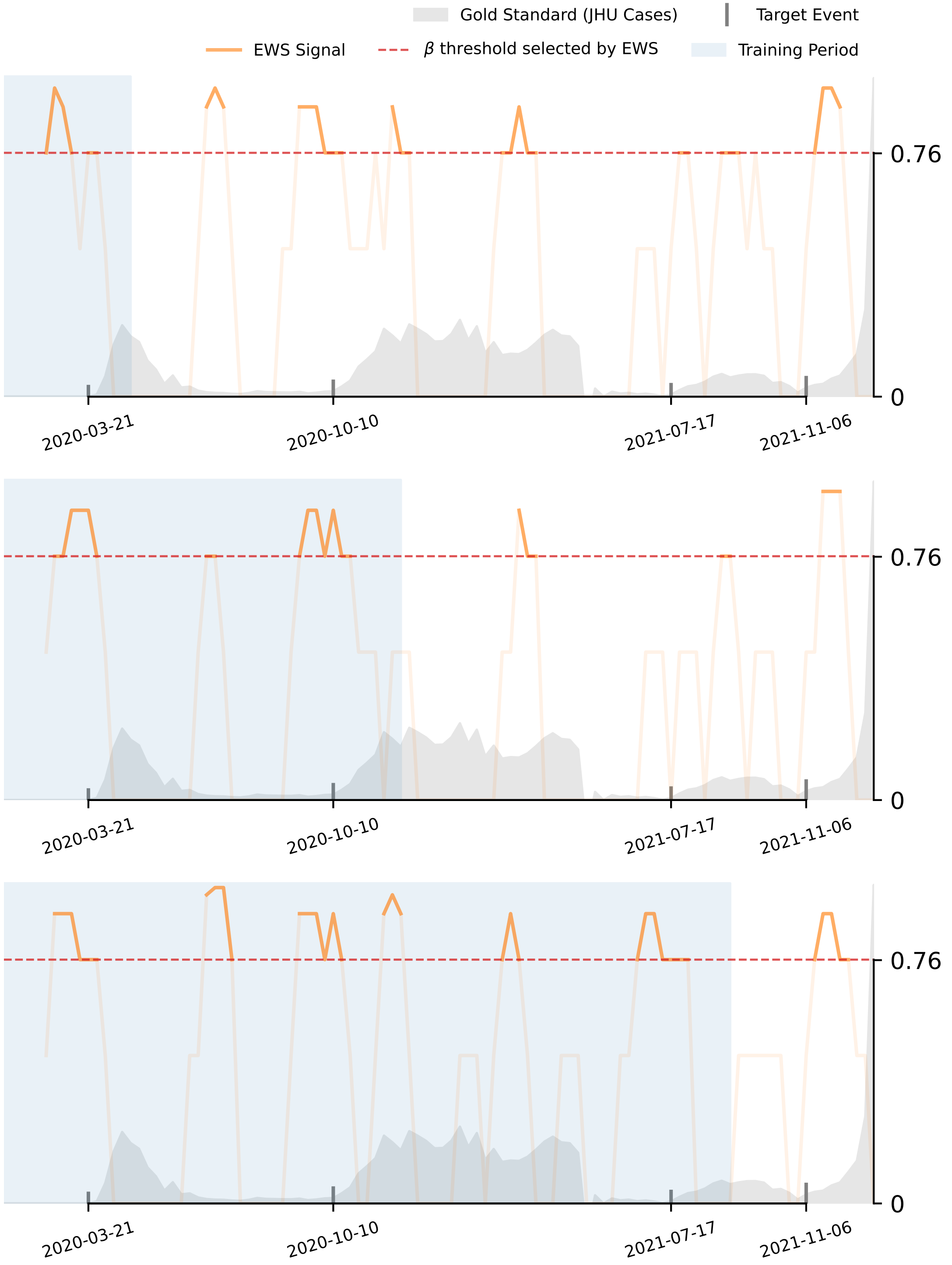

Performance as a function of β

Model 1

β	Training tp	Training fp	Training fn	Out of sample tp	Out of sample fp	Out of sample fn
1.00	0	0	1	0	0	3
0.99	0	0	1	0	0	3
0.96	0	0	1	0	3	2
0.91	1	0	0	1	8	1
0.76	1	0	0	2	22	0
0.46	1	2	0	3	33	0
0.00	1	3	0	3	67	0

Model 2

β	Training tp	Training fp	Training fn	Out of sample tp	Out of sample fp	Out of sample fn
1.00	0	0	2	0	0	2
0.99	0	0	2	0	0	2
0.96	0	0	2	0	3	1
0.91	0	0	2	0	4	1
0.76	2	0	0	0	8	1
0.46	2	5	0	2	18	0
0.00	2	15	0	2	42	0

Model 3

β	Training tp	Training fp	Training fn	Out of sample tp	Out of sample fp	Out of sample fn
1.00	0	0	3	0	0	1
0.99	0	0	3	0	0	1
0.96	0	2	3	0	0	1
0.91	0	4	3	0	2	0
0.76	3	7	0	0	5	0
0.46	3	14	0	1	9	0
0.00	3	32	0	1	10	0

NJ, Middlesex

Proxies selected by the Multiple Sources method

Model 1

(GT) covid
(GT) how long does covid last
(GT) loss smell
(GT) covid-19
(GT) loss taste
(GT) chest pain

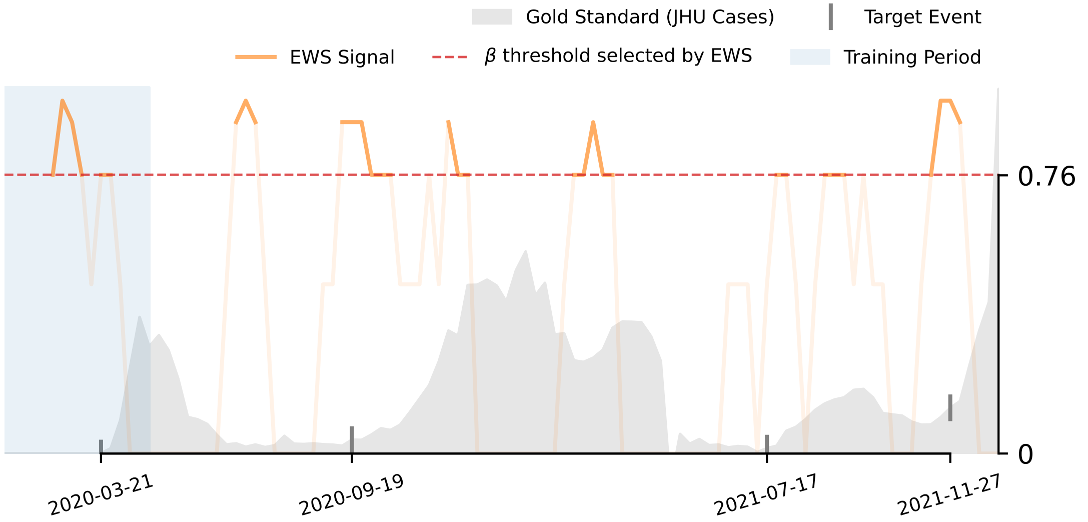

Performance as a function of β

β	Training tp	Training fp	Training fn	Out of sample tp	Out of sample fp	Out of sample fn
1.00	0	0	1	0	0	3
0.99	0	0	1	0	0	3
0.96	0	0	1	1	1	2
0.91	1	0	0	2	7	1
0.76	1	0	0	2	23	0
0.46	1	2	0	3	36	0
0.00	1	3	0	3	67	0

Model 2

(GT) how long does covid last
(GT) loss smell
(GT) sars-cov-2
(GT) Asthma
(GT) Cough
(GT) Wheeze

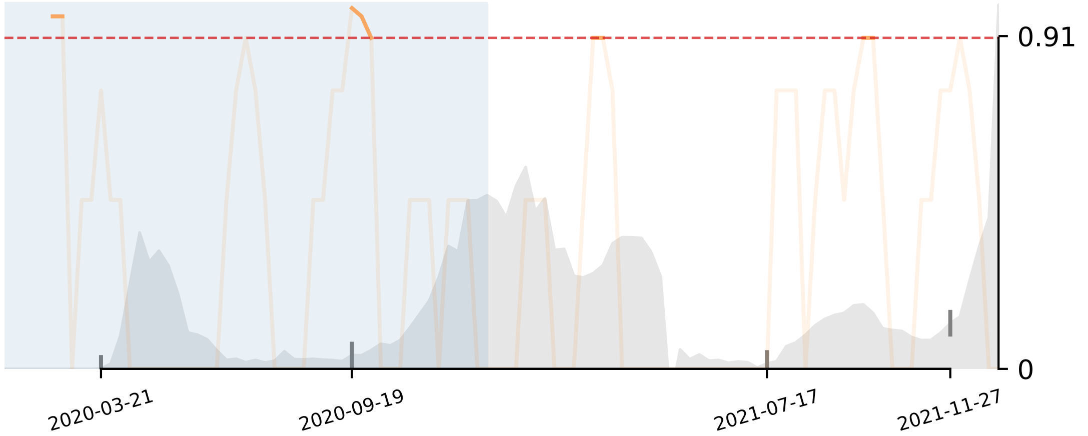

β	Training tp	Training fp	Training fn	Out of sample tp	Out of sample fp	Out of sample fn
1.00	0	0	2	0	0	2
0.99	0	0	2	0	0	2
0.96	1	0	1	0	0	2
0.91	2	2	0	0	5	1
0.76	2	4	0	1	13	0
0.46	2	6	0	1	21	0
0.00	2	16	0	2	39	0

Model 3

(GT) Chest pain
(GT) chest pain
(GT) how long does covid last
(GT) covid-19 who
(GT) sars-cov-2
(GT) Asthma

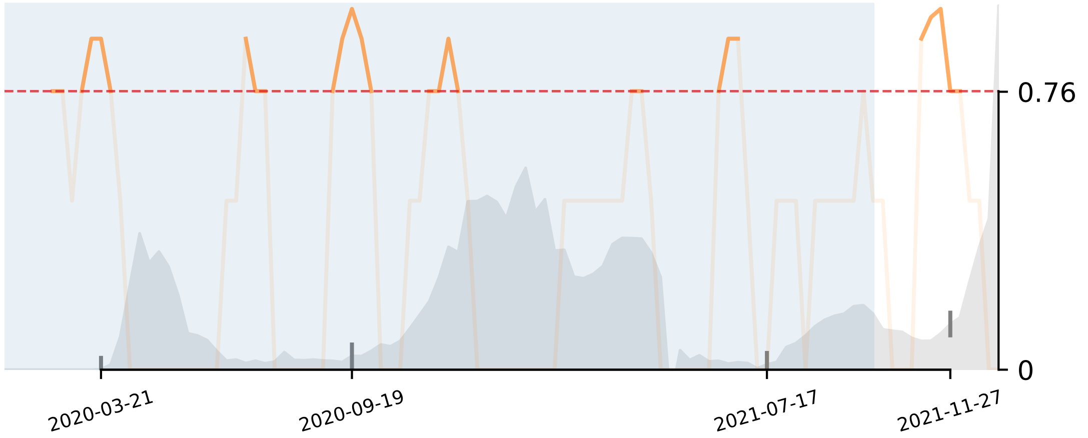

β	Training tp	Training fp	Training fn	Out of sample tp	Out of sample fp	Out of sample fn
1.00	0	0	3	0	0	1
0.99	0	0	3	1	0	0
0.96	1	0	2	1	0	0
0.91	1	0	2	1	0	0
0.76	3	3	0	1	1	0
0.46	3	15	0	1	4	0
0.00	3	38	0	1	6	0

NM, Bernalillo

Proxies selected by the Multiple Sources method

Model 1

(GT) covid
(GT) covid-19
(GT) covid symptoms
(GT) quarantine
(GT) Acute bronchitis
(GT) Bronchitis

Model 2

(GT) covid
(GT) covid-19
(GT) covid symptoms
(GT) quarantine
(GT) Acute bronchitis
(GT) Bronchitis

Model 3

(GT) Acute bronchitis
(GT) Bronchitis
(GT) Chills
(GT) Cough
(GT) Phlegm
(GT) Pneumonia

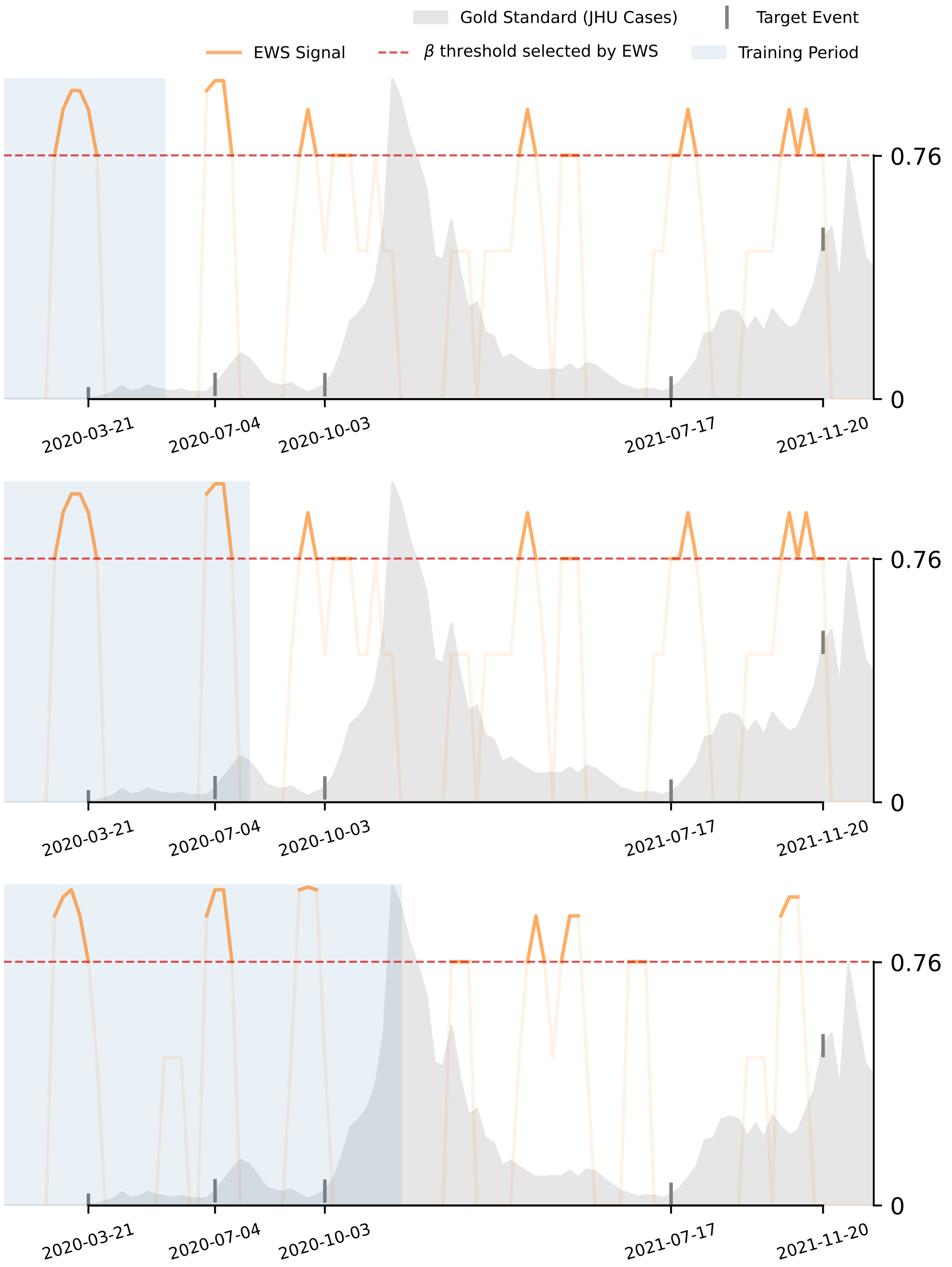

Performance as a function of β

Model 1

β	Training tp	Training fp	Training fn	Out of sample tp	Out of sample fp	Out of sample fn
1.00	0	0	1	1	1	3
0.99	0	0	1	1	1	3
0.96	0	0	1	1	1	3
0.91	1	0	0	3	3	0
0.76	1	0	0	4	15	0
0.46	1	1	0	4	31	0
0.00	1	1	0	4	57	0

Model 2

β	Training tp	Training fp	Training fn	Out of sample tp	Out of sample fp	Out of sample fn
1.00	0	0	2	0	0	3
0.99	1	1	1	0	0	3
0.96	1	1	1	0	0	3
0.91	2	1	0	2	2	0
0.76	2	1	0	3	13	0
0.46	2	3	0	3	29	0
0.00	2	3	0	3	53	0

Model 3

β	Training tp	Training fp	Training fn	Out of sample tp	Out of sample fp	Out of sample fn
1.00	0	0	3	0	0	2
0.99	1	0	2	0	0	2
0.96	3	1	0	1	0	1
0.91	3	1	0	1	3	1
0.76	3	1	0	2	9	0
0.46	3	2	0	2	15	0
0.00	3	5	0	2	42	0

NV, Clark

Proxies selected by the Multiple Sources method

Model 1

(GT) covid
(GT) covid-19
(GT) chest pain
(GT) covid symptoms
(GT) quarantine
(GT) fever

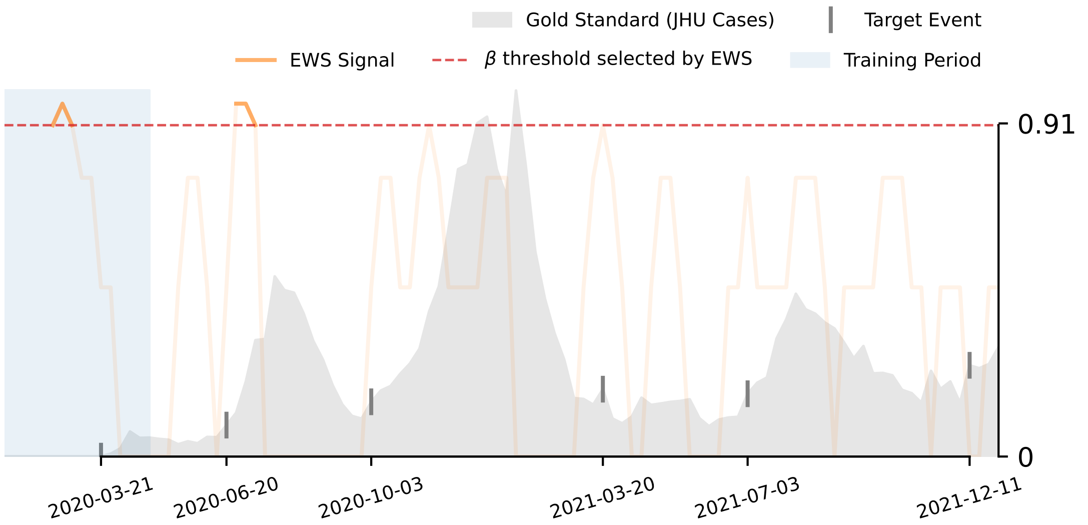

Performance as a function of β

β	Training tp	Training fp	Training fn	Out of sample tp	Out of sample fp	Out of sample fn
1.00	0	0	1	0	0	5
0.99	0	0	1	0	0	5
0.96	0	0	1	0	2	4
0.91	1	0	0	1	4	3
0.76	1	1	0	3	20	1
0.46	1	1	0	5	40	0
0.00	1	2	0	5	53	0

Model 2

(GT) covid-19
(GT) covid symptoms
(GT) fever
(GT) Ageusia
(GT) Anosmia
(GT) Burning Chest Pain

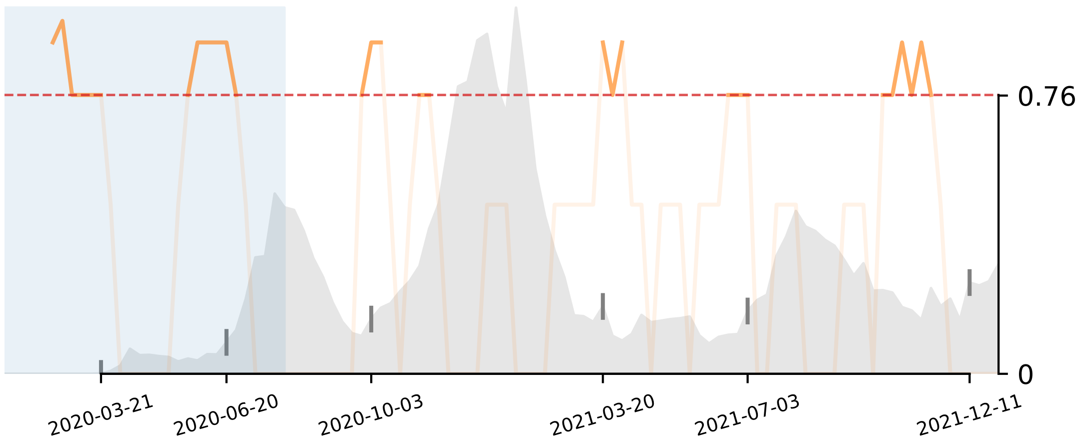

β	Training tp	Training fp	Training fn	Out of sample tp	Out of sample fp	Out of sample fn
1.00	0	0	2	0	0	4
0.99	0	0	2	0	0	4
0.96	0	0	2	0	0	4
0.91	1	0	1	3	3	1
0.76	2	1	0	4	8	0
0.46	2	2	0	4	25	0
0.00	2	5	0	4	46	0

Model 3

(GT) Ageusia
(GT) Anosmia
(GT) Burning Chest Pain
(GT) Chills
(GT) Fever
(GT) Phlegm

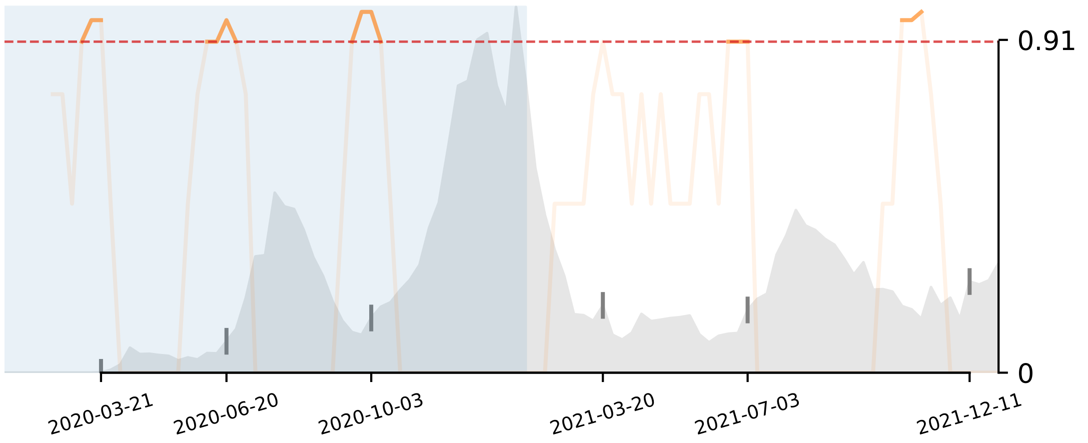

β	Training tp	Training fp	Training fn	Out of sample tp	Out of sample fp	Out of sample fn
1.00	0	0	3	0	0	3
0.99	0	0	3	1	0	2
0.96	1	0	2	1	1	2
0.91	3	0	0	3	1	0
0.76	3	2	0	3	5	0
0.46	3	4	0	3	11	0
0.00	3	6	0	3	28	0

NV, Washoe

Proxies selected by the Multiple Sources method

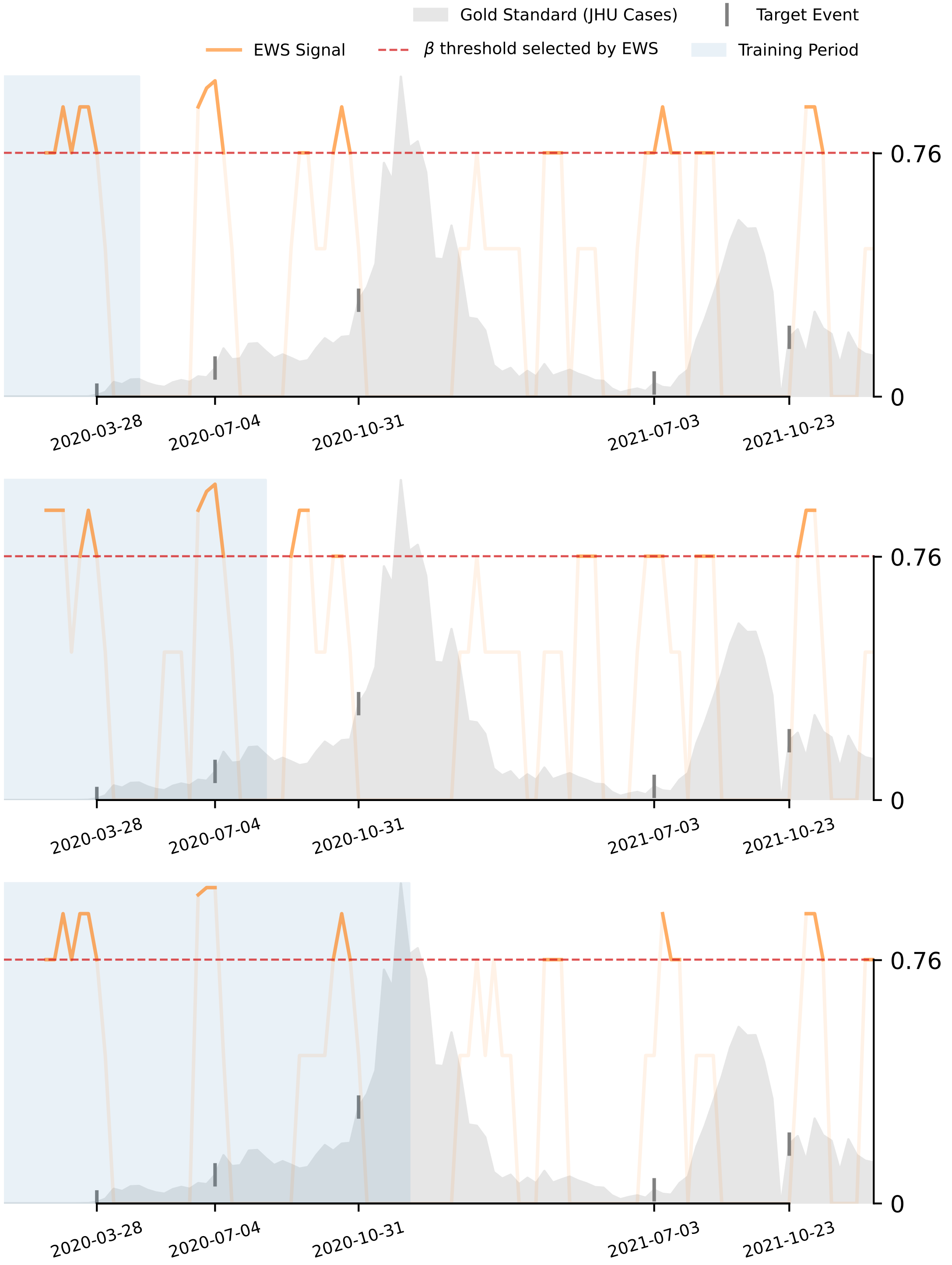

Performance as a function of β

Model 1

(GT) covid
(GT) covid-19
(GT) chest pain
(GT) covid symptoms
(GT) Acute bronchitis
(GT) Bronchitis

β	Training tp	Training fp	Training fn	Out of sample tp	Out of sample fp	Out of sample fn
1.00	0	0	1	0	0	4
0.99	0	0	1	1	0	3
0.96	0	0	1	1	0	3
0.91	0	0	1	2	3	0
0.76	1	0	0	3	15	0
0.46	1	2	0	3	30	0
0.00	1	3	0	4	59	0

Model 2

(GT) covid
(GT) chest pain
(GT) covid symptoms
(GT) Acute bronchitis
(GT) Bronchitis
(GT) Chills

β	Training tp	Training fp	Training fn	Out of sample tp	Out of sample fp	Out of sample fn
1.00	0	0	2	0	0	3
0.99	0	0	2	0	0	3
0.96	1	0	1	0	0	3
0.91	1	0	1	1	3	1
0.76	2	2	0	2	13	0
0.46	2	3	0	2	28	0
0.00	2	7	0	3	51	0

Model 3

(GT) covid
(GT) chest pain
(GT) covid-19
(GT) Bronchitis
(GT) Sore throat
(GT) covid symptoms

β	Training tp	Training fp	Training fn	Out of sample tp	Out of sample fp	Out of sample fn
1.00	0	0	3	0	0	2
0.99	0	0	3	0	0	2
0.96	1	0	2	0	0	2
0.91	1	0	2	0	3	0
0.76	3	0	0	0	13	0
0.46	3	2	0	1	22	0
0.00	3	7	0	2	41	0

Proxies selected by the Multiple Sources method

Model 1

(GT) covid
(GT) how long does covid last
(GT) loss smell
(GT) covid-19
(GT) loss taste
(GT) chest pain

Model 2

(GT) how long does covid last
(GT) nose bleed
(GT) covid-19
(GT) covid symptoms
(GT) sars-cov-2
(GT) Throat irritation

Model 3

(GT) covid
(GT) covid symptoms
(GT) Throat irritation
(GT) Asphyxia
(GT) nose bleed
(GT) how long does covid last

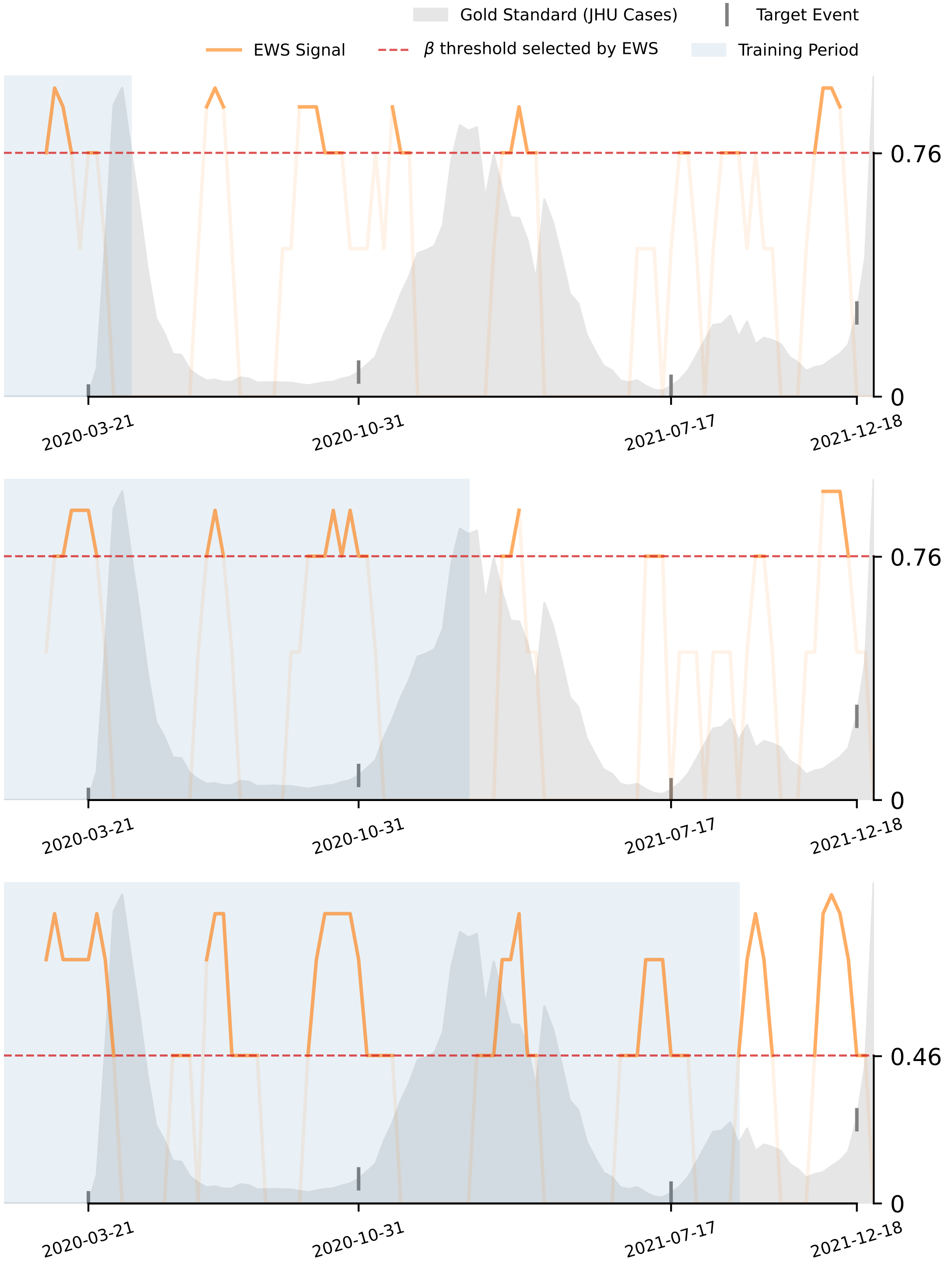

Performance as a function of β

Model 1

β	Training tp	Training fp	Training fn	Out of sample tp	Out of sample fp	Out of sample fn
1.00	0	0	1	0	0	3
0.99	0	0	1	0	0	3
0.96	0	0	1	1	1	2
0.91	1	0	0	2	6	1
0.76	1	0	0	2	19	0
0.46	1	2	0	3	31	0
0.00	1	3	0	3	67	0

Model 2

β	Training tp	Training fp	Training fn	Out of sample tp	Out of sample fp	Out of sample fn
1.00	0	0	2	0	0	2
0.99	0	0	2	0	0	2
0.96	0	0	2	1	0	1
0.91	0	0	2	1	1	1
0.76	2	1	0	2	5	0
0.46	2	7	0	2	16	0
0.00	2	14	0	2	34	0

Model 3

β	Training tp	Training fp	Training fn	Out of sample tp	Out of sample fp	Out of sample fn
1.00	0	0	3	0	0	1
0.99	0	0	3	0	0	1
0.96	0	0	3	1	0	0
0.91	0	0	3	1	1	0
0.76	2	4	1	1	3	0
0.46	3	10	0	1	5	0
0.00	3	33	0	1	9	0

Proxies selected by the Multiple Sources method

Model 1

(GT) covid
(GT) how long does covid last
(GT) loss smell
(GT) covid-19
(GT) loss taste
(GT) chest pain

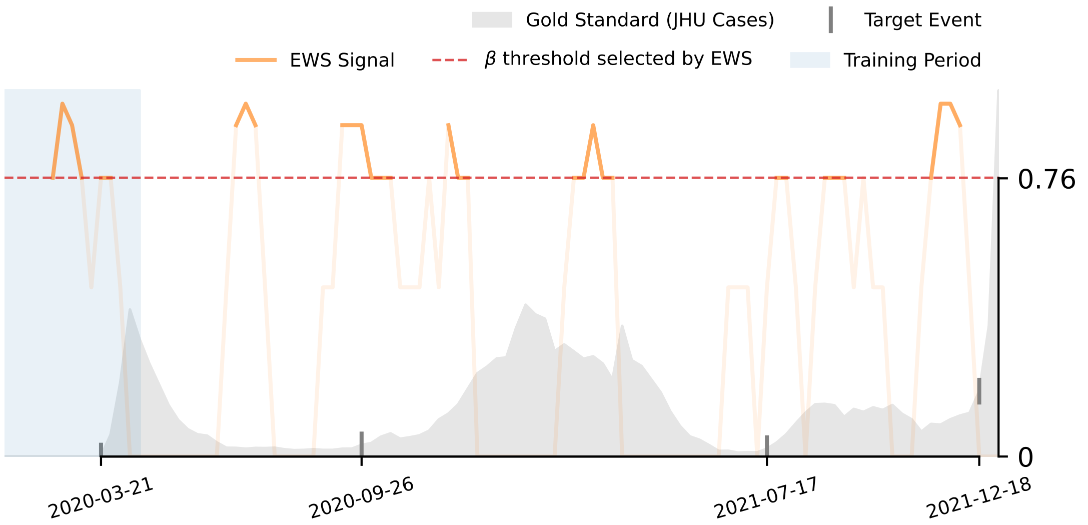

Performance as a function of β

	Training			Out of sample		
β	tp	fp	fn	tp	fp	fn
1.00	0	0	1	0	0	3
0.99	0	0	1	0	0	3
0.96	0	0	1	1	1	2
0.91	1	0	0	2	5	1
0.76	1	0	0	2	21	0
0.46	1	2	0	3	33	0
0.00	1	3	0	3	68	0

Model 2

(GT) how long does covid last
(GT) loss smell
(GT) covid symptoms
(GT) sars-cov-2
(GT) Asthma
(GT) Sore throat

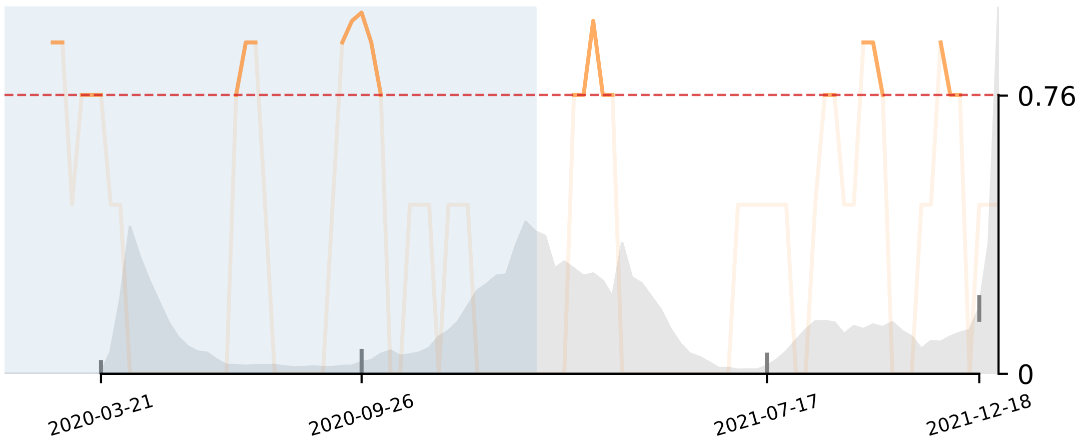

	Training			Out of sample		
β	tp	fp	fn	tp	fp	fn
1.00	0	0	2	0	0	2
0.99	0	0	2	0	0	2
0.96	1	0	1	0	1	2
0.91	1	0	1	1	3	1
0.76	2	4	0	1	10	1
0.46	2	6	0	2	17	0
0.00	2	15	0	2	34	0

Model 3

(GT) Asthma
(GT) covid symptoms
(GT) Chronic pain
(GT) Shortness of breath
(GT) chest pain
(GT) Ageusia

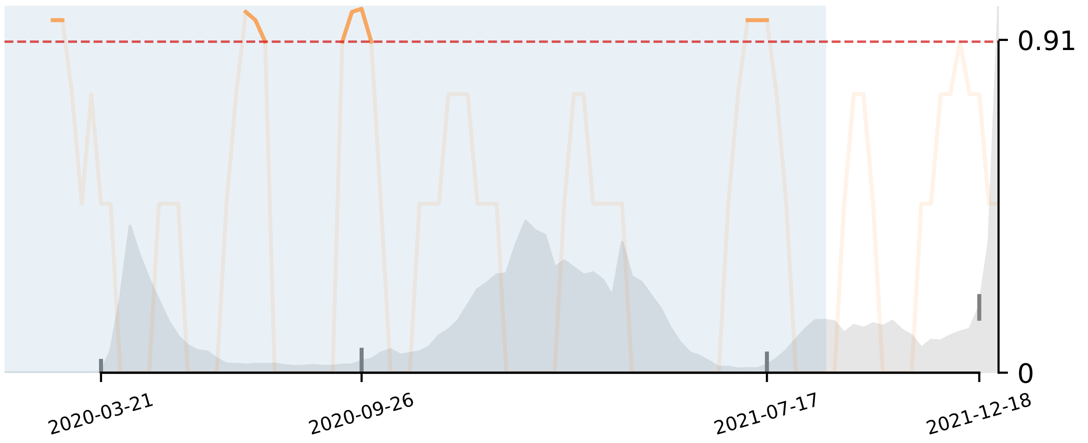

	Training			Out of sample		
β	tp	fp	fn	tp	fp	fn
1.00	0	0	3	0	0	1
0.99	1	0	2	0	0	1
0.96	1	1	2	0	0	1
0.91	3	3	0	1	0	0
0.76	3	5	0	1	2	0
0.46	3	12	0	1	6	0
0.00	3	30	0	1	11	0

NY, Monroe

Proxies selected by the Multiple Sources method

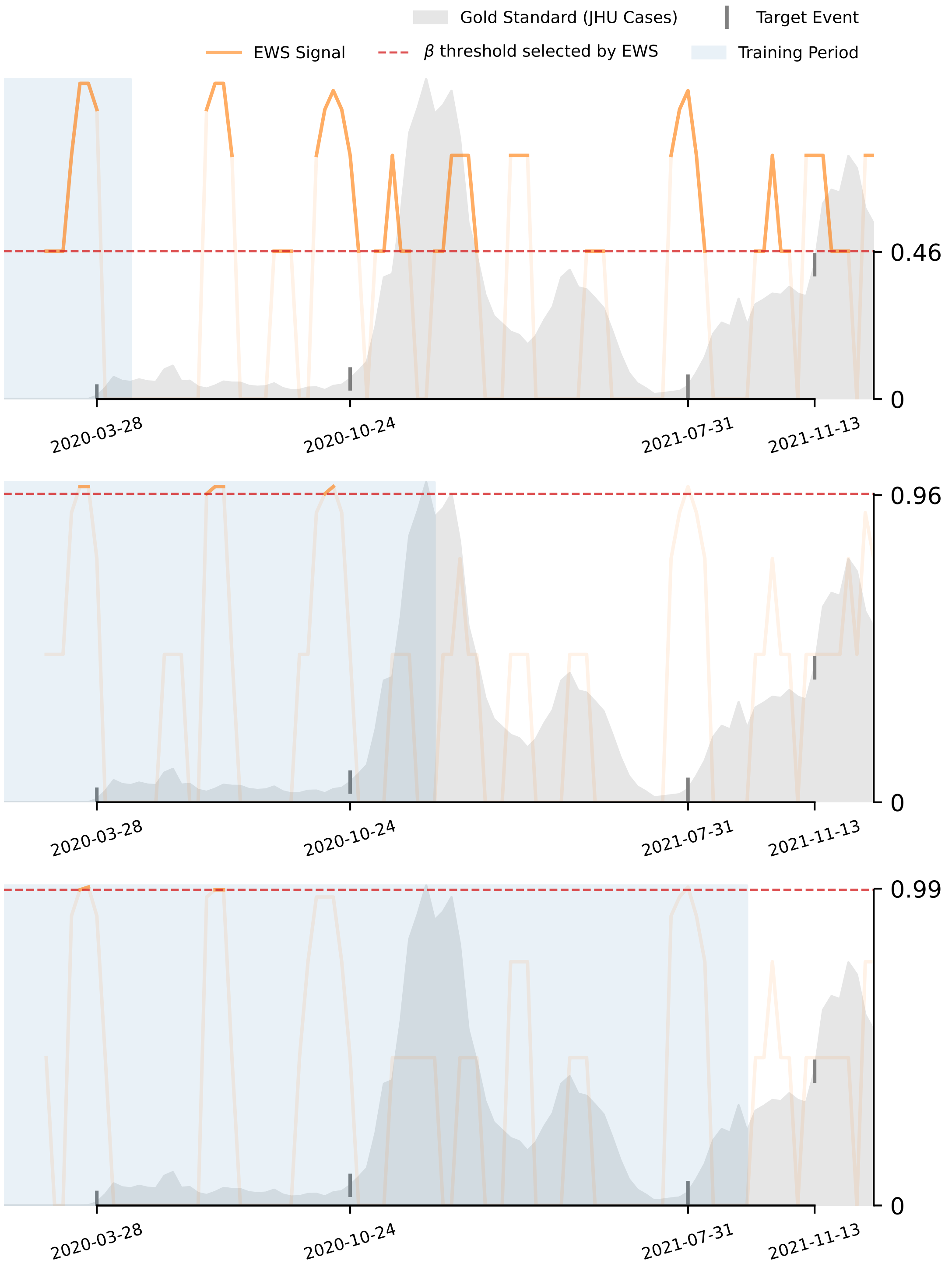

Performance as a function of β

Model 1

(GT) covid
(GT) covid-19
(GT) chest pain
(GT) covid symptoms
(GT) quarantine
(GT) fever

β	Training tp	Training fp	Training fn	Out of sample tp	Out of sample fp	Out of sample fn
1.00	0	0	1	0	0	3
0.99	0	0	1	0	2	3
0.96	1	0	0	2	2	1
0.91	1	0	0	2	3	1
0.76	1	0	0	3	15	0
0.46	1	0	0	3	34	0
0.00	1	2	0	3	67	0

Model 2

(GT) covid
(GT) covid-19
(GT) covid symptoms
(GT) Ageusia
(GT) Diarrhea
(GT) quarantine

β	Training tp	Training fp	Training fn	Out of sample tp	Out of sample fp	Out of sample fn
1.00	0	0	2	0	0	2
0.99	0	0	2	1	0	1
0.96	2	2	0	1	0	1
0.91	2	3	0	1	2	1
0.76	2	3	0	2	6	0
0.46	2	3	0	2	21	0
0.00	2	14	0	2	38	0

Model 3

(GT) covid
(GT) covid-19
(GT) covid symptoms
(GT) quarantine
(GT) Ageusia
(GT) Hyperventilation

β	Training tp	Training fp	Training fn	Out of sample tp	Out of sample fp	Out of sample fn
1.00	0	0	3	0	0	1
0.99	2	0	1	0	0	1
0.96	2	2	1	0	0	1
0.91	3	3	0	0	0	1
0.76	3	4	0	1	2	0
0.46	3	9	0	1	7	0
0.00	3	25	0	1	8	0

NY, Nassau

112

Proxies selected by the Multiple Sources method

Model 1

(GT) covid
(GT) how long does covid last
(GT) loss smell
(GT) covid-19
(GT) loss taste
(GT) chest pain

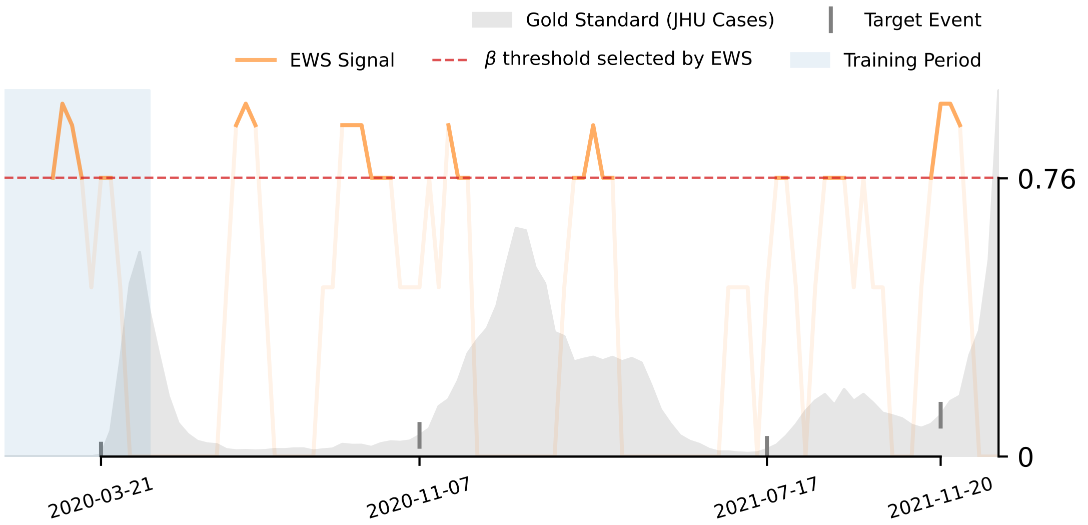

Performance as a function of β

β	Training tp	Training fp	Training fn	Out of sample tp	Out of sample fp	Out of sample fn
1.00	0	0	1	0	0	3
0.99	0	0	1	0	0	3
0.96	0	0	1	1	2	2
0.91	1	0	0	2	9	1
0.76	1	0	0	2	22	0
0.46	1	2	0	3	33	0
0.00	1	3	0	3	67	0

Model 2

(GT) nose bleed
(GT) covid-19
(GT) covid symptoms
(GT) sars-cov-2
(GT) Abdominal pain
(GT) Otitis

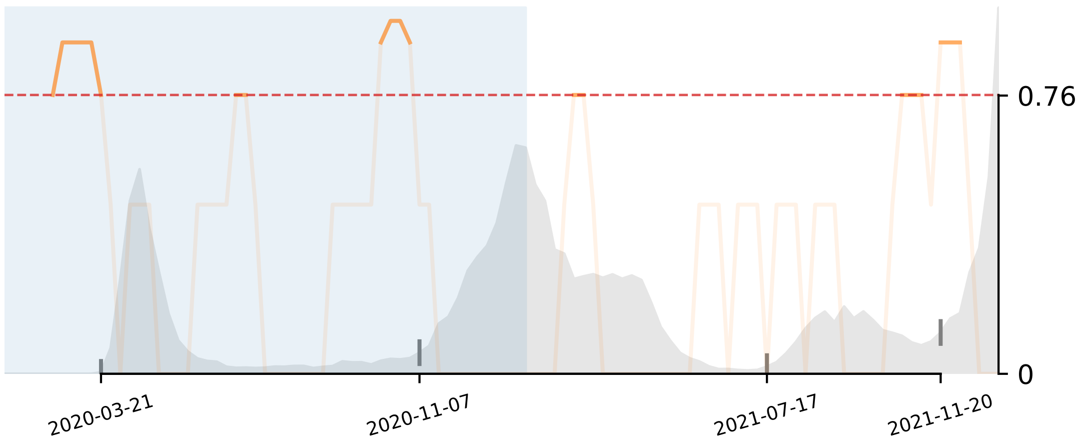

β	Training tp	Training fp	Training fn	Out of sample tp	Out of sample fp	Out of sample fn
1.00	0	0	2	0	0	2
0.99	0	0	2	0	0	2
0.96	0	0	2	0	0	2
0.91	1	0	1	1	2	1
0.76	2	0	0	1	4	1
0.46	2	3	0	2	14	0
0.00	2	18	0	2	35	0

Model 3

(GT) covid
(GT) covid symptoms
(GT) nose bleed
(GT) covid-19
(GT) covid-19 who
(GT) sars-cov-2

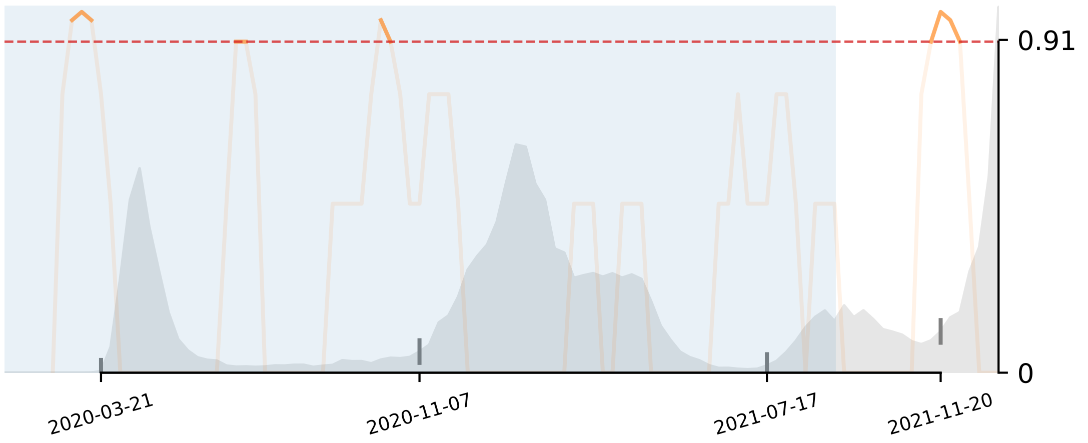

β	Training tp	Training fp	Training fn	Out of sample tp	Out of sample fp	Out of sample fn
1.00	0	0	3	0	0	1
0.99	0	0	3	1	0	0
0.96	1	0	2	1	1	0
0.91	2	0	1	1	2	0
0.76	2	2	1	1	2	0
0.46	3	9	0	1	3	0
0.00	3	27	0	1	10	0

NY, New York

Proxies selected by the Multiple Sources method

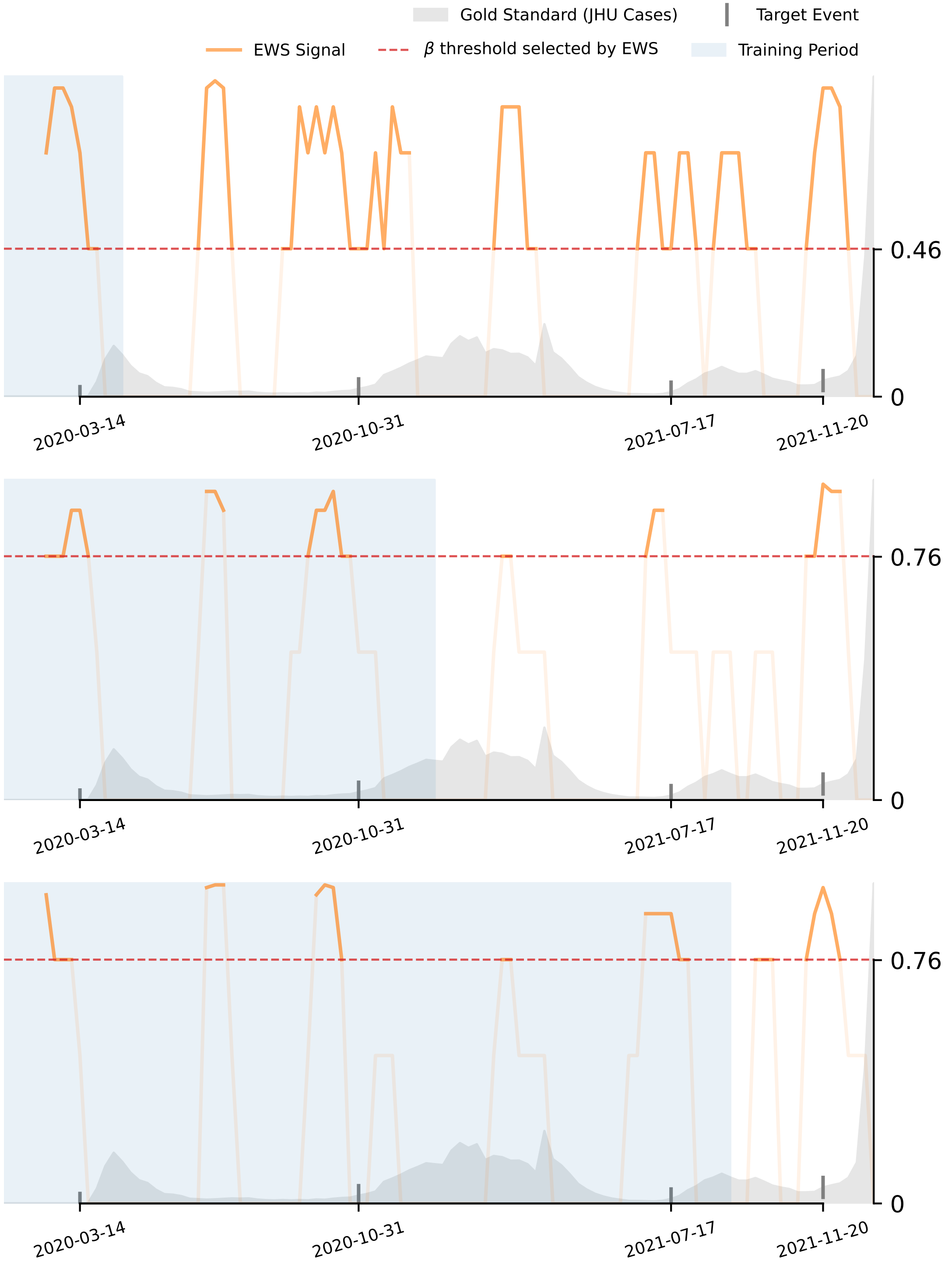

Performance as a function of β

Model 1

(GT) covid
(GT) loss smell
(GT) covid-19
(GT) loss taste
(GT) chest pain
(GT) covid symptoms

	Training			Out of sample		
β	tp	fp	fn	tp	fp	fn
1.00	0	0	1	0	0	3
0.99	0	0	1	0	1	3
0.96	0	0	1	1	4	2
0.91	1	0	0	2	10	1
0.76	1	0	0	3	18	0
0.46	1	0	0	3	32	0
0.00	1	2	0	3	68	0

Model 2

(GT) nose bleed
(GT) covid-19
(GT) covid symptoms
(GT) quarantine
(GT) sars-cov-2
(GT) Ageusia

β	tp	fp	fn	tp	fp	fn
1.00	0	0	2	0	0	2
0.99	0	0	2	1	0	1
0.96	0	0	2	1	2	1
0.91	1	2	1	2	2	0
0.76	2	4	0	2	4	0
0.46	2	6	0	2	18	0
0.00	2	12	0	2	38	0

Model 3

(GT) Shortness of breath
Twitter (state level signal)
(GT) covid
(GT) covid symptoms
(GT) quarantine
(GT) Ageusia

β	tp	fp	fn	tp	fp	fn
1.00	0	0	3	0	0	1
0.99	1	2	2	1	0	0
0.96	1	3	2	1	0	0
0.91	2	4	1	1	1	0
0.76	3	4	0	1	4	0
0.46	3	8	0	1	7	0
0.00	3	19	0	1	10	0

NY, Queens

Proxies selected by the Multiple Sources method

Model 1

(GT) covid
(GT) how long does covid last
(GT) loss smell
(GT) covid-19
(GT) loss taste
(GT) chest pain

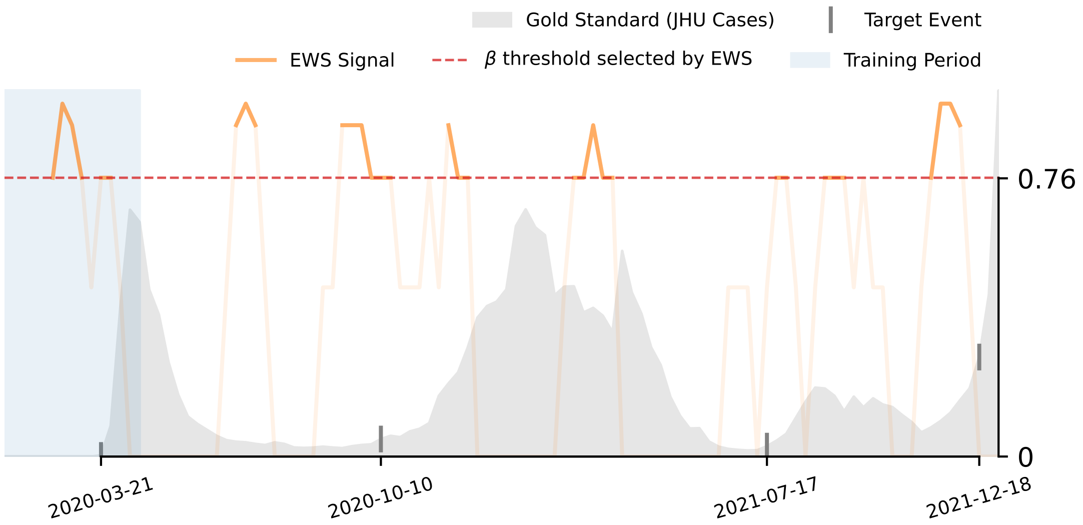

Performance as a function of β

	Training			Out of sample		
β	tp	fp	fn	tp	fp	fn
1.00	0	0	1	0	0	3
0.99	0	0	1	0	0	3
0.96	0	0	1	1	1	2
0.91	1	0	0	2	5	1
0.76	1	0	0	2	19	0
0.46	1	2	0	3	31	0
0.00	1	3	0	3	68	0

Model 2

(GT) how long does covid last
(GT) nose bleed
(GT) loss smell
(GT) covid-19
(GT) covid symptoms
(GT) sars-cov-2

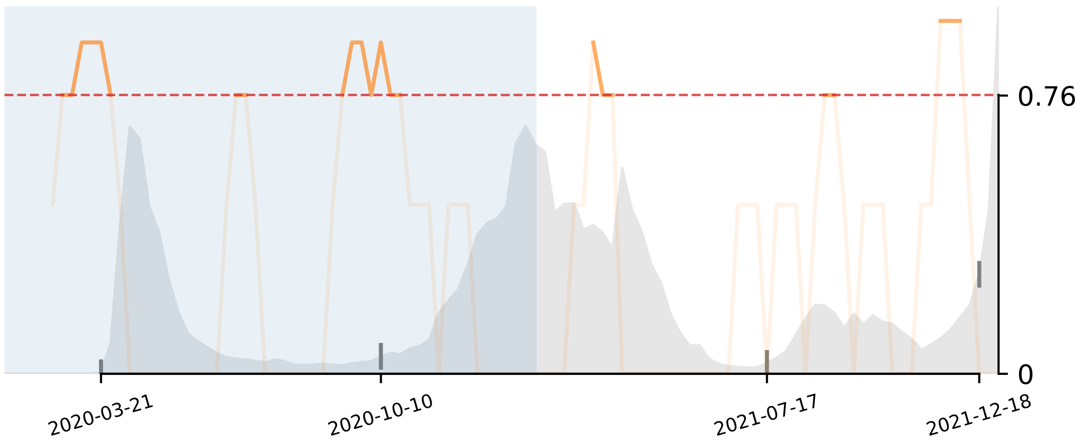

	Training			Out of sample		
β	tp	fp	fn	tp	fp	fn
1.00	0	0	2	0	0	2
0.99	0	0	2	0	0	2
0.96	0	0	2	1	0	1
0.91	0	0	2	1	1	1
0.76	2	0	0	1	5	1
0.46	2	5	0	2	15	0
0.00	2	16	0	2	34	0

Model 3

(GT) Asthma
(GT) covid
(GT) covid symptoms
(GT) Ageusia
(GT) chest pain
(GT) Bronchitis

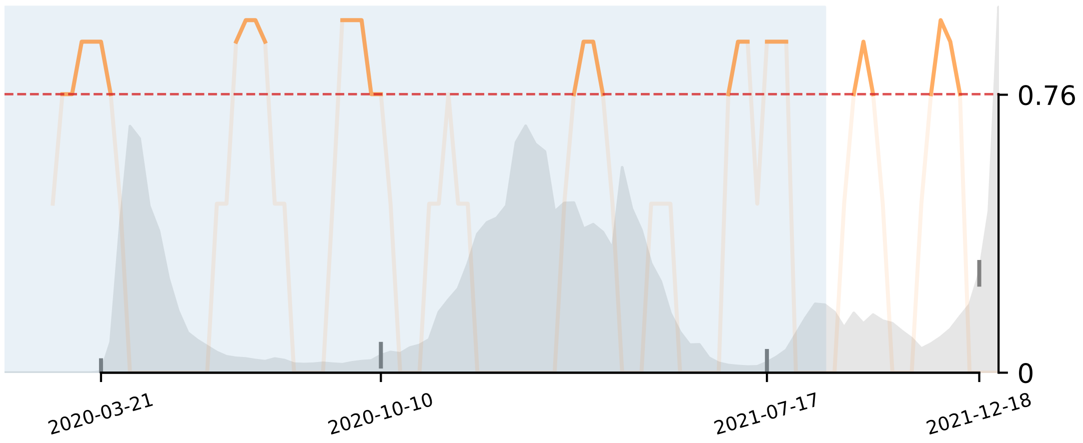

	Training			Out of sample		
β	tp	fp	fn	tp	fp	fn
1.00	0	0	3	0	0	1
0.99	0	0	3	0	0	1
0.96	0	0	3	1	0	0
0.91	1	2	2	1	1	0
0.76	3	8	0	1	3	0
0.46	3	12	0	1	5	0
0.00	3	29	0	1	11	0

NY, Suffolk

Proxies selected by the Multiple Sources method

Model 1

(GT) covid
(GT) how long does covid last
(GT) loss smell
(GT) covid-19
(GT) loss taste
(GT) chest pain

Model 2

(GT) how long does covid last
(GT) nose bleed
(GT) loss smell
(GT) covid-19
(GT) covid symptoms
(GT) sars-cov-2

Model 3

(GT) covid-19
(GT) covid
(GT) covid symptoms
(GT) Anosmia
(GT) chest pain
(GT) Viral pneumonia

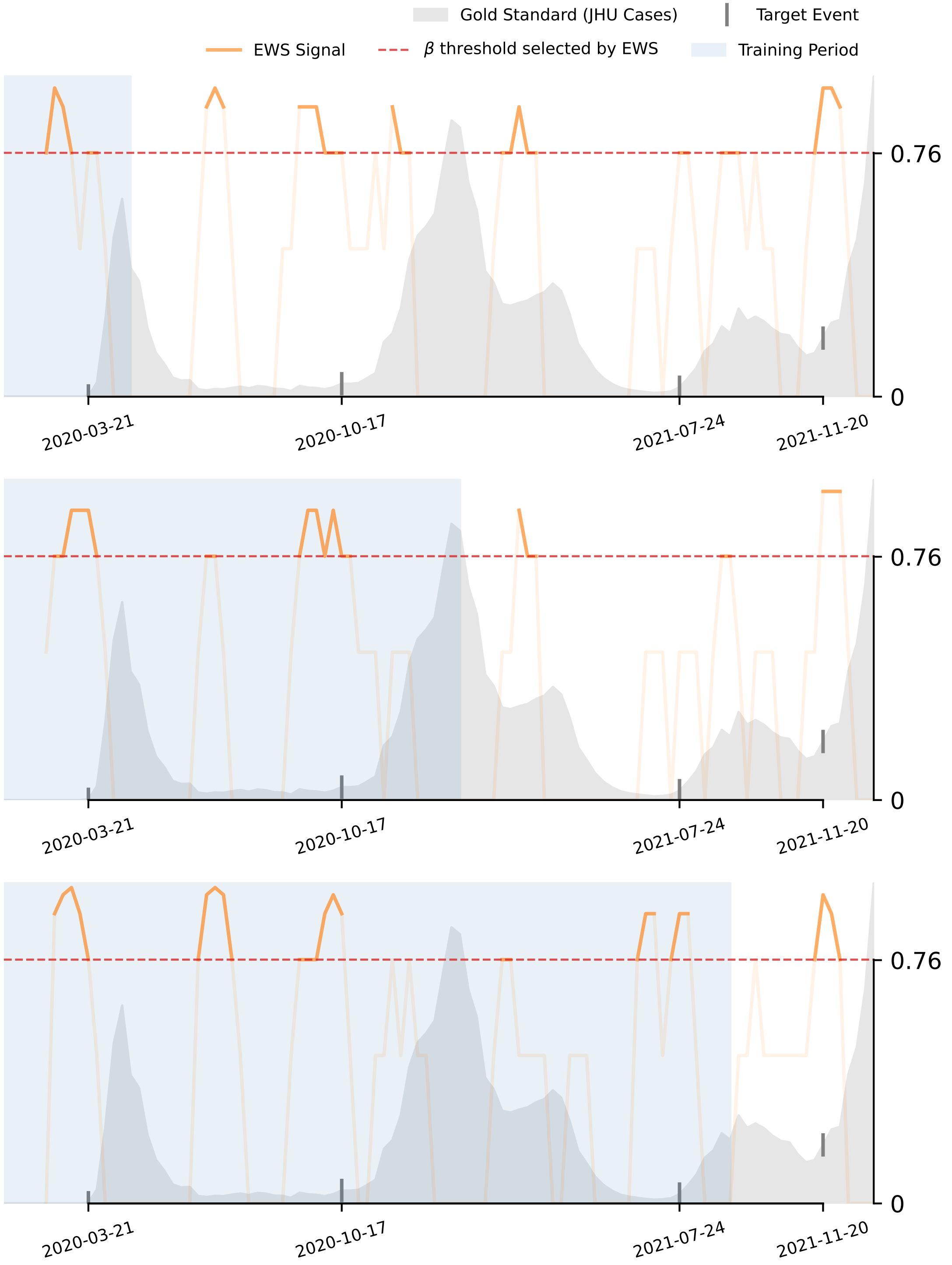

Performance as a function of β

Model 1

β	Training tp	Training fp	Training fn	Out of sample tp	Out of sample fp	Out of sample fn
1.00	0	0	1	0	0	3
0.99	0	0	1	0	0	3
0.96	0	0	1	1	2	2
0.91	1	0	0	2	7	1
0.76	1	0	0	3	19	0
0.46	1	2	0	3	32	0
0.00	1	3	0	3	67	0

Model 2

β	Training tp	Training fp	Training fn	Out of sample tp	Out of sample fp	Out of sample fn
1.00	0	0	2	0	0	2
0.99	0	0	2	0	0	2
0.96	0	0	2	1	2	1
0.91	0	0	2	1	3	1
0.76	2	0	0	1	7	1
0.46	2	5	0	2	16	0
0.00	2	16	0	2	35	0

Model 3

β	Training tp	Training fp	Training fn	Out of sample tp	Out of sample fp	Out of sample fn
1.00	0	0	3	0	0	1
0.99	0	0	3	0	0	1
0.96	1	1	2	1	0	0
0.91	2	3	1	1	1	0
0.76	3	4	0	1	3	0
0.46	3	12	0	1	6	0
0.00	3	30	0	1	10	0

OH, Cuyahoga

Proxies selected by the Multiple Sources method

Model 1

(GT) covid
(GT) covid-19
(GT) covid symptoms
(GT) chest tightness
(GT) quarantine
(GT) Abdominal pain

Model 2

(GT) covid
(GT) covid-19
(GT) covid symptoms
(GT) chest tightness
(GT) quarantine
(GT) Acute bronchitis

Model 3

(GT) covid
(GT) covid symptoms
(GT) Acute bronchitis
(GT) Anosmia
(GT) Asthma
(GT) Bronchitis

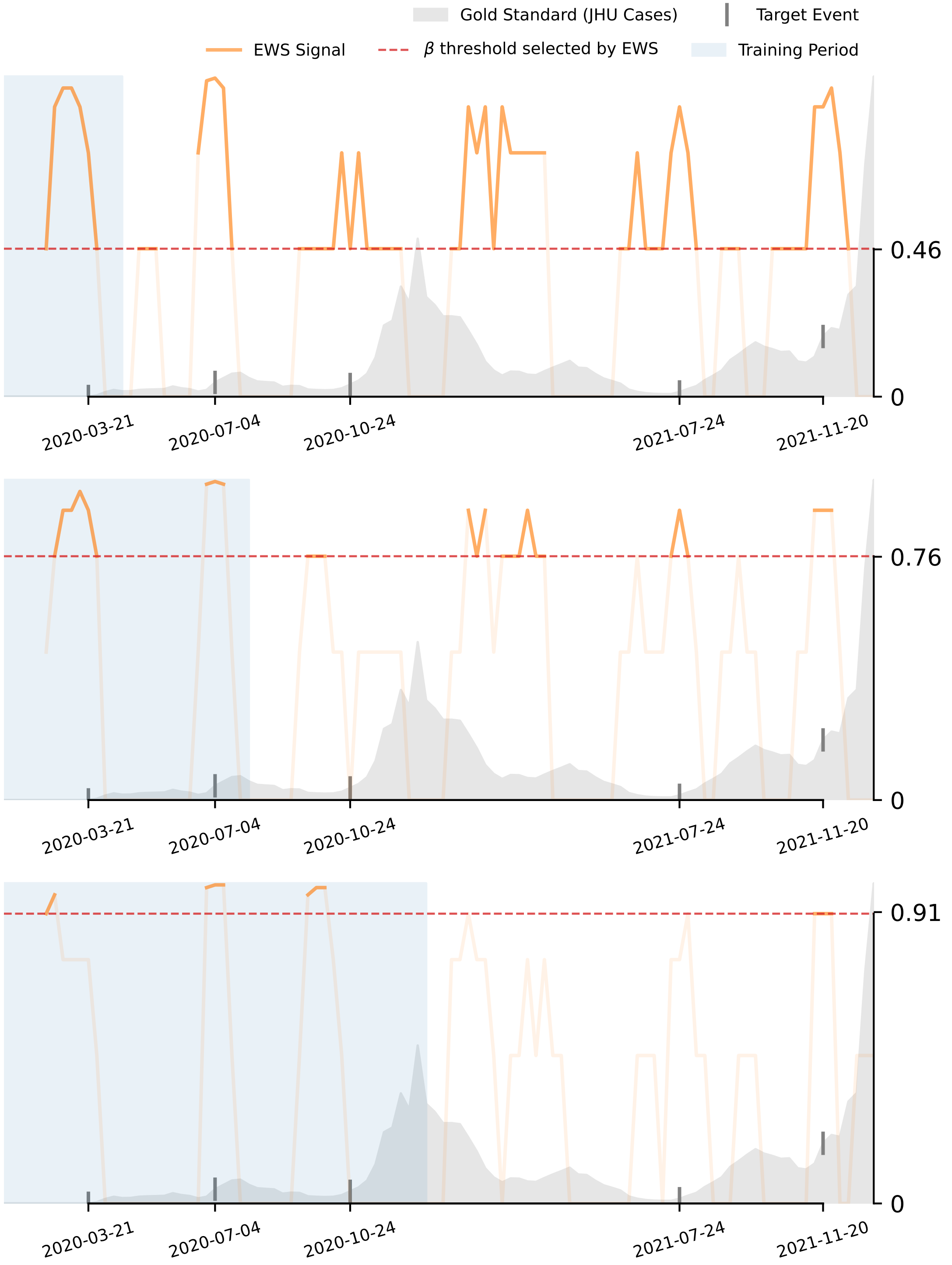

Performance as a function of β

Model 1

	Training			Out of sample		
β	tp	fp	fn	tp	fp	fn
1.00	0	0	1	1	0	3
0.99	0	0	1	1	0	3
0.96	0	0	1	1	2	2
0.91	1	0	0	3	5	1
0.76	1	0	0	4	14	0
0.46	1	0	0	4	32	0
0.00	1	2	0	4	61	0

Model 2

β	tp	fp	fn	tp	fp	fn
1.00	0	0	2	0	0	3
0.99	1	0	1	0	0	3
0.96	1	1	1	0	0	3
0.91	2	1	0	2	4	1
0.76	2	1	0	3	12	0
0.46	2	2	0	3	28	0
0.00	2	4	0	3	53	0

Model 3

β	tp	fp	fn	tp	fp	fn
1.00	0	0	3	0	0	2
0.99	1	1	2	0	0	2
0.96	2	1	1	0	0	2
0.91	3	2	0	1	3	0
0.76	3	3	0	2	9	0
0.46	3	3	0	2	23	0
0.00	3	6	0	2	39	0

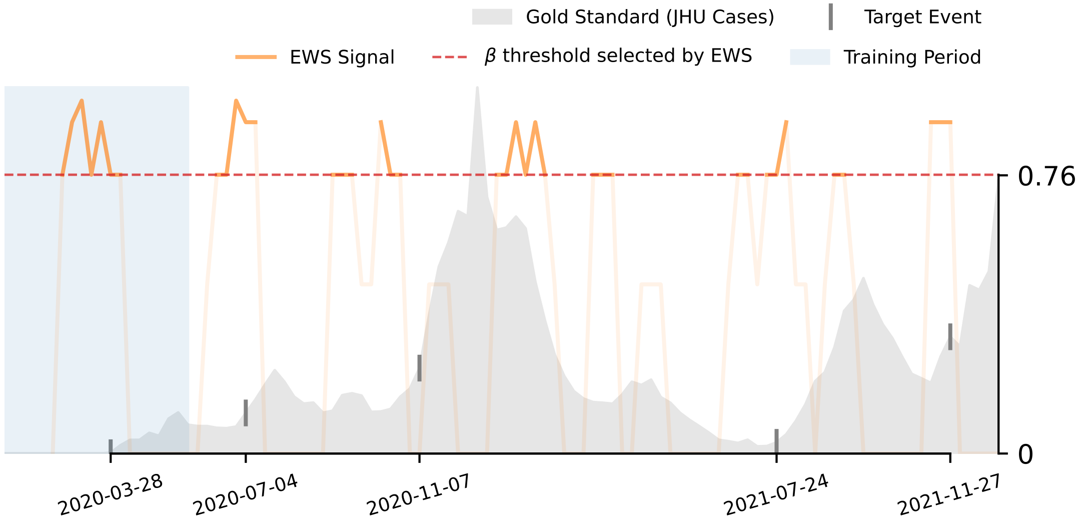

Proxies selected by the Multiple Sources method

Model 1

(GT) covid
(GT) covid-19
(GT) chest pain
(GT) chest tightness
(GT) Burning Chest Pain
(GT) Hyperventilation

Performance as a function of β

	Training			Out of sample		
β	tp	fp	fn	tp	fp	fn
1.00	0	0	1	0	0	4
0.99	0	0	1	0	0	4
0.96	0	0	1	1	0	3
0.91	1	0	0	3	4	0
0.76	1	0	0	4	16	0
0.46	1	2	0	4	27	0
0.00	1	2	0	4	57	0

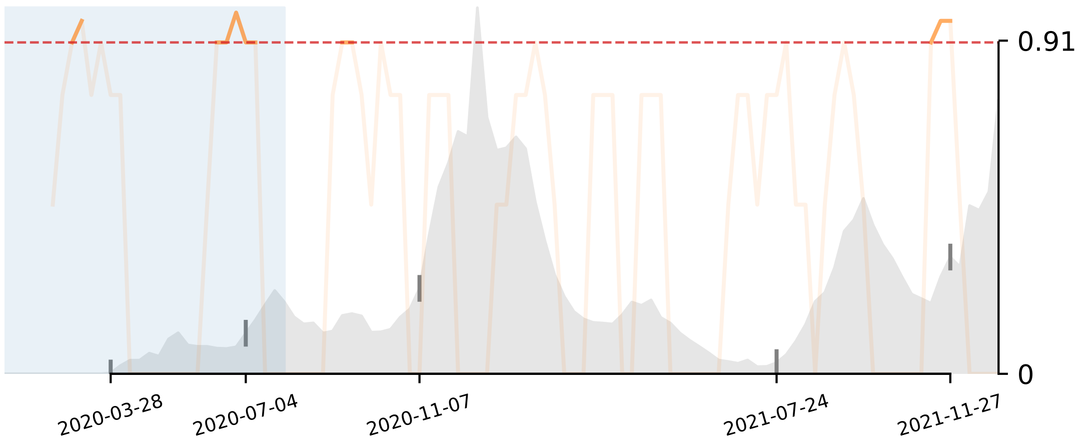

Model 2

(GT) covid
(GT) covid-19
(GT) chest pain
(GT) Burning Chest Pain
(GT) Hyperventilation
(GT) Chest pain

β	tp	fp	fn	tp	fp	fn
1.00	0	0	2	0	0	3
0.99	0	0	2	0	0	3
0.96	1	0	1	1	0	2
0.91	2	0	0	2	5	0
0.76	2	1	0	3	20	0
0.46	2	3	0	3	28	0
0.00	2	4	0	3	53	0

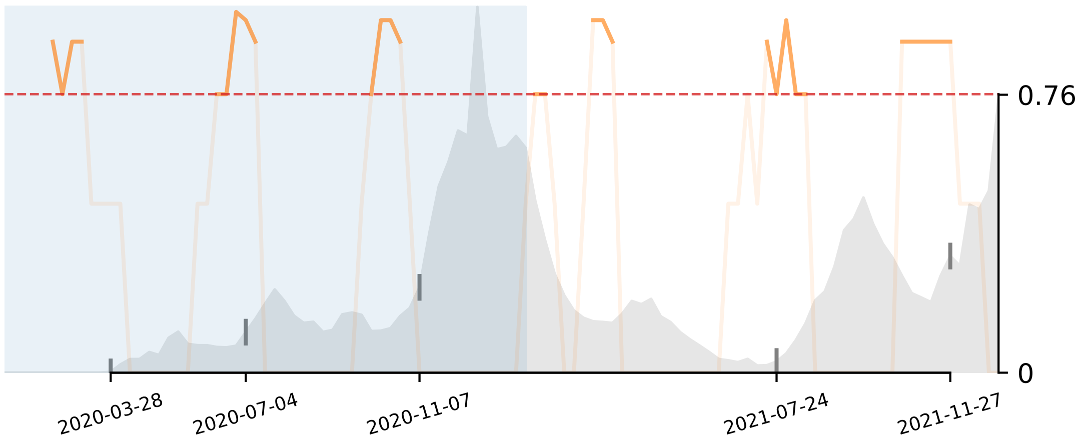

Model 3

(GT) covid-19
(GT) chest pain
(GT) covid
(GT) quarantine
(GT) Chills
(GT) Diarrhea

β	tp	fp	fn	tp	fp	fn
1.00	0	0	3	0	0	2
0.99	0	0	3	0	0	2
0.96	1	0	2	0	3	1
0.91	2	0	1	2	4	0
0.76	3	2	0	2	8	0
0.46	3	4	0	2	13	0
0.00	3	8	0	2	35	0

Proxies selected by the Multiple Sources method

Model 1

(GT) covid
(GT) covid-19
(GT) covid symptoms
(GT) chest tightness
(GT) quarantine
(GT) sars-cov-2

Model 2

(GT) covid symptoms
(GT) Otitis
(GT) Phlegm

Model 3

(GT) covid symptoms
(GT) Otitis
(GT) Phlegm
(GT) Ageusia
(GT) Anosmia
(GT) quarantine

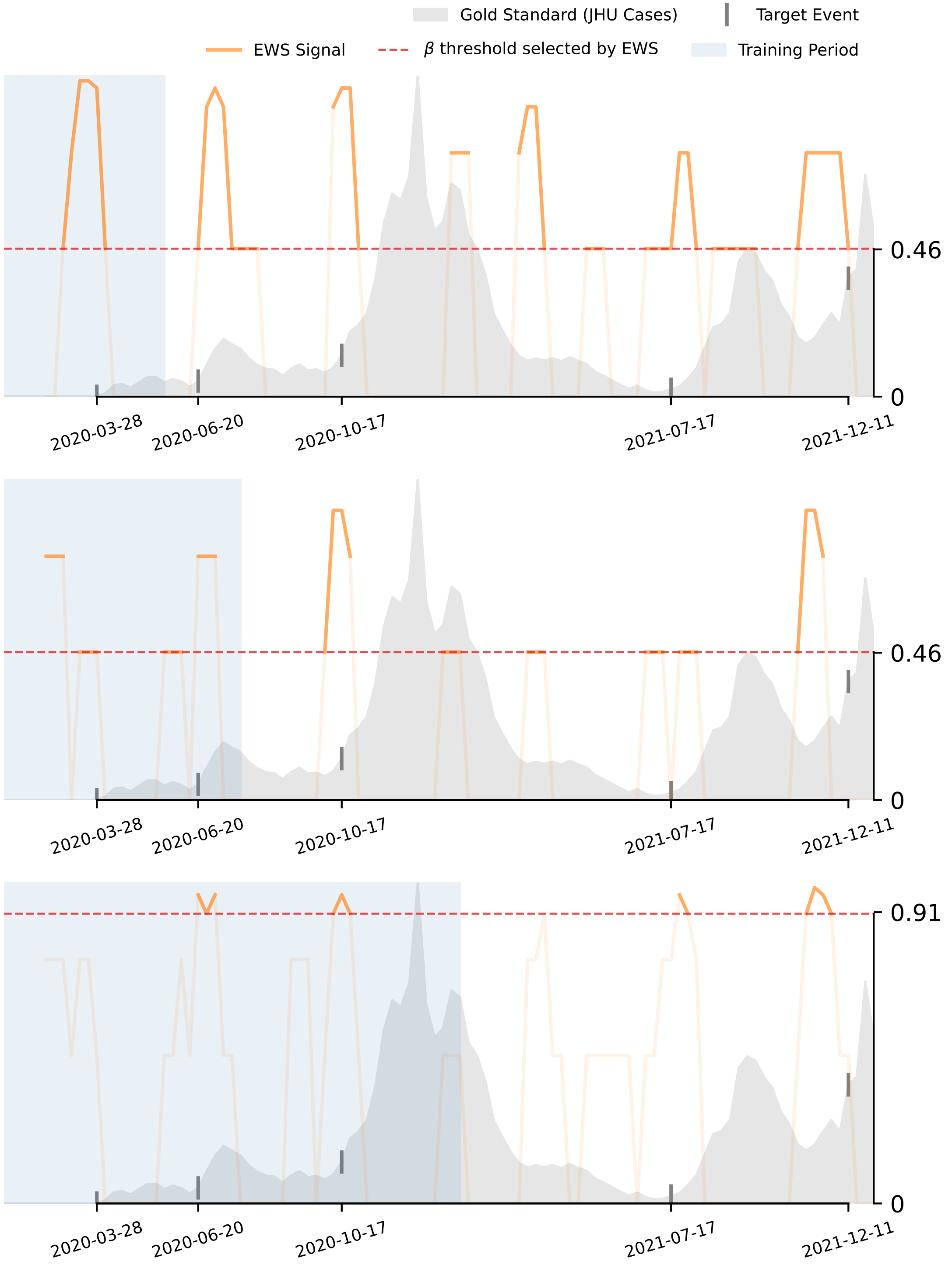

Performance as a function of β

Model 1

β	Training tp	Training fp	Training fn	Out of sample tp	Out of sample fp	Out of sample fn
1.00	0	0	1	0	0	4
0.99	0	0	1	0	0	4
0.96	1	0	0	1	2	2
0.91	1	0	0	1	6	2
0.76	1	0	0	2	12	0
0.46	1	0	0	4	28	0
0.00	1	1	0	4	59	0

Model 2

β	Training tp	Training fp	Training fn	Out of sample tp	Out of sample fp	Out of sample fn
0.91	0	0	2	2	0	1
0.76	0	0	2	2	1	1
0.46	2	4	0	3	10	0
0.00	2	4	0	3	54	0

Model 3

β	Training tp	Training fp	Training fn	Out of sample tp	Out of sample fp	Out of sample fn
1.00	0	0	3	0	0	2
0.99	0	0	3	1	0	1
0.96	0	0	3	1	1	0
0.91	2	1	1	1	3	0
0.76	2	3	1	2	6	0
0.46	3	7	0	2	12	0
0.00	3	13	0	2	35	0

OH, Summit

Proxies selected by the Multiple Sources method

Performance as a function of β

Model 1

(GT) covid
(GT) how long does covid last
(GT) covid-19
(GT) covid symptoms
(GT) chest tightness
(GT) quarantine

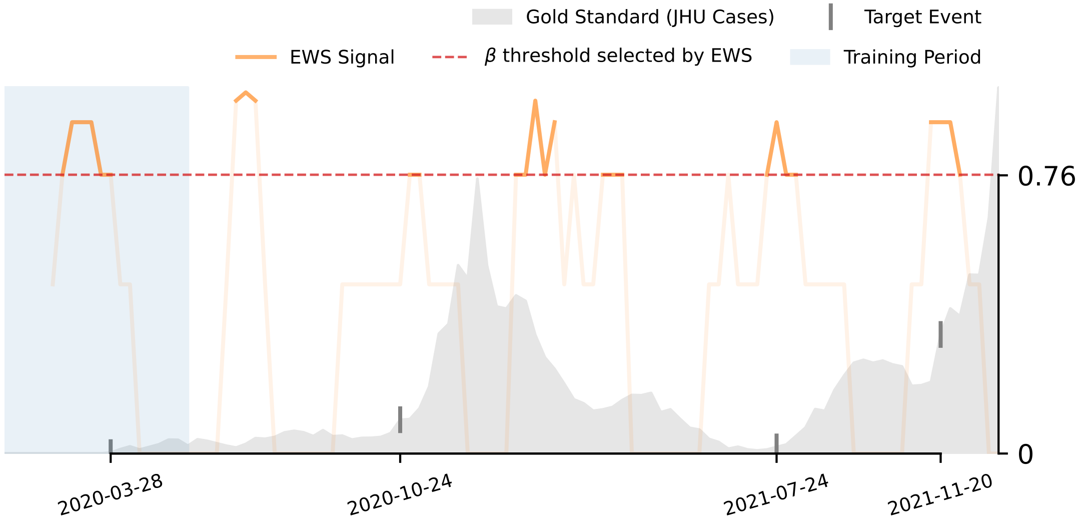

β	Training tp	Training fp	Training fn	Out of sample tp	Out of sample fp	Out of sample fn
1.00	0	0	1	0	0	3
0.99	0	0	1	0	1	3
0.96	0	0	1	0	4	3
0.91	0	0	1	2	6	1
0.76	1	0	0	2	18	0
0.46	1	1	0	3	35	0
0.00	1	4	0	3	63	0

Model 2

(GT) how long does covid last
(GT) covid
(GT) covid symptoms
(GT) Otitis

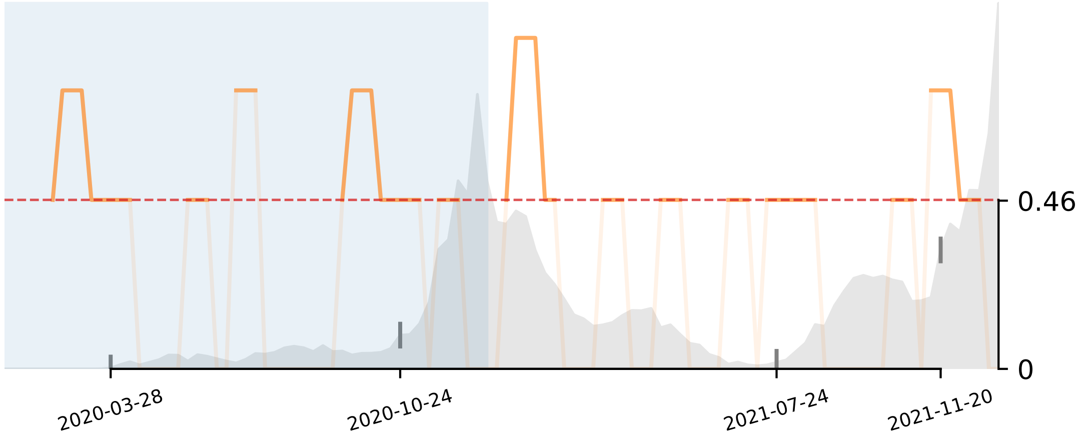

β	Training tp	Training fp	Training fn	Out of sample tp	Out of sample fp	Out of sample fn
0.96	0	0	2	0	0	2
0.91	0	0	2	0	3	2
0.76	0	0	2	1	4	1
0.46	2	5	0	2	20	0
0.00	2	17	0	2	39	0

Model 3

(GT) covid
(GT) covid symptoms
(GT) how long does covid last
(GT) Asthma
(GT) Nasal congestion
Twitter (state level signal)

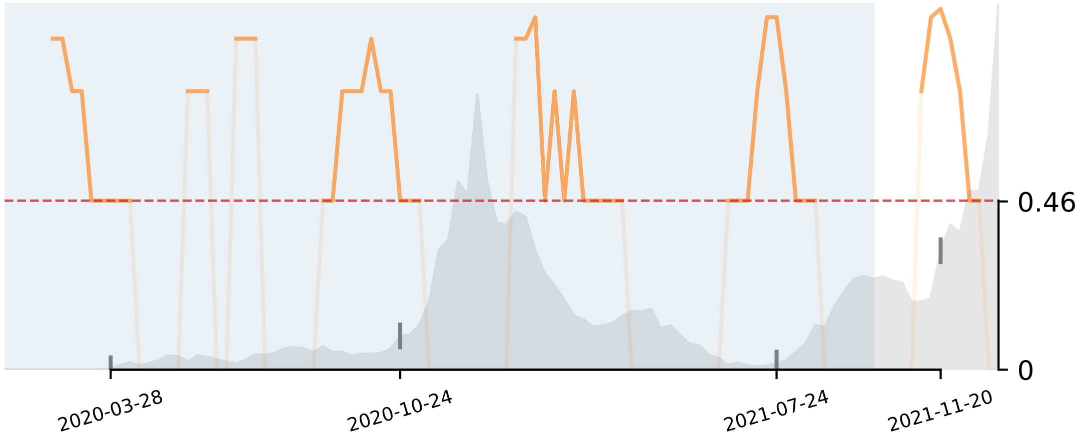

β	Training tp	Training fp	Training fn	Out of sample tp	Out of sample fp	Out of sample fn
1.00	0	0	3	0	0	1
0.99	0	0	3	1	0	0
0.96	0	0	3	1	0	0
0.91	1	1	2	1	1	0
0.76	2	8	1	1	2	0
0.46	3	16	0	1	4	0
0.00	3	33	0	1	6	0

OH, Warren

Proxies selected by the Multiple Sources method

Model 1

(GT) chest tightness
(GT) covid-19 who
(GT) covid
(GT) covid-19
(GT) covid symptoms
(GT) quarantine

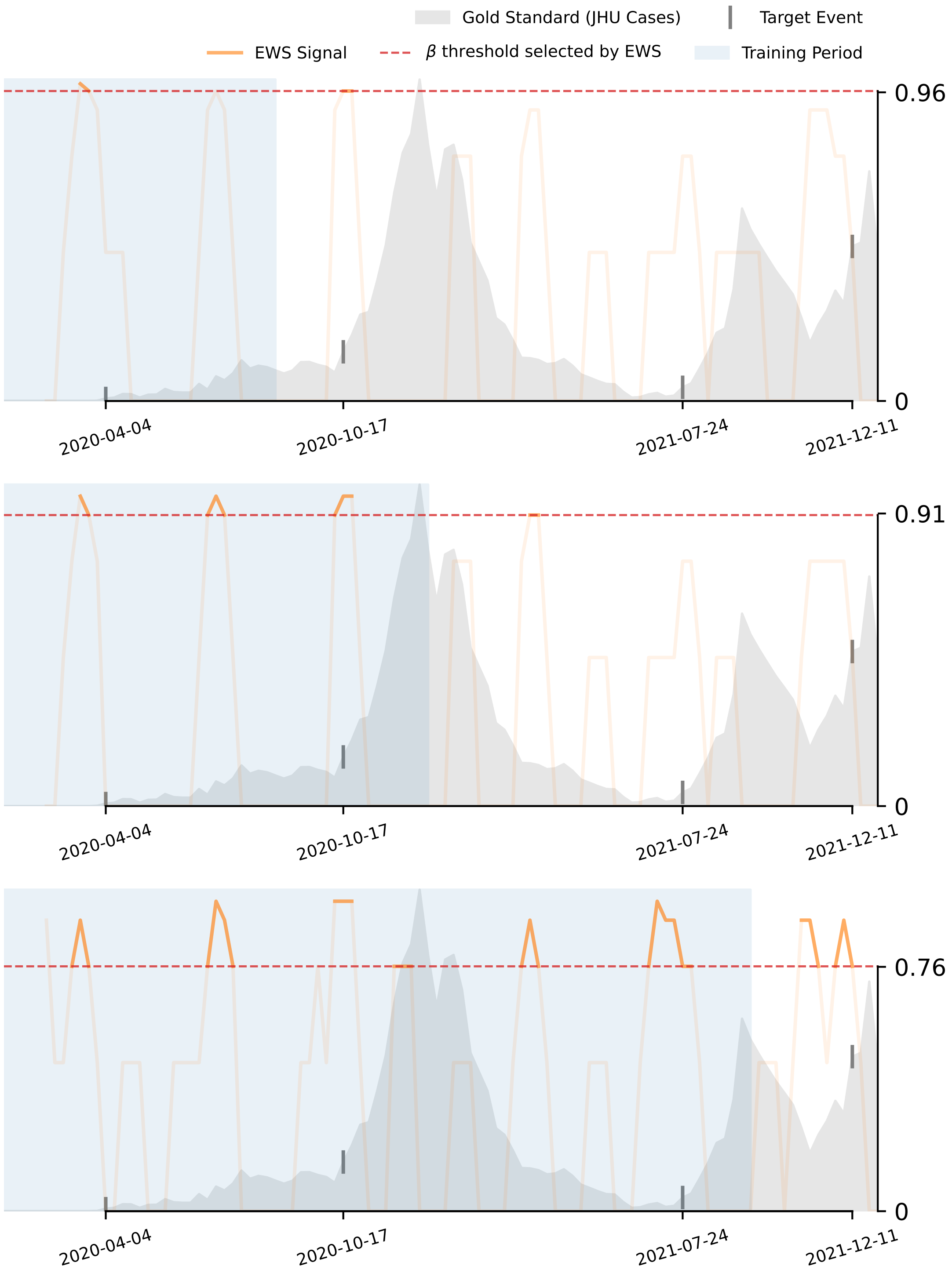

Performance as a function of β

β	Training tp	Training fp	Training fn	Out of sample tp	Out of sample fp	Out of sample fn
1.00	0	0	1	0	0	3
0.99	0	0	1	0	0	3
0.96	1	0	0	1	1	2
0.91	1	1	0	2	3	1
0.76	1	3	0	3	8	0
0.46	1	3	0	3	20	0
0.00	1	8	0	3	50	0

Model 2

(GT) covid
(GT) covid-19
(GT) covid symptoms
(GT) quarantine

β	Training tp	Training fp	Training fn	Out of sample tp	Out of sample fp	Out of sample fn
0.96	0	0	2	0	0	2
0.91	2	2	0	0	2	2
0.76	2	4	0	2	7	0
0.46	2	4	0	2	15	0
0.00	2	8	0	2	39	0

Model 3

(GT) covid symptoms
(GT) quarantine
(GT) covid
(GT) fever
(GT) Fatigue
(GT) Sore throat

β	Training tp	Training fp	Training fn	Out of sample tp	Out of sample fp	Out of sample fn
1.00	0	0	3	0	0	1
0.99	0	0	3	0	0	1
0.96	0	0	3	0	0	1
0.91	2	2	1	1	0	0
0.76	3	5	0	1	0	0
0.46	3	13	0	1	5	0
0.00	3	34	0	1	8	0

Proxies selected by the Multiple Sources method

Model 1

(GT) covid
(GT) covid-19
(GT) covid symptoms
(GT) quarantine
(GT) Acute bronchitis
(GT) Anosmia

Model 2

(GT) covid symptoms
(GT) Anosmia
(GT) Asthma
(GT) Cough
(GT) Nasal congestion
(GT) Throat irritation

Model 3

(GT) Anosmia
(GT) Wheeze
(GT) Nasal congestion
(GT) covid
(GT) covid-19
(GT) quarantine

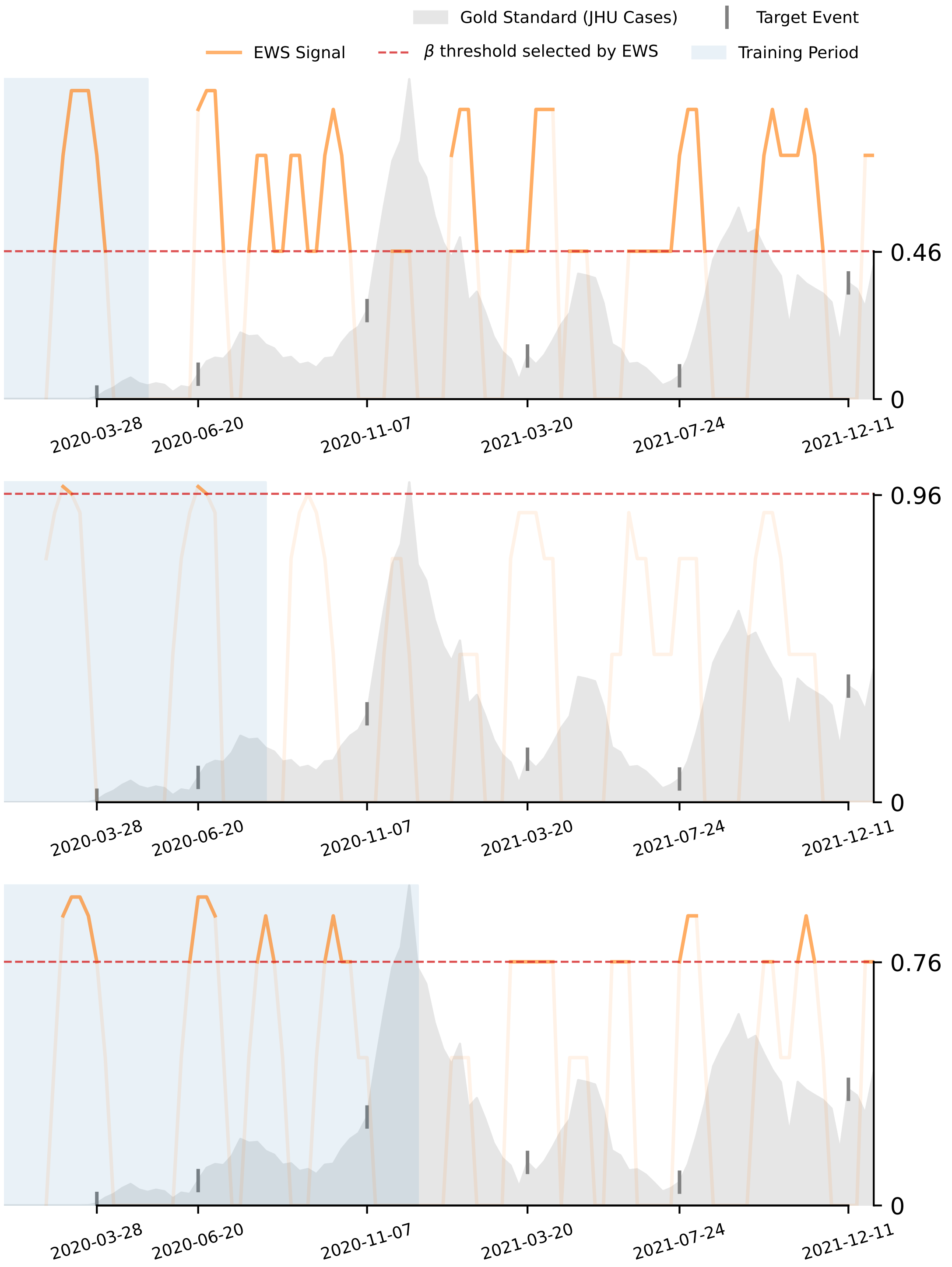

Performance as a function of β

Model 1

β	Training tp	Training fp	Training fn	Out of sample tp	Out of sample fp	Out of sample fn
1.00	0	0	1	0	0	5
0.99	0	0	1	0	0	5
0.96	0	0	1	0	2	4
0.91	1	0	0	3	10	0
0.76	1	0	0	4	20	0
0.46	1	0	0	5	33	0
0.00	1	2	0	5	52	0

Model 2

β	Training tp	Training fp	Training fn	Out of sample tp	Out of sample fp	Out of sample fn
1.00	0	0	2	0	0	4
0.99	0	0	2	0	0	4
0.96	2	0	0	0	1	4
0.91	2	1	0	3	5	1
0.76	2	3	0	3	14	1
0.46	2	4	0	4	22	0
0.00	2	4	0	4	44	0

Model 3

β	Training tp	Training fp	Training fn	Out of sample tp	Out of sample fp	Out of sample fn
1.00	0	0	3	0	0	3
0.99	0	0	3	0	0	3
0.96	0	0	3	0	0	3
0.91	2	1	1	1	2	1
0.76	3	3	0	3	11	0
0.46	3	6	0	3	21	0
0.00	3	12	0	3	33	0

Proxies selected by the Multiple Sources method

Model 1

(GT) covid
(GT) how long does covid last
(GT) covid-19
(GT) chest pain
(GT) covid symptoms
(GT) chest tightness

Model 2

(GT) covid
(GT) covid-19
(GT) chest pain
(GT) covid symptoms
(GT) chest tightness
(GT) quarantine

Model 3

(GT) covid-19
(GT) covid symptoms
(GT) chest tightness
(GT) quarantine
(GT) Anosmia
(GT) Burning Chest Pain

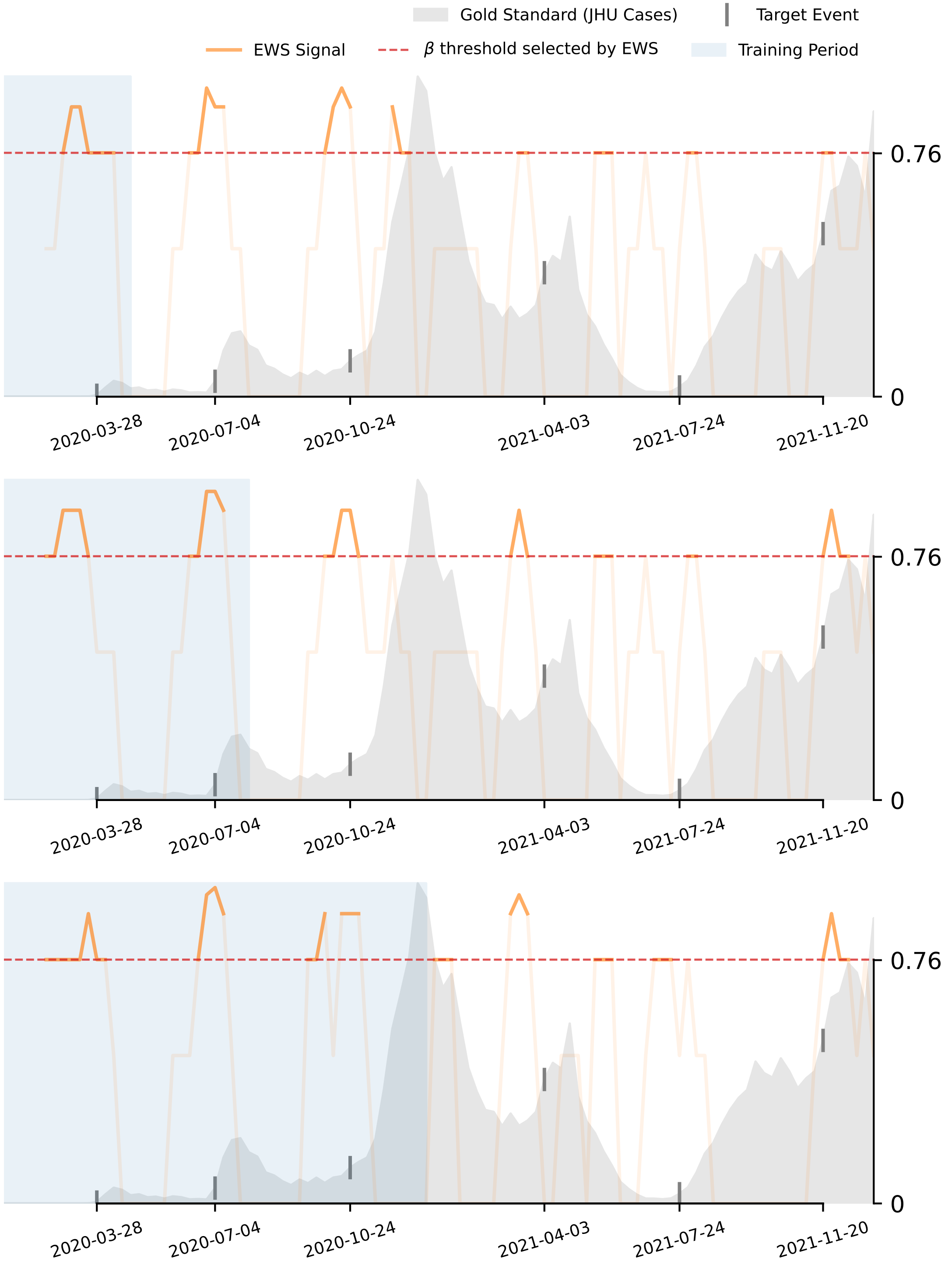

Performance as a function of β

Model 1

β	Training tp	Training fp	Training fn	Out of sample tp	Out of sample fp	Out of sample fn
1.00	0	0	1	0	0	5
0.99	0	0	1	0	0	5
0.96	0	0	1	2	0	3
0.91	0	0	1	2	2	3
0.76	1	0	0	5	11	0
0.46	1	2	0	5	28	0
0.00	1	4	0	5	53	0

Model 2

β	Training tp	Training fp	Training fn	Out of sample tp	Out of sample fp	Out of sample fn
1.00	0	0	2	0	0	4
0.99	0	0	2	0	0	4
0.96	0	0	2	0	0	4
0.91	1	0	1	2	1	1
0.76	2	1	0	4	11	0
0.46	2	3	0	4	26	0
0.00	2	7	0	4	46	0

Model 3

β	Training tp	Training fp	Training fn	Out of sample tp	Out of sample fp	Out of sample fn
1.00	0	0	3	0	0	3
0.99	0	0	3	0	0	3
0.96	1	0	2	1	0	2
0.91	1	0	2	1	1	1
0.76	3	2	0	3	11	0
0.46	3	6	0	3	18	0
0.00	3	10	0	3	32	0

PA, Philadelphia

Proxies selected by the Multiple Sources method

Model 1

(GT) covid
(GT) how long does covid last
(GT) covid-19
(GT) covid symptoms
(GT) covid-19 who
(GT) sars-cov-2

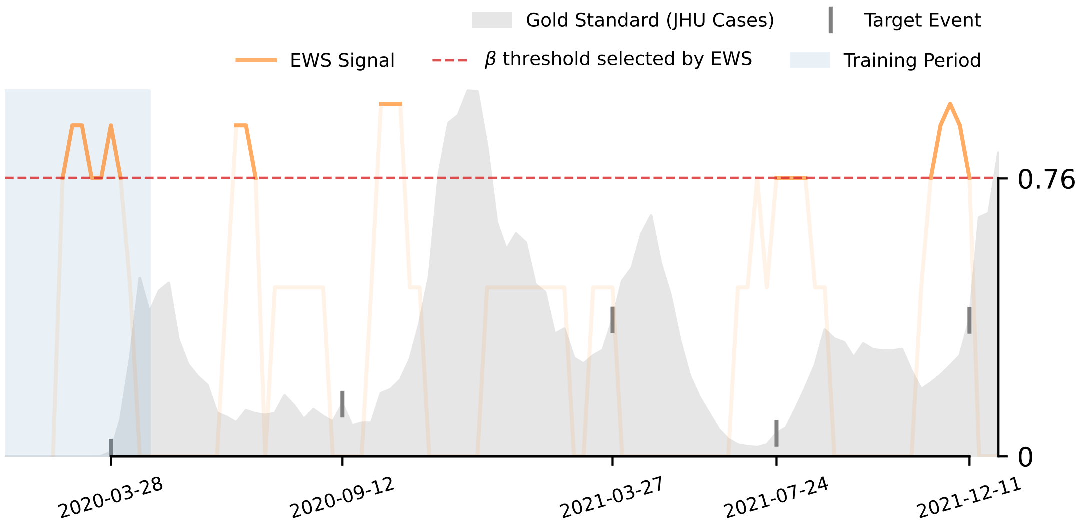

Performance as a function of β

β	Training tp	Training fp	Training fn	Out of sample tp	Out of sample fp	Out of sample fn
1.00	0	0	1	0	0	4
0.99	0	0	1	0	0	4
0.96	0	0	1	1	3	3
0.91	0	0	1	1	5	3
0.76	1	0	0	2	9	2
0.46	1	2	0	4	23	0
0.00	1	3	0	4	60	0

Model 2

(GT) sars-cov-2
(GT) Burning Chest Pain
(GT) Ageusia

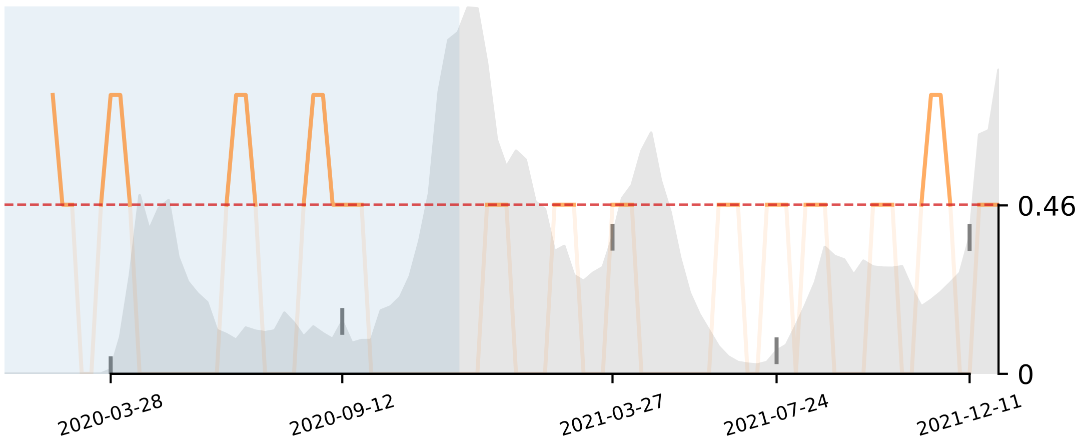

β	Training tp	Training fp	Training fn	Out of sample tp	Out of sample fp	Out of sample fn
0.91	0	0	2	0	0	3
0.76	0	0	2	1	0	2
0.46	2	4	0	3	15	0
0.00	2	10	0	3	35	0

Model 3

(GT) Burning Chest Pain
(GT) Ageusia
(GT) sars-cov-2
(GT) Nasal congestion
(GT) covid symptoms
(GT) nose bleed

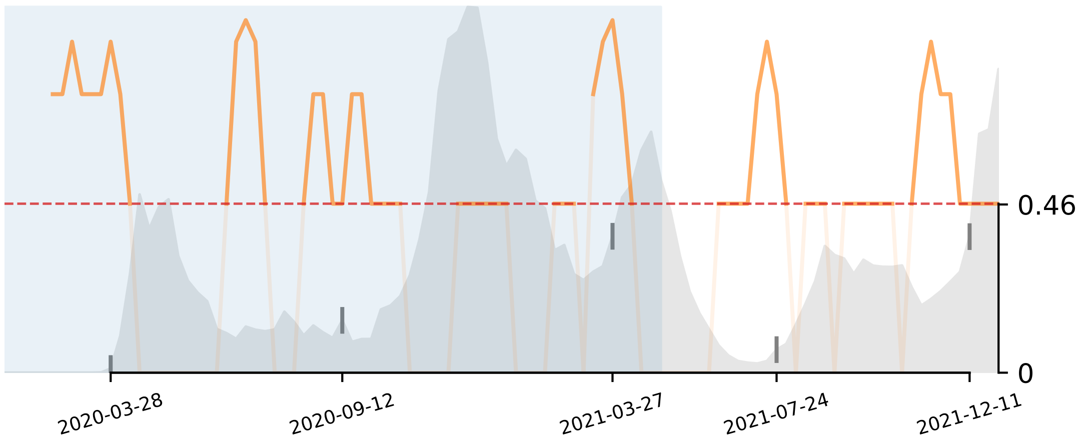

β	Training tp	Training fp	Training fn	Out of sample tp	Out of sample fp	Out of sample fn
1.00	0	0	3	0	0	2
0.99	0	0	3	0	0	2
0.96	0	0	3	0	0	2
0.91	1	1	2	2	0	0
0.76	2	3	1	2	0	0
0.46	3	9	0	2	13	0
0.00	3	25	0	2	21	0

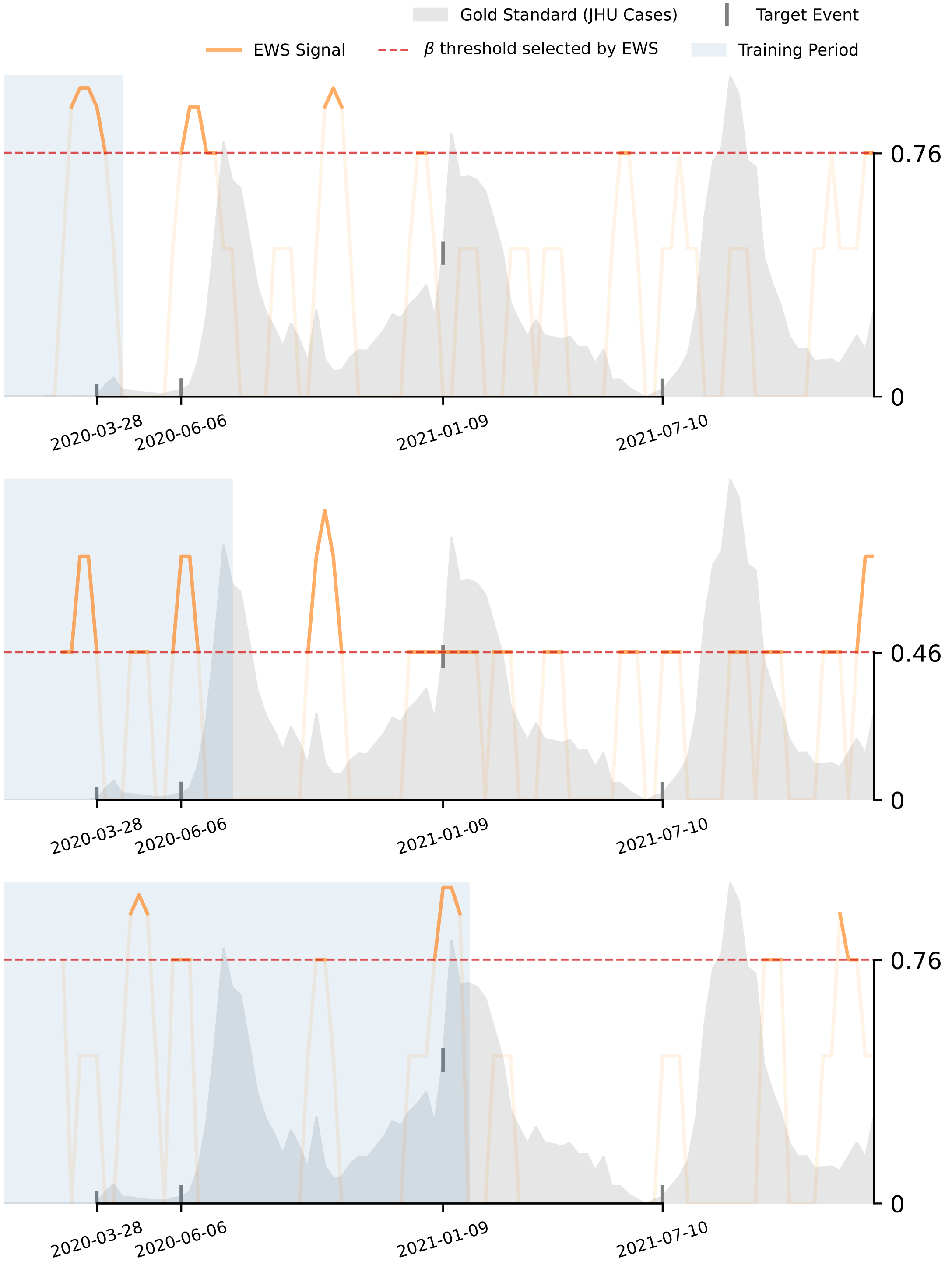

Proxies selected by the Multiple Sources method

Model 1

(GT) covid
(GT) covid-19
(GT) covid symptoms
(GT) quarantine
(GT) covid-19 who
(GT) fever

Model 2

(GT) covid symptoms
(GT) fever
Apple Mobility (transit)

Model 3

(GT) covid symptoms
Apple Mobility (transit)
(GT) Chronic pain
(GT) Diarrhea
(GT) Fatigue
Apple Mobility (driving)

Performance as a function of β

Model 1

β	Training tp	Training fp	Training fn	Out of sample tp	Out of sample fp	Out of sample fn
1.00	0	0	1	0	0	3
0.99	0	0	1	0	0	3
0.96	0	0	1	0	1	3
0.91	1	0	0	0	5	2
0.76	1	0	0	3	11	0
0.46	1	1	0	3	38	0
0.00	1	2	0	3	68	0

Model 2

β	Training tp	Training fp	Training fn	Out of sample tp	Out of sample fp	Out of sample fn
0.91	0	0	2	0	1	2
0.76	0	0	2	0	5	2
0.46	2	1	0	2	29	0
0.00	2	4	0	2	62	0

Model 3

β	Training tp	Training fp	Training fn	Out of sample tp	Out of sample fp	Out of sample fn
1.00	0	0	3	0	0	1
0.99	0	0	3	0	0	1
0.96	1	1	2	0	0	1
0.91	1	2	2	0	1	1
0.76	2	4	1	0	6	1
0.46	3	7	0	1	15	0
0.00	3	10	0	1	41	0

SC, Richland

Proxies selected by the Multiple Sources method

Model 1

(GT) covid
(GT) covid-19
(GT) covid symptoms
(GT) quarantine
up2date
(GT) Chest pain

Model 2

(GT) covid
(GT) covid symptoms
(GT) Chest pain
(GT) Chronic pain

Model 3

(GT) Chest pain
(GT) covid
(GT) covid symptoms
(GT) Chills
(GT) Chronic pain
(GT) Fatigue

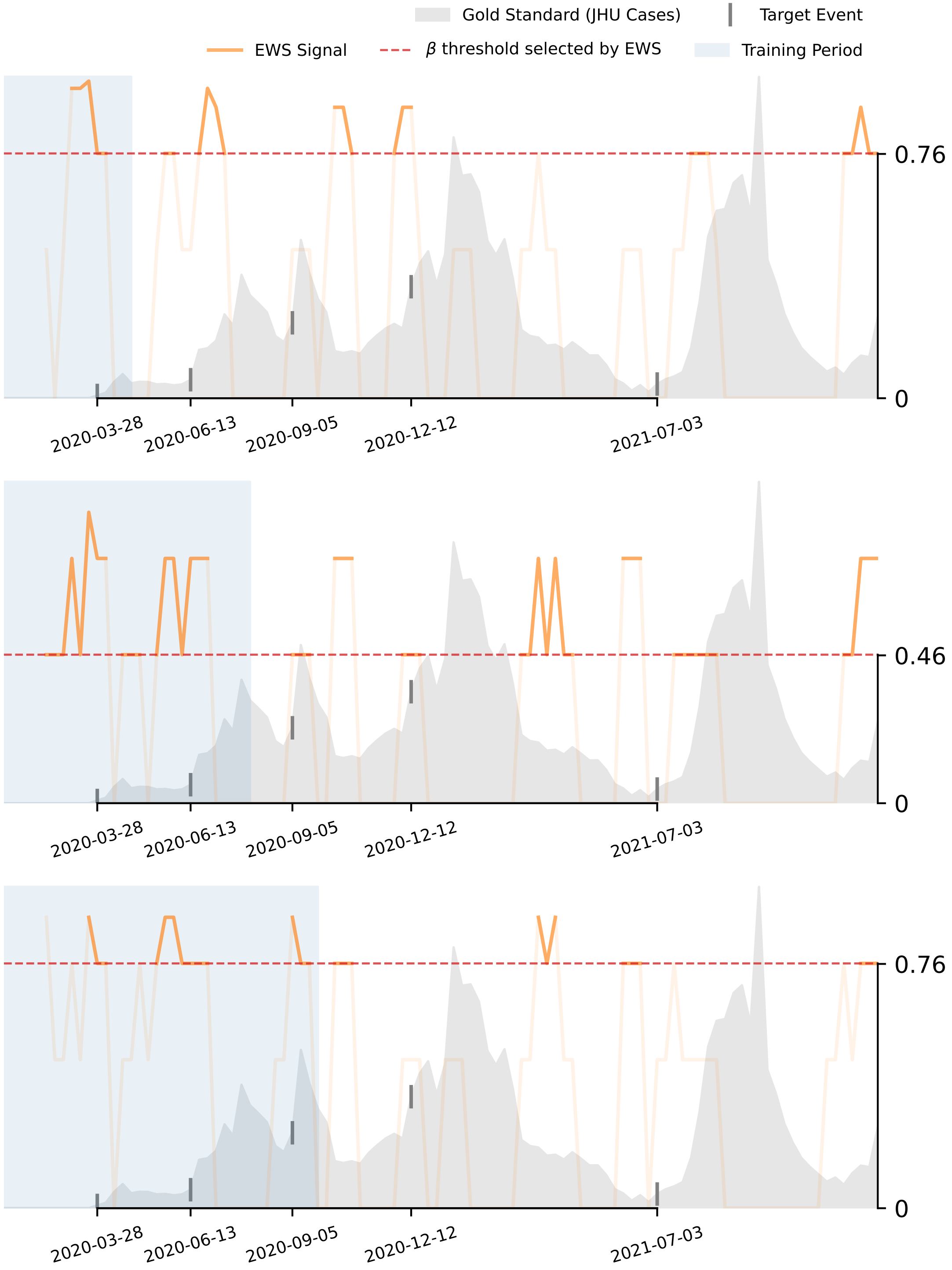

Performance as a function of β

Model 1

β	Training tp	Training fp	Training fn	Out of sample tp	Out of sample fp	Out of sample fn
1.00	0	0	1	0	0	4
0.99	0	0	1	0	0	4
0.96	1	0	0	0	1	3
0.91	1	0	0	1	5	2
0.76	1	0	0	2	16	2
0.46	1	1	0	4	30	0
0.00	1	2	0	4	60	0

Model 2

β	Training tp	Training fp	Training fn	Out of sample tp	Out of sample fp	Out of sample fn
0.96	0	0	2	0	0	3
0.91	0	0	2	0	0	3
0.76	1	0	1	1	8	2
0.46	2	3	0	3	24	0
0.00	2	8	0	3	55	0

Model 3

β	Training tp	Training fp	Training fn	Out of sample tp	Out of sample fp	Out of sample fn
1.00	0	0	3	0	0	2
0.99	0	0	3	0	0	2
0.96	0	0	3	0	0	2
0.91	0	0	3	0	2	2
0.76	3	1	0	1	11	1
0.46	3	7	0	2	28	0
0.00	3	11	0	2	52	0

Proxies selected by the Multiple Sources method

Model 1

(GT) covid
(GT) covid-19
(GT) covid symptoms
(GT) covid-19 who
(GT) fever
(GT) Diarrhea

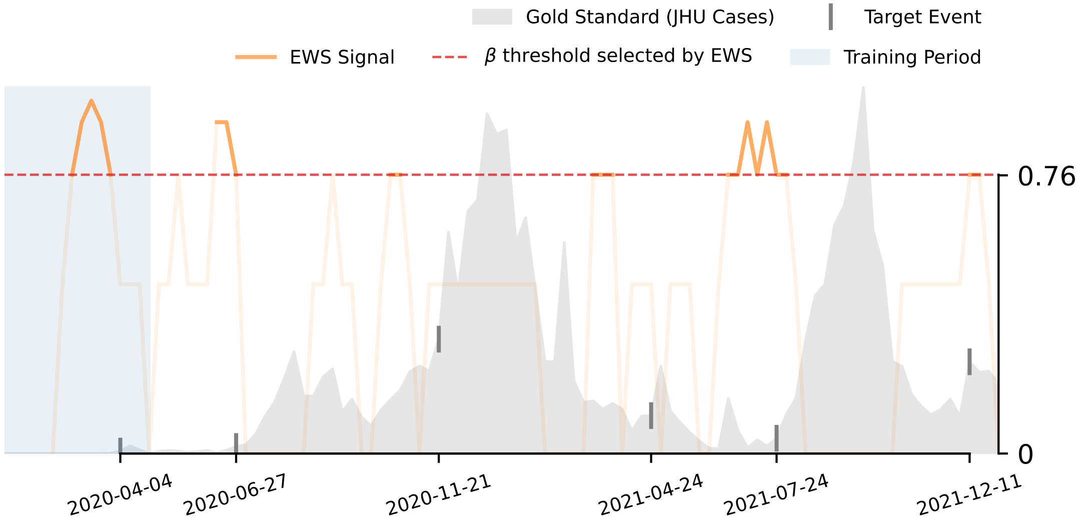

Performance as a function of β

β	Training tp	Training fp	Training fn	Out of sample tp	Out of sample fp	Out of sample fn
1.00	0	0	1	0	0	5
0.99	0	0	1	0	0	5
0.96	0	0	1	0	0	5
0.91	1	0	0	2	0	3
0.76	1	0	0	5	3	0
0.46	1	1	0	5	25	0
0.00	1	4	0	5	53	0

Model 2

(GT) covid
(GT) covid-19
(GT) covid symptoms
(GT) fever

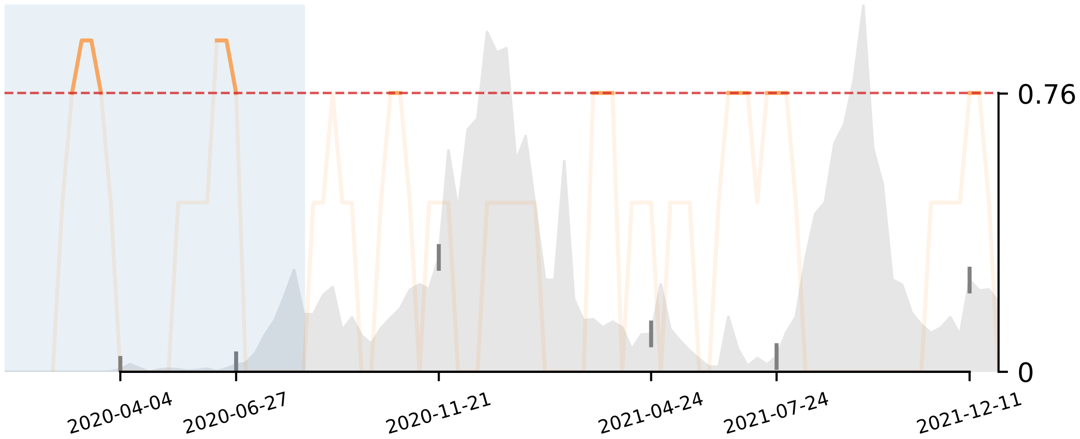

β	Training tp	Training fp	Training fn	Out of sample tp	Out of sample fp	Out of sample fn
0.96	0	0	2	0	0	4
0.91	0	0	2	0	0	4
0.76	2	0	0	4	3	0
0.46	2	1	0	4	19	0
0.00	2	4	0	4	44	0

Model 3

(GT) covid
(GT) covid symptoms
(GT) fever
(GT) covid-19
(GT) Abdominal pain
(GT) Fever

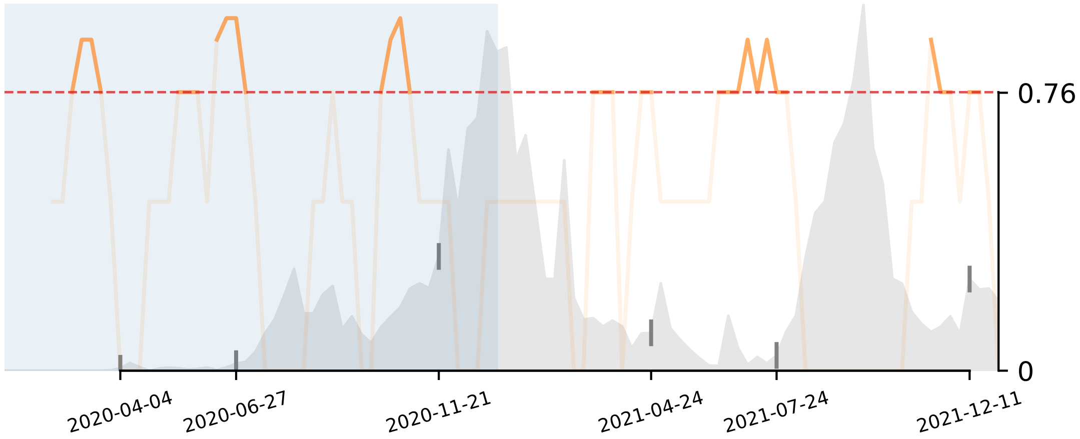

β	Training tp	Training fp	Training fn	Out of sample tp	Out of sample fp	Out of sample fn
1.00	0	0	3	0	0	3
0.99	0	0	3	0	0	3
0.96	0	0	3	0	0	3
0.91	2	0	1	2	0	1
0.76	3	1	0	3	2	0
0.46	3	7	0	3	17	0
0.00	3	20	0	3	31	0

TN, Davidson

Proxies selected by the Multiple Sources method

Model 1

(GT) covid
(GT) covid-19
(GT) covid symptoms
(GT) Acute bronchitis
(GT) Bronchitis
(GT) Chest pain

Model 2

(GT) covid
(GT) covid-19
(GT) covid symptoms
(GT) chest tightness
(GT) Acute bronchitis
(GT) Bronchitis

Model 3

(GT) covid symptoms
(GT) Acute bronchitis
(GT) Bronchitis
(GT) Cough
(GT) Nasal congestion
(GT) Pneumonia

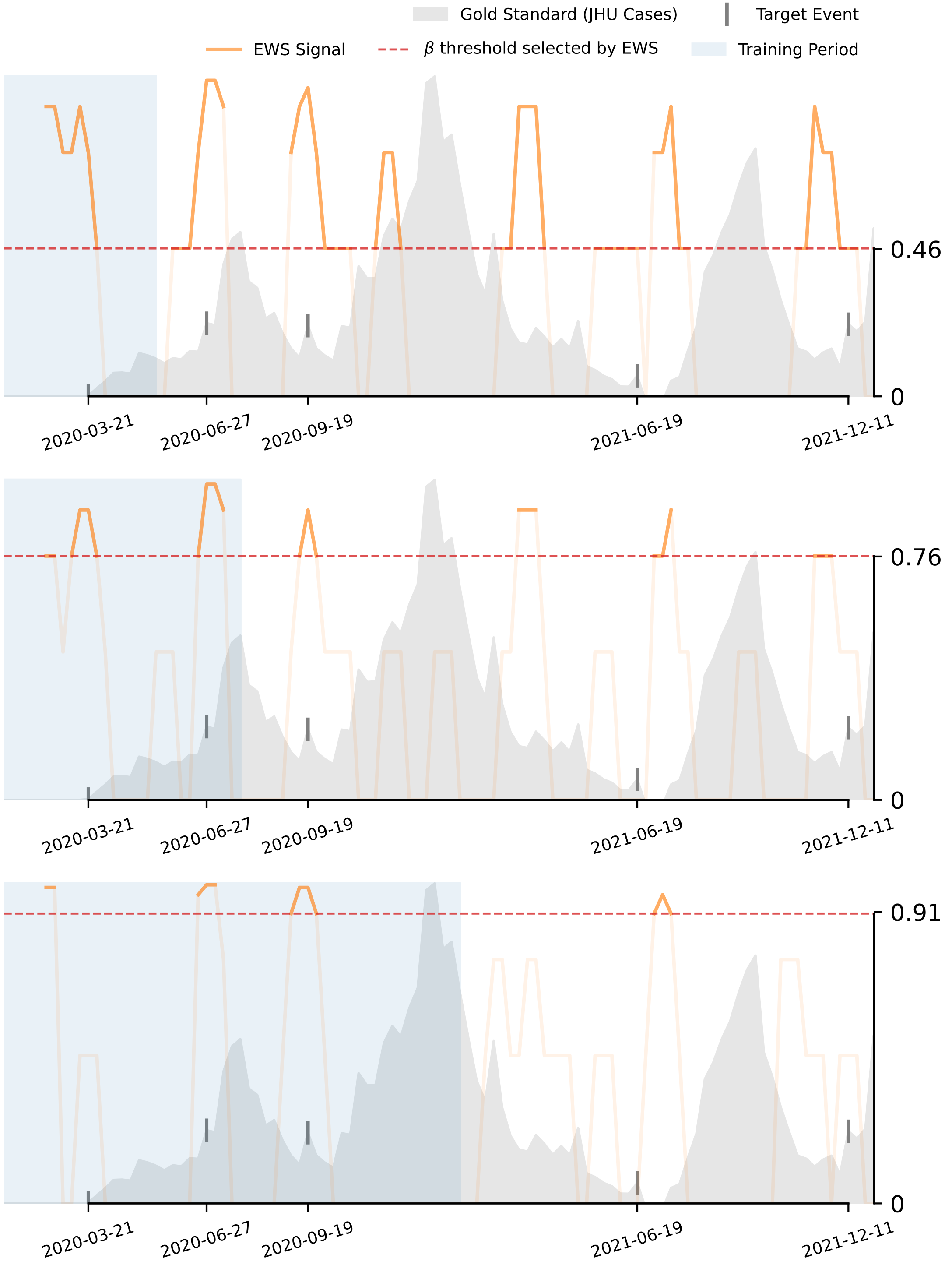

Performance as a function of β

Model 1

	Training			Out of sample		
β	tp	fp	fn	tp	fp	fn
1.00	0	0	1	0	0	4
0.99	0	0	1	1	1	3
0.96	0	0	1	2	1	2
0.91	0	0	1	3	6	1
0.76	1	1	0	3	11	0
0.46	1	1	0	4	23	0
0.00	1	2	0	4	58	0

Model 2

β	tp	fp	fn	tp	fp	fn
1.00	0	0	2	0	0	3
0.99	0	0	2	0	0	3
0.96	1	1	1	0	0	3
0.91	1	1	1	1	4	2
0.76	2	2	0	2	7	0
0.46	2	4	0	3	26	0
0.00	2	7	0	3	54	0

Model 3

β	tp	fp	fn	tp	fp	fn
1.00	0	0	3	0	0	2
0.99	1	1	2	0	0	2
0.96	3	2	0	0	1	2
0.91	3	2	0	0	3	1
0.76	3	3	0	1	9	0
0.46	3	4	0	2	19	0
0.00	3	6	0	2	35	0

TN, Knox

Proxies selected by the Multiple Sources method

Performance as a function of β

Model 1

(GT) covid
(GT) covid-19
(GT) covid symptoms
(GT) covid-19 who
(GT) fever
(GT) Chills

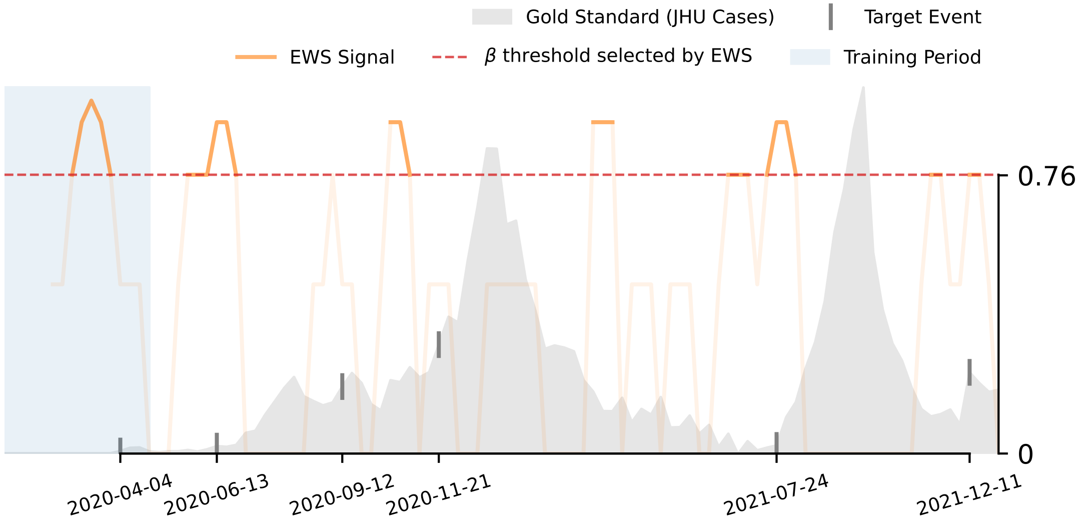

β	Training tp	Training fp	Training fn	Out of sample tp	Out of sample fp	Out of sample fn
1.00	0	0	1	0	0	5
0.99	0	0	1	0	0	5
0.96	0	0	1	0	0	5
0.91	1	0	0	3	5	2
0.76	1	0	0	5	8	0
0.46	1	1	0	5	23	0
0.00	1	5	0	5	53	0

Model 2

(GT) covid
(GT) covid-19
(GT) covid symptoms
(GT) fever
(GT) Chills

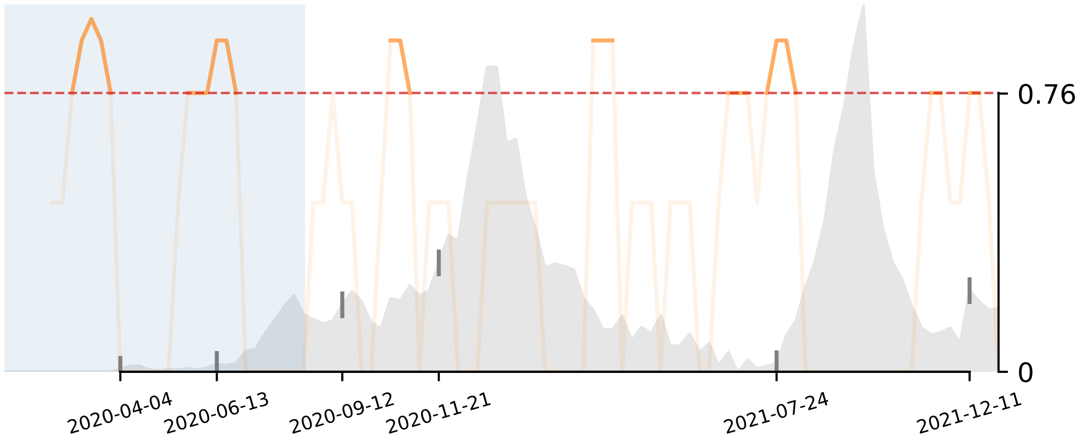

β	Training tp	Training fp	Training fn	Out of sample tp	Out of sample fp	Out of sample fn
0.99	0	0	2	0	0	4
0.96	0	0	2	0	0	4
0.91	1	0	1	2	4	2
0.76	2	1	0	4	6	0
0.46	2	3	0	4	21	0
0.00	2	5	0	4	47	0

Model 3

(GT) covid symptoms
(GT) fever
(GT) covid-19
(GT) Diarrhea
(GT) covid
(GT) chest pain

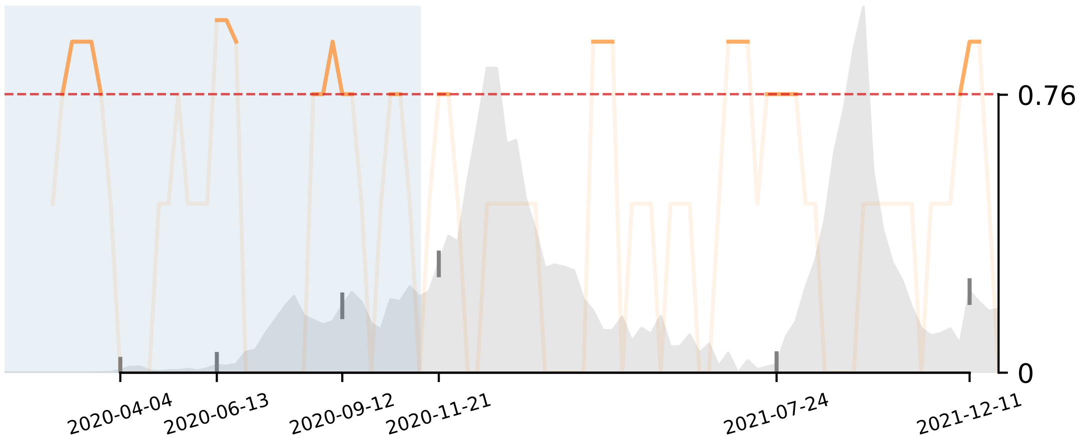

β	Training tp	Training fp	Training fn	Out of sample tp	Out of sample fp	Out of sample fn
1.00	0	0	3	0	0	3
0.99	0	0	3	0	0	3
0.96	0	0	3	0	0	3
0.91	1	1	2	2	4	1
0.76	3	3	0	3	7	0
0.46	3	7	0	3	28	0
0.00	3	13	0	3	44	0

Proxies selected by the Multiple Sources method

Model 1

(GT) covid
(GT) covid symptoms
(GT) Diarrhea
(GT) Dizziness
(GT) Fatigue
(GT) Nasal congestion

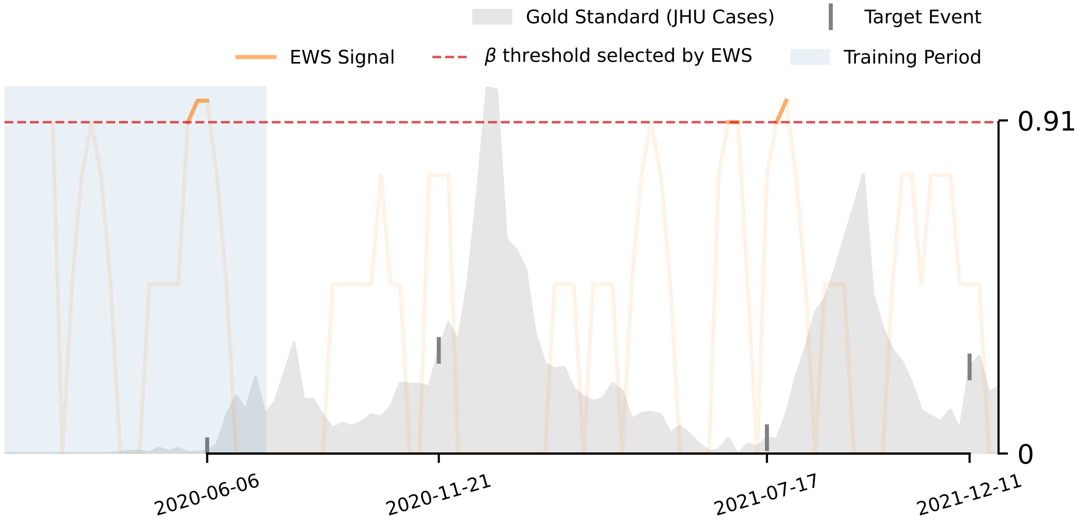

Performance as a function of β

β	Training tp	Training fp	Training fn	Out of sample tp	Out of sample fp	Out of sample fn
1.00	0	0	1	0	0	3
0.99	0	0	1	0	0	3
0.96	0	0	1	0	1	2
0.91	1	0	0	1	3	2
0.76	1	2	0	3	8	0
0.46	1	5	0	3	27	0
0.00	1	10	0	3	55	0

Model 2

(GT) covid
(GT) Vomiting
(GT) covid symptoms
(GT) Abdominal pain
(GT) Fatigue

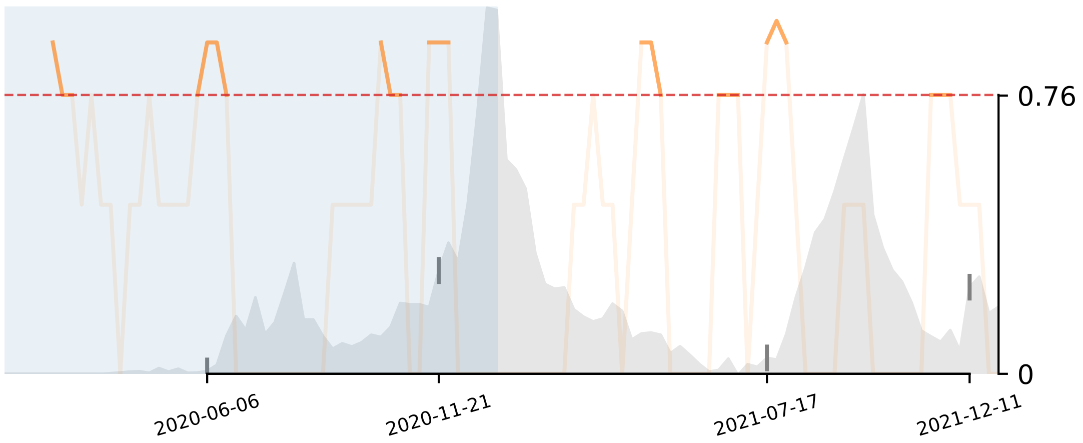

β	Training tp	Training fp	Training fn	Out of sample tp	Out of sample fp	Out of sample fn
0.99	0	0	2	0	0	2
0.96	0	0	2	0	1	1
0.91	0	0	2	1	4	1
0.76	2	4	0	2	6	0
0.46	2	10	0	2	16	0
0.00	2	21	0	2	38	0

Model 3

(GT) covid
(GT) Abdominal pain
(GT) Fatigue
(GT) Vomiting
(GT) covid symptoms
(GT) Diarrhea

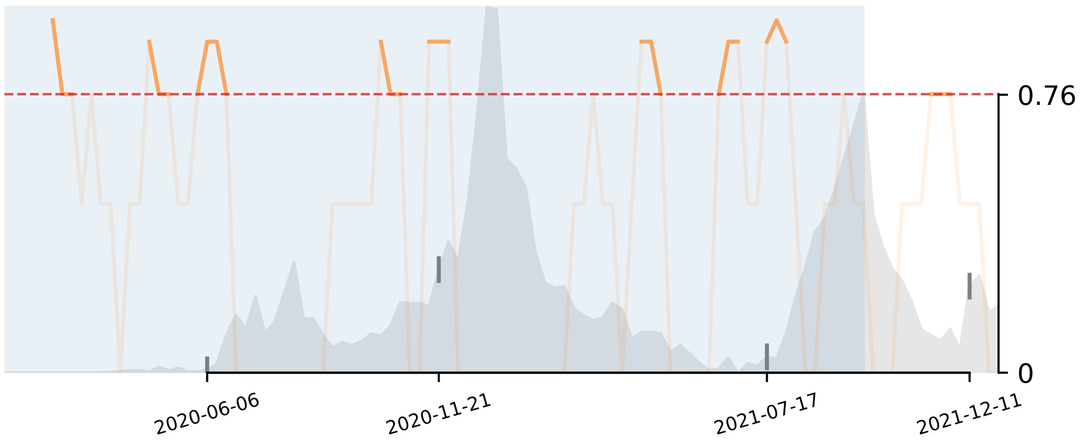

β	Training tp	Training fp	Training fn	Out of sample tp	Out of sample fp	Out of sample fn
1.00	0	0	3	0	0	1
0.99	0	0	3	0	0	1
0.96	0	0	3	0	0	1
0.91	0	2	3	0	0	1
0.76	3	9	0	1	0	0
0.46	3	19	0	1	2	0
0.00	3	39	0	1	7	0

Proxies selected by the Multiple Sources method

Model 1

(GT) covid
(GT) covid-19
(GT) chest pain
(GT) covid symptoms
(GT) quarantine
(GT) fever

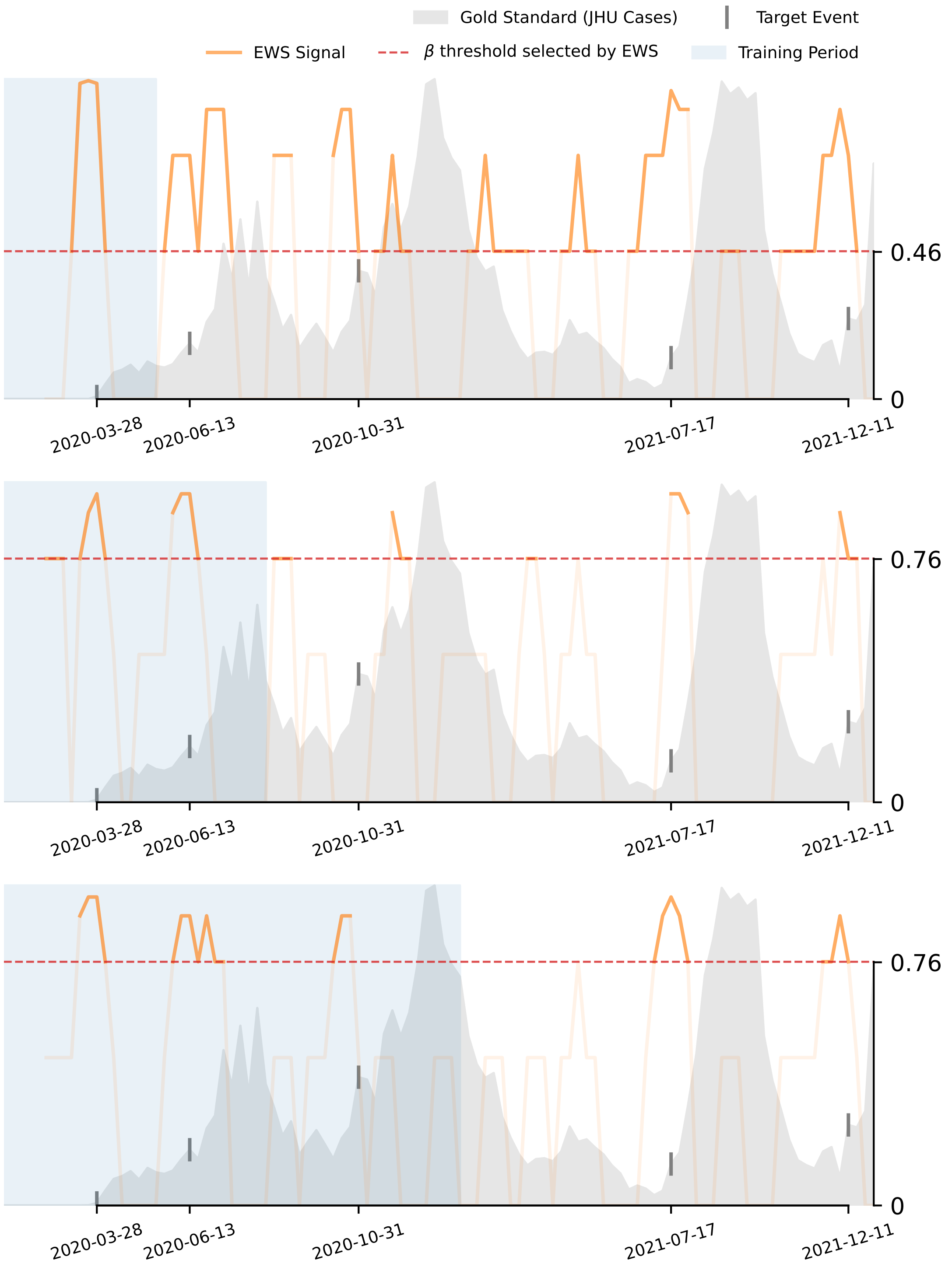

Performance as a function of β

β	Training tp	Training fp	Training fn	Out of sample tp	Out of sample fp	Out of sample fn
1.00	0	0	1	0	0	4
0.99	1	0	0	0	0	4
0.96	1	0	0	1	0	3
0.91	1	0	0	3	5	0
0.76	1	0	0	4	11	0
0.46	1	0	0	4	34	0
0.00	1	1	0	4	60	0

Model 2

(GT) covid-19
(GT) chest pain
(GT) fever
(GT) Ageusia
(GT) Diarrhea
(GT) Fatigue

β	Training tp	Training fp	Training fn	Out of sample tp	Out of sample fp	Out of sample fn
1.00	0	0	2	0	0	3
0.99	0	0	2	0	0	3
0.96	0	0	2	1	1	2
0.91	2	0	0	2	3	1
0.76	2	0	0	2	12	1
0.46	2	4	0	3	28	0
0.00	2	8	0	3	51	0

Model 3

(GT) Ageusia
(GT) Otitis
(GT) covid
(GT) covid-19
(GT) chest pain
(GT) quarantine

β	Training tp	Training fp	Training fn	Out of sample tp	Out of sample fp	Out of sample fn
1.00	0	0	3	0	0	2
0.99	0	0	3	0	0	2
0.96	0	0	3	1	0	1
0.91	1	0	2	2	1	0
0.76	3	1	0	2	3	0
0.46	3	5	0	2	19	0
0.00	3	19	0	2	35	0

Proxies selected by the Multiple Sources method

Model 1

(GT) covid
(GT) covid-19
(GT) chest pain
(GT) covid symptoms
(GT) quarantine
(GT) fever

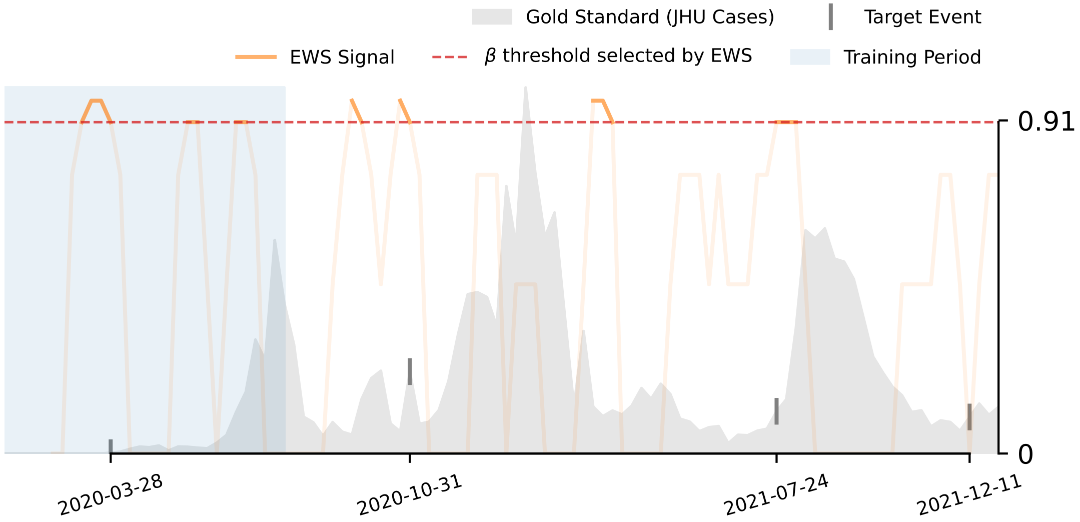

Model 2

(GT) covid
(GT) covid symptoms
(GT) Ageusia
(GT) Anosmia
(GT) Asphyxia
(GT) Asthma

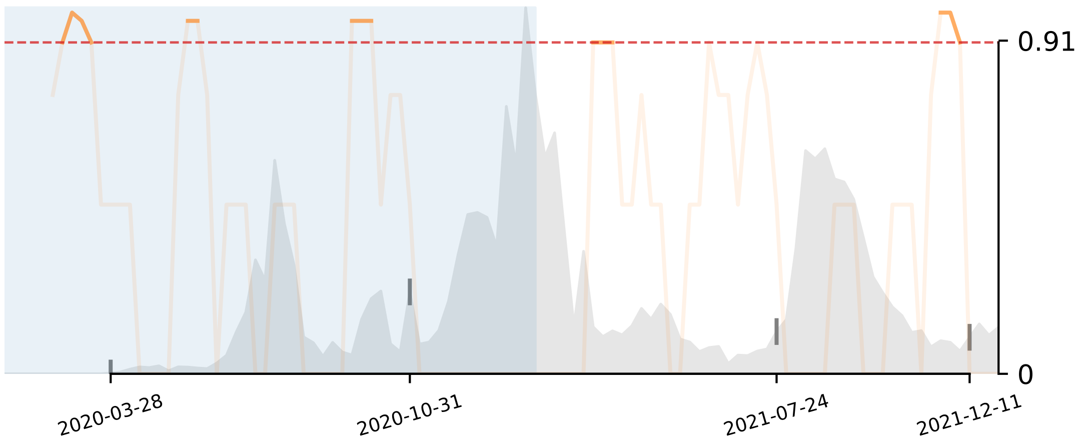

Model 3

(GT) covid
(GT) covid symptoms
(GT) quarantine
(GT) Asthma
(GT) covid-19
(GT) Otitis

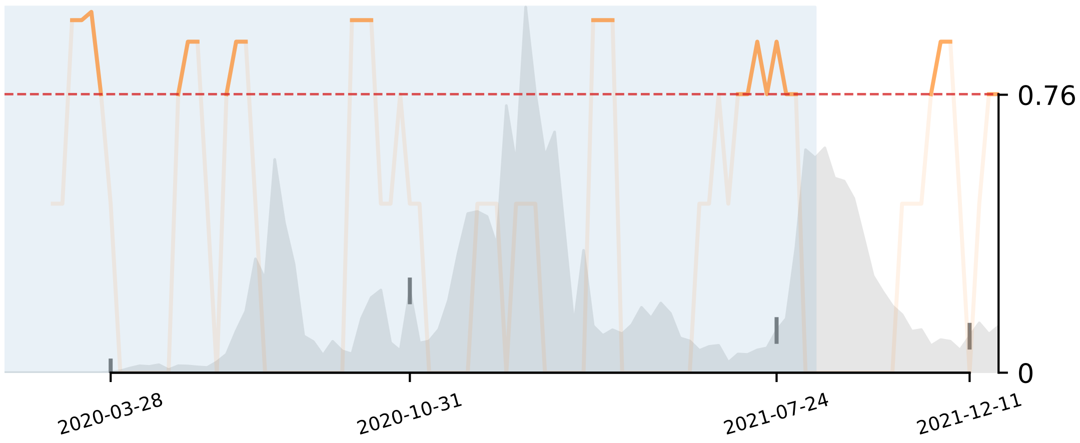

Performance as a function of β

Model 1

	Training			Out of sample		
β	tp	fp	fn	tp	fp	fn
1.00	0	0	1	0	0	3
0.99	0	0	1	0	0	3
0.96	0	0	1	1	2	2
0.91	1	0	0	2	5	1
0.76	1	4	0	3	15	0
0.46	1	7	0	3	25	0
0.00	1	9	0	3	53	0

Model 2

β	tp	fp	fn	tp	fp	fn
1.00	0	0	2	0	0	2
0.99	0	0	2	1	0	1
0.96	1	0	1	1	0	1
0.91	2	4	0	2	4	0
0.76	2	5	0	2	5	0
0.46	2	8	0	2	16	0
0.00	2	16	0	2	34	0

Model 3

β	tp	fp	fn	tp	fp	fn
1.00	0	0	3	0	0	1
0.99	0	0	3	0	0	1
0.96	1	0	2	0	0	1
0.91	2	5	1	1	0	0
0.76	3	9	0	1	2	0
0.46	3	14	0	1	4	0
0.00	3	28	0	1	12	0

TX, Collin

Proxies selected by the Multiple Sources method

Model 1

(GT) covid
(GT) covid-19
(GT) loss taste
(GT) chest pain
(GT) covid symptoms
(GT) sars-cov-2

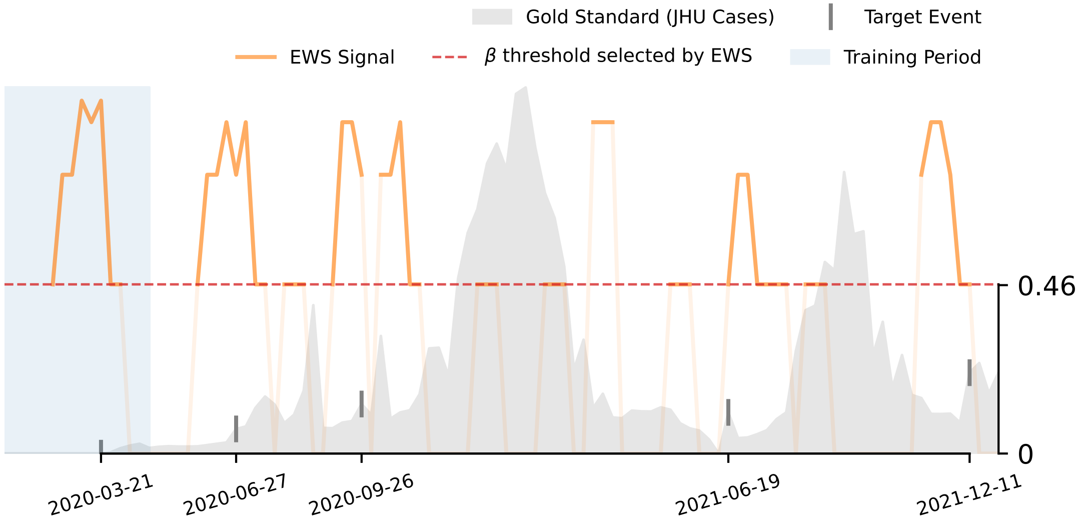

Model 2

(GT) covid
(GT) covid-19
(GT) chest pain
(GT) covid symptoms
(GT) Acute bronchitis
(GT) Anosmia

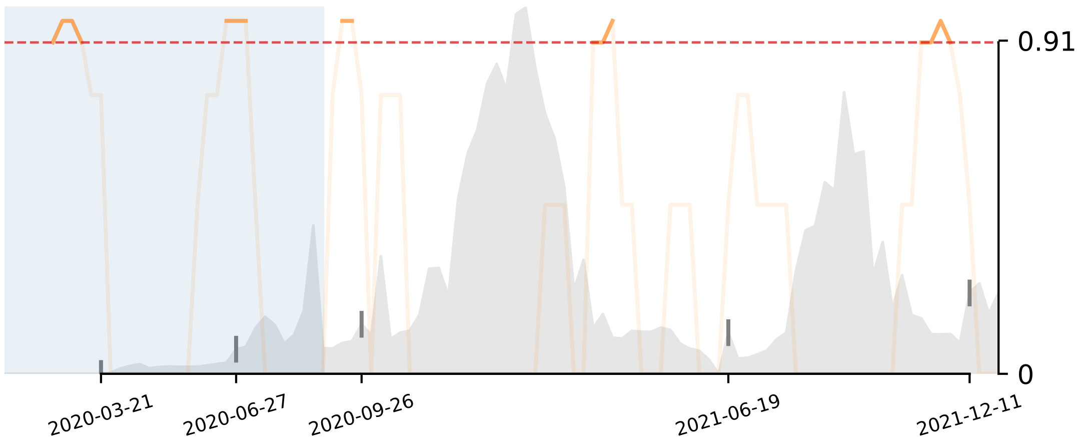

Model 3

(GT) chest pain
(GT) covid symptoms
(GT) Acute bronchitis
(GT) Anosmia
(GT) Bronchitis
(GT) Chills

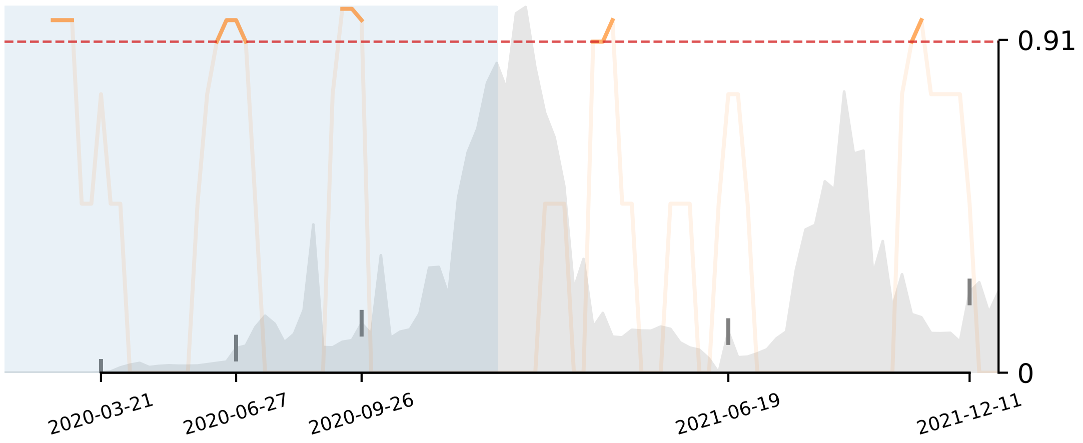

Performance as a function of β

Model 1

β	Training tp	Training fp	Training fn	Out of sample tp	Out of sample fp	Out of sample fn
1.00	0	0	1	0	0	4
0.99	0	0	1	0	0	4
0.96	0	0	1	0	0	4
0.91	1	0	0	3	5	1
0.76	1	0	0	3	9	0
0.46	1	0	0	4	28	0
0.00	1	3	0	4	60	0

Model 2

β	Training tp	Training fp	Training fn	Out of sample tp	Out of sample fp	Out of sample fn
1.00	0	0	2	0	0	3
0.99	0	0	2	0	0	3
0.96	0	0	2	2	1	1
0.91	2	1	0	2	3	1
0.76	2	2	0	2	8	0
0.46	2	2	0	3	18	0
0.00	2	3	0	3	52	0

Model 3

β	Training tp	Training fp	Training fn	Out of sample tp	Out of sample fp	Out of sample fn
1.00	0	0	3	0	0	2
0.99	1	0	2	0	0	2
0.96	1	0	2	1	1	1
0.91	3	1	0	1	3	1
0.76	3	2	0	2	5	0
0.46	3	2	0	2	11	0
0.00	3	5	0	2	38	0

Proxies selected by the Multiple Sources method

Performance as a function of β

Model 1

(GT) sars-cov-2
(GT) Hyperventilation
(GT) covid
(GT) covid-19
(GT) loss taste
(GT) chest pain

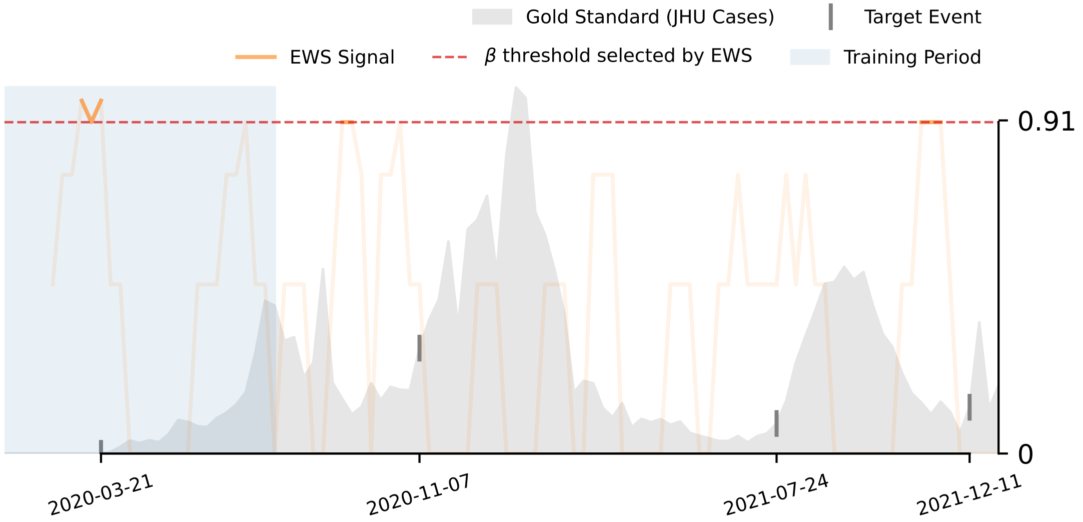

β	Training tp	Training fp	Training fn	Out of sample tp	Out of sample fp	Out of sample fn
1.00	0	0	1	0	0	3
0.99	0	0	1	0	0	3
0.96	0	0	1	0	0	3
0.91	1	0	0	2	2	1
0.76	1	1	0	3	7	0
0.46	1	3	0	3	24	0
0.00	1	11	0	3	54	0

Model 2

(GT) covid
(GT) covid-19
(GT) Viral pneumonia
(GT) loss taste
(GT) Burning Chest Pain

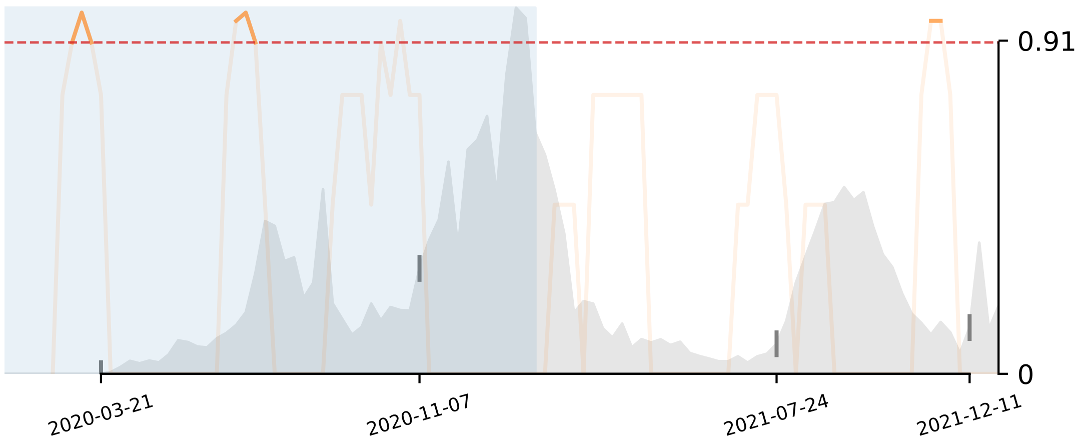

β	Training tp	Training fp	Training fn	Out of sample tp	Out of sample fp	Out of sample fn
0.99	0	0	2	0	0	2
0.96	1	1	1	1	0	1
0.91	2	2	0	1	0	1
0.76	2	3	0	2	6	0
0.46	2	7	0	2	13	0
0.00	2	10	0	2	34	0

Model 3

(GT) covid
(GT) covid-19
(GT) Burning Chest Pain
(GT) loss smell
(GT) loss of smell
(GT) Hyperventilation

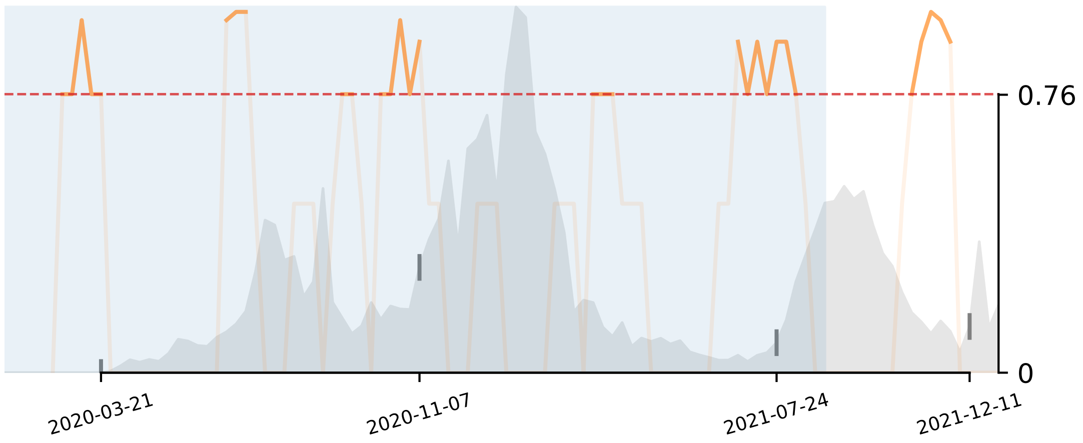

β	Training tp	Training fp	Training fn	Out of sample tp	Out of sample fp	Out of sample fn
1.00	0	0	3	0	0	1
0.99	0	0	3	1	0	0
0.96	0	2	3	1	0	0
0.91	2	3	1	1	0	0
0.76	3	4	0	1	0	0
0.46	3	10	0	1	1	0
0.00	3	30	0	1	11	0

Proxies selected by the Multiple Sources method

Model 1

(GT) covid
(GT) covid-19
(GT) chest pain
(GT) covid symptoms
(GT) quarantine
(GT) fever

Model 2

(GT) covid symptoms
(GT) fever
(GT) Ageusia
(GT) Anosmia
(GT) Chills
(GT) Cough

Model 3

(GT) covid symptoms
(GT) fever
(GT) Ageusia
(GT) Anosmia
(GT) Cough
(GT) Diarrhea

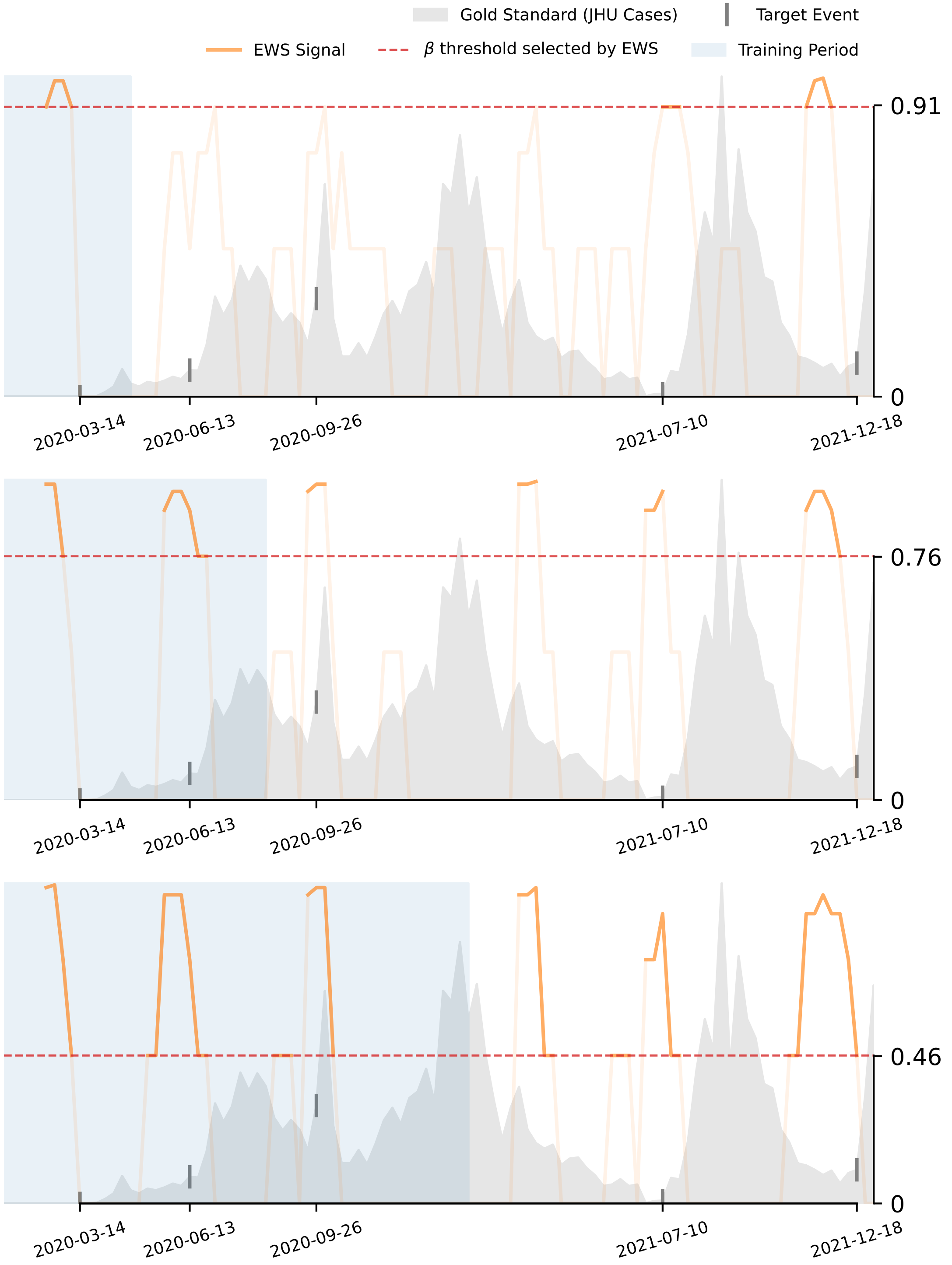

Performance as a function of β

Model 1

	Training			Out of sample		
β	tp	fp	fn	tp	fp	fn
1.00	0	0	1	1	0	3
0.99	0	0	1	1	0	3
0.96	1	0	0	1	0	3
0.91	1	0	0	2	5	1
0.76	1	0	0	4	11	0
0.46	1	0	0	4	34	0
0.00	1	0	0	4	60	0

Model 2

β	tp	fp	fn	tp	fp	fn
1.00	0	0	2	0	1	3
0.99	0	0	2	1	4	2
0.96	1	0	1	3	4	0
0.91	2	0	0	3	4	0
0.76	2	0	0	3	4	0
0.46	2	2	0	3	13	0
0.00	2	2	0	3	52	0

Model 3

β	tp	fp	fn	tp	fp	fn
1.00	0	0	3	0	0	2
0.99	1	0	2	0	1	2
0.96	2	1	1	1	3	1
0.91	3	1	0	2	3	0
0.76	3	1	0	2	3	0
0.46	3	1	0	2	9	0
0.00	3	6	0	2	34	0

Proxies selected by the Multiple Sources method

Model 1

(GT) covid
(GT) covid-19
(GT) loss taste
(GT) covid symptoms
(GT) sars-cov-2
(GT) Anosmia

Model 2

(GT) covid
(GT) covid-19
(GT) chest pain
(GT) covid symptoms
(GT) Ageusia
(GT) Anosmia

Model 3

(GT) chest pain
(GT) covid symptoms
(GT) Ageusia
(GT) Anosmia
(GT) Hyperventilation
(GT) Chills

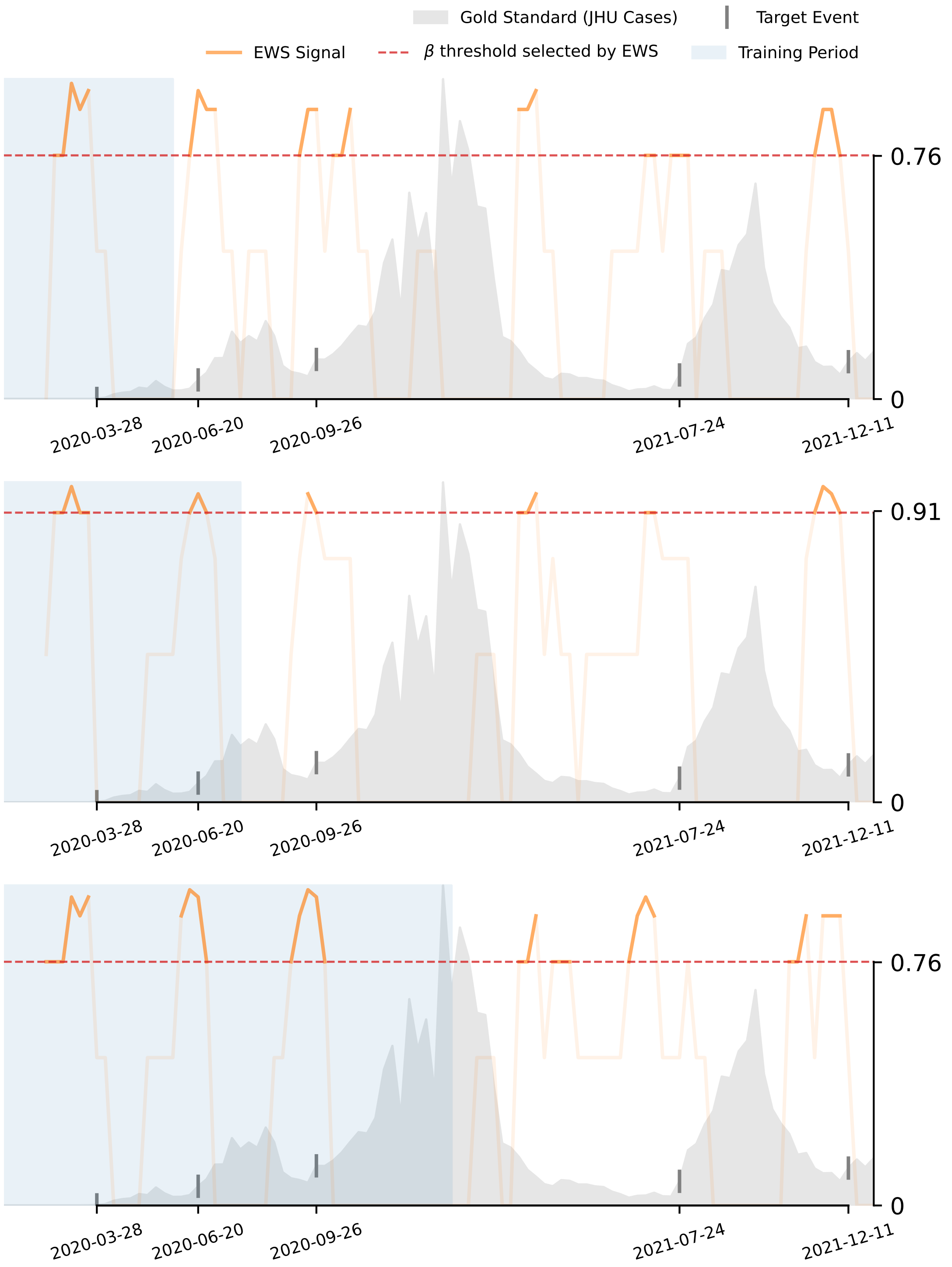

Performance as a function of β

Model 1

	Training			Out of sample		
β	tp	fp	fn	tp	fp	fn
1.00	0	0	1	0	0	4
0.99	0	0	1	0	0	4
0.96	1	0	0	1	1	3
0.91	1	0	0	3	6	1
0.76	1	0	0	4	9	0
0.46	1	1	0	4	26	0
0.00	1	2	0	4	59	0

Model 2

β	tp	fp	fn	tp	fp	fn
1.00	0	0	2	0	0	3
0.99	0	0	2	1	0	2
0.96	1	0	1	2	1	1
0.91	2	0	0	3	3	0
0.76	2	2	0	3	9	0
0.46	2	3	0	3	20	0
0.00	2	6	0	3	54	0

Model 3

β	tp	fp	fn	tp	fp	fn
1.00	0	0	3	0	0	2
0.99	0	0	3	0	0	2
0.96	2	0	1	1	0	1
0.91	3	0	0	2	1	0
0.76	3	0	0	2	8	0
0.46	3	4	0	2	20	0
0.00	3	8	0	2	36	0

Proxies selected by the Multiple Sources method

Model 1

(GT) covid
(GT) covid-19
(GT) covid symptoms
(GT) quarantine
(GT) covid-19 who
(GT) fever

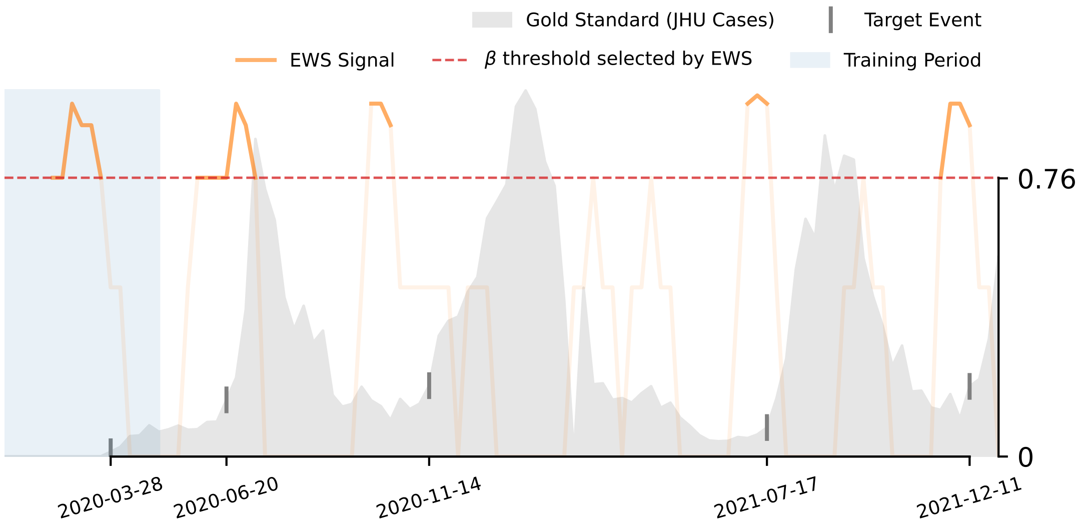

Performance as a function of β

β	Training tp	Training fp	Training fn	Out of sample tp	Out of sample fp	Out of sample fn
1.00	0	0	1	0	0	4
0.99	0	0	1	1	0	3
0.96	0	0	1	3	1	0
0.91	1	0	0	3	2	0
0.76	1	0	0	4	6	0
0.46	1	2	0	4	27	0
0.00	1	3	0	4	59	0

Model 2

(GT) covid
(GT) covid-19
(GT) covid symptoms
(GT) covid-19 who
(GT) Acute bronchitis
(GT) Anosmia

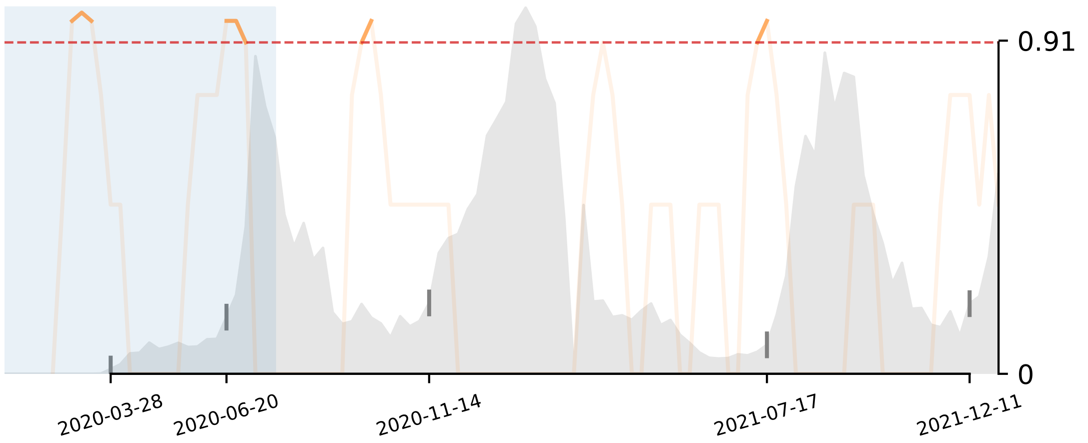

β	Training tp	Training fp	Training fn	Out of sample tp	Out of sample fp	Out of sample fn
1.00	0	0	2	0	0	3
0.99	0	0	2	0	0	3
0.96	1	0	1	2	0	1
0.91	2	1	0	2	2	1
0.76	2	2	0	3	7	0
0.46	2	2	0	3	21	0
0.00	2	4	0	3	54	0

Model 3

(GT) covid
(GT) covid-19
(GT) covid-19 who
(GT) covid symptoms
(GT) quarantine
(GT) Acute bronchitis

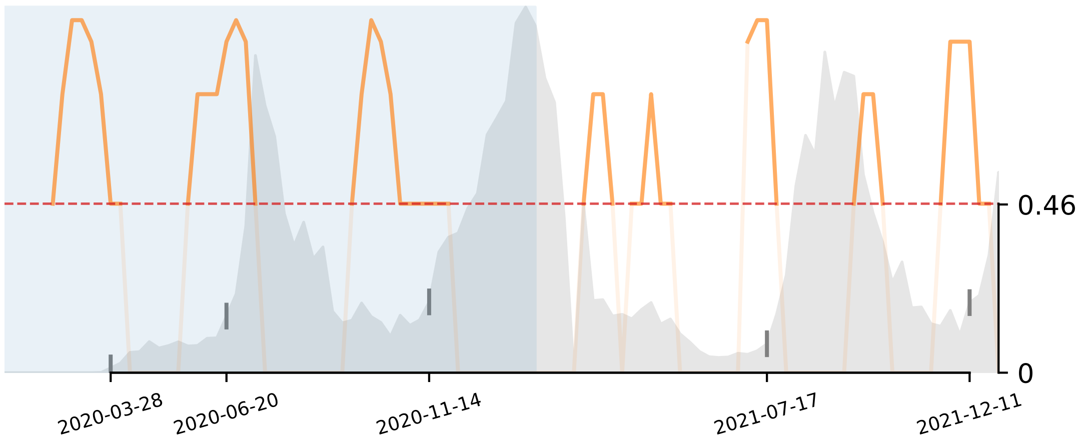

β	Training tp	Training fp	Training fn	Out of sample tp	Out of sample fp	Out of sample fn
1.00	0	0	3	0	0	2
0.99	0	0	3	0	0	2
0.96	0	0	3	1	0	1
0.91	1	2	2	2	0	0
0.76	2	4	1	2	5	0
0.46	3	6	0	2	16	0
0.00	3	12	0	2	34	0

Proxies selected by the Multiple Sources method

Performance as a function of β

Model 1

(GT) Abdominal pain
(GT) Chest pain
(GT) Nasal congestion
(GT) Vertigo
(GT) Cough
(GT) Diarrhea

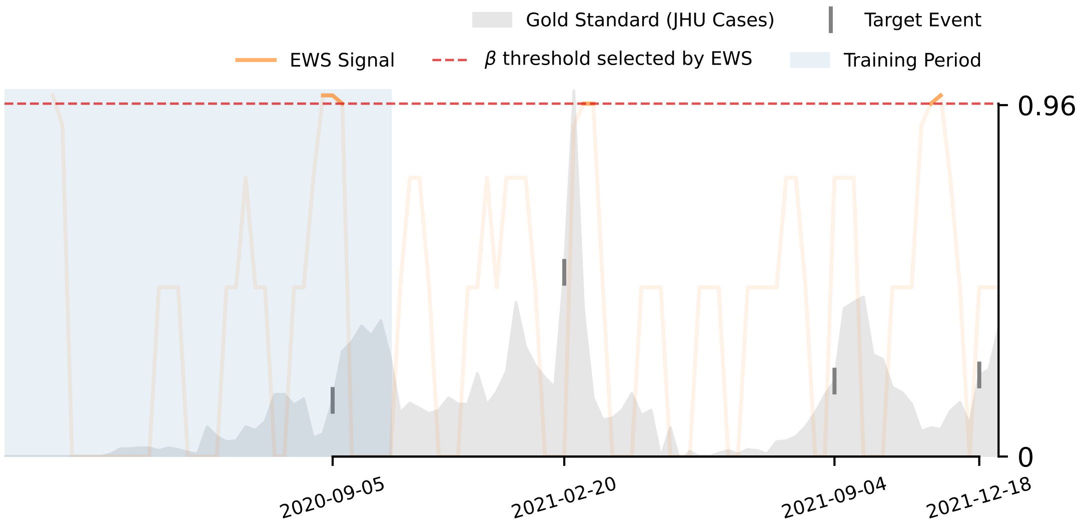

β	Training tp	Training fp	Training fn	Out of sample tp	Out of sample fp	Out of sample fn
1.00	0	0	1	0	0	3
0.99	0	0	1	1	0	2
0.96	1	1	0	1	2	1
0.91	1	2	0	1	3	1
0.76	1	3	0	3	8	0
0.46	1	4	0	3	28	0
0.00	1	11	0	3	42	0

Model 2

(GT) Cough
(GT) Otitis
Apple Mobility (transit)
(GT) Diarrhea
(GT) Vomiting

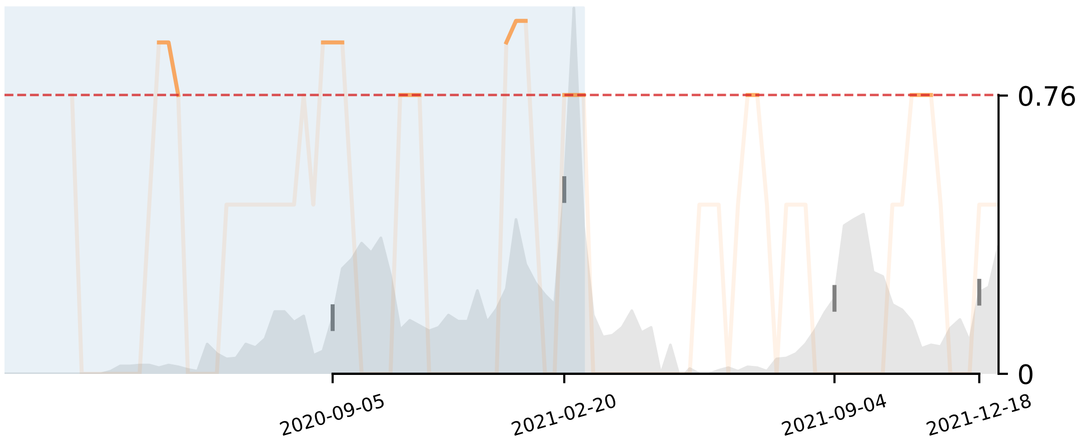

β	Training tp	Training fp	Training fn	Out of sample tp	Out of sample fp	Out of sample fn
0.99	0	0	2	0	0	2
0.96	0	0	2	0	0	2
0.91	1	1	1	0	0	2
0.76	2	5	0	1	3	1
0.46	2	12	0	2	12	0
0.00	2	21	0	2	29	0

Model 3

(GT) Cough
Apple Mobility (transit)
(GT) Chest pain
(GT) Otitis
(GT) Pneumonia
(GT) Sore throat

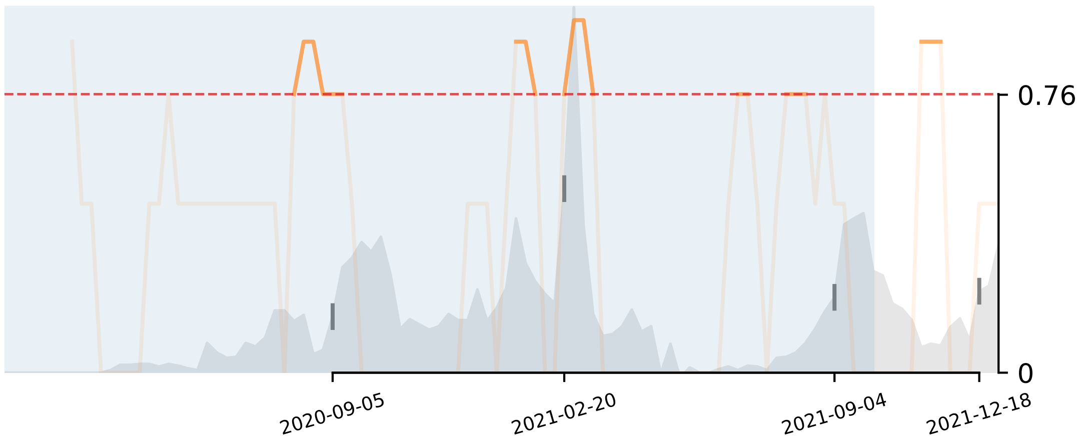

β	Training tp	Training fp	Training fn	Out of sample tp	Out of sample fp	Out of sample fn
1.00	0	0	3	0	0	1
0.99	0	0	3	0	0	1
0.96	0	0	3	0	0	1
0.91	0	2	3	1	0	0
0.76	2	4	1	1	0	0
0.46	3	10	0	1	2	0
0.00	3	34	0	1	6	0

Proxies selected by the Multiple Sources method

Model 1

(GT) covid
(GT) how long does covid last
(GT) covid-19
(GT) chest pain
(GT) covid-19 who
(GT) sars-cov-2

Model 2

(GT) covid symptoms
(GT) sars-cov-2
(GT) Chest pain
(GT) Ageusia
(GT) Otitis

Model 3

(GT) covid symptoms
(GT) Ageusia
(GT) Chest pain
(GT) loss of smell
(GT) sars-cov-2
(GT) Fatigue

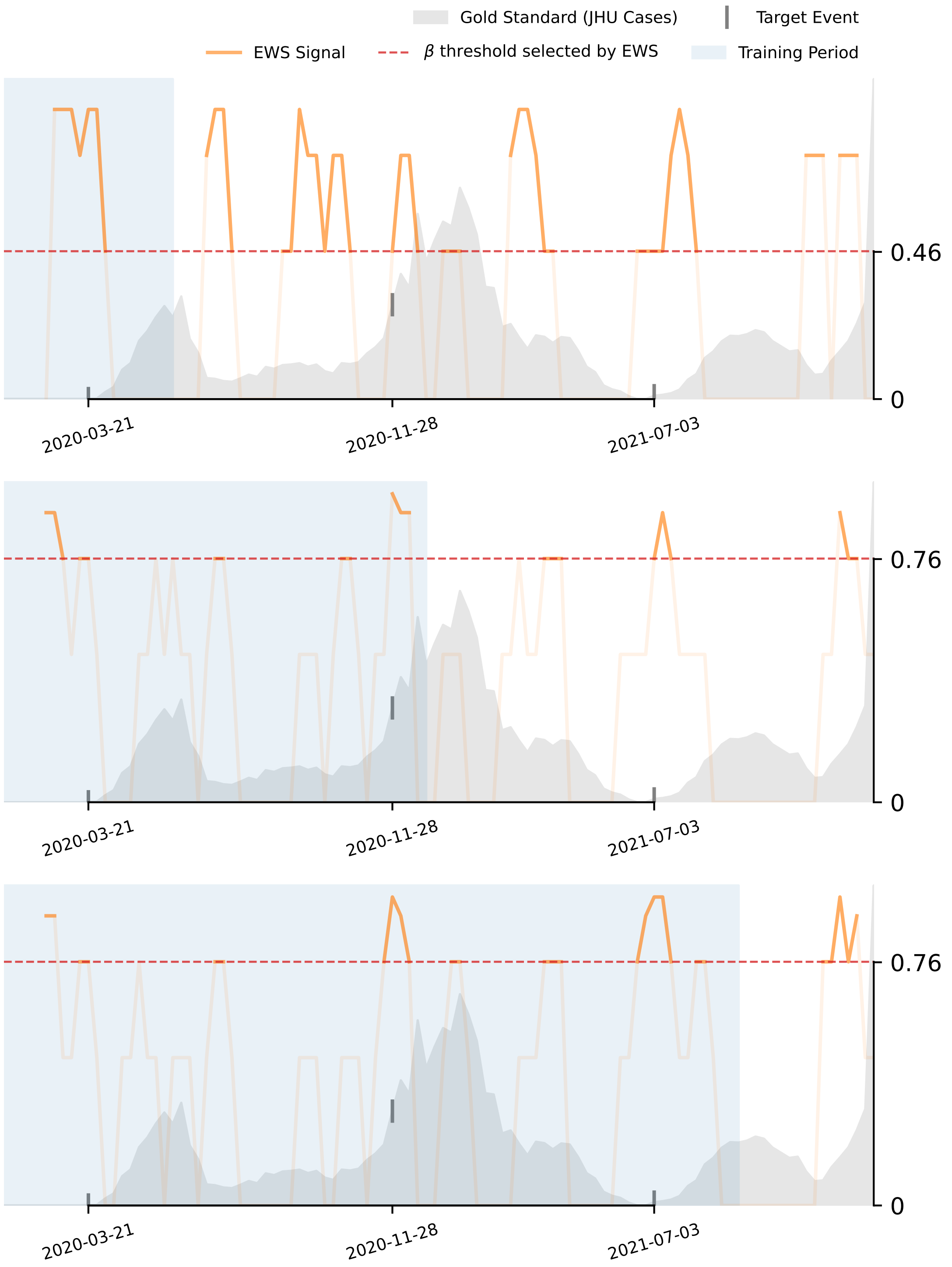

Performance as a function of β

Model 1

β	Training tp	Training fp	Training fn	Out of sample tp	Out of sample fp	Out of sample fn
1.00	0	0	1	0	0	2
0.99	0	0	1	0	0	2
0.96	0	0	1	0	0	2
0.91	0	0	1	0	6	2
0.76	1	1	0	1	22	0
0.46	1	1	0	2	34	0
0.00	1	2	0	2	69	0

Model 2

β	Training tp	Training fp	Training fn	Out of sample tp	Out of sample fp	Out of sample fn
0.99	0	0	2	0	0	1
0.96	0	0	2	0	0	1
0.91	1	0	1	0	2	0
0.76	2	3	0	1	9	0
0.46	2	9	0	1	24	0
0.00	2	21	0	1	46	0

Model 3

β	Training tp	Training fp	Training fn	Out of sample tp	Out of sample fp	Out of sample fn
1.00	0	0	3	0	0	0
0.99	0	0	3	0	0	0
0.96	0	0	3	0	1	0
0.91	2	1	1	0	2	0
0.76	3	3	0	0	5	0
0.46	3	15	0	0	7	0
0.00	3	38	0	0	16	0

Proxies selected by the Multiple Sources method

Model 1

(GT) covid
(GT) how long does covid last
(GT) covid-19
(GT) chest pain
(GT) covid symptoms
(GT) chest tightness

Model 2

(GT) covid
(GT) covid-19
(GT) chest pain
(GT) covid symptoms
(GT) fever
(GT) Ageusia

Model 3

(GT) chest pain
(GT) covid symptoms
(GT) Ageusia
(GT) Anosmia
(GT) Throat irritation
up2date

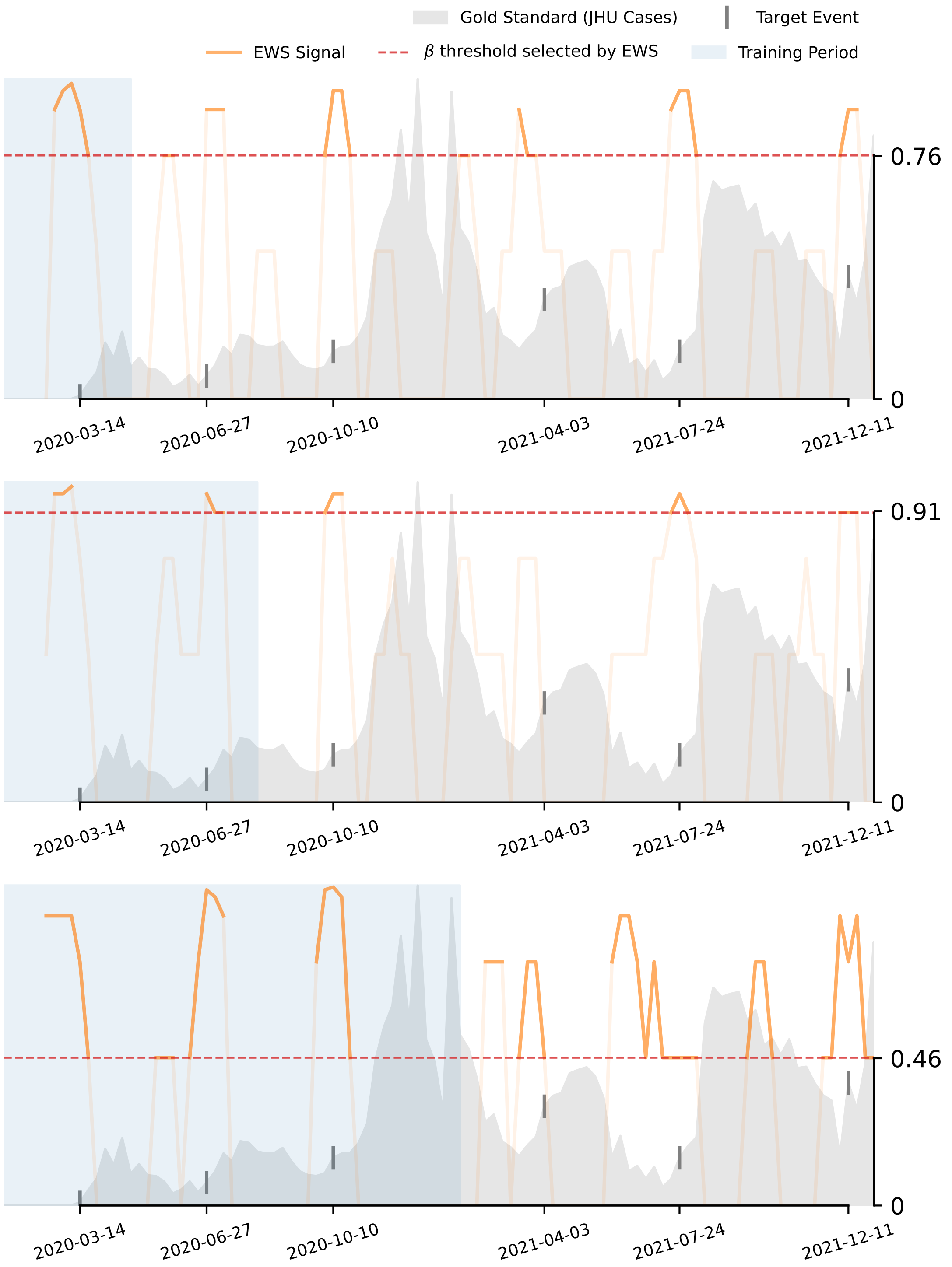

Performance as a function of β

Model 1

β	Training tp	Training fp	Training fn	Out of sample tp	Out of sample fp	Out of sample fn
1.00	0	0	1	0	0	5
0.99	0	0	1	0	0	5
0.96	1	0	0	2	2	3
0.91	1	0	0	5	5	0
0.76	1	0	0	5	9	0
0.46	1	1	0	5	25	0
0.00	1	2	0	5	53	0

Model 2

β	Training tp	Training fp	Training fn	Out of sample tp	Out of sample fp	Out of sample fn
1.00	0	0	2	0	0	4
0.99	0	0	2	0	0	4
0.96	1	0	1	2	1	2
0.91	2	0	0	3	3	1
0.76	2	2	0	4	7	0
0.46	2	3	0	4	21	0
0.00	2	5	0	4	45	0

Model 3

β	Training tp	Training fp	Training fn	Out of sample tp	Out of sample fp	Out of sample fn
1.00	0	0	3	0	0	3
0.99	1	0	2	0	0	3
0.96	2	0	1	0	0	3
0.91	2	2	1	2	2	1
0.76	3	3	0	3	6	0
0.46	3	3	0	3	12	0
0.00	3	7	0	3	28	0

Figures S140-S187.

Visualization of the multiple sources model as a function of time. The model's output (orange) is compared against the onsets (vertical gray tick marks) of the COVID-19 reported cases (gray-filled curve). As more information becomes available, the EWS model is re-trained to select the proxies that best fit the training data available (represented by the blue shading on each Figure). A list of the selected proxies for each model, along with a performance table are also presented.

Alabama

Proxies selected by the Multiple Sources method

Model 1

(GT) chest tightness
(GT) sars-cov-2
(GT) covid
(GT) covid-19
(GT) covid symptoms
(GT) quarantine

Model 2

(GT) Acute bronchitis
(GT) Nasal congestion
(GT) Wheeze
(GT) Ageusia
(GT) Burning Chest Pain

Model 3

(GT) Acute bronchitis
(GT) Nasal congestion
(GT) loss taste
(GT) Ageusia
(GT) Bronchitis
(GT) Burning Chest Pain

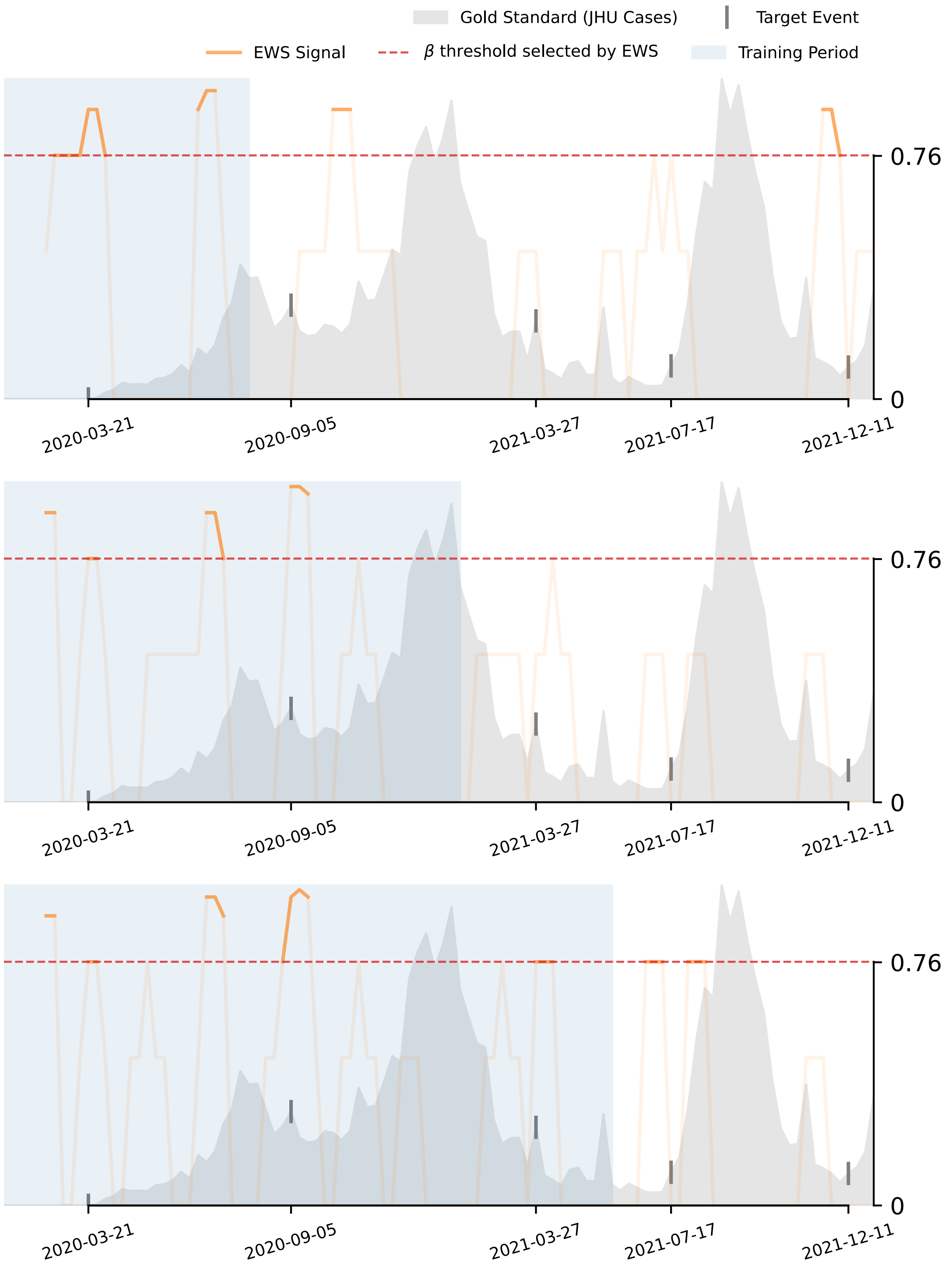

Performance as a function of β

Model 1

β	Training tp	Training fp	Training fn	Out of sample tp	Out of sample fp	Out of sample fn
1.00	0	0	1	0	0	4
0.99	0	0	1	0	0	4
0.96	0	0	1	0	0	4
0.91	0	2	1	1	3	3
0.76	1	4	0	2	3	2
0.46	1	5	0	3	19	0
0.00	1	7	0	4	48	0

Model 2

β	Training tp	Training fp	Training fn	Out of sample tp	Out of sample fp	Out of sample fn
0.99	0	0	2	0	0	3
0.96	1	1	1	0	0	3
0.91	1	2	1	0	0	3
0.76	2	5	0	0	1	2
0.46	2	8	0	3	8	0
0.00	2	20	0	3	28	0

Model 3

β	Training tp	Training fp	Training fn	Out of sample tp	Out of sample fp	Out of sample fn
1.00	0	0	3	0	0	2
0.99	0	0	3	0	0	2
0.96	0	1	3	0	0	2
0.91	1	4	2	0	0	2
0.76	2	6	1	1	3	1
0.46	3	11	0	2	3	0
0.00	3	27	0	2	17	0

Proxies selected by the Multiple Sources method

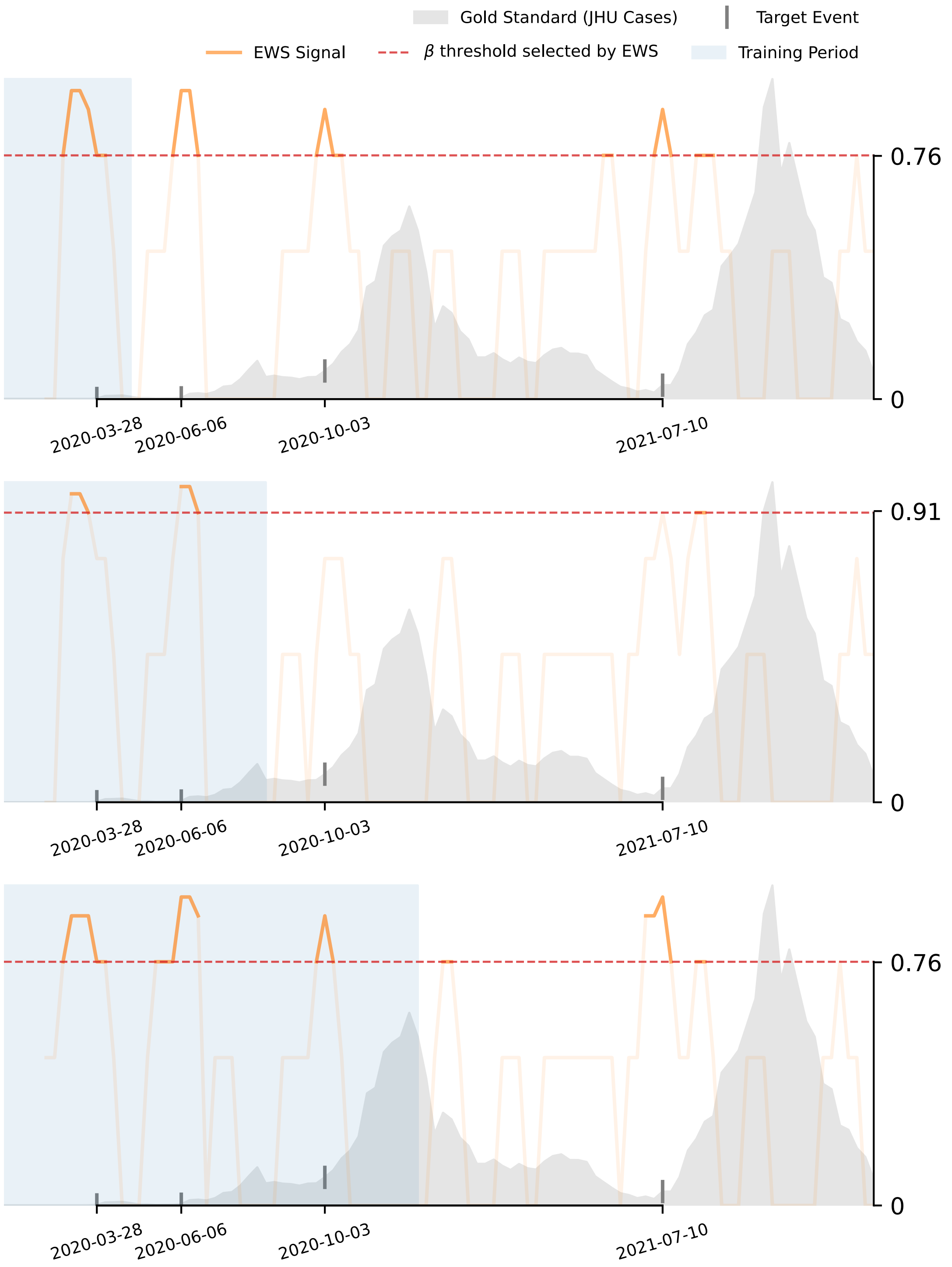

Performance as a function of β

Model 1

(JHU) cases
(GT) covid
(GT) covid-19
(GT) covid symptoms
(GT) quarantine
(GT) fever

β	Training tp	Training fp	Training fn	Out of sample tp	Out of sample fp	Out of sample fn
1.00	0	0	1	0	0	3
0.99	0	0	1	0	0	3
0.96	0	0	1	1	1	2
0.91	1	0	0	3	1	0
0.76	1	0	0	3	10	0
0.46	1	1	0	3	39	0
0.00	1	2	0	3	68	0

Model 2

(JHU) cases
(GT) covid
(GT) covid-19
(GT) covid symptoms
(GT) fever
(GT) Cardiac arrest

β	Training tp	Training fp	Training fn	Out of sample tp	Out of sample fp	Out of sample fn
1.00	0	0	2	0	0	2
0.99	0	0	2	0	0	2
0.96	1	1	1	0	0	2
0.91	2	1	0	1	2	1
0.76	2	2	0	2	9	0
0.46	2	3	0	2	33	0
0.00	2	4	0	2	58	0

Model 3

(JHU) cases
(GT) covid
(GT) covid symptoms
(GT) Asthma
(GT) fever
(GT) Cardiac arrest

β	Training tp	Training fp	Training fn	Out of sample tp	Out of sample fp	Out of sample fn
1.00	0	0	3	0	0	1
0.99	0	0	3	0	0	1
0.96	0	0	3	1	0	0
0.91	1	1	2	1	0	0
0.76	3	2	0	1	6	0
0.46	3	4	0	1	29	0
0.00	3	12	0	1	47	0

Proxies selected by the Multiple Sources method

Performance as a function of β

Model 1

(JHU) cases
(GT) covid
(GT) how long does covid last
(GT) covid-19
(GT) chest pain
(GT) covid symptoms

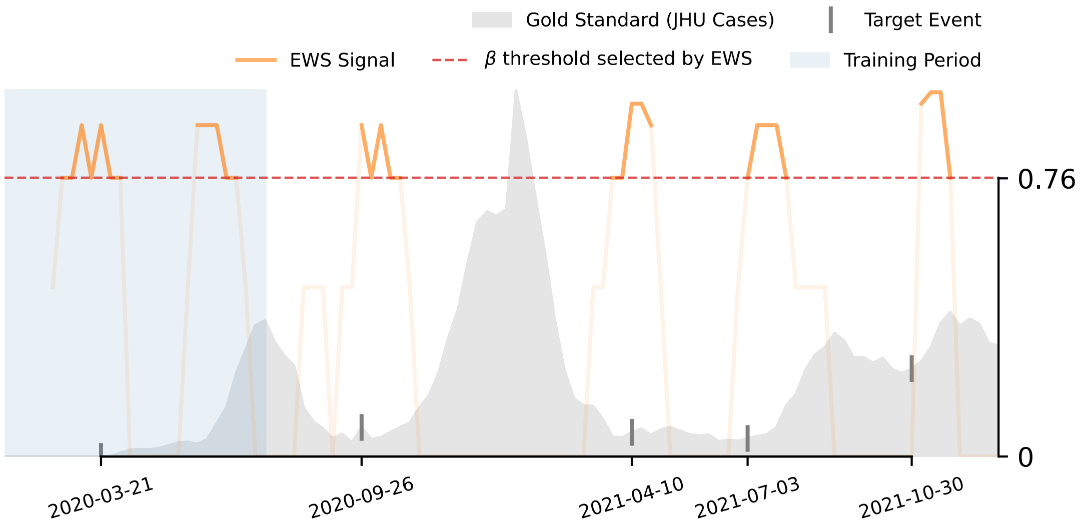

β	Training tp	Training fp	Training fn	Out of sample tp	Out of sample fp	Out of sample fn
1.00	0	0	1	0	2	3
0.99	0	0	1	0	2	3
0.96	0	0	1	1	4	2
0.91	0	0	1	2	9	0
0.76	1	3	0	3	14	0
0.46	1	7	0	3	20	0
0.00	1	10	0	4	48	0

Model 2

(JHU) cases
(GT) covid-19
(GT) chest pain
(GT) covid symptoms
(GT) fever
(GT) Acute bronchitis

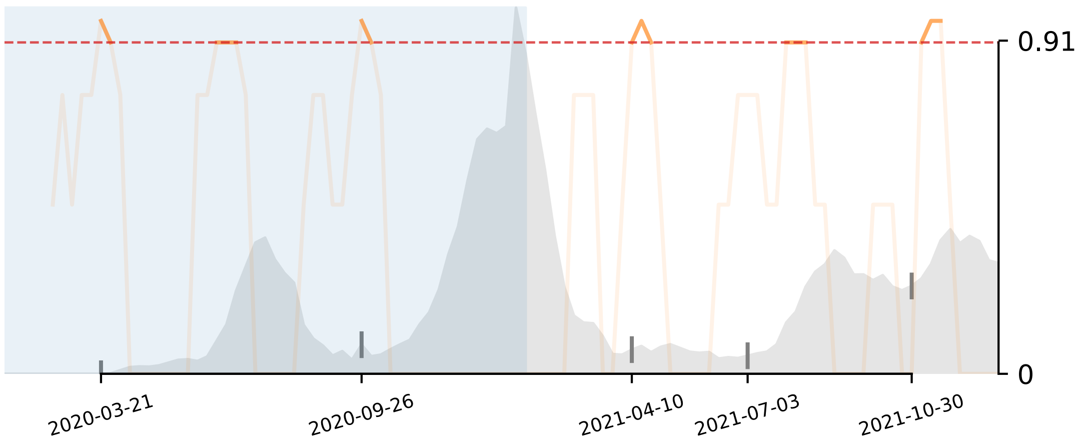

β	Training tp	Training fp	Training fn	Out of sample tp	Out of sample fp	Out of sample fn
1.00	0	0	2	0	0	3
0.99	0	0	2	0	0	3
0.96	0	0	2	0	3	1
0.91	2	0	0	1	8	1
0.76	2	5	0	2	9	0
0.46	2	11	0	3	15	0
0.00	2	13	0	3	28	0

Model 3

(GT) covid-19
(GT) chest pain
(GT) covid symptoms
(GT) fever
(GT) Acute bronchitis
(GT) Chills

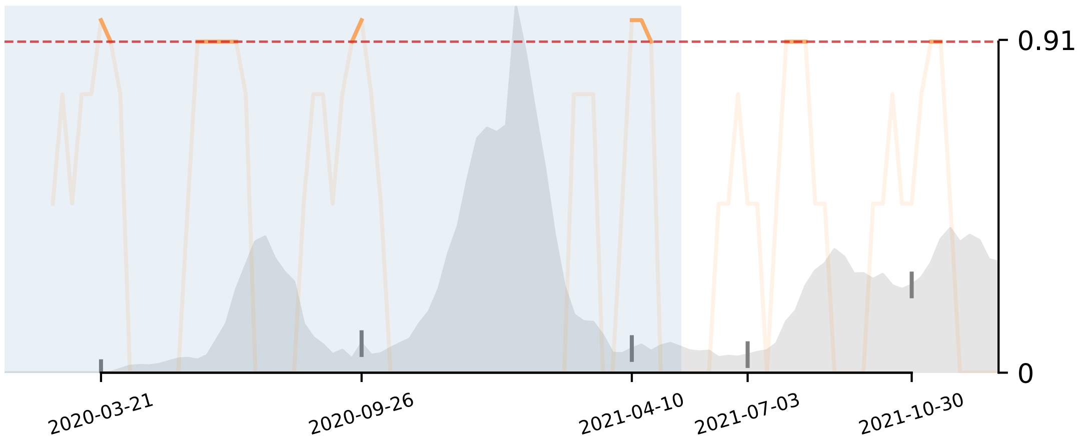

β	Training tp	Training fp	Training fn	Out of sample tp	Out of sample fp	Out of sample fn
1.00	0	0	3	0	0	2
0.99	0	0	3	0	0	2
0.96	0	0	3	0	0	2
0.91	3	1	0	0	5	1
0.76	3	8	0	2	6	0
0.46	3	14	0	2	11	0
0.00	3	18	0	2	19	0

Arkansas

Proxies selected by the Multiple Sources method

Model 1

(GT) chest tightness
(GT) Viral pneumonia
(GT) covid
(GT) covid-19
(GT) chest pain
(GT) covid symptoms

Model 2

(GT) chest pain
(GT) covid symptoms
(GT) fever
(GT) Abdominal pain
(GT) Acute bronchitis
(GT) Asthma

Model 3

(GT) Burning Chest Pain
(GT) covid
(GT) covid-19
(GT) covid symptoms
(GT) quarantine
(GT) Abdominal pain

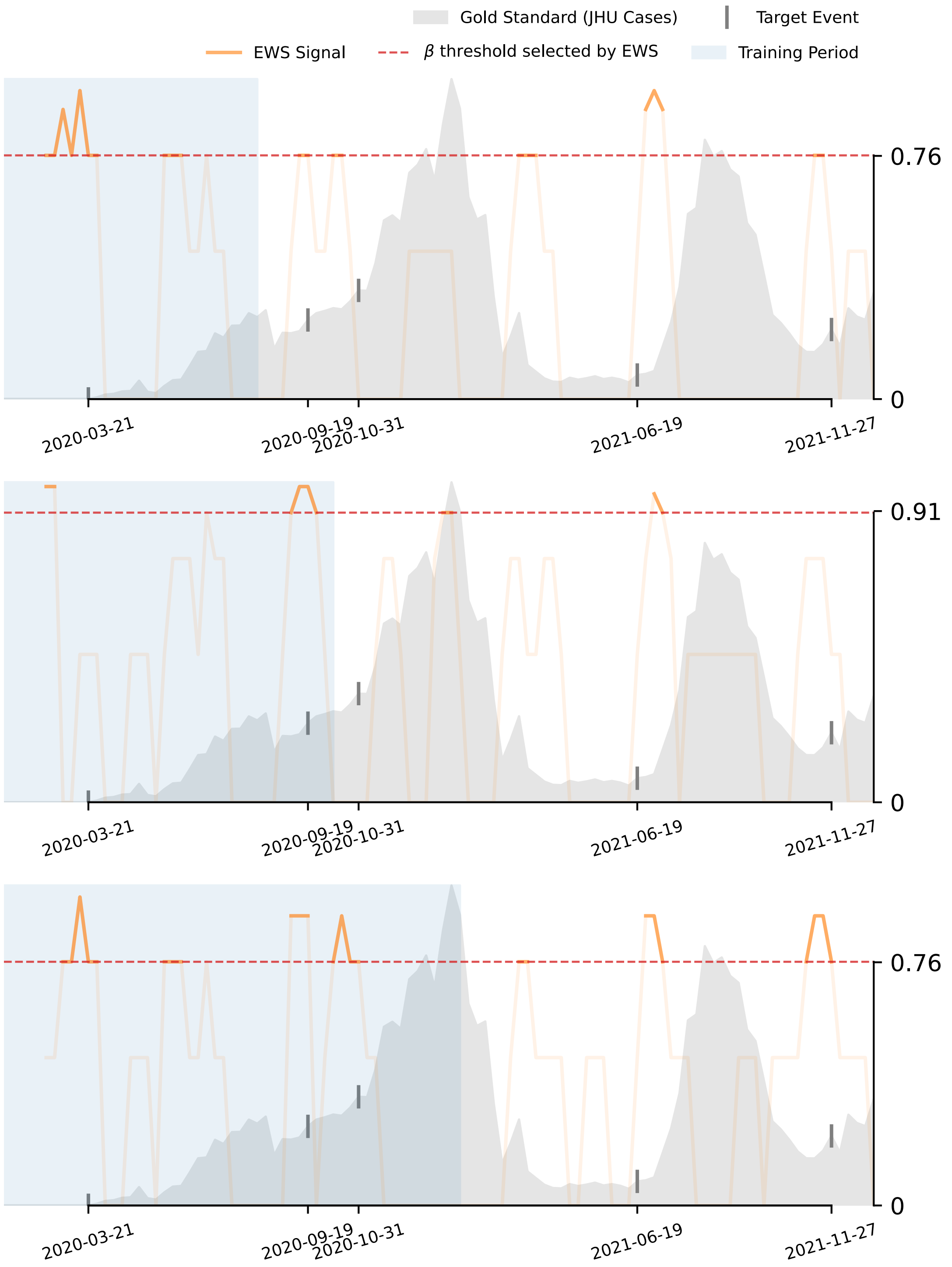

Performance as a function of β

Model 1

β	Training tp	Training fp	Training fn	Out of sample tp	Out of sample fp	Out of sample fn
1.00	0	0	1	0	0	4
0.99	0	0	1	0	0	4
0.96	0	0	1	0	1	3
0.91	1	0	0	0	3	3
0.76	1	0	0	3	6	0
0.46	1	6	0	4	19	0
0.00	1	10	0	4	47	0

Model 2

β	Training tp	Training fp	Training fn	Out of sample tp	Out of sample fp	Out of sample fn
1.00	0	0	2	0	0	3
0.99	0	0	2	0	0	3
0.96	2	1	0	0	1	2
0.91	2	1	0	0	4	2
0.76	2	3	0	1	13	1
0.46	2	8	0	2	30	0
0.00	2	15	0	3	47	0

Model 3

β	Training tp	Training fp	Training fn	Out of sample tp	Out of sample fp	Out of sample fn
1.00	0	0	3	0	0	2
0.99	0	0	3	0	0	2
0.96	0	0	3	0	0	2
0.91	1	0	2	1	2	0
0.76	3	0	0	1	5	0
0.46	3	5	0	2	21	0
0.00	3	15	0	2	35	0

California

Proxies selected by the Multiple Sources method

Model 1

(JHU) cases
(GT) Anxiety
(GT) covid
(GT) covid-19
(GT) loss taste
(GT) loss of smell

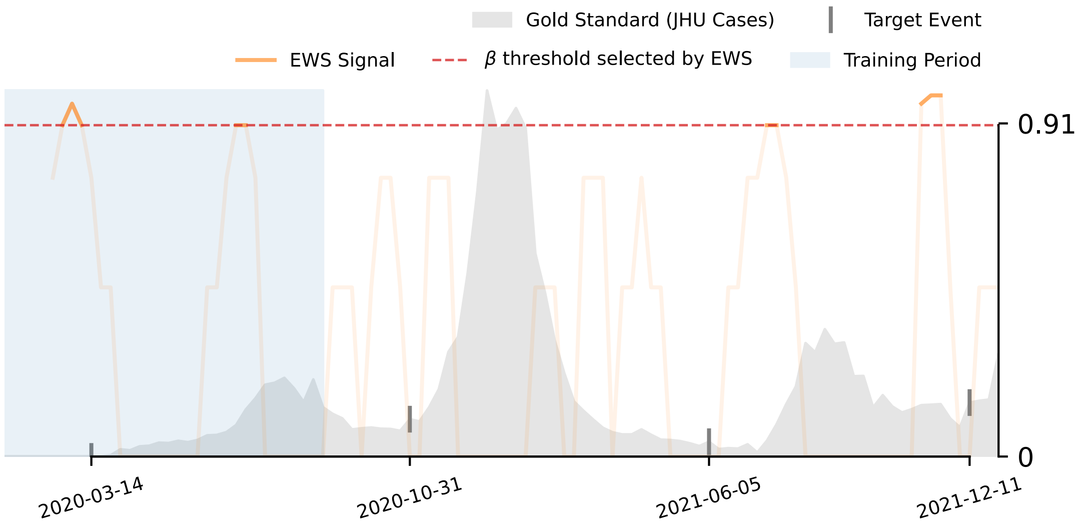

Performance as a function of β

	Training			Out of sample		
β	tp	fp	fn	tp	fp	fn
1.00	0	0	1	0	0	3
0.99	0	0	1	1	0	2
0.96	0	0	1	1	0	2
0.91	1	0	0	1	2	2
0.76	1	2	0	2	12	1
0.46	1	4	0	3	25	0
0.00	1	8	0	3	49	0

Model 2

(JHU) cases
(GT) loss of smell
(GT) chest tightness
(GT) quarantine
(GT) sars-cov-2
(GT) Ageusia

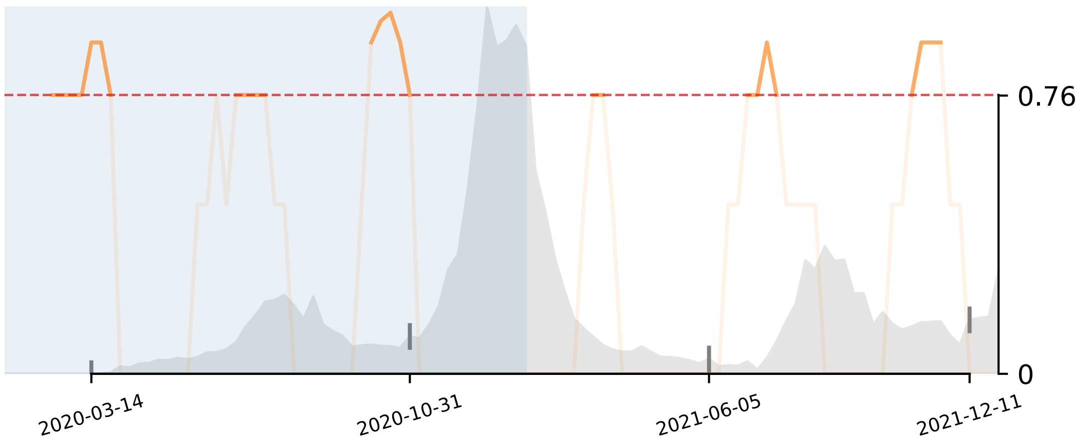

β	tp	fp	fn	tp	fp	fn
1.00	0	0	2	0	0	2
0.99	0	0	2	0	0	2
0.96	1	0	1	0	0	2
0.91	1	0	1	1	1	1
0.76	2	1	0	1	6	1
0.46	2	7	0	1	16	0
0.00	2	13	0	2	35	0

Model 3

(JHU) cases
(GT) Ageusia
(GT) Anosmia
(GT) Viral pneumonia
(GT) loss of smell
(GT) quarantine

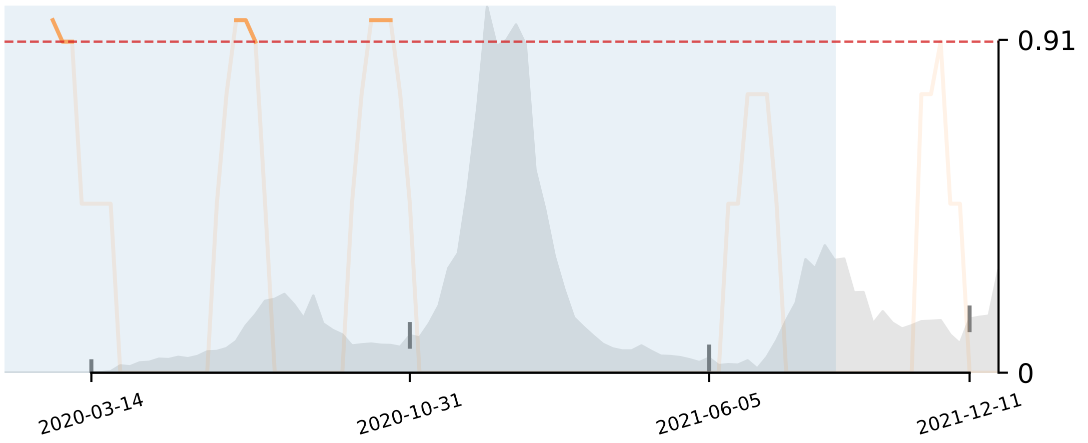

β	tp	fp	fn	tp	fp	fn
1.00	0	0	3	0	0	1
0.99	0	0	3	0	0	1
0.96	0	0	3	0	0	1
0.91	2	2	1	1	0	0
0.76	2	3	1	1	0	0
0.46	2	8	1	1	0	0
0.00	2	16	1	1	10	0

Proxies selected by the Multiple Sources method

Model 1

(GT) covid
(GT) covid-19
(GT) chest pain
(GT) covid symptoms
(GT) quarantine
(GT) nose bleed

Model 2

(GT) covid
(GT) covid-19
(GT) chest pain
(GT) covid symptoms
(GT) quarantine
(GT) Acute bronchitis

Model 3

(GT) covid symptoms
(GT) Acute bronchitis
(GT) Ageusia
(GT) Anosmia
(GT) Asthma
(GT) Bronchitis

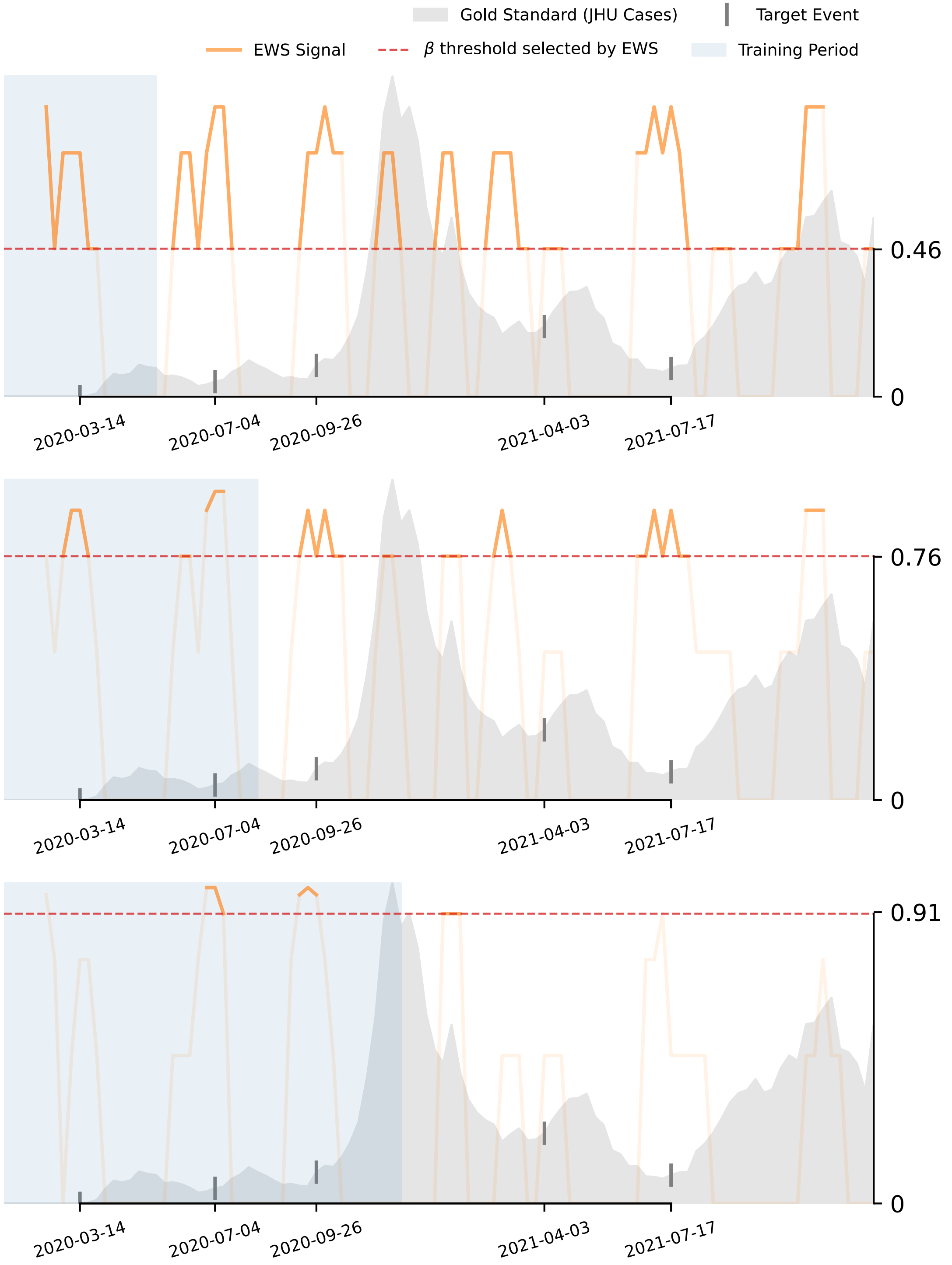

Performance as a function of β

Model 1

	Training			Out of sample		
β	tp	fp	fn	tp	fp	fn
1.00	0	0	1	0	0	4
0.99	0	0	1	0	0	4
0.96	0	0	1	0	0	4
0.91	0	0	1	2	5	1
0.76	1	0	0	4	12	0
0.46	1	0	0	4	29	0
0.00	1	2	0	4	57	0

Model 2

β	tp	fp	fn	tp	fp	fn
1.00	0	0	2	0	0	3
0.99	0	0	2	0	0	3
0.96	0	0	2	0	0	3
0.91	1	1	1	3	4	0
0.76	2	1	0	3	13	0
0.46	2	2	0	3	28	0
0.00	2	5	0	3	52	0

Model 3

β	tp	fp	fn	tp	fp	fn
1.00	0	0	3	0	0	2
0.99	0	0	3	0	0	2
0.96	2	0	1	0	0	2
0.91	3	0	0	1	3	1
0.76	3	1	0	1	4	1
0.46	3	3	0	2	14	0
0.00	3	6	0	2	42	0

Connecticut

Proxies selected by the Multiple Sources method

Model 1

(GT) covid
(GT) how long does covid last
(GT) covid-19
(GT) chest pain
(GT) covid symptoms
(GT) covid-19 who

Model 2

(GT) covid
(GT) covid-19
(GT) chest pain
(GT) Acute bronchitis
(GT) Ageusia
(GT) Bronchitis

Model 3

(GT) covid
(GT) Asthma
(GT) covid-19
(GT) chest pain
(GT) Chest pain
(GT) Shortness of breath

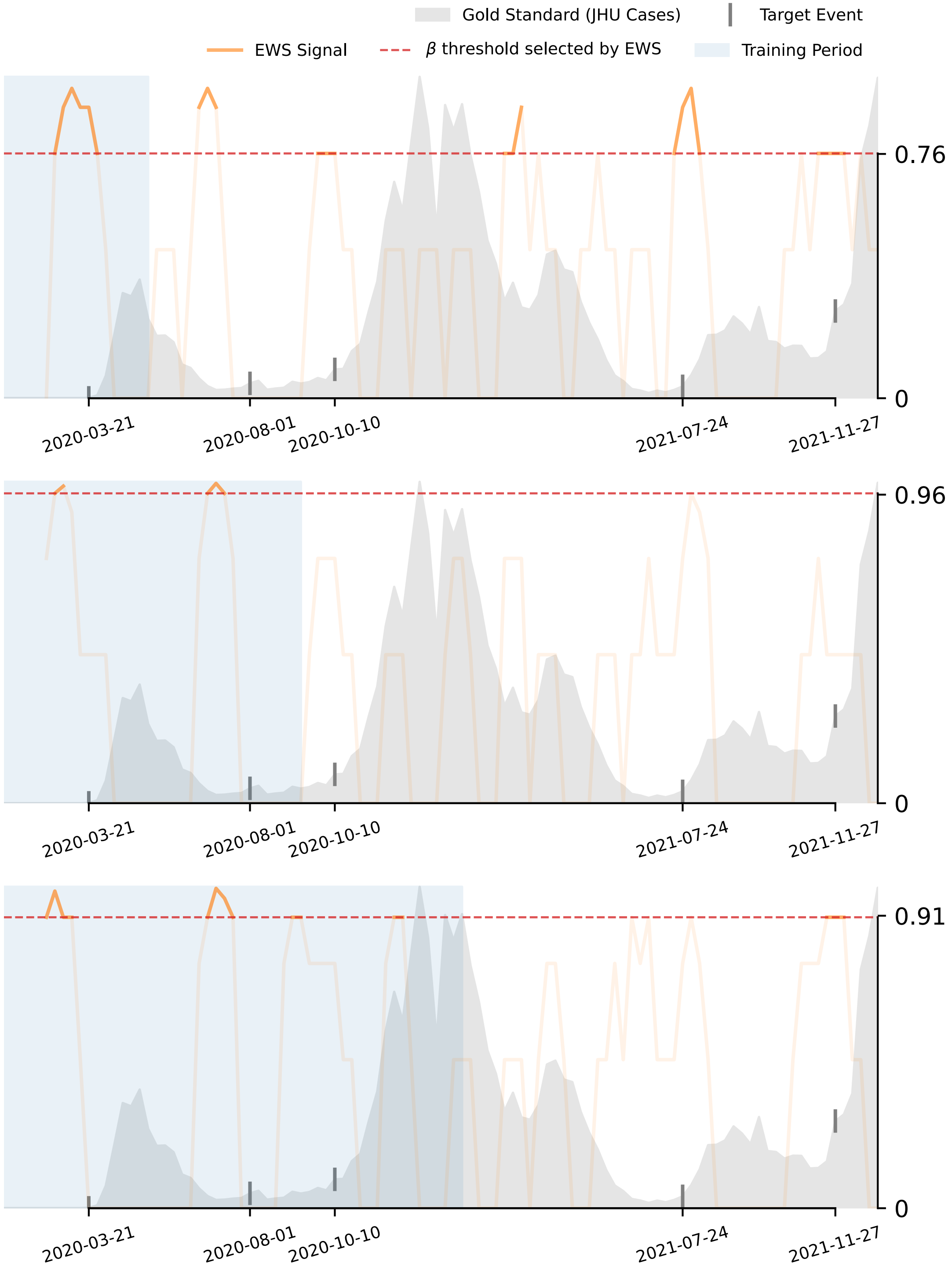

Performance as a function of β

Model 1

β	Training tp	Training fp	Training fn	Out of sample tp	Out of sample fp	Out of sample fn
1.00	0	0	1	0	0	4
0.99	0	0	1	0	0	4
0.96	0	0	1	1	1	2
0.91	1	0	0	2	2	2
0.76	1	0	0	4	9	0
0.46	1	1	0	4	35	0
0.00	1	2	0	4	58	0

Model 2

β	Training tp	Training fp	Training fn	Out of sample tp	Out of sample fp	Out of sample fn
1.00	0	0	2	0	0	3
0.99	1	0	1	0	0	3
0.96	2	0	0	0	1	2
0.91	2	1	0	0	2	2
0.76	2	1	0	3	8	0
0.46	2	3	0	3	24	0
0.00	2	5	0	3	50	0

Model 3

β	Training tp	Training fp	Training fn	Out of sample tp	Out of sample fp	Out of sample fn
1.00	0	0	3	0	0	2
0.99	1	0	2	0	0	2
0.96	2	0	1	0	0	2
0.91	2	0	1	2	2	0
0.76	3	5	0	2	6	0
0.46	3	8	0	2	18	0
0.00	3	13	0	2	35	0

Delaware

Proxies selected by the Multiple Sources method

Model 1

(GT) covid
(GT) covid-19
(GT) covid symptoms
(GT) quarantine
(GT) fever
(GT) Acute bronchitis

Model 2

(GT) quarantine
(GT) Throat irritation
(GT) Bronchitis
(GT) Wheeze
(GT) covid symptoms

Model 3

(GT) quarantine
(JHU) cases
(GT) Throat irritation
(GT) covid
(GT) Acute bronchitis
(GT) Bronchitis

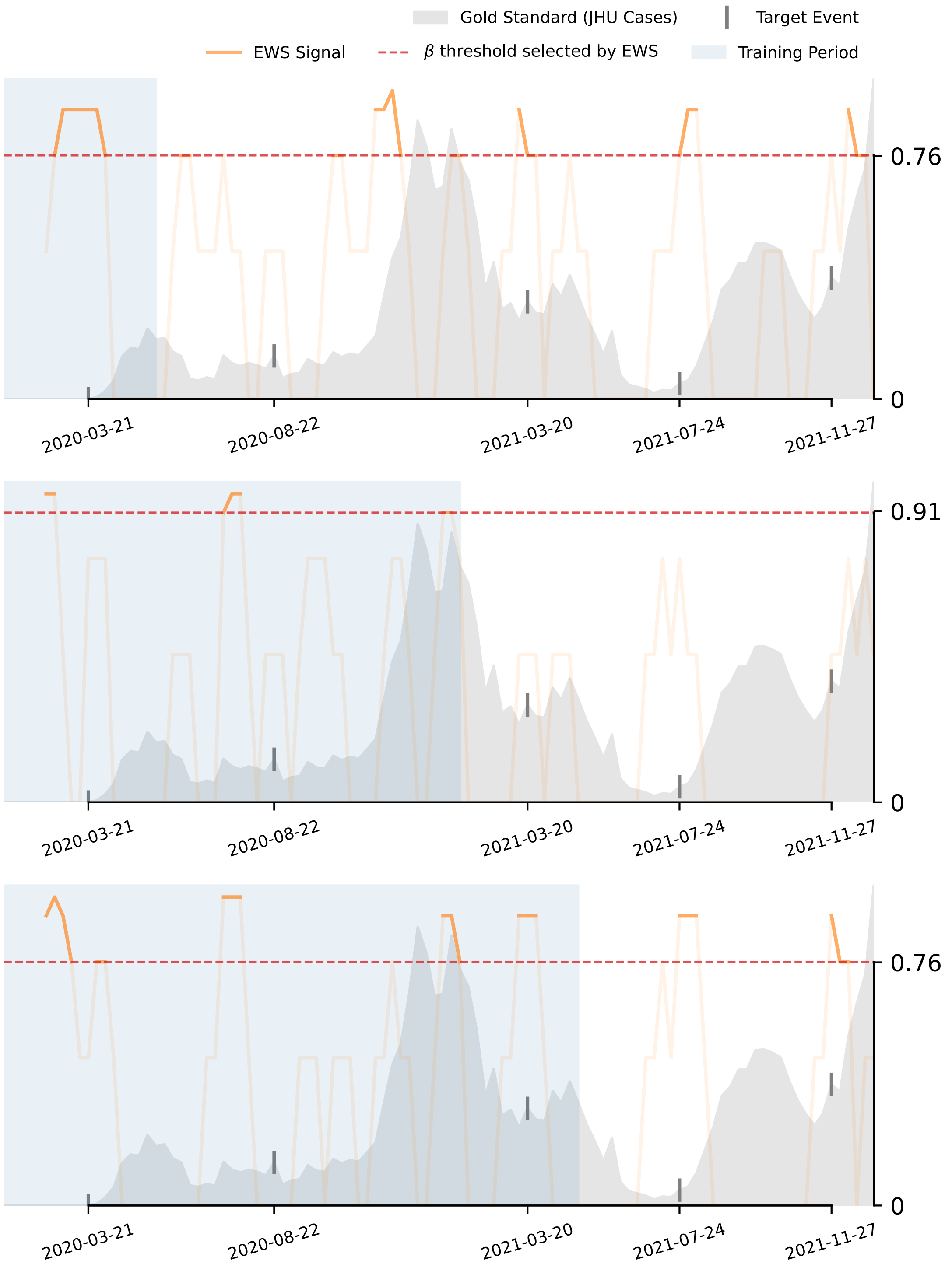

Performance as a function of β

Model 1

β	Training tp	Training fp	Training fn	Out of sample tp	Out of sample fp	Out of sample fn
1.00	0	0	1	0	0	4
0.99	0	0	1	0	0	4
0.96	0	0	1	0	1	4
0.91	0	0	1	1	6	1
0.76	1	1	0	4	17	0
0.46	1	2	0	4	37	0
0.00	1	3	0	4	57	0

Model 2

β	Training tp	Training fp	Training fn	Out of sample tp	Out of sample fp	Out of sample fn
0.99	0	0	2	0	0	3
0.96	0	0	2	0	0	3
0.91	2	2	0	0	0	3
0.76	2	5	0	1	2	1
0.46	2	13	0	3	11	0
0.00	2	23	0	3	28	0

Model 3

β	Training tp	Training fp	Training fn	Out of sample tp	Out of sample fp	Out of sample fn
1.00	0	0	3	0	0	2
0.99	0	0	3	0	0	2
0.96	0	0	3	0	0	2
0.91	2	2	1	2	2	0
0.76	3	6	0	2	4	0
0.46	3	10	0	2	7	0
0.00	3	25	0	2	21	0

Proxies selected by the Multiple Sources method

Model 1

(GT) covid
(GT) how long does covid last
(GT) covid-19
(GT) loss taste
(GT) chest pain
(GT) covid symptoms

Model 2

(GT) how long does covid last
(GT) loss taste
(GT) chest pain
(GT) covid symptoms
(GT) chest tightness
(GT) Ageusia

Model 3

(GT) how long does covid last
(GT) loss taste
(GT) Ageusia
(GT) Shortness of breath
(GT) chest pain
(GT) covid symptoms

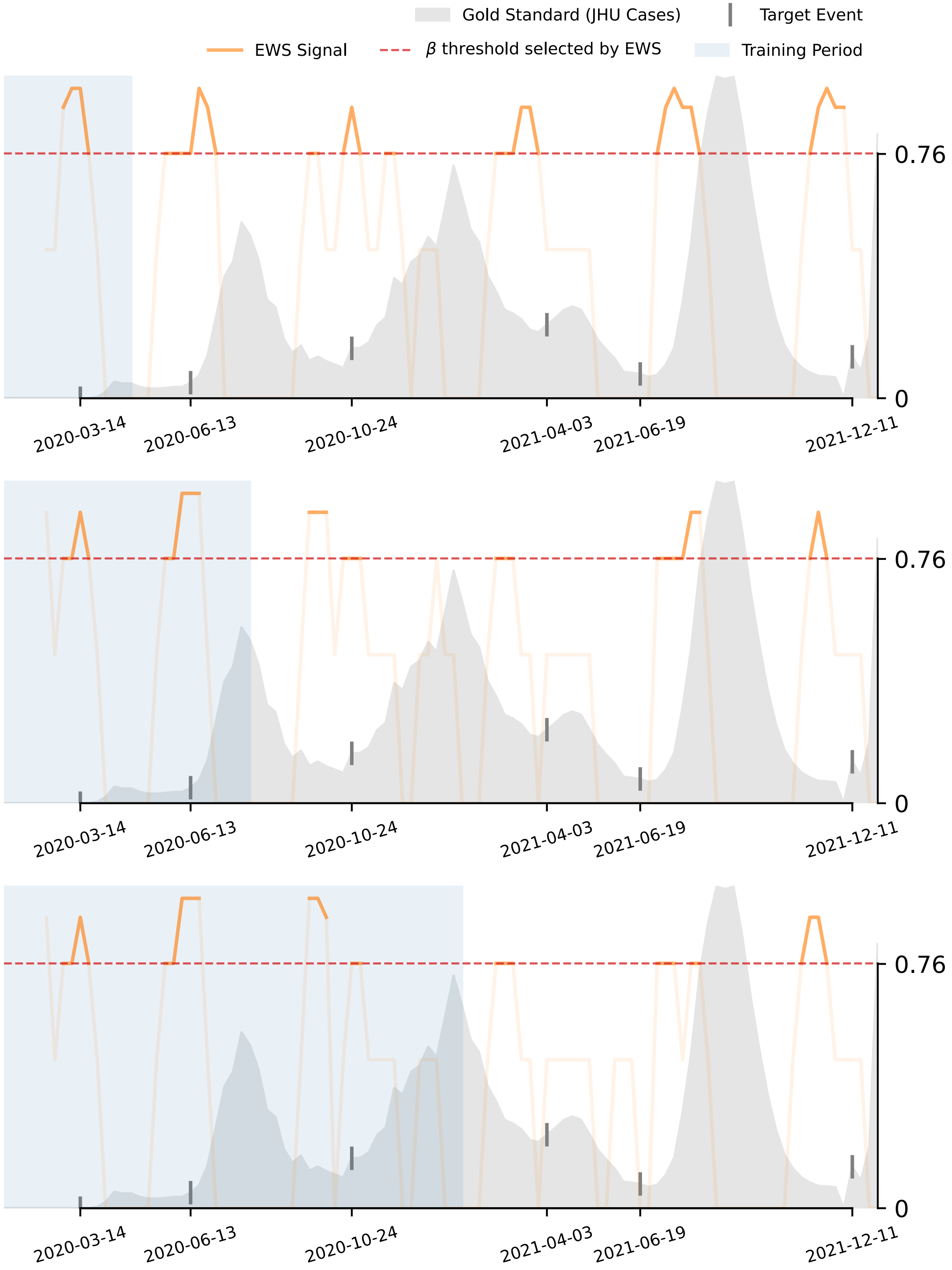

Performance as a function of β

Model 1

β	Training tp	Training fp	Training fn	Out of sample tp	Out of sample fp	Out of sample fn
1.00	0	0	1	0	0	5
0.99	0	0	1	0	0	5
0.96	0	0	1	1	2	3
0.91	1	0	0	3	6	1
0.76	1	0	0	4	12	0
0.46	1	1	0	5	25	0
0.00	1	2	0	5	53	0

Model 2

β	Training tp	Training fp	Training fn	Out of sample tp	Out of sample fp	Out of sample fn
1.00	0	0	2	0	0	4
0.99	0	0	2	0	0	4
0.96	0	0	2	0	0	4
0.91	1	1	1	2	2	2
0.76	2	1	0	3	8	0
0.46	2	2	0	4	23	0
0.00	2	4	0	4	46	0

Model 3

β	Training tp	Training fp	Training fn	Out of sample tp	Out of sample fp	Out of sample fn
1.00	0	0	3	0	0	3
0.99	0	0	3	0	0	3
0.96	0	0	3	0	0	3
0.91	2	2	1	1	0	2
0.76	3	2	0	2	5	0
0.46	3	4	0	3	14	0
0.00	3	14	0	3	28	0

Proxies selected by the Multiple Sources method

Model 1

(GT) covid
(GT) covid-19
(GT) loss taste
(GT) chest pain
(GT) covid symptoms
(GT) chest tightness

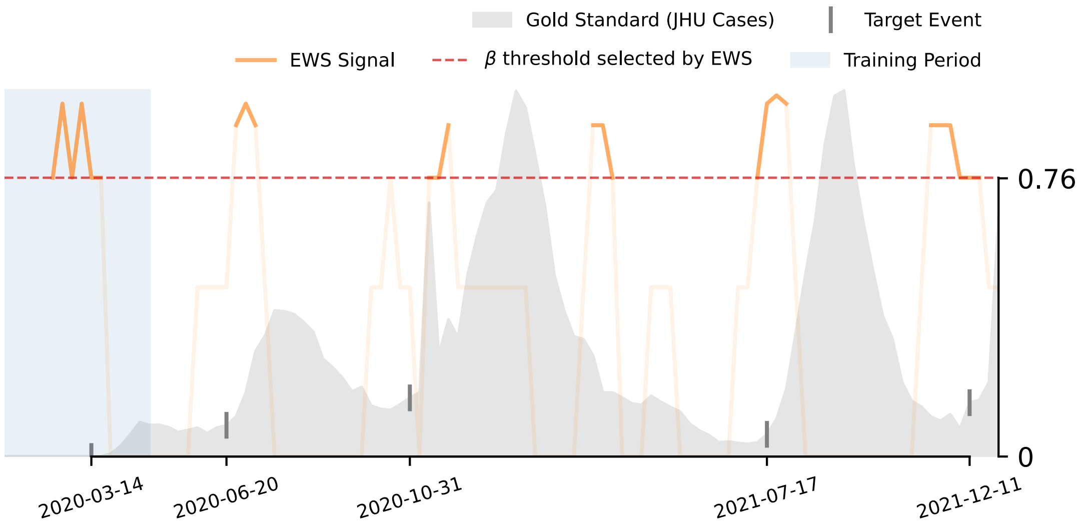

Performance as a function of β

β	Training tp	Training fp	Training fn	Out of sample tp	Out of sample fp	Out of sample fn
1.00	0	0	1	0	0	4
0.99	0	0	1	0	1	3
0.96	0	0	1	1	3	2
0.91	1	0	0	2	8	1
0.76	1	0	0	3	12	0
0.46	1	1	0	4	28	0
0.00	1	1	0	4	60	0

Model 2

(GT) chest pain
(GT) covid symptoms
(GT) fever
(GT) Ageusia
(GT) Anosmia
(GT) Asthma

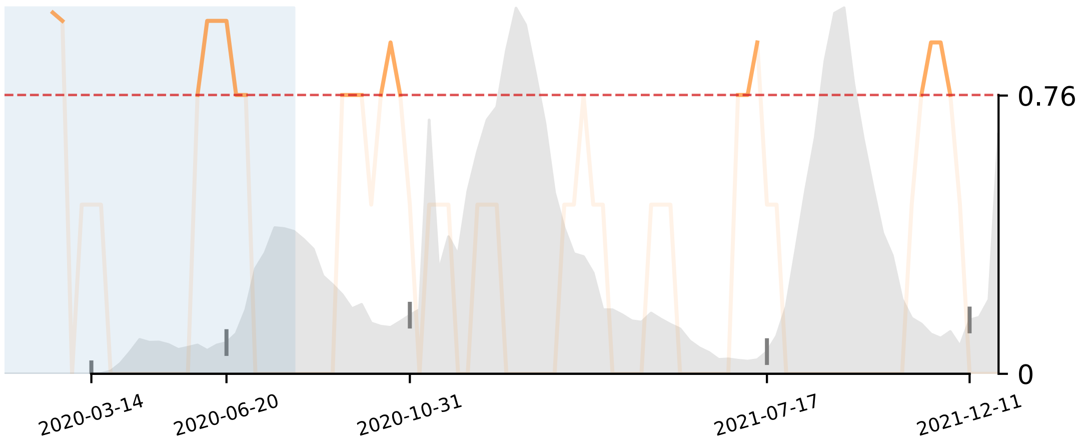

β	Training tp	Training fp	Training fn	Out of sample tp	Out of sample fp	Out of sample fn
1.00	0	0	2	0	0	3
0.99	0	0	2	0	0	3
0.96	1	0	1	0	0	3
0.91	2	0	0	3	0	0
0.76	2	0	0	3	2	0
0.46	2	2	0	3	16	0
0.00	2	3	0	3	52	0

Model 3

(GT) fever
(GT) Ageusia
(GT) Chills
(GT) Shortness of breath
(GT) chest pain
(GT) Burning Chest Pain

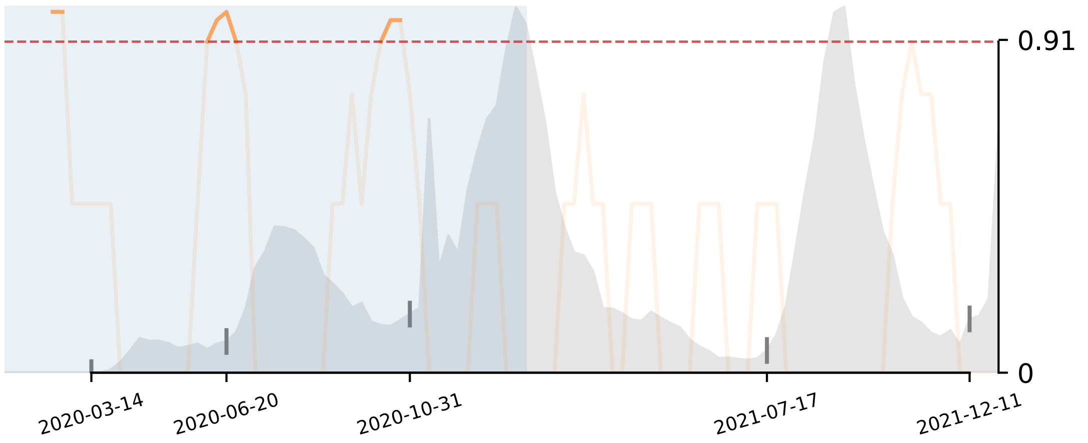

β	Training tp	Training fp	Training fn	Out of sample tp	Out of sample fp	Out of sample fn
1.00	0	0	3	0	0	2
0.99	0	0	3	0	0	2
0.96	2	0	1	0	0	2
0.91	3	0	0	1	0	1
0.76	3	1	0	1	2	1
0.46	3	3	0	2	12	0
0.00	3	12	0	2	35	0

Proxies selected by the Multiple Sources method

Model 1

(JHU) cases
(GT) covid
(GT) covid-19
(GT) covid symptoms
(GT) fever
(GT) Otitis

Model 2

(JHU) cases
(GT) covid symptoms
(GT) Otitis

Model 3

(JHU) cases
(GT) Vomiting
(GT) covid-19
(GT) covid symptoms
(GT) quarantine
(GT) fever

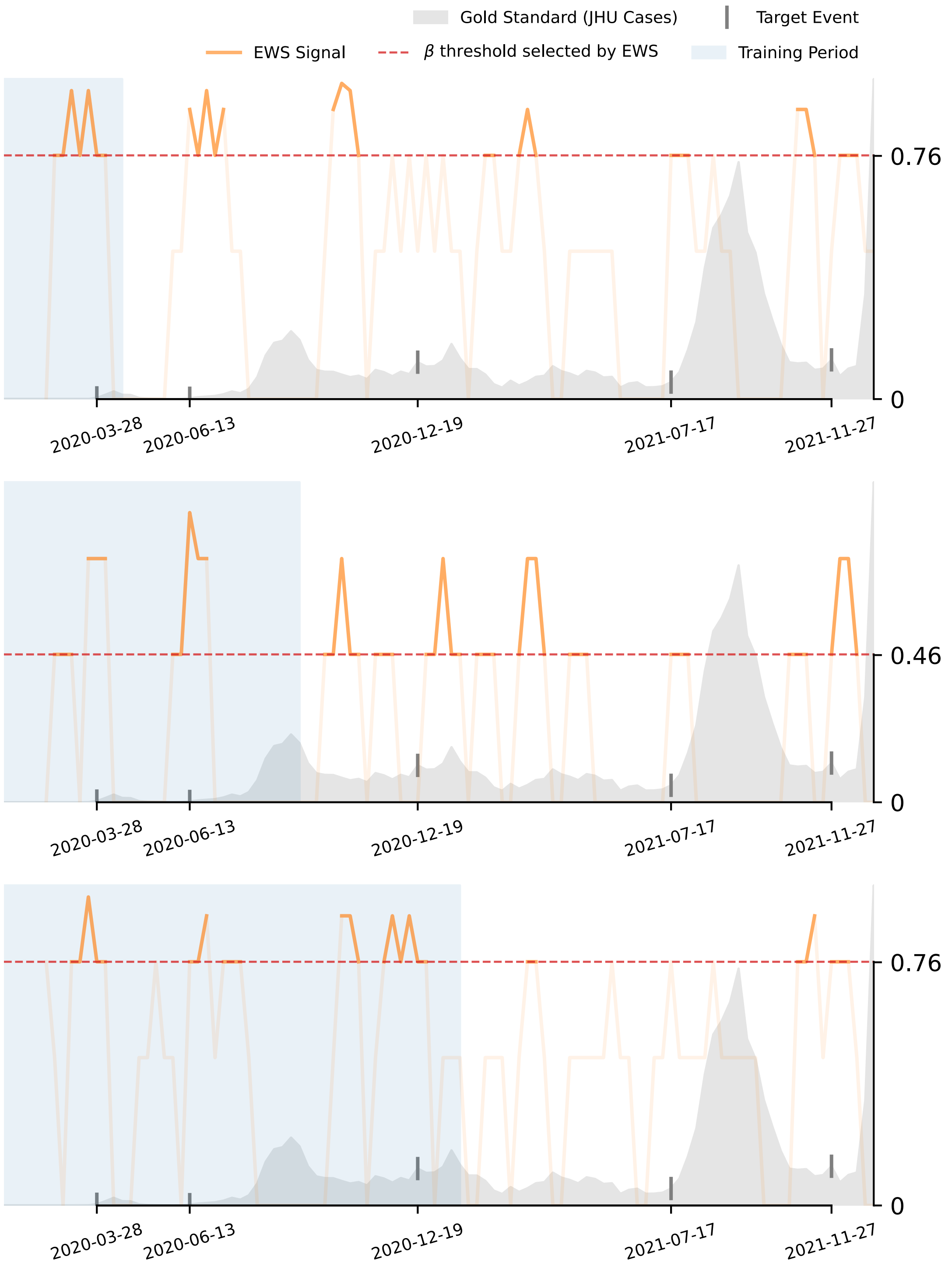

Performance as a function of β

Model 1

β	Training tp	Training fp	Training fn	Out of sample tp	Out of sample fp	Out of sample fn
1.00	0	0	1	0	0	4
0.99	0	0	1	0	1	4
0.96	0	0	1	0	3	3
0.91	1	0	0	2	6	2
0.76	1	0	0	4	21	0
0.46	1	2	0	4	43	0
0.00	1	2	0	4	61	0

Model 2

β	Training tp	Training fp	Training fn	Out of sample tp	Out of sample fp	Out of sample fn
0.91	0	0	2	0	0	3
0.76	1	0	1	0	6	2
0.46	2	3	0	3	25	0
0.00	2	4	0	3	47	0

Model 3

β	Training tp	Training fp	Training fn	Out of sample tp	Out of sample fp	Out of sample fn
1.00	0	0	3	0	0	2
0.99	0	0	3	0	0	2
0.96	0	0	3	0	0	2
0.91	1	0	2	1	0	1
0.76	2	3	1	2	6	0
0.46	3	11	0	2	26	0
0.00	3	21	0	2	35	0

Idaho

Proxies selected by the Multiple Sources method

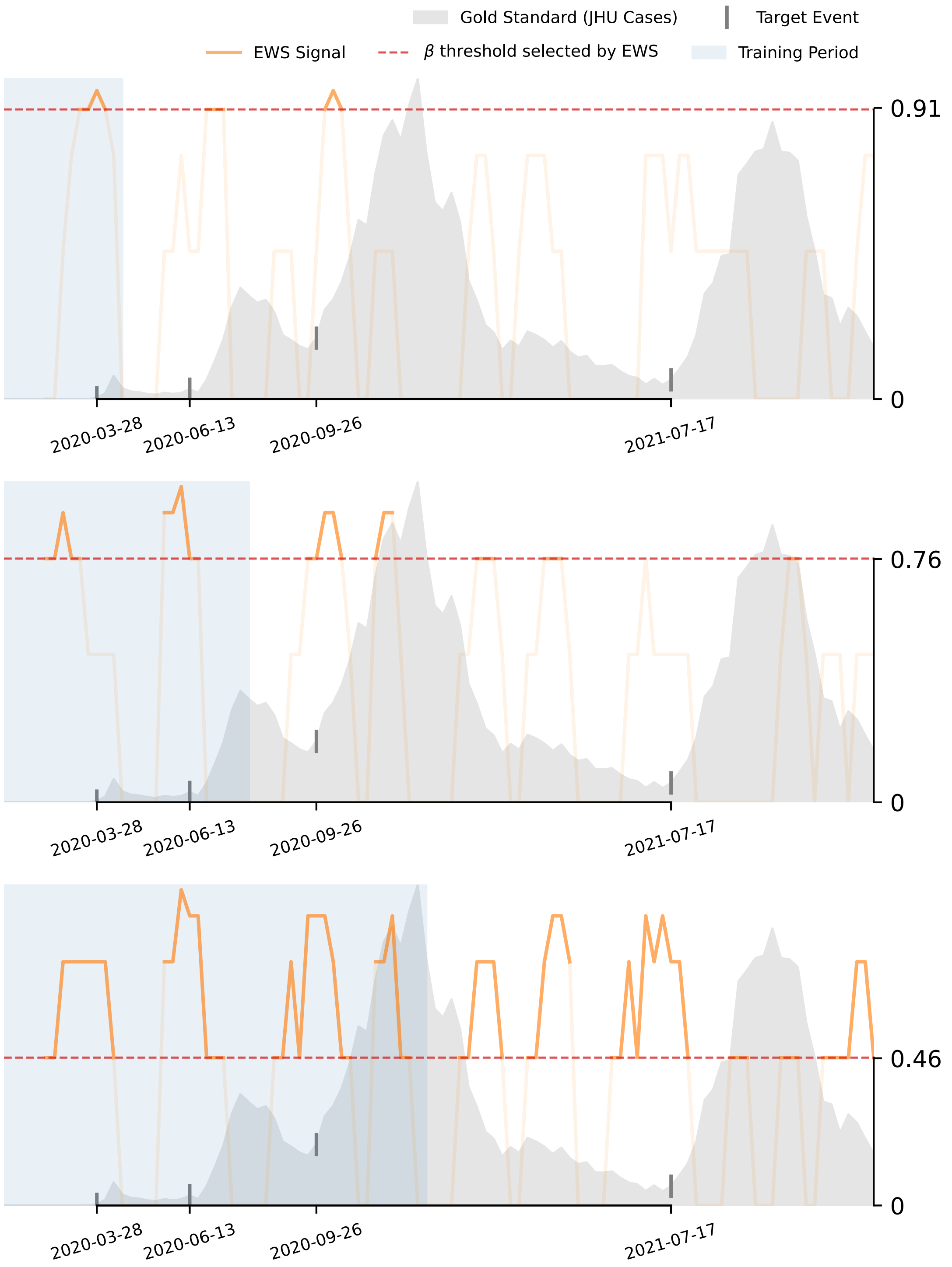

Model 1

(JHU) cases
(GT) covid
(GT) how long does covid last
(GT) covid-19
(GT) quarantine
(GT) fever

Performance as a function of β

	Training			Out of sample		
β	tp	fp	fn	tp	fp	fn
1.00	0	0	1	0	0	3
0.99	0	0	1	0	0	3
0.96	0	0	1	0	1	2
0.91	1	0	0	0	6	1
0.76	1	1	0	2	15	0
0.46	1	2	0	3	36	0
0.00	1	2	0	3	68	0

Model 2

(JHU) cases
(GT) covid-19
(GT) Chest pain
(GT) Chills
(GT) Otitis

β	tp	fp	fn	tp	fp	fn
0.99	0	0	2	0	0	2
0.96	1	0	1	0	0	2
0.91	1	0	1	0	4	1
0.76	2	0	0	2	14	0
0.46	2	3	0	2	32	0
0.00	2	5	0	2	60	0

Model 3

(JHU) cases
(GT) Chest pain
(GT) Chills
(GT) covid-19
(GT) covid symptoms
(GT) fever

β	tp	fp	fn	tp	fp	fn
1.00	0	0	3	0	0	1
0.99	0	0	3	0	0	1
0.96	1	0	2	0	0	1
0.91	1	0	2	1	2	0
0.76	2	3	1	1	10	0
0.46	3	7	0	1	28	0
0.00	3	18	0	1	46	0

Proxies selected by the Multiple Sources method

Model 1

(JHU) cases
(GT) covid
(GT) how long does covid last
(GT) covid-19
(GT) covid symptoms
(GT) chest tightness

Model 2

(JHU) cases
(GT) Anosmia
(GT) Nasal congestion
(GT) Viral pneumonia
(GT) covid
(GT) how long does covid last

Model 3

(GT) how long does covid last
(GT) covid symptoms
(GT) Hyperventilation
(GT) Nasal congestion
(GT) Acute bronchitis
(GT) Cough

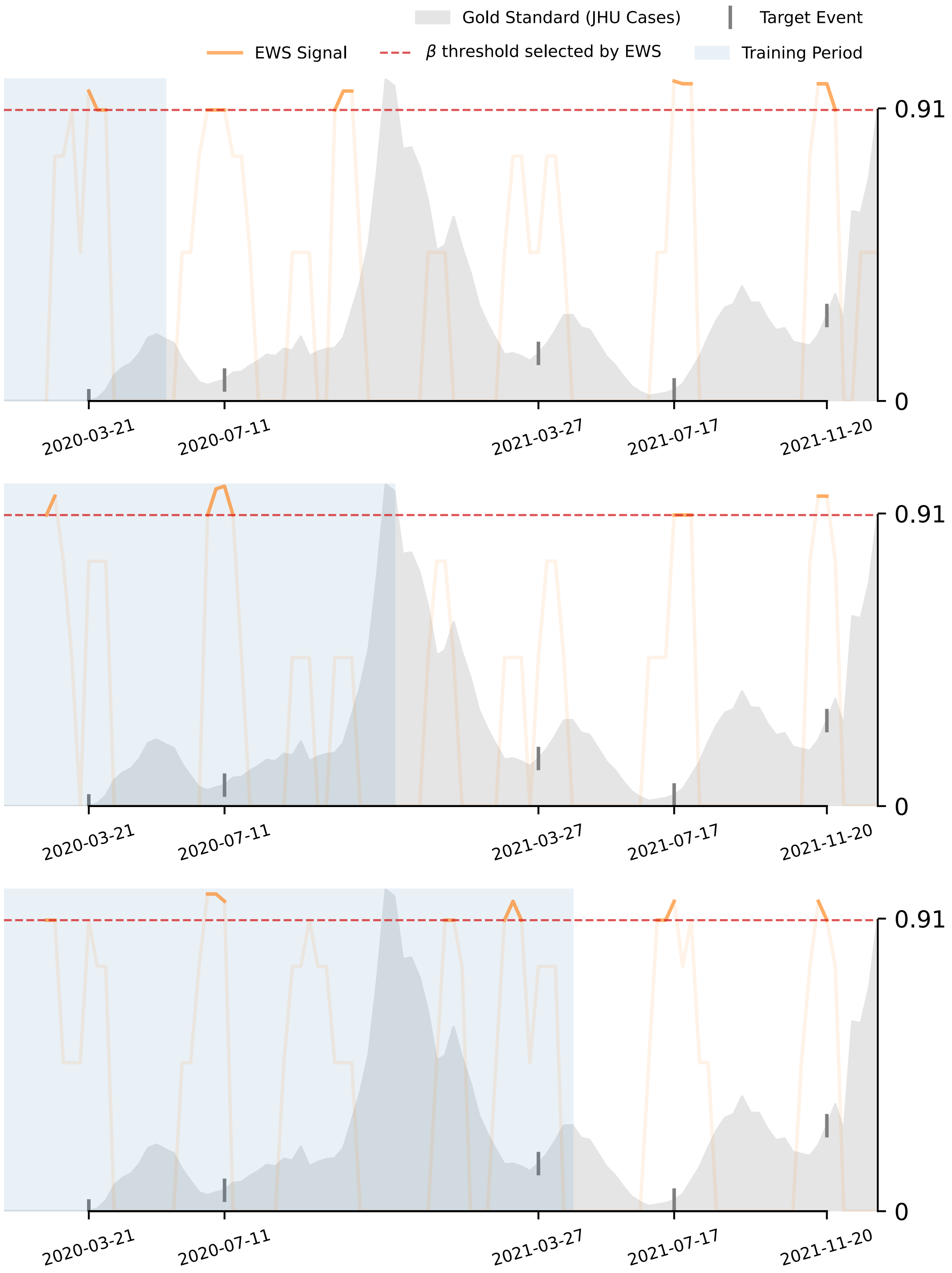

Performance as a function of β

Model 1

β	Training tp	Training fp	Training fn	Out of sample tp	Out of sample fp	Out of sample fn
1.00	0	0	1	1	0	3
0.99	0	0	1	2	2	2
0.96	0	0	1	2	4	2
0.91	1	0	0	3	6	1
0.76	1	2	0	4	10	0
0.46	1	2	0	4	22	0
0.00	1	2	0	4	56	0

Model 2

β	Training tp	Training fp	Training fn	Out of sample tp	Out of sample fp	Out of sample fn
1.00	0	0	2	0	0	3
0.99	1	0	1	0	0	3
0.96	1	0	1	1	0	2
0.91	2	0	0	2	2	1
0.76	2	2	0	2	7	0
0.46	2	4	0	3	10	0
0.00	2	11	0	3	36	0

Model 3

β	Training tp	Training fp	Training fn	Out of sample tp	Out of sample fp	Out of sample fn
1.00	0	0	3	0	0	2
0.99	0	0	3	0	0	2
0.96	1	0	2	2	0	0
0.91	2	0	1	2	1	0
0.76	3	4	0	2	3	0
0.46	3	13	0	2	5	0
0.00	3	20	0	2	22	0

Proxies selected by the Multiple Sources method

Model 1

(JHU) cases
(GT) covid
(GT) covid-19
(GT) covid symptoms
(GT) chest tightness
(GT) covid-19 who

Model 2

(JHU) cases
(GT) covid
(GT) covid-19
(GT) covid symptoms
(GT) chest tightness
(GT) fever

Model 3

(JHU) cases
(GT) covid
(GT) covid symptoms
(GT) chest tightness
(GT) Acute bronchitis
(GT) Ageusia

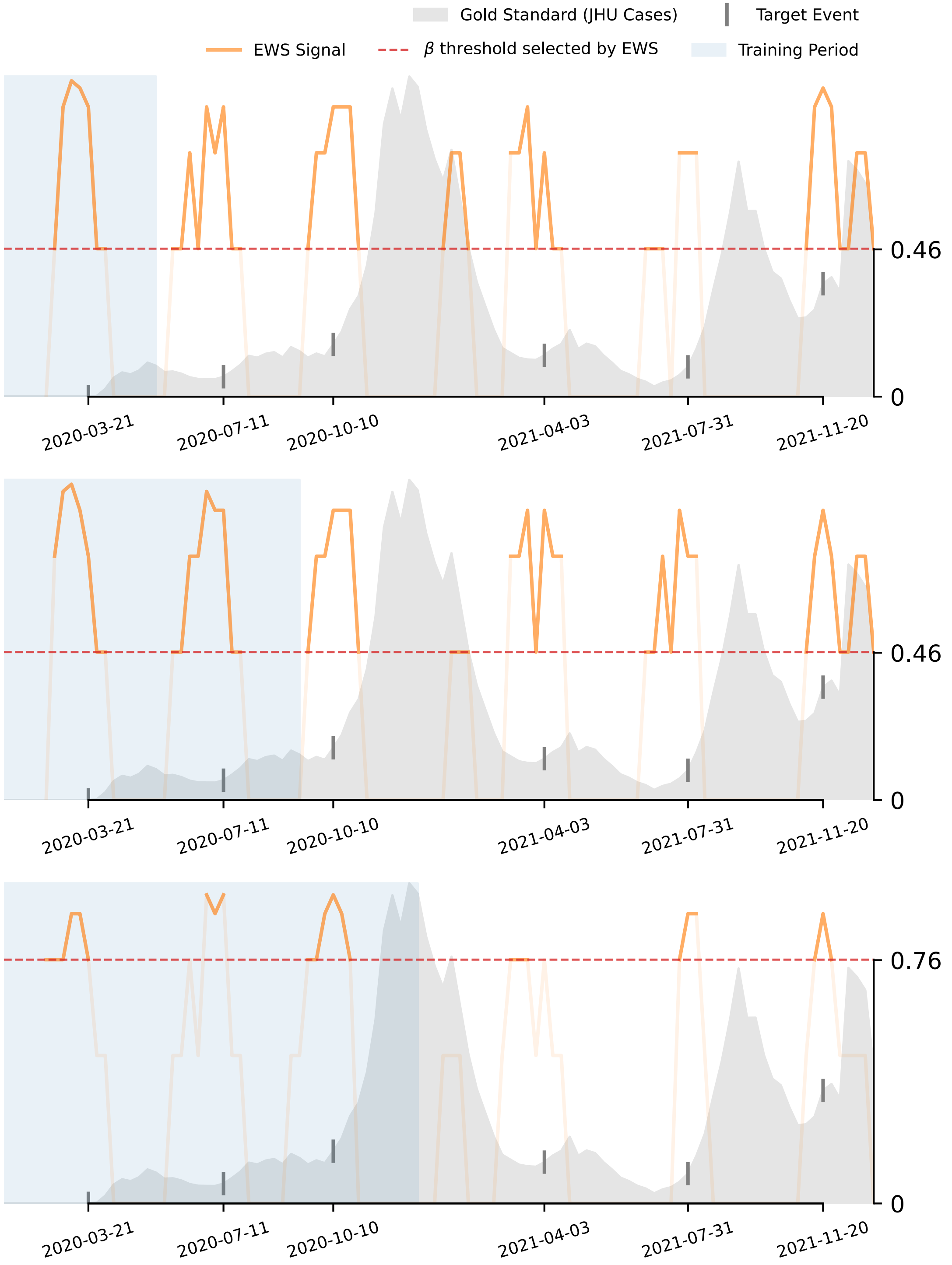

Performance as a function of β

Model 1

β	Training tp	Training fp	Training fn	Out of sample tp	Out of sample fp	Out of sample fn
1.00	0	0	1	0	0	5
0.99	0	0	1	0	0	5
0.96	1	0	0	1	0	4
0.91	1	0	0	4	3	1
0.76	1	0	0	5	8	0
0.46	1	0	0	5	18	0
0.00	1	2	0	5	50	0

Model 2

β	Training tp	Training fp	Training fn	Out of sample tp	Out of sample fp	Out of sample fn
1.00	0	0	2	0	0	4
0.99	0	0	2	0	0	4
0.96	1	0	1	0	0	4
0.91	2	0	0	4	2	0
0.76	2	0	0	4	8	0
0.46	2	0	0	4	15	0
0.00	2	6	0	4	43	0

Model 3

β	Training tp	Training fp	Training fn	Out of sample tp	Out of sample fp	Out of sample fn
1.00	0	0	3	0	0	3
0.99	0	0	3	0	0	3
0.96	0	0	3	0	0	3
0.91	2	0	1	2	1	1
0.76	3	1	0	3	2	0
0.46	3	3	0	3	12	0
0.00	3	10	0	3	33	0

Proxies selected by the Multiple Sources method

Performance as a function of β

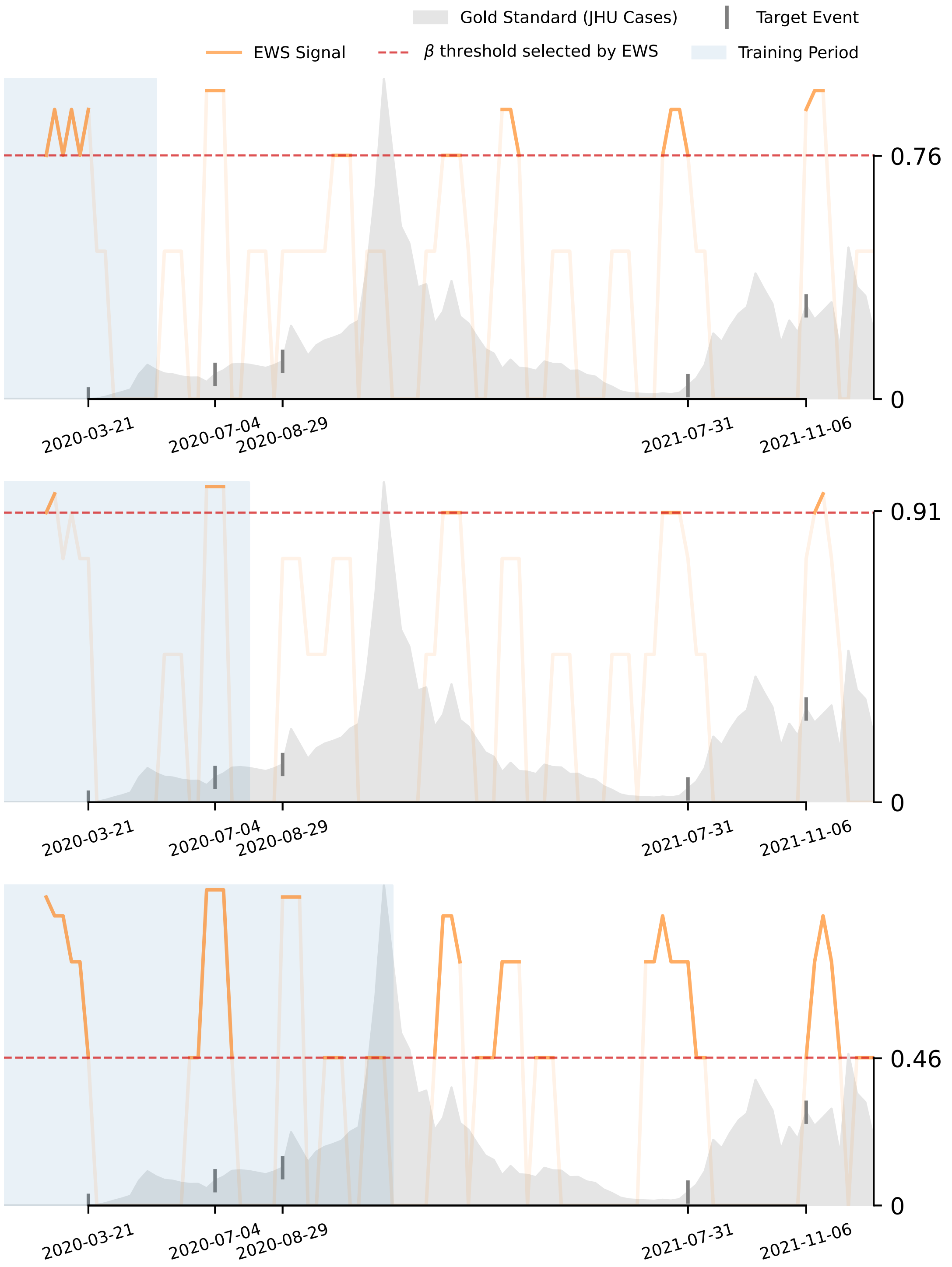

Model 1

(GT) covid
(GT) how long does covid last
(GT) covid-19
(GT) covid symptoms
(GT) fever
(GT) Acute bronchitis

β	Training tp	Training fp	Training fn	Out of sample tp	Out of sample fp	Out of sample fn
1.00	0	0	1	0	0	4
0.99	0	0	1	0	0	4
0.96	0	0	1	1	3	2
0.91	0	0	1	3	5	1
0.76	1	0	0	3	12	1
0.46	1	1	0	4	36	0
0.00	1	3	0	4	57	0

Model 2

(GT) covid
(GT) covid-19
(GT) covid symptoms
(GT) fever
(GT) Acute bronchitis
(GT) Asthma

β	Training tp	Training fp	Training fn	Out of sample tp	Out of sample fp	Out of sample fn
1.00	0	0	2	0	0	3
0.99	0	0	2	0	0	3
0.96	1	1	1	0	1	2
0.91	2	1	0	1	5	1
0.76	2	2	0	3	14	0
0.46	2	2	0	3	29	0
0.00	2	4	0	3	56	0

Model 3

(GT) covid symptoms
(GT) Asthma
Twitter (state level signal)
(GT) Acute bronchitis
(GT) Anosmia
(GT) Nasal congestion

β	Training tp	Training fp	Training fn	Out of sample tp	Out of sample fp	Out of sample fn
1.00	0	0	3	0	0	2
0.99	0	0	3	0	0	2
0.96	1	1	2	0	0	2
0.91	2	4	1	1	3	0
0.76	3	4	0	1	9	0
0.46	3	4	0	2	22	0
0.00	3	11	0	2	43	0

Proxies selected by the Multiple Sources method

Model 1

(GT) covid
(GT) covid-19
(GT) covid symptoms
(GT) quarantine
(GT) Acute bronchitis
(GT) Bronchitis

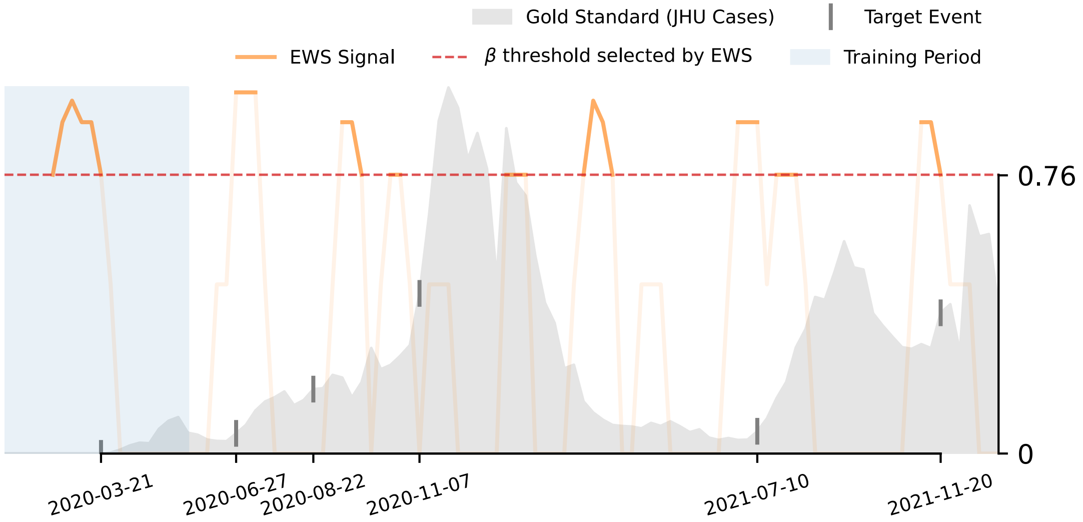

Performance as a function of β

β	Training tp	Training fp	Training fn	Out of sample tp	Out of sample fp	Out of sample fn
1.00	0	0	1	0	0	5
0.99	0	0	1	2	1	3
0.96	0	0	1	2	2	3
0.91	1	0	0	4	5	1
0.76	1	0	0	5	13	0
0.46	1	1	0	5	26	0
0.00	1	2	0	5	51	0

Model 2

(GT) covid
(GT) covid-19
(GT) covid symptoms
(GT) quarantine
(GT) Acute bronchitis
(GT) Chest pain

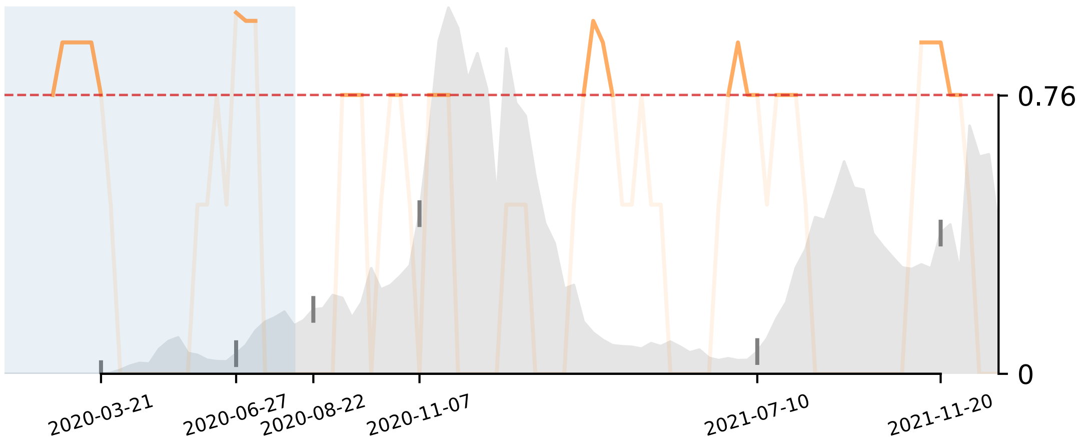

β	Training tp	Training fp	Training fn	Out of sample tp	Out of sample fp	Out of sample fn
1.00	0	0	2	0	0	4
0.99	0	0	2	0	0	4
0.96	1	0	1	0	1	4
0.91	1	2	1	2	2	2
0.76	2	2	0	3	15	1
0.46	2	3	0	3	26	1
0.00	2	4	0	4	50	0

Model 3

(GT) Wheeze
(GT) covid-19
(GT) quarantine
(GT) Chest pain
(GT) Nasal congestion
(GT) covid

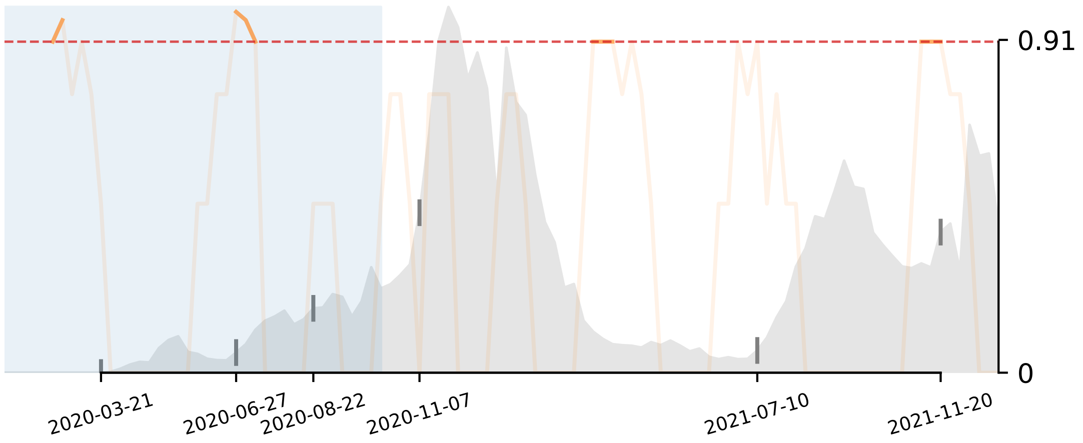

β	Training tp	Training fp	Training fn	Out of sample tp	Out of sample fp	Out of sample fn
1.00	0	0	3	0	0	3
0.99	0	0	3	0	0	3
0.96	1	0	2	0	0	3
0.91	2	1	1	2	4	1
0.76	2	3	1	3	14	0
0.46	2	3	1	3	22	0
0.00	3	6	0	3	46	0

Proxies selected by the Multiple Sources method

Model 1

(JHU) cases
(GT) covid
(GT) covid-19
(GT) covid symptoms
(GT) quarantine
(GT) fever

Model 2

(GT) covid
(GT) covid-19
(GT) covid symptoms
(GT) quarantine
(GT) fever
(GT) Acute bronchitis

Model 3

(GT) covid symptoms
(GT) quarantine
(GT) Acute bronchitis
(GT) Pneumonia
(GT) covid
(GT) covid-19

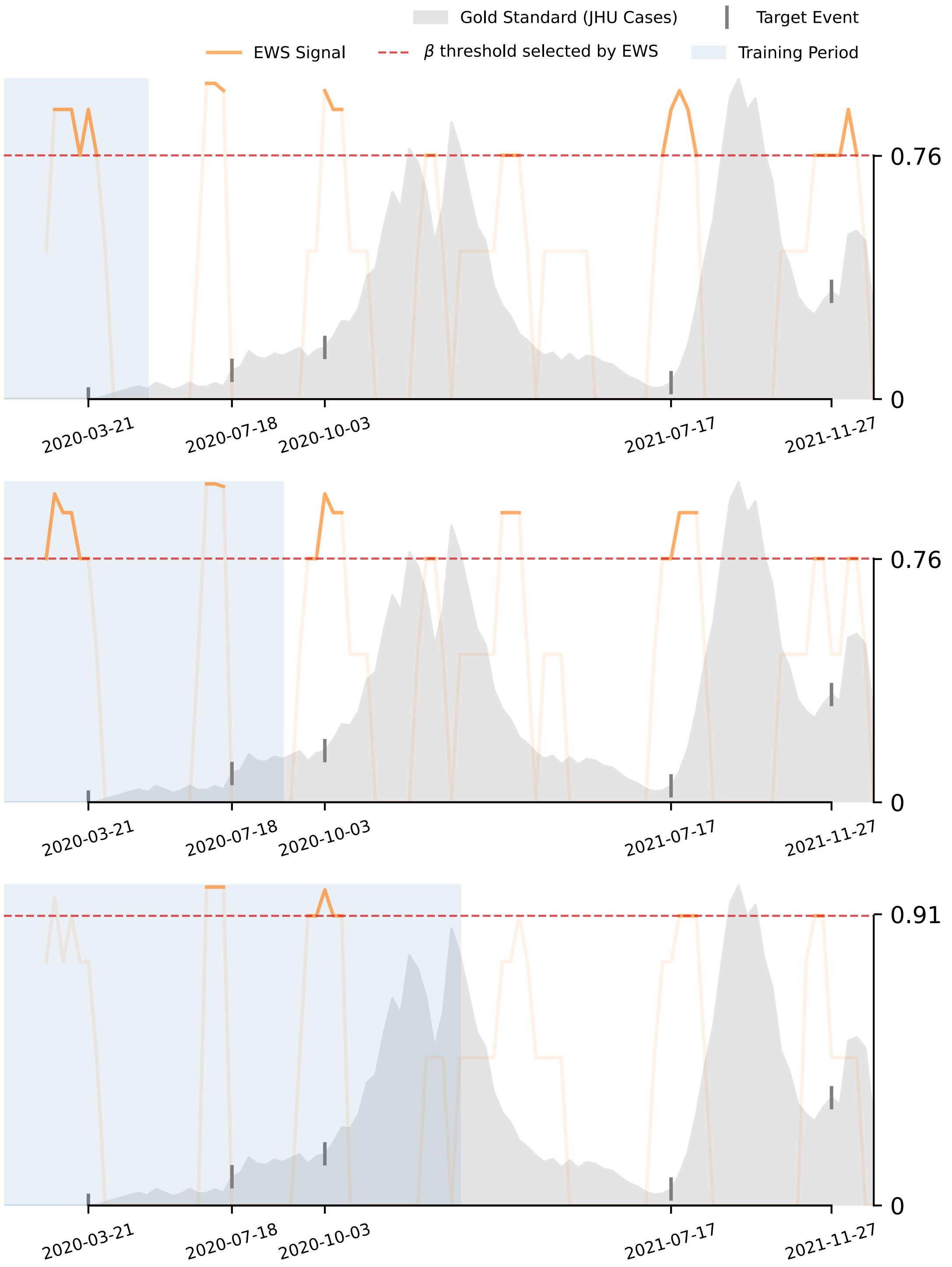

Performance as a function of β

Model 1

β	Training tp	Training fp	Training fn	Out of sample tp	Out of sample fp	Out of sample fn
1.00	0	0	1	0	0	4
0.99	0	0	1	1	0	3
0.96	0	0	1	2	1	1
0.91	0	0	1	3	5	0
0.76	1	0	0	4	13	0
0.46	1	1	0	4	31	0
0.00	1	3	0	4	58	0

Model 2

β	Training tp	Training fp	Training fn	Out of sample tp	Out of sample fp	Out of sample fn
1.00	0	0	2	0	0	3
0.99	1	0	1	0	0	3
0.96	1	0	1	1	0	2
0.91	2	0	0	1	8	1
0.76	2	0	0	3	12	0
0.46	2	1	0	3	29	0
0.00	2	2	0	3	51	0

Model 3

β	Training tp	Training fp	Training fn	Out of sample tp	Out of sample fp	Out of sample fn
1.00	0	0	3	0	0	2
0.99	1	0	2	0	0	2
0.96	2	0	1	0	0	2
0.91	3	0	0	1	4	0
0.76	3	2	0	2	7	0
0.46	3	3	0	2	19	0
0.00	3	8	0	2	35	0

Louisiana

Proxies selected by the Multiple Sources method

Model 1

(GT) covid
(GT) covid-19
(GT) chest pain
(GT) covid symptoms
(GT) covid-19 who
(GT) Abdominal pain

Model 2

(GT) Abdominal pain
(GT) Diarrhea

Model 3

(GT) Abdominal pain
(GT) Diarrhea
(GT) covid
(GT) chest pain
(GT) covid symptoms
(GT) Ageusia

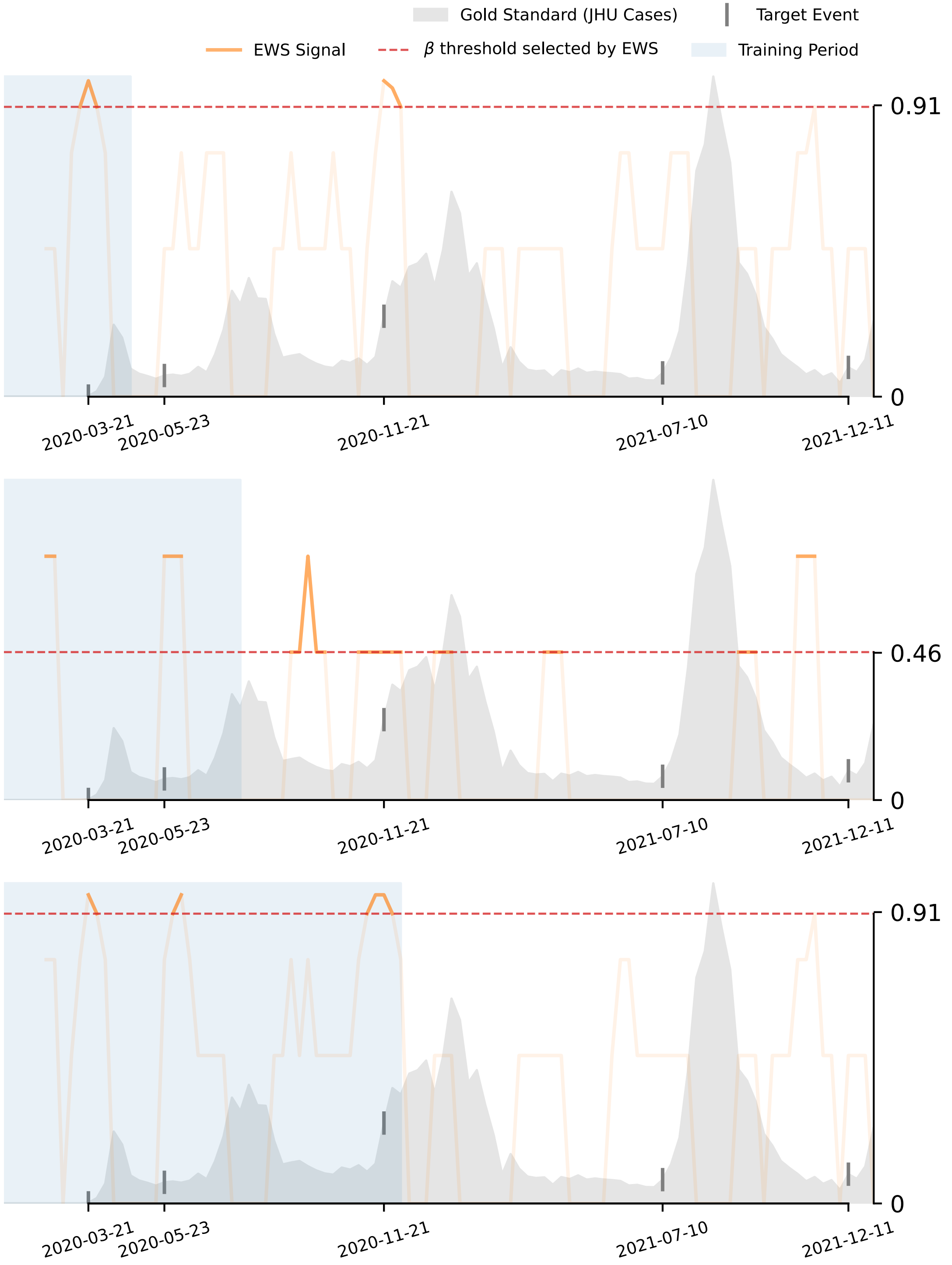

Performance as a function of β

Model 1

β	Training tp	Training fp	Training fn	Out of sample tp	Out of sample fp	Out of sample fn
1.00	0	0	1	0	0	4
0.99	0	0	1	1	0	3
0.96	1	0	0	1	1	3
0.91	1	0	0	2	2	2
0.76	1	1	0	3	10	0
0.46	1	2	0	4	36	0
0.00	1	3	0	4	63	0

Model 2

β	Training tp	Training fp	Training fn	Out of sample tp	Out of sample fp	Out of sample fn
0.76	0	0	2	1	1	2
0.46	2	3	0	2	16	1
0.00	2	3	0	3	54	0

Model 3

β	Training tp	Training fp	Training fn	Out of sample tp	Out of sample fp	Out of sample fn
1.00	0	0	3	0	0	2
0.99	0	0	3	0	0	2
0.96	0	0	3	0	0	2
0.91	2	1	1	1	0	1
0.76	2	4	1	2	0	0
0.46	3	10	0	2	20	0
0.00	3	21	0	2	42	0

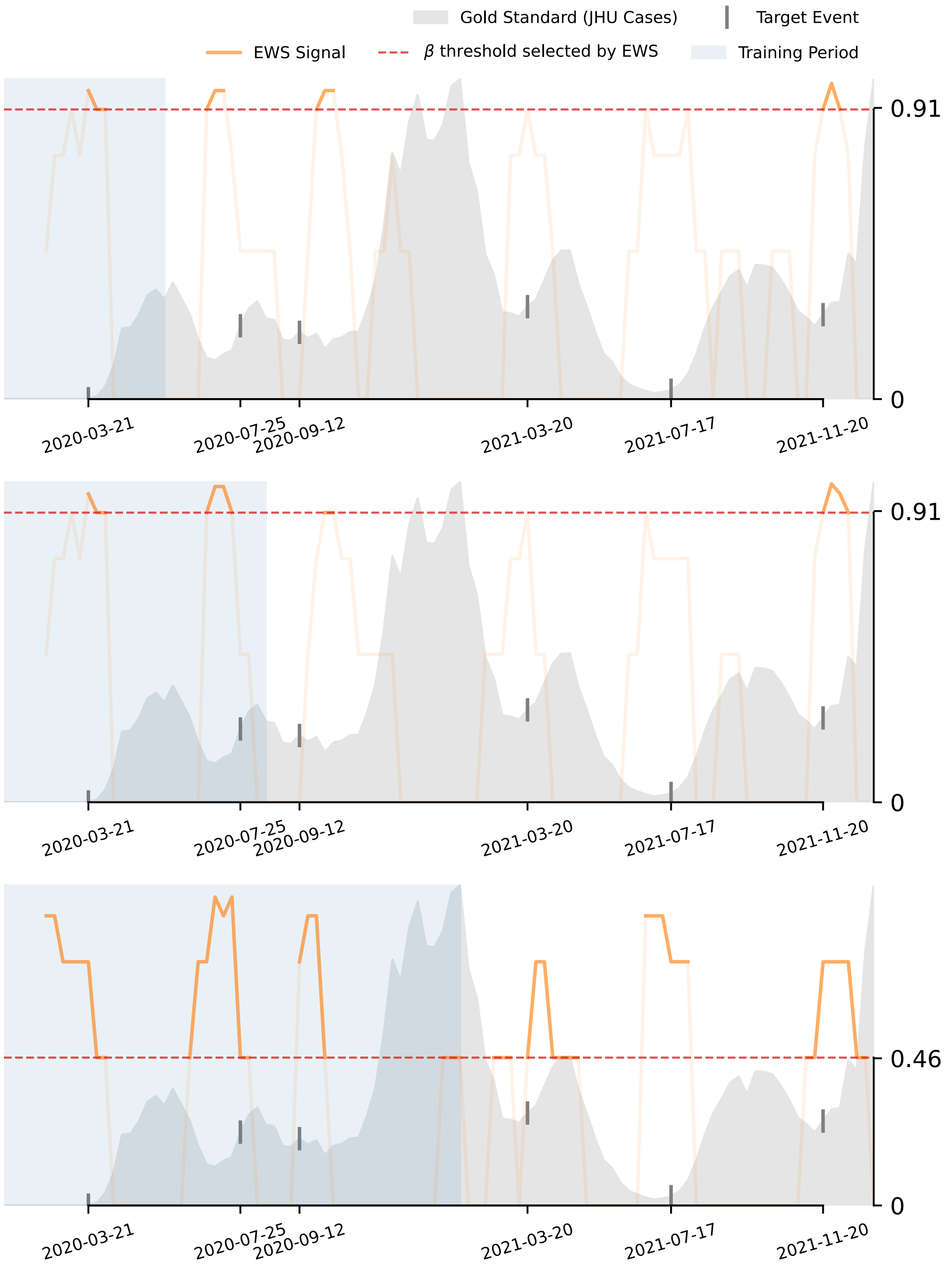

Proxies selected by the Multiple Sources method

Performance as a function of β

Model 1

(JHU) cases
(GT) covid
(GT) how long does covid last
(GT) covid-19
(GT) chest pain
(GT) covid symptoms

	Training			Out of sample		
β	tp	fp	fn	tp	fp	fn
1.00	0	0	1	0	0	5
0.99	0	0	1	0	1	4
0.96	0	0	1	1	3	3
0.91	1	0	0	4	6	0
0.76	1	2	0	4	12	0
0.46	1	2	0	5	24	0
0.00	1	3	0	5	49	0

Model 2

(JHU) cases
(GT) covid
(GT) covid-19
(GT) chest pain
(GT) covid symptoms
(GT) chest tightness

β	tp	fp	fn	tp	fp	fn
1.00	0	0	2	0	1	3
0.99	0	0	2	0	1	3
0.96	1	0	1	0	2	3
0.91	2	0	0	3	5	1
0.76	2	2	0	3	10	0
0.46	2	2	0	3	21	0
0.00	2	4	0	4	47	0

Model 3

(GT) Bronchitis
(GT) Asthma
(GT) covid-19 who
(GT) Hyperventilation
(GT) Nasal congestion
(JHU) cases

β	tp	fp	fn	tp	fp	fn
1.00	0	0	3	0	0	3
0.99	0	0	3	0	0	3
0.96	0	0	3	0	0	3
0.91	1	0	2	1	0	2
0.76	2	3	1	2	7	0
0.46	3	4	0	3	13	0
0.00	3	12	0	3	28	0

Proxies selected by the Multiple Sources method

Model 1

(GT) covid
(GT) how long does covid last
(GT) covid-19
(GT) chest pain
(GT) covid symptoms
(GT) quarantine

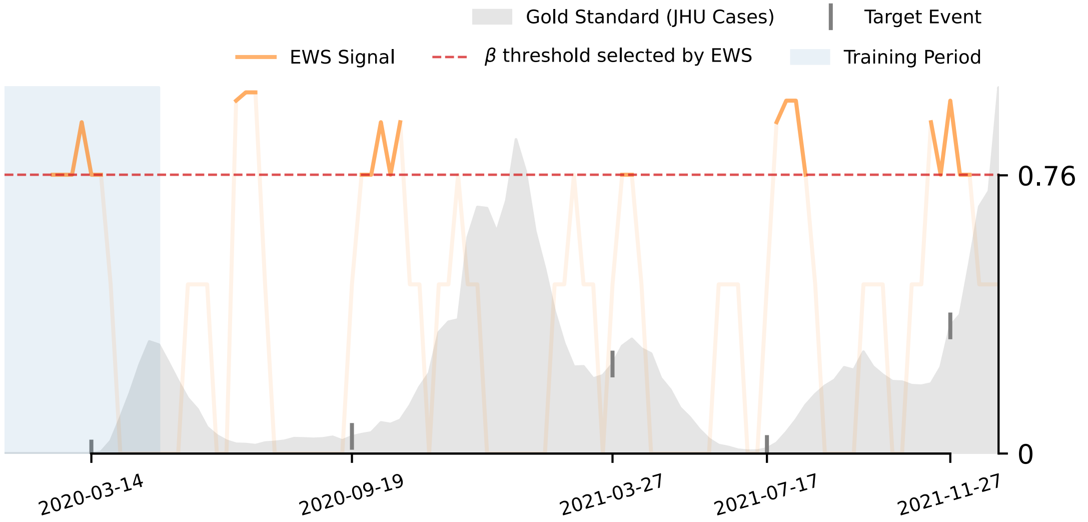

Performance as a function of β

β	Training tp	Training fp	Training fn	Out of sample tp	Out of sample fp	Out of sample fn
1.00	0	0	1	0	0	4
0.99	0	0	1	0	2	4
0.96	0	0	1	1	5	2
0.91	0	0	1	1	8	2
0.76	1	0	0	2	17	0
0.46	1	1	0	4	35	0
0.00	1	2	0	4	59	0

Model 2

(GT) covid-19
(GT) Ageusia
(GT) Asthma
(GT) Wheeze
(GT) Anosmia
(GT) Fever

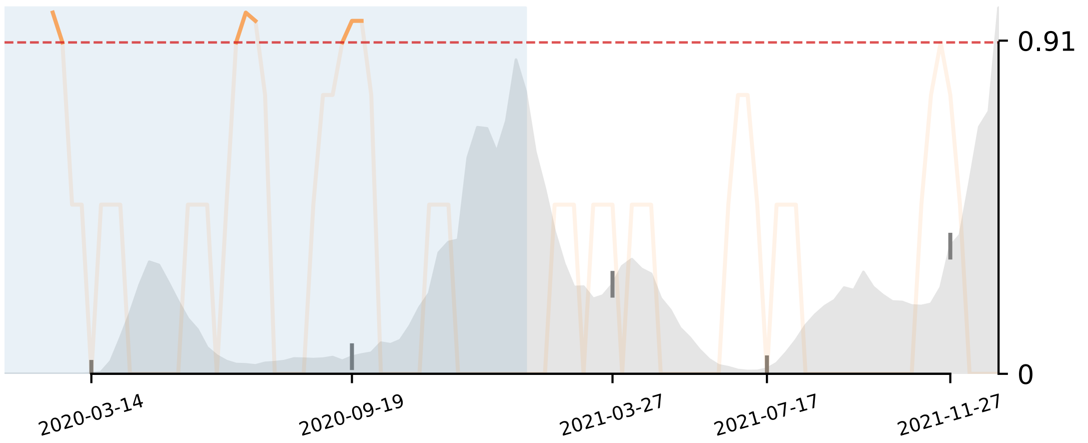

β	Training tp	Training fp	Training fn	Out of sample tp	Out of sample fp	Out of sample fn
1.00	0	0	2	0	0	3
0.99	0	0	2	0	0	3
0.96	1	1	1	0	0	3
0.91	2	3	0	1	0	2
0.76	2	4	0	2	0	1
0.46	2	6	0	3	7	0
0.00	2	16	0	3	28	0

Model 3

(GT) covid-19
(GT) Fever
(GT) Acute bronchitis
(GT) Ageusia
(GT) Asthma
(GT) Cough

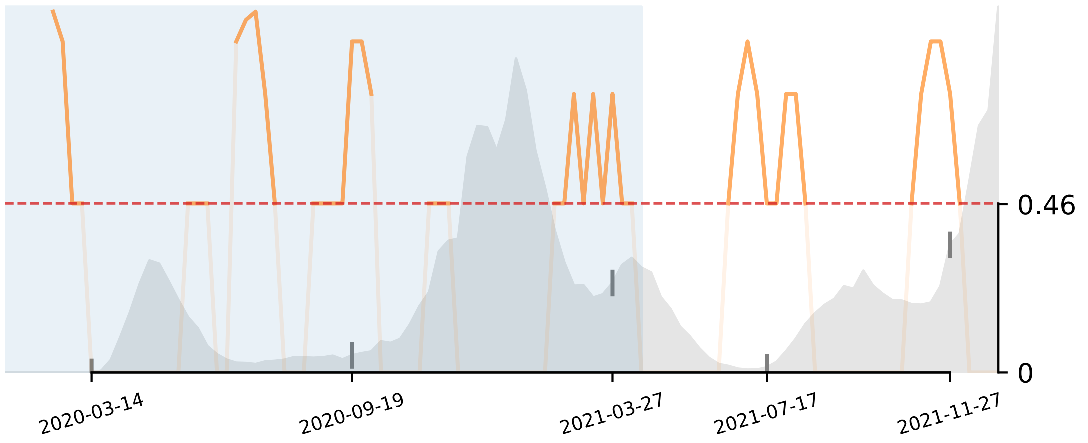

β	Training tp	Training fp	Training fn	Out of sample tp	Out of sample fp	Out of sample fn
1.00	0	0	3	0	0	2
0.99	0	0	3	0	0	2
0.96	1	1	2	0	0	2
0.91	1	2	2	2	0	0
0.76	2	4	1	2	2	0
0.46	3	6	0	2	5	0
0.00	3	17	0	2	23	0

Michigan

Proxies selected by the Multiple Sources method

Model 1

(GT) covid
(GT) how long does covid last
(GT) covid-19
(GT) chest pain
(GT) covid symptoms
(GT) chest tightness

Model 2

(GT) covid-19 who
(GT) Acute bronchitis
(GT) Bronchitis
(GT) Cough
(GT) Nasal congestion
(GT) Pneumonia

Model 3

(GT) Acute bronchitis
(GT) Bronchitis
(GT) Cough
(GT) Nasal congestion
(GT) Pneumonia
(GT) Asthma

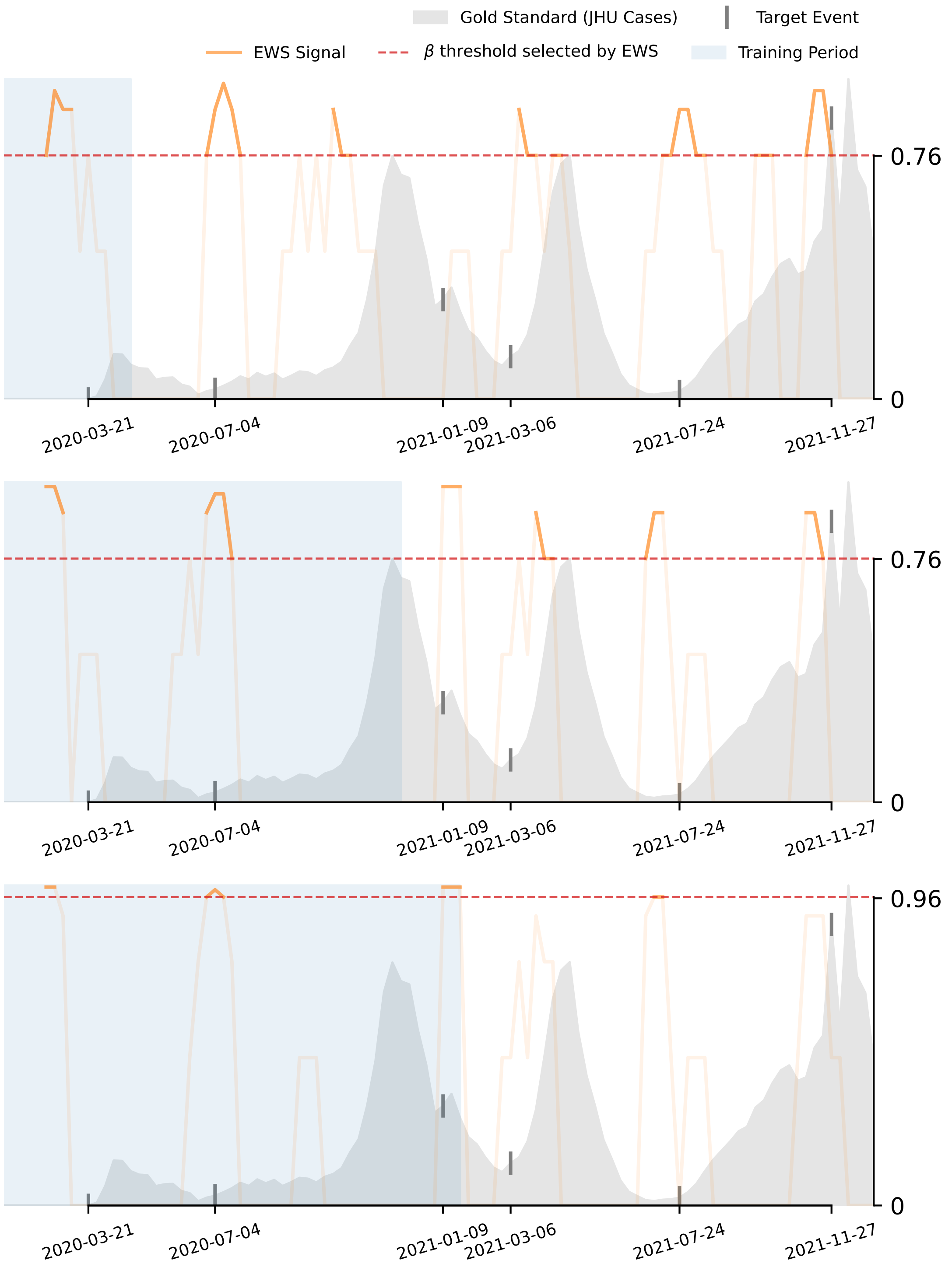

Performance as a function of β

Model 1

β	Training tp	Training fp	Training fn	Out of sample tp	Out of sample fp	Out of sample fn
1.00	0	0	1	0	0	5
0.99	0	0	1	0	1	4
0.96	0	0	1	1	1	3
0.91	1	0	0	3	5	1
0.76	1	0	0	3	19	1
0.46	1	1	0	4	31	0
0.00	1	3	0	5	53	0

Model 2

β	Training tp	Training fp	Training fn	Out of sample tp	Out of sample fp	Out of sample fn
1.00	0	0	2	0	0	4
0.99	0	0	2	2	1	2
0.96	1	1	1	2	1	2
0.91	2	2	0	4	2	0
0.76	2	2	0	4	5	0
0.46	2	3	0	4	9	0
0.00	2	5	0	4	30	0

Model 3

β	Training tp	Training fp	Training fn	Out of sample tp	Out of sample fp	Out of sample fn
1.00	0	0	3	0	0	3
0.99	2	3	1	0	0	3
0.96	3	3	0	1	0	2
0.91	3	4	0	2	1	1
0.76	3	4	0	2	4	0
0.46	3	5	0	3	9	0
0.00	3	8	0	3	29	0

Proxies selected by the Multiple Sources method

Model 1

(JHU) cases
(GT) covid
(GT) covid-19
(GT) fever
(GT) Acute bronchitis
(GT) Asthma

Model 2

(JHU) cases
(GT) covid
(GT) covid-19
(GT) fever
(GT) Acute bronchitis
(GT) Asthma

Model 3

(GT) Nasal congestion
(GT) Ageusia
(JHU) cases
(GT) Acute bronchitis
(GT) Asthma
(GT) Wheeze

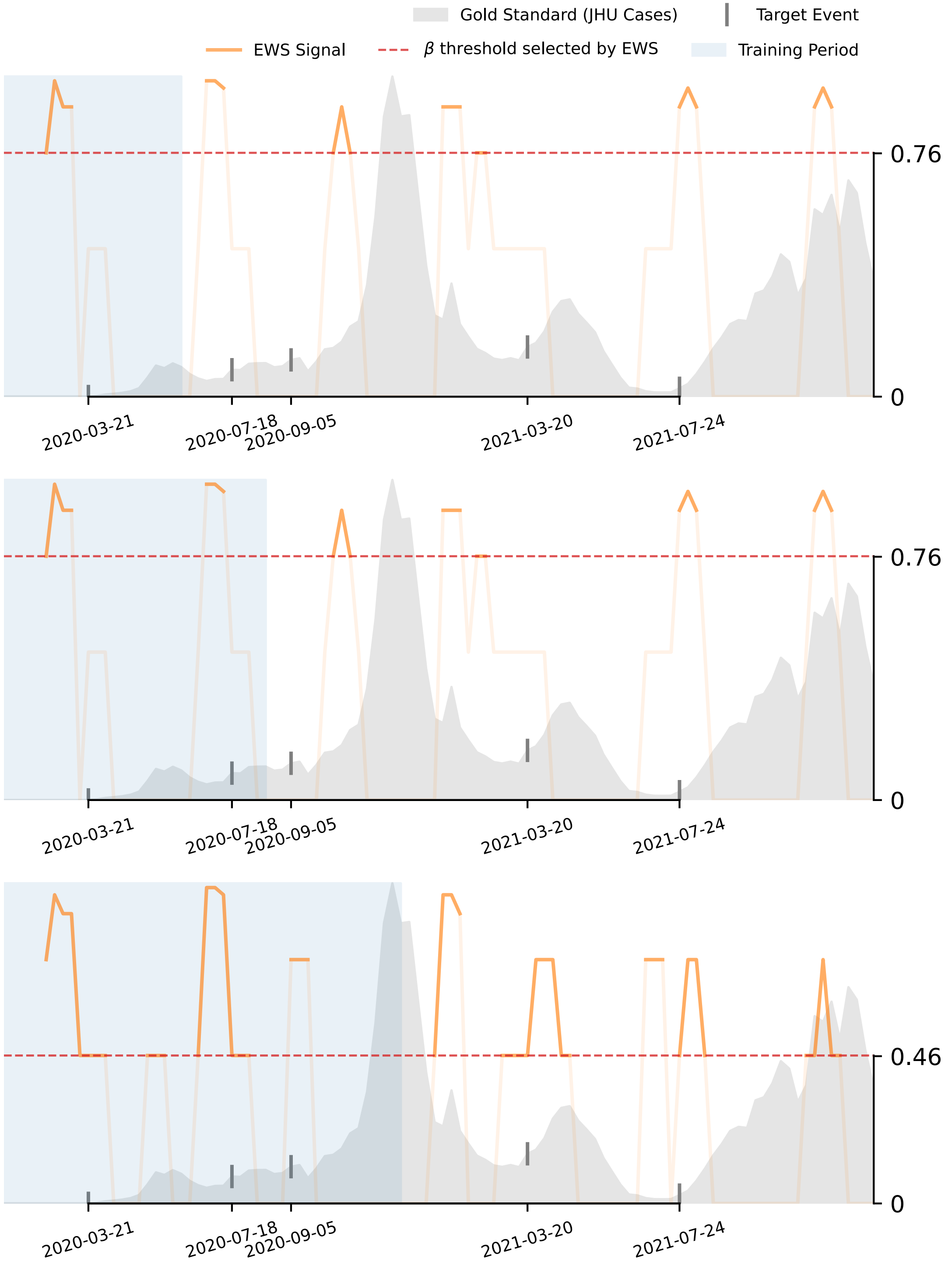

Performance as a function of β

Model 1

β	Training tp	Training fp	Training fn	Out of sample tp	Out of sample fp	Out of sample fn
1.00	0	0	1	0	0	4
0.99	0	0	1	1	0	3
0.96	1	0	0	1	2	2
0.91	1	0	0	2	9	2
0.76	1	0	0	3	11	1
0.46	1	1	0	4	19	0
0.00	1	3	0	4	55	0

Model 2

β	Training tp	Training fp	Training fn	Out of sample tp	Out of sample fp	Out of sample fn
1.00	0	0	2	0	0	3
0.99	0	0	2	0	0	3
0.96	2	0	0	0	2	2
0.91	2	0	0	1	9	2
0.76	2	0	0	2	11	1
0.46	2	1	0	2	19	1
0.00	2	5	0	3	55	0

Model 3

β	Training tp	Training fp	Training fn	Out of sample tp	Out of sample fp	Out of sample fn
1.00	0	0	3	0	0	2
0.99	0	0	3	0	0	2
0.96	1	0	2	0	2	2
0.91	2	0	1	0	3	2
0.76	2	0	1	1	9	0
0.46	3	3	0	2	17	0
0.00	3	10	0	2	42	0

Mississippi

Proxies selected by the Multiple Sources method

Model 1

(GT) covid
(GT) covid-19
(GT) covid symptoms
(GT) Acute bronchitis
(GT) Bronchitis
(GT) Burning Chest Pain

Model 2

(GT) covid symptoms
(GT) fever
(GT) Chest pain
(GT) Hyperventilation

Model 3

(GT) covid symptoms
(GT) Chest pain
(GT) Hyperventilation
(GT) fever
(GT) Anosmia
(GT) covid

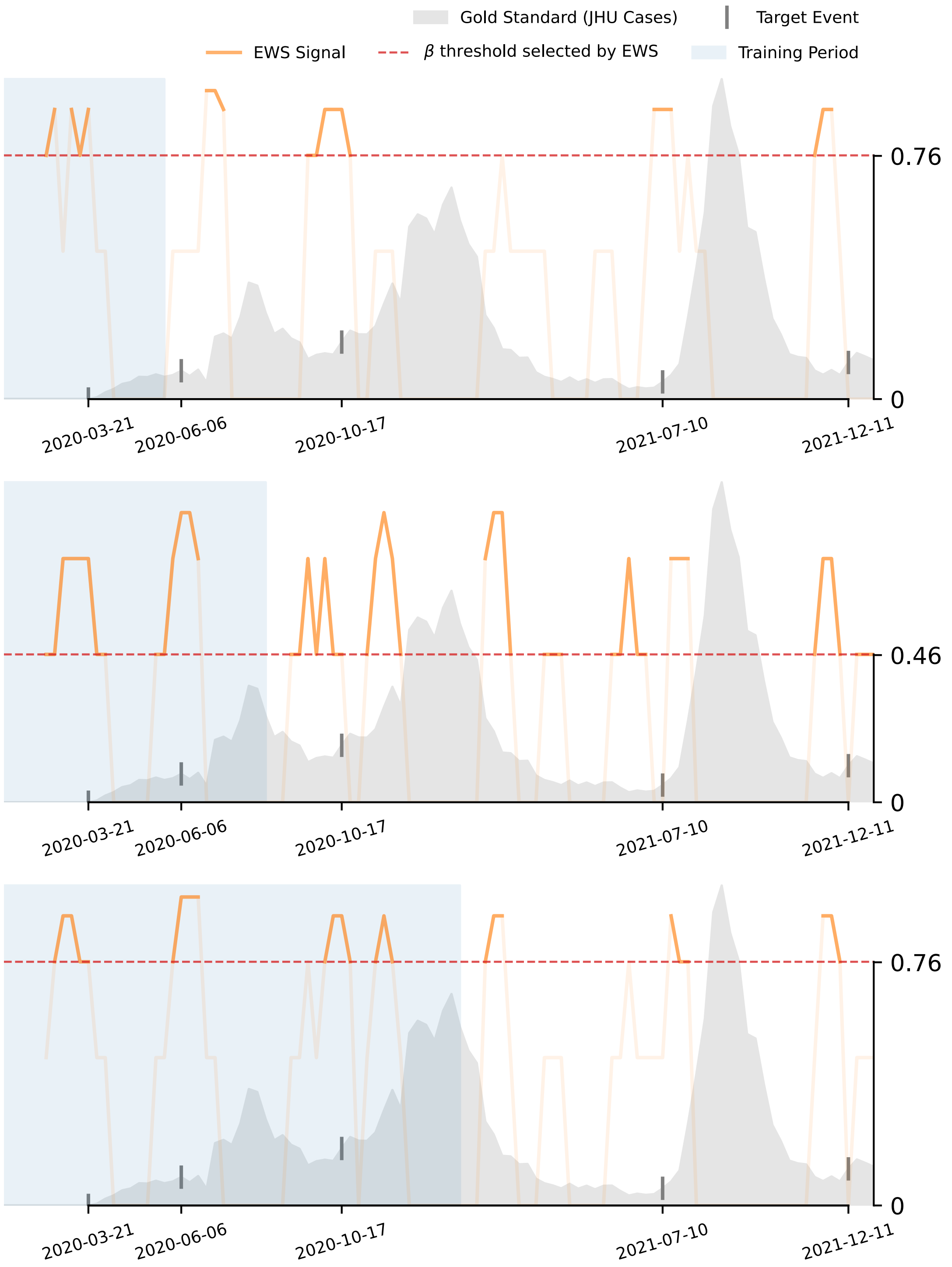

Performance as a function of β

Model 1

β	Training tp	Training fp	Training fn	Out of sample tp	Out of sample fp	Out of sample fn
1.00	0	0	1	0	0	4
0.99	0	0	1	0	0	4
0.96	0	0	1	0	2	4
0.91	0	0	1	3	4	1
0.76	1	0	0	3	7	1
0.46	1	1	0	4	24	0
0.00	1	3	0	4	61	0

Model 2

β	Training tp	Training fp	Training fn	Out of sample tp	Out of sample fp	Out of sample fn
0.96	0	0	2	0	0	3
0.91	0	0	2	0	3	3
0.76	1	1	1	3	9	0
0.46	2	2	0	3	18	0
0.00	2	5	0	3	51	0

Model 3

β	Training tp	Training fp	Training fn	Out of sample tp	Out of sample fp	Out of sample fn
1.00	0	0	3	0	0	2
0.99	0	0	3	0	0	2
0.96	0	0	3	0	0	2
0.91	1	2	2	1	3	0
0.76	3	3	0	2	6	0
0.46	3	6	0	2	13	0
0.00	3	15	0	2	35	0

Missouri

Proxies selected by the Multiple Sources method

Model 1

(JHU) cases
(GT) covid
(GT) covid-19
(GT) chest pain
(GT) covid symptoms
(GT) covid-19 who

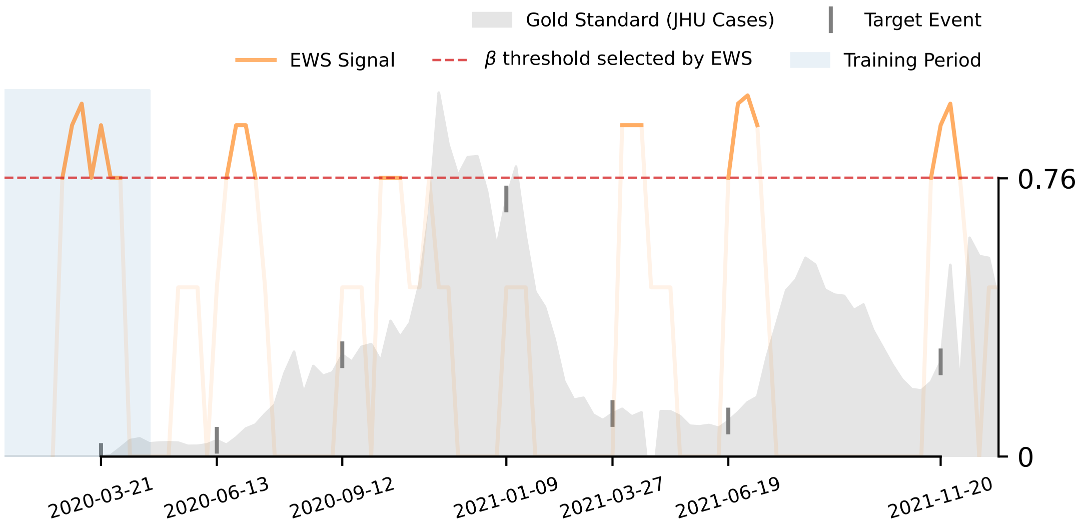

Performance as a function of β

β	Training tp	Training fp	Training fn	Out of sample tp	Out of sample fp	Out of sample fn
1.00	0	0	1	0	0	6
0.99	0	0	1	0	1	5
0.96	0	0	1	0	3	4
0.91	1	0	0	1	9	2
0.76	1	0	0	2	16	2
0.46	1	2	0	5	30	0
0.00	1	2	0	6	46	0

Model 2

(GT) chest pain
(GT) covid symptoms
(GT) Chest pain
(GT) Nasal congestion
(GT) Otitis
(GT) Sore throat

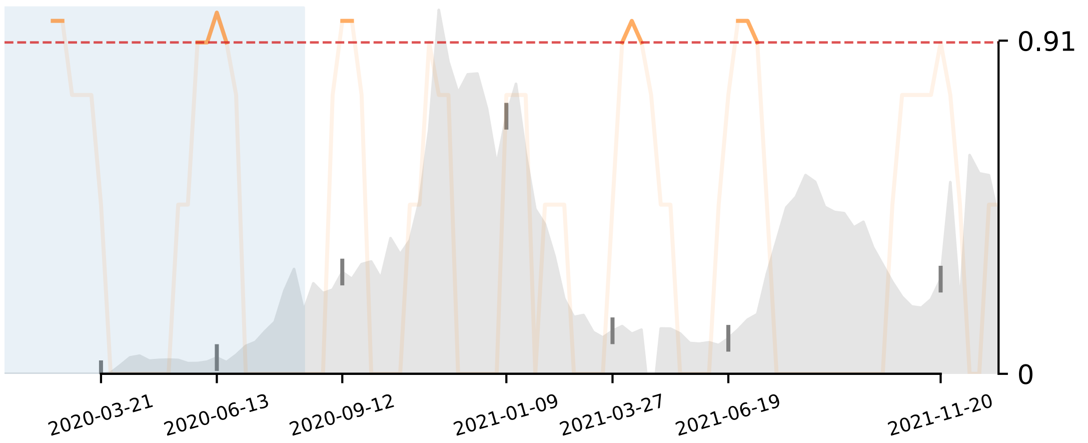

β	Training tp	Training fp	Training fn	Out of sample tp	Out of sample fp	Out of sample fn
1.00	0	0	2	0	0	5
0.99	0	0	2	0	0	5
0.96	1	0	1	1	4	2
0.91	2	1	0	2	8	1
0.76	2	2	0	4	14	0
0.46	2	3	0	5	22	0
0.00	2	3	0	5	40	0

Model 3

(GT) covid symptoms
(GT) Nasal congestion
(GT) Sore throat
(GT) Throat irritation
(GT) Chest pain
(GT) chest pain

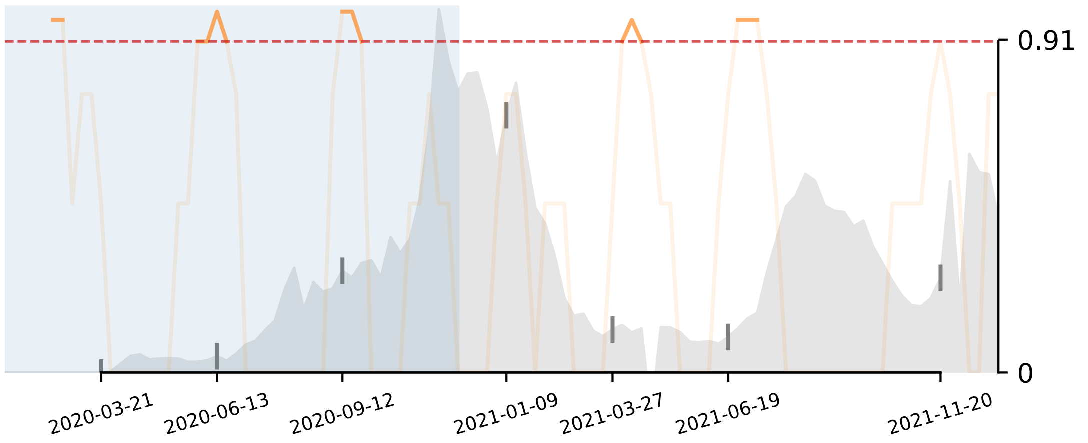

β	Training tp	Training fp	Training fn	Out of sample tp	Out of sample fp	Out of sample fn
1.00	0	0	3	0	0	4
0.99	0	0	3	0	0	4
0.96	2	1	1	0	4	2
0.91	3	2	0	1	6	1
0.76	3	4	0	3	12	0
0.46	3	6	0	4	17	0
0.00	3	10	0	4	30	0

Proxies selected by the Multiple Sources method

Model 1

(GT) covid
(GT) covid-19
(GT) chest pain
(GT) covid symptoms
(GT) quarantine
(GT) fever

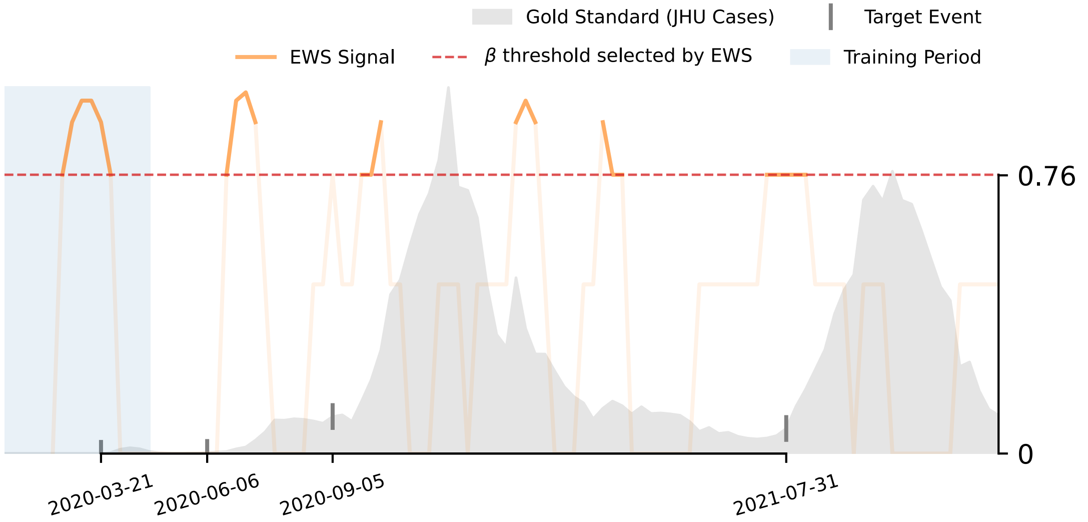

Performance as a function of β

β	Training tp	Training fp	Training fn	Out of sample tp	Out of sample fp	Out of sample fn
1.00	0	0	1	0	0	3
0.99	0	0	1	0	1	3
0.96	0	0	1	0	3	3
0.91	1	0	0	0	8	3
0.76	1	0	0	2	15	0
0.46	1	1	0	2	45	0
0.00	1	1	0	3	68	0

Model 2

(GT) Diarrhea
(GT) Phlegm

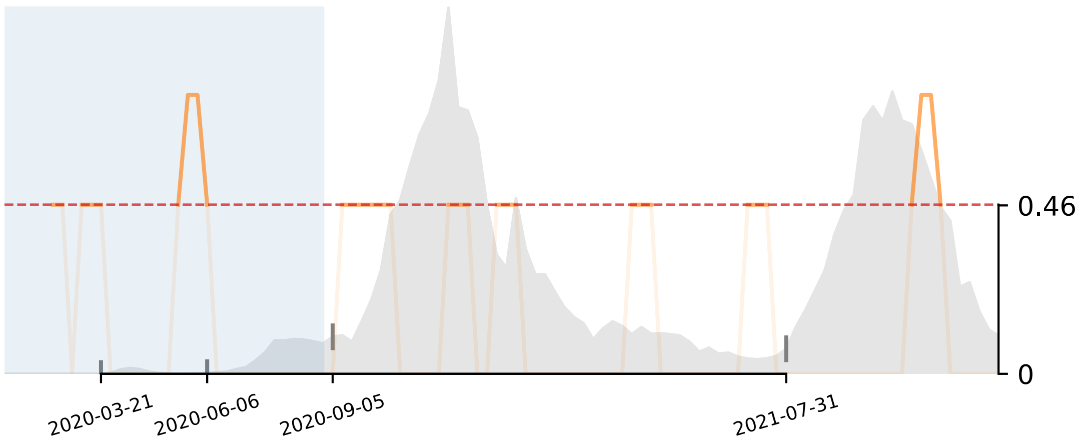

β	Training tp	Training fp	Training fn	Out of sample tp	Out of sample fp	Out of sample fn
0.76	0	0	2	0	2	2
0.46	1	0	1	1	19	0
0.00	2	1	0	2	62	0

Model 3

(GT) Vomiting
(GT) covid
(GT) Nasal congestion
(GT) Phlegm
(GT) Shortness of breath
(GT) fever

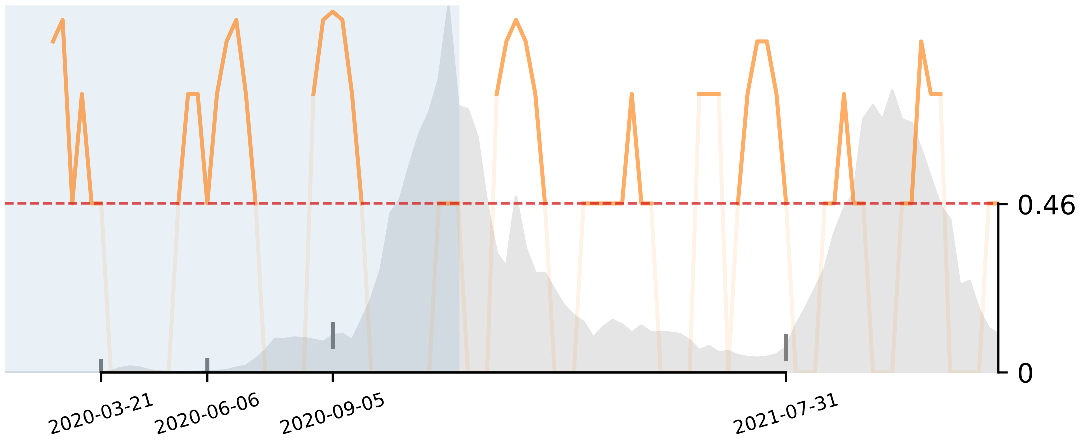

β	Training tp	Training fp	Training fn	Out of sample tp	Out of sample fp	Out of sample fn
1.00	0	0	3	0	0	1
0.99	0	0	3	0	0	1
0.96	1	0	2	0	1	1
0.91	2	2	1	1	4	0
0.76	2	4	1	1	13	0
0.46	3	7	0	1	29	0
0.00	3	12	0	1	49	0

Proxies selected by the Multiple Sources method

Model 1

(JHU) cases
(GT) covid
(GT) covid-19
(GT) chest pain
(GT) quarantine
(GT) fever

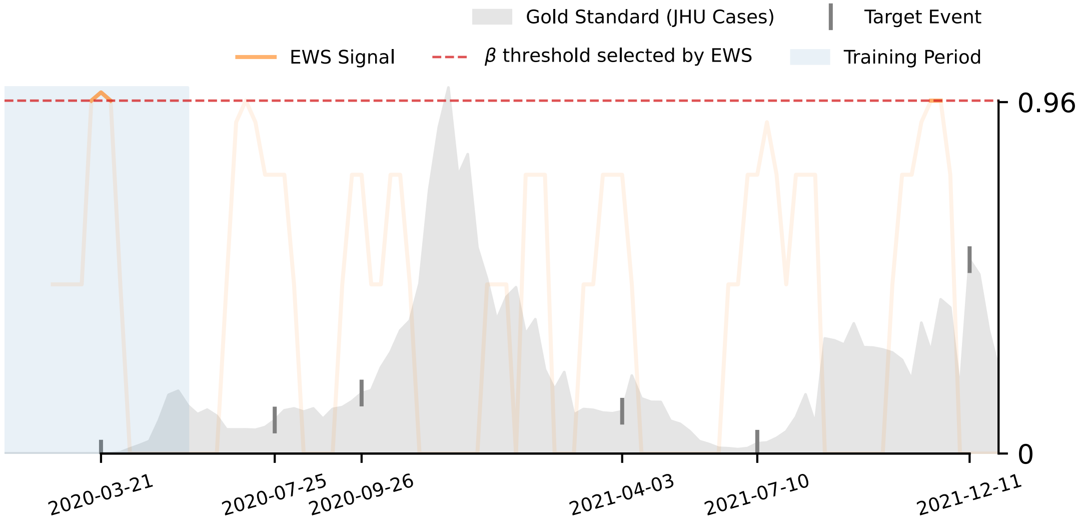

Performance as a function of β

β	Training tp	Training fp	Training fn	Out of sample tp	Out of sample fp	Out of sample fn
1.00	0	0	1	0	0	5
0.99	0	0	1	0	0	5
0.96	1	0	0	2	0	3
0.91	1	1	0	2	1	2
0.76	1	1	0	5	12	0
0.46	1	1	0	5	22	0
0.00	1	3	0	5	49	0

Model 2

(JHU) cases
(GT) covid
(GT) covid-19
(GT) chest pain
(GT) quarantine
(GT) fever

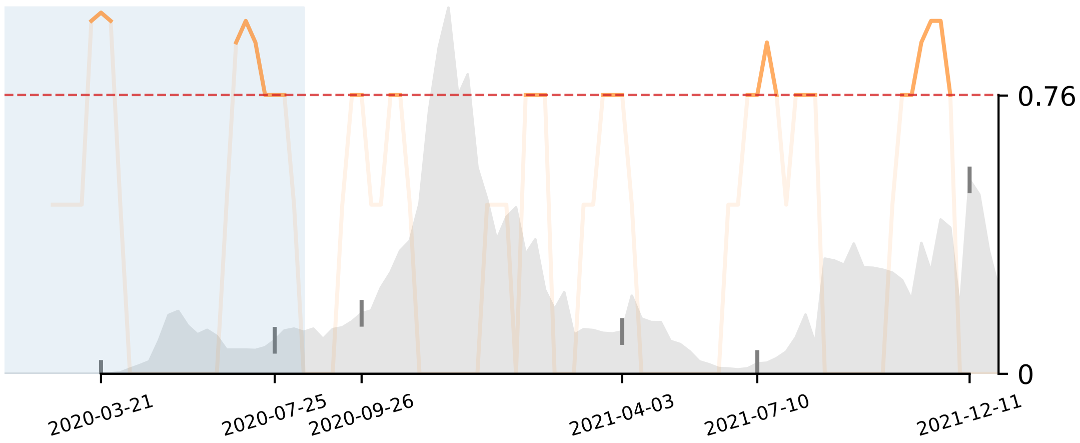

β	Training tp	Training fp	Training fn	Out of sample tp	Out of sample fp	Out of sample fn
1.00	0	0	2	0	0	4
0.99	0	0	2	0	0	4
0.96	1	0	1	1	0	3
0.91	2	1	0	1	1	2
0.76	2	1	0	4	11	0
0.46	2	2	0	4	20	0
0.00	2	6	0	4	45	0

Model 3

(GT) covid-19
(GT) fever
(GT) Asthma
(GT) Pneumonia
(JHU) cases
(GT) quarantine

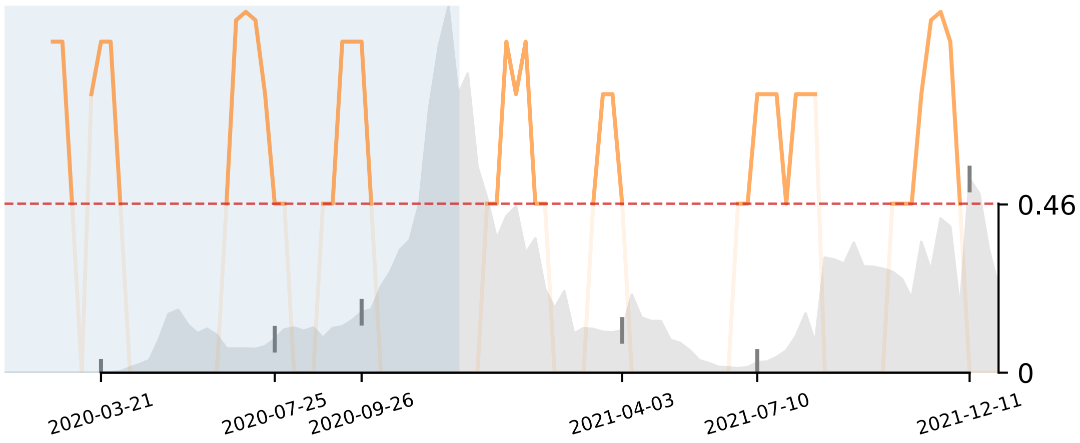

β	Training tp	Training fp	Training fn	Out of sample tp	Out of sample fp	Out of sample fn
1.00	0	0	3	0	0	3
0.99	0	0	3	1	0	2
0.96	1	0	2	1	0	2
0.91	1	0	2	1	2	2
0.76	3	2	0	3	8	0
0.46	3	2	0	3	15	0
0.00	3	6	0	3	35	0

Proxies selected by the Multiple Sources method

Model 1

(JHU) cases
(GT) covid
(GT) covid-19
(GT) chest pain
(GT) covid symptoms
(GT) covid-19 who

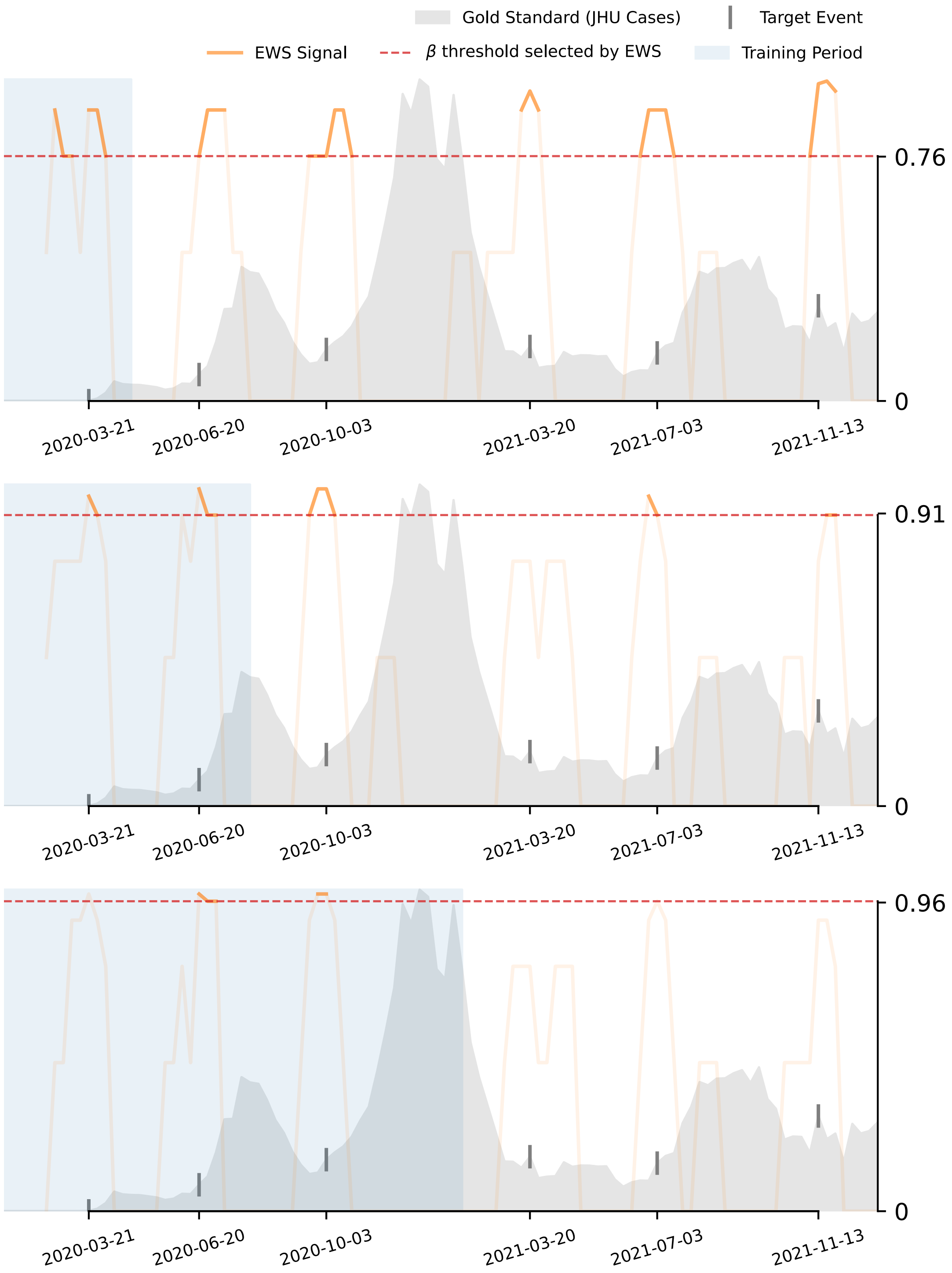

Performance as a function of β

Model 1

β	Training tp	Training fp	Training fn	Out of sample tp	Out of sample fp	Out of sample fn
1.00	0	0	1	0	1	4
0.99	0	0	1	1	1	4
0.96	0	0	1	2	2	3
0.91	0	0	1	3	9	0
0.76	1	1	0	5	11	0
0.46	1	2	0	5	22	0
0.00	1	3	0	5	53	0

Model 2

(JHU) cases
(GT) covid-19
(GT) fever
(GT) Anosmia
(GT) Chest pain
(GT) Chills

β	Training tp	Training fp	Training fn	Out of sample tp	Out of sample fp	Out of sample fn
1.00	0	0	2	0	0	4
0.99	0	0	2	1	0	3
0.96	1	0	1	2	0	2
0.91	2	0	0	2	3	1
0.76	2	3	0	4	7	0
0.46	2	4	0	4	17	0
0.00	2	5	0	4	46	0

Model 3

(JHU) cases
(GT) covid-19
(GT) fever
(GT) Anosmia
(GT) Chills
(GT) Phlegm

β	Training tp	Training fp	Training fn	Out of sample tp	Out of sample fp	Out of sample fn
1.00	0	0	3	0	0	3
0.99	0	0	3	0	0	3
0.96	3	0	0	1	0	2
0.91	3	2	0	2	2	1
0.76	3	4	0	3	6	0
0.46	3	5	0	3	12	0
0.00	3	6	0	3	28	0

New Hampshire

Proxies selected by the Multiple Sources method

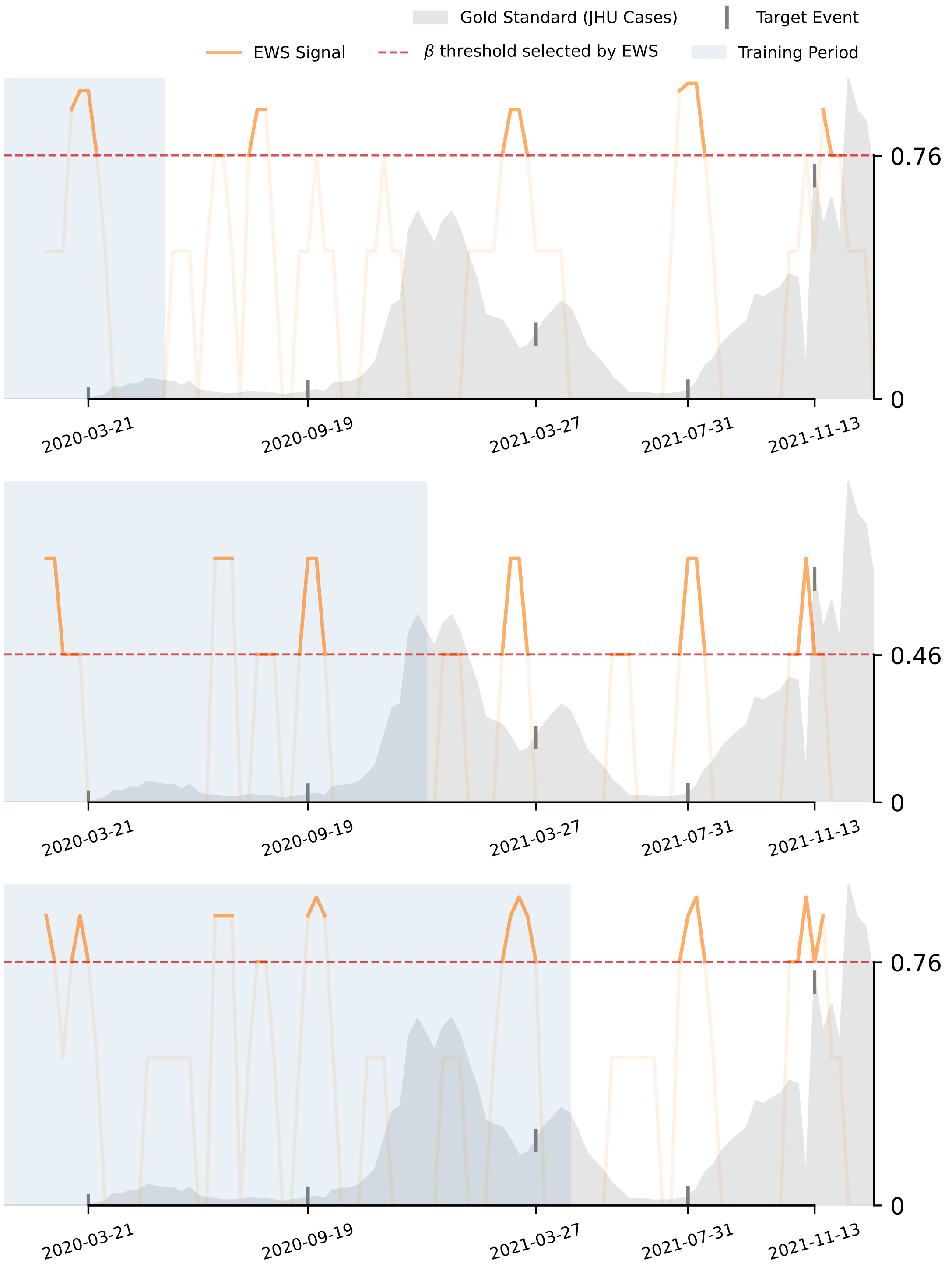

Performance as a function of β

Model 1

(JHU) cases
(GT) covid
(GT) covid-19
(GT) covid symptoms
(GT) quarantine
(GT) Acute bronchitis

	Training			Out of sample		
β	tp	fp	fn	tp	fp	fn
1.00	0	0	1	0	0	4
0.99	0	0	1	1	1	3
0.96	0	0	1	1	1	3
0.91	1	0	0	3	2	0
0.76	1	0	0	4	10	0
0.46	1	1	0	4	30	0
0.00	1	3	0	4	56	0

Model 2

(GT) Acute bronchitis
(GT) covid
(GT) Shortness of breath

β	tp	fp	fn	tp	fp	fn
0.91	0	0	2	0	0	3
0.76	0	0	2	3	1	0
0.46	2	5	0	3	9	0
0.00	2	8	0	3	32	0

Model 3

(GT) Acute bronchitis
(GT) covid
(GT) covid symptoms
(GT) quarantine
(GT) Shortness of breath
(GT) Fever

β	tp	fp	fn	tp	fp	fn
1.00	0	0	3	0	0	2
0.99	0	0	3	0	0	2
0.96	0	0	3	1	1	0
0.91	1	1	2	2	2	0
0.76	3	6	0	2	3	0
0.46	3	8	0	2	9	0
0.00	3	24	0	2	22	0

New Jersey

Proxies selected by the Multiple Sources method

Model 1

(JHU) cases
(GT) covid
(GT) covid-19
(GT) covid symptoms
(GT) chest tightness
(GT) fever

Model 2

(GT) chest tightness
(GT) covid symptoms
(GT) Ageusia
(GT) Anosmia
(GT) Asthma
(GT) Burning Chest Pain

Model 3

(GT) chest tightness
(GT) covid symptoms
(GT) nose bleed
(GT) Acute bronchitis
(GT) Ageusia
(GT) Anosmia

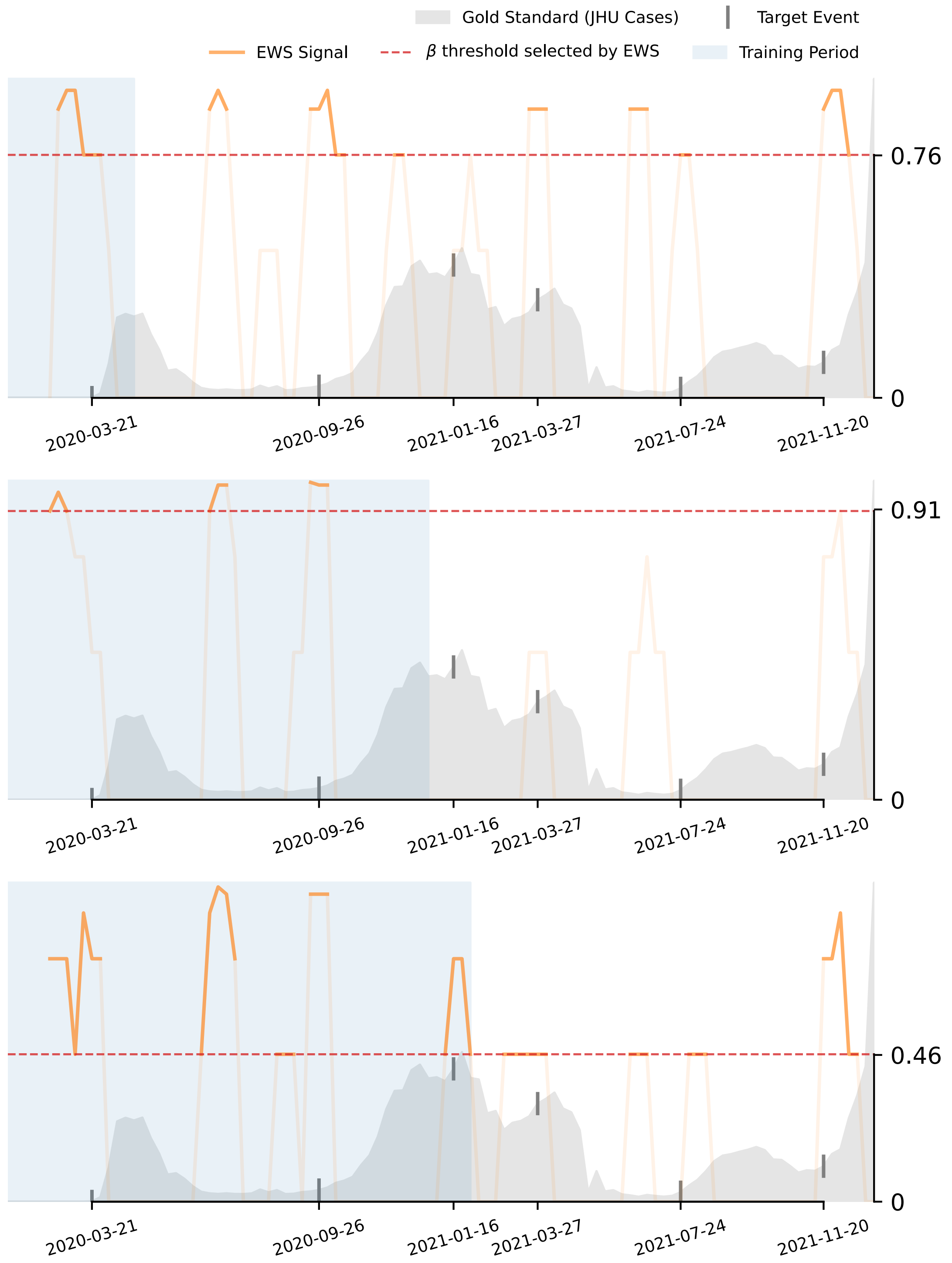

Performance as a function of β

Model 1

β	Training tp	Training fp	Training fn	Out of sample tp	Out of sample fp	Out of sample fn
1.00	0	0	1	0	0	5
0.99	0	0	1	0	0	5
0.96	0	0	1	0	4	3
0.91	1	0	0	4	7	1
0.76	1	0	0	5	13	0
0.46	1	1	0	5	21	0
0.00	1	2	0	5	53	0

Model 2

β	Training tp	Training fp	Training fn	Out of sample tp	Out of sample fp	Out of sample fn
1.00	0	0	2	0	0	4
0.99	1	0	1	0	0	4
0.96	1	3	1	0	0	4
0.91	2	3	0	0	1	3
0.76	2	5	0	2	2	2
0.46	2	6	0	3	5	1
0.00	2	7	0	4	29	0

Model 3

β	Training tp	Training fp	Training fn	Out of sample tp	Out of sample fp	Out of sample fn
1.00	0	0	3	0	0	3
0.99	0	0	3	0	0	3
0.96	0	1	3	0	0	3
0.91	1	3	2	0	1	2
0.76	2	4	1	1	2	2
0.46	3	8	0	3	8	0
0.00	3	11	0	3	27	0

New Mexico

Proxies selected by the Multiple Sources method

Model 1

(GT) covid
(GT) covid-19
(GT) chest pain
(GT) quarantine
(GT) sars-cov-2
(GT) Acute bronchitis

Model 2

(GT) Otitis
(GT) covid symptoms
(GT) Chest pain
(GT) Fever

Model 3

(GT) Otitis
(GT) covid symptoms
(GT) Fever
(GT) chest pain
(GT) Asphyxia
(GT) Vomiting

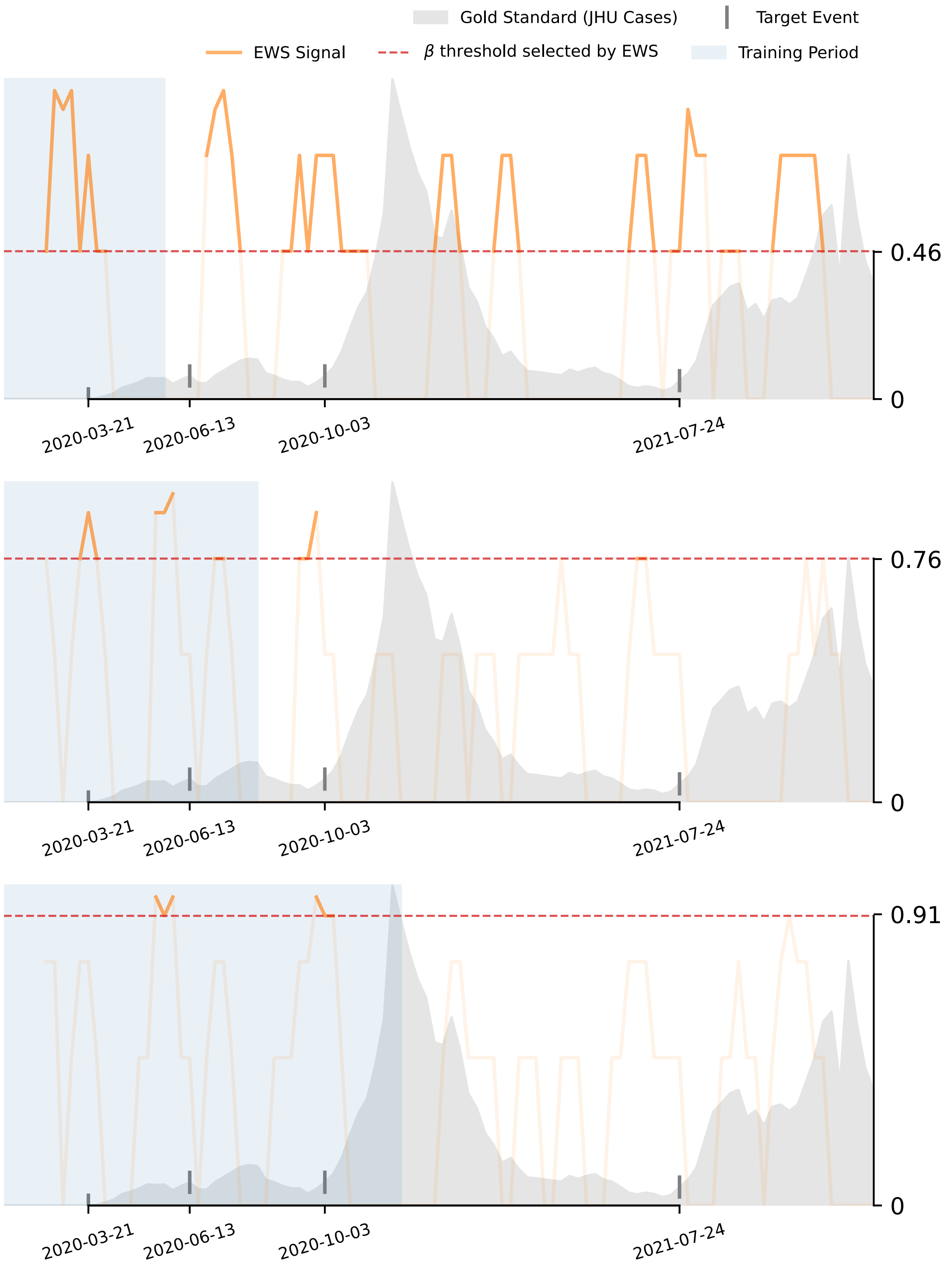

Performance as a function of β

Model 1

β	Training tp	Training fp	Training fn	Out of sample tp	Out of sample fp	Out of sample fn
1.00	0	0	1	0	0	3
0.99	0	0	1	0	0	3
0.96	0	0	1	0	1	3
0.91	1	0	0	0	3	2
0.76	1	0	0	2	17	0
0.46	1	0	0	2	31	0
0.00	1	3	0	3	67	0

Model 2

β	Training tp	Training fp	Training fn	Out of sample tp	Out of sample fp	Out of sample fn
0.96	0	0	2	0	0	2
0.91	1	0	1	1	0	1
0.76	2	0	0	2	3	0
0.46	2	4	0	2	25	0
0.00	2	7	0	2	59	0

Model 3

β	Training tp	Training fp	Training fn	Out of sample tp	Out of sample fp	Out of sample fn
1.00	0	0	3	0	0	1
0.99	0	0	3	0	0	1
0.96	0	0	3	0	0	1
0.91	2	0	1	0	1	1
0.76	2	1	1	1	7	0
0.46	3	4	0	1	27	0
0.00	3	12	0	1	49	0

Proxies selected by the Multiple Sources method

Model 1

(GT) covid
(GT) how long does covid last
(GT) loss smell
(GT) covid-19
(GT) loss taste
(GT) chest pain

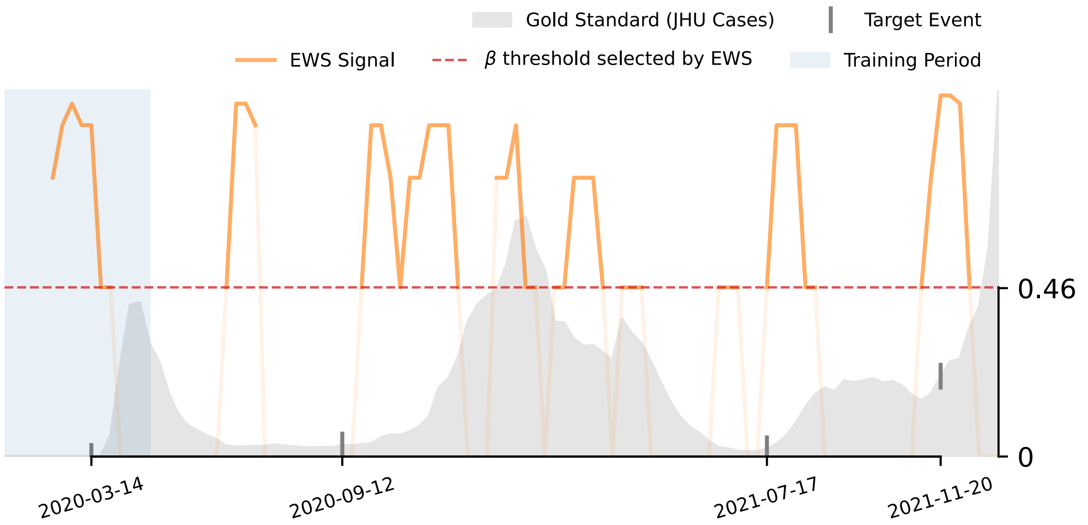

Performance as a function of β

β	Training tp	Training fp	Training fn	Out of sample tp	Out of sample fp	Out of sample fn
1.00	0	0	1	0	0	3
0.99	0	0	1	1	1	2
0.96	0	0	1	1	4	2
0.91	1	0	0	1	14	1
0.76	1	0	0	1	22	1
0.46	1	0	0	2	37	0
0.00	1	2	0	3	67	0

Model 2

(GT) nose bleed
(GT) sars-cov-2
(GT) Anosmia
(GT) Asthma
(GT) Burning Chest Pain
(GT) Pneumonia

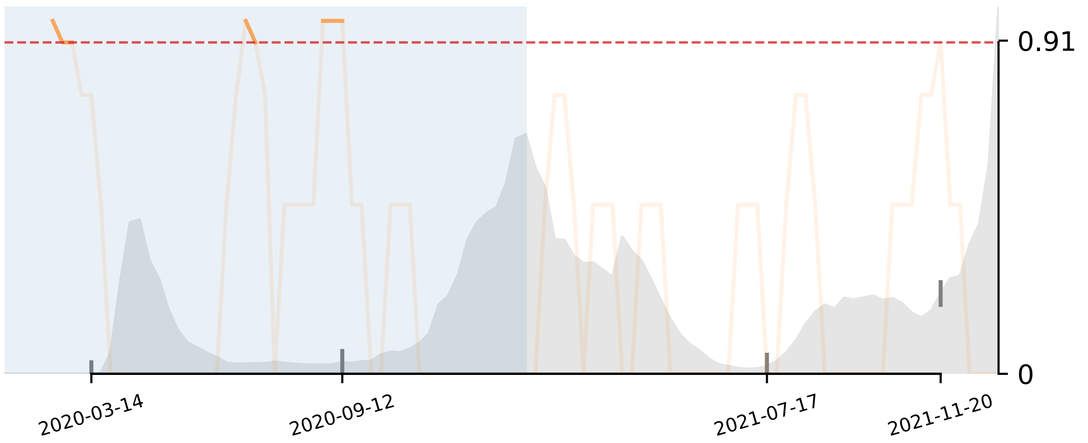

β	Training tp	Training fp	Training fn	Out of sample tp	Out of sample fp	Out of sample fn
1.00	0	0	2	0	0	2
0.99	0	0	2	0	0	2
0.96	0	0	2	0	0	2
0.91	2	1	0	1	0	1
0.76	2	2	0	1	4	1
0.46	2	4	0	2	16	0
0.00	2	13	0	2	35	0

Model 3

(GT) Asthma
(GT) covid symptoms
(GT) covid-19 who
(GT) Anosmia
(GT) Bronchitis
(GT) Burning Chest Pain

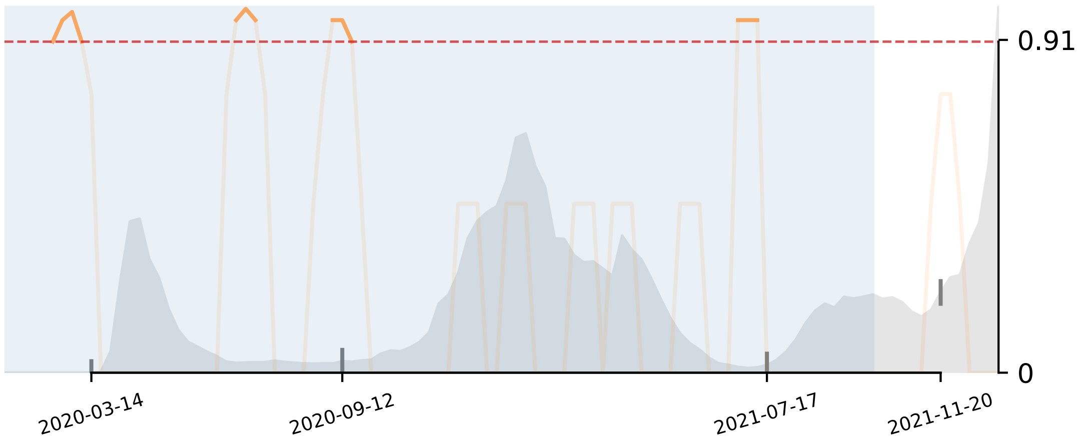

β	Training tp	Training fp	Training fn	Out of sample tp	Out of sample fp	Out of sample fn
1.00	0	0	3	0	0	1
0.99	0	1	3	0	0	1
0.96	1	1	2	0	0	1
0.91	3	3	0	0	0	1
0.76	3	4	0	1	1	0
0.46	3	6	0	1	2	0
0.00	3	22	0	1	6	0

North Carolina

Proxies selected by the Multiple Sources method

Model 1

(JHU) cases
(GT) sars-cov-2
(GT) covid
(GT) covid-19
(GT) chest pain
(GT) covid symptoms

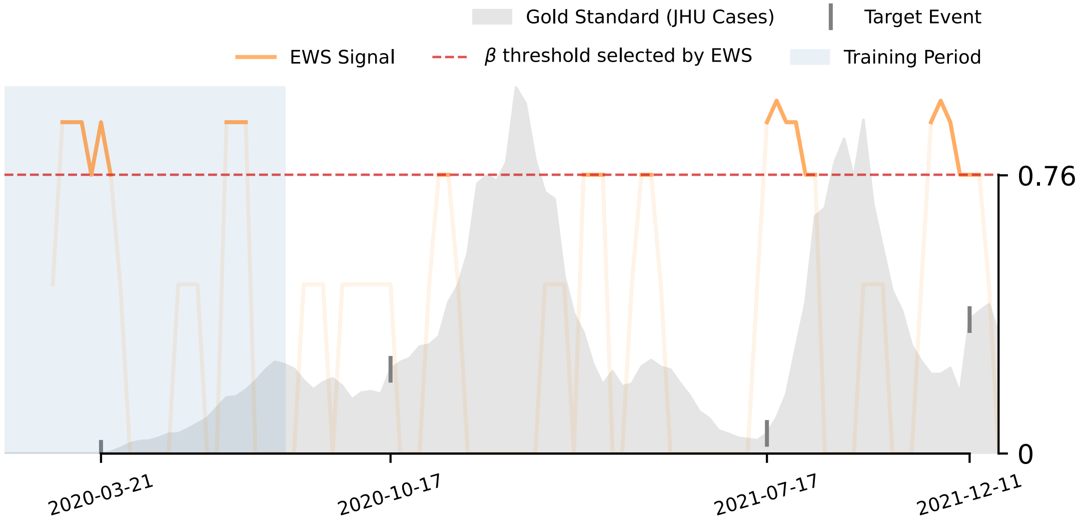

Performance as a function of β

β	Training tp	Training fp	Training fn	Out of sample tp	Out of sample fp	Out of sample fn
1.00	0	0	1	0	0	3
0.99	0	0	1	0	0	3
0.96	0	0	1	1	1	1
0.91	0	0	1	2	3	1
0.76	1	3	0	2	13	1
0.46	1	4	0	3	27	0
0.00	1	9	0	3	53	0

Model 2

(GT) chest pain
(GT) covid symptoms
(GT) Acute bronchitis
(GT) Ageusia
(GT) Anosmia
(GT) Bronchitis

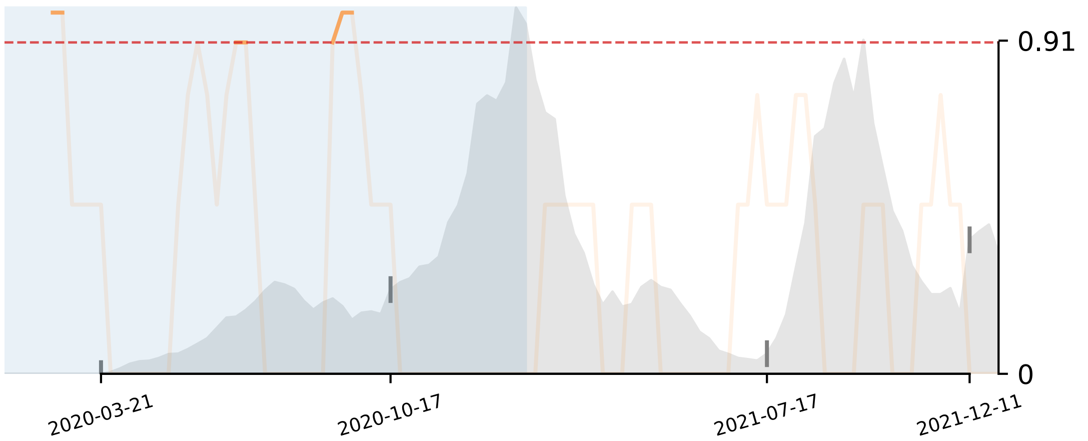

β	Training tp	Training fp	Training fn	Out of sample tp	Out of sample fp	Out of sample fn
1.00	0	0	2	0	0	2
0.99	0	0	2	0	0	2
0.96	2	2	0	0	0	2
0.91	2	2	0	0	0	2
0.76	2	6	0	2	2	0
0.46	2	9	0	2	17	0
0.00	2	12	0	2	35	0

Model 3

(GT) Bronchitis
(GT) Throat irritation
(GT) covid symptoms
(GT) Cough
(GT) Nasal congestion
(GT) Phlegm

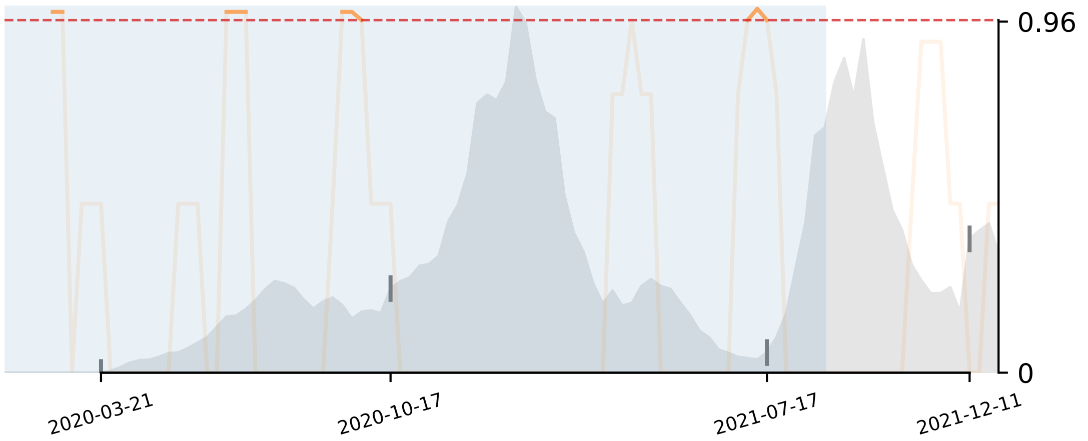

β	Training tp	Training fp	Training fn	Out of sample tp	Out of sample fp	Out of sample fn
1.00	0	0	3	0	0	1
0.99	1	0	2	0	0	1
0.96	3	5	0	0	0	1
0.91	3	6	0	1	0	0
0.76	3	6	0	1	0	0
0.46	3	11	0	1	2	0
0.00	3	15	0	1	11	0

North Dakota

Proxies selected by the Multiple Sources method

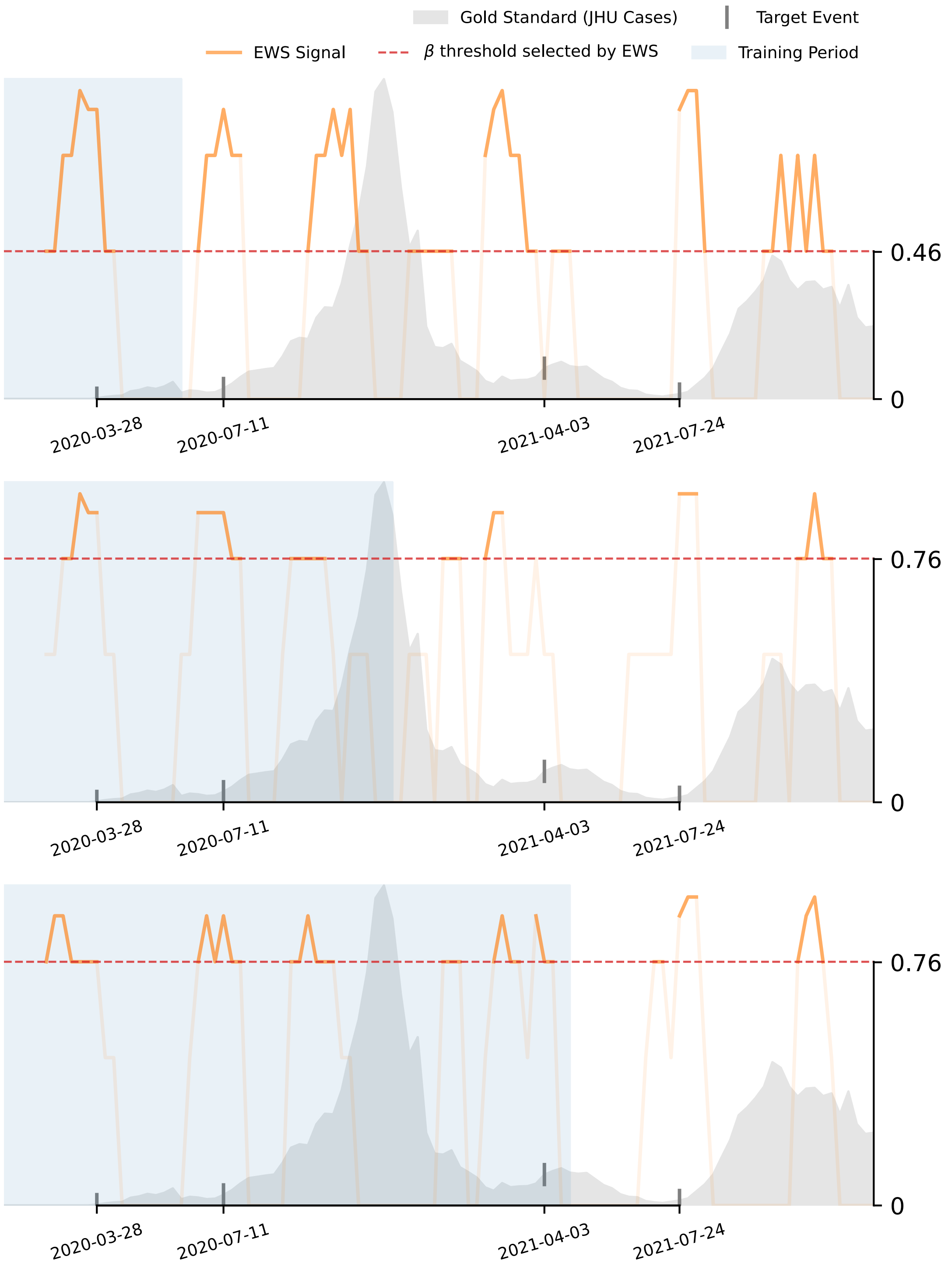

Performance as a function of β

Model 1

(JHU) cases
(GT) covid
(GT) covid-19
(GT) quarantine
(GT) Acute bronchitis
(GT) Chills

β	Training tp	Training fp	Training fn	Out of sample tp	Out of sample fp	Out of sample fn
1.00	0	0	1	0	0	3
0.99	0	0	1	0	0	3
0.96	0	0	1	1	2	1
0.91	1	0	0	3	4	0
0.76	1	0	0	3	13	0
0.46	1	0	0	3	32	0
0.00	1	4	0	3	63	0

Model 2

(JHU) cases
(GT) covid
(GT) covid-19
(GT) covid symptoms
(GT) quarantine
(GT) Cough

β	Training tp	Training fp	Training fn	Out of sample tp	Out of sample fp	Out of sample fn
1.00	0	0	2	0	0	2
0.99	0	0	2	0	0	2
0.96	0	0	2	1	3	1
0.91	1	0	1	2	3	0
0.76	2	0	0	2	11	0
0.46	2	7	0	2	18	0
0.00	2	17	0	2	43	0

Model 3

(JHU) cases
(GT) quarantine
(GT) Cough
(GT) Nasal congestion
(GT) Acute bronchitis
(GT) covid

β	Training tp	Training fp	Training fn	Out of sample tp	Out of sample fp	Out of sample fn
1.00	0	0	3	0	0	1
0.99	0	0	3	0	0	1
0.96	0	0	3	0	3	0
0.91	0	0	3	1	4	0
0.76	3	3	0	1	6	0
0.46	3	16	0	1	8	0
0.00	3	21	0	1	29	0

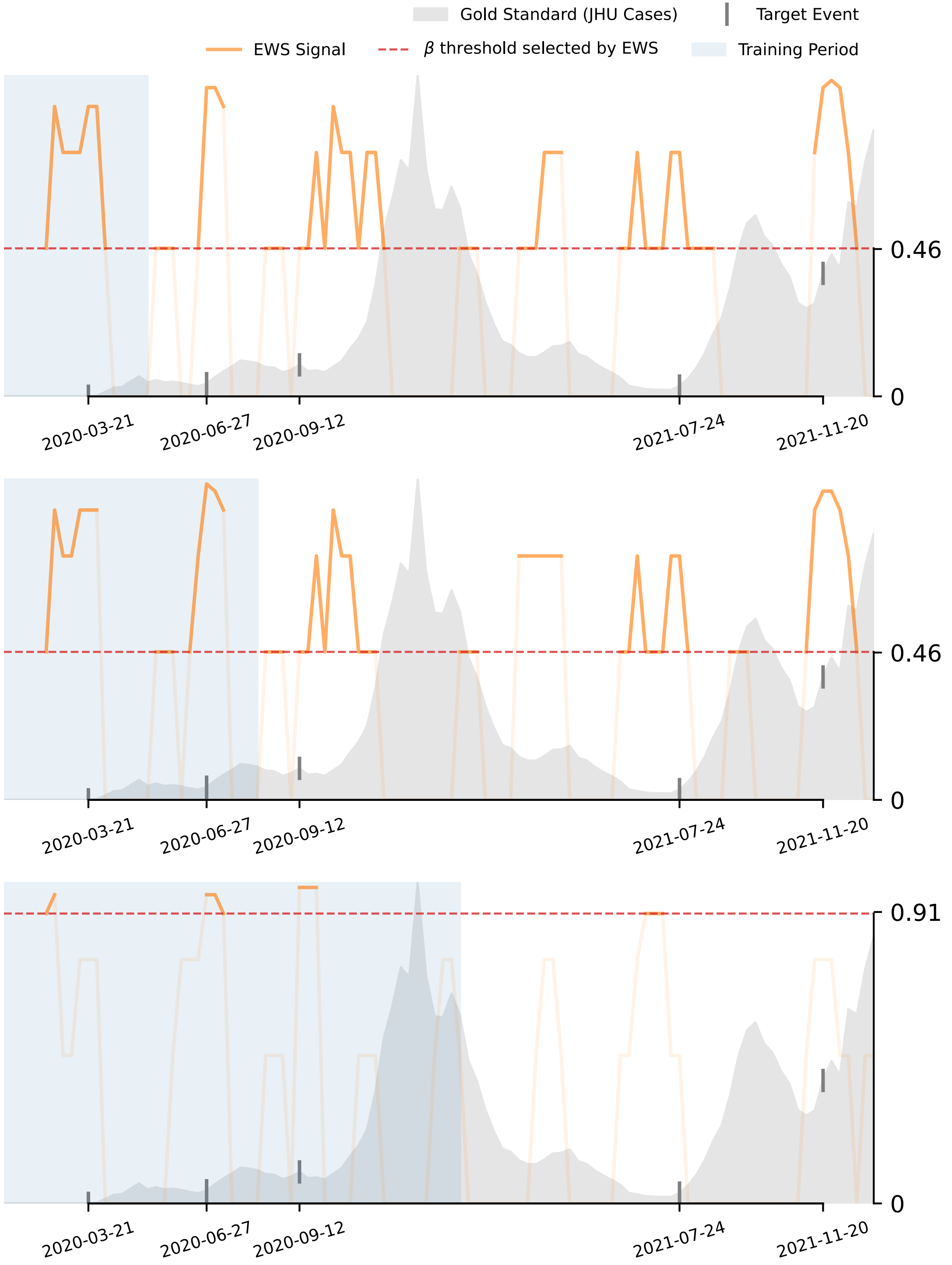

Proxies selected by the Multiple Sources method

Model 1

(GT) covid
(GT) how long does covid last
(GT) covid-19
(GT) chest pain
(GT) covid symptoms
(GT) chest tightness

Model 2

(GT) covid
(GT) covid-19
(GT) chest pain
(GT) covid symptoms
(GT) chest tightness
(GT) covid-19 who

Model 3

(GT) covid symptoms
(GT) Burning Chest Pain
(GT) Nasal congestion
(GT) chest tightness
(GT) Bronchitis
(GT) Throat irritation

Performance as a function of β

Model 1

β	Training tp	Training fp	Training fn	Out of sample tp	Out of sample fp	Out of sample fn
1.00	0	0	1	0	0	4
0.99	0	0	1	0	1	3
0.96	0	0	1	2	3	2
0.91	0	0	1	2	5	2
0.76	1	1	0	3	14	0
0.46	1	1	0	4	30	0
0.00	1	3	0	4	58	0

Model 2

β	Training tp	Training fp	Training fn	Out of sample tp	Out of sample fp	Out of sample fn
1.00	0	0	2	0	0	3
0.99	0	0	2	0	0	3
0.96	1	0	1	1	1	2
0.91	1	1	1	1	3	2
0.76	2	3	0	2	13	0
0.46	2	3	0	3	27	0
0.00	2	6	0	3	54	0

Model 3

β	Training tp	Training fp	Training fn	Out of sample tp	Out of sample fp	Out of sample fn
1.00	0	0	3	0	0	2
0.99	0	0	3	0	0	2
0.96	1	2	2	0	0	2
0.91	3	3	0	1	0	1
0.76	3	5	0	2	3	0
0.46	3	8	0	2	10	0
0.00	3	13	0	2	35	0

Oklahoma

Proxies selected by the Multiple Sources method

Performance as a function of β

Model 1

(JHU) cases
(GT) covid
(GT) how long does covid last
(GT) covid-19
(GT) chest pain
(GT) covid symptoms

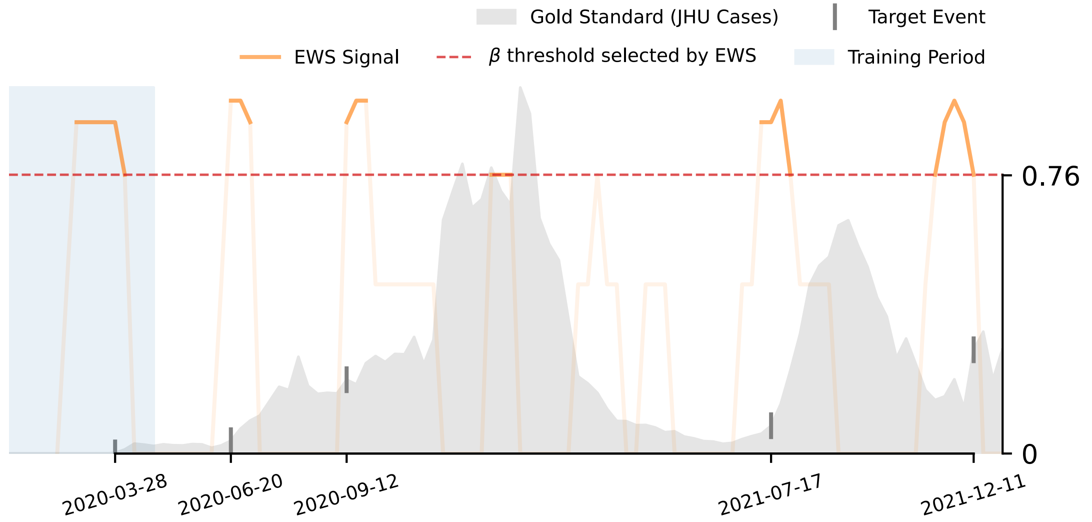

	Training			Out of sample		
β	tp	fp	fn	tp	fp	fn
1.00	0	0	1	0	0	4
0.99	0	0	1	0	0	4
0.96	0	0	1	2	4	0
0.91	0	0	1	4	5	0
0.76	1	0	0	4	10	0
0.46	1	1	0	4	28	0
0.00	1	2	0	4	60	0

Model 2

(GT) covid
(GT) covid-19
(GT) chest pain
(GT) covid symptoms
(GT) Acute bronchitis
(GT) Ageusia

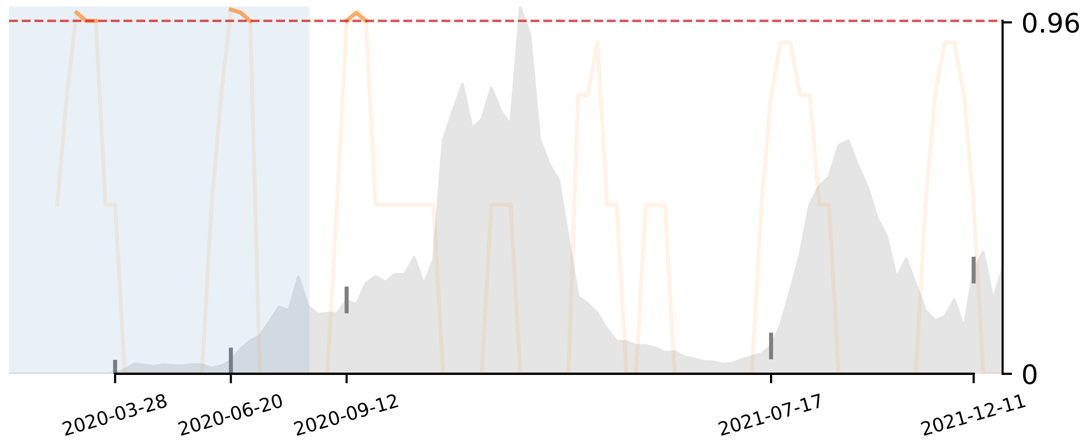

β	tp	fp	fn	tp	fp	fn
1.00	0	0	2	0	0	3
0.99	1	0	1	0	1	2
0.96	2	1	0	1	2	2
0.91	2	2	0	2	5	0
0.76	2	2	0	3	9	0
0.46	2	3	0	3	26	0
0.00	2	4	0	3	54	0

Model 3

(GT) Acute bronchitis
(GT) Ageusia
(GT) Anosmia
(GT) chest pain
(GT) covid symptoms
(GT) Chills

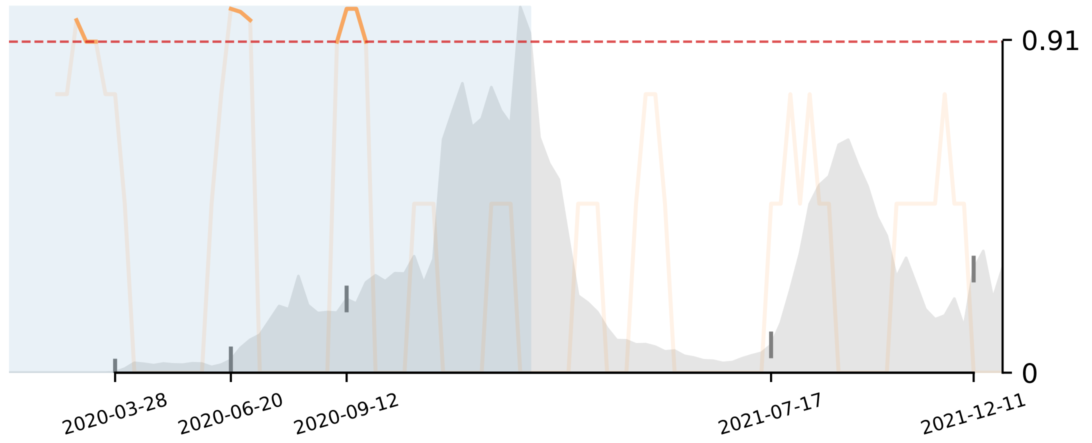

β	tp	fp	fn	tp	fp	fn
1.00	0	0	3	0	0	2
0.99	2	1	1	0	0	2
0.96	2	2	1	0	0	2
0.91	3	3	0	0	0	2
0.76	3	4	0	1	4	0
0.46	3	6	0	2	15	0
0.00	3	13	0	2	35	0

Proxies selected by the Multiple Sources method

Model 1

(JHU) cases
(GT) covid-19 who
(GT) covid
(GT) covid-19
(GT) chest pain
(GT) covid symptoms

Model 2

(JHU) cases
(GT) covid symptoms
(GT) quarantine
(GT) covid-19 who
(GT) fever
(GT) Anosmia

Model 3

(JHU) cases
(GT) covid symptoms
(GT) fever
(GT) Cough
(GT) Fever
(GT) Hyperventilation

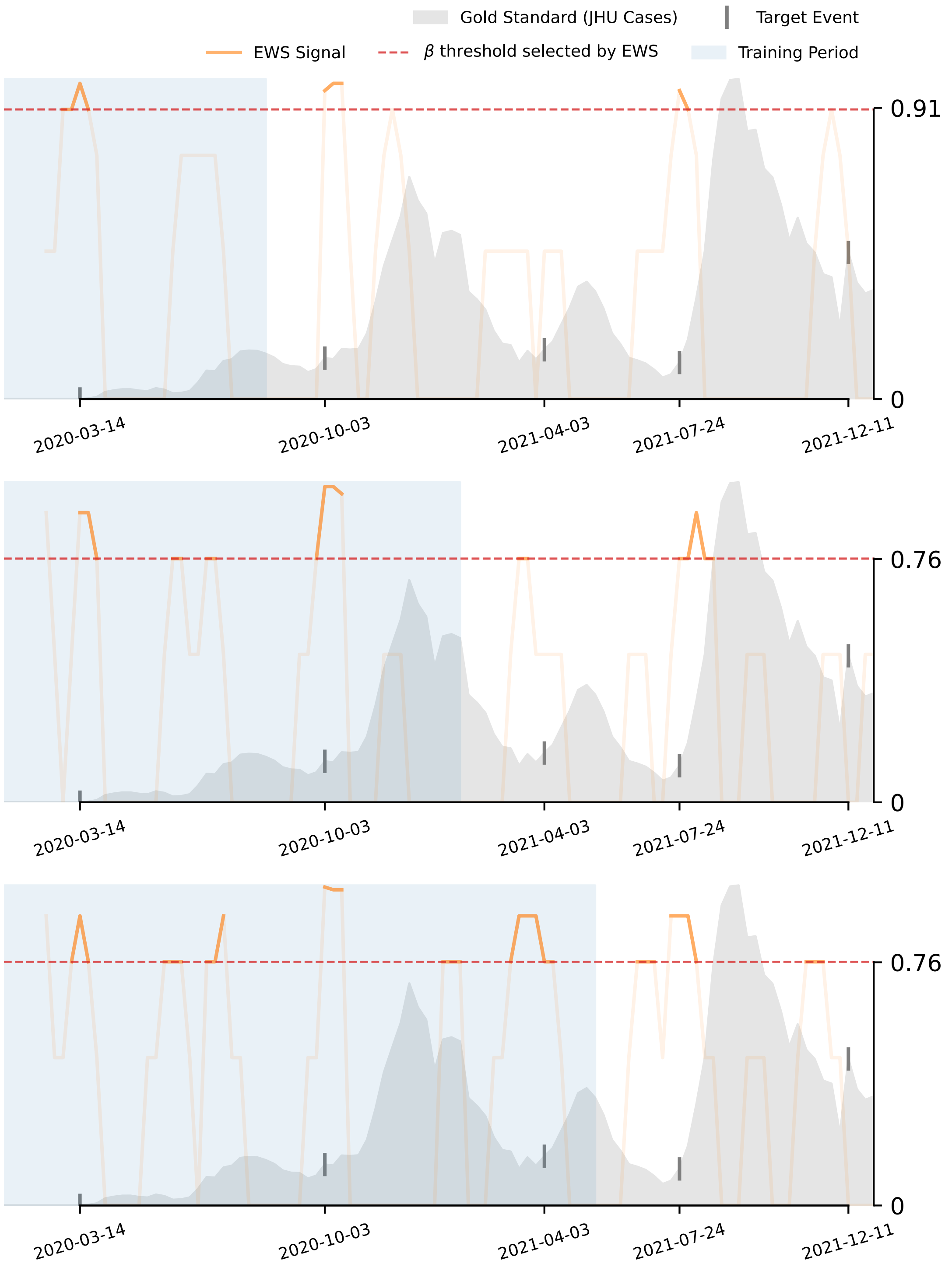

Performance as a function of β

Model 1

β	Training tp	Training fp	Training fn	Out of sample tp	Out of sample fp	Out of sample fn
1.00	0	0	1	0	0	4
0.99	0	0	1	0	2	3
0.96	1	0	0	2	2	2
0.91	1	0	0	3	4	1
0.76	1	1	0	3	7	1
0.46	1	7	0	4	13	0
0.00	1	9	0	4	44	0

Model 2

β	Training tp	Training fp	Training fn	Out of sample tp	Out of sample fp	Out of sample fn
1.00	0	0	2	0	0	3
0.99	0	0	2	0	0	3
0.96	1	1	1	0	0	3
0.91	1	2	1	0	1	2
0.76	2	3	0	2	4	1
0.46	2	8	0	3	11	0
0.00	2	15	0	3	28	0

Model 3

β	Training tp	Training fp	Training fn	Out of sample tp	Out of sample fp	Out of sample fn
1.00	0	0	3	0	0	2
0.99	1	0	2	0	0	2
0.96	1	2	2	0	0	2
0.91	1	2	2	1	1	1
0.76	3	3	0	2	2	0
0.46	3	13	0	2	7	0
0.00	3	22	0	2	19	0

Pennsylvania

Proxies selected by the Multiple Sources method

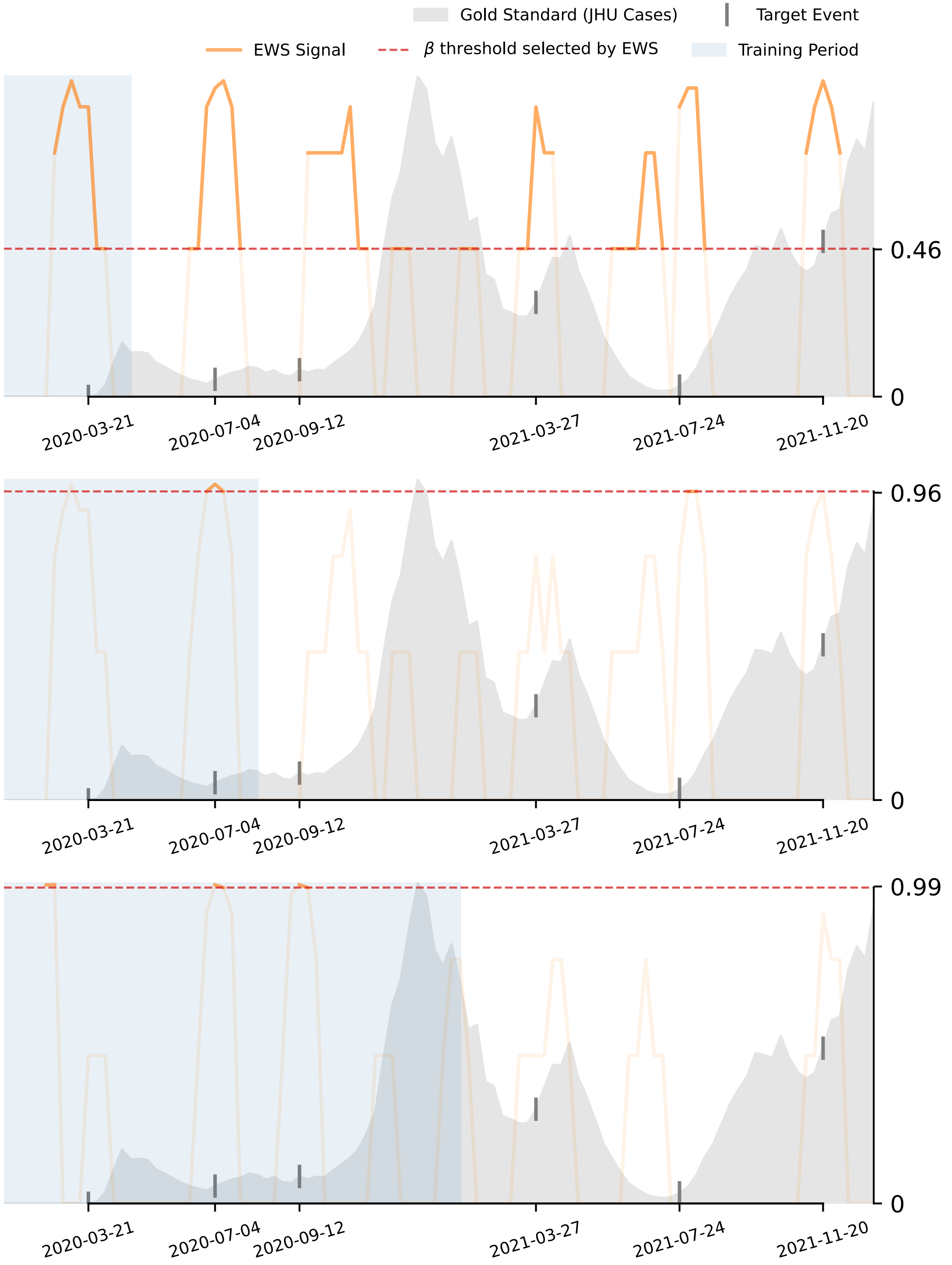

Performance as a function of β

Model 1

(JHU) cases
(GT) covid
(GT) covid-19
(GT) loss taste
(GT) chest pain
(GT) covid symptoms

β	Training tp	Training fp	Training fn	Out of sample tp	Out of sample fp	Out of sample fn
1.00	0	0	1	0	0	5
0.99	0	0	1	1	1	3
0.96	1	0	0	2	3	2
0.91	1	0	0	4	6	1
0.76	1	0	0	4	14	0
0.46	1	0	0	4	26	0
0.00	1	2	0	5	53	0

Model 2

(GT) covid
(GT) covid-19
(GT) loss taste
(GT) chest pain
(GT) covid symptoms
(GT) covid-19 who

β	Training tp	Training fp	Training fn	Out of sample tp	Out of sample fp	Out of sample fn
1.00	0	0	2	0	0	4
0.99	0	0	2	0	0	4
0.96	2	0	0	1	2	2
0.91	2	1	0	1	3	2
0.76	2	1	0	3	8	1
0.46	2	2	0	3	25	0
0.00	2	4	0	4	47	0

Model 3

(GT) Anosmia
(GT) Pneumonia
(GT) Viral pneumonia
(GT) Asthma
(GT) Chest pain
(GT) Wheeze

β	Training tp	Training fp	Training fn	Out of sample tp	Out of sample fp	Out of sample fn
1.00	0	0	3	0	0	3
0.99	3	1	0	0	0	3
0.96	3	3	0	0	0	3
0.91	3	3	0	1	0	2
0.76	3	4	0	2	4	0
0.46	3	7	0	3	7	0
0.00	3	13	0	3	28	0

Proxies selected by the Multiple Sources method

Model 1

(JHU) cases
(GT) covid
(GT) covid-19
(GT) chest pain
(GT) covid symptoms
(GT) quarantine

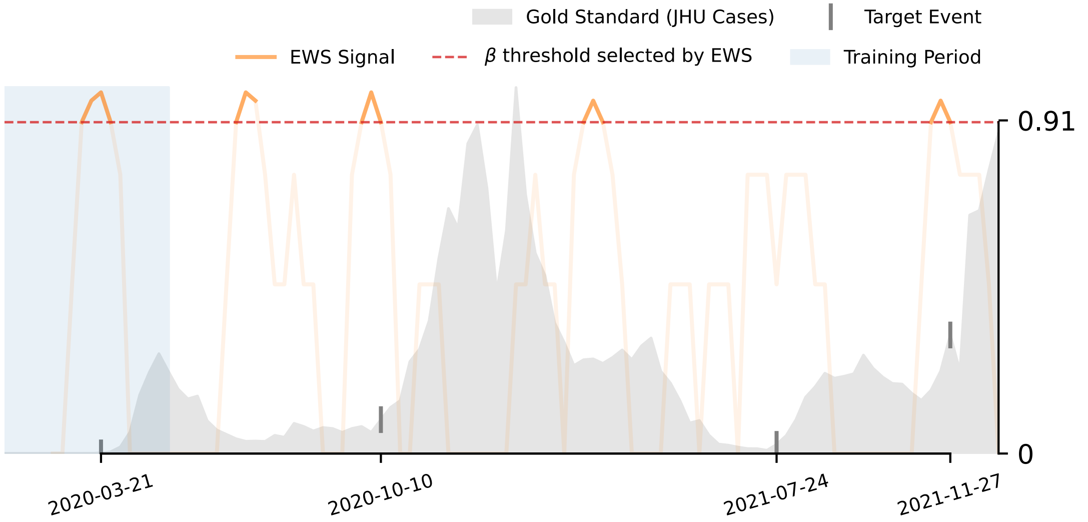

Performance as a function of β

β	Training tp	Training fp	Training fn	Out of sample tp	Out of sample fp	Out of sample fn
1.00	0	0	1	0	0	3
0.99	0	0	1	1	1	2
0.96	1	0	0	2	3	1
0.91	1	0	0	2	6	1
0.76	1	1	0	3	18	0
0.46	1	2	0	3	38	0
0.00	1	2	0	3	65	0

Model 2

(GT) Acute bronchitis
(JHU) cases
(GT) covid
(GT) covid-19
(GT) covid symptoms
(GT) quarantine

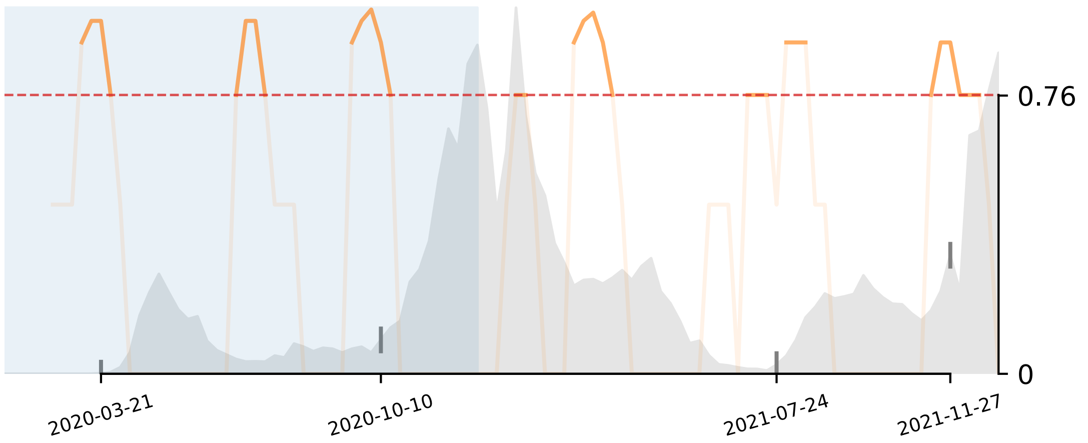

β	Training tp	Training fp	Training fn	Out of sample tp	Out of sample fp	Out of sample fn
1.00	0	0	2	0	0	2
0.99	1	0	1	0	1	2
0.96	1	0	1	0	2	2
0.91	2	2	0	1	7	0
0.76	2	2	0	2	13	0
0.46	2	6	0	2	20	0
0.00	2	11	0	2	40	0

Model 3

(GT) Throat irritation
(JHU) cases
(GT) covid
(GT) Asthma
(GT) Sore throat
(GT) covid symptoms

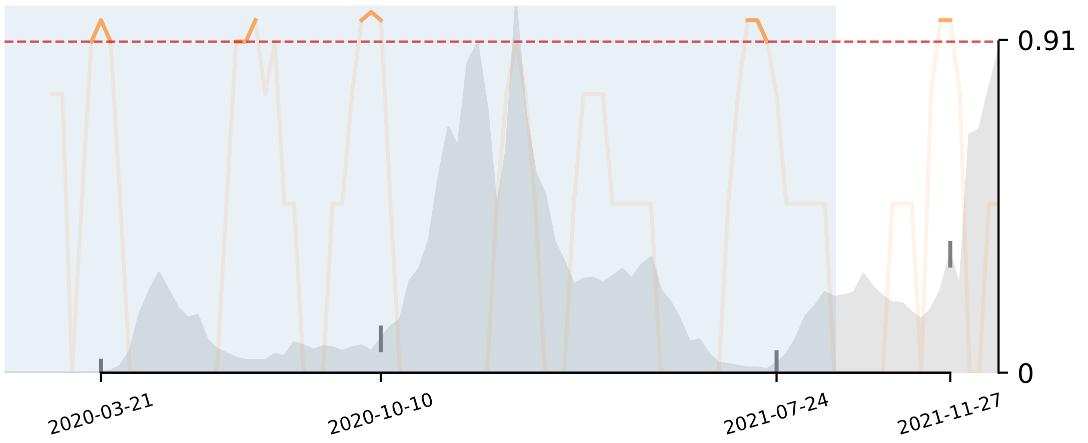

β	Training tp	Training fp	Training fn	Out of sample tp	Out of sample fp	Out of sample fn
1.00	0	0	3	0	0	1
0.99	0	0	3	0	0	1
0.96	1	0	2	1	0	0
0.91	3	1	0	1	0	0
0.76	3	6	0	1	1	0
0.46	3	13	0	1	3	0
0.00	3	33	0	1	10	0

South Carolina

Proxies selected by the Multiple Sources method

Model 1

(JHU) cases
(GT) covid
(GT) covid-19
(GT) chest pain
(GT) covid symptoms
(GT) quarantine

Model 2

(GT) chest pain
(GT) Ageusia
(GT) Anosmia
(GT) Chest pain
(GT) Otitis

Model 3

(GT) chest pain
(GT) Anosmia
(GT) Chest pain
(JHU) cases
(GT) Ageusia
(GT) Otitis

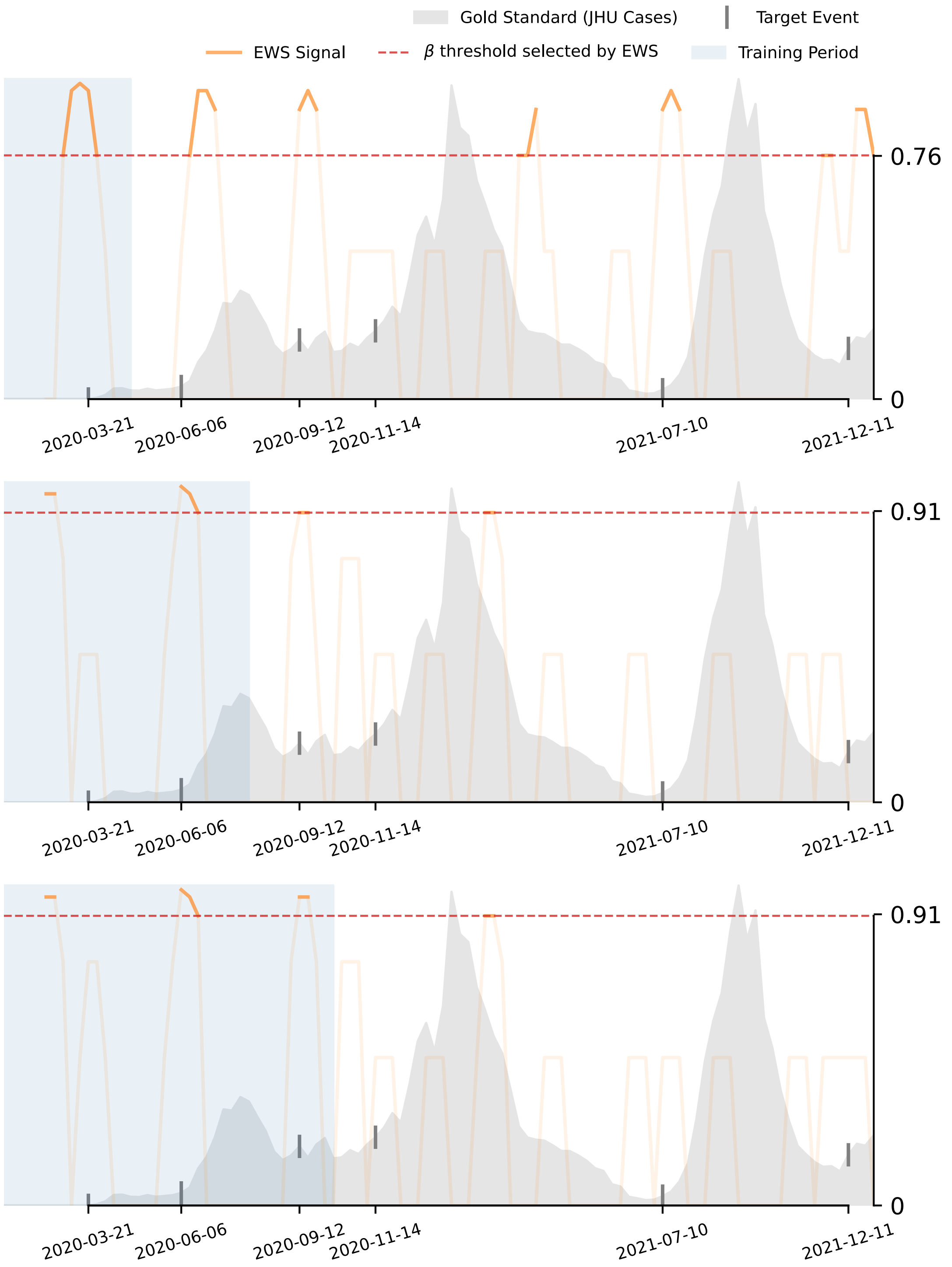

Performance as a function of β

Model 1

β	Training tp	Training fp	Training fn	Out of sample tp	Out of sample fp	Out of sample fn
1.00	0	0	1	0	0	5
0.99	0	0	1	0	0	5
0.96	1	0	0	0	4	2
0.91	1	0	0	2	10	1
0.76	1	0	0	3	14	1
0.46	1	1	0	5	29	0
0.00	1	2	0	5	54	0

Model 2

β	Training tp	Training fp	Training fn	Out of sample tp	Out of sample fp	Out of sample fn
0.99	0	0	2	0	0	4
0.96	1	0	1	0	0	4
0.91	2	2	0	1	3	3
0.76	2	3	0	2	4	2
0.46	2	3	0	4	18	0
0.00	2	4	0	4	47	0

Model 3

β	Training tp	Training fp	Training fn	Out of sample tp	Out of sample fp	Out of sample fn
1.00	0	0	3	0	0	3
0.99	0	0	3	0	0	3
0.96	1	0	2	0	0	3
0.91	3	3	0	0	2	3
0.76	3	4	0	1	3	2
0.46	3	6	0	3	20	0
0.00	3	7	0	3	45	0

Proxies selected by the Multiple Sources method

Model 1

(GT) covid
(GT) covid symptoms
(GT) quarantine
(GT) Acute bronchitis
(GT) Asthma
(GT) Bronchitis

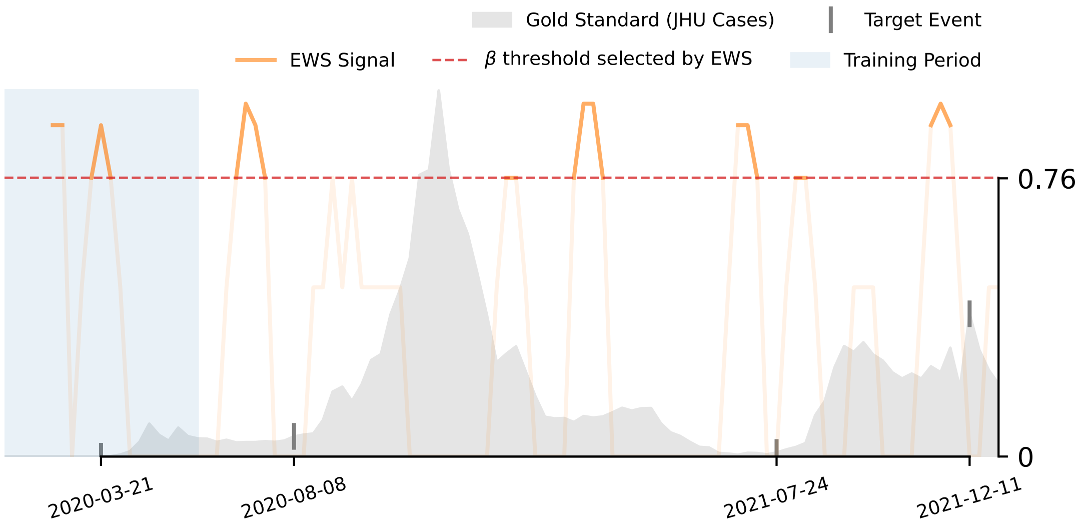

Performance as a function of β

	Training			Out of sample		
β	tp	fp	fn	tp	fp	fn
1.00	0	0	1	0	0	3
0.99	0	0	1	0	0	3
0.96	0	0	1	2	2	1
0.91	0	0	1	3	2	0
0.76	1	1	0	3	10	0
0.46	1	2	0	3	28	0
0.00	1	3	0	3	62	0

Model 2

(GT) covid
(GT) covid symptoms
(GT) Bronchitis
(GT) Cardiac arrest
(GT) Wheeze
Twitter (state level signal)

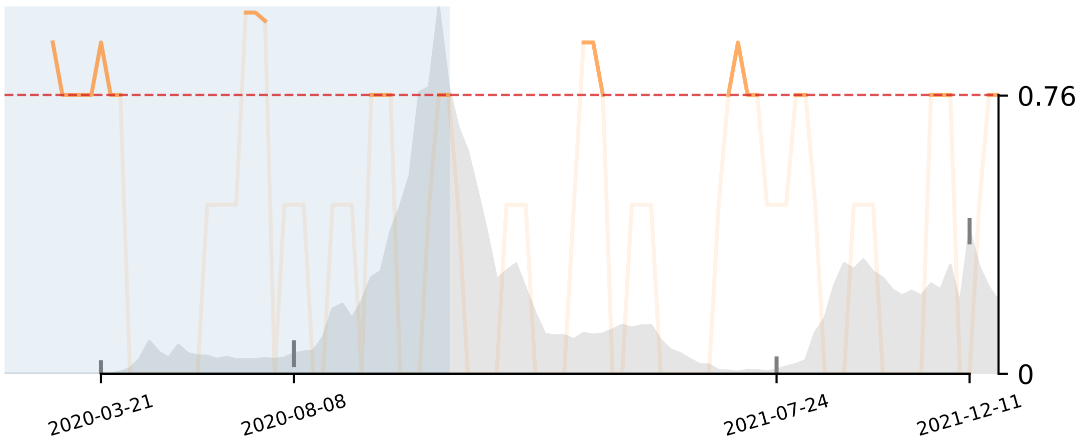

β	tp	fp	fn	tp	fp	fn
1.00	0	0	2	0	0	2
0.99	0	0	2	0	0	2
0.96	1	1	1	0	0	2
0.91	1	1	1	1	2	1
0.76	2	2	0	2	7	0
0.46	2	9	0	2	21	0
0.00	2	18	0	2	43	0

Model 3

(GT) Bronchitis
(GT) covid
(GT) Cardiac arrest
Twitter (state level signal)
(GT) covid symptoms
(GT) covid-19

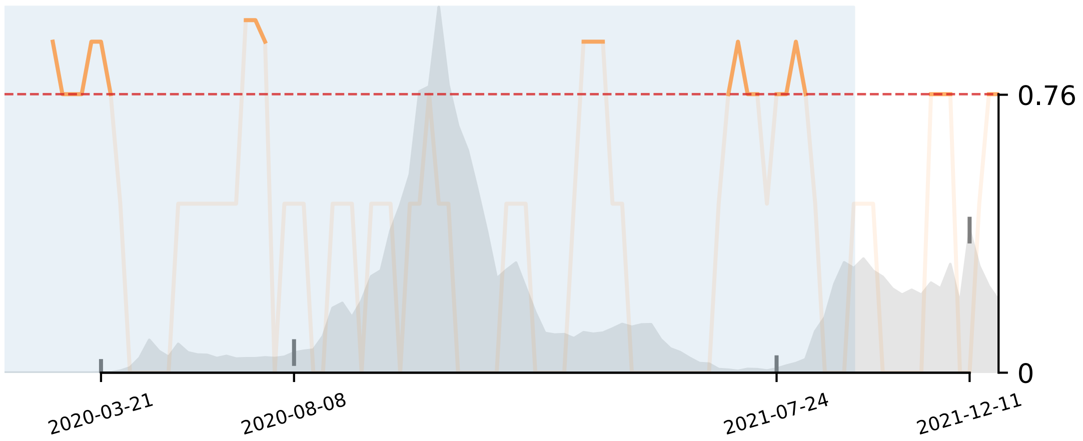

β	tp	fp	fn	tp	fp	fn
1.00	0	0	3	0	0	1
0.99	0	0	3	0	0	1
0.96	0	0	3	0	0	1
0.91	1	1	2	0	0	1
0.76	3	6	0	1	2	0
0.46	3	11	0	1	5	0
0.00	3	39	0	1	8	0

Tennessee

Proxies selected by the Multiple Sources method

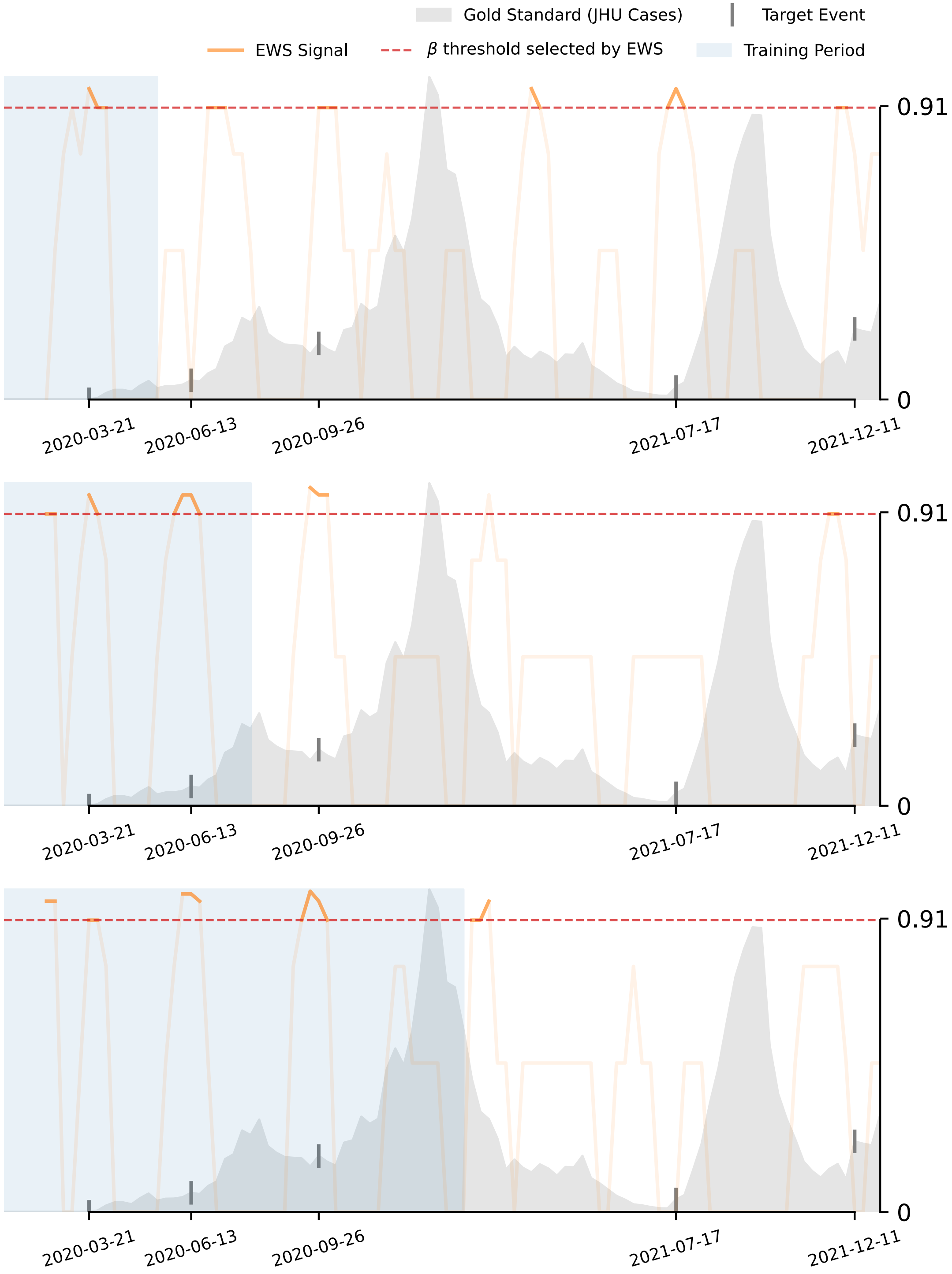

Model 1

(JHU) cases
(GT) covid
(GT) how long does covid last
(GT) covid-19
(GT) chest tightness
(GT) covid-19 who

Model 2

(GT) covid symptoms
(GT) fever
(GT) Ageusia
(GT) Anosmia
(GT) Chest pain
(GT) Diarrhea

Model 3

(GT) Ageusia
(GT) Anosmia
(GT) Chest pain
(GT) fever
(GT) Diarrhea
(GT) Shortness of breath

Performance as a function of β

Model 1

β	Training tp	Training fp	Training fn	Out of sample tp	Out of sample fp	Out of sample fn
1.00	0	0	1	0	0	4
0.99	0	0	1	0	0	4
0.96	0	0	1	1	1	3
0.91	1	0	0	3	8	0
0.76	1	2	0	3	16	0
0.46	1	2	0	4	36	0
0.00	1	2	0	4	60	0

Model 2

β	Training tp	Training fp	Training fn	Out of sample tp	Out of sample fp	Out of sample fn
1.00	0	0	2	0	0	3
0.99	0	0	2	1	0	2
0.96	0	0	2	1	2	2
0.91	2	0	0	2	2	1
0.76	2	3	0	2	6	1
0.46	2	4	0	3	28	0
0.00	2	5	0	3	53	0

Model 3

β	Training tp	Training fp	Training fn	Out of sample tp	Out of sample fp	Out of sample fn
1.00	0	0	3	0	0	2
0.99	1	0	2	0	0	2
0.96	2	0	1	0	1	2
0.91	3	2	0	0	3	2
0.76	3	4	0	2	3	0
0.46	3	7	0	2	21	0
0.00	3	13	0	2	35	0

Texas

Proxies selected by the Multiple Sources method

Model 1

(GT) side effects of vaccine
(GT) covid
(GT) how long does covid last
(GT) covid-19
(GT) loss taste
(GT) chest pain

Model 2

(GT) covid
(GT) loss taste
(GT) chest pain
(GT) covid symptoms
(GT) fever
(GT) Acute bronchitis

Model 3

(GT) loss taste
(GT) Fever
(GT) Shortness of breath
(GT) Vomiting
(GT) chest pain
(GT) Sore throat

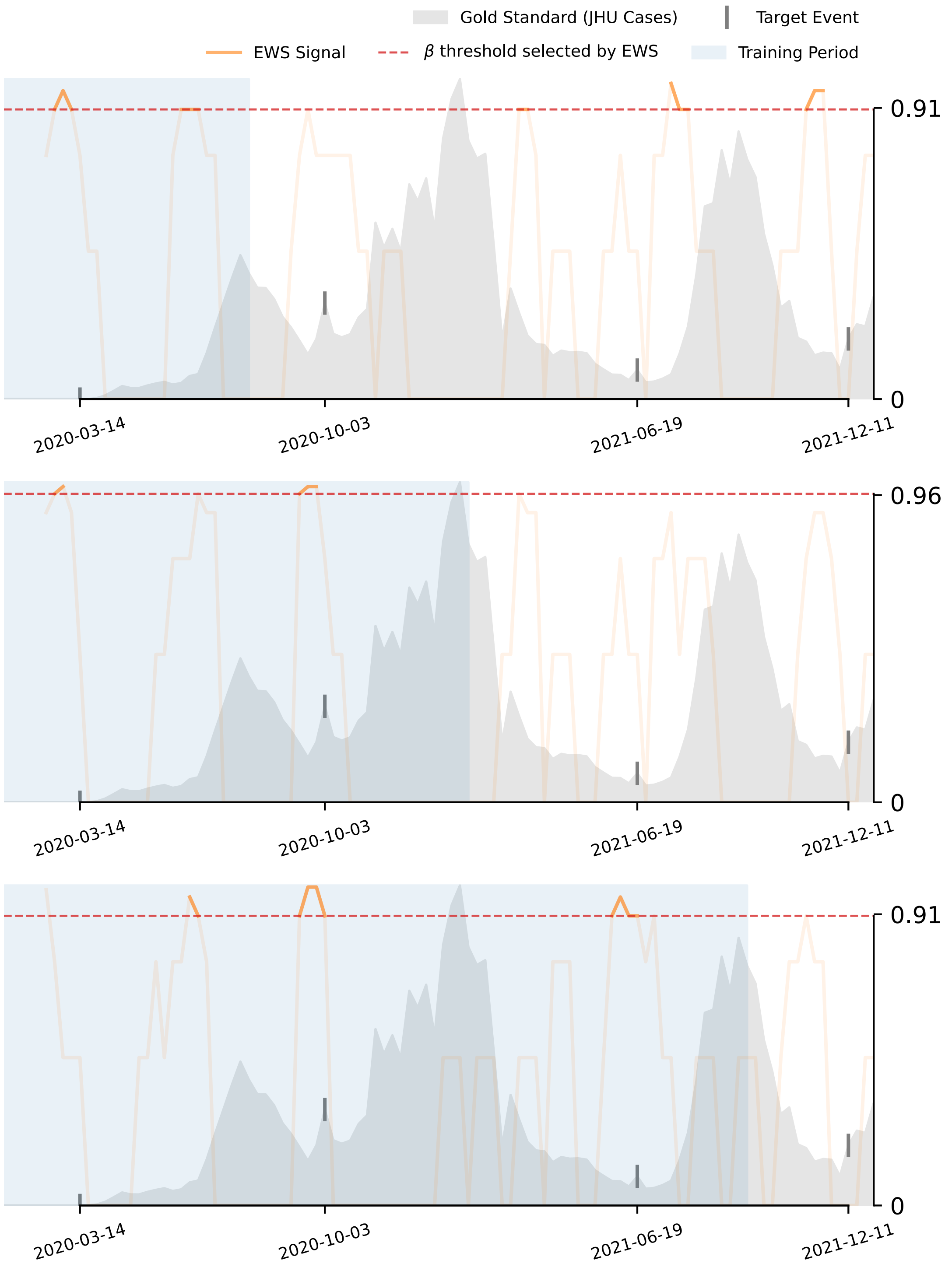

Performance as a function of β

Model 1

β	Training tp	Training fp	Training fn	Out of sample tp	Out of sample fp	Out of sample fn
1.00	0	0	1	0	0	3
0.99	0	0	1	0	1	3
0.96	0	0	1	1	1	2
0.91	1	0	0	2	5	1
0.76	1	3	0	3	13	0
0.46	1	6	0	3	28	0
0.00	1	8	0	3	53	0

Model 2

β	Training tp	Training fp	Training fn	Out of sample tp	Out of sample fp	Out of sample fn
1.00	0	0	2	0	0	2
0.99	0	0	2	0	0	2
0.96	2	0	0	0	1	2
0.91	2	1	0	1	4	1
0.76	2	3	0	2	9	0
0.46	2	6	0	2	18	0
0.00	2	10	0	2	34	0

Model 3

β	Training tp	Training fp	Training fn	Out of sample tp	Out of sample fp	Out of sample fn
1.00	0	0	3	0	0	1
0.99	1	0	2	0	0	1
0.96	2	0	1	0	0	1
0.91	3	1	0	1	0	0
0.76	3	3	0	1	1	0
0.46	3	11	0	1	5	0
0.00	3	30	0	1	8	0

Proxies selected by the Multiple Sources method

Model 1

(JHU) cases
(GT) covid
(GT) covid-19
(GT) chest pain
(GT) covid-19 who
(GT) Acute bronchitis

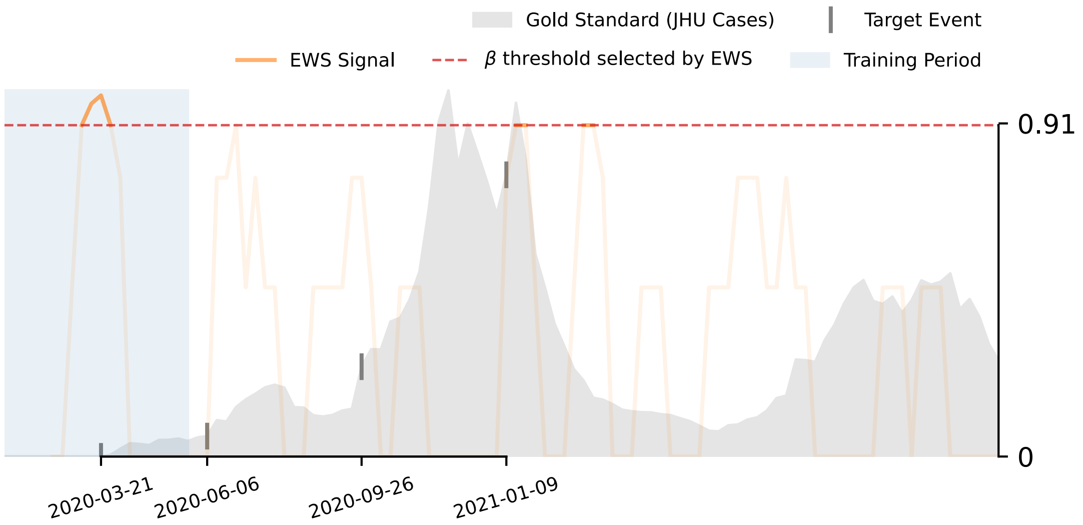

Performance as a function of β

	Training			Out of sample		
β	tp	fp	fn	tp	fp	fn
1.00	0	0	1	0	0	3
0.99	0	0	1	0	0	3
0.96	1	0	0	0	0	3
0.91	1	0	0	0	5	2
0.76	1	1	0	2	13	0
0.46	1	2	0	2	38	0
0.00	1	2	0	3	68	0

Model 2

(GT) covid symptoms
(GT) fever
(GT) Chest pain
(GT) Throat irritation
(GT) Chills

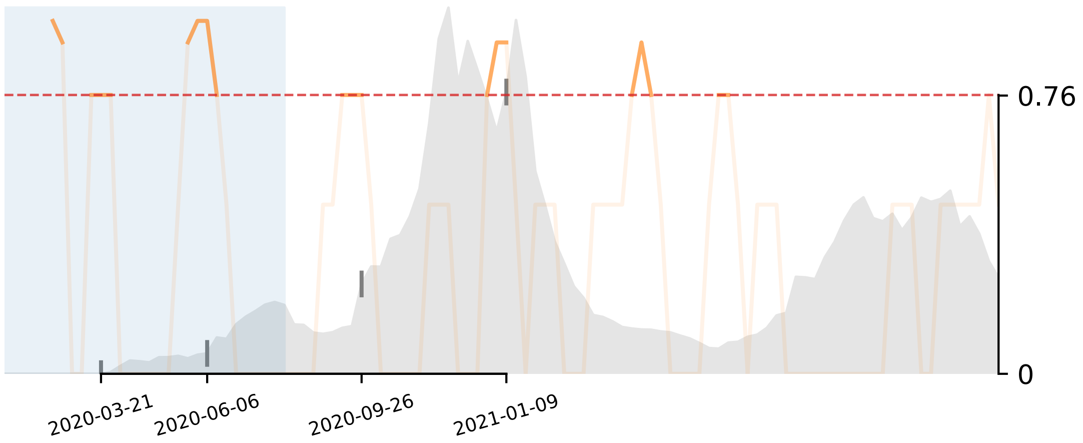

β	tp	fp	fn	tp	fp	fn
0.99	0	0	2	0	0	2
0.96	0	0	2	0	0	2
0.91	1	1	1	1	1	1
0.76	2	1	0	2	6	0
0.46	2	3	0	2	32	0
0.00	2	4	0	2	60	0

Model 3

(GT) fever
(GT) Chest pain
(GT) Chills
(JHU) cases
(GT) covid symptoms
(GT) Ageusia

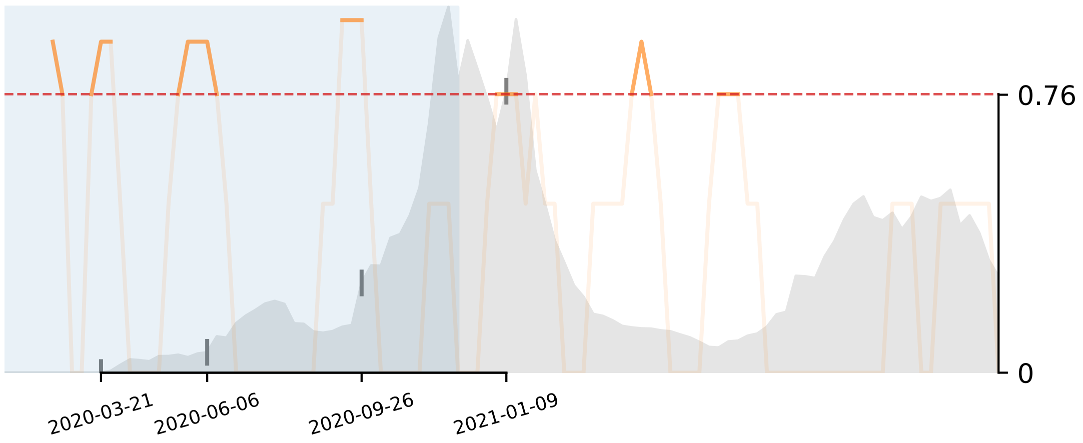

β	tp	fp	fn	tp	fp	fn
1.00	0	0	3	0	0	1
0.99	0	0	3	0	0	1
0.96	0	0	3	0	0	1
0.91	1	0	2	0	1	1
0.76	3	2	0	1	8	0
0.46	3	3	0	1	28	0
0.00	3	9	0	1	51	0

Vermont

Proxies selected by the Multiple Sources method

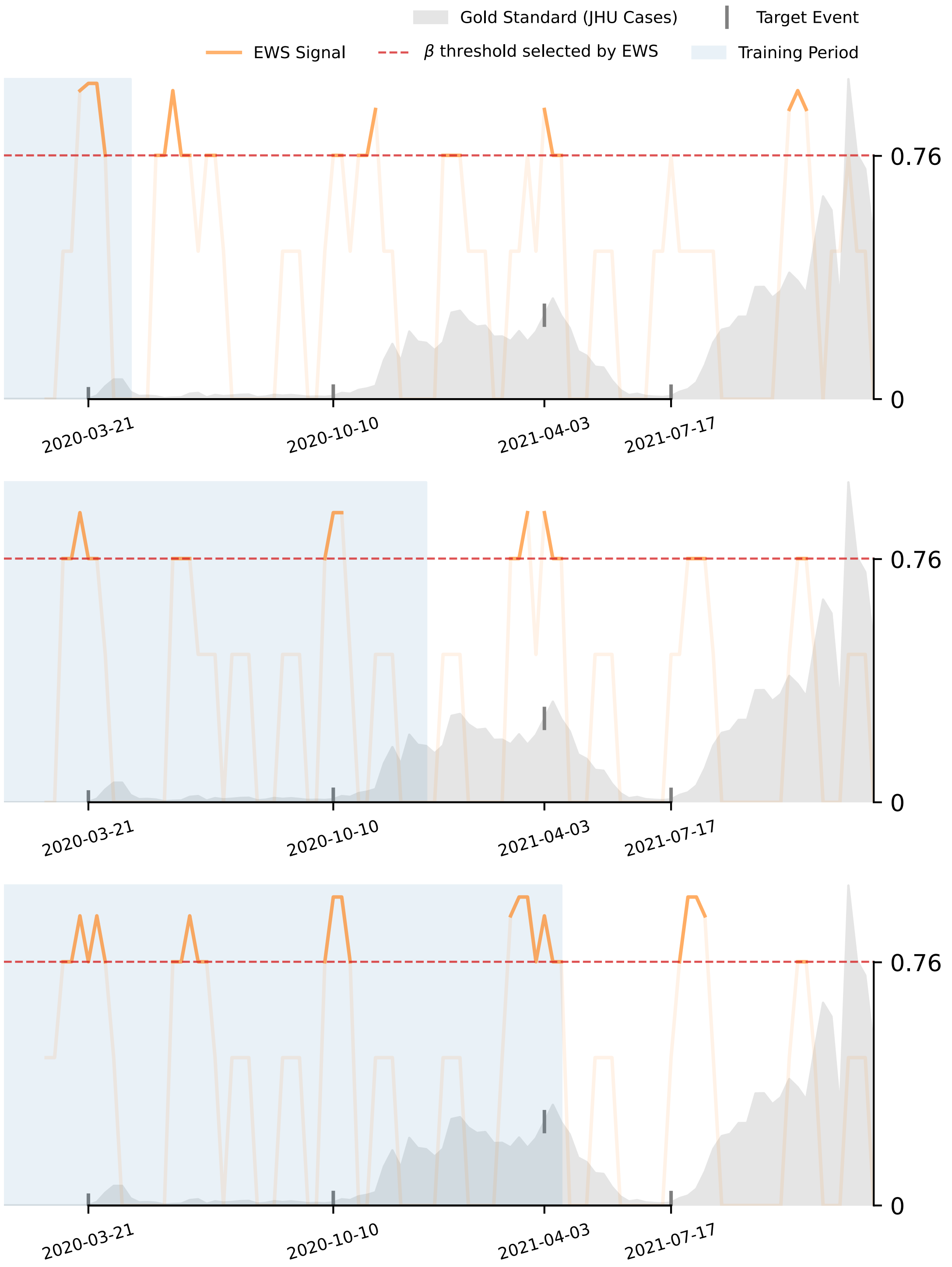

Performance as a function of β

Model 1

(GT) covid
(GT) covid-19
(GT) chest pain
(GT) covid symptoms
(GT) quarantine
(GT) fever

	Training			Out of sample		
β	tp	fp	fn	tp	fp	fn
1.00	0	0	1	0	0	3
0.99	0	0	1	0	0	3
0.96	1	1	0	0	2	3
0.91	1	1	0	1	5	2
0.76	1	1	0	3	20	0
0.46	1	2	0	3	42	0
0.00	1	2	0	3	67	0

Model 2

(GT) covid-19
(GT) fever
Twitter (state level signal)
(GT) covid

β	tp	fp	fn	tp	fp	fn
0.96	0	0	2	0	0	2
0.91	0	0	2	1	0	1
0.76	2	1	0	1	7	0
0.46	2	5	0	2	20	0
0.00	2	18	0	2	39	0

Model 3

(GT) covid-19
Twitter (state level signal)
(GT) covid
(GT) fever
(GT) Acute bronchitis
(JHU) cases

β	tp	fp	fn	tp	fp	fn
1.00	0	0	3	0	0	1
0.99	0	0	3	0	0	1
0.96	0	0	3	0	2	0
0.91	2	1	1	0	3	0
0.76	3	3	0	0	6	0
0.46	3	11	0	1	15	0
0.00	3	26	0	1	30	0

Virginia

Proxies selected by the Multiple Sources method

Performance as a function of β

Model 1

(GT) covid
(GT) how long does covid last
(GT) covid-19
(GT) chest pain
(GT) nose bleed
(GT) Acute bronchitis

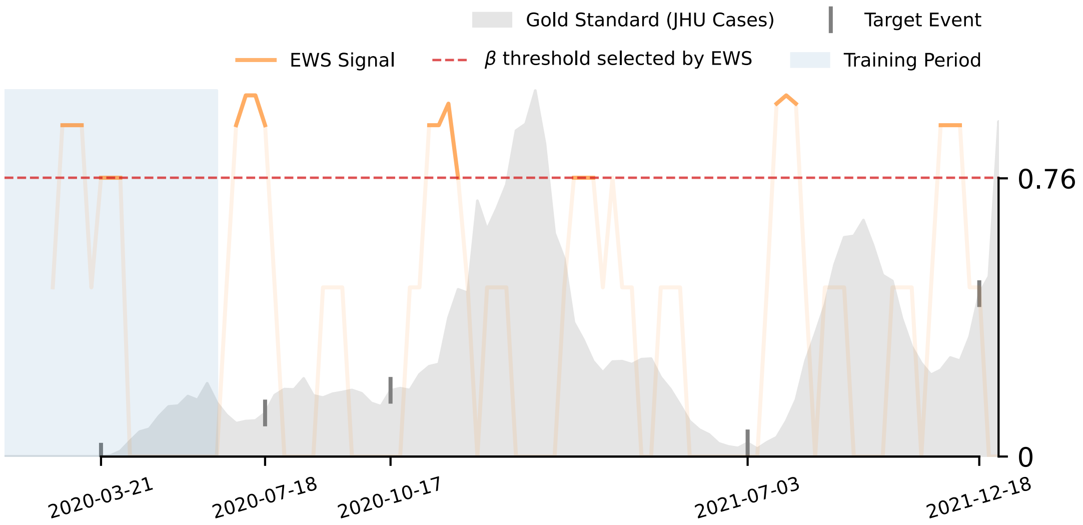

β	Training tp	Training fp	Training fn	Out of sample tp	Out of sample fp	Out of sample fn
1.00	0	0	1	0	0	4
0.99	0	0	1	1	1	3
0.96	0	0	1	1	4	3
0.91	0	0	1	2	6	2
0.76	1	0	0	2	11	2
0.46	1	2	0	3	34	0
0.00	1	3	0	4	55	0

Model 2

(GT) covid
(GT) how long does covid last
(GT) covid-19
(GT) chest pain
(GT) nose bleed
(GT) Acute bronchitis

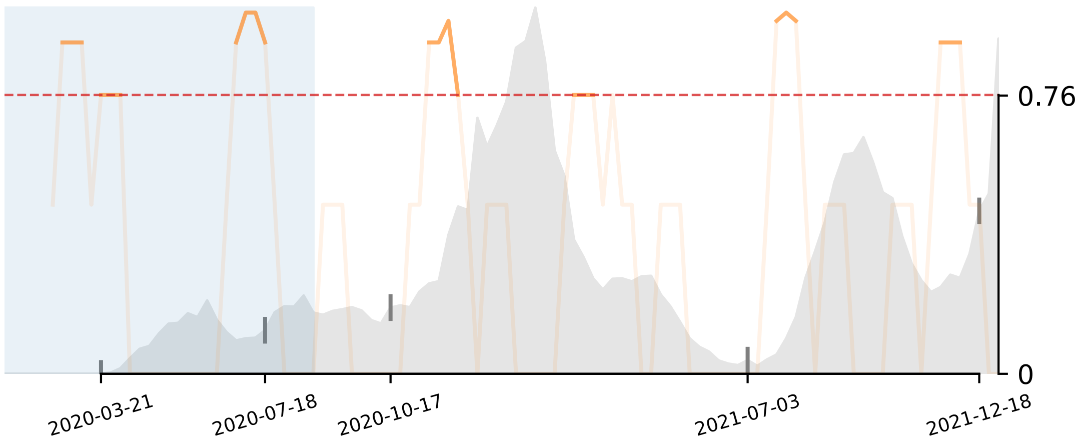

β	Training tp	Training fp	Training fn	Out of sample tp	Out of sample fp	Out of sample fn
1.00	0	0	2	0	0	3
0.99	0	0	2	0	1	3
0.96	1	0	1	0	4	3
0.91	1	0	1	1	6	2
0.76	2	0	0	1	11	2
0.46	2	2	0	2	33	0
0.00	2	4	0	3	50	0

Model 3

(GT) Anosmia
(GT) Asthma
(GT) Bronchitis
(GT) Cough
(GT) Throat irritation
(GT) Chest pain

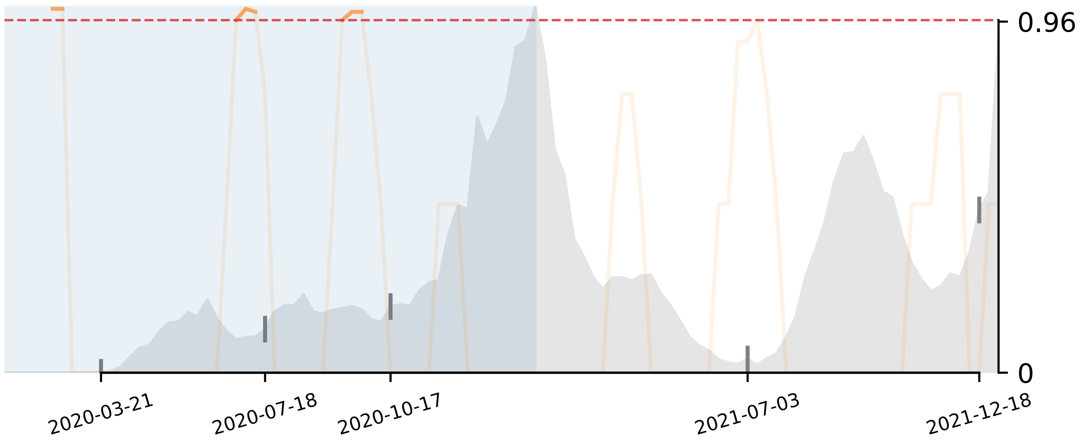

β	Training tp	Training fp	Training fn	Out of sample tp	Out of sample fp	Out of sample fn
1.00	0	0	3	0	0	2
0.99	2	1	1	0	0	2
0.96	3	1	0	0	1	1
0.91	3	2	0	1	1	1
0.76	3	2	0	2	4	0
0.46	3	2	0	2	10	0
0.00	3	6	0	2	34	0

Washington

Proxies selected by the Multiple Sources method

Model 1

(JHU) cases
(JHU) deaths
(GT) covid
(GT) how long does covid last
(GT) covid-19
(GT) chest pain

Model 2

(GT) Anxiety
(GT) Chest pain
(GT) Diarrhea
(GT) Vomiting
(GT) Fever
(GT) Otitis

Model 3

(GT) Chest pain
(GT) Vomiting
(GT) Fever
(GT) Otitis
(JHU) cases
(GT) Anxiety

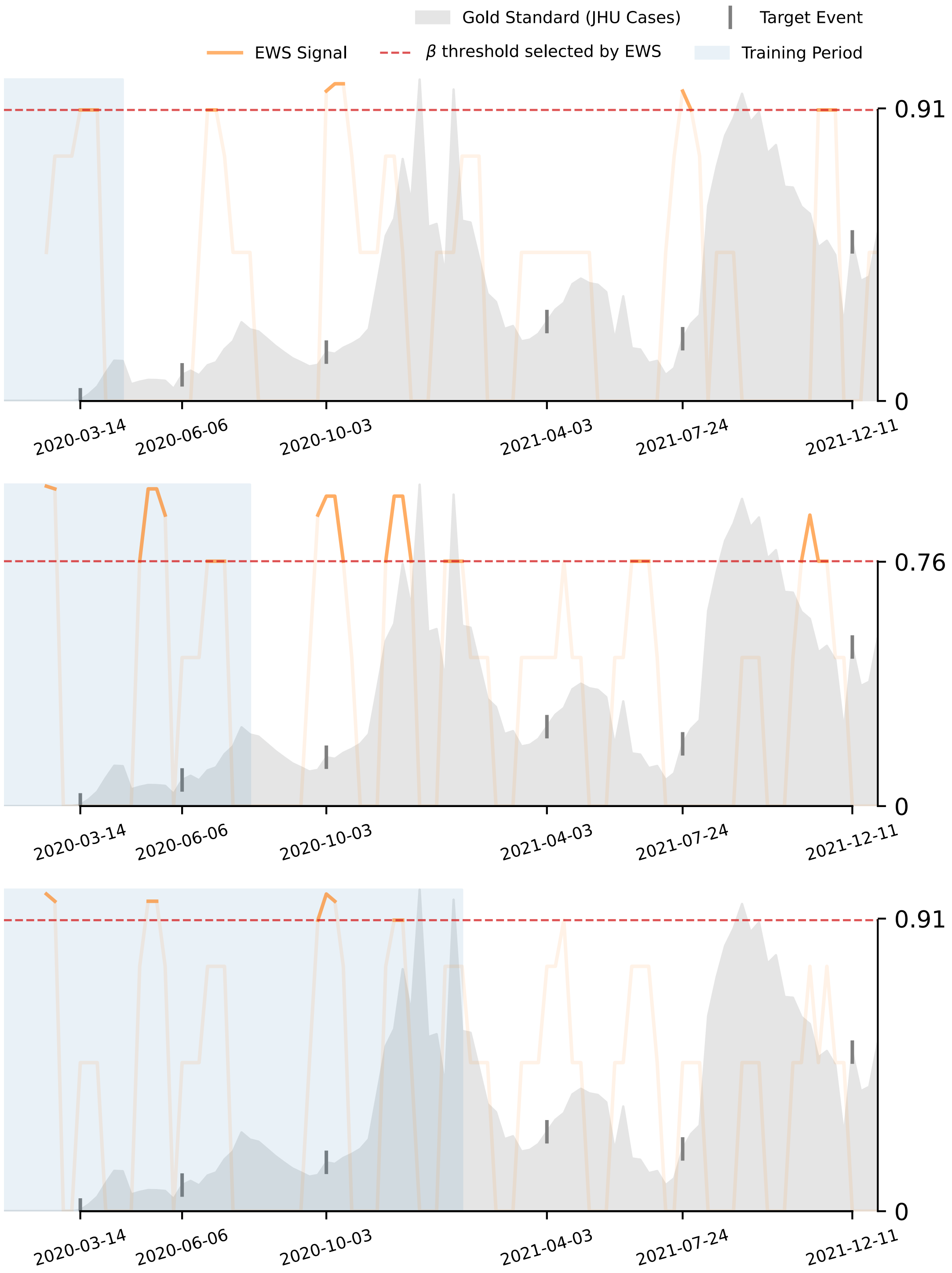

Performance as a function of β

Model 1

β	Training tp	Training fp	Training fn	Out of sample tp	Out of sample fp	Out of sample fn
1.00	0	0	1	0	0	5
0.99	0	0	1	0	2	4
0.96	0	0	1	2	2	3
0.91	0	0	1	3	5	2
0.76	1	2	0	3	13	2
0.46	1	2	0	4	34	0
0.00	1	2	0	5	54	0

Model 2

β	Training tp	Training fp	Training fn	Out of sample tp	Out of sample fp	Out of sample fn
1.00	0	0	2	0	0	4
0.99	1	0	1	0	0	4
0.96	2	0	0	1	3	3
0.91	2	0	0	2	3	2
0.76	2	0	0	3	10	0
0.46	2	4	0	4	23	0
0.00	2	6	0	4	46	0

Model 3

β	Training tp	Training fp	Training fn	Out of sample tp	Out of sample fp	Out of sample fn
1.00	0	0	3	0	0	3
0.99	0	0	3	0	0	3
0.96	2	0	1	0	0	3
0.91	3	1	0	0	1	2
0.76	3	3	0	3	2	0
0.46	3	12	0	3	15	0
0.00	3	17	0	3	28	0

Wisconsin

186

Proxies selected by the Multiple Sources method

Model 1

(GT) how long does covid last
(GT) chest pain
(GT) chest tightness
(GT) covid-19 who
(GT) covid
(GT) covid-19

Model 2

(GT) chest pain
(GT) how long does covid last
(GT) quarantine
(GT) Ageusia
(GT) Anosmia
(GT) Bronchitis

Model 3

(GT) how long does covid last
(GT) Hyperventilation
(GT) Cough
(GT) Nasal congestion
(GT) side effects of vaccine
(GT) Ageusia

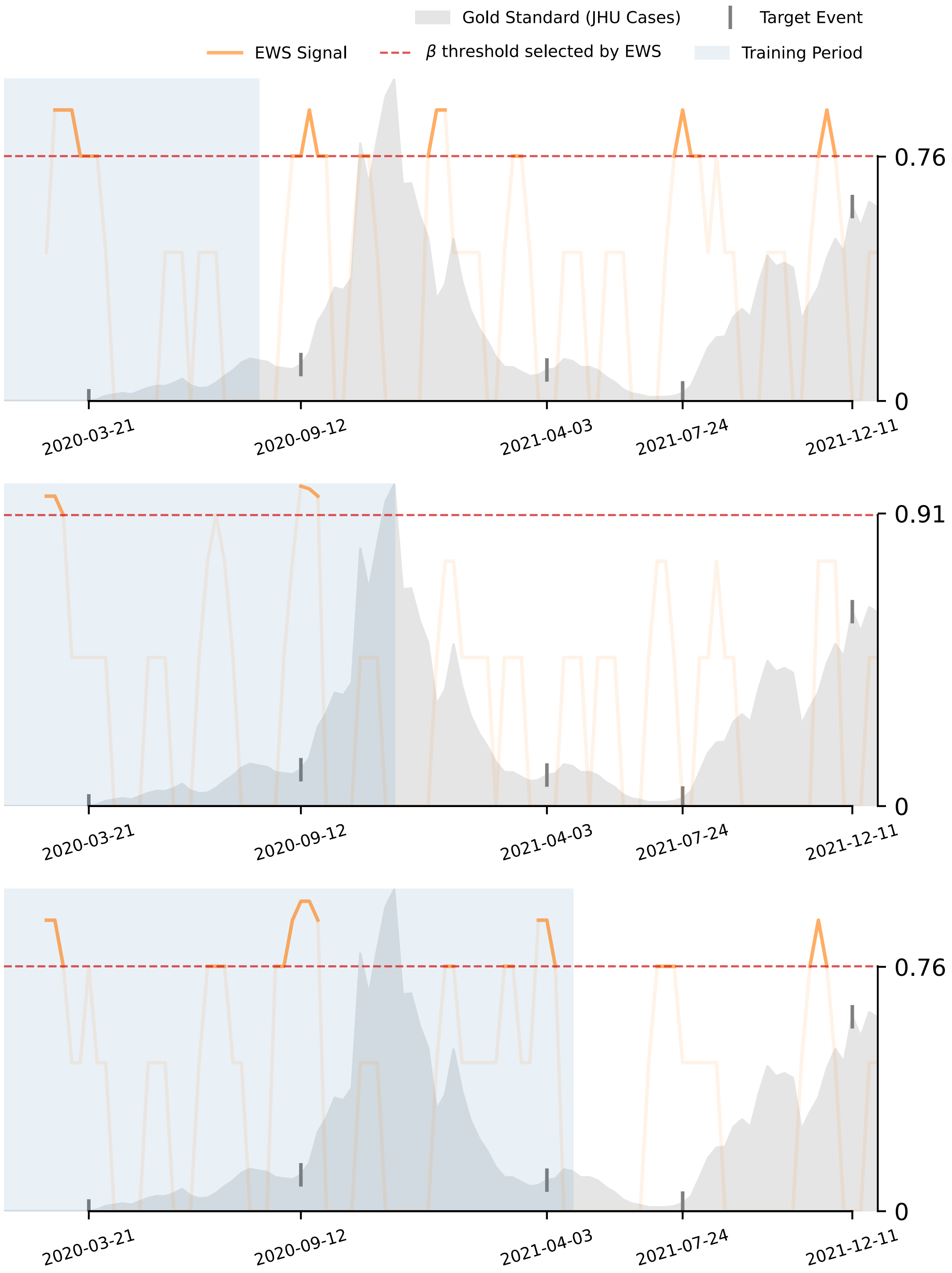

Performance as a function of β

Model 1

	Training			Out of sample		
β	tp	fp	fn	tp	fp	fn
1.00	0	0	1	0	0	4
0.99	0	0	1	0	0	4
0.96	0	0	1	0	0	4
0.91	0	0	1	2	3	1
0.76	1	0	0	4	11	0
0.46	1	1	0	4	31	0
0.00	1	9	0	4	47	0

Model 2

β	tp	fp	fn	tp	fp	fn
1.00	0	0	2	0	0	3
0.99	1	0	1	0	0	3
0.96	1	1	1	0	0	3
0.91	2	3	0	0	0	3
0.76	2	4	0	2	3	1
0.46	2	6	0	3	20	0
0.00	2	16	0	3	36	0

Model 3

β	tp	fp	fn	tp	fp	fn
1.00	0	0	3	0	0	2
0.99	0	0	3	0	0	2
0.96	0	0	3	0	0	2
0.91	1	1	2	1	0	1
0.76	3	3	0	2	0	0
0.46	3	10	0	2	6	0
0.00	3	27	0	2	22	0

Proxies selected by the Multiple Sources method

Model 1

(JHU) cases
(GT) covid
(GT) covid-19
(GT) covid symptoms
(GT) quarantine
(GT) fever

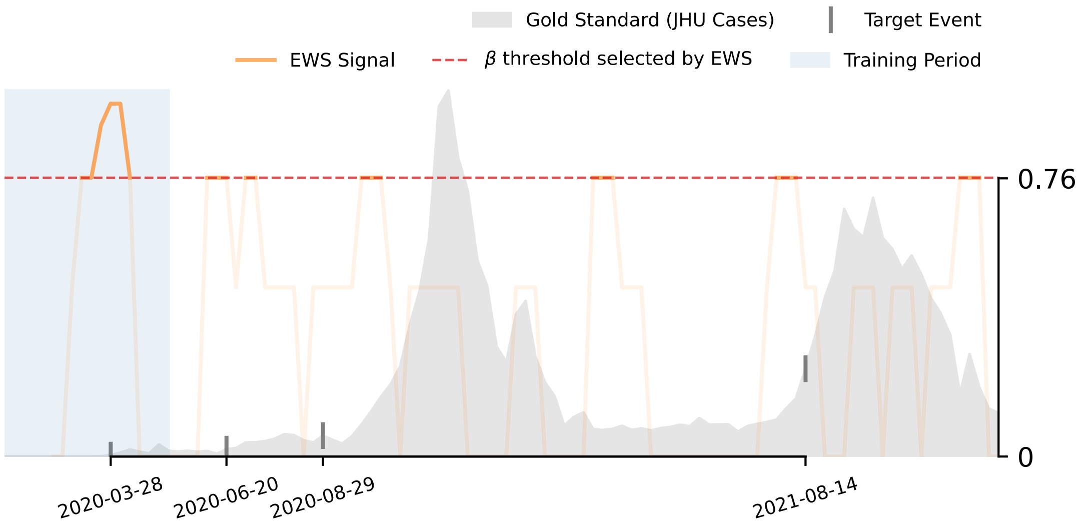

Performance as a function of β

β	Training tp	Training fp	Training fn	Out of sample tp	Out of sample fp	Out of sample fn
1.00	0	0	1	0	0	3
0.99	0	0	1	0	0	3
0.96	0	0	1	0	0	3
0.91	1	1	0	0	0	3
0.76	1	1	0	2	11	1
0.46	1	2	0	3	38	0
0.00	1	2	0	3	66	0

Model 2

(JHU) cases
(GT) Nasal congestion
(GT) fever

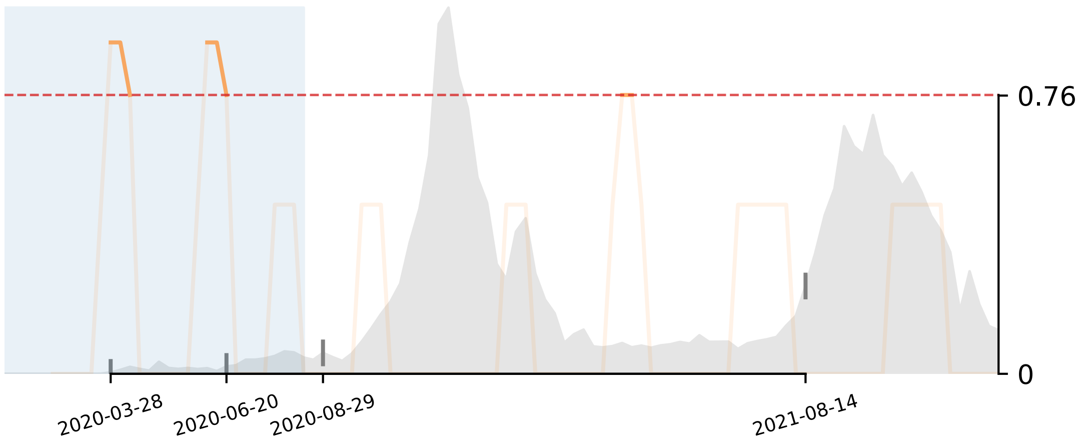

β	Training tp	Training fp	Training fn	Out of sample tp	Out of sample fp	Out of sample fn
0.91	0	0	2	0	0	2
0.76	2	1	0	0	2	2
0.46	2	2	0	1	17	1
0.00	2	5	0	2	63	0

Model 3

(GT) fever
(GT) covid symptoms
(GT) Nasal congestion
(JHU) cases
(GT) Cardiac arrest

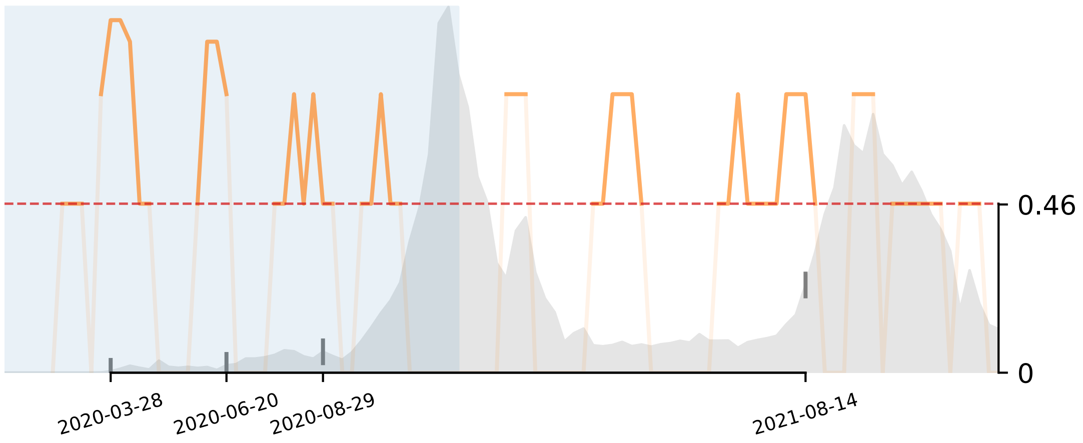

β	Training tp	Training fp	Training fn	Out of sample tp	Out of sample fp	Out of sample fn
0.99	0	0	3	0	0	1
0.96	0	0	3	0	0	1
0.91	1	1	2	0	0	1
0.76	2	2	1	1	10	0
0.46	3	3	0	1	25	0
0.00	3	12	0	1	49	0

Figures S188-S189.
Comparative analysis between the outbreak onset dates as created by using Parag’s method against the CDC's daily transmission levels. (A) represents a histogram on the number of 'associated events' between both sources (associated events occur in time within a time difference of at most 6 weeks). (B) Represents the timing (in weeks) between associated events over all counties. (C) The number of non-associated onsets within the CDC's data. Panels (D) and (E) show a count of the number of events expected to see within a county.

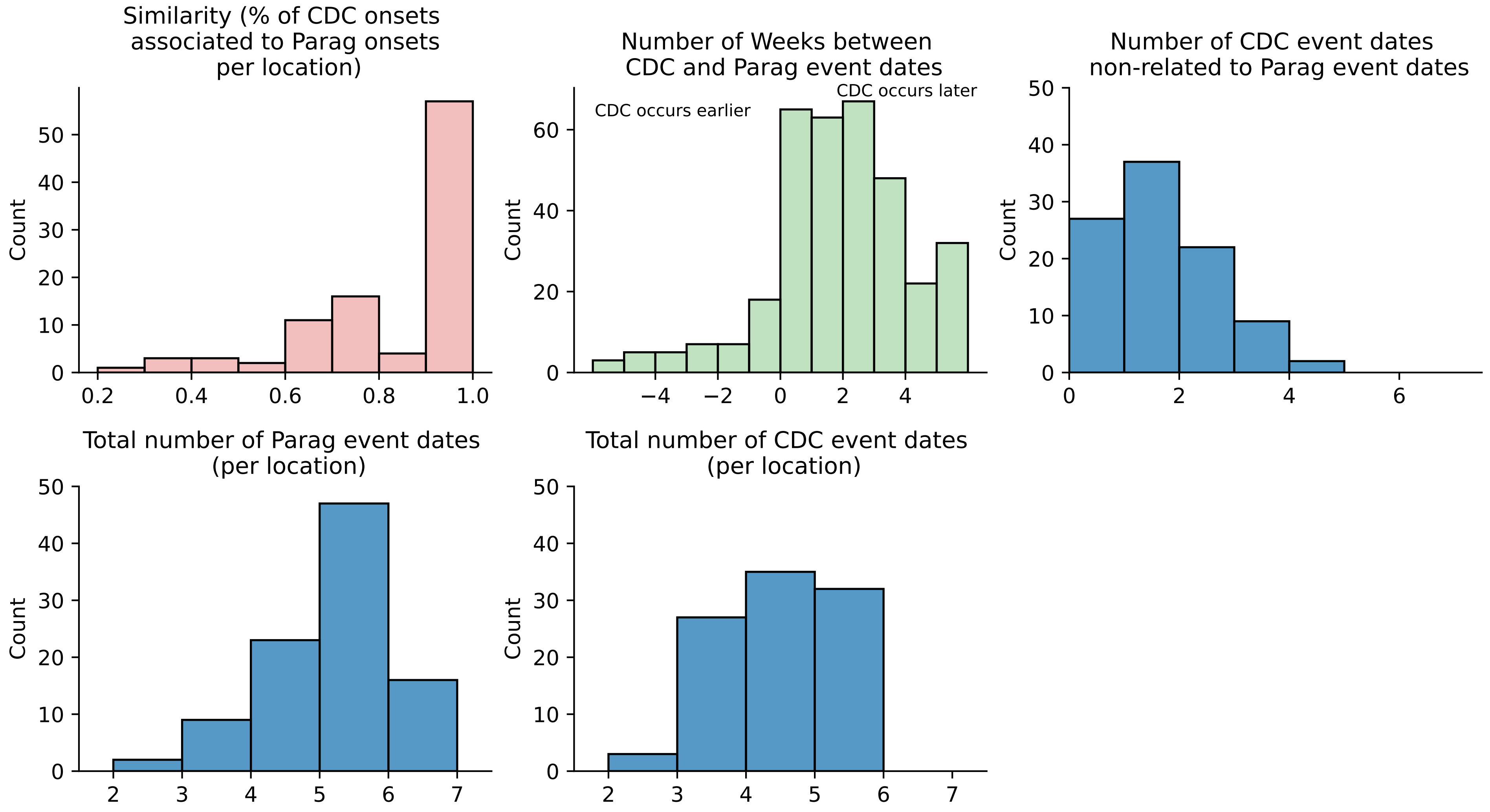

Similarity (% of CDC onsets associated to Parag onsets per location)
Count
0.2
0.4
0.6
0.8
1.0
Number of Weeks between CDC and Parag event dates
CDC occurs earlier
CDC occurs later
−4
−2
0
2
4
Number of CDC event dates non-related to Parag event dates
Total number of Parag event dates (per location)
Total number of CDC event dates (per location)

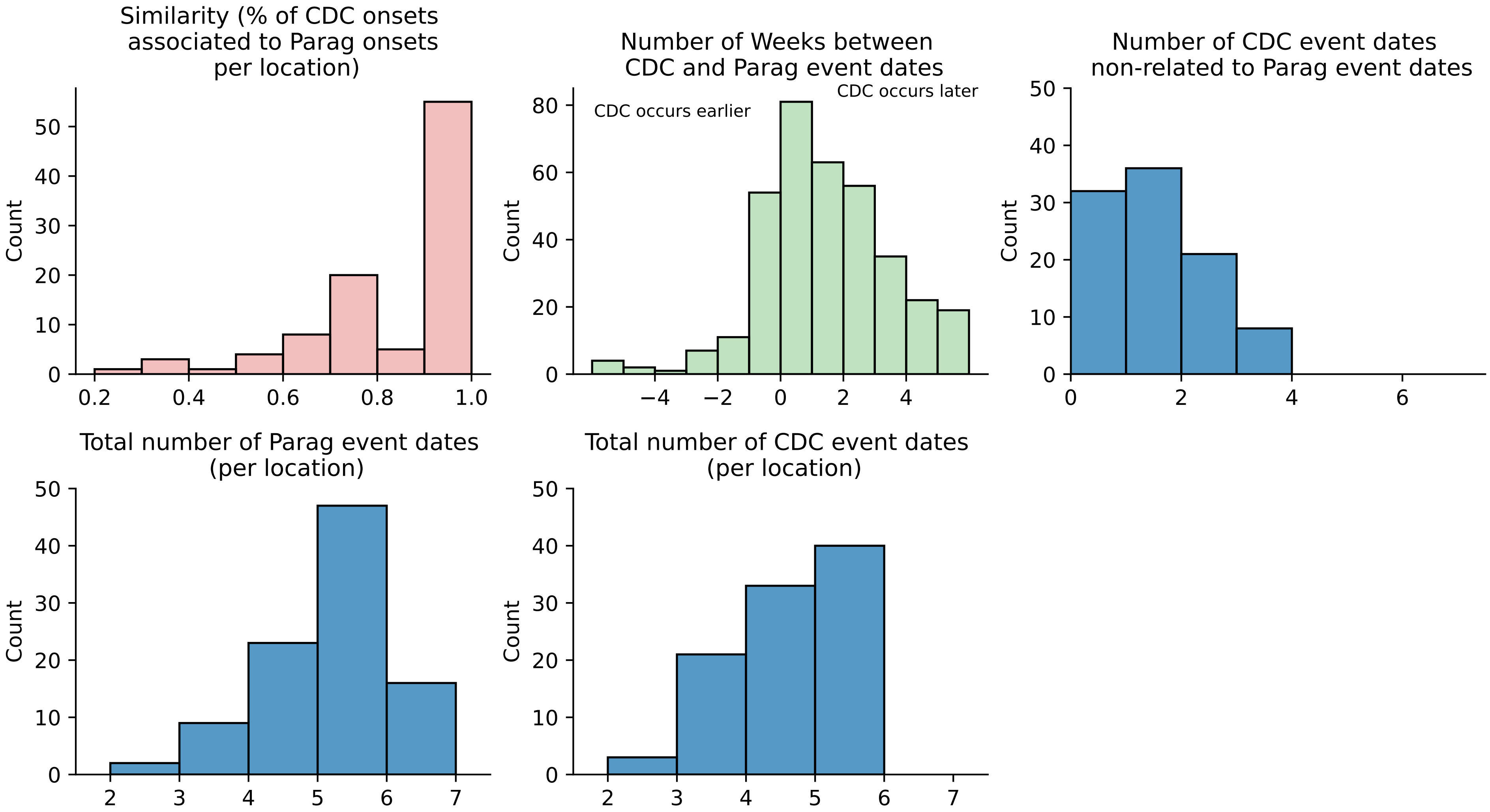

Similarity (% of CDC onsets associated to Parag onsets per location)
Count
Number of Weeks between CDC and Parag event dates
CDC occurs earlier
CDC occurs later
Number of CDC event dates non-related to Parag event dates
Total number of Parag event dates (per location)
Total number of CDC event dates (per location)

Figures S190-S286.
Visual comparison (per county) of the probability of resurgence (timeseries in green, onsets as vertical dotted red lines),against the onsets generated using CDC's COVID-19 transmission-level data (timeseries in blue, onsets as vertical blue tick marks).

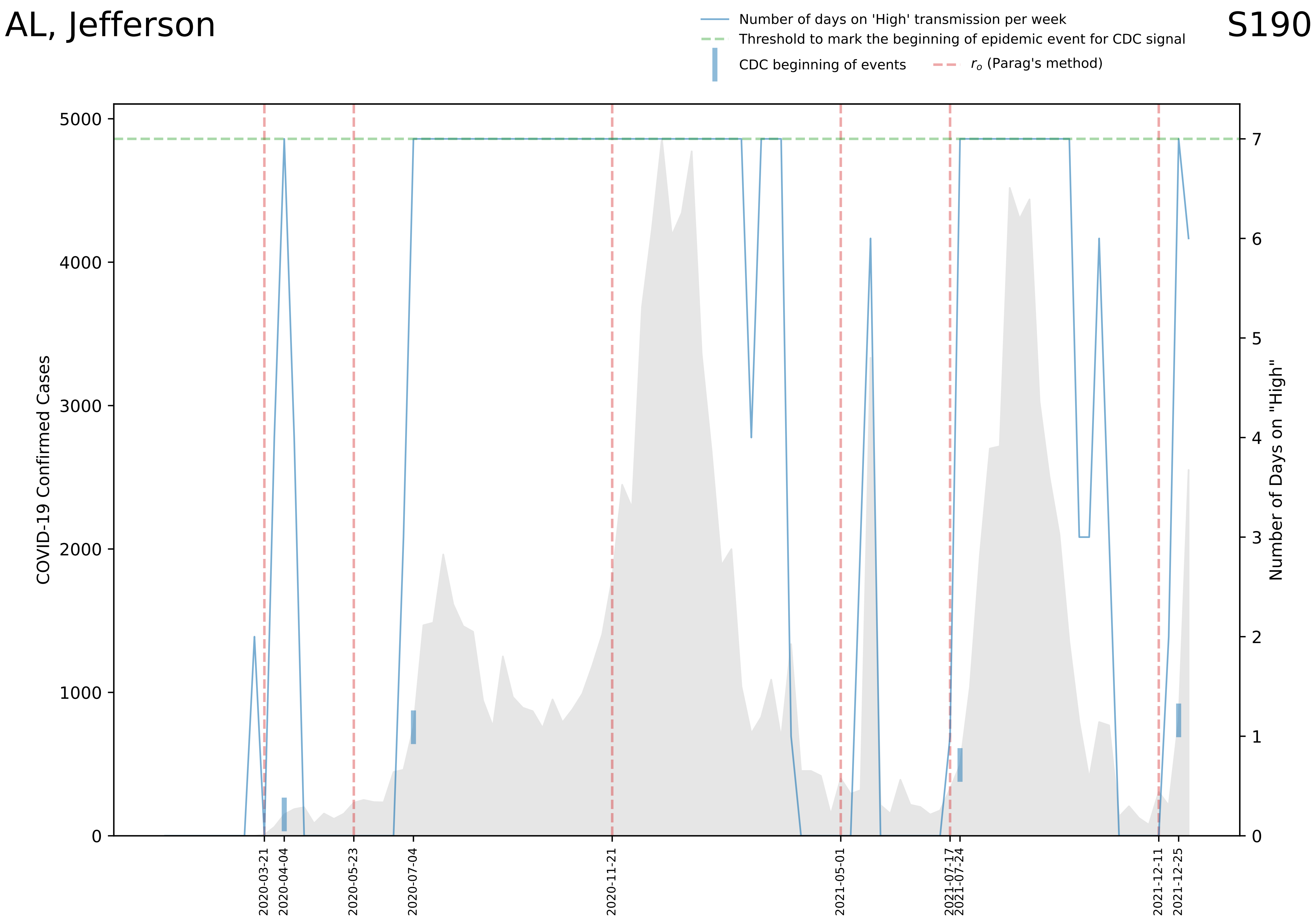

AL, Jefferson
S190
Number of days on 'High' transmission per week
Threshold to mark the beginning of epidemic event for CDC signal
CDC beginning of events
r_o (Parag's method)
COVID-19 Confirmed Cases
Number of Days on "High"
0
1000
2000
3000
4000
5000
0
1
2
3
4
5
6
7
2020-03-21
2020-04-04
2020-05-23
2020-07-04
2020-11-21
2021-05-01
2021-07-17
2021-07-24
2021-12-11
2021-12-25

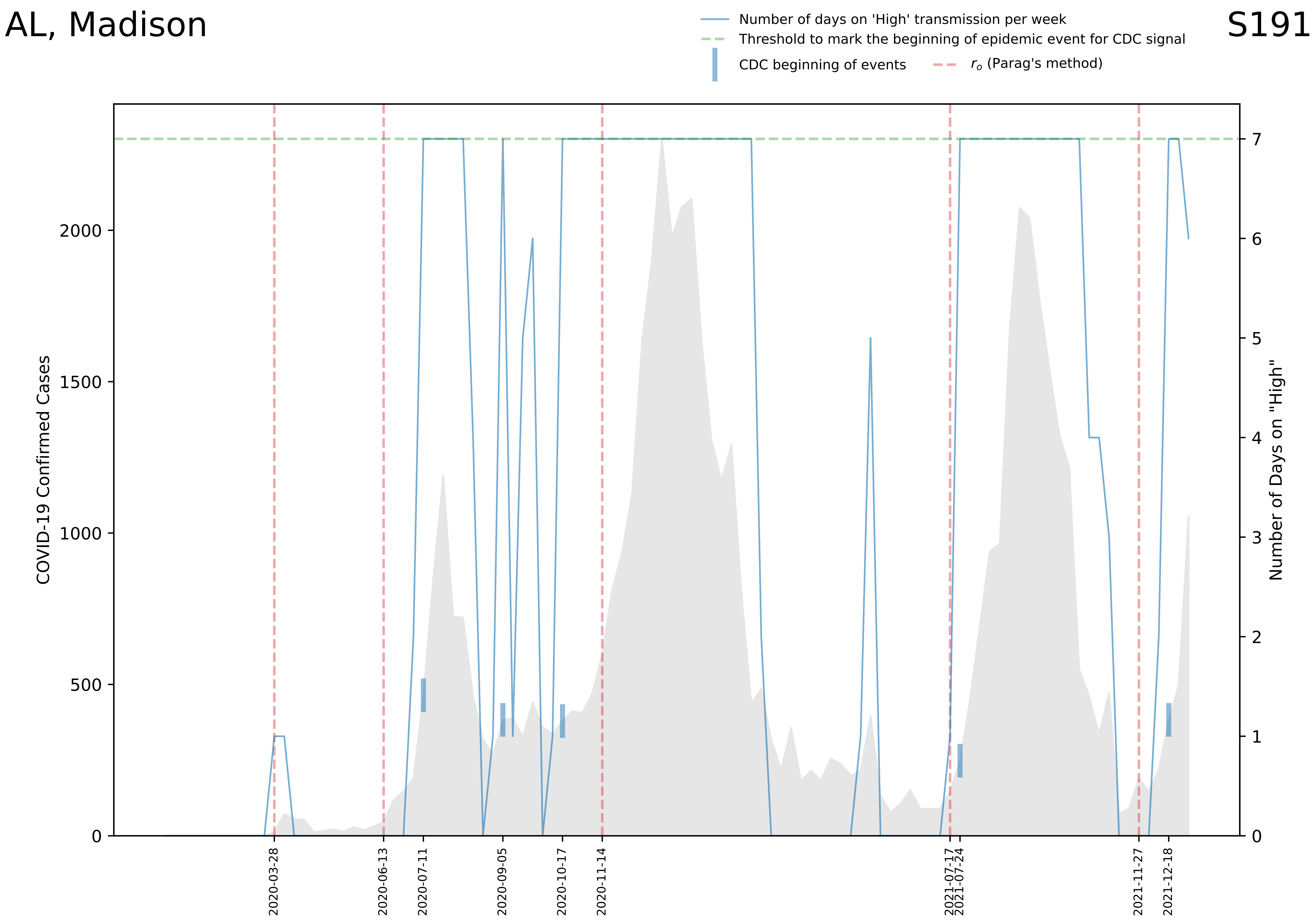

AL, Madison
S191
Number of days on 'High' transmission per week
Threshold to mark the beginning of epidemic event for CDC signal
CDC beginning of events
r_o (Parag's method)
COVID-19 Confirmed Cases
Number of Days on "High"
0
500
1000
1500
2000
0
1
2
3
4
5
6
7
2020-03-28
2020-06-13
2020-07-11
2020-09-05
2020-10-17
2020-11-14
2021-07-17
2021-07-24
2021-11-27
2021-12-18

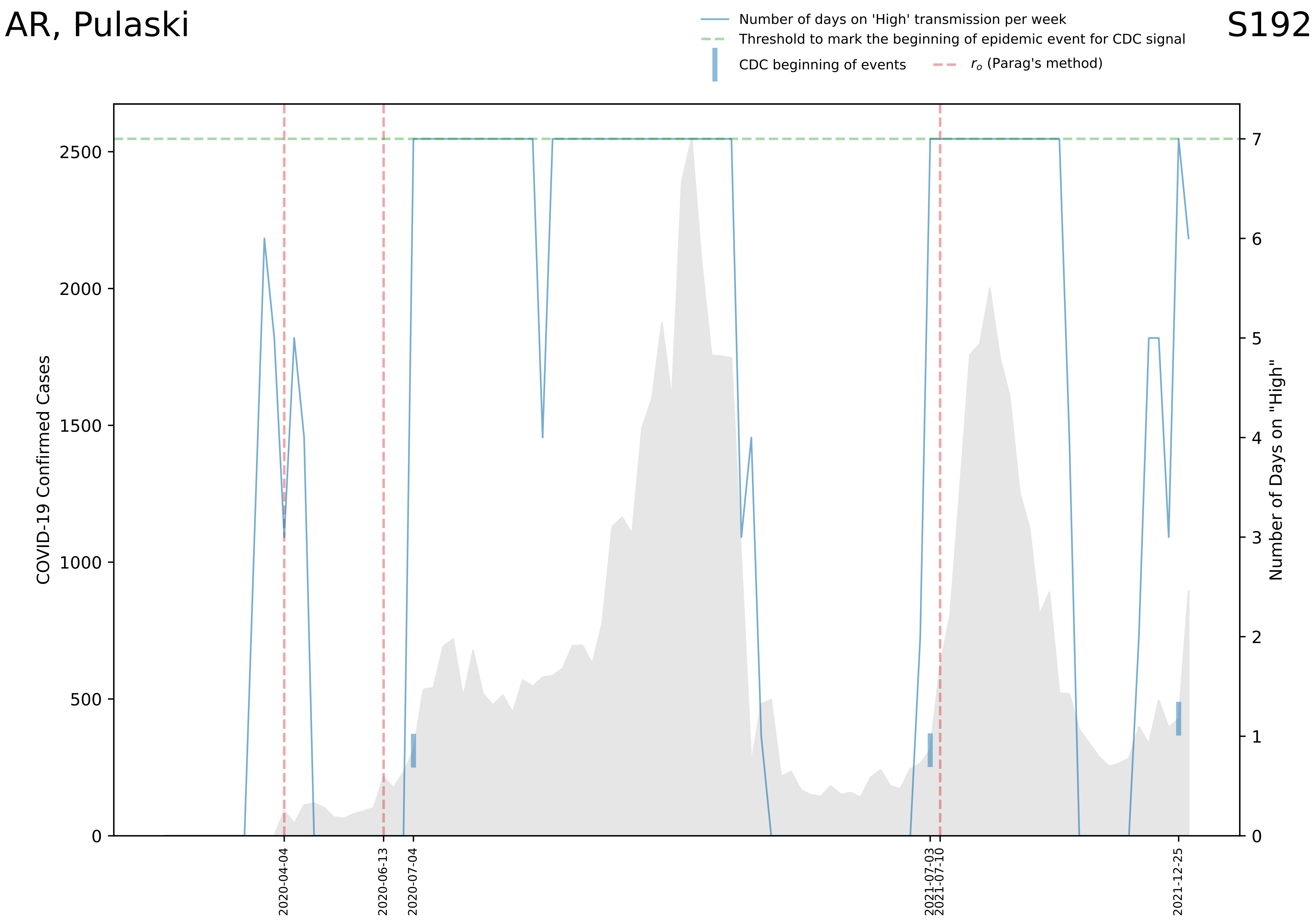

AR, Pulaski
S192
Number of days on 'High' transmission per week
Threshold to mark the beginning of epidemic event for CDC signal
CDC beginning of events
r_o (Parag's method)
COVID-19 Confirmed Cases
Number of Days on "High"
0
500
1000
1500
2000
2500
0
1
2
3
4
5
6
7
2020-04-04
2020-06-13
2020-07-04
2021-07-03
2021-07-10
2021-12-25

AZ, Maricopa

S193

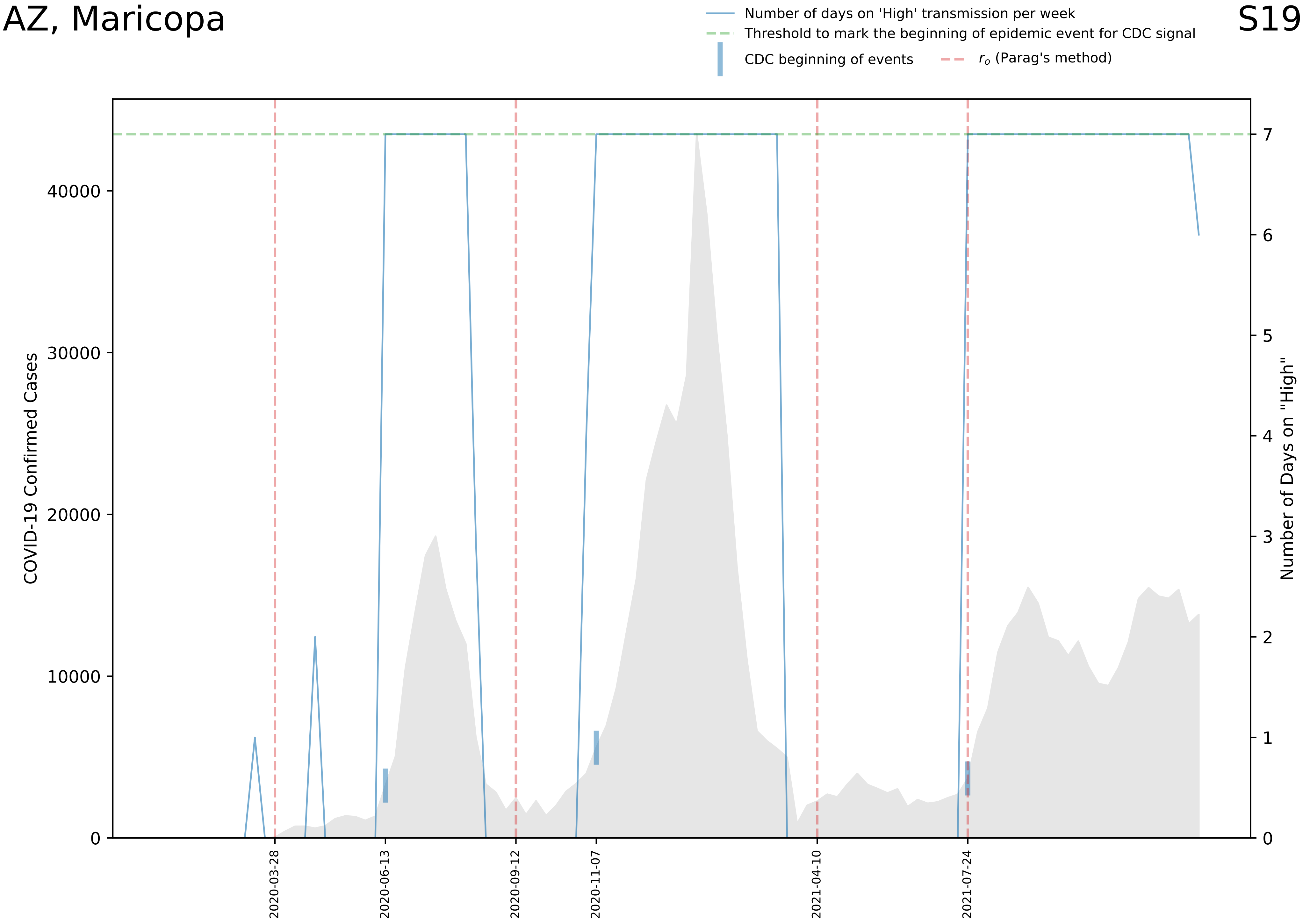

AZ, Pima

S194

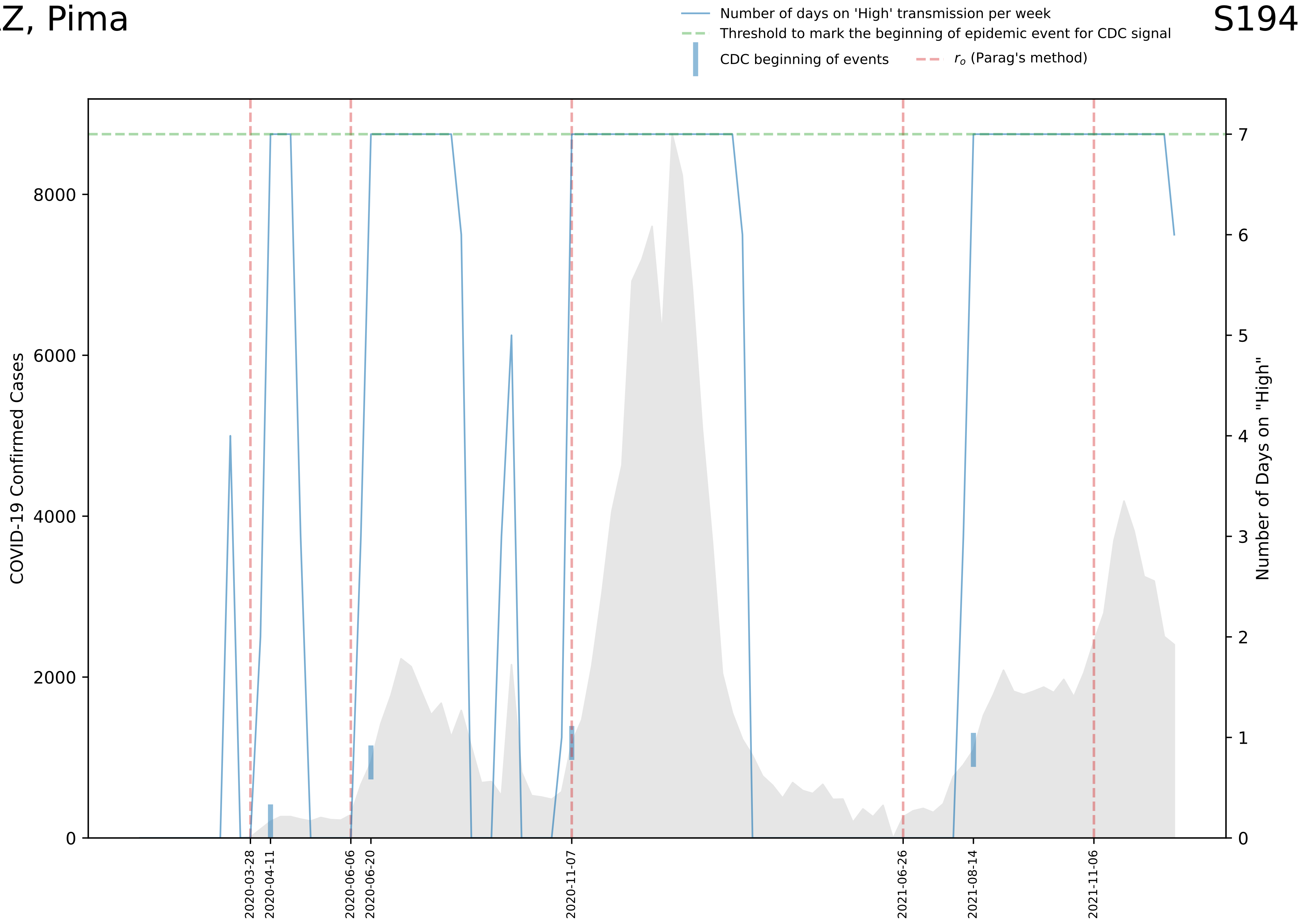

Number of days on 'High' transmission per week
Threshold to mark the beginning of epidemic event for CDC signal
CDC beginning of events
r_o (Parag's method)
COVID-19 Confirmed Cases
Number of Days on "High"
0
2000
4000
6000
8000
0
1
2
3
4
5
6
7
2020-03-28
2020-04-11
2020-06-06
2020-06-20
2020-11-07
2021-06-26
2021-08-14
2021-11-06

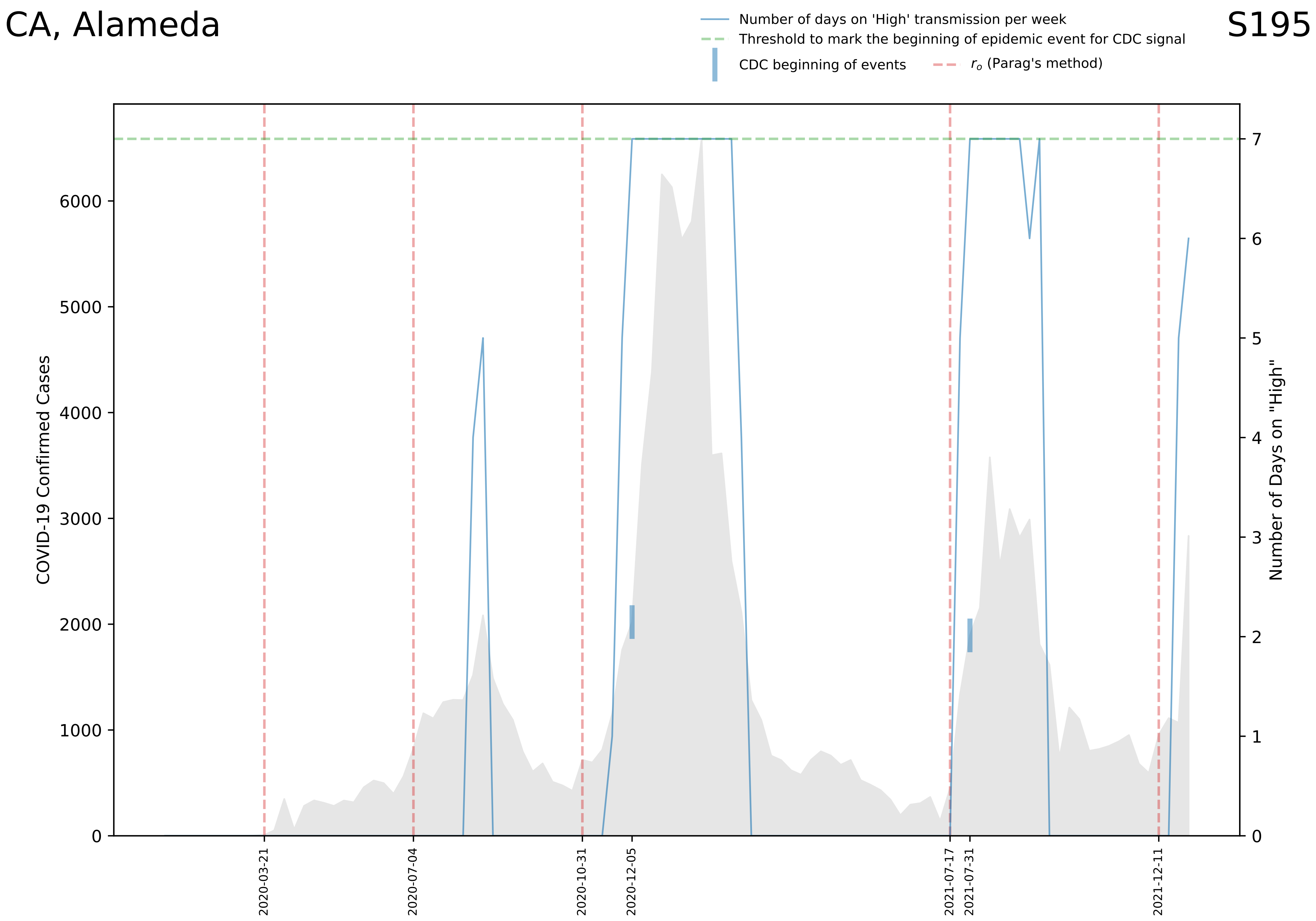

CA, Alameda
S195
Number of days on 'High' transmission per week
Threshold to mark the beginning of epidemic event for CDC signal
CDC beginning of events
r_o (Parag's method)
COVID-19 Confirmed Cases
Number of Days on "High"
0
1000
2000
3000
4000
5000
6000
0
1
2
3
4
5
6
7
2020-03-21
2020-07-04
2020-10-31
2020-12-05
2021-07-17
2021-07-31
2021-12-11

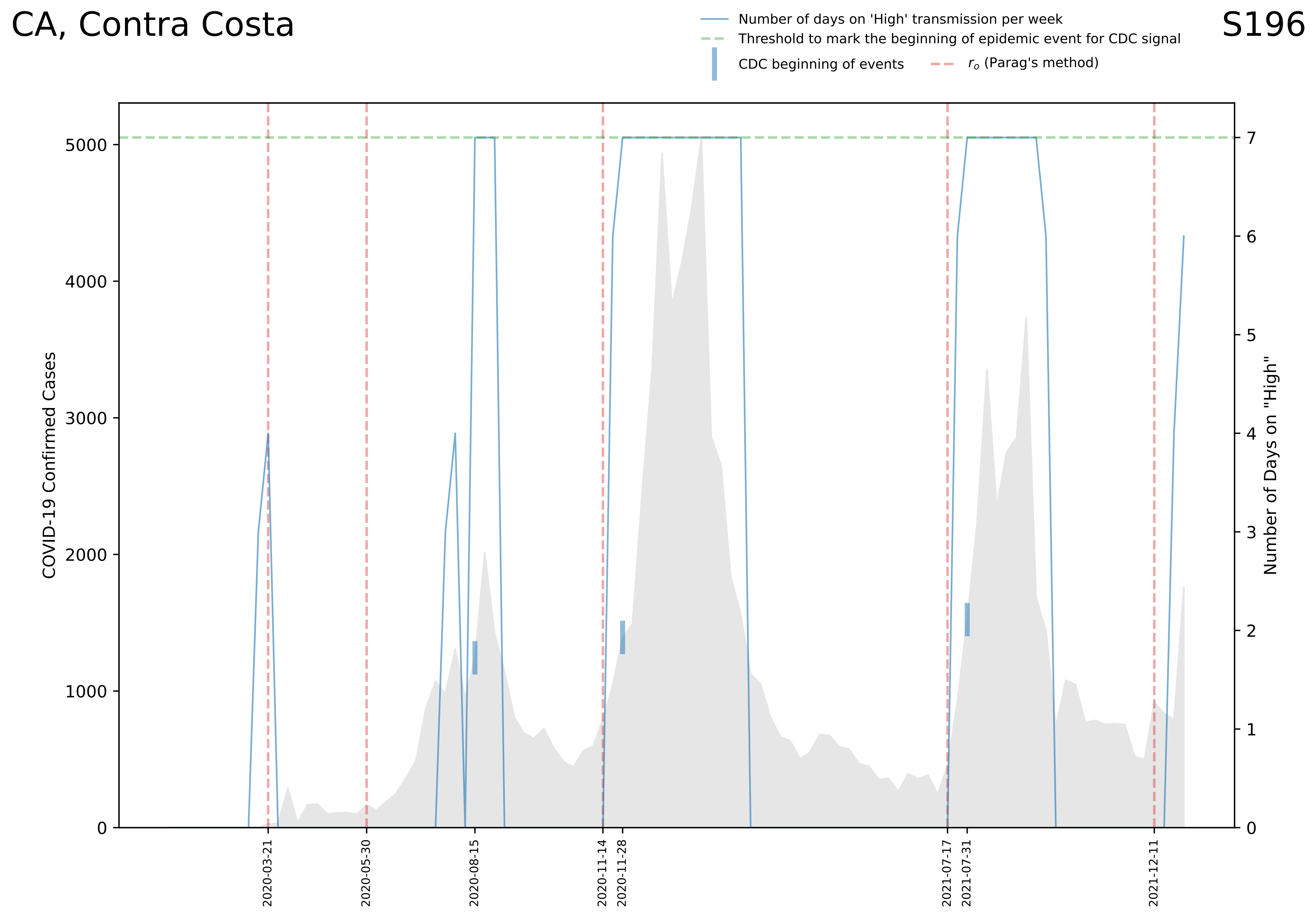

CA, Contra Costa
S196
Number of days on 'High' transmission per week
Threshold to mark the beginning of epidemic event for CDC signal
CDC beginning of events
r_o (Parag's method)
COVID-19 Confirmed Cases
Number of Days on "High"
0
1000
2000
3000
4000
5000
0
1
2
3
4
5
6
7
2020-03-21
2020-05-30
2020-08-15
2020-11-14
2020-11-28
2021-07-17
2021-07-31
2021-12-11

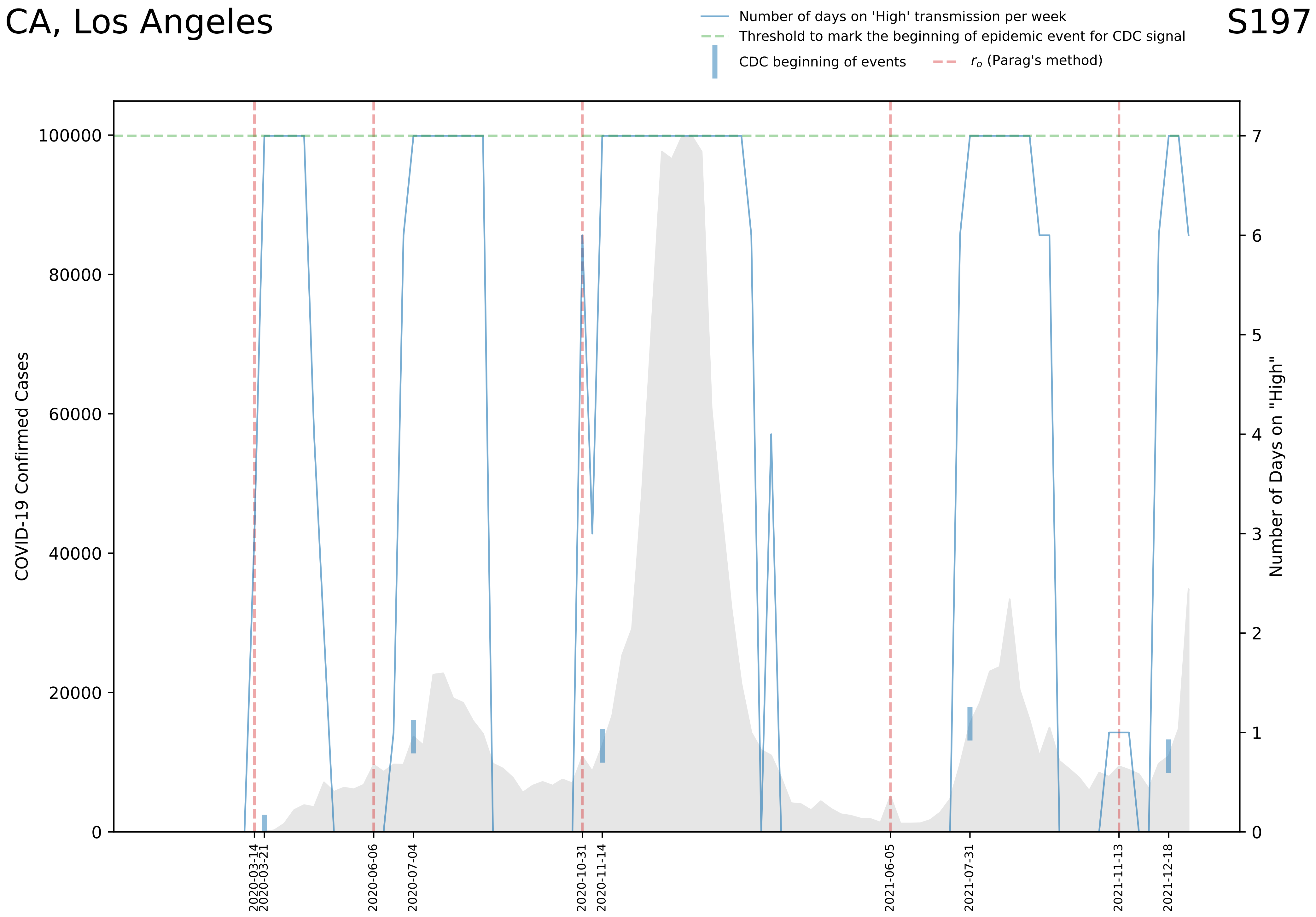

CA, Los Angeles
S197
Number of days on 'High' transmission per week
Threshold to mark the beginning of epidemic event for CDC signal
CDC beginning of events
r_o (Parag's method)
COVID-19 Confirmed Cases
Number of Days on "High"
0
20000
40000
60000
80000
100000
0
1
2
3
4
5
6
7
2020-03-14
2020-03-21
2020-06-06
2020-07-04
2020-10-31
2020-11-14
2021-06-05
2021-07-31
2021-11-13
2021-12-18

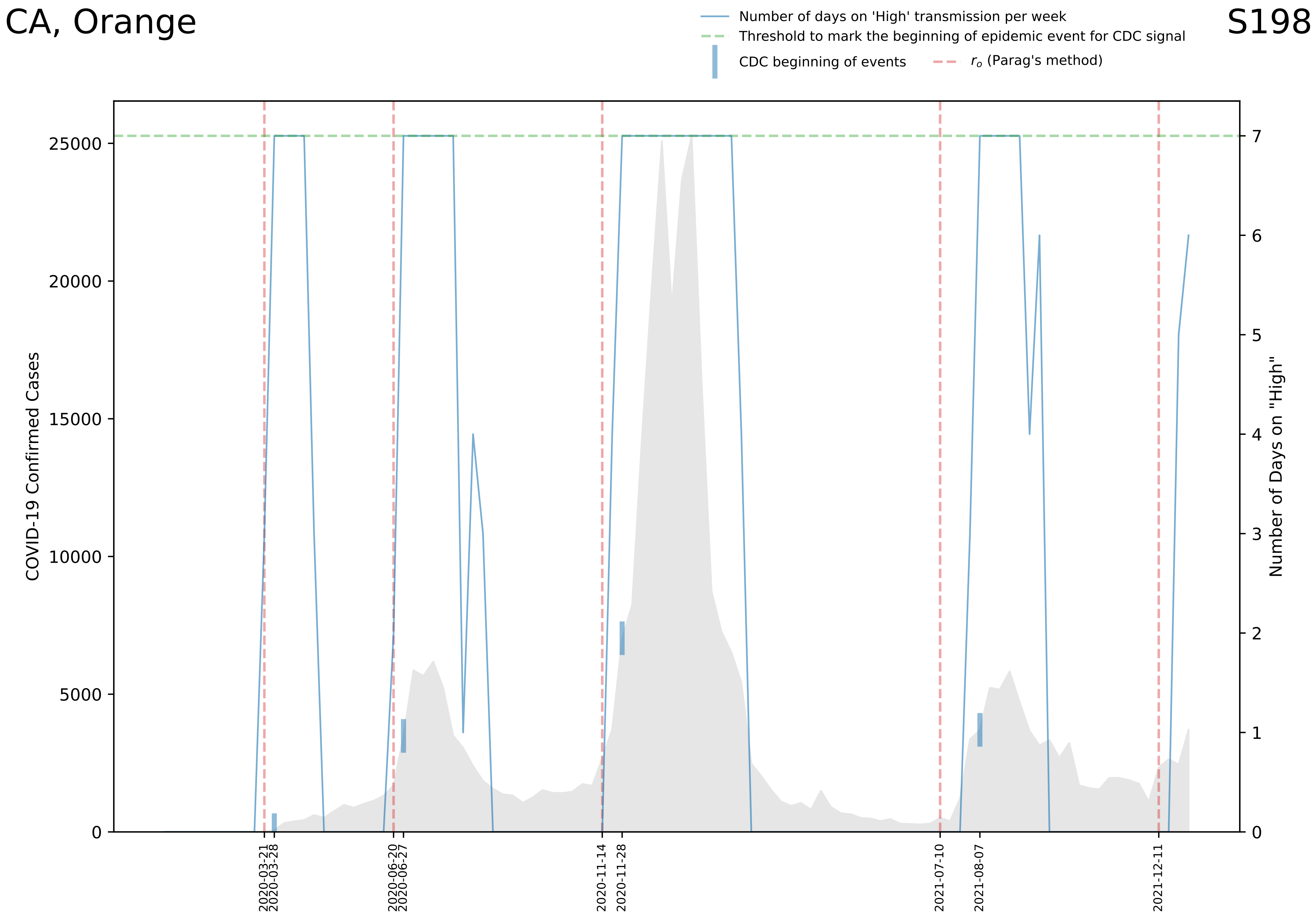

CA, Orange
S198
Number of days on 'High' transmission per week
Threshold to mark the beginning of epidemic event for CDC signal
CDC beginning of events
r_o (Parag's method)
COVID-19 Confirmed Cases
Number of Days on "High"
0
5000
10000
15000
20000
25000
0
1
2
3
4
5
6
7
2020-03-21
2020-03-28
2020-06-20
2020-06-27
2020-11-14
2020-11-28
2021-07-10
2021-08-07
2021-12-11

CA, Riverside

S199

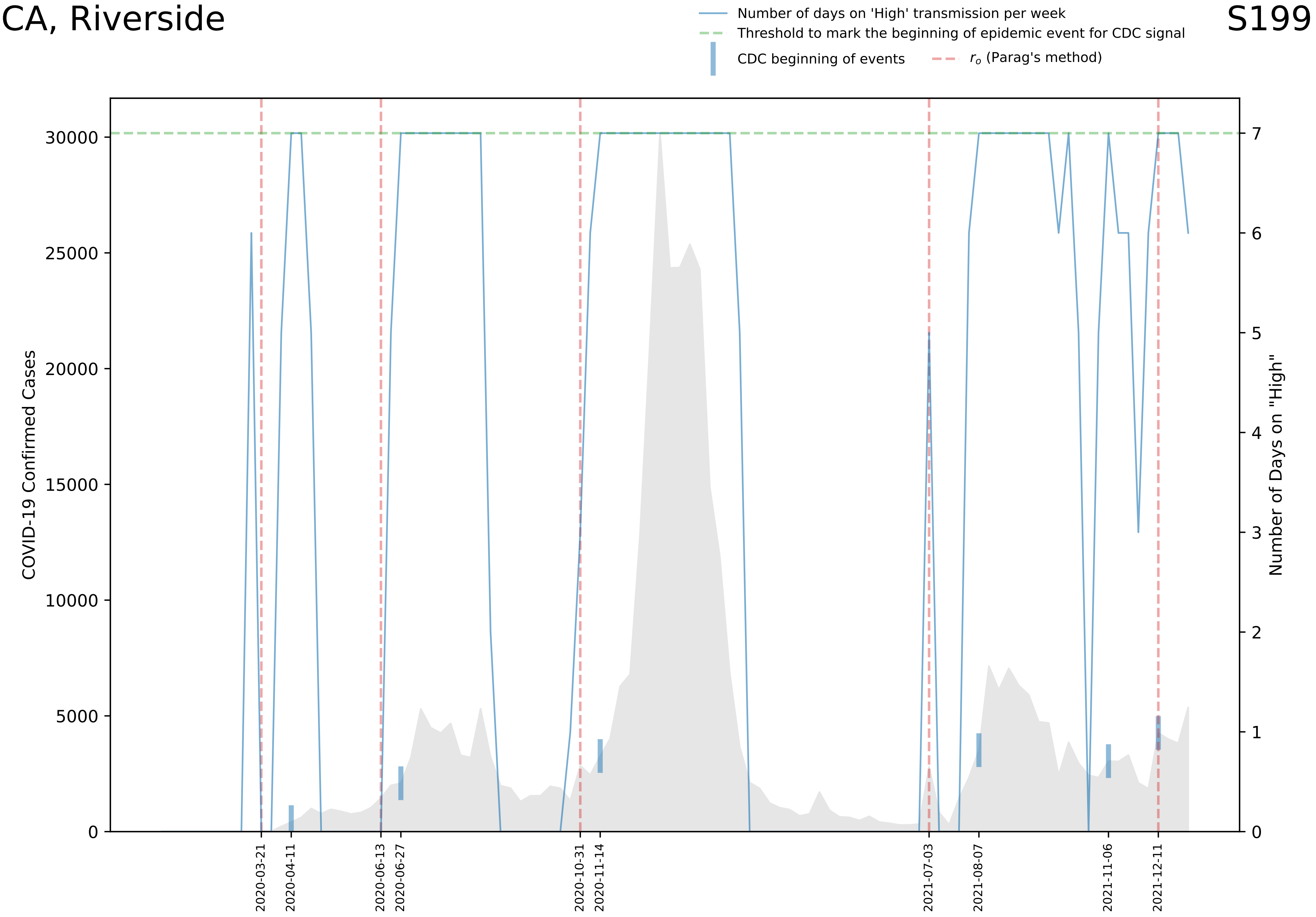

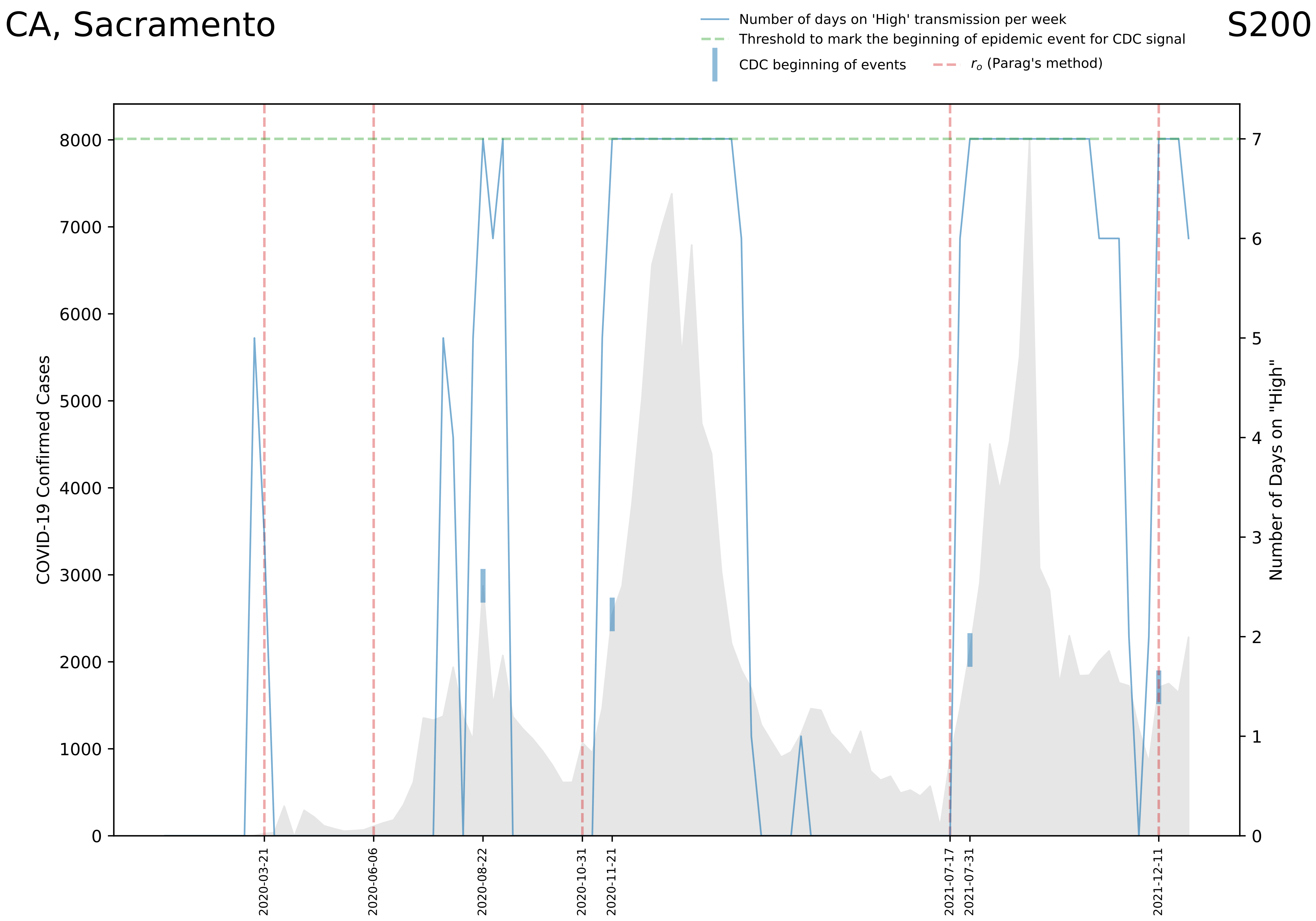

CA, Sacramento
S200
Number of days on 'High' transmission per week
Threshold to mark the beginning of epidemic event for CDC signal
CDC beginning of events
r_o (Parag's method)
COVID-19 Confirmed Cases
Number of Days on "High"
0
1000
2000
3000
4000
5000
6000
7000
8000
0
1
2
3
4
5
6
7
2020-03-21
2020-06-06
2020-08-22
2020-10-31
2020-11-21
2021-07-17
2021-07-31
2021-12-11

CA, San Bernardino

S201

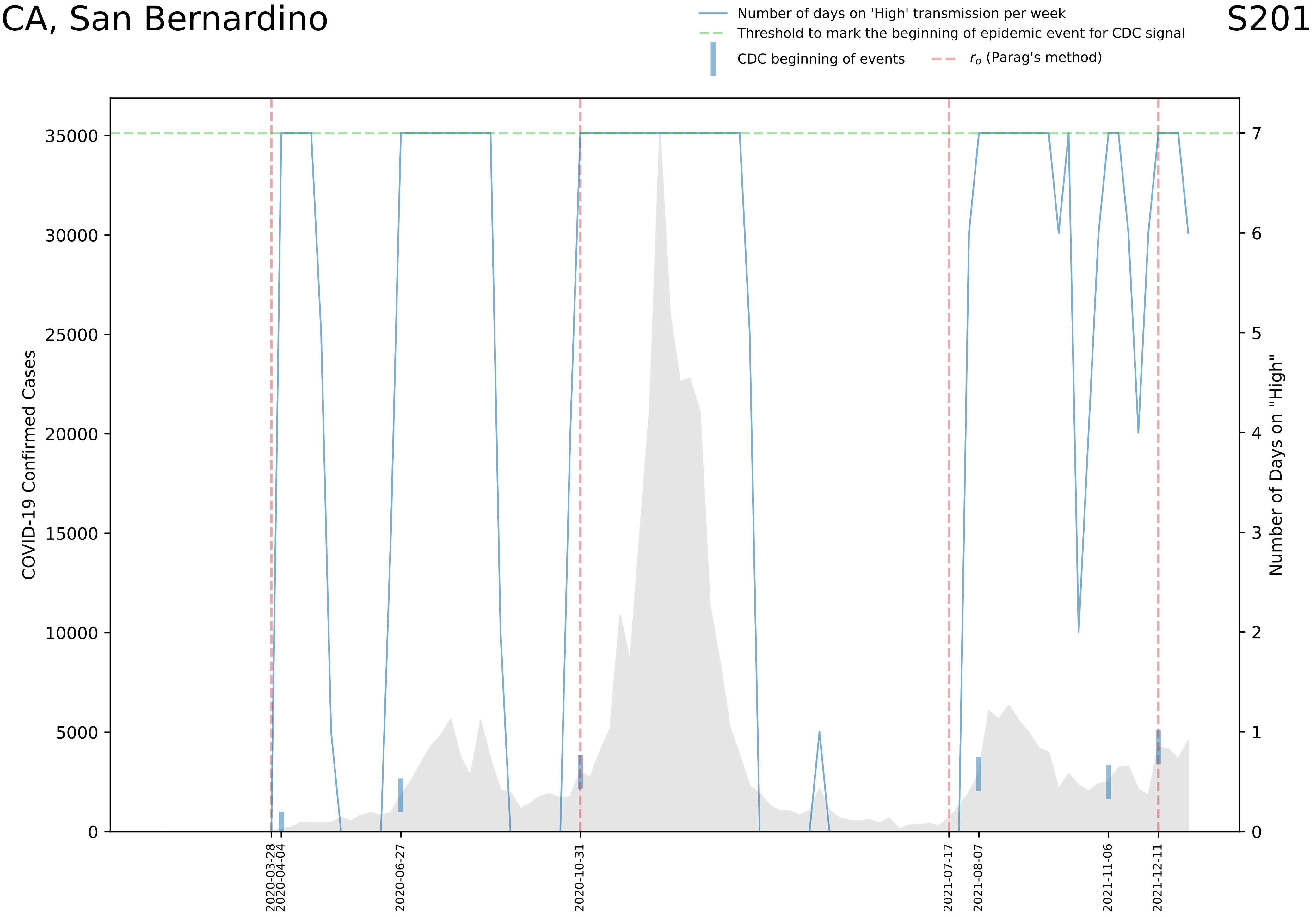

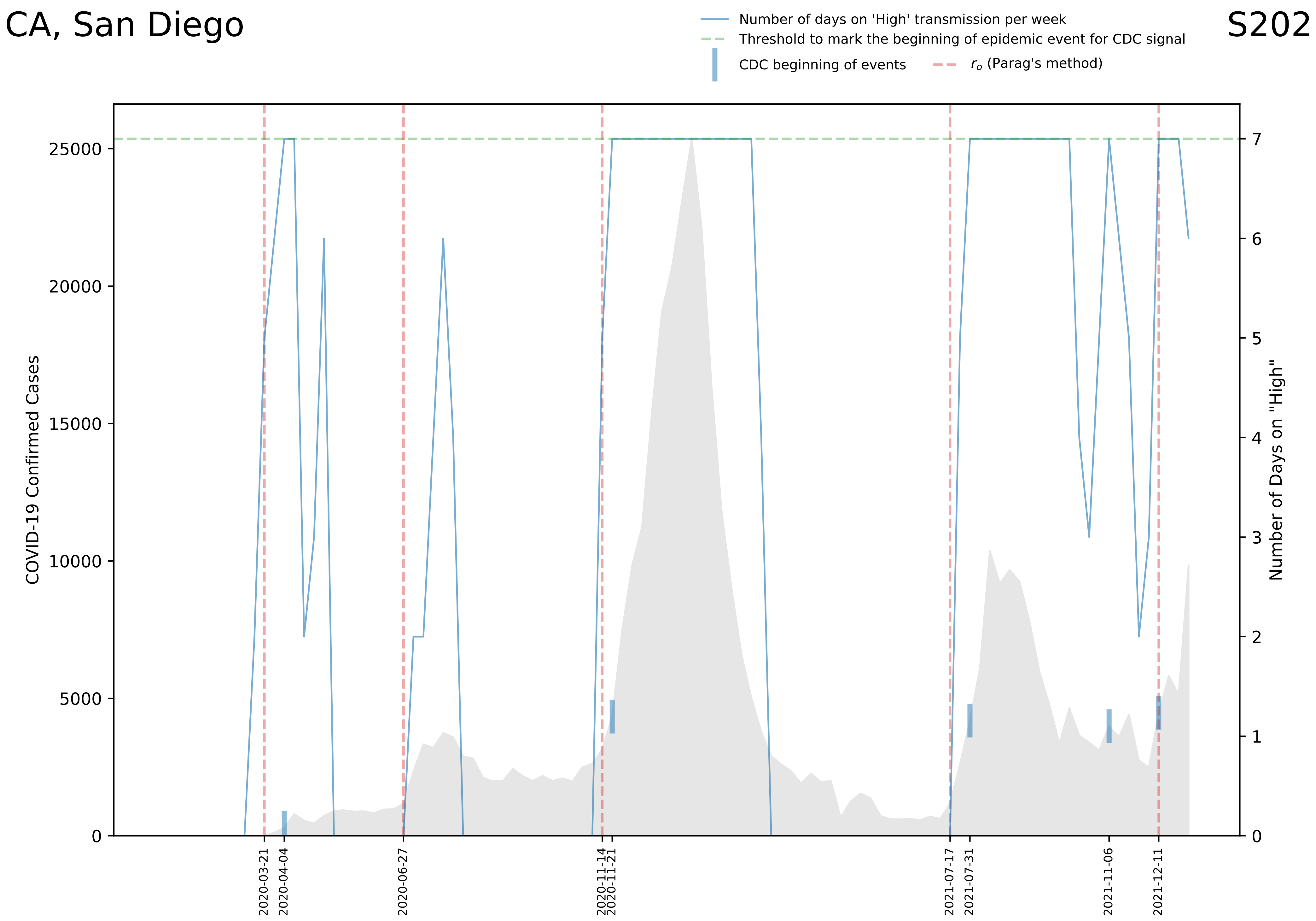

CA, San Diego
S202
Number of days on 'High' transmission per week
Threshold to mark the beginning of epidemic event for CDC signal
CDC beginning of events
r_o (Parag's method)
COVID-19 Confirmed Cases
Number of Days on "High"
0
5000
10000
15000
20000
25000
0
1
2
3
4
5
6
7
2020-03-21
2020-04-04
2020-06-27
2020-11-14
2020-11-21
2021-07-17
2021-07-31
2021-11-06
2021-12-11

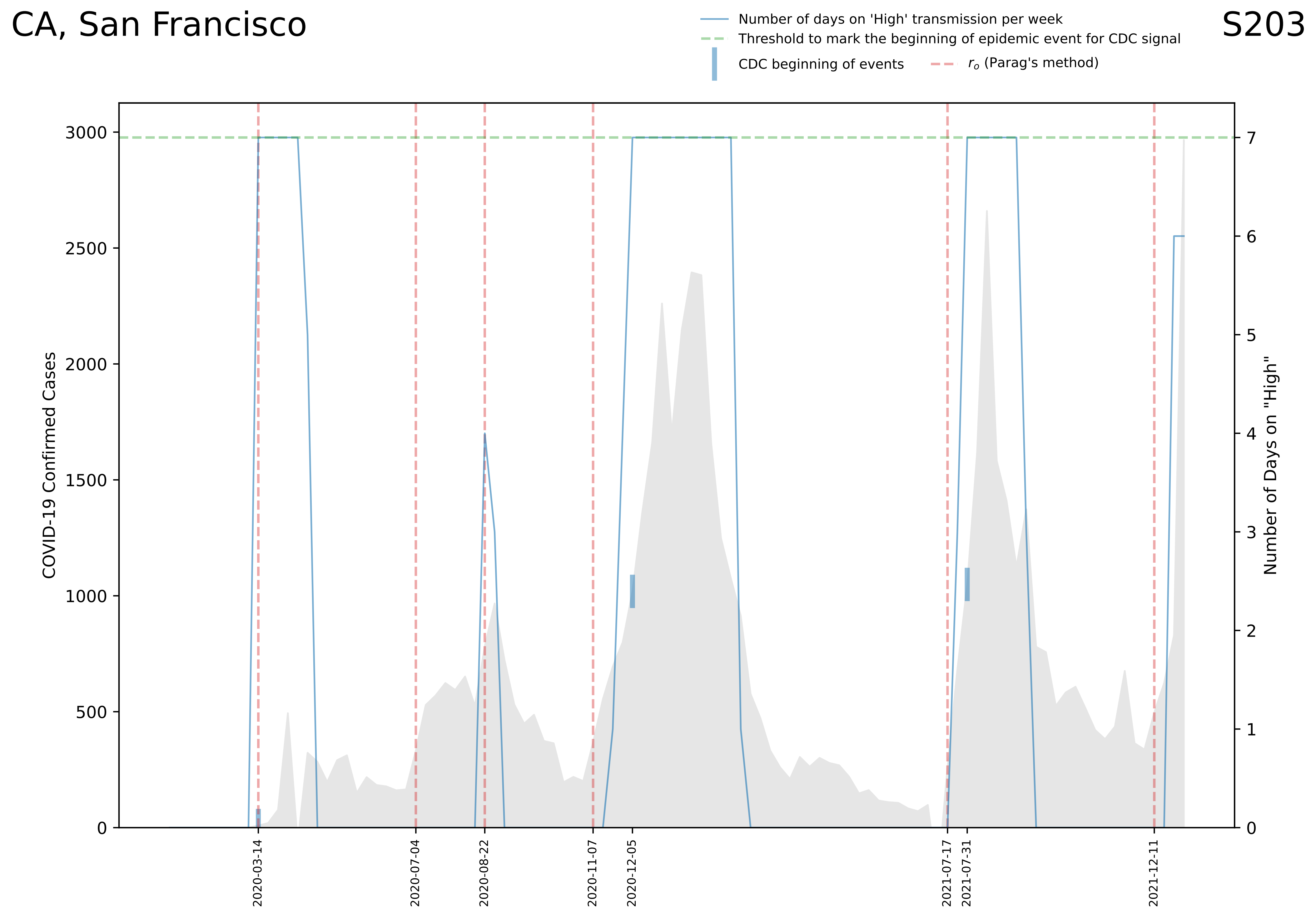

CA, San Francisco
S203
Number of days on 'High' transmission per week
Threshold to mark the beginning of epidemic event for CDC signal
CDC beginning of events
r_o (Parag's method)
COVID-19 Confirmed Cases
Number of Days on "High"
0
500
1000
1500
2000
2500
3000
0
1
2
3
4
5
6
7
2020-03-14
2020-07-04
2020-08-22
2020-11-07
2020-12-05
2021-07-17
2021-07-31
2021-12-11

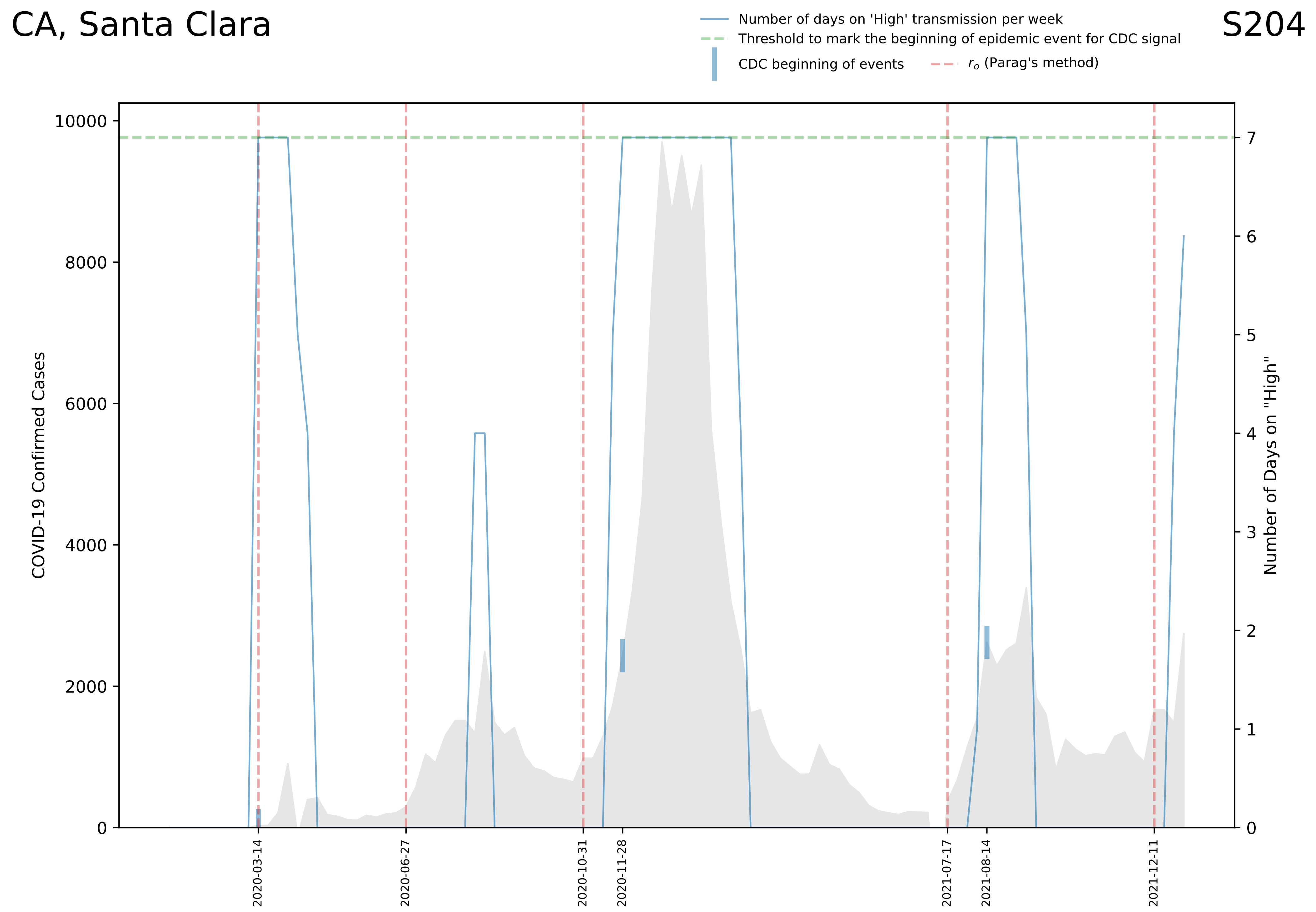

CA, Santa Clara
S204
Number of days on 'High' transmission per week
Threshold to mark the beginning of epidemic event for CDC signal
CDC beginning of events
r_o (Parag's method)
COVID-19 Confirmed Cases
Number of Days on "High"
0
2000
4000
6000
8000
10000
0
1
2
3
4
5
6
7
2020-03-14
2020-06-27
2020-10-31
2020-11-28
2021-07-17
2021-08-14
2021-12-11

CO, Adams

S205

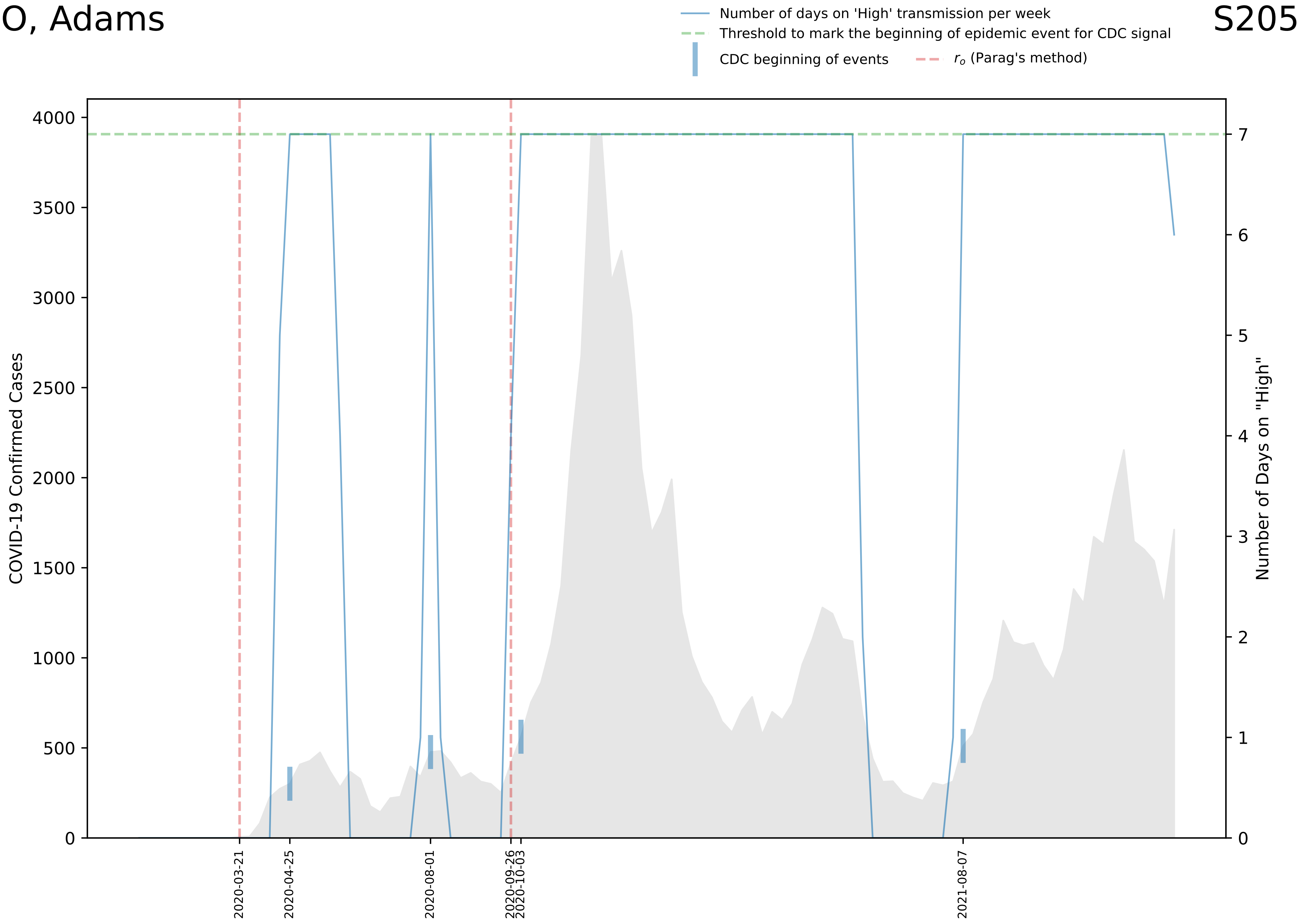

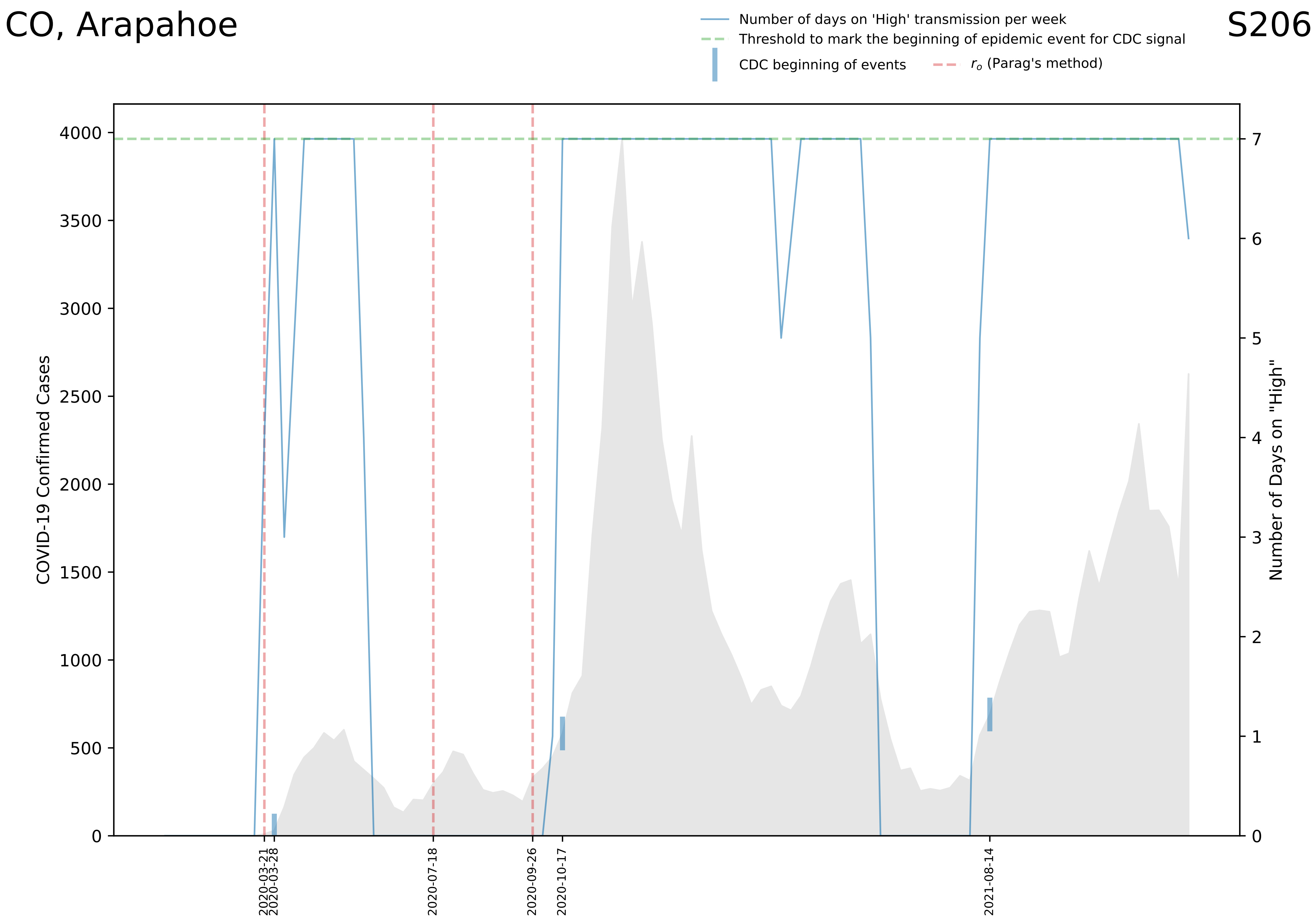

CO, Arapahoe
S206
Number of days on 'High' transmission per week
Threshold to mark the beginning of epidemic event for CDC signal
CDC beginning of events
r_o (Parag's method)
COVID-19 Confirmed Cases
Number of Days on "High"
0
500
1000
1500
2000
2500
3000
3500
4000
0
1
2
3
4
5
6
7
2020-03-21
2020-03-28
2020-07-18
2020-09-26
2020-10-17
2021-08-14

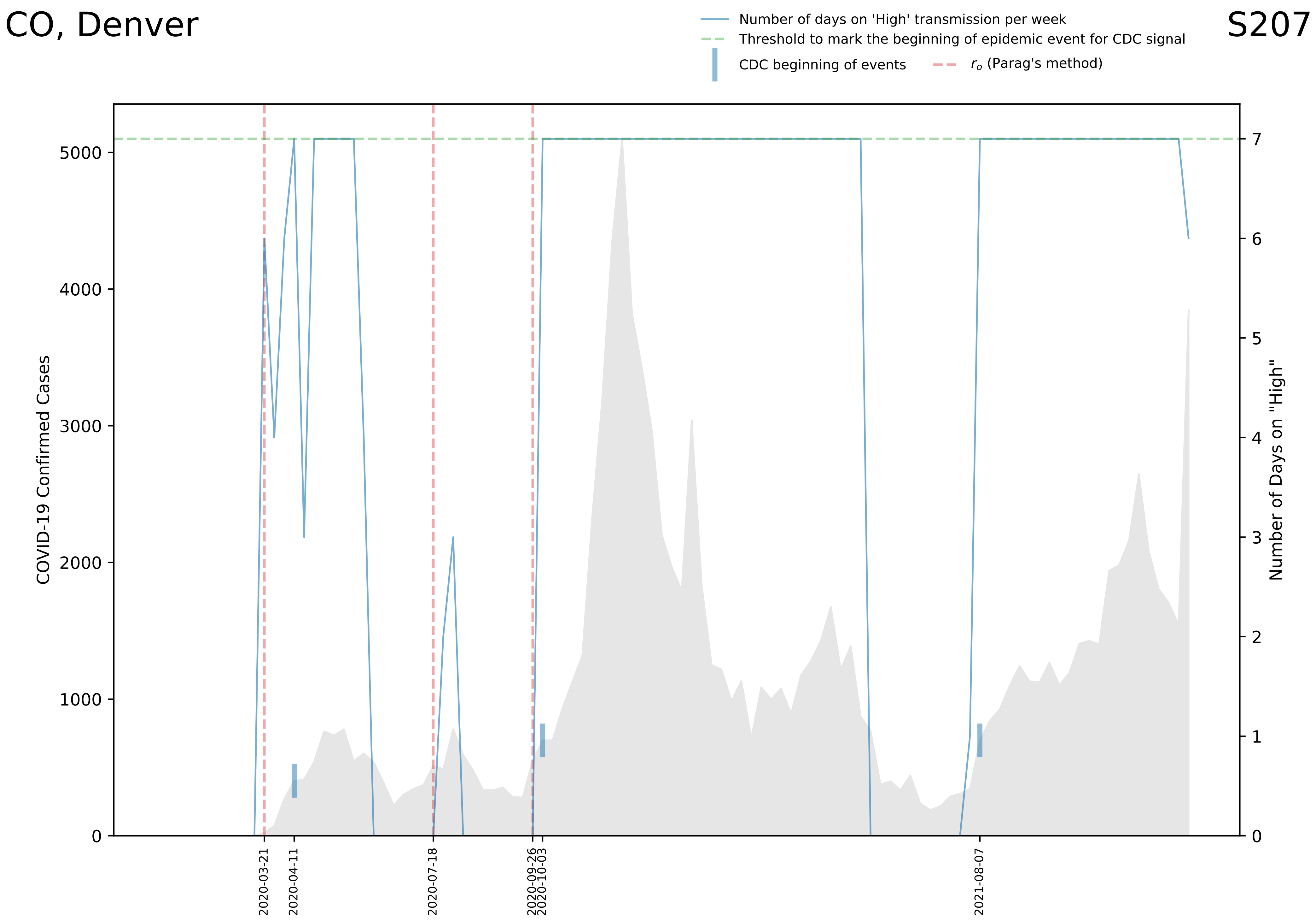
CO, Denver
S207
Number of days on 'High' transmission per week
Threshold to mark the beginning of epidemic event for CDC signal
CDC beginning of events
r_o (Parag's method)
COVID-19 Confirmed Cases
Number of Days on "High"
0
1000
2000
3000
4000
5000
0
1
2
3
4
5
6
7
2020-03-21
2020-04-11
2020-07-18
2020-09-26
2020-10-03
2021-08-07

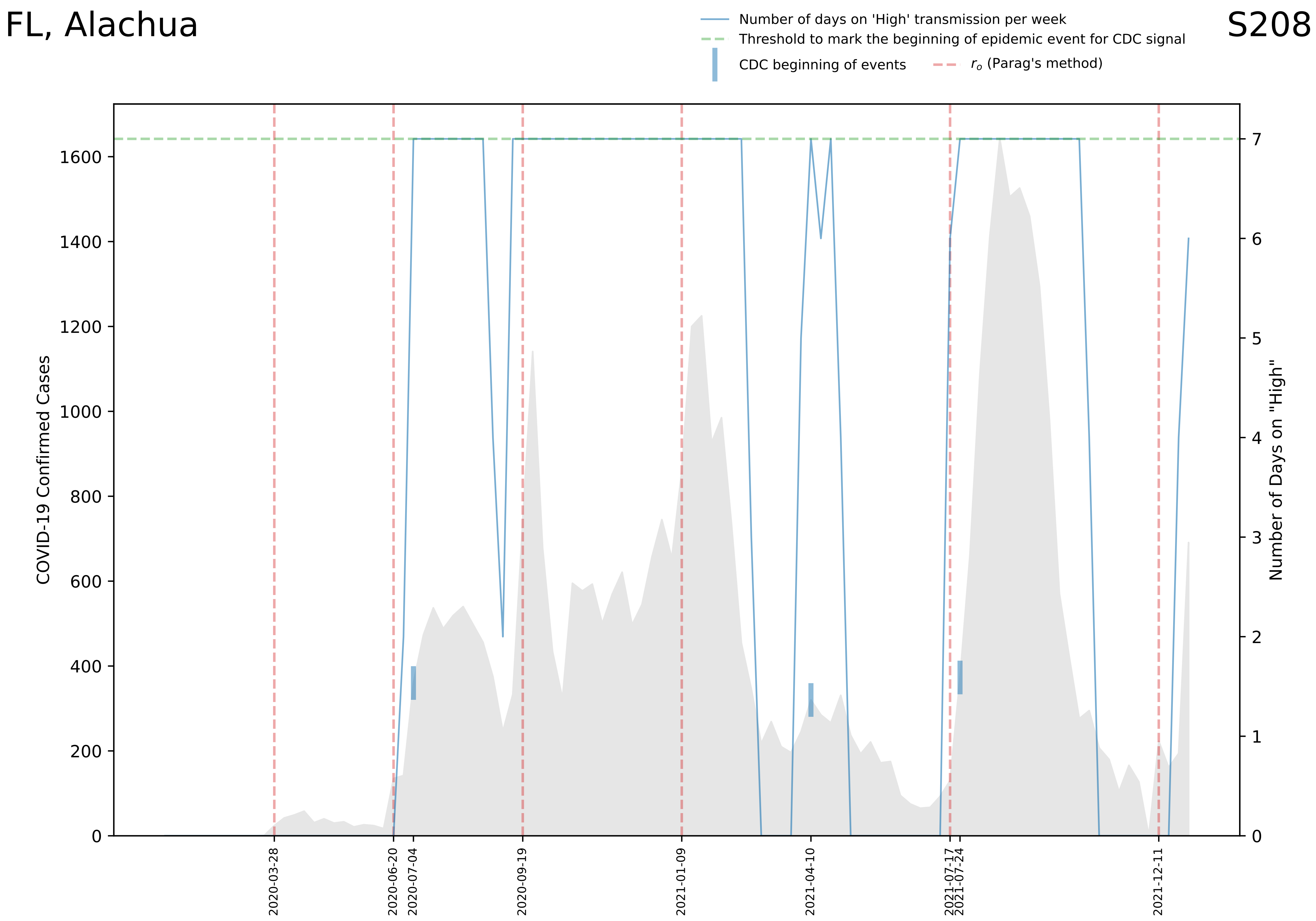

FL, Alachua
S208
Number of days on 'High' transmission per week
Threshold to mark the beginning of epidemic event for CDC signal
CDC beginning of events
r_o (Parag's method)
COVID-19 Confirmed Cases
Number of Days on "High"
0
200
400
600
800
1000
1200
1400
1600
0
1
2
3
4
5
6
7
2020-03-28
2020-06-20
2020-07-04
2020-09-19
2021-01-09
2021-04-10
2021-07-17
2021-07-24
2021-12-11

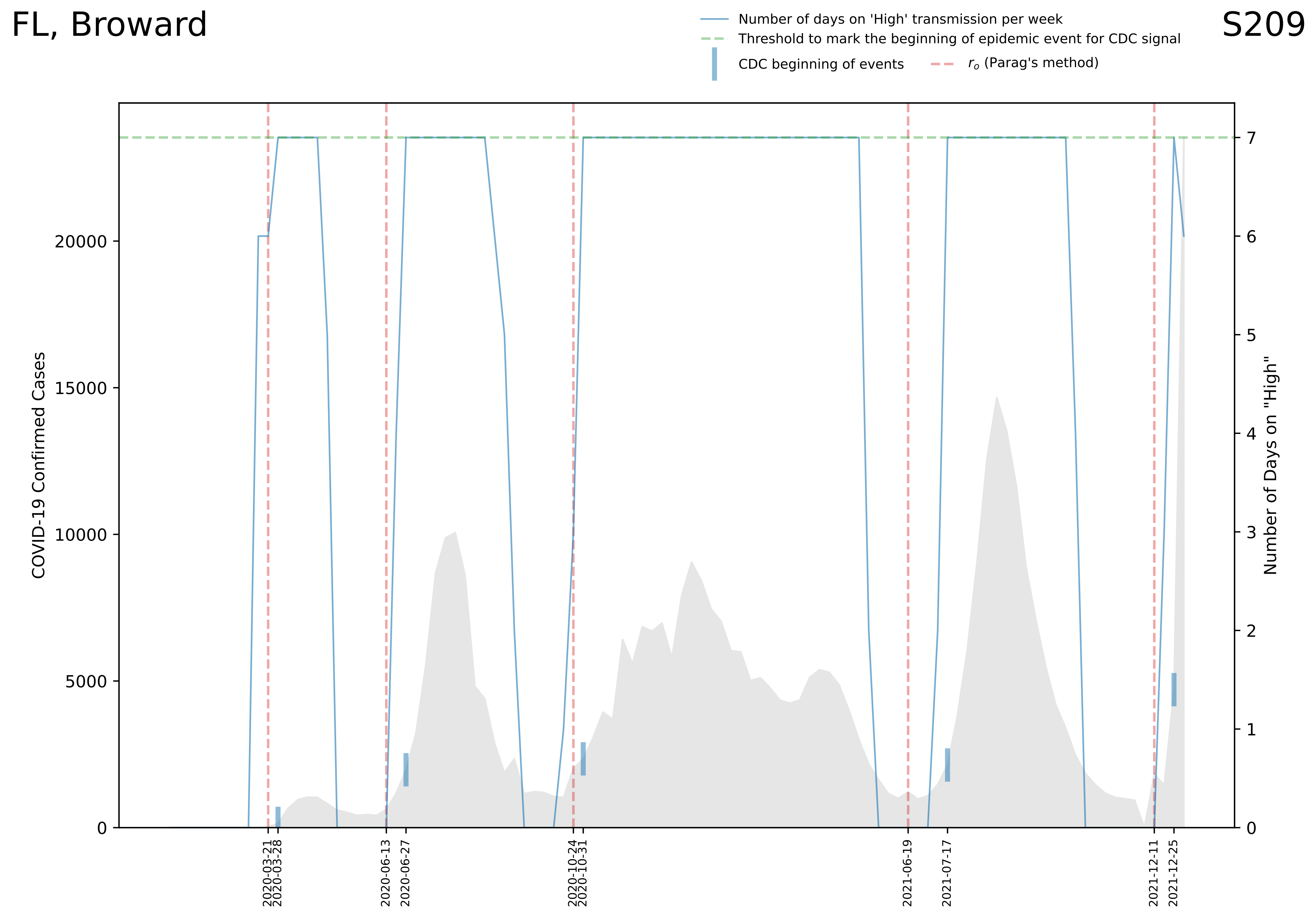

FL, Broward
S209
Number of days on 'High' transmission per week
Threshold to mark the beginning of epidemic event for CDC signal
CDC beginning of events
r_o (Parag's method)
COVID-19 Confirmed Cases
Number of Days on "High"
0
5000
10000
15000
20000
0
1
2
3
4
5
6
7
2020-03-21
2020-03-28
2020-06-13
2020-06-27
2020-10-24
2020-10-31
2021-06-19
2021-07-17
2021-12-11
2021-12-25

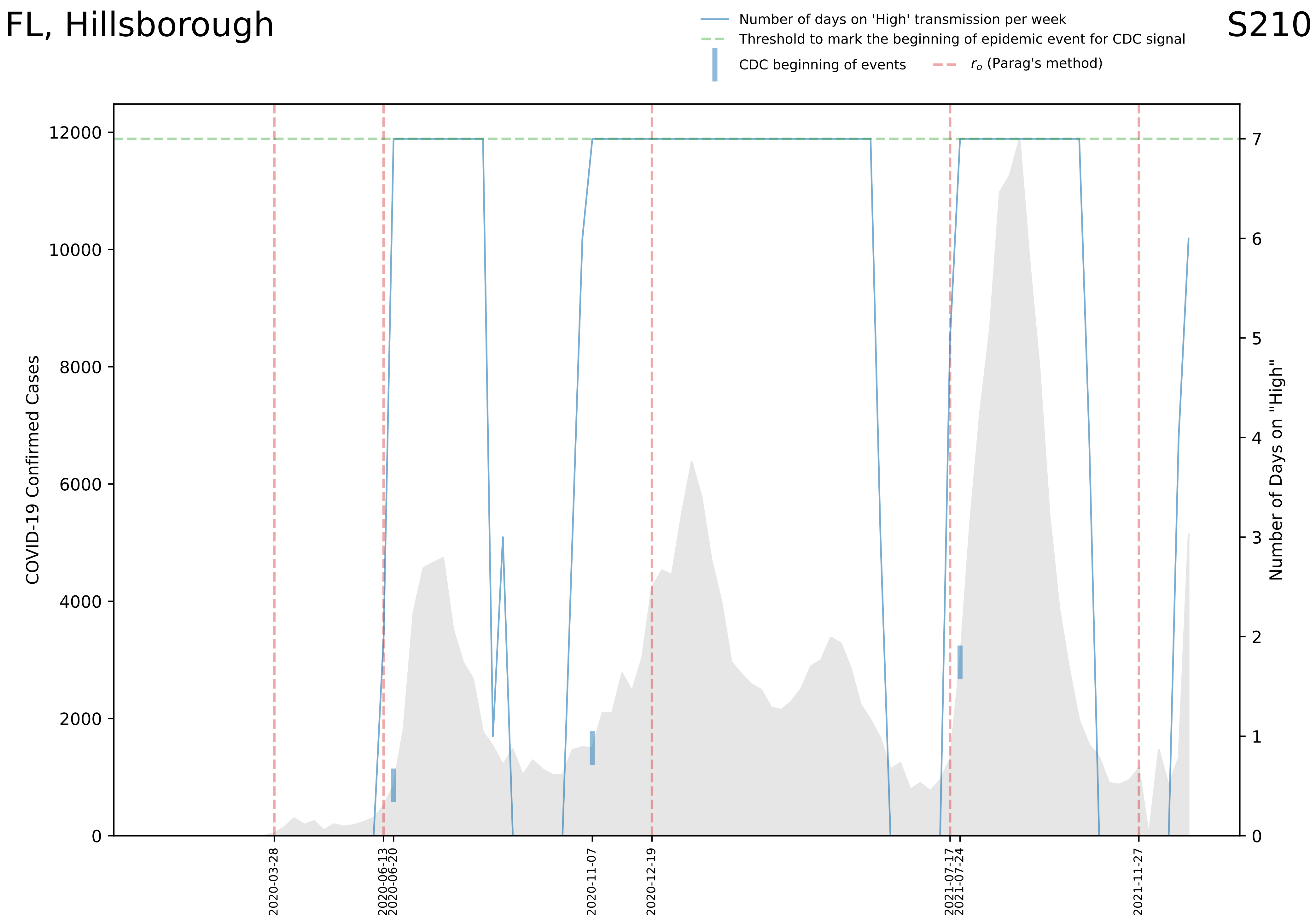

FL, Hillsborough
S210
Number of days on 'High' transmission per week
Threshold to mark the beginning of epidemic event for CDC signal
CDC beginning of events
r_o (Parag's method)
COVID-19 Confirmed Cases
Number of Days on "High"
0
2000
4000
6000
8000
10000
12000
0
1
2
3
4
5
6
7
2020-03-28
2020-06-13
2020-06-20
2020-11-07
2020-12-19
2021-07-17
2021-07-24
2021-11-27

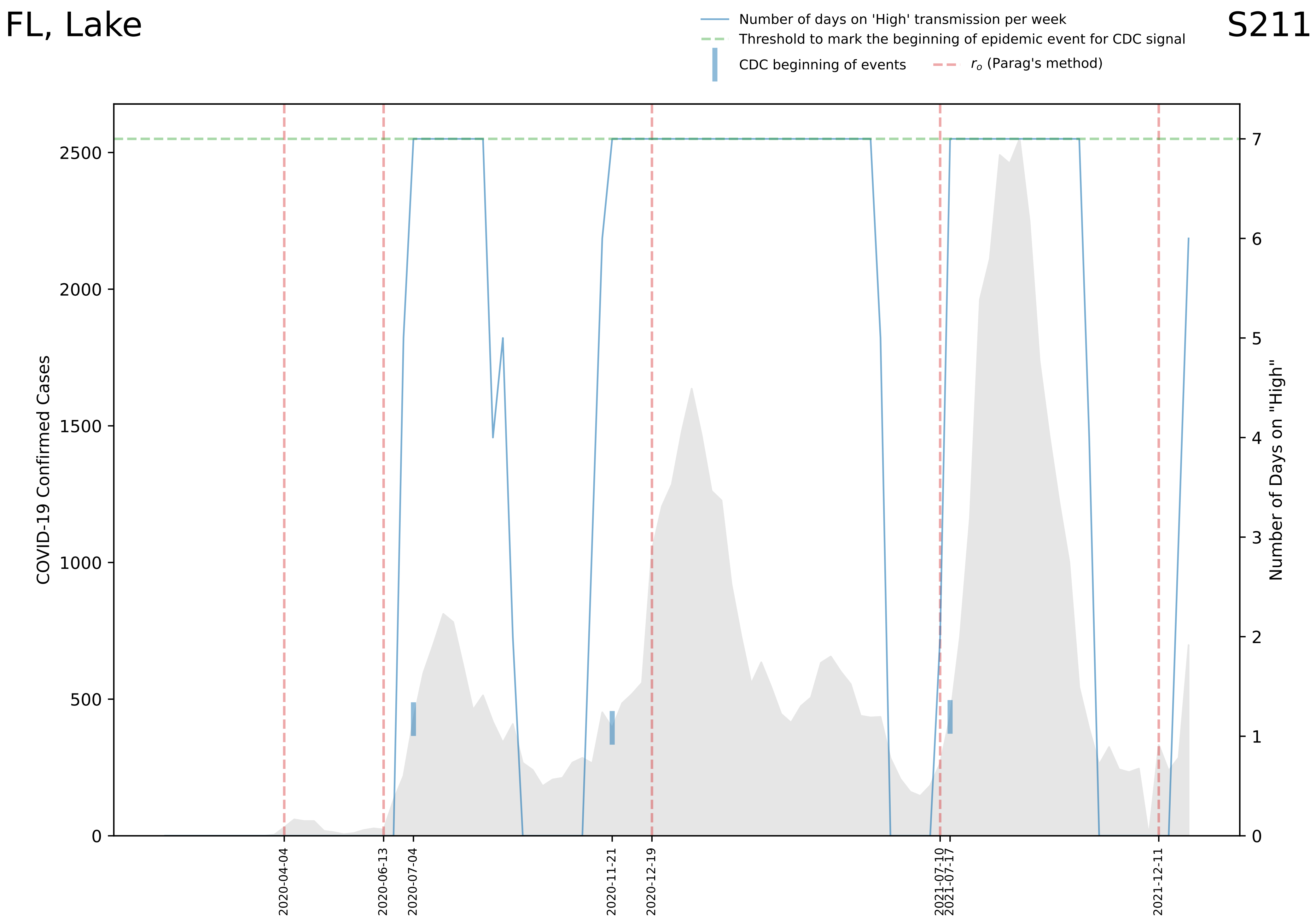

FL, Lake
S211
Number of days on 'High' transmission per week
Threshold to mark the beginning of epidemic event for CDC signal
CDC beginning of events
r_o (Parag's method)
COVID-19 Confirmed Cases
Number of Days on "High"
0
500
1000
1500
2000
2500
0
1
2
3
4
5
6
7
2020-04-04
2020-06-13
2020-07-04
2020-11-21
2020-12-19
2021-07-10
2021-07-17
2021-12-11

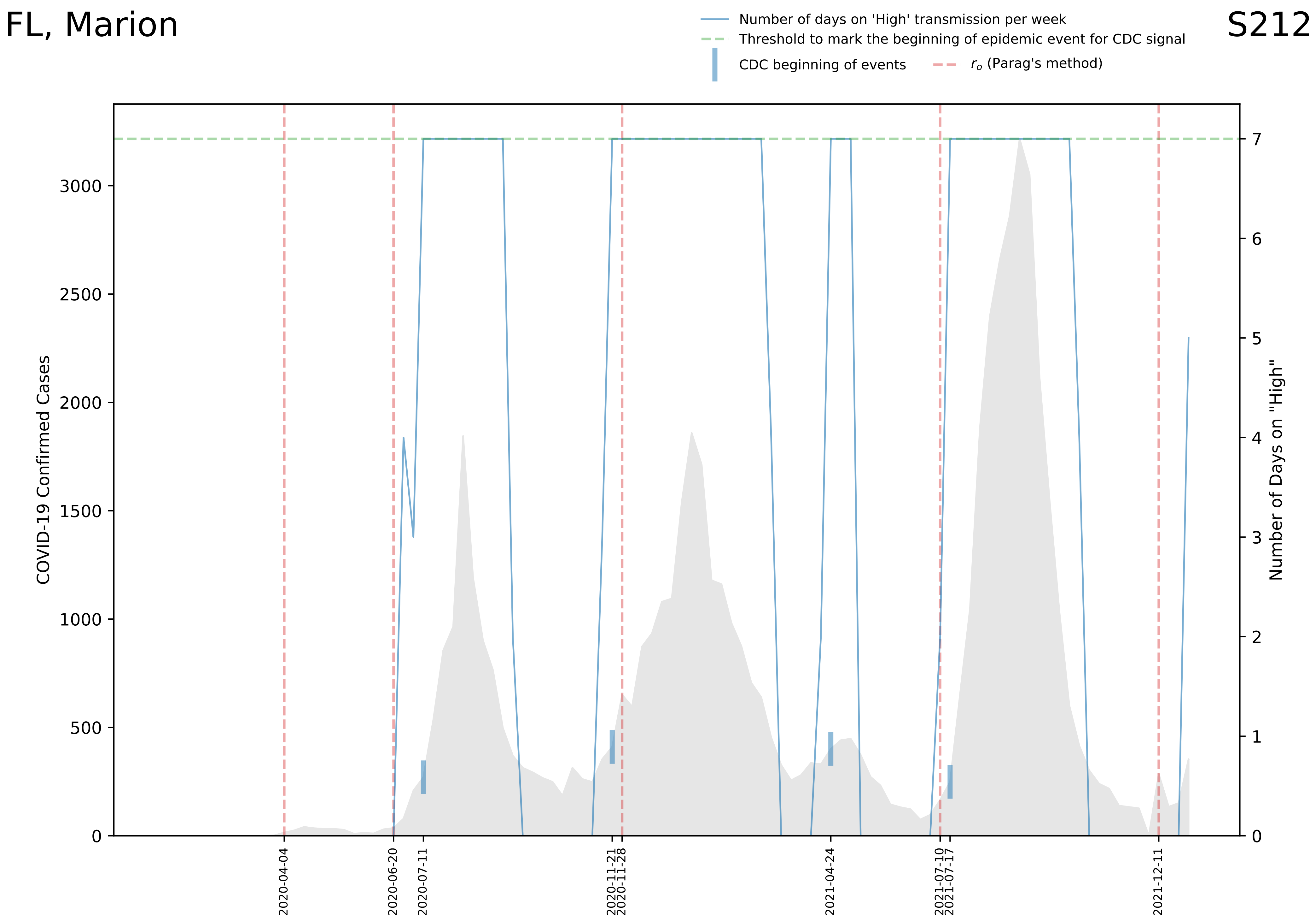

FL, Marion
S212
Number of days on 'High' transmission per week
Threshold to mark the beginning of epidemic event for CDC signal
CDC beginning of events
r_o (Parag's method)
COVID-19 Confirmed Cases
Number of Days on "High"
0
500
1000
1500
2000
2500
3000
0
1
2
3
4
5
6
7
2020-04-04
2020-06-20
2020-07-11
2020-11-21
2020-11-28
2021-04-24
2021-07-10
2021-07-17
2021-12-11

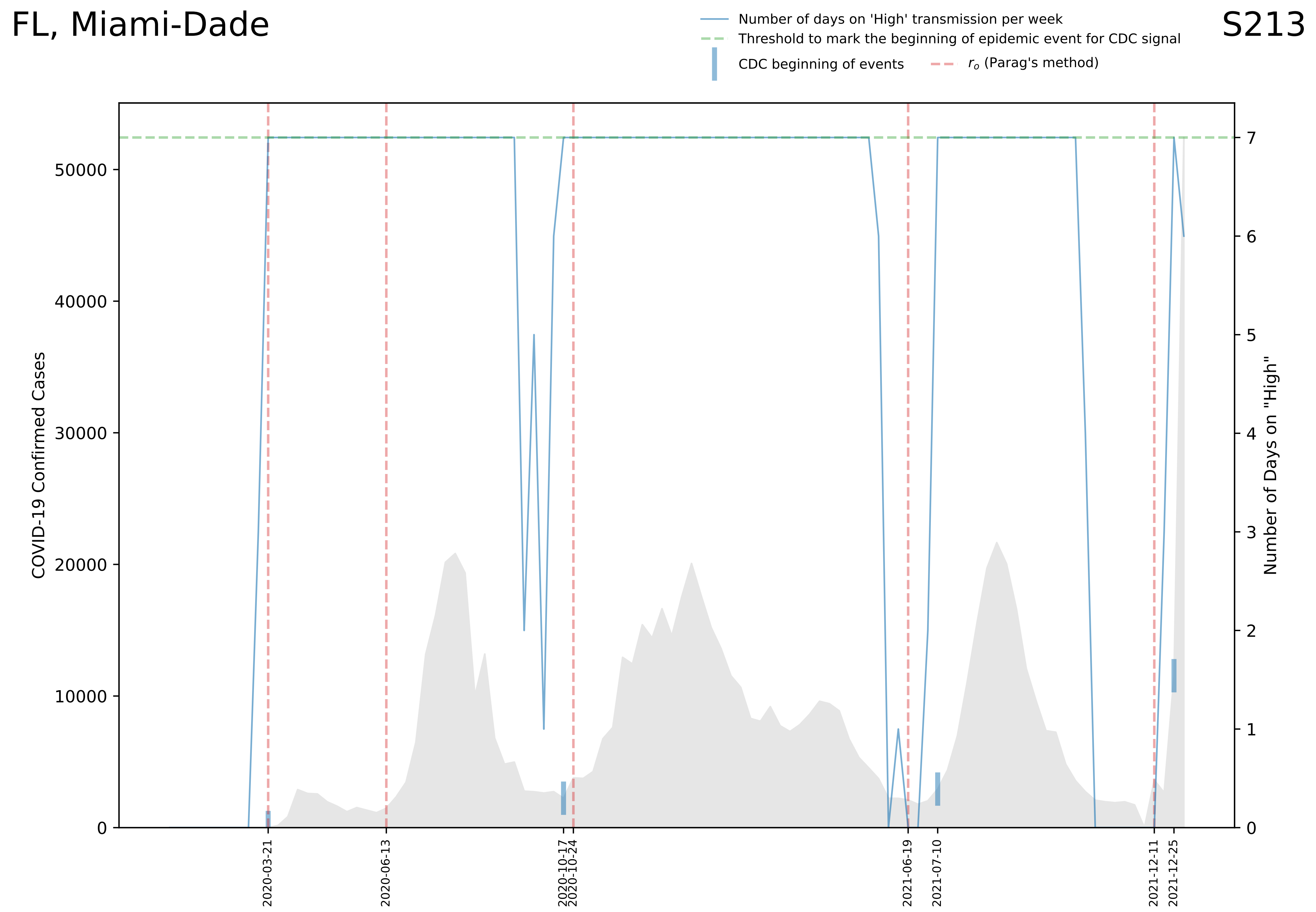

FL, Miami-Dade
S213
Number of days on 'High' transmission per week
Threshold to mark the beginning of epidemic event for CDC signal
CDC beginning of events
r_o (Parag's method)
COVID-19 Confirmed Cases
Number of Days on "High"
0
10000
20000
30000
40000
50000
0
1
2
3
4
5
6
7
2020-03-21
2020-06-13
2020-10-17
2020-10-24
2021-06-19
2021-07-10
2021-12-11
2021-12-25

FL, Orange

S214

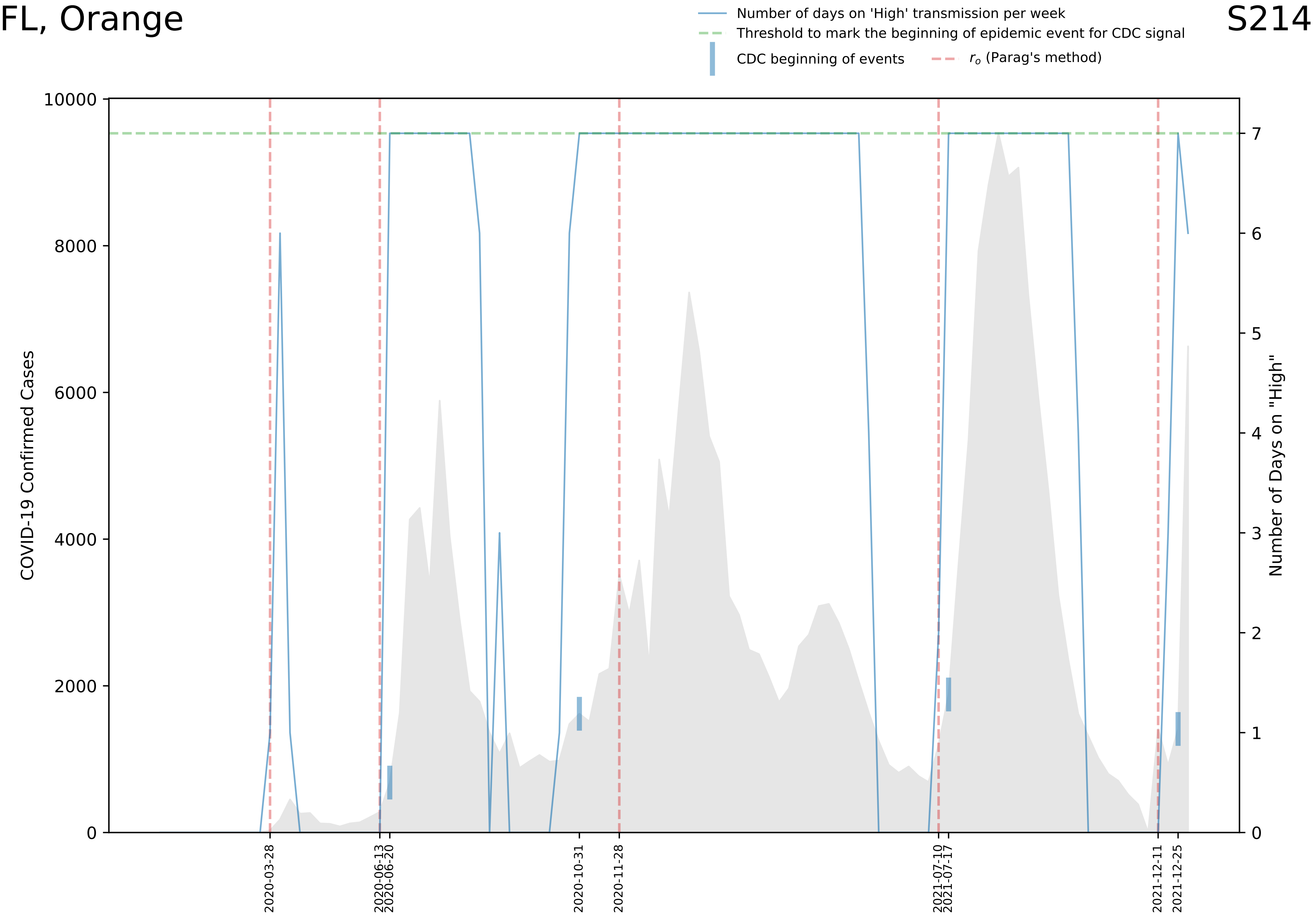

FL, Palm Beach

S215

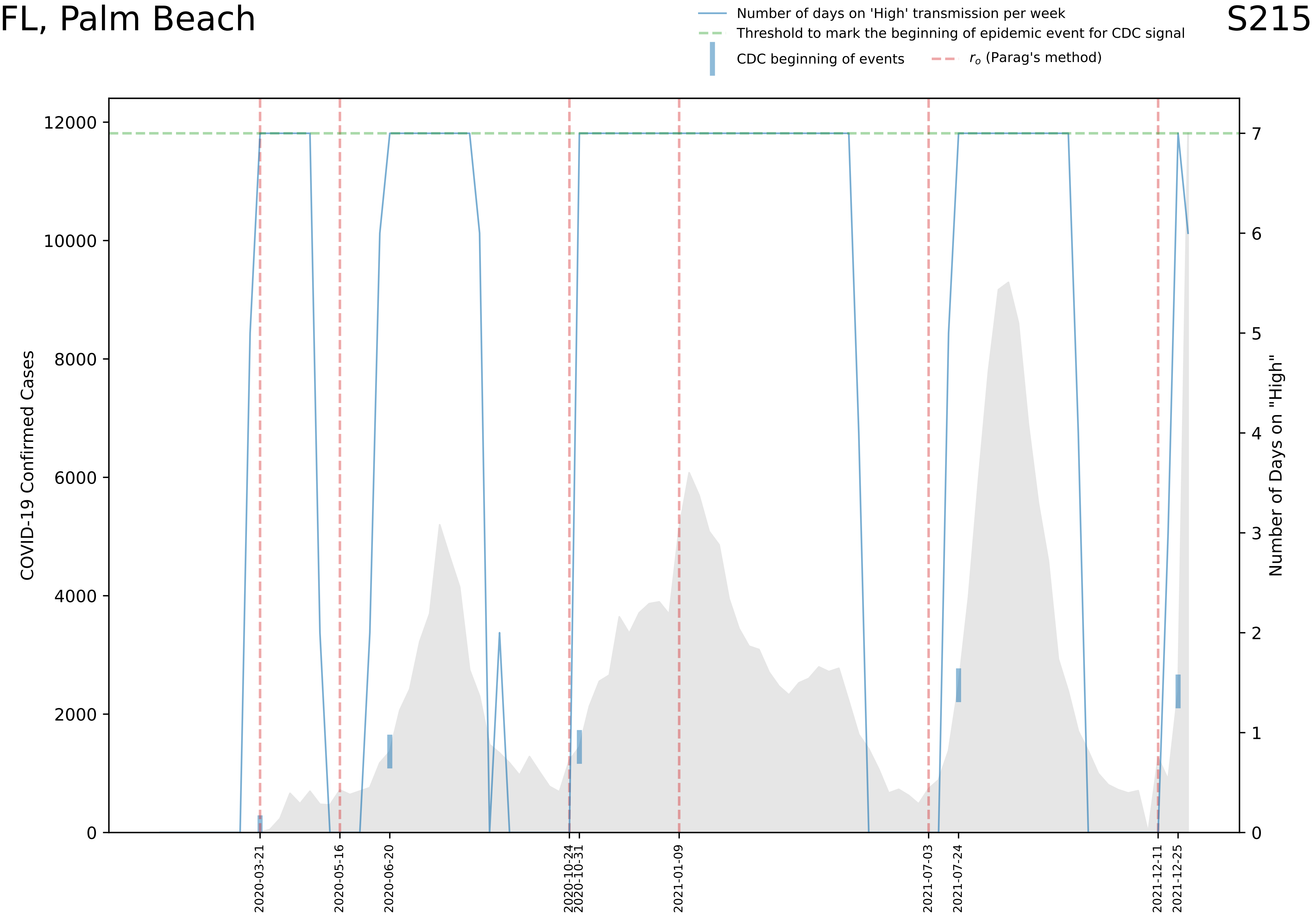

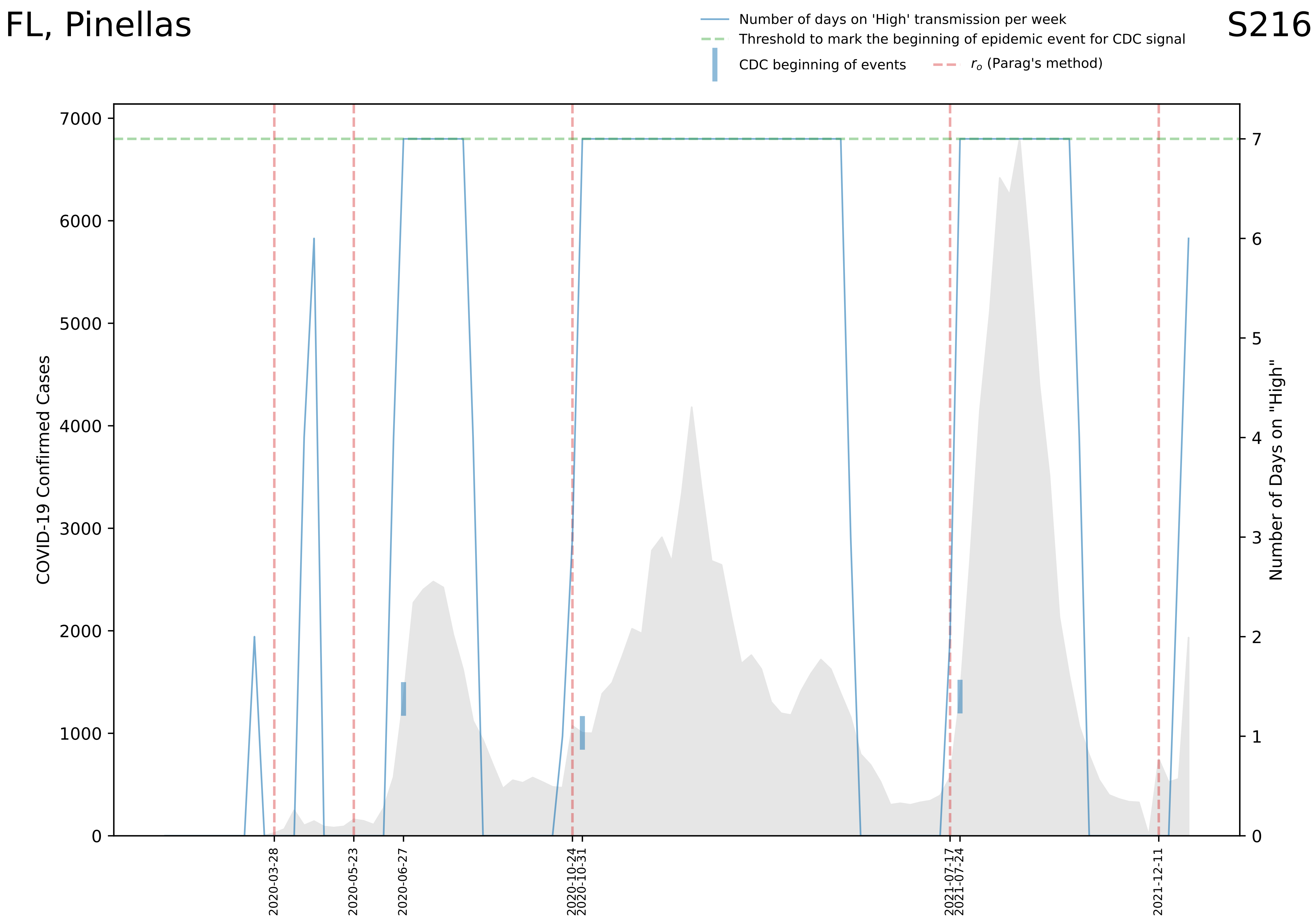

FL, Pinellas
S216
Number of days on 'High' transmission per week
Threshold to mark the beginning of epidemic event for CDC signal
CDC beginning of events
r_o (Parag's method)
COVID-19 Confirmed Cases
Number of Days on "High"
0
1000
2000
3000
4000
5000
6000
7000
0
1
2
3
4
5
6
7
2020-03-28
2020-05-23
2020-06-27
2020-10-24
2020-10-31
2021-07-17
2021-07-24
2021-12-11

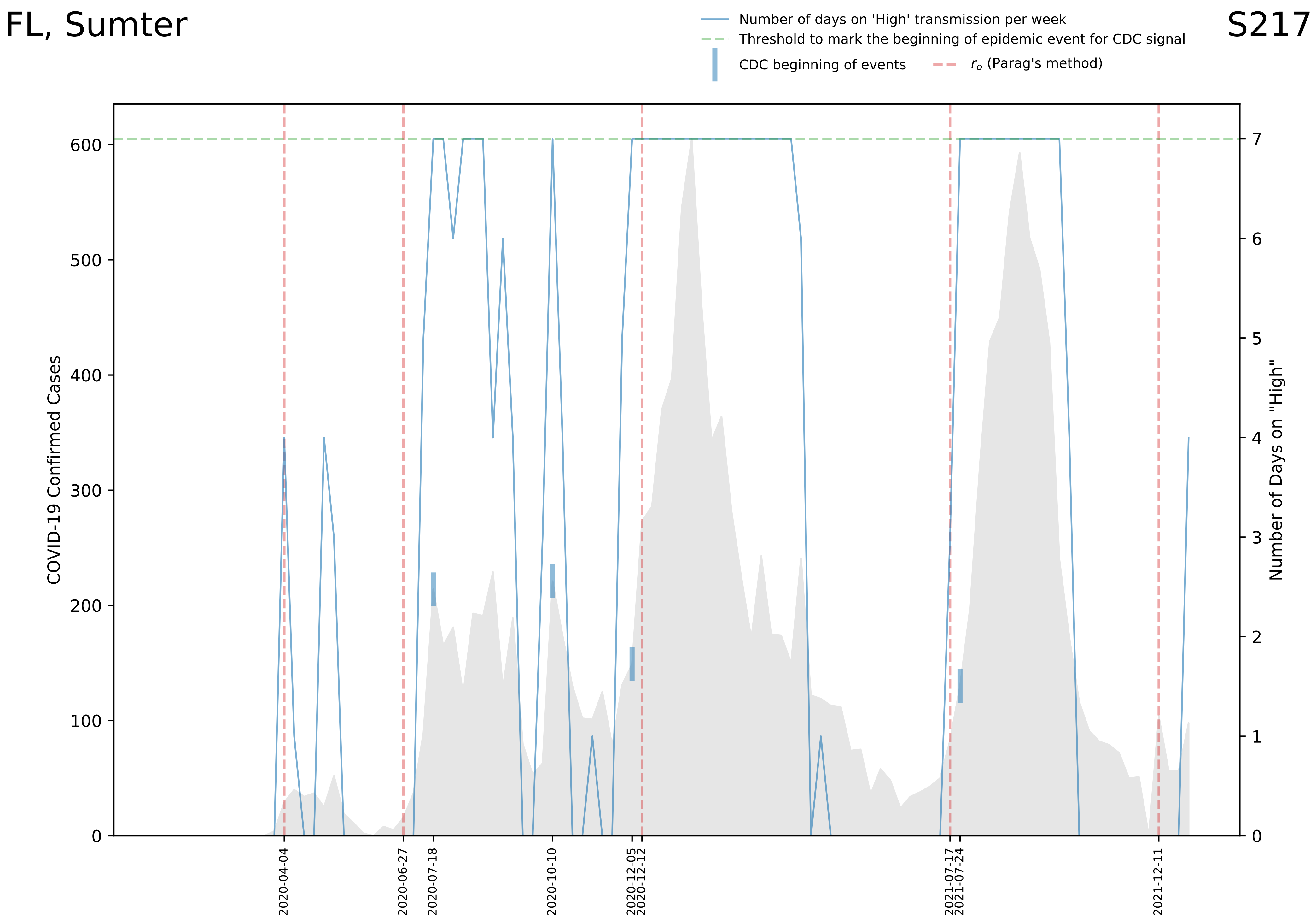

FL, Sumter
S217
Number of days on 'High' transmission per week
Threshold to mark the beginning of epidemic event for CDC signal
CDC beginning of events
r_o (Parag's method)
COVID-19 Confirmed Cases
Number of Days on "High"
0
100
200
300
400
500
600
1
2
3
4
5
6
7
2020-04-04
2020-06-27
2020-07-18
2020-10-10
2020-12-05
2020-12-12
2021-07-17
2021-07-24
2021-12-11

GA, DeKalb

S218

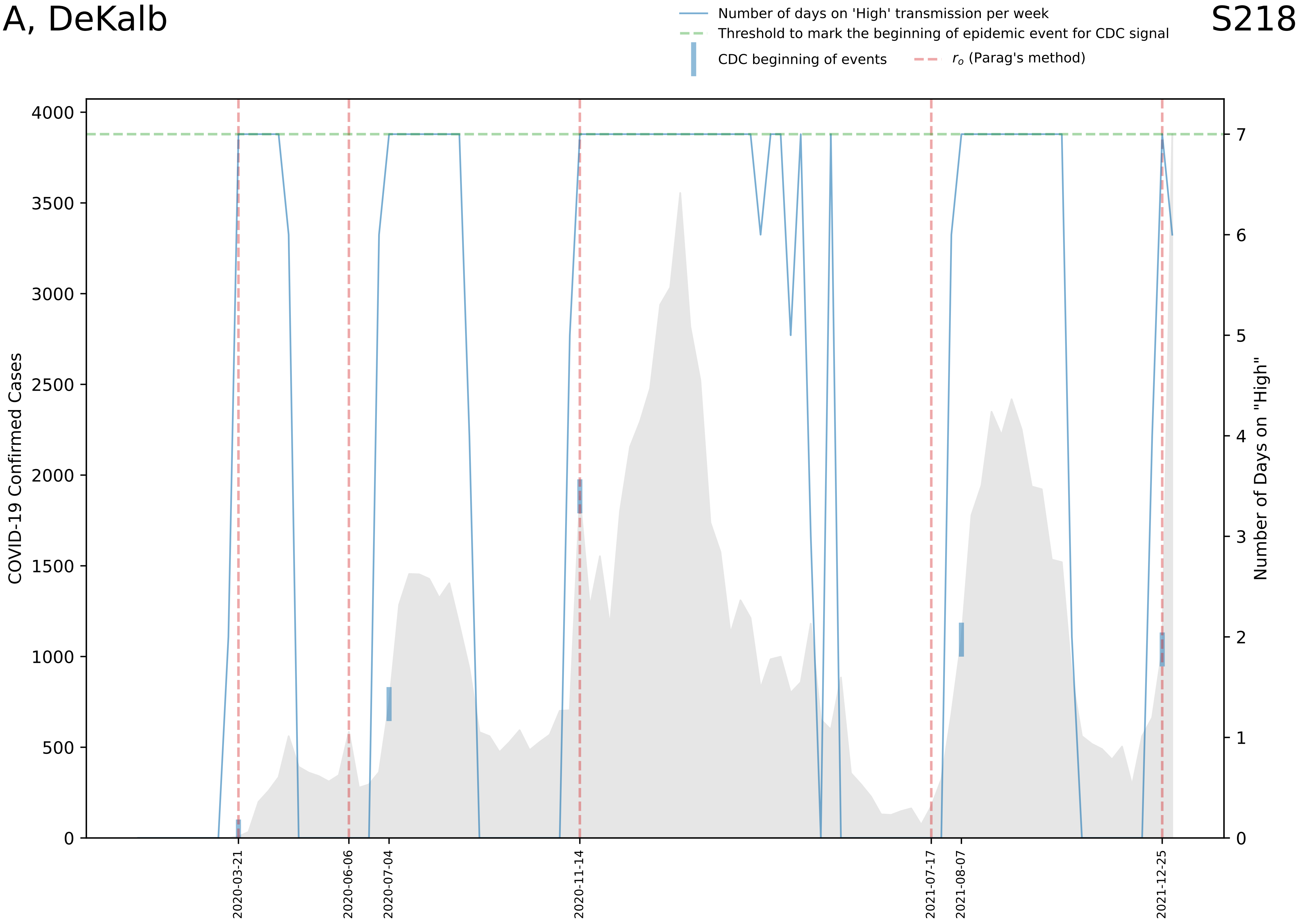

GA, Fulton

S219

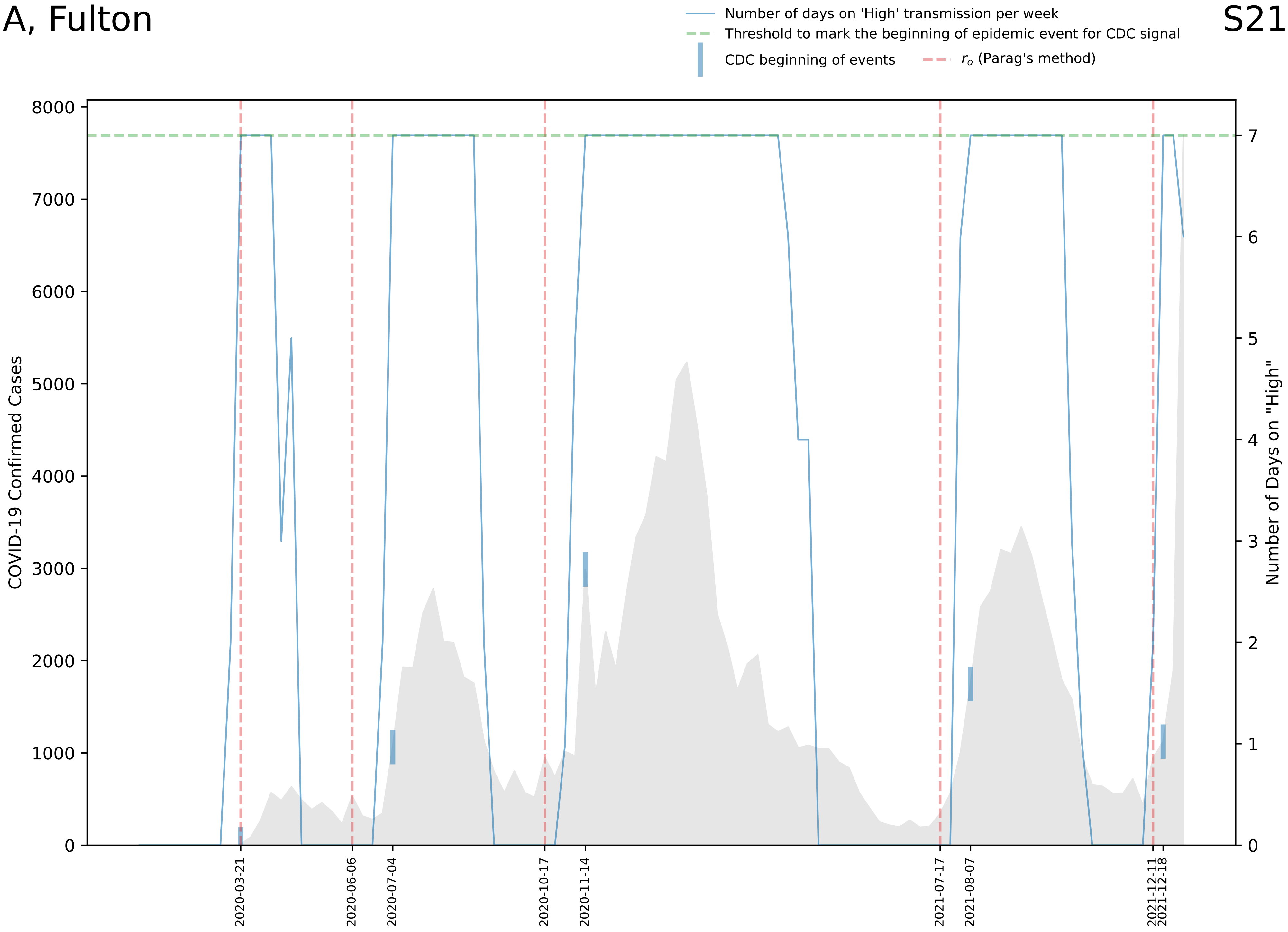

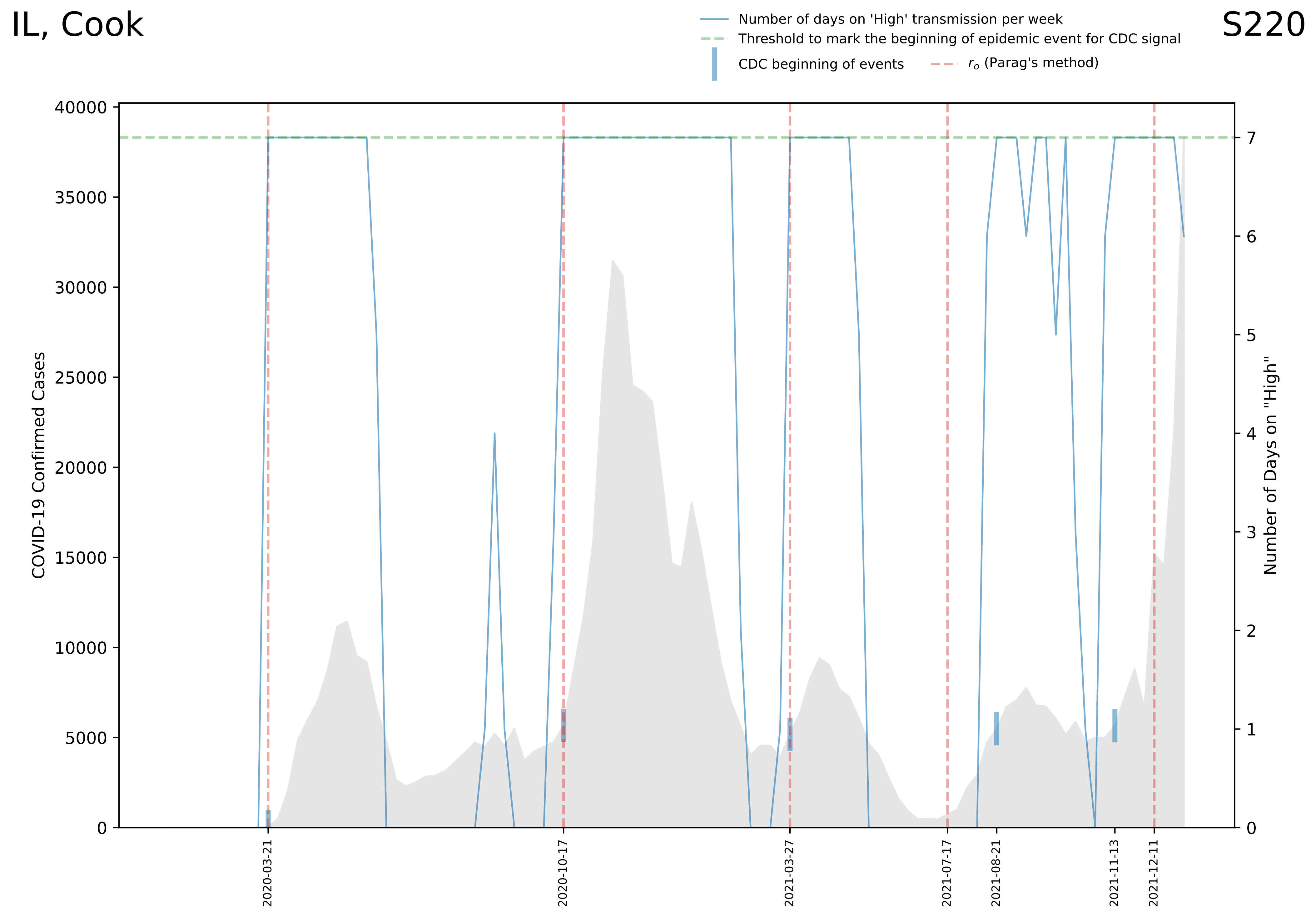

IL, Cook
S220
Number of days on 'High' transmission per week
Threshold to mark the beginning of epidemic event for CDC signal
CDC beginning of events
r_o (Parag's method)
COVID-19 Confirmed Cases
Number of Days on "High"
2020-03-21
2020-10-17
2021-03-27
2021-07-17
2021-08-21
2021-11-13
2021-12-11

IL, Peoria

S221

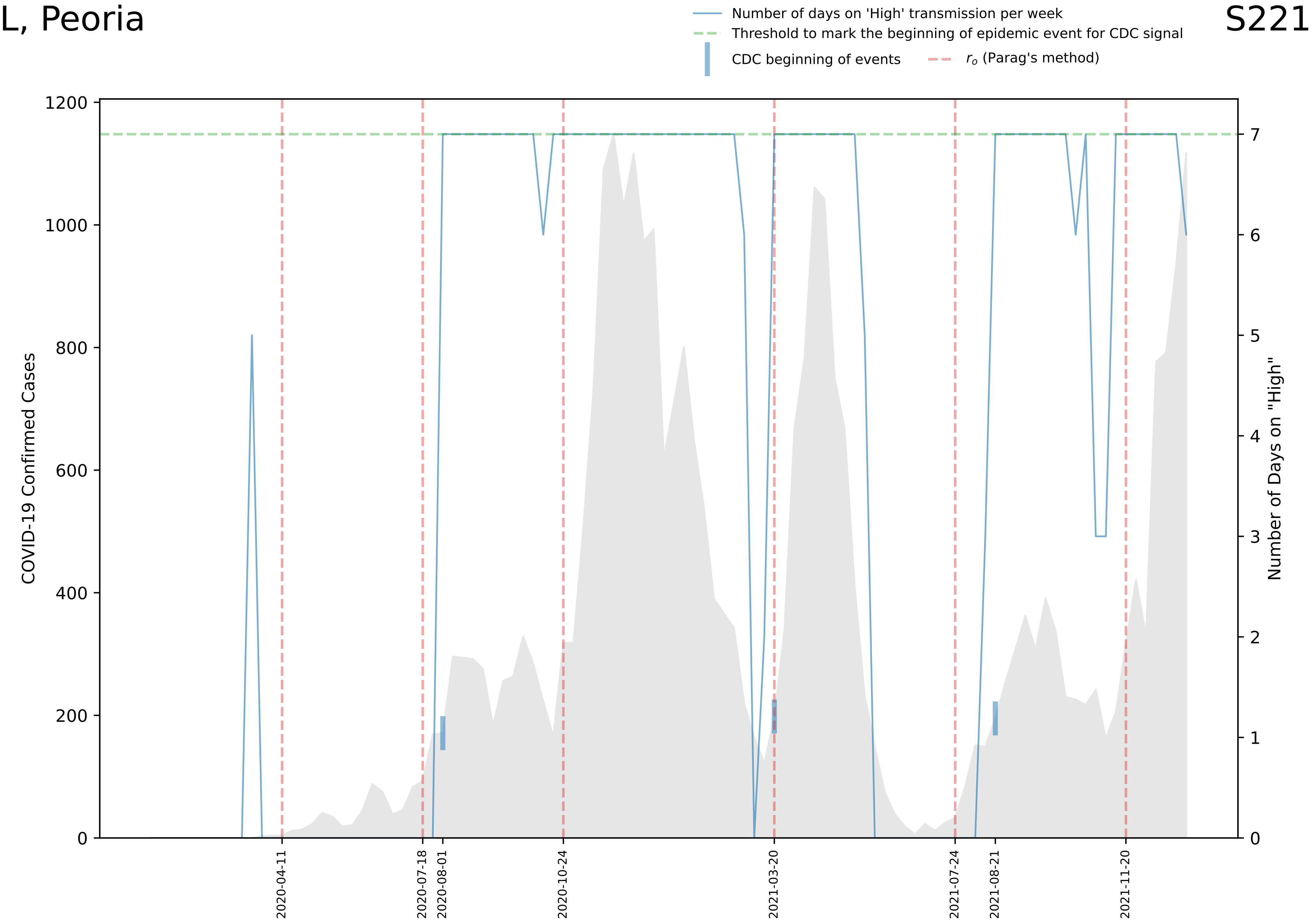

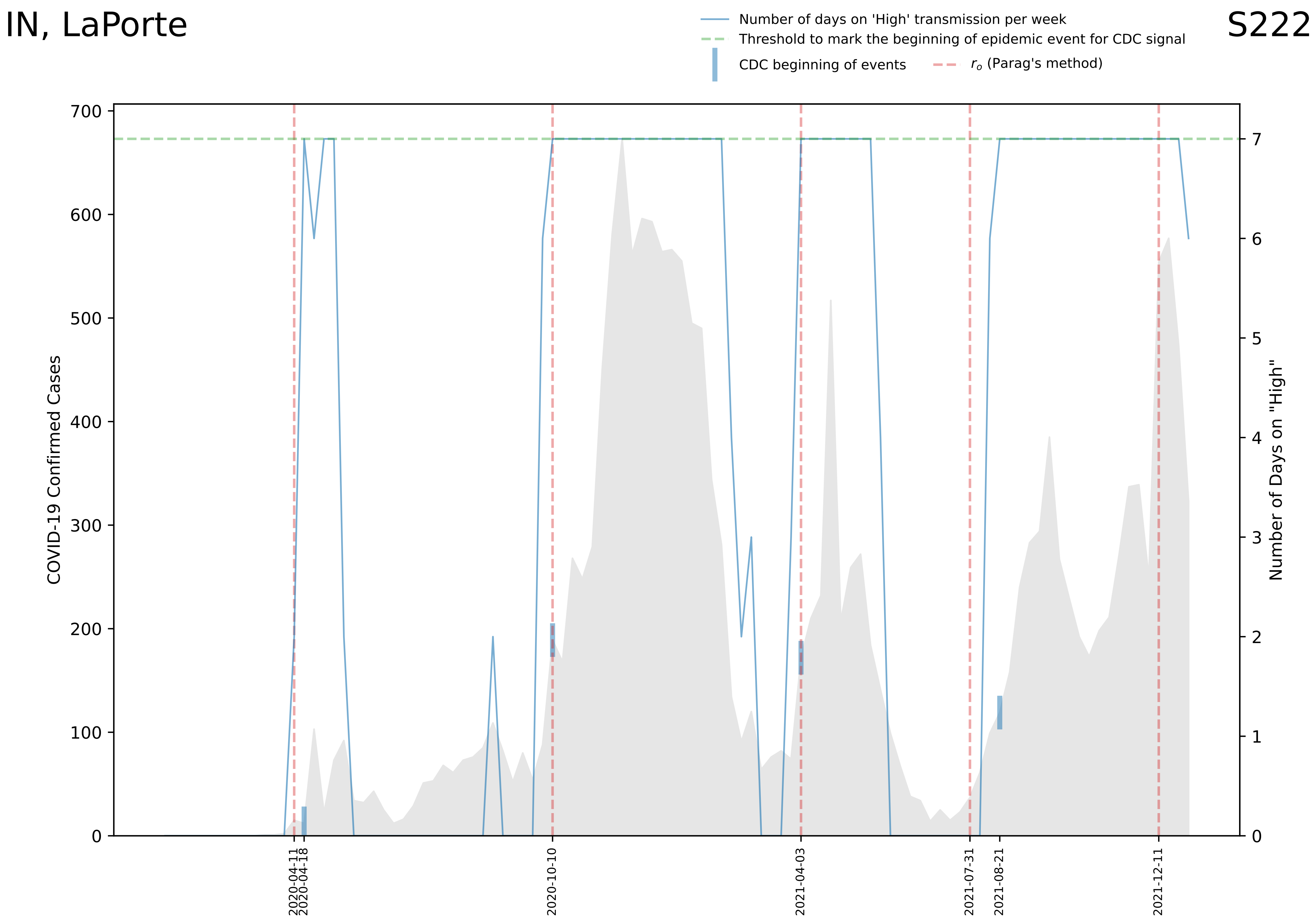
IN, LaPorte
S222
Number of days on 'High' transmission per week
Threshold to mark the beginning of epidemic event for CDC signal
CDC beginning of events
r_o (Parag's method)
COVID-19 Confirmed Cases
Number of Days on "High"
0
100
200
300
400
500
600
700
0
1
2
3
4
5
6
7
2020-04-11
2020-04-18
2020-10-10
2021-04-03
2021-07-31
2021-08-21
2021-12-11

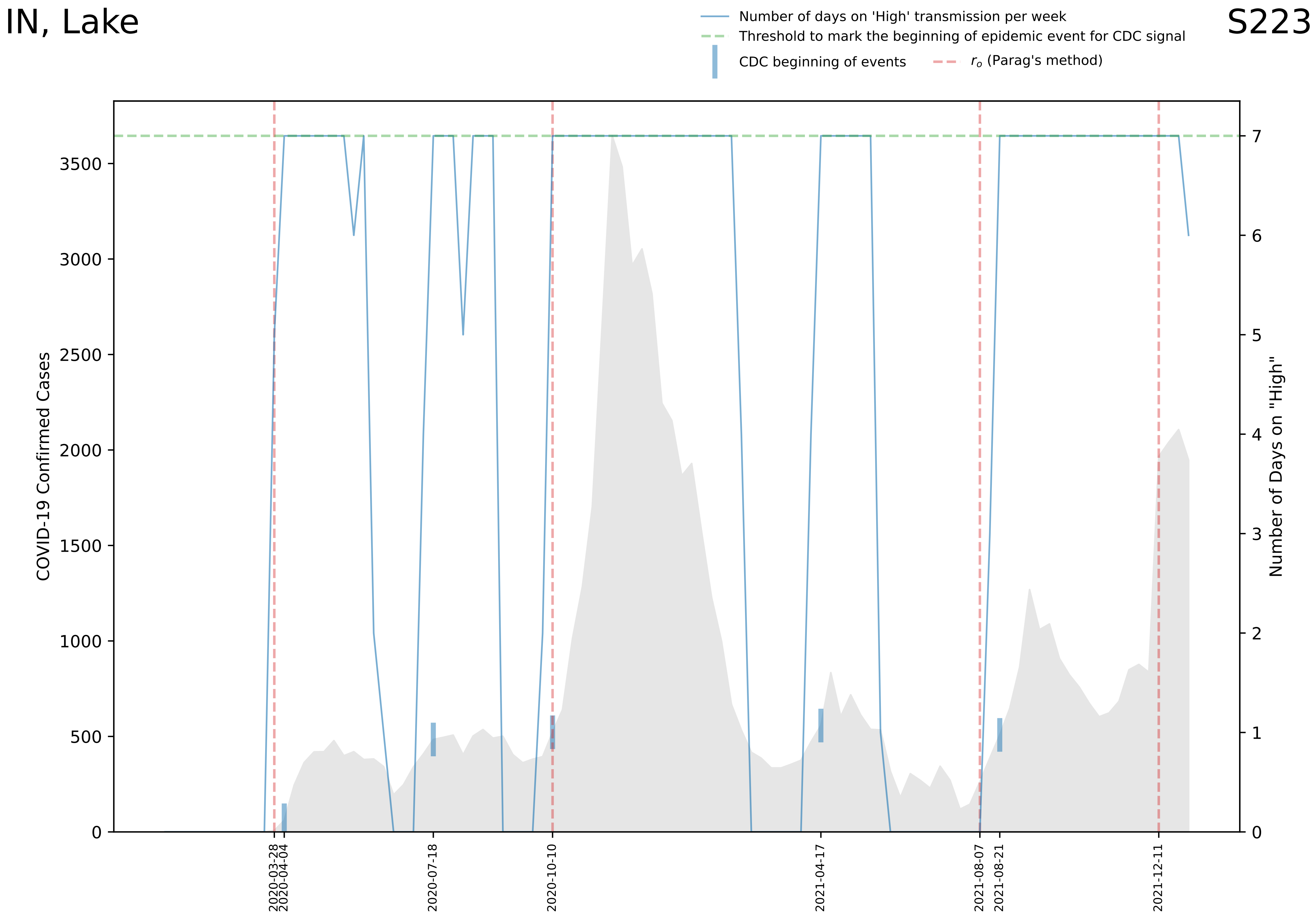

IN, Lake
S223
Number of days on 'High' transmission per week
Threshold to mark the beginning of epidemic event for CDC signal
CDC beginning of events
r_o (Parag's method)
COVID-19 Confirmed Cases
Number of Days on "High"
0
500
1000
1500
2000
2500
3000
3500
0
1
2
3
4
5
6
7
2020-03-28
2020-04-04
2020-07-18
2020-10-10
2021-04-17
2021-08-07
2021-08-21
2021-12-11

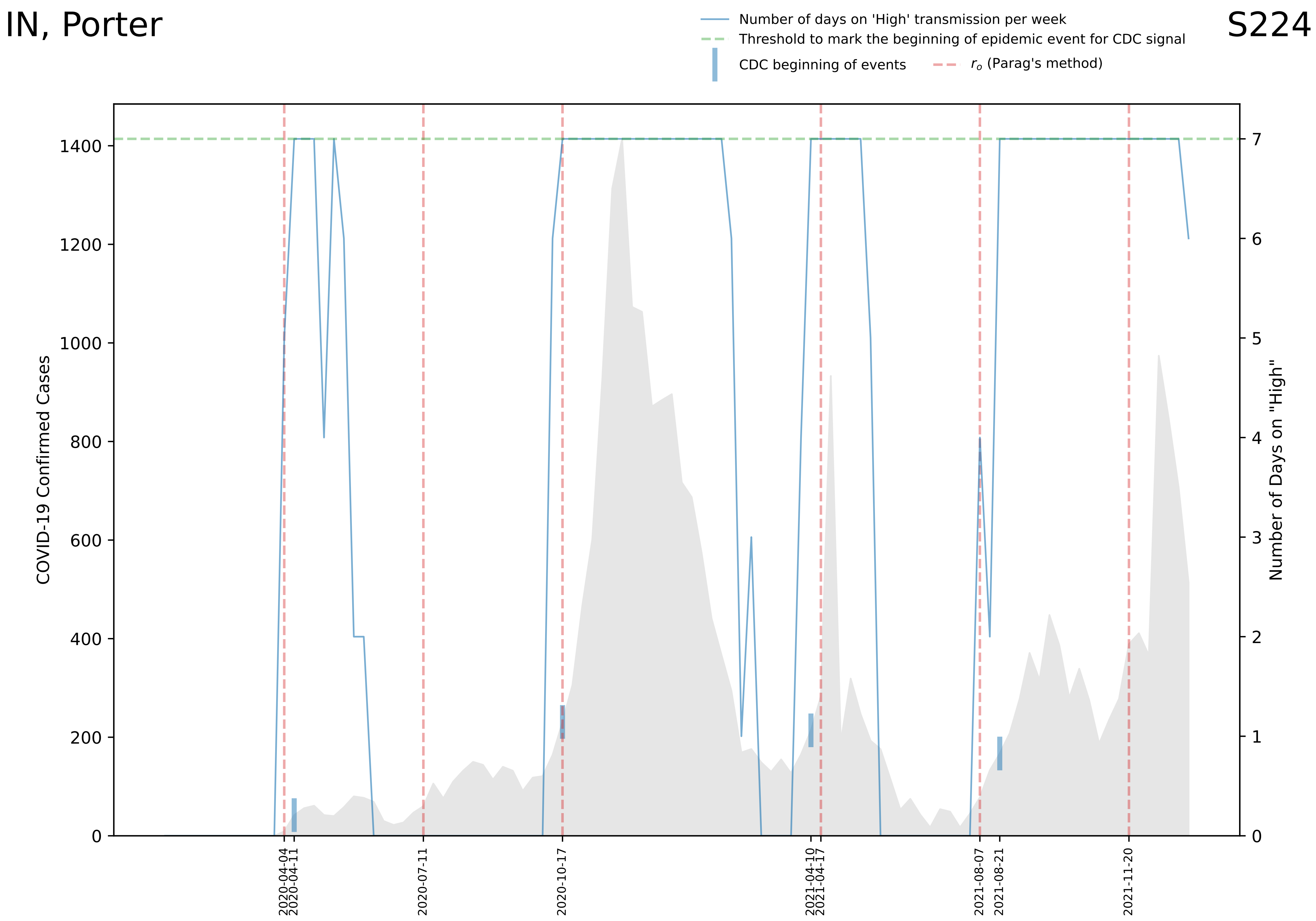

IN, Porter
S224
Number of days on 'High' transmission per week
Threshold to mark the beginning of epidemic event for CDC signal
CDC beginning of events
r_o (Parag's method)
COVID-19 Confirmed Cases
Number of Days on "High"
0
200
400
600
800
1000
1200
1400
0
1
2
3
4
5
6
7
2020-04-04
2020-04-11
2020-07-11
2020-10-17
2021-04-10
2021-04-17
2021-08-07
2021-08-21
2021-11-20

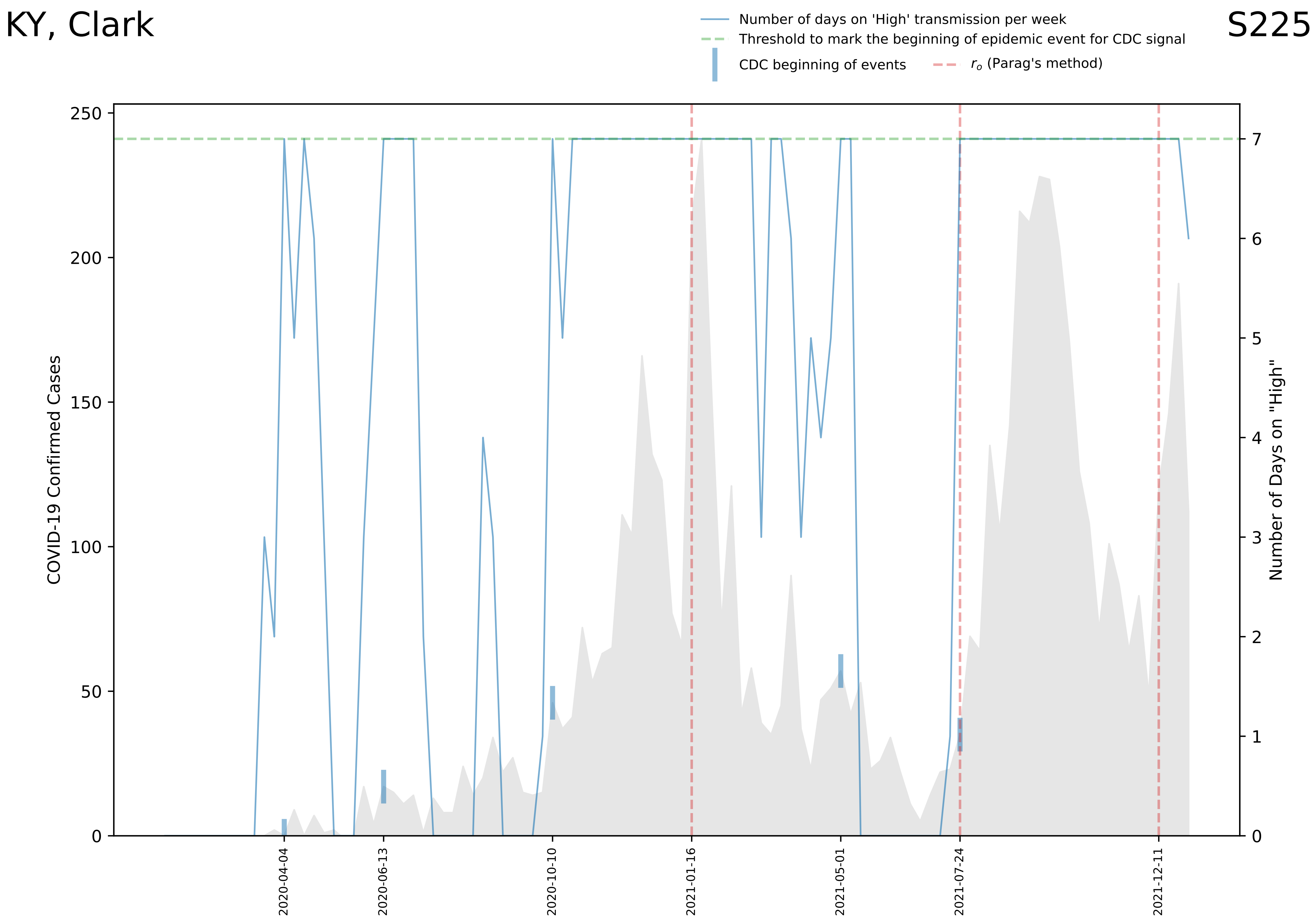

KY, Clark
S225
Number of days on 'High' transmission per week
Threshold to mark the beginning of epidemic event for CDC signal
CDC beginning of events
r_o (Parag's method)
COVID-19 Confirmed Cases
Number of Days on "High"
0
50
100
150
200
250
0
1
2
3
4
5
6
7
2020-04-04
2020-06-13
2020-10-10
2021-01-16
2021-05-01
2021-07-24
2021-12-11

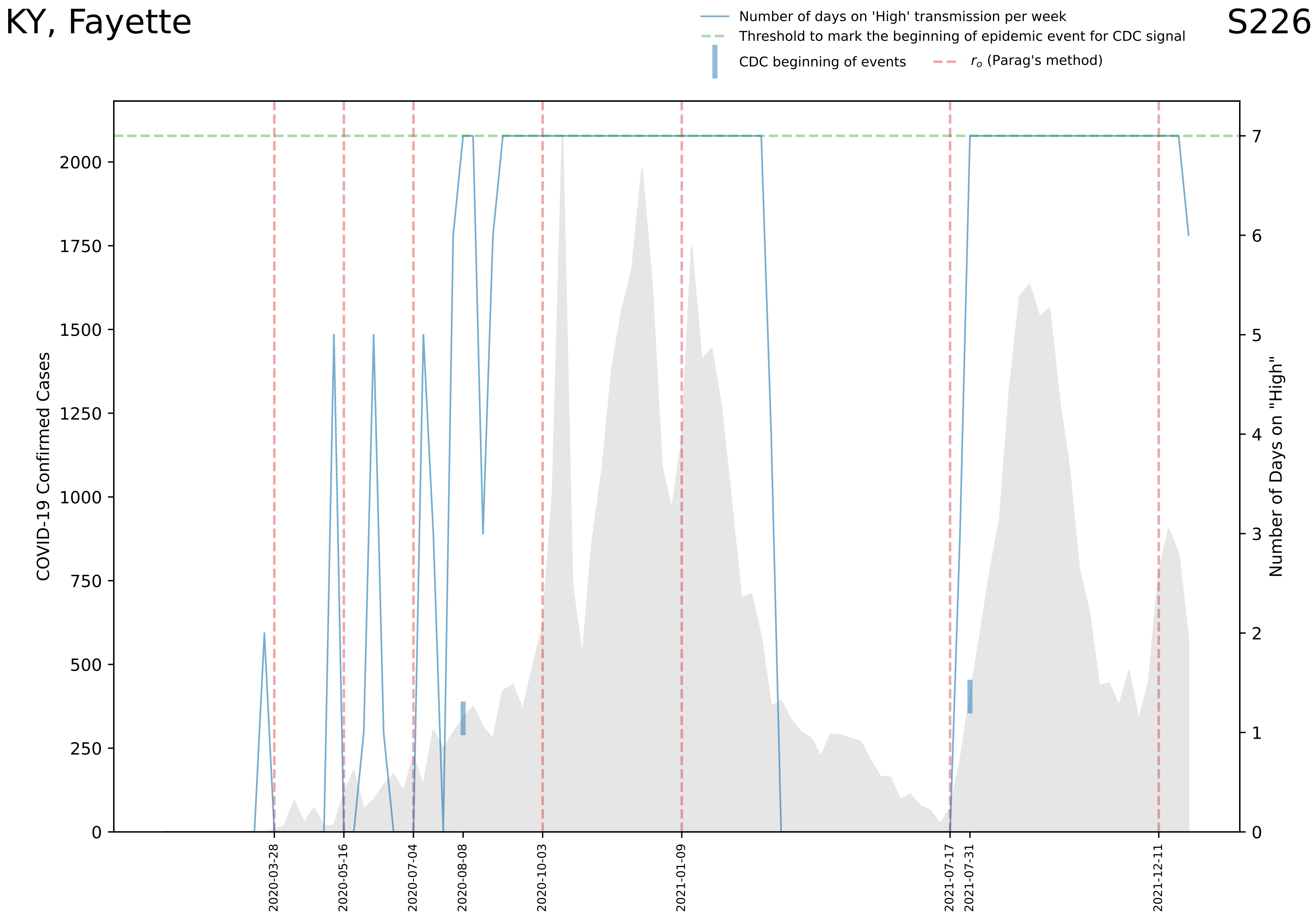

KY, Fayette
S226
Number of days on 'High' transmission per week
Threshold to mark the beginning of epidemic event for CDC signal
CDC beginning of events
r_o (Parag's method)
COVID-19 Confirmed Cases
Number of Days on "High"
0
250
500
750
1000
1250
1500
1750
2000
0
1
2
3
4
5
6
7
2020-03-28
2020-05-16
2020-07-04
2020-08-08
2020-10-03
2021-01-09
2021-07-17
2021-07-31
2021-12-11

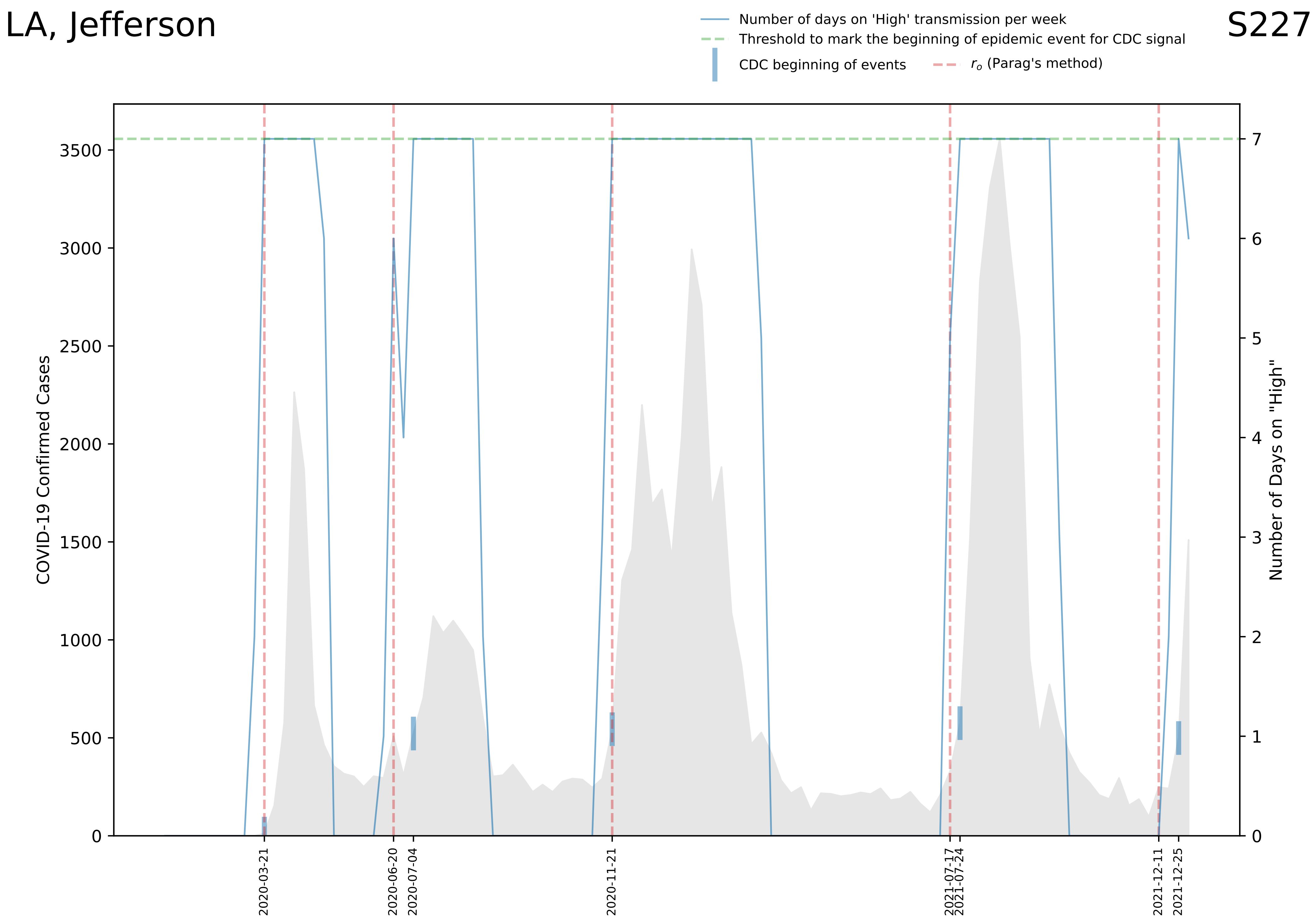

LA, Jefferson
S227
Number of days on 'High' transmission per week
Threshold to mark the beginning of epidemic event for CDC signal
CDC beginning of events
r_o (Parag's method)
COVID-19 Confirmed Cases
Number of Days on "High"
0
500
1000
1500
2000
2500
3000
3500
0
1
2
3
4
5
6
7
2020-03-21
2020-06-20
2020-07-04
2020-11-21
2021-07-17
2021-07-24
2021-12-11
2021-12-25

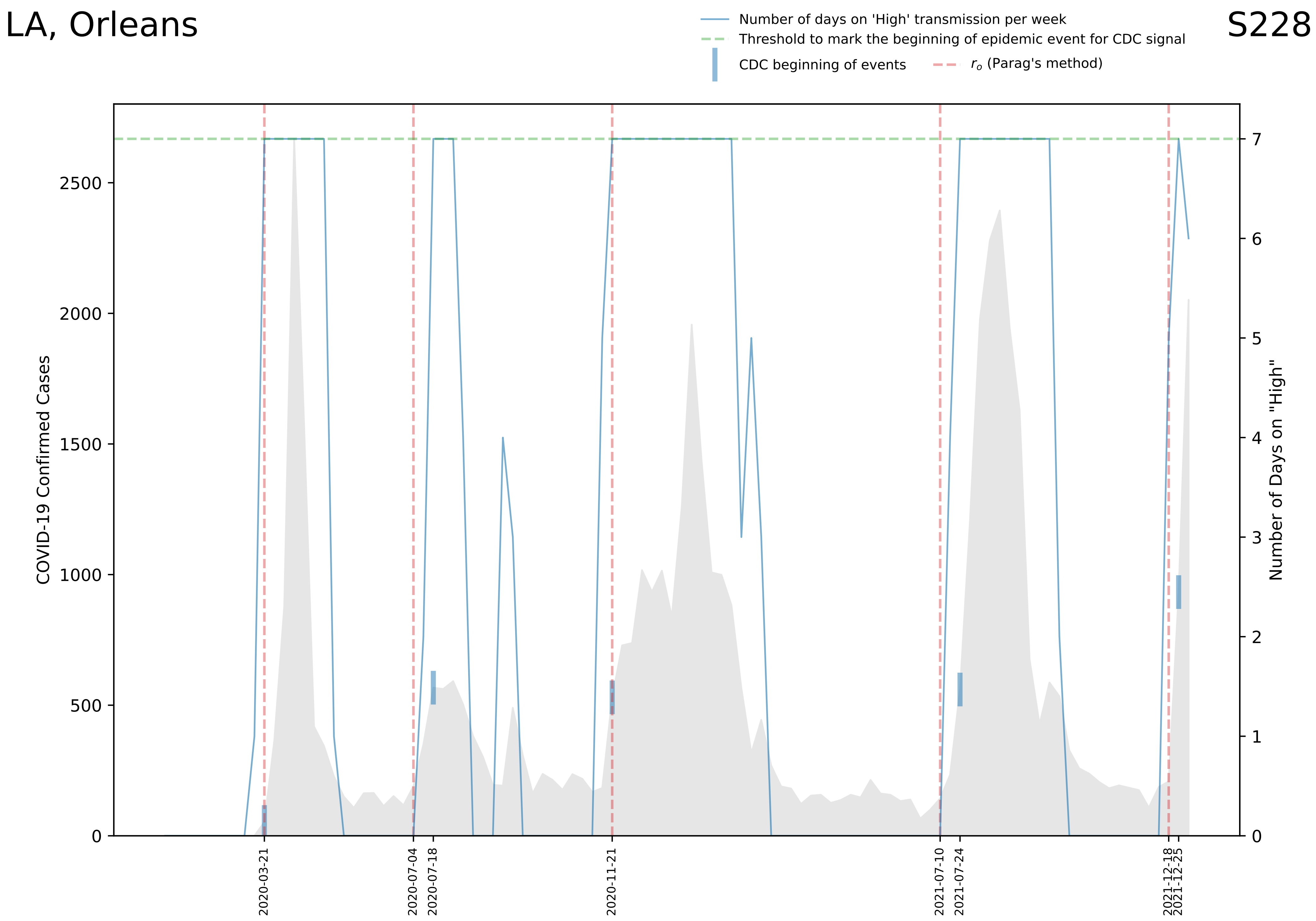

LA, Orleans
S228
Number of days on 'High' transmission per week
Threshold to mark the beginning of epidemic event for CDC signal
CDC beginning of events
r_o (Parag's method)
COVID-19 Confirmed Cases
Number of Days on "High"
0
500
1000
1500
2000
2500
0
1
2
3
4
5
6
7
2020-03-21
2020-07-04
2020-07-18
2020-11-21
2021-07-10
2021-07-24
2021-12-18
2021-12-25

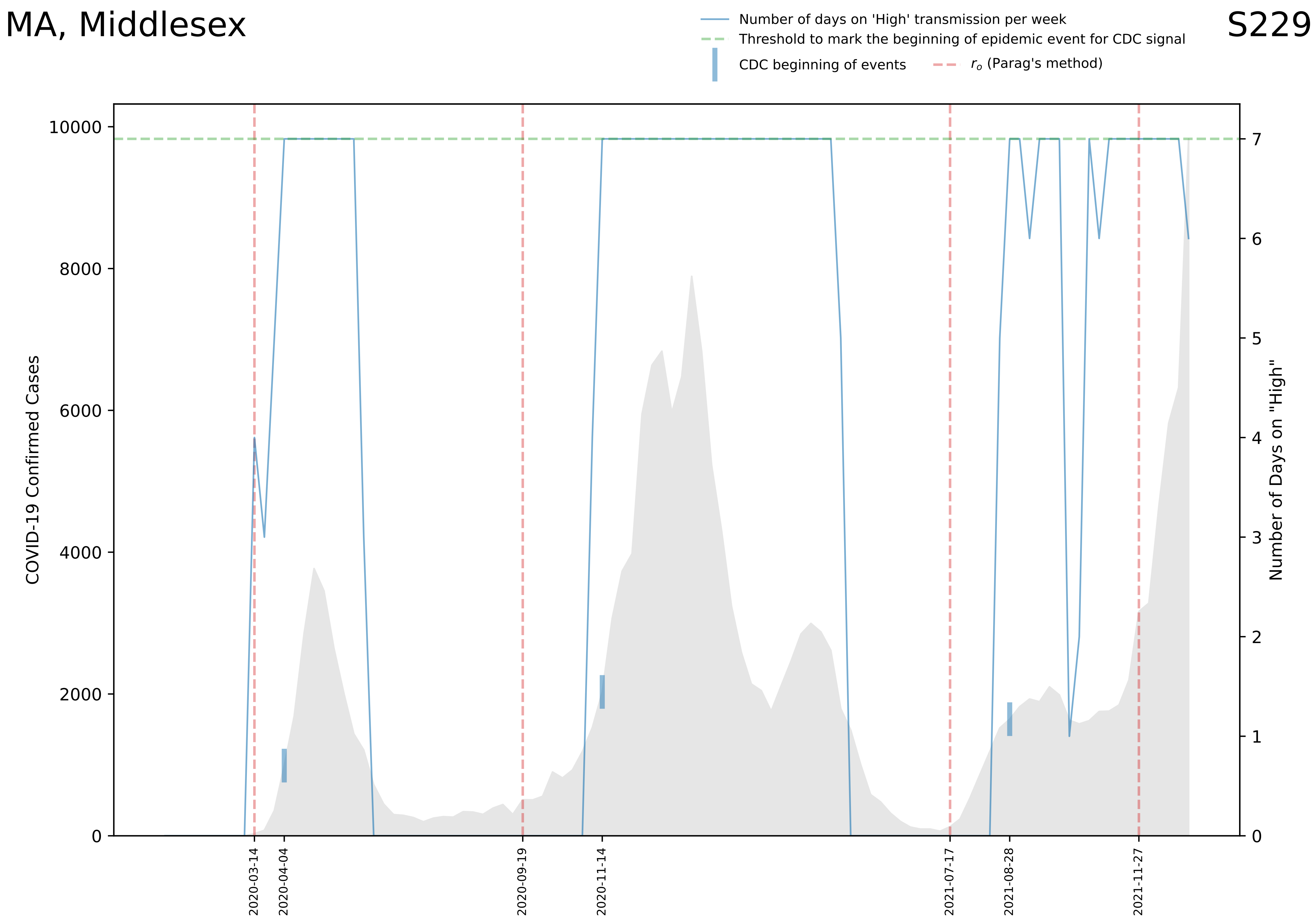

MA, Middlesex
S229
Number of days on 'High' transmission per week
Threshold to mark the beginning of epidemic event for CDC signal
CDC beginning of events
r_o (Parag's method)
COVID-19 Confirmed Cases
Number of Days on "High"
0
2000
4000
6000
8000
10000
0
1
2
3
4
5
6
7
2020-03-14
2020-04-04
2020-09-19
2020-11-14
2021-07-17
2021-08-28
2021-11-27

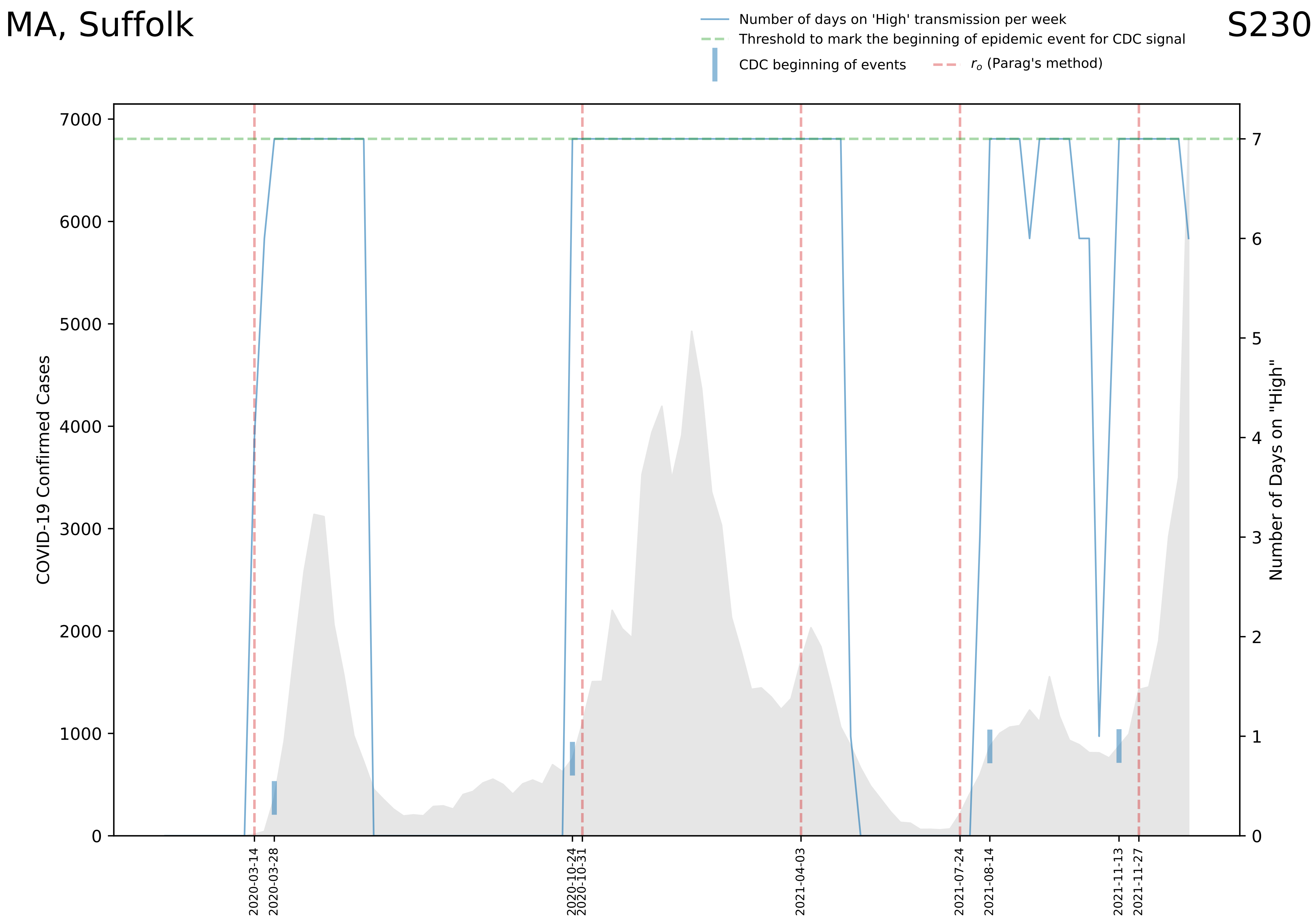

MA, Suffolk
S230
Number of days on 'High' transmission per week
Threshold to mark the beginning of epidemic event for CDC signal
CDC beginning of events
r_o (Parag's method)
COVID-19 Confirmed Cases
Number of Days on "High"
0
1000
2000
3000
4000
5000
6000
7000
0
1
2
3
4
5
6
7
2020-03-14
2020-03-28
2020-10-24
2020-10-31
2021-04-03
2021-07-24
2021-08-14
2021-11-13
2021-11-27

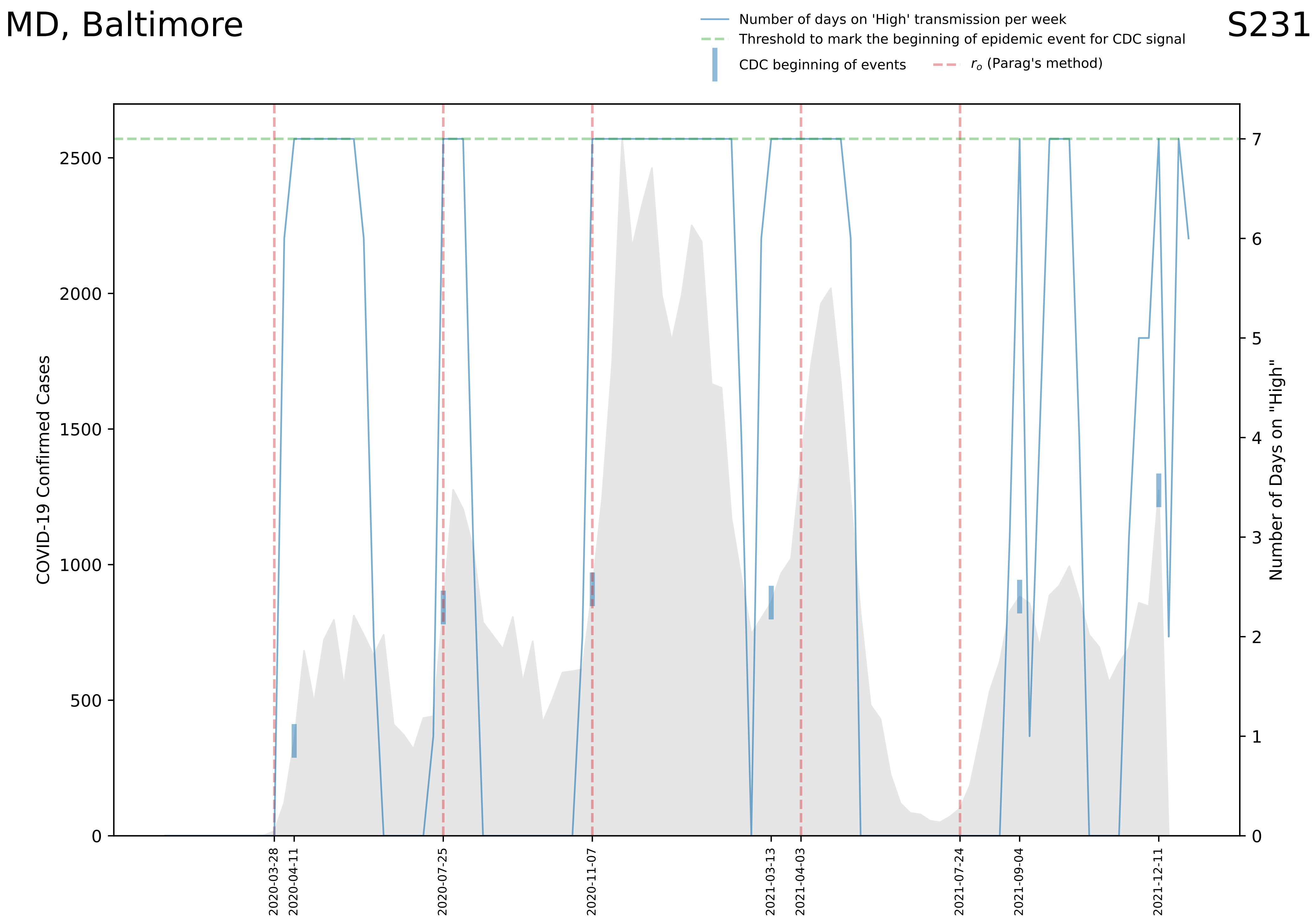

MD, Baltimore
S231
Number of days on 'High' transmission per week
Threshold to mark the beginning of epidemic event for CDC signal
CDC beginning of events
r_o (Parag's method)
COVID-19 Confirmed Cases
Number of Days on "High"
0
500
1000
1500
2000
2500
0
1
2
3
4
5
6
7
2020-03-28
2020-04-11
2020-07-25
2020-11-07
2021-03-13
2021-04-03
2021-07-24
2021-09-04
2021-12-11

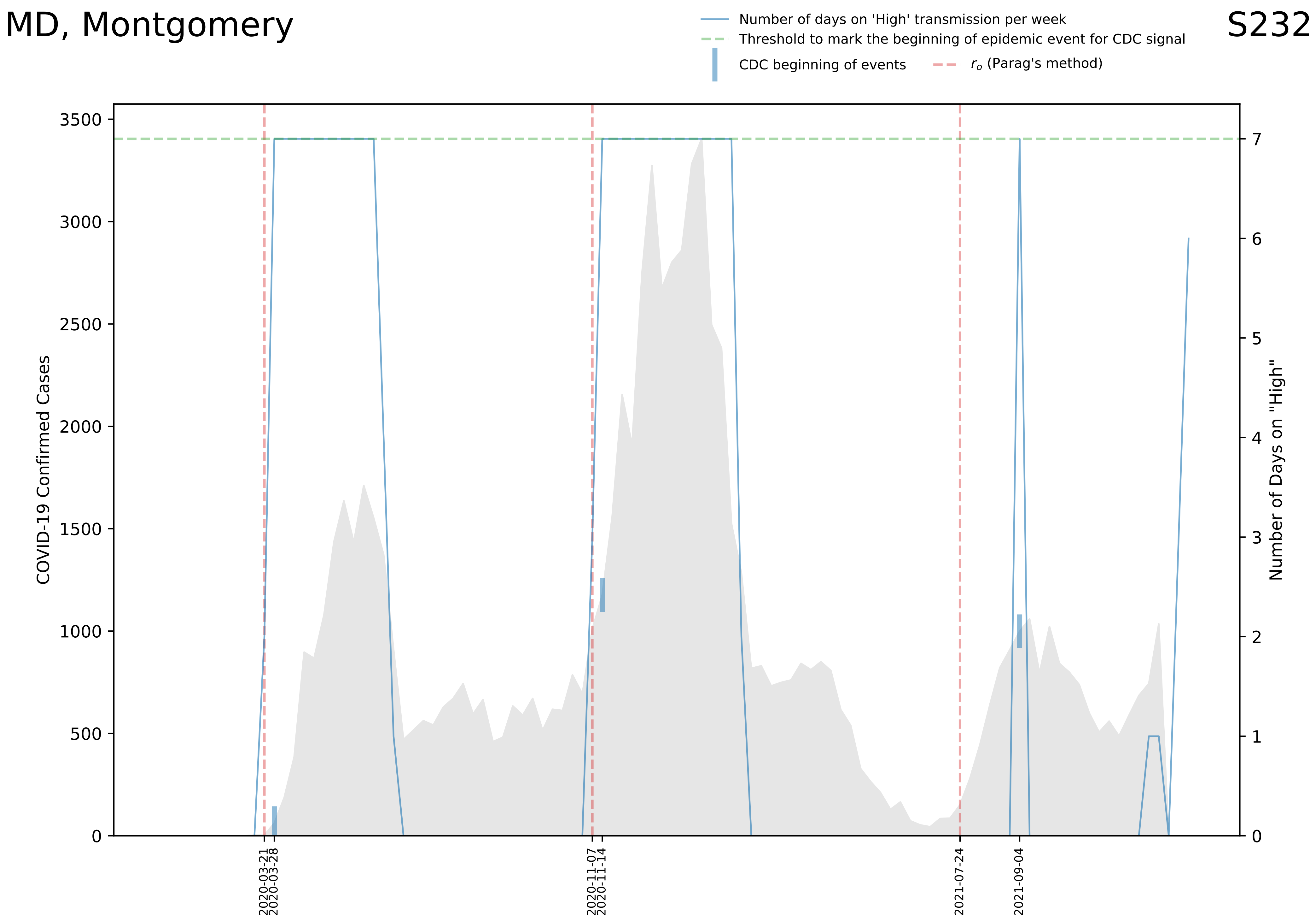

MD, Montgomery
S232
Number of days on 'High' transmission per week
Threshold to mark the beginning of epidemic event for CDC signal
CDC beginning of events
r_o (Parag's method)
COVID-19 Confirmed Cases
Number of Days on "High"
0
500
1000
1500
2000
2500
3000
3500
0
1
2
3
4
5
6
7
2020-03-21
2020-03-28
2020-11-07
2020-11-14
2021-07-24
2021-09-04

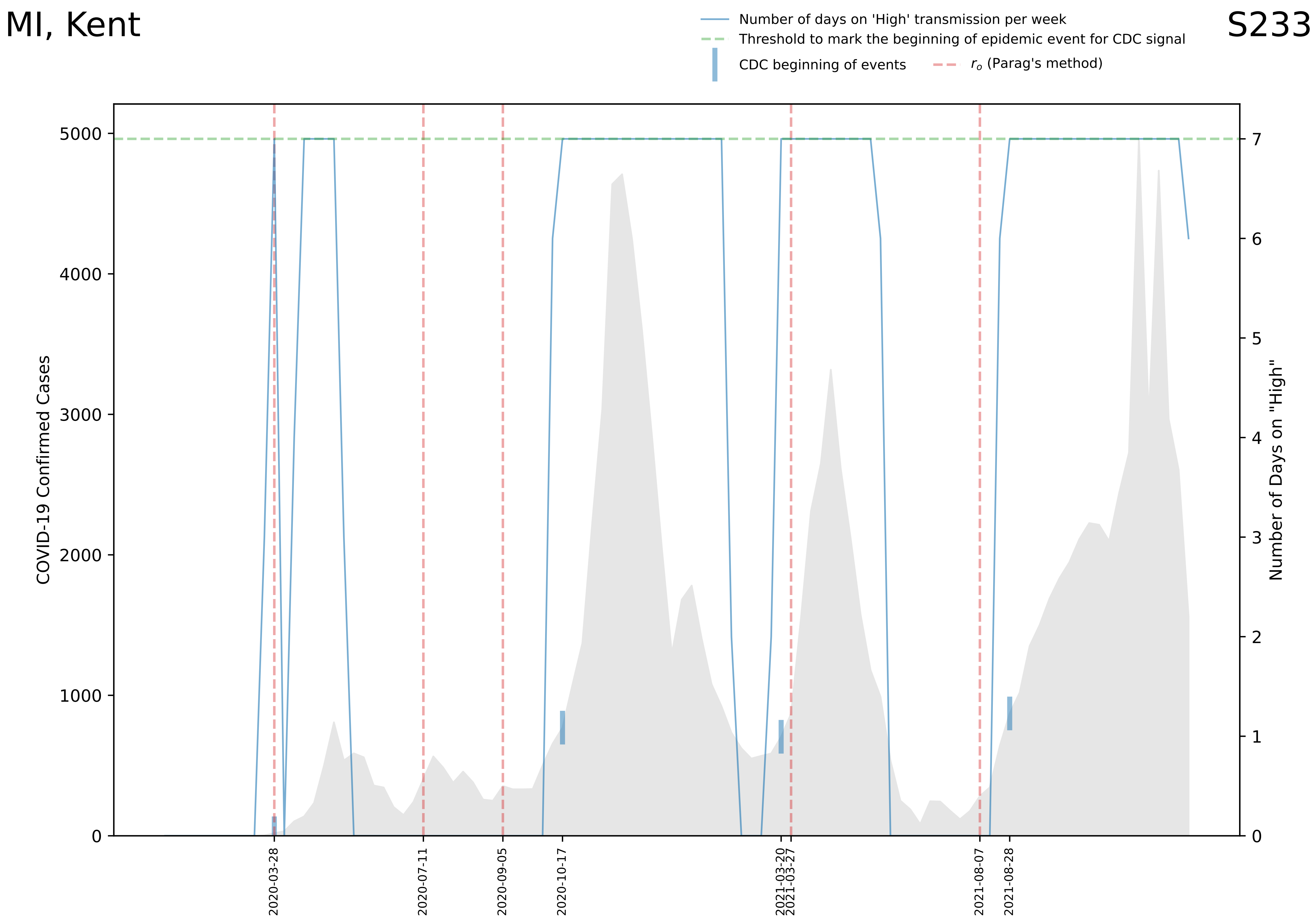

MI, Kent
S233
Number of days on 'High' transmission per week
Threshold to mark the beginning of epidemic event for CDC signal
CDC beginning of events
r_o (Parag's method)
COVID-19 Confirmed Cases
Number of Days on "High"
0
1000
2000
3000
4000
5000
0
1
2
3
4
5
6
7
2020-03-28
2020-07-11
2020-09-05
2020-10-17
2021-03-20
2021-03-27
2021-08-07
2021-08-28

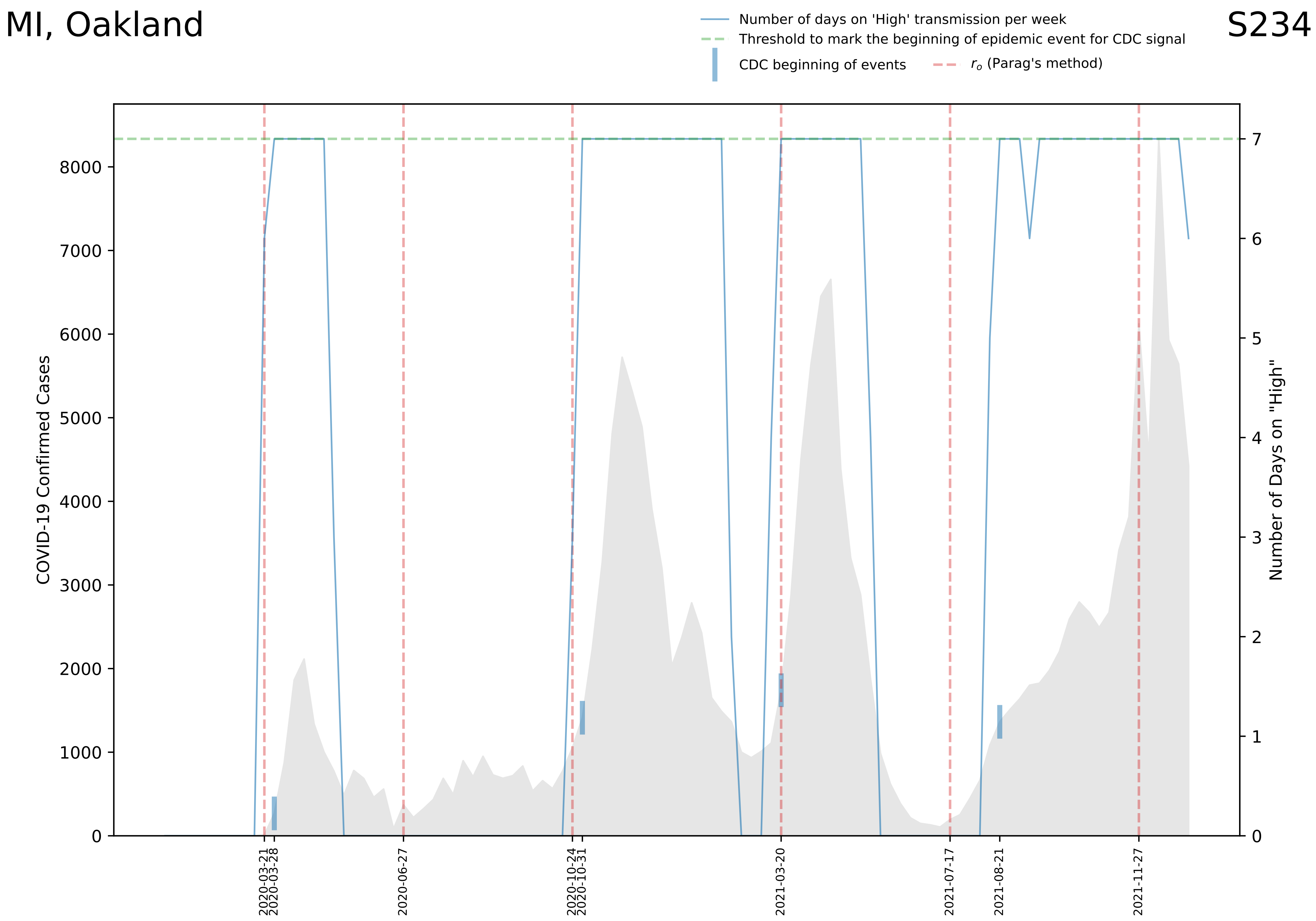

MI, Oakland
S234
Number of days on 'High' transmission per week
Threshold to mark the beginning of epidemic event for CDC signal
CDC beginning of events
r_o (Parag's method)
COVID-19 Confirmed Cases
Number of Days on "High"
0
1000
2000
3000
4000
5000
6000
7000
8000
0
1
2
3
4
5
6
7
2020-03-21
2020-03-28
2020-06-27
2020-10-24
2020-10-31
2021-03-20
2021-07-17
2021-08-21
2021-11-27

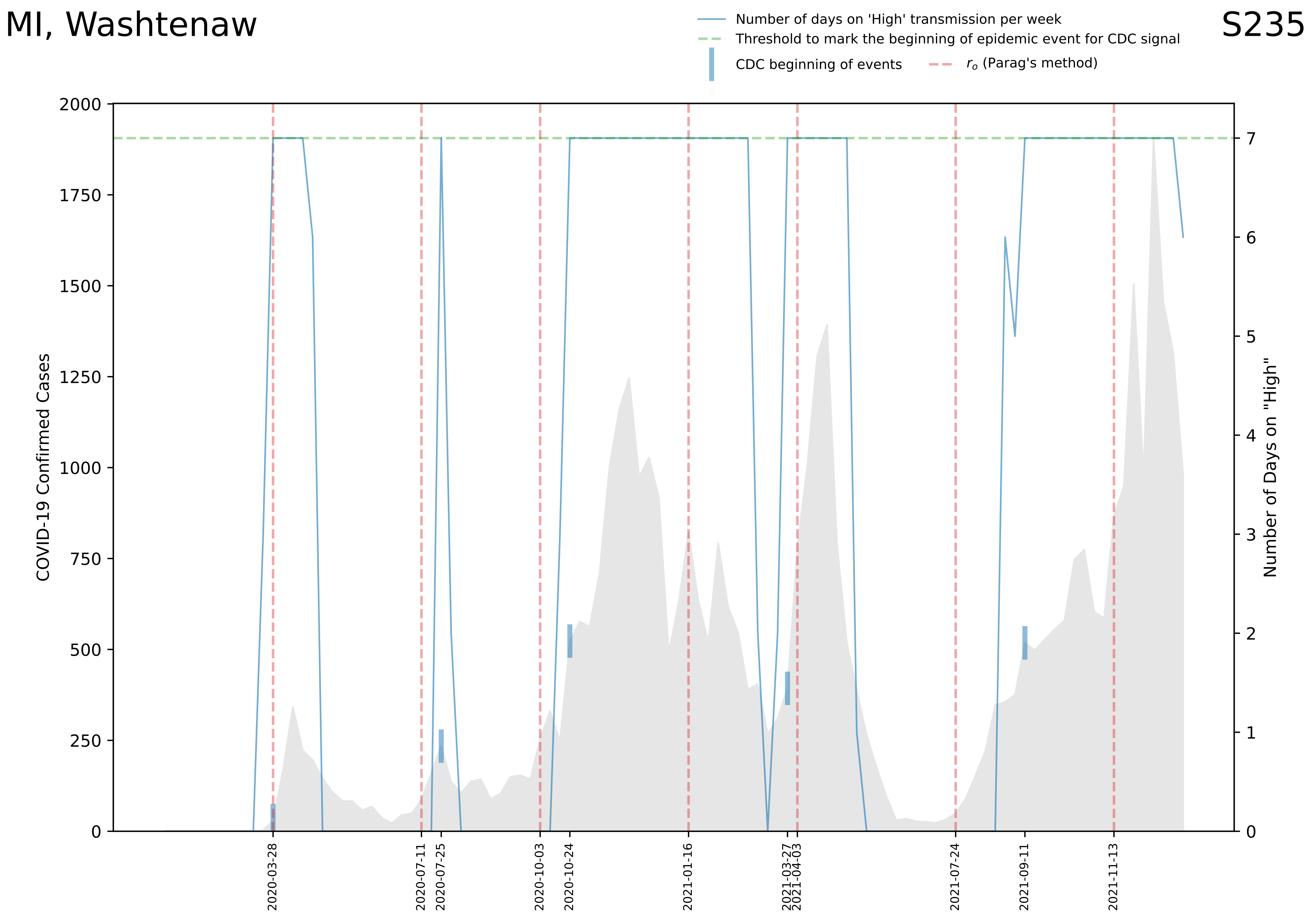
MI, Washtenaw
S235
Number of days on 'High' transmission per week
Threshold to mark the beginning of epidemic event for CDC signal
CDC beginning of events
r_o (Parag's method)
COVID-19 Confirmed Cases
Number of Days on "High"
0
250
500
750
1000
1250
1500
1750
2000
0
1
2
3
4
5
6
7
2020-03-28
2020-07-11
2020-07-25
2020-10-03
2020-10-24
2021-01-16
2021-03-27
2021-04-03
2021-07-24
2021-09-11
2021-11-13

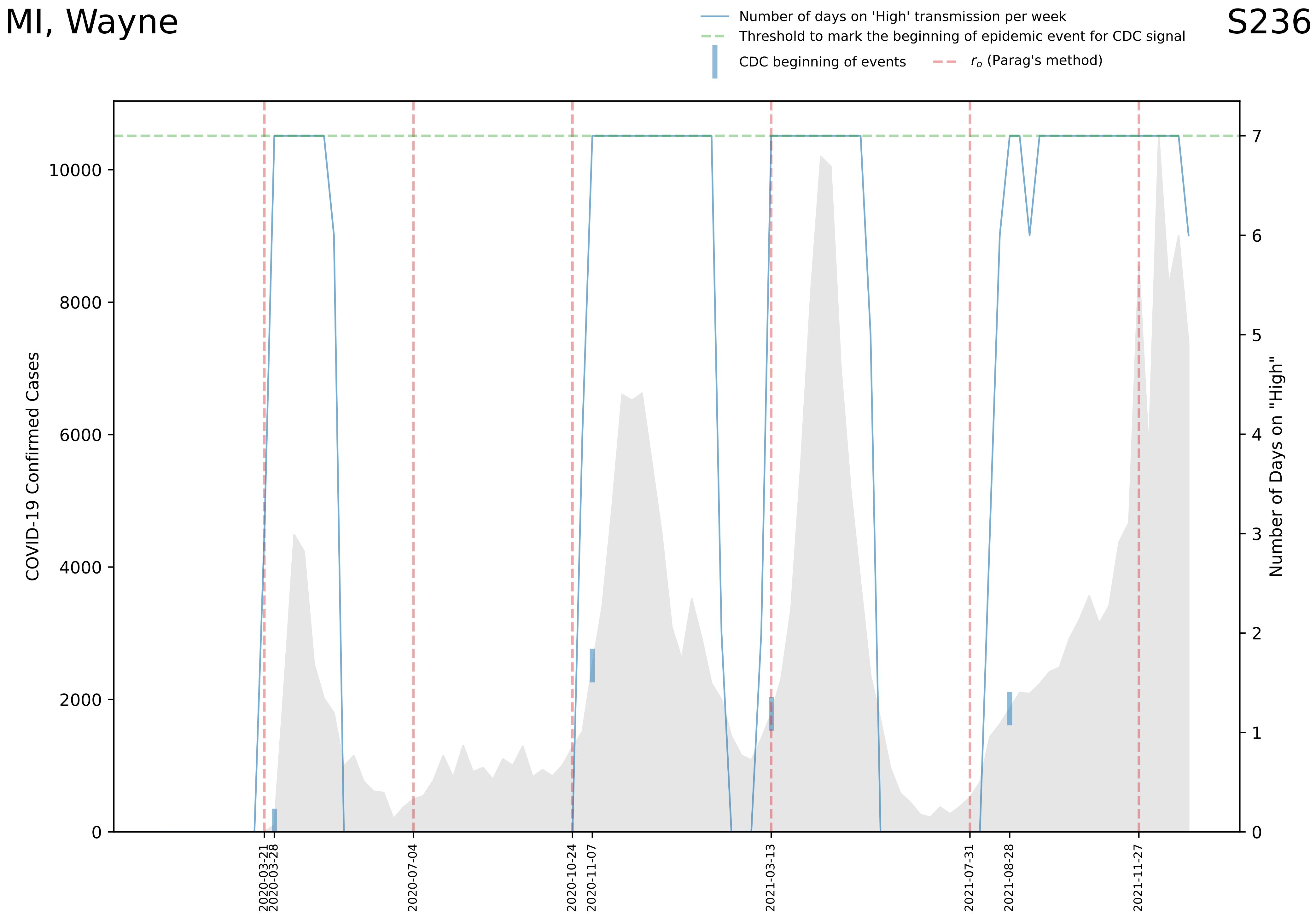

MI, Wayne
S236
Number of days on 'High' transmission per week
Threshold to mark the beginning of epidemic event for CDC signal
CDC beginning of events
r_o (Parag's method)
COVID-19 Confirmed Cases
Number of Days on "High"
0
2000
4000
6000
8000
10000
0
1
2
3
4
5
6
7
2020-03-21
2020-03-28
2020-07-04
2020-10-24
2020-11-07
2021-03-13
2021-07-31
2021-08-28
2021-11-27

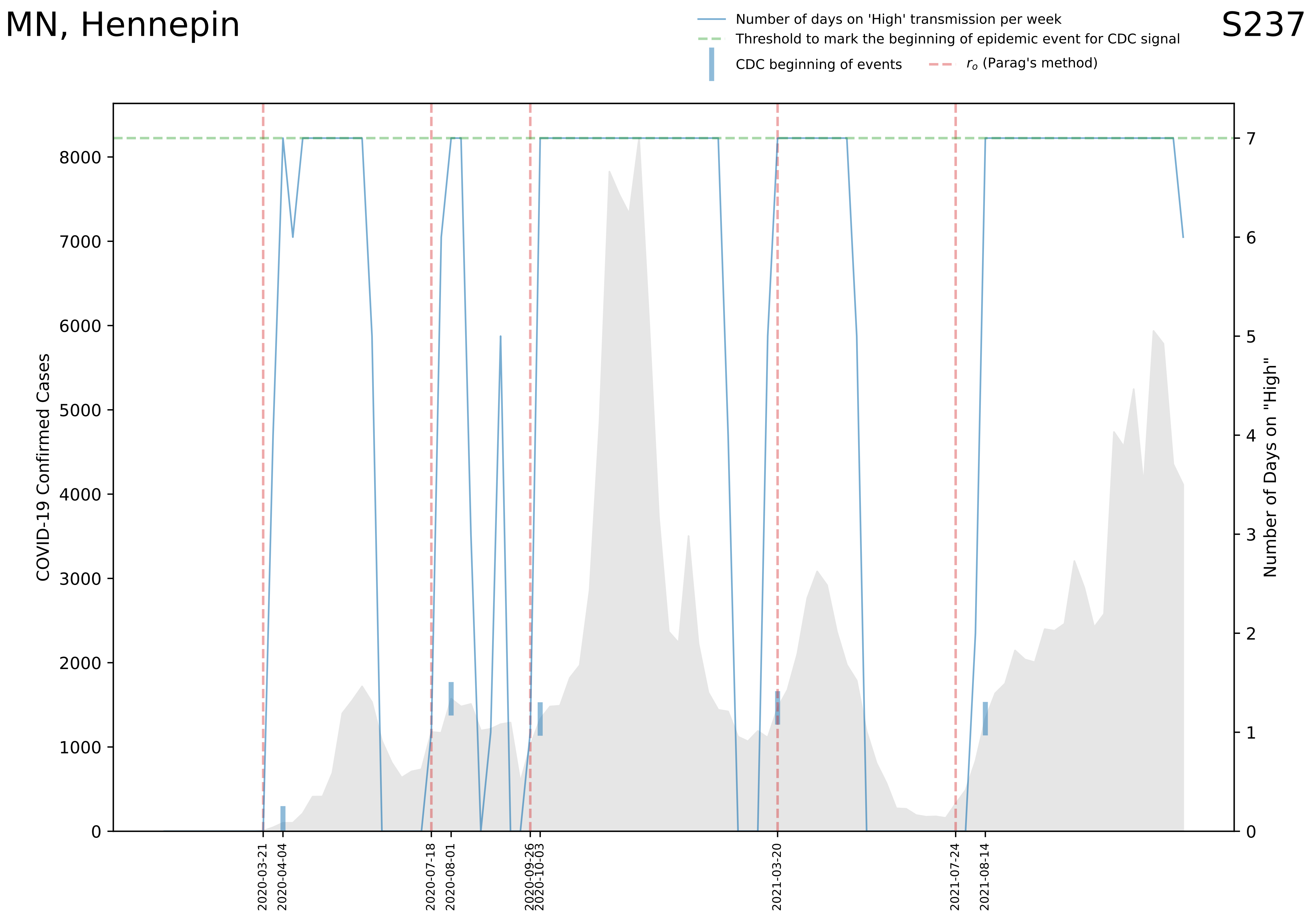

MN, Hennepin
S237
Number of days on 'High' transmission per week
Threshold to mark the beginning of epidemic event for CDC signal
CDC beginning of events
r_o (Parag's method)
COVID-19 Confirmed Cases
Number of Days on "High"
0
1000
2000
3000
4000
5000
6000
7000
8000
0
1
2
3
4
5
6
7
2020-03-21
2020-04-04
2020-07-18
2020-08-01
2020-09-26
2020-10-03
2021-03-20
2021-07-24
2021-08-14

MO, Clay

S238

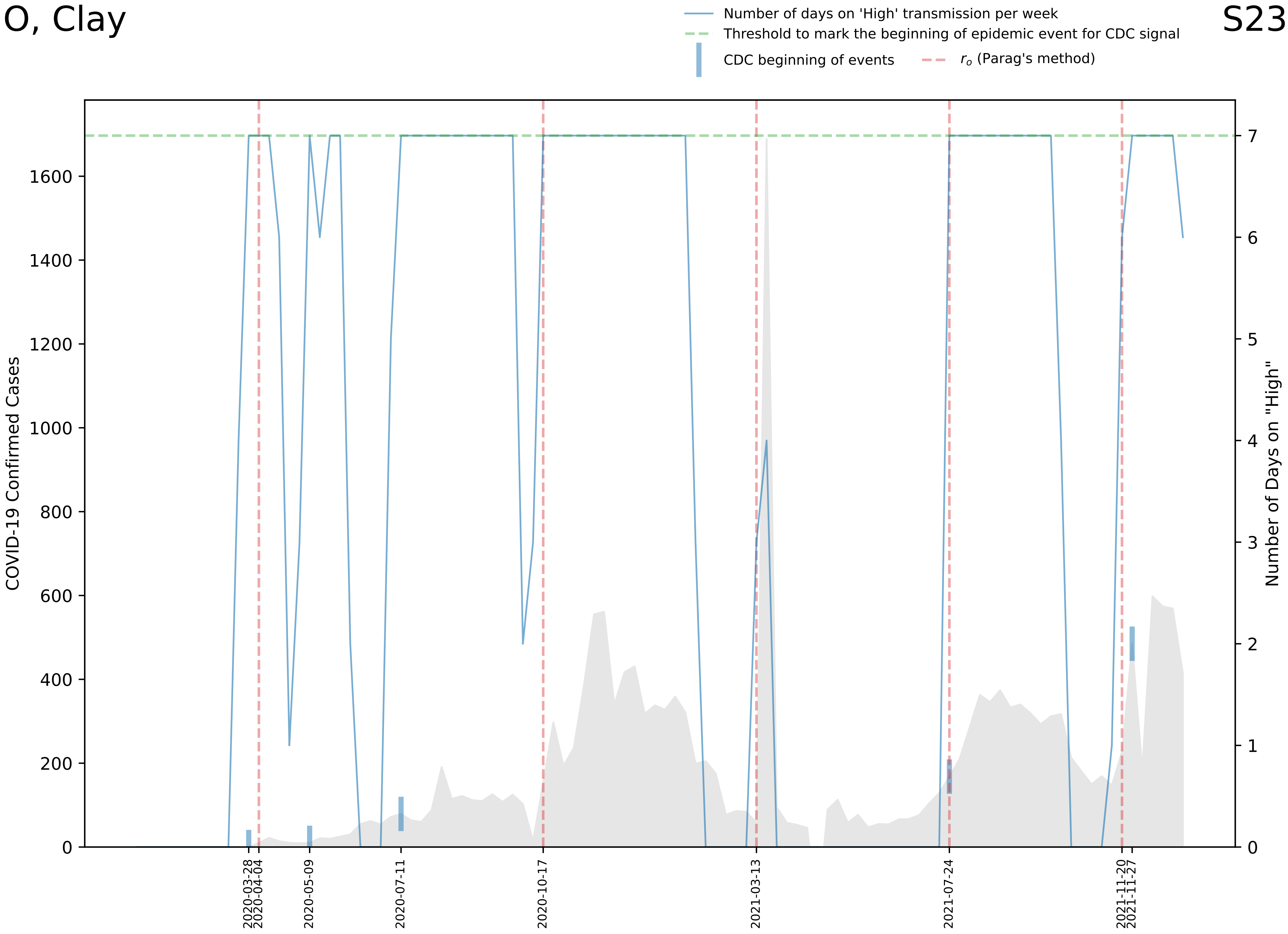

Number of days on 'High' transmission per week
Threshold to mark the beginning of epidemic event for CDC signal
CDC beginning of events
r_o (Parag's method)
COVID-19 Confirmed Cases
Number of Days on "High"
0
200
400
600
800
1000
1200
1400
1600
0
1
2
3
4
5
6
7
2020-03-28
2020-04-04
2020-05-09
2020-07-11
2020-10-17
2021-03-13
2021-07-24
2021-11-20
2021-11-27

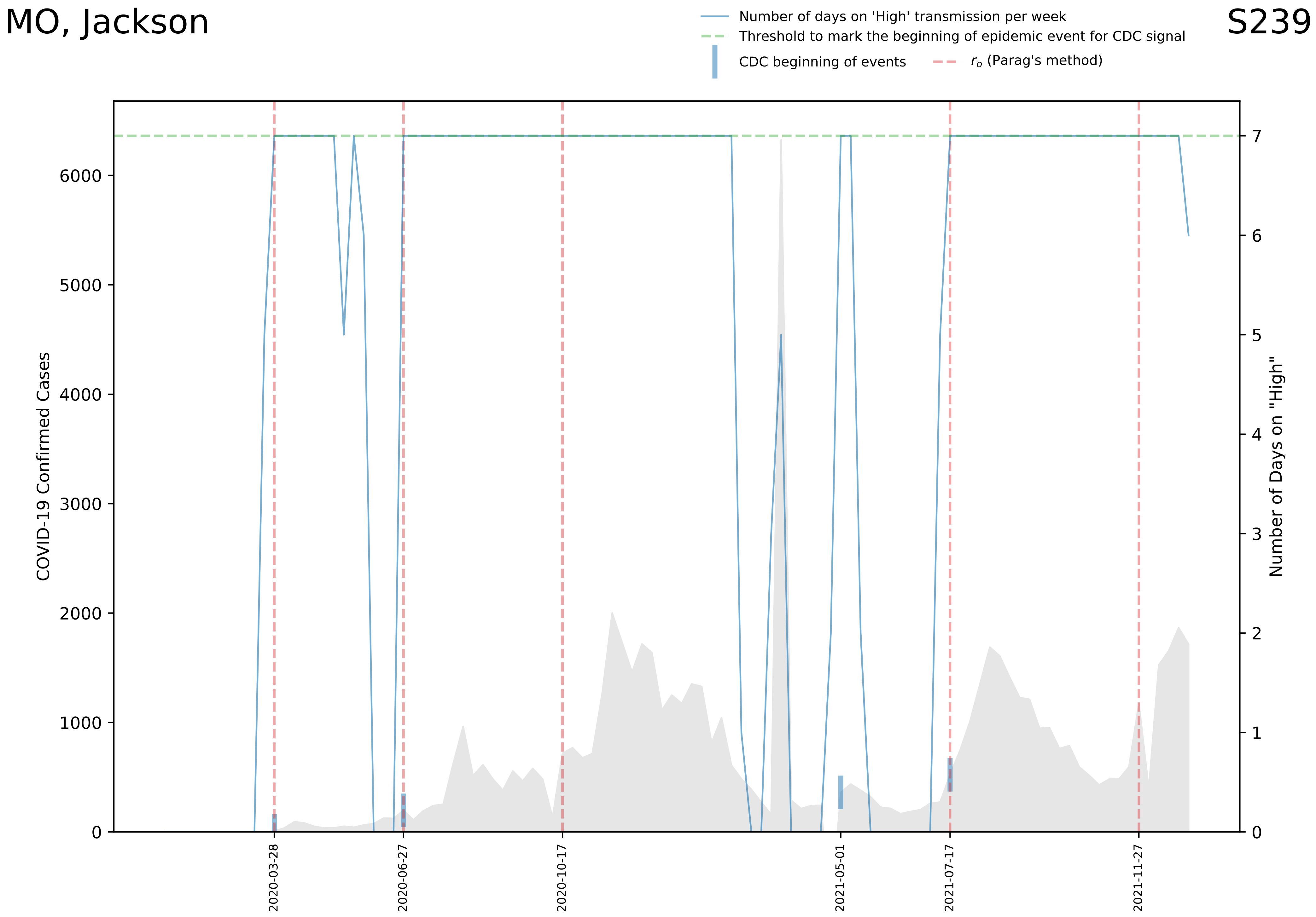

MO, Jackson
S239
Number of days on 'High' transmission per week
Threshold to mark the beginning of epidemic event for CDC signal
CDC beginning of events
r_o (Parag's method)
COVID-19 Confirmed Cases
Number of Days on "High"
0
1000
2000
3000
4000
5000
6000
0
1
2
3
4
5
6
7
2020-03-28
2020-06-27
2020-10-17
2021-05-01
2021-07-17
2021-11-27

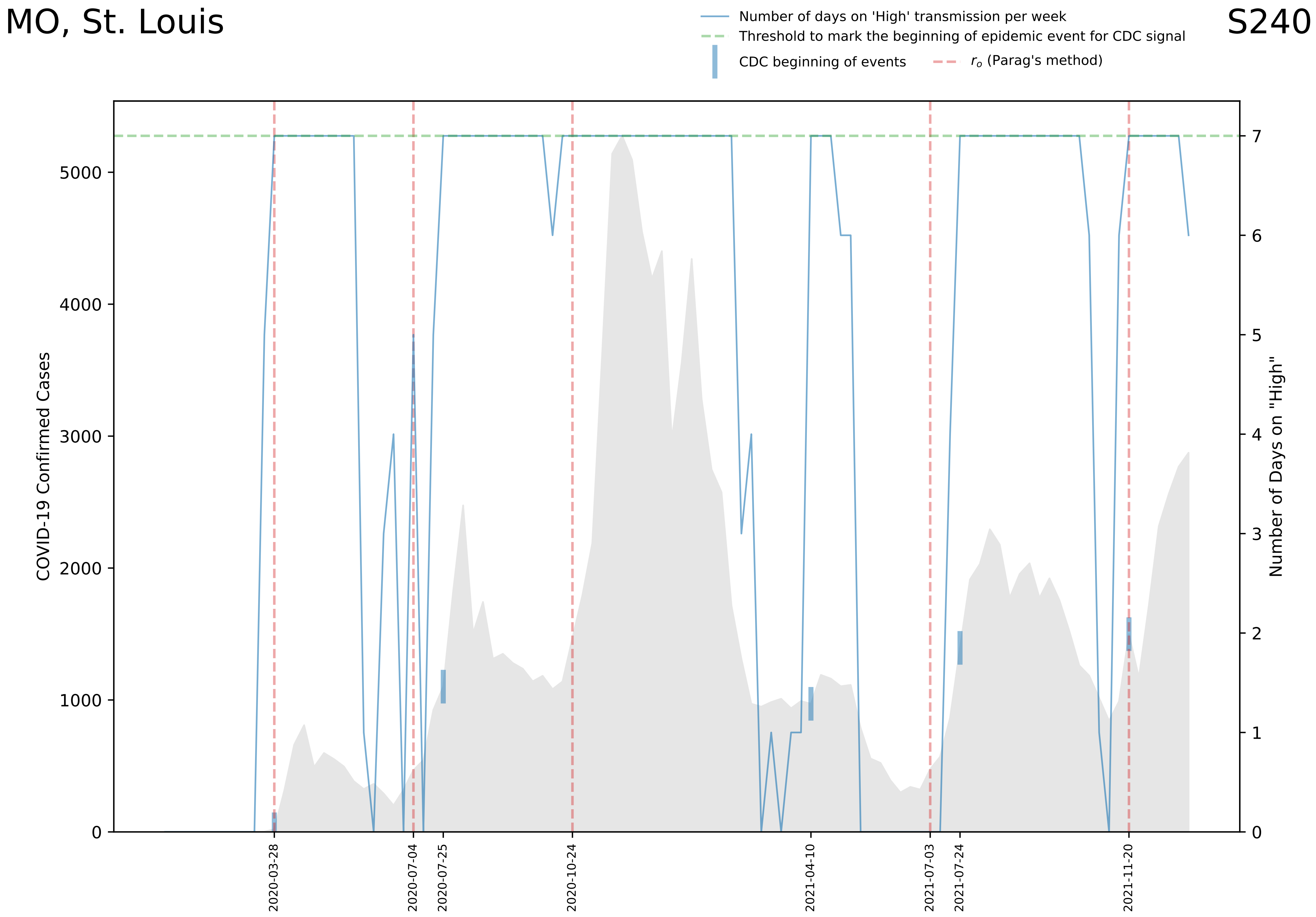

MO, St. Louis
S240
Number of days on 'High' transmission per week
Threshold to mark the beginning of epidemic event for CDC signal
CDC beginning of events
r_o (Parag's method)
COVID-19 Confirmed Cases
Number of Days on "High"
0
1000
2000
3000
4000
5000
0
1
2
3
4
5
6
7
2020-03-28
2020-07-04
2020-07-25
2020-10-24
2021-04-10
2021-07-03
2021-07-24
2021-11-20

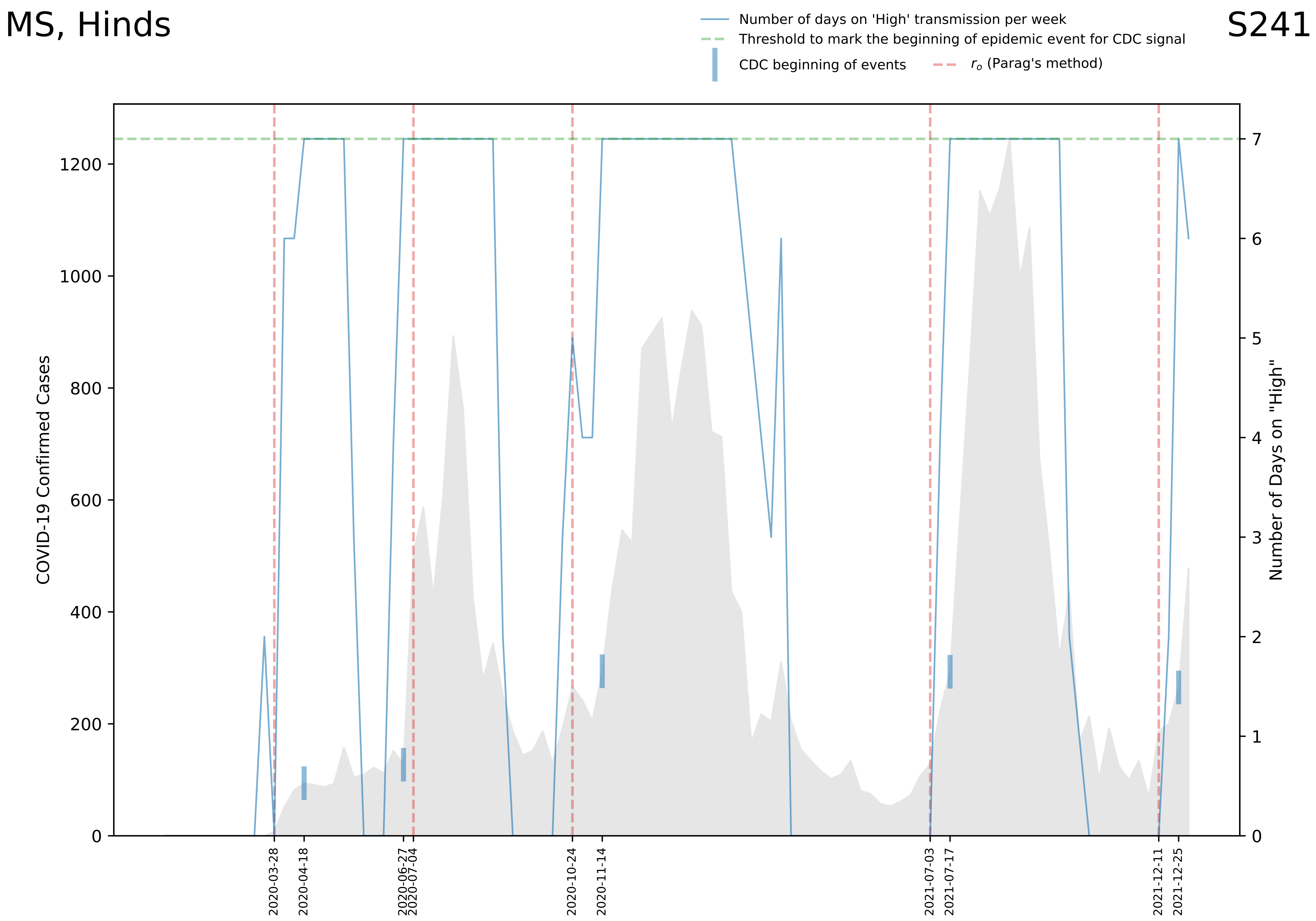

MS, Hinds
S241
Number of days on 'High' transmission per week
Threshold to mark the beginning of epidemic event for CDC signal
CDC beginning of events
r_o (Parag's method)
COVID-19 Confirmed Cases
Number of Days on "High"
0
200
400
600
800
1000
1200
0
1
2
3
4
5
6
7
2020-03-28
2020-04-18
2020-06-27
2020-07-04
2020-10-24
2020-11-14
2021-07-03
2021-07-17
2021-12-11
2021-12-25

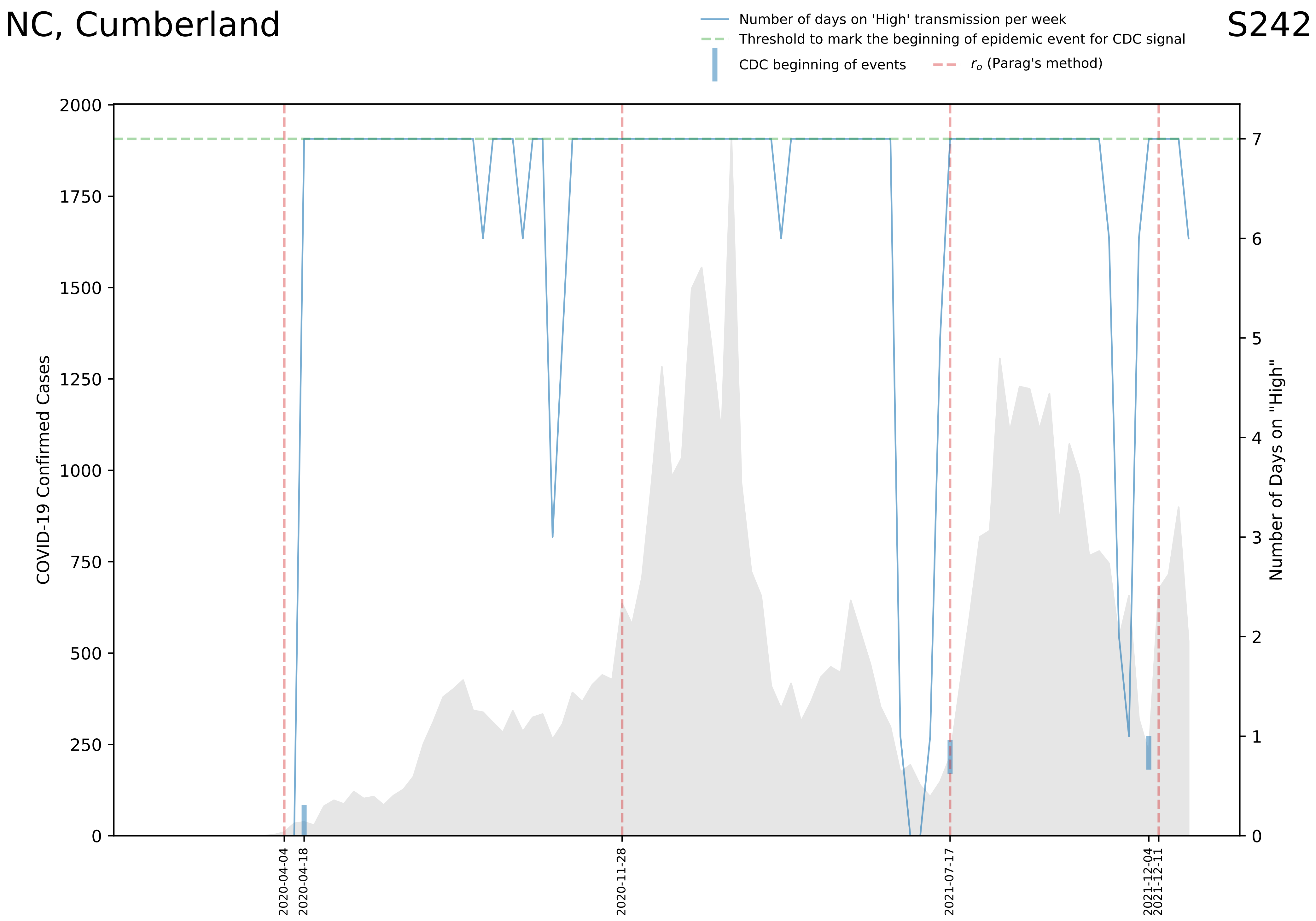

NC, Cumberland
S242
Number of days on 'High' transmission per week
Threshold to mark the beginning of epidemic event for CDC signal
CDC beginning of events
r_o (Parag's method)
COVID-19 Confirmed Cases
Number of Days on "High"
0
250
500
750
1000
1250
1500
1750
2000
0
1
2
3
4
5
6
7
2020-04-04
2020-04-18
2020-11-28
2021-07-17
2021-12-04
2021-12-11

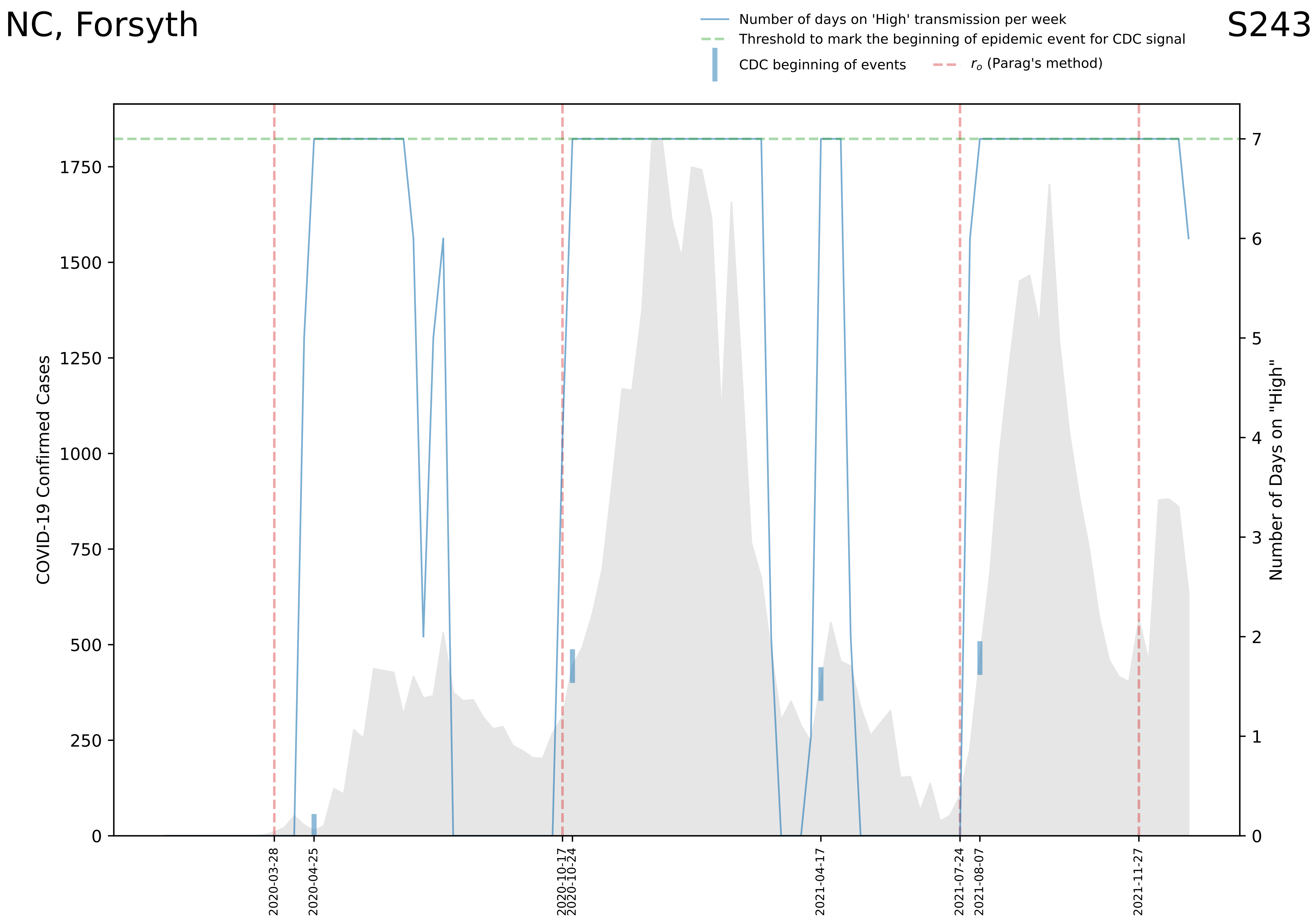

NC, Forsyth
S243
Number of days on 'High' transmission per week
Threshold to mark the beginning of epidemic event for CDC signal
CDC beginning of events
r_o (Parag's method)
COVID-19 Confirmed Cases
Number of Days on "High"
0
250
500
750
1000
1250
1500
1750
0
1
2
3
4
5
6
7
2020-03-28
2020-04-25
2020-10-17
2020-10-24
2021-04-17
2021-07-24
2021-08-07
2021-11-27

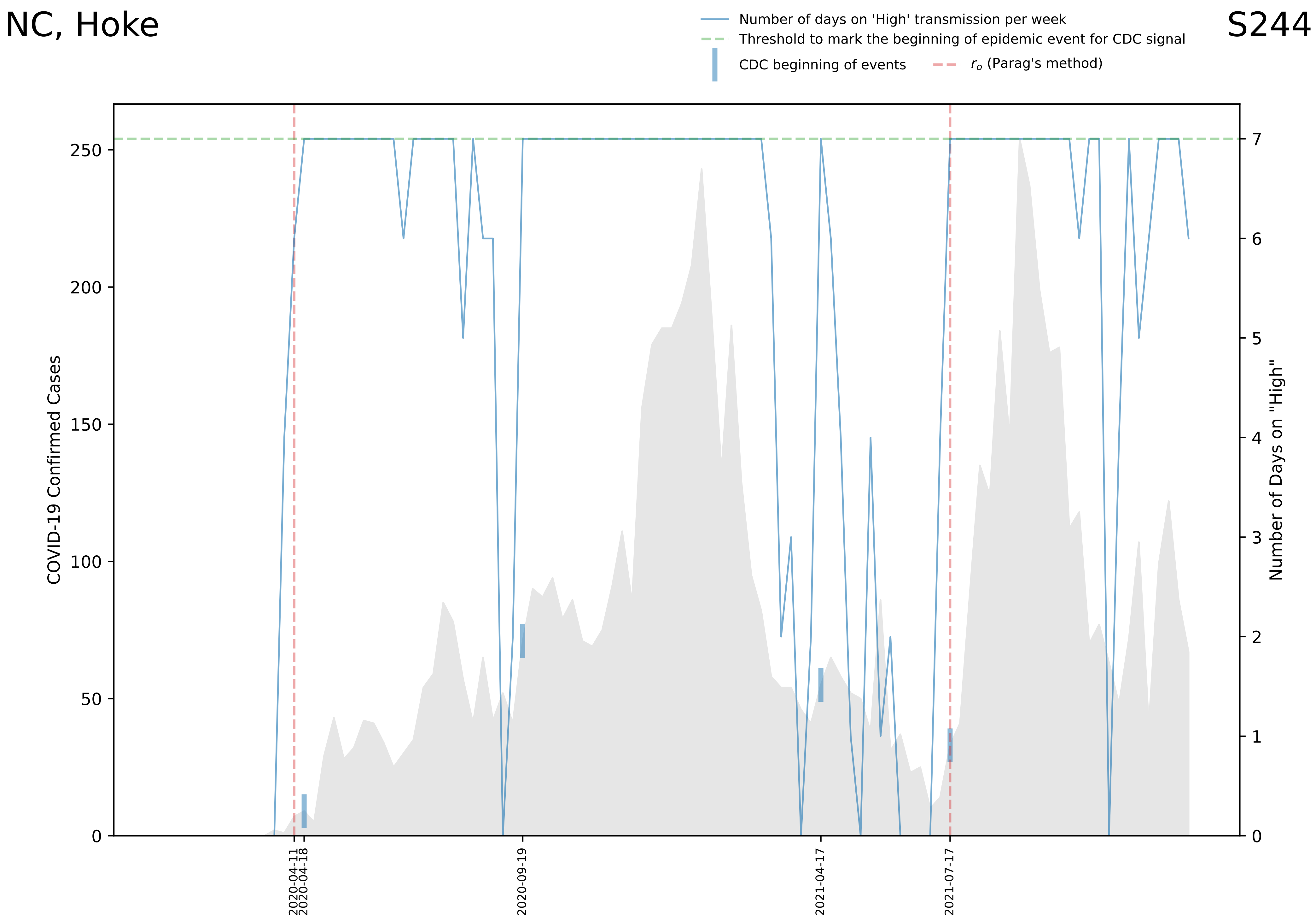

NC, Hoke
S244
Number of days on 'High' transmission per week
Threshold to mark the beginning of epidemic event for CDC signal
CDC beginning of events
r_o (Parag's method)
COVID-19 Confirmed Cases
Number of Days on "High"
0
50
100
150
200
250
0
1
2
3
4
5
6
7
2020-04-11
2020-04-18
2020-09-19
2021-04-17
2021-07-17

NC, Johnston

S245

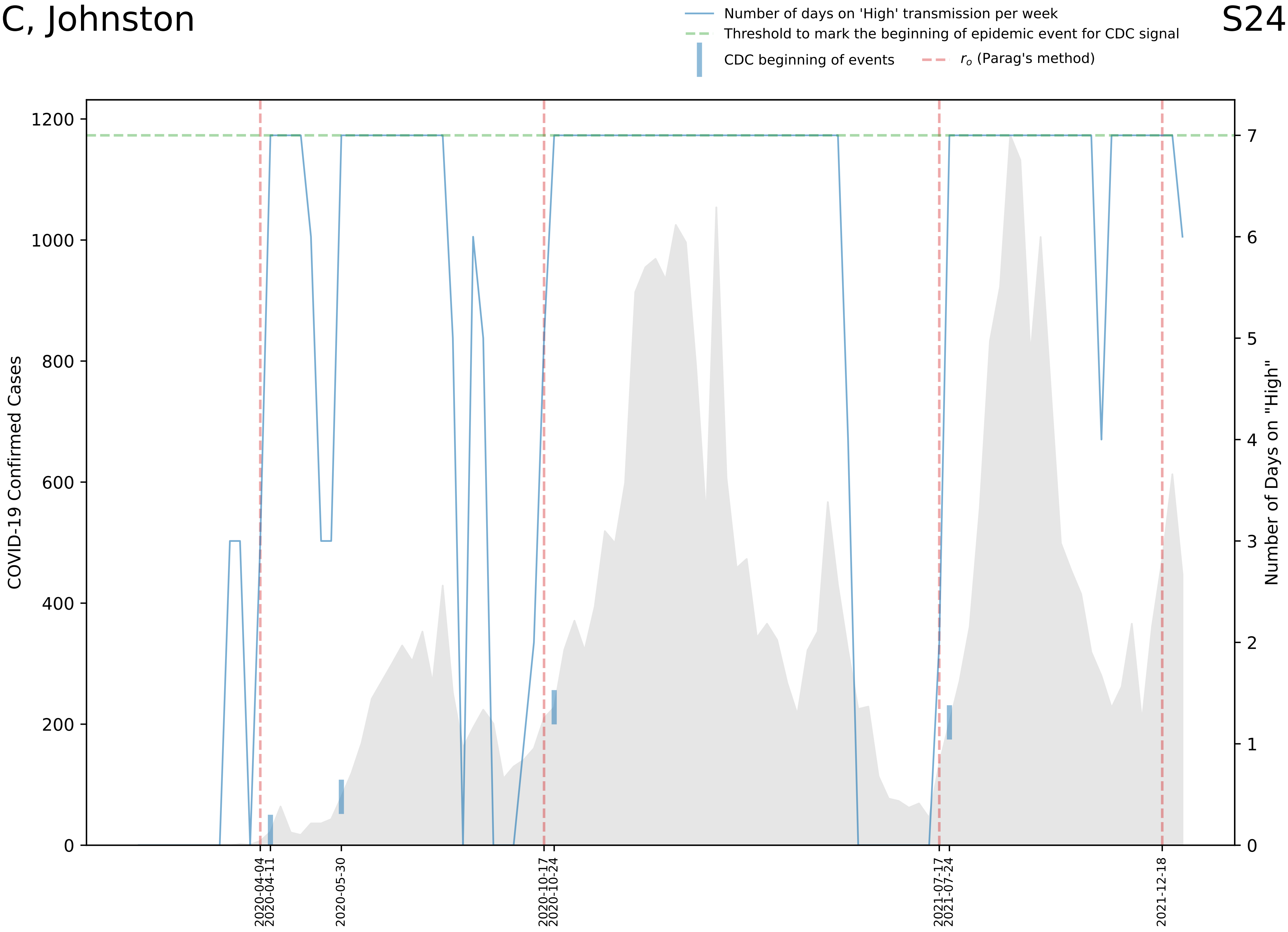

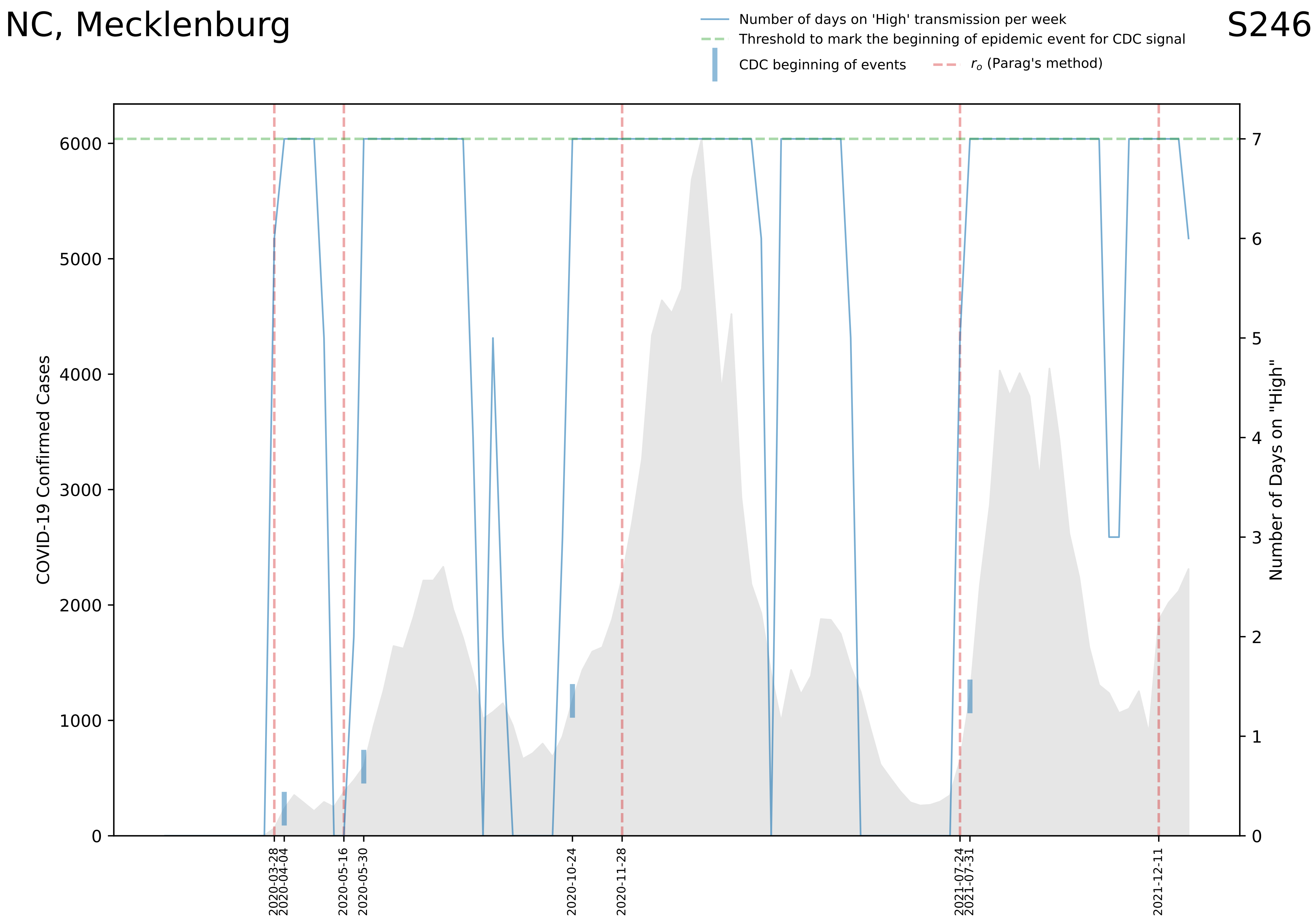

NC, Mecklenburg
S246
Number of days on 'High' transmission per week
Threshold to mark the beginning of epidemic event for CDC signal
CDC beginning of events
r_o (Parag's method)
COVID-19 Confirmed Cases
Number of Days on "High"
0
1000
2000
3000
4000
5000
6000
0
1
2
3
4
5
6
7
2020-03-28
2020-04-04
2020-05-16
2020-05-30
2020-10-24
2020-11-28
2021-07-24
2021-07-31
2021-12-11

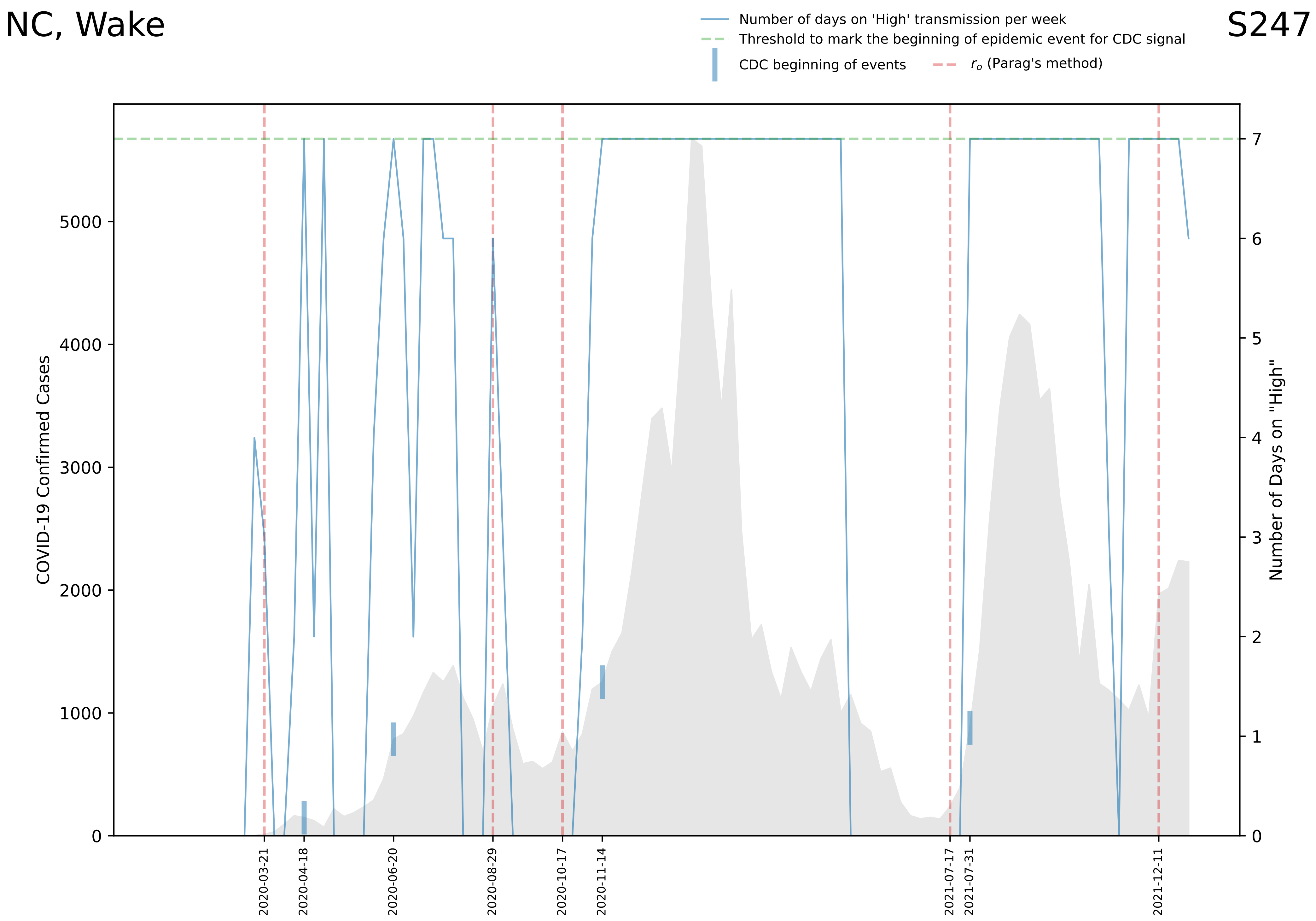

NC, Wake
S247
Number of days on 'High' transmission per week
Threshold to mark the beginning of epidemic event for CDC signal
CDC beginning of events
r_o (Parag's method)
COVID-19 Confirmed Cases
Number of Days on "High"
0
1000
2000
3000
4000
5000
0
1
2
3
4
5
6
7
2020-03-21
2020-04-18
2020-06-20
2020-08-29
2020-10-17
2020-11-14
2021-07-17
2021-07-31
2021-12-11

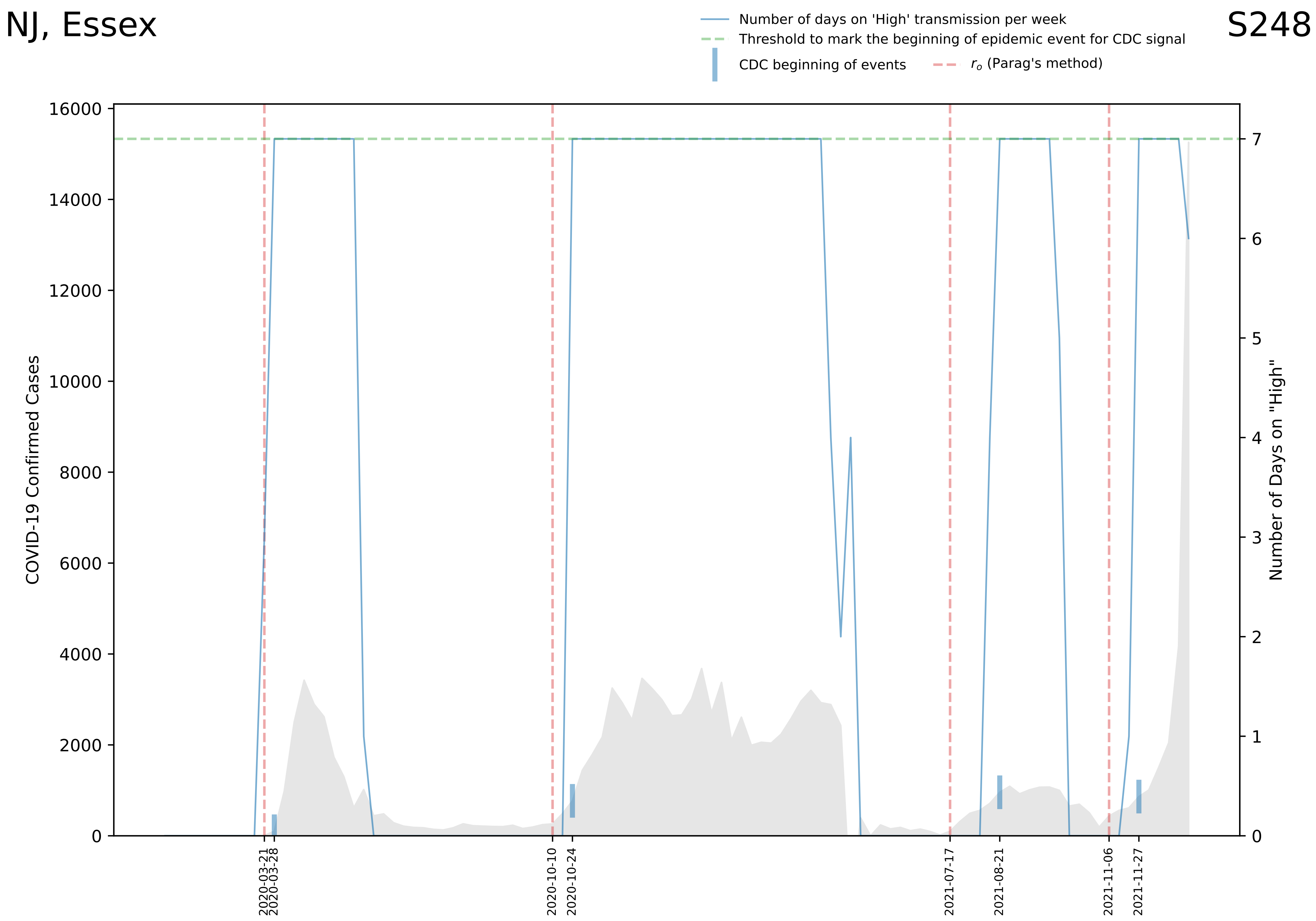

NJ, Essex
S248
Number of days on 'High' transmission per week
Threshold to mark the beginning of epidemic event for CDC signal
CDC beginning of events
r_o (Parag's method)
COVID-19 Confirmed Cases
Number of Days on "High"
16000
14000
12000
10000
8000
6000
4000
2000
0
7
6
5
4
3
2
1
0
2020-03-21
2020-03-28
2020-10-10
2020-10-24
2021-07-17
2021-08-21
2021-11-06
2021-11-27

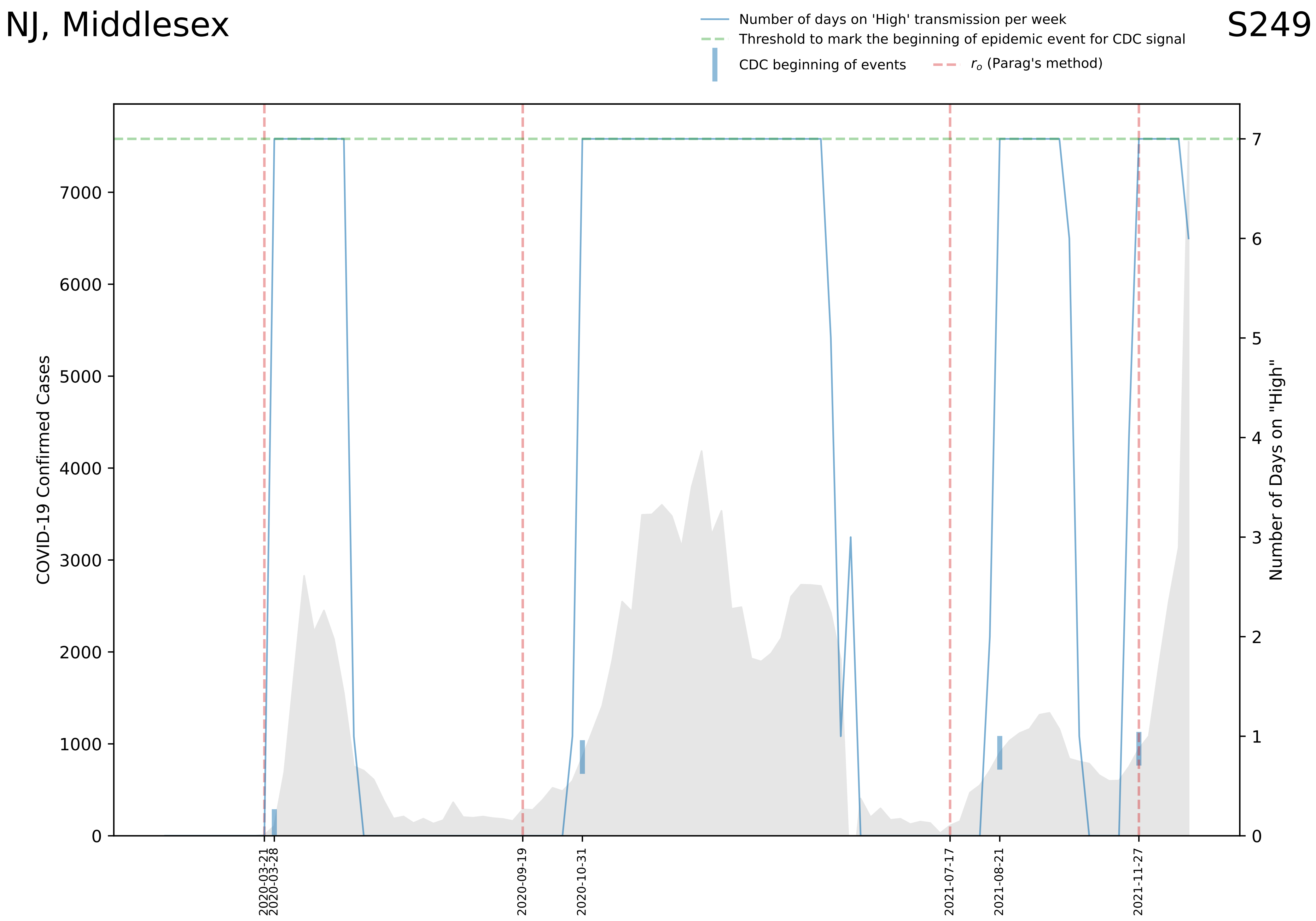

NJ, Middlesex
S249
Number of days on 'High' transmission per week
Threshold to mark the beginning of epidemic event for CDC signal
CDC beginning of events
r_o (Parag's method)
COVID-19 Confirmed Cases
Number of Days on "High"
0
1000
2000
3000
4000
5000
6000
7000
0
1
2
3
4
5
6
7
2020-03-21
2020-03-28
2020-09-19
2020-10-31
2021-07-17
2021-08-21
2021-11-27

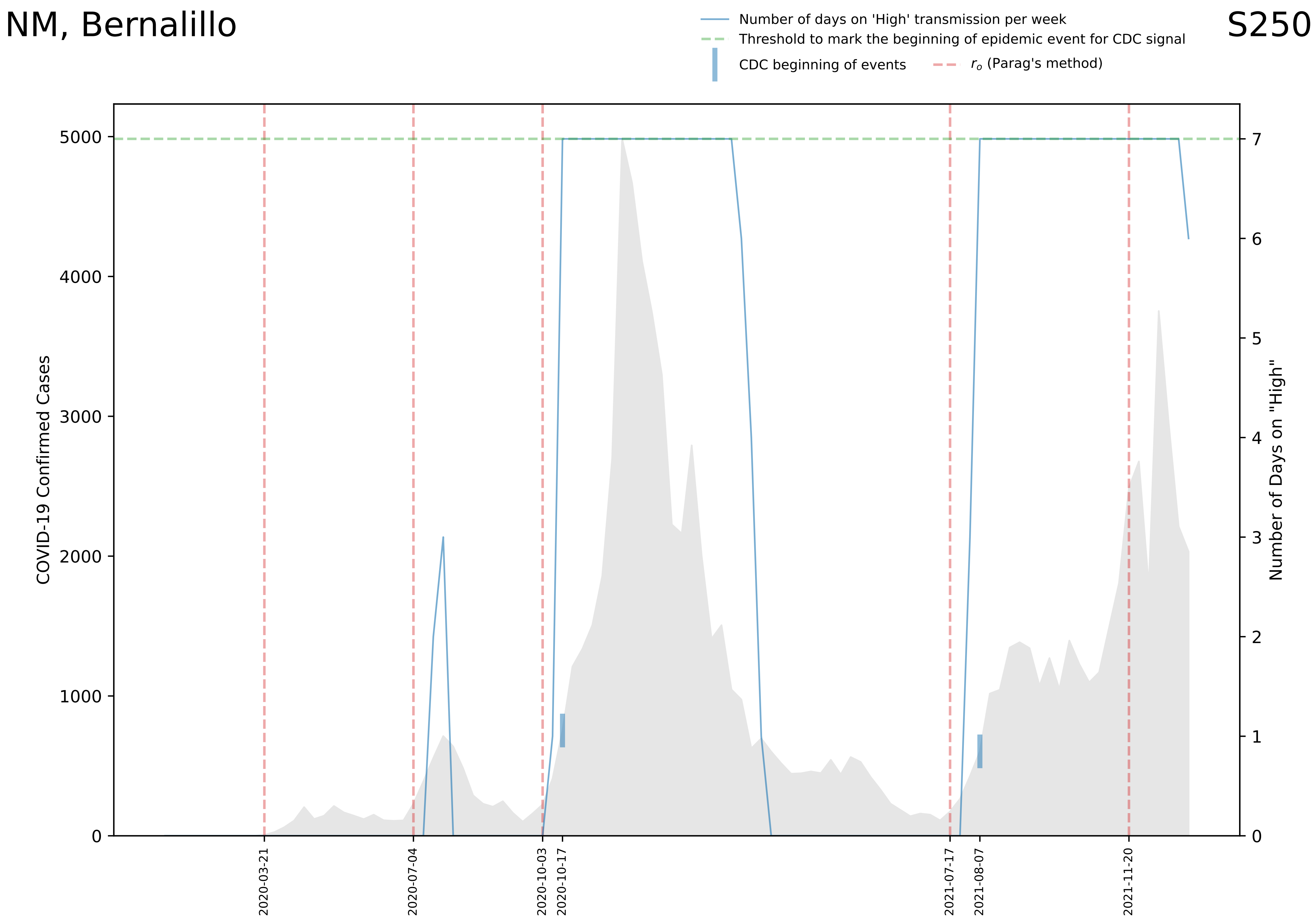

NM, Bernalillo
S250
Number of days on 'High' transmission per week
Threshold to mark the beginning of epidemic event for CDC signal
CDC beginning of events
r_o (Parag's method)
COVID-19 Confirmed Cases
Number of Days on "High"
0
1000
2000
3000
4000
5000
0
1
2
3
4
5
6
7
2020-03-21
2020-07-04
2020-10-03
2020-10-17
2021-07-17
2021-08-07
2021-11-20

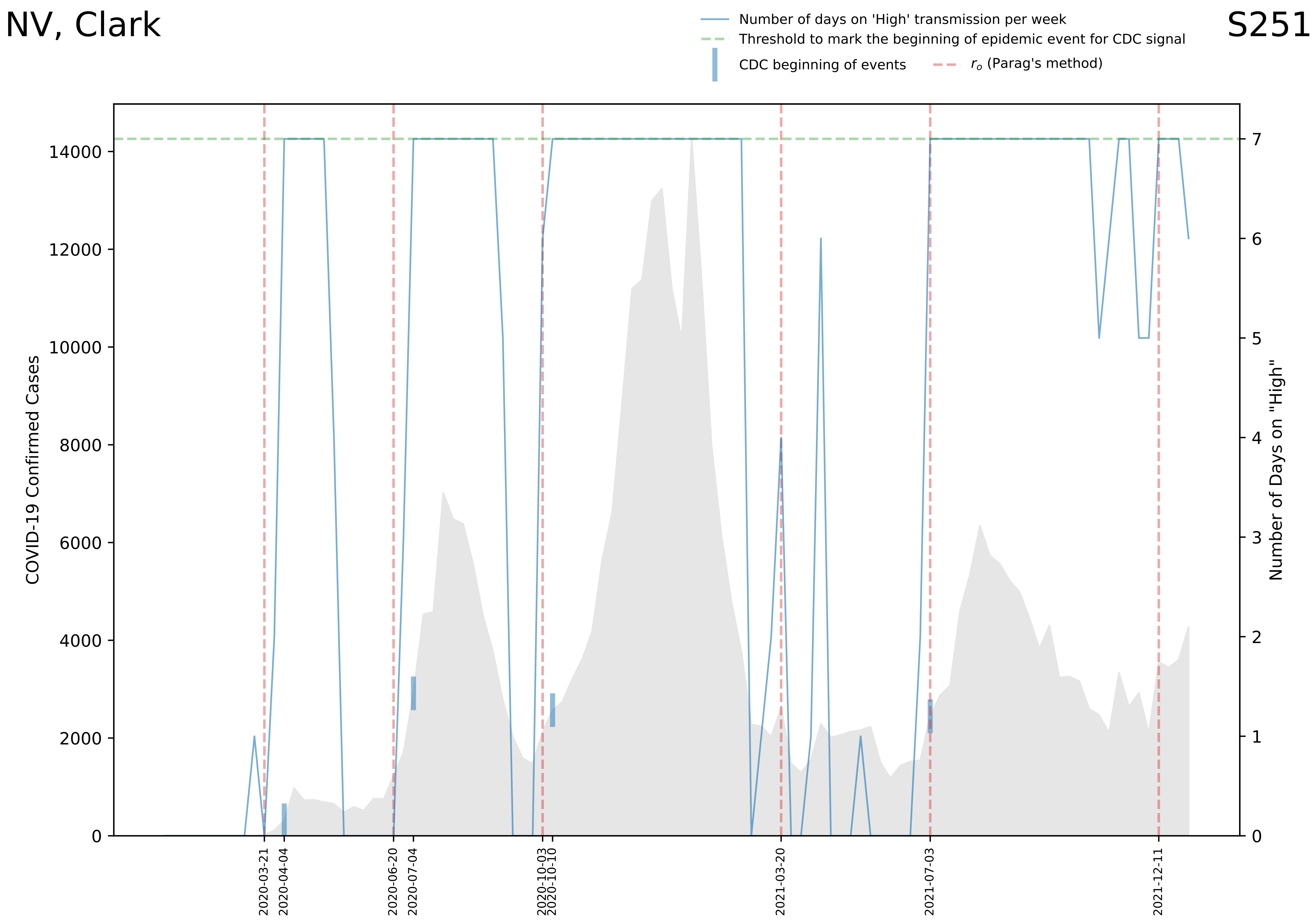

NV, Clark
S251
Number of days on 'High' transmission per week
Threshold to mark the beginning of epidemic event for CDC signal
CDC beginning of events
r_o (Parag's method)
COVID-19 Confirmed Cases
Number of Days on "High"
0
2000
4000
6000
8000
10000
12000
14000
0
1
2
3
4
5
6
7
2020-03-21
2020-04-04
2020-06-20
2020-07-04
2020-10-03
2020-10-10
2021-03-20
2021-07-03
2021-12-11

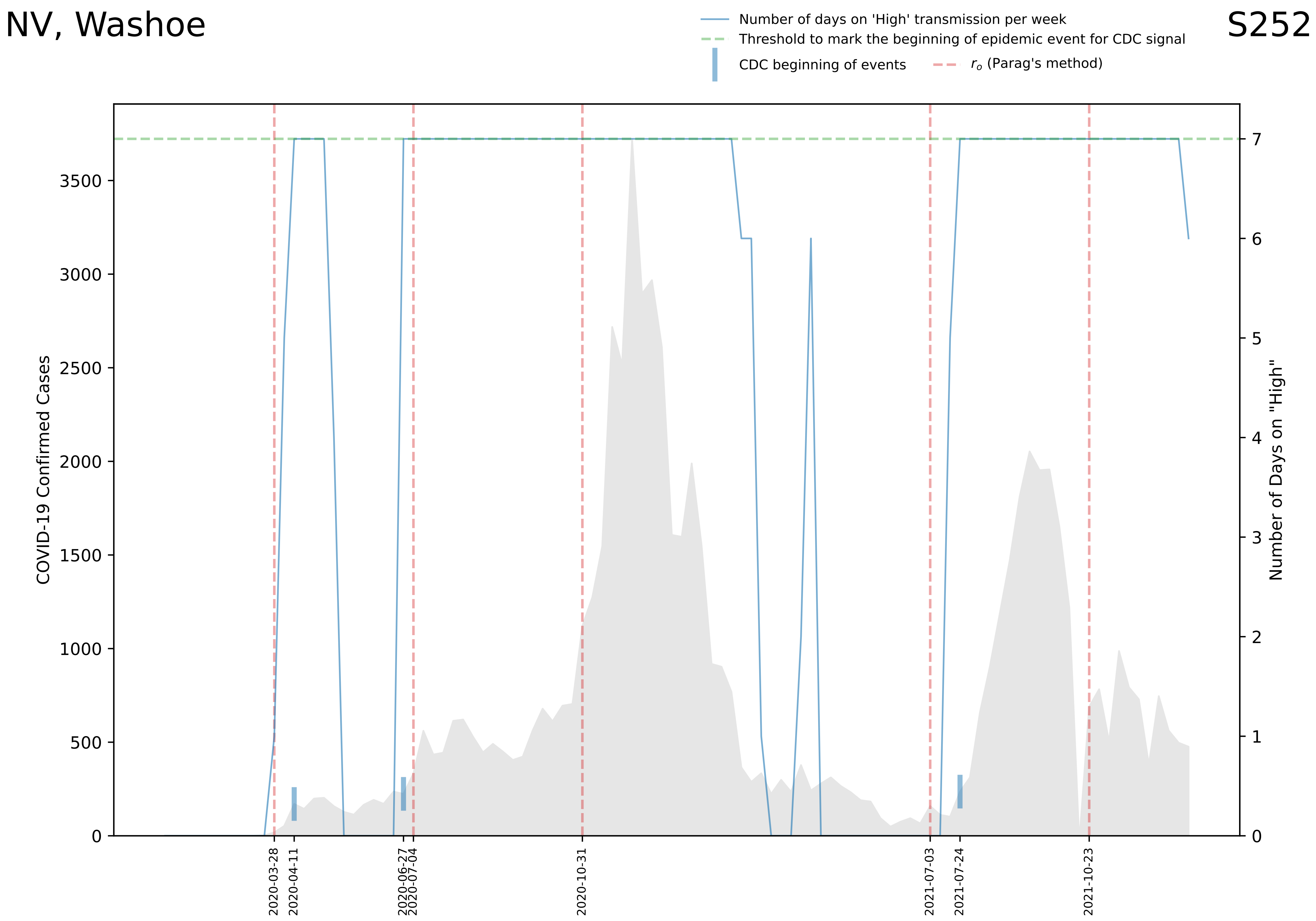

NV, Washoe
S252
Number of days on 'High' transmission per week
Threshold to mark the beginning of epidemic event for CDC signal
CDC beginning of events
r_o (Parag's method)
COVID-19 Confirmed Cases
Number of Days on "High"
0
500
1000
1500
2000
2500
3000
3500
0
1
2
3
4
5
6
7
2020-03-28
2020-04-11
2020-06-27
2020-07-04
2020-10-31
2021-07-03
2021-07-24
2021-10-23

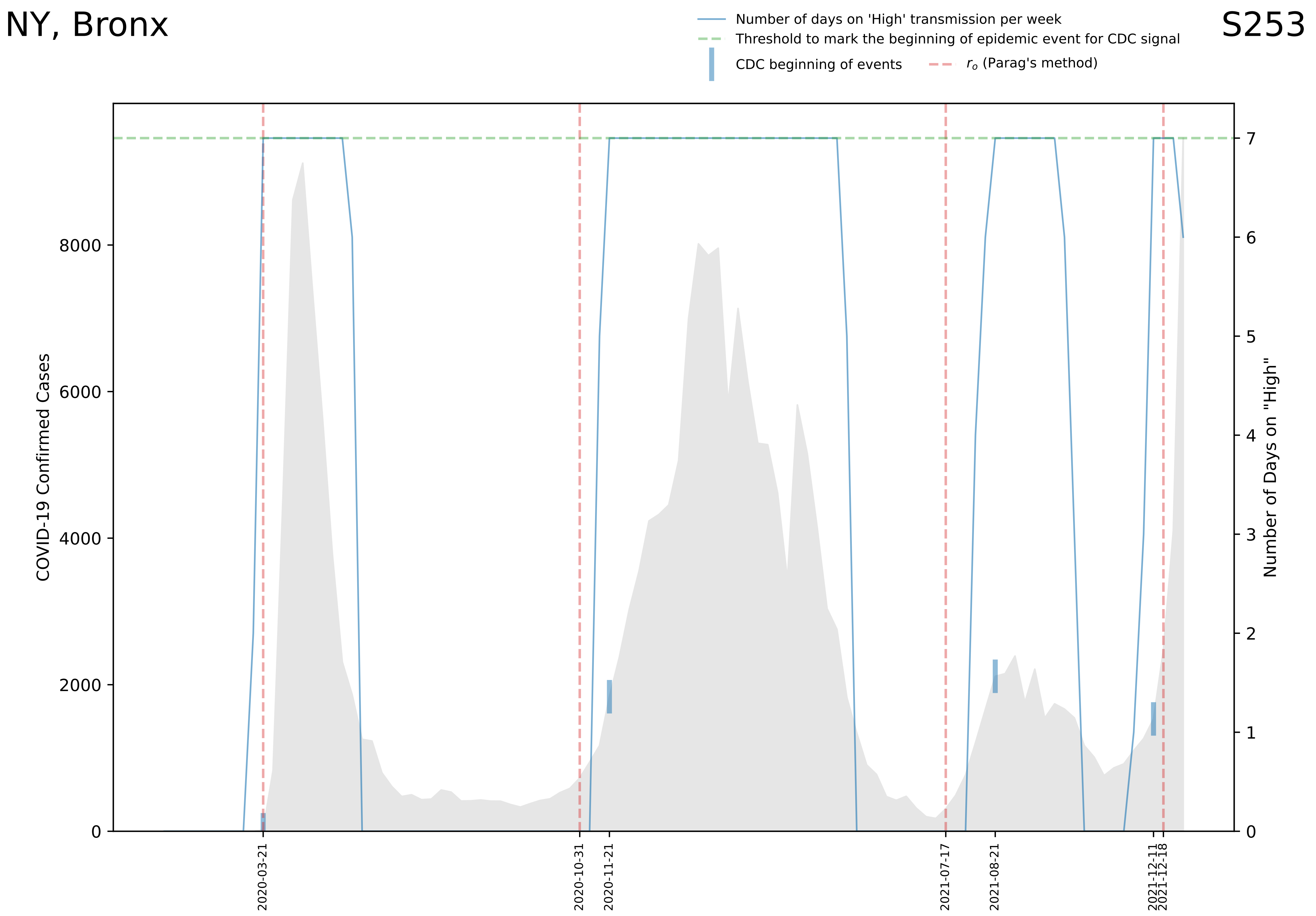
NY, Bronx
S253
Number of days on 'High' transmission per week
Threshold to mark the beginning of epidemic event for CDC signal
CDC beginning of events
r_o (Parag's method)
COVID-19 Confirmed Cases
Number of Days on "High"
0
2000
4000
6000
8000
0
1
2
3
4
5
6
7
2020-03-21
2020-10-31
2020-11-21
2021-07-17
2021-08-21
2021-12-11
2021-12-18

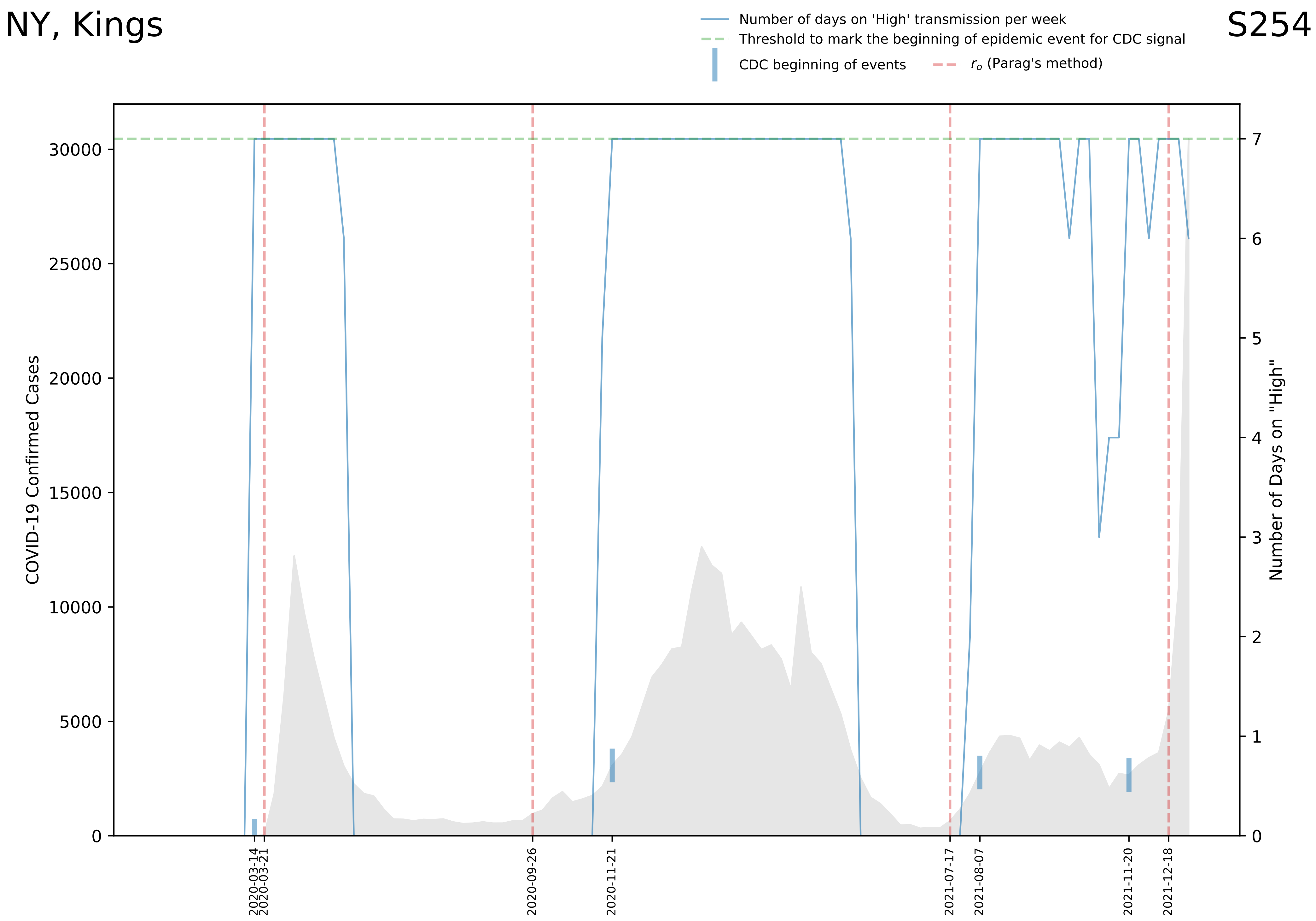

NY, Kings
S254
Number of days on 'High' transmission per week
Threshold to mark the beginning of epidemic event for CDC signal
CDC beginning of events
r_o (Parag's method)
COVID-19 Confirmed Cases
Number of Days on "High"
0
5000
10000
15000
20000
25000
30000
0
1
2
3
4
5
6
7
2020-03-14
2020-03-21
2020-09-26
2020-11-21
2021-07-17
2021-08-07
2021-11-20
2021-12-18

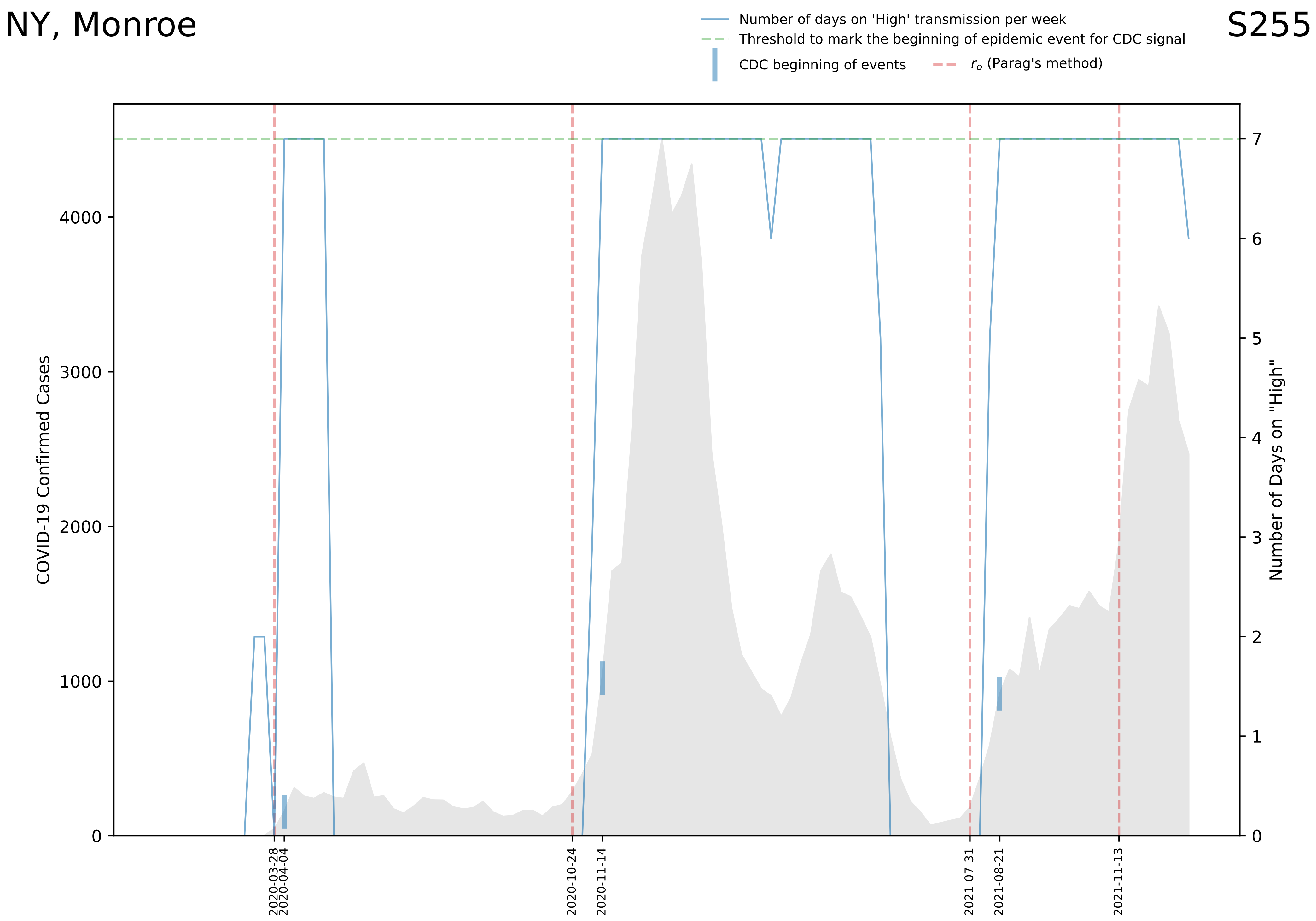
NY, Monroe
S255
Number of days on 'High' transmission per week
Threshold to mark the beginning of epidemic event for CDC signal
CDC beginning of events
r_o (Parag's method)
COVID-19 Confirmed Cases
Number of Days on "High"
0
1000
2000
3000
4000
0
1
2
3
4
5
6
7
2020-03-28
2020-04-04
2020-10-24
2020-11-14
2021-07-31
2021-08-21
2021-11-13

NY, Nassau

S256

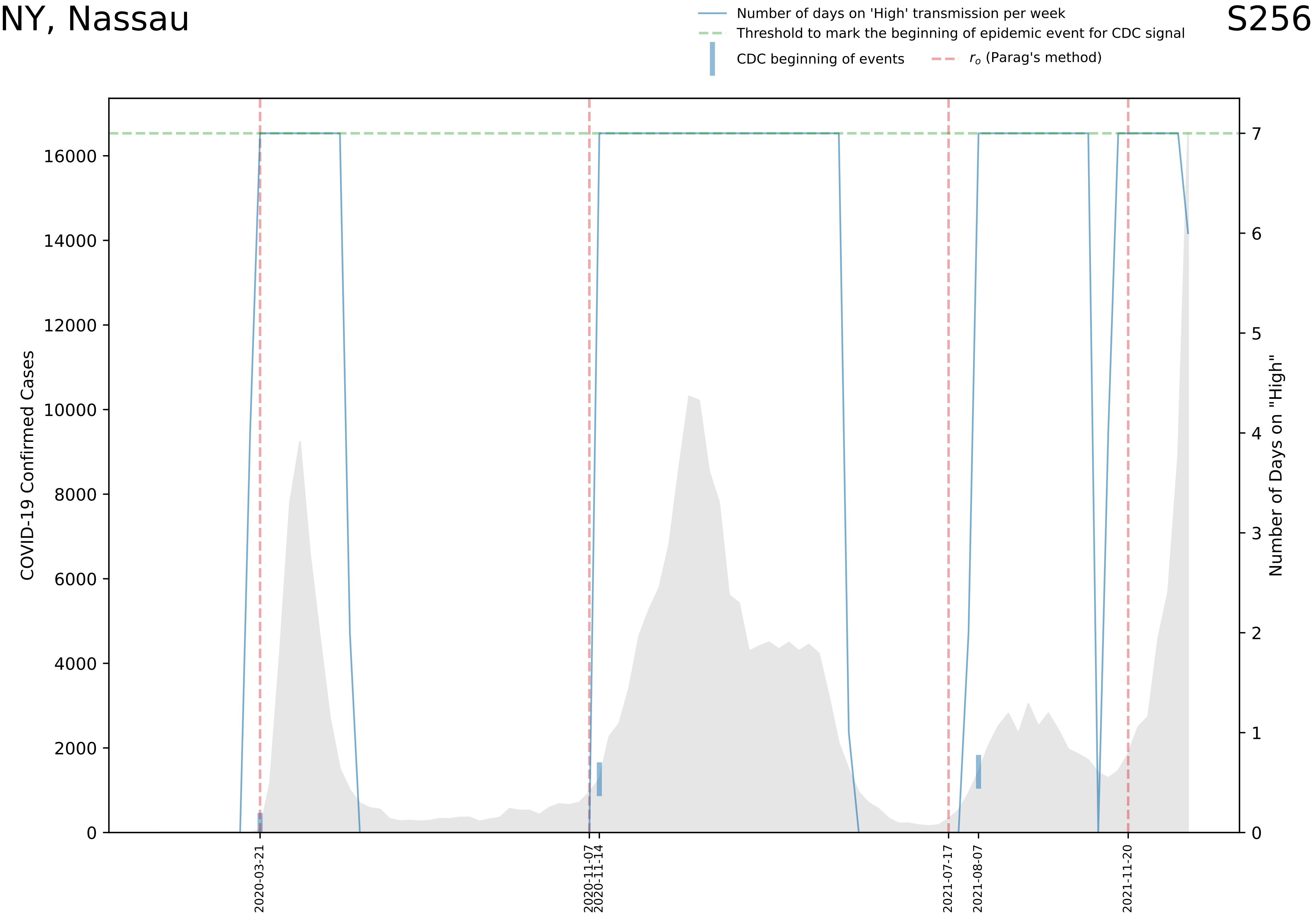

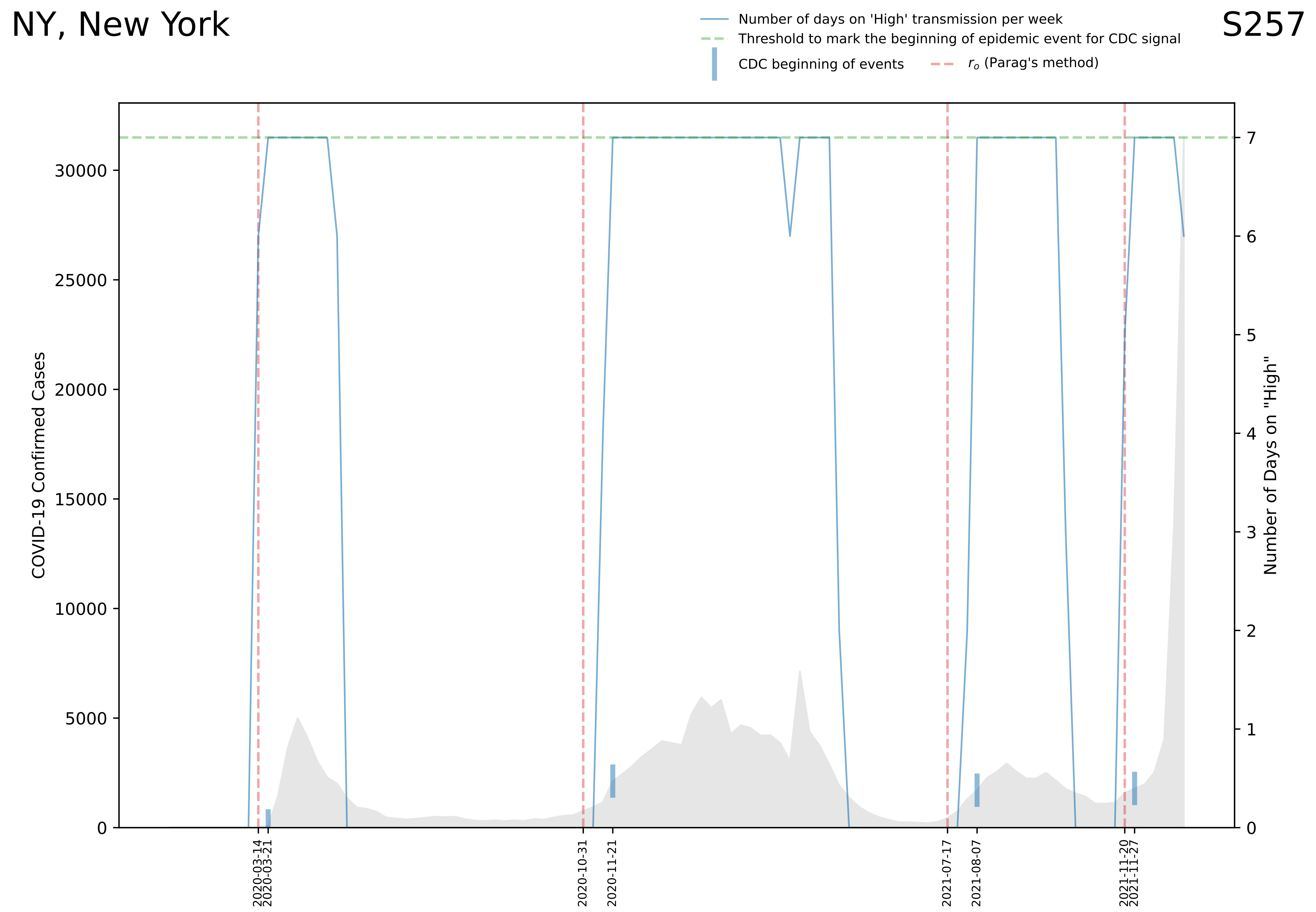

NY, New York
S257
Number of days on 'High' transmission per week
Threshold to mark the beginning of epidemic event for CDC signal
CDC beginning of events
r_o (Parag's method)
COVID-19 Confirmed Cases
Number of Days on "High"
0
5000
10000
15000
20000
25000
30000
0
1
2
3
4
5
6
7
2020-03-14
2020-03-21
2020-10-31
2020-11-21
2021-07-17
2021-08-07
2021-11-20
2021-11-27

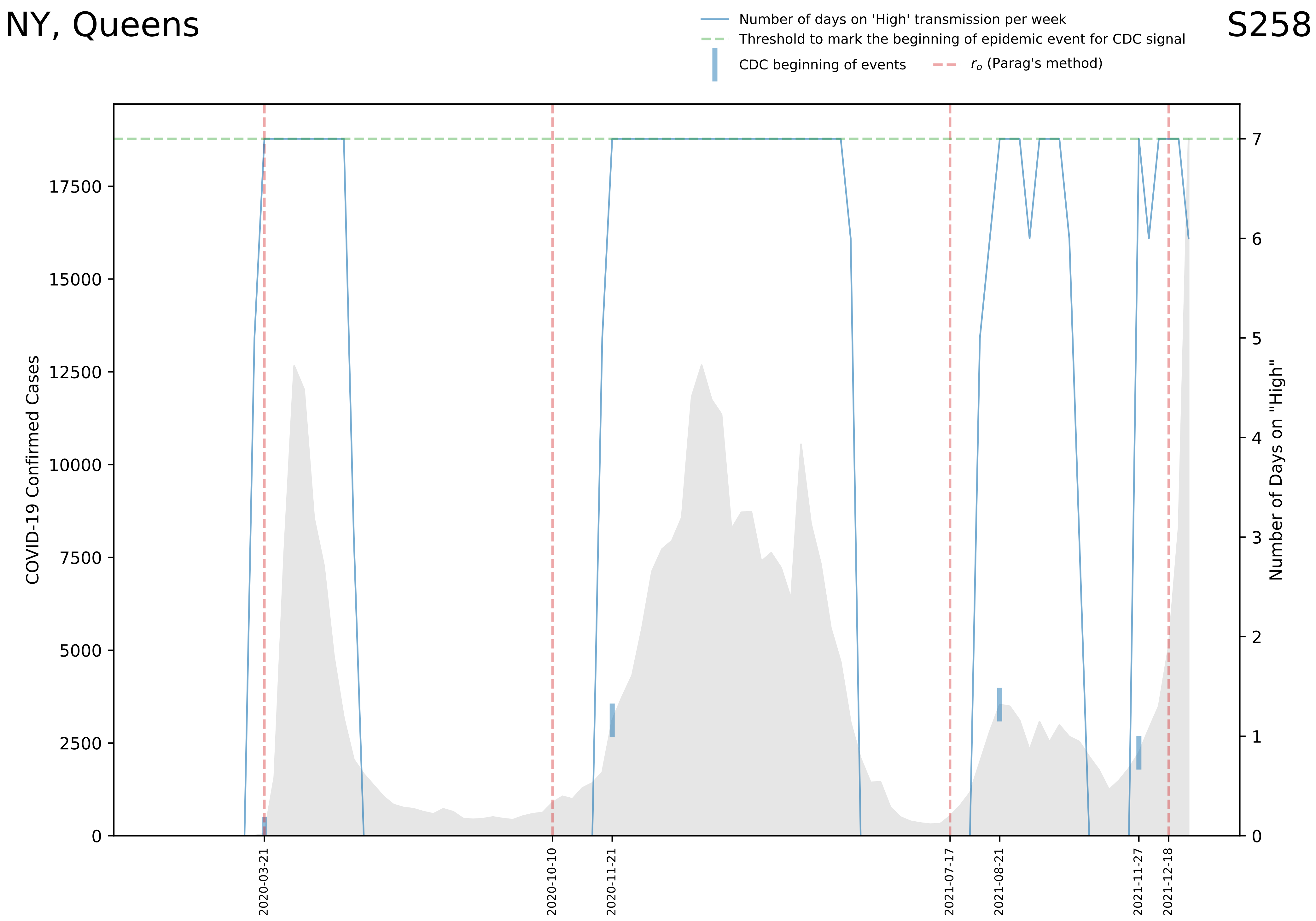

NY, Queens
S258
Number of days on 'High' transmission per week
Threshold to mark the beginning of epidemic event for CDC signal
CDC beginning of events
r_o (Parag's method)
COVID-19 Confirmed Cases
Number of Days on "High"
0
2500
5000
7500
10000
12500
15000
17500
0
1
2
3
4
5
6
7
2020-03-21
2020-10-10
2020-11-21
2021-07-17
2021-08-21
2021-11-27
2021-12-18

NY, Suffolk

S259

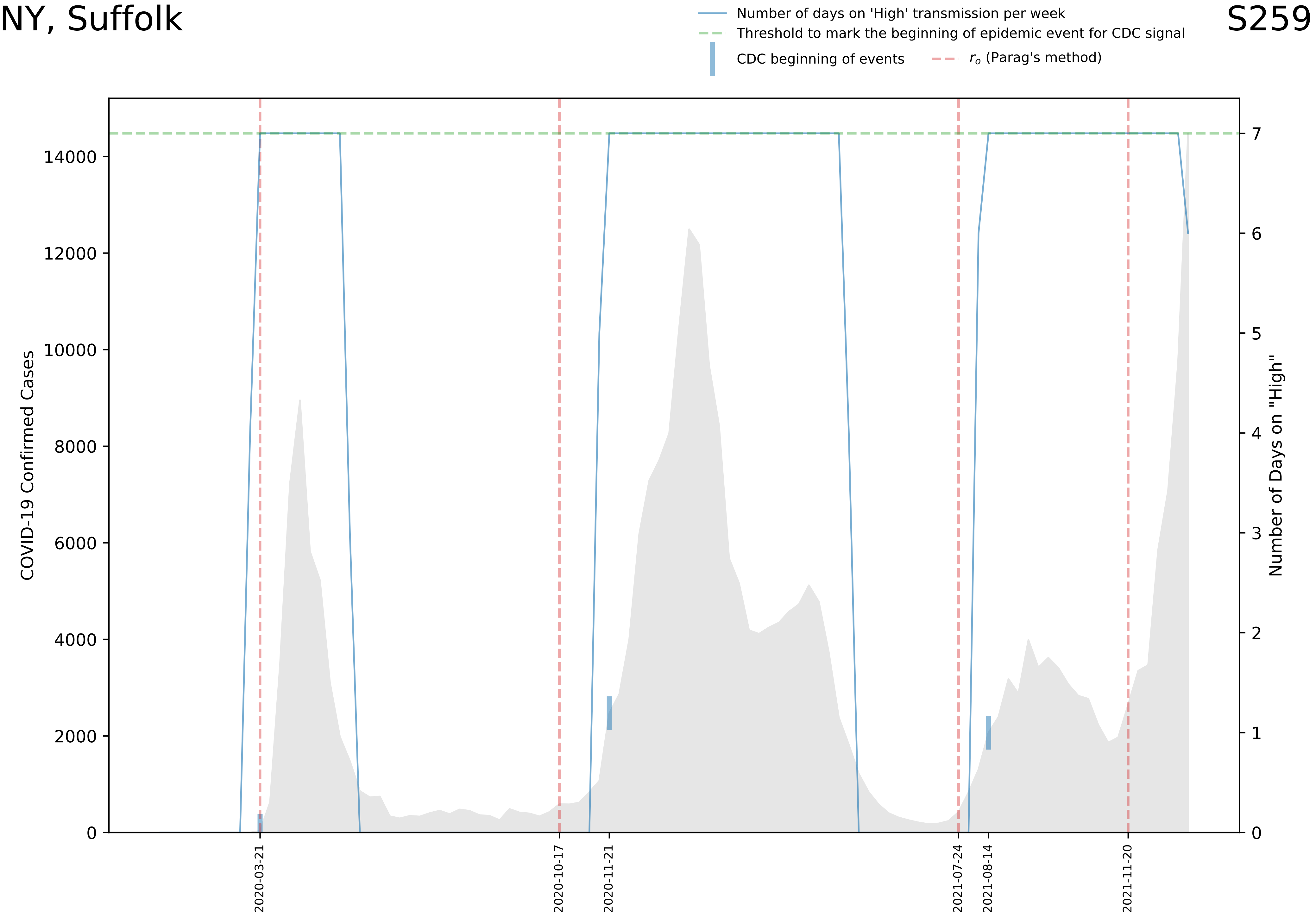

OH, Cuyahoga

S260

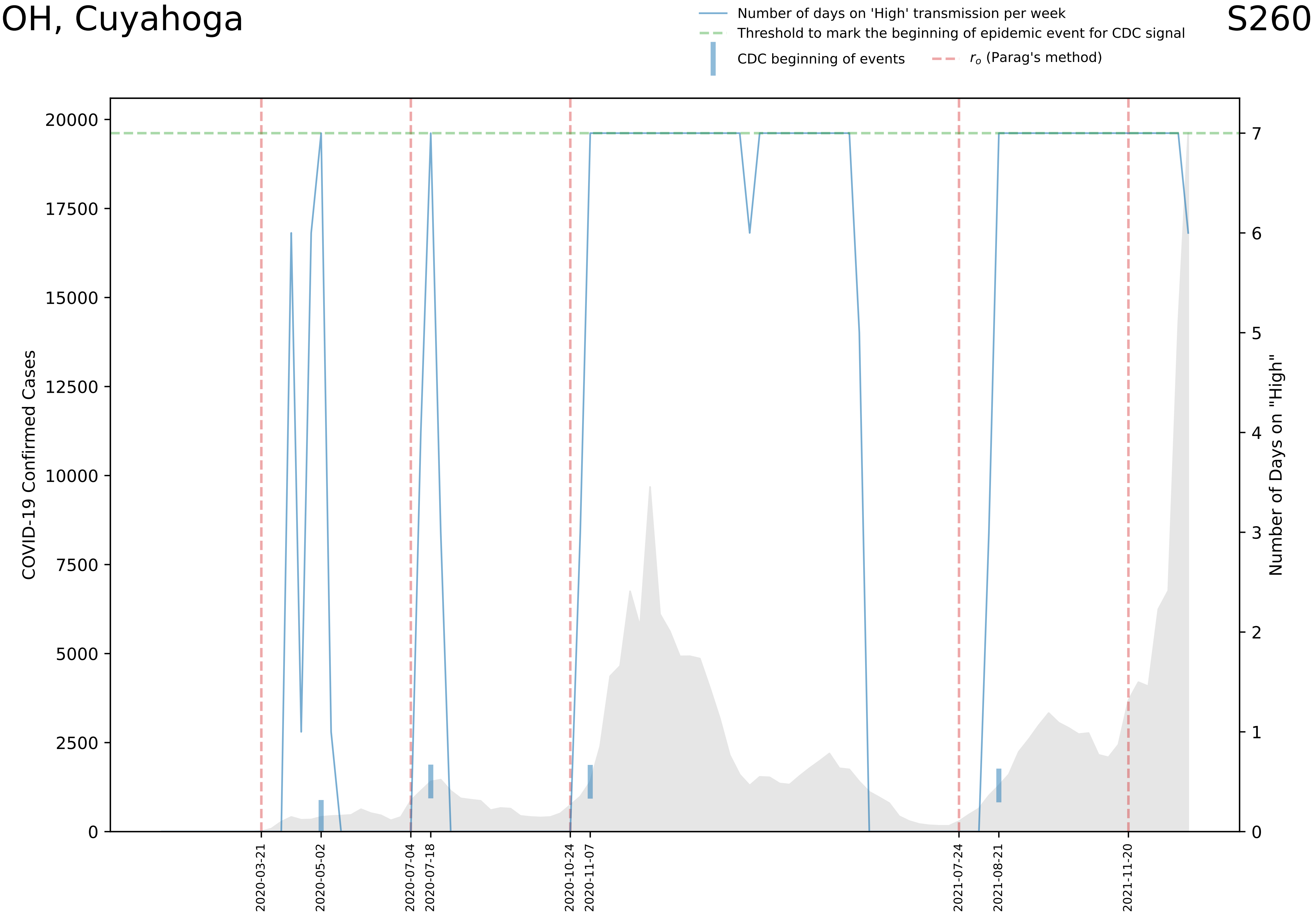

OH, Franklin

S261

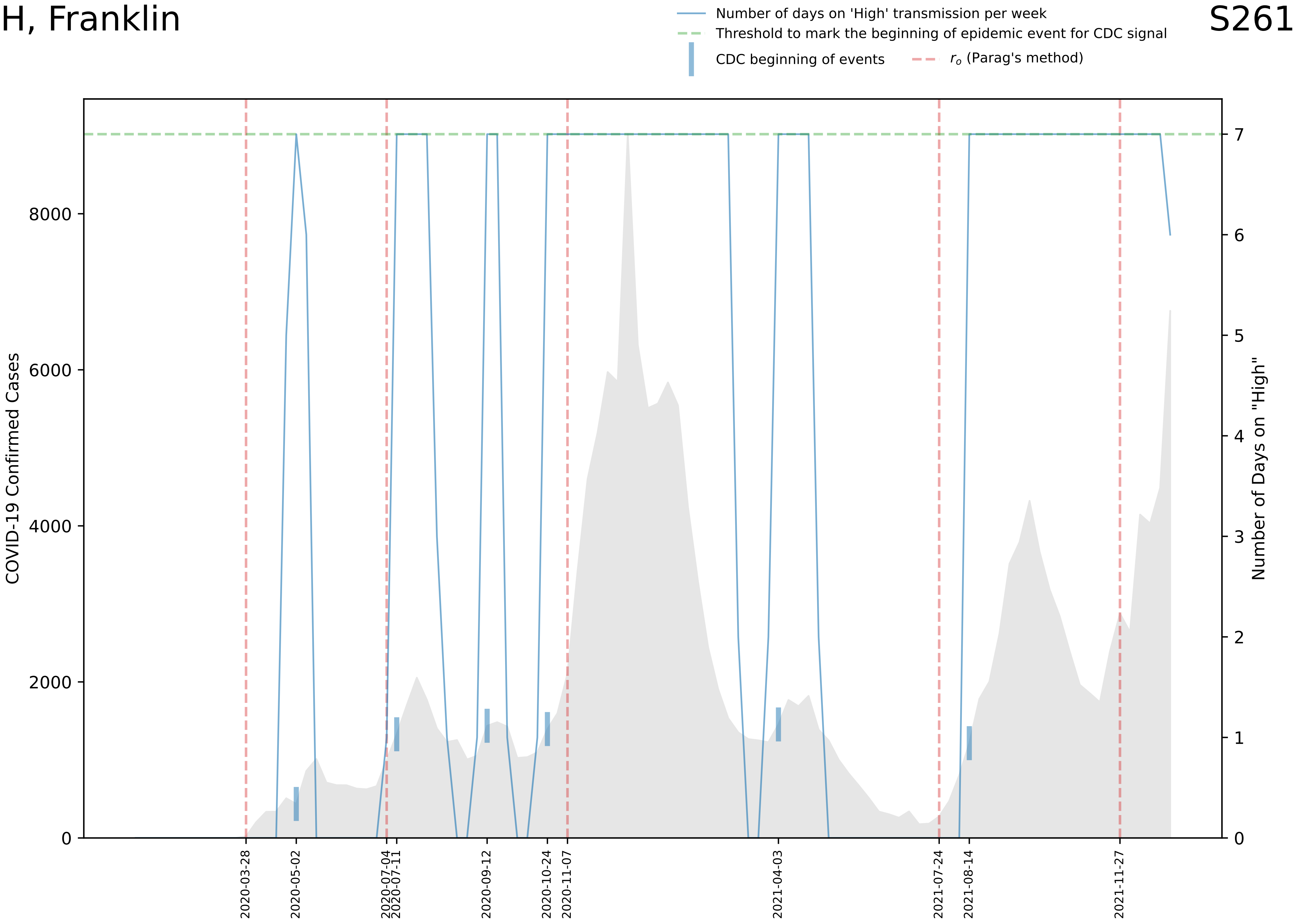

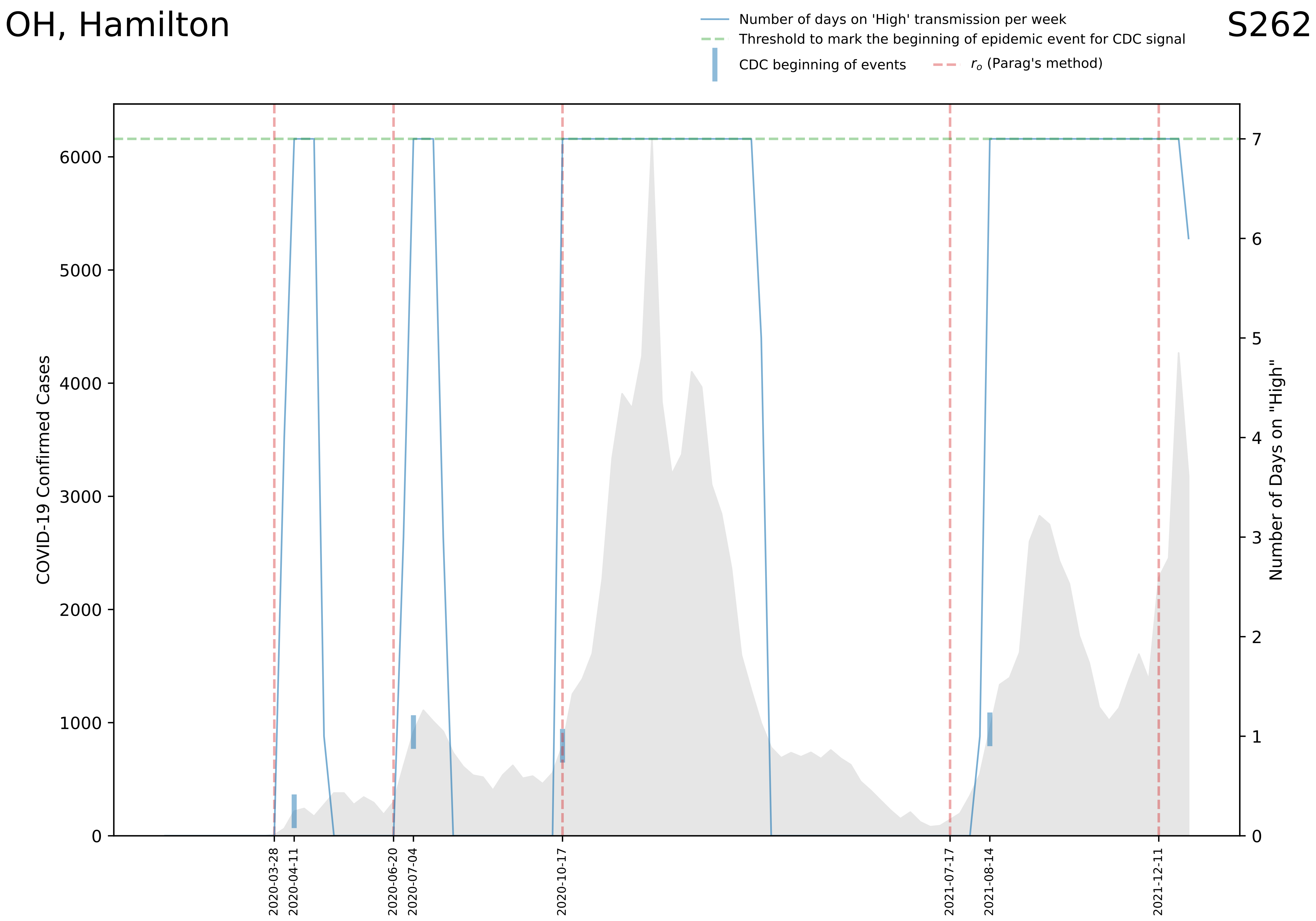

OH, Hamilton
S262
Number of days on 'High' transmission per week
Threshold to mark the beginning of epidemic event for CDC signal
CDC beginning of events
r_o (Parag's method)
COVID-19 Confirmed Cases
Number of Days on "High"
0
1000
2000
3000
4000
5000
6000
0
1
2
3
4
5
6
7
2020-03-28
2020-04-11
2020-06-20
2020-07-04
2020-10-17
2021-07-17
2021-08-14
2021-12-11

OH, Summit

S263

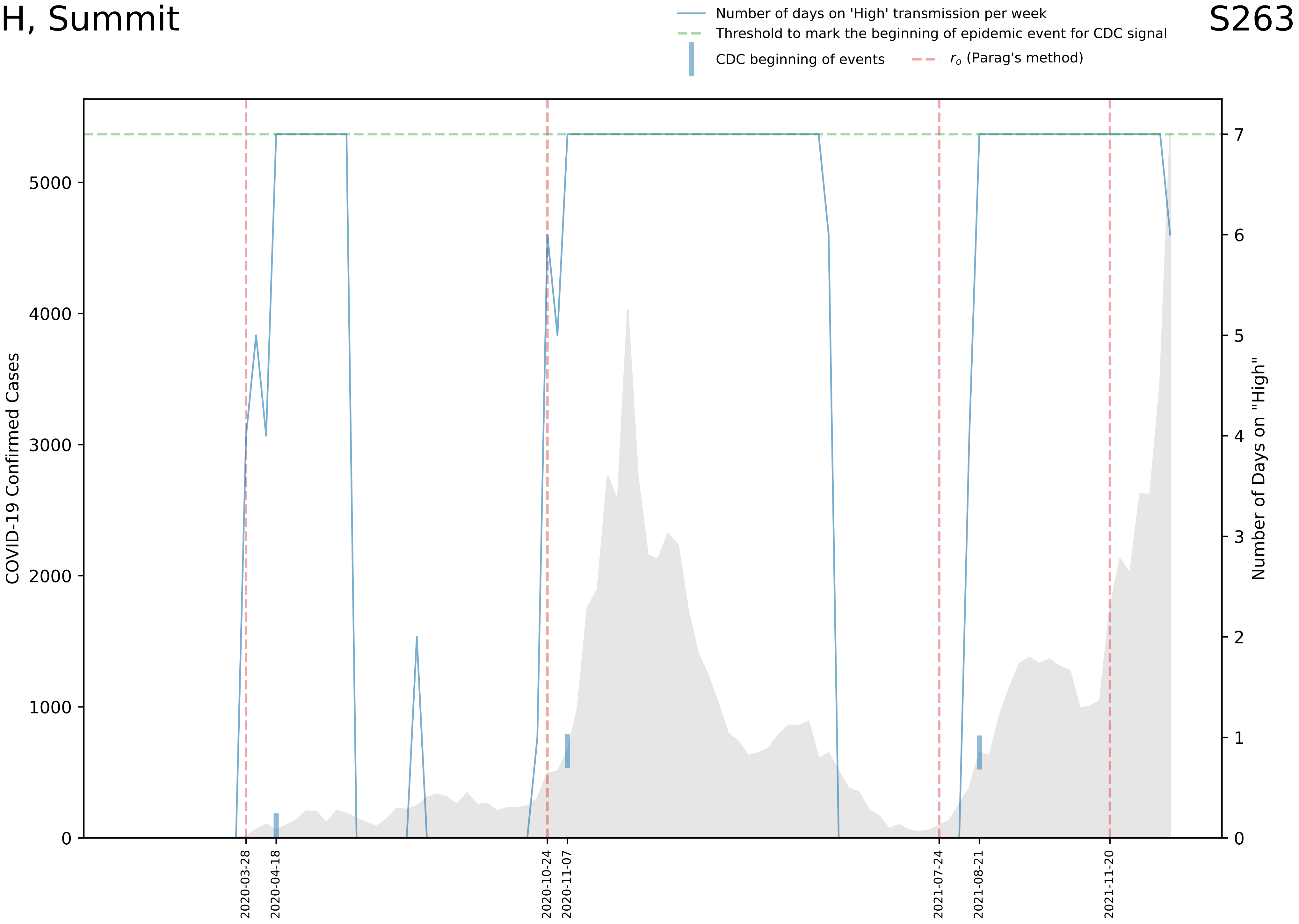

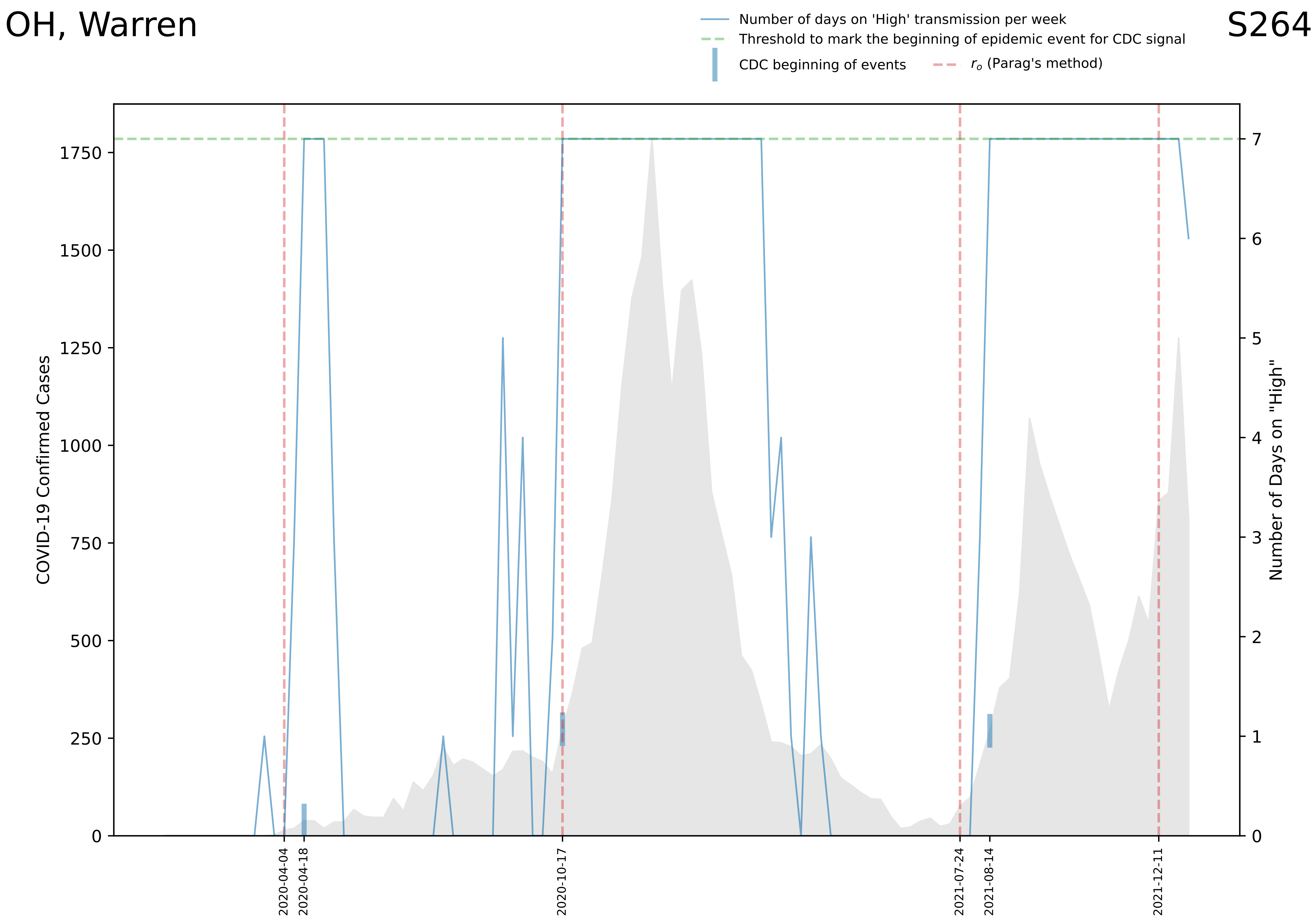

OH, Warren
S264
Number of days on 'High' transmission per week
Threshold to mark the beginning of epidemic event for CDC signal
CDC beginning of events
r_o (Parag's method)
COVID-19 Confirmed Cases
Number of Days on "High"
0
250
500
750
1000
1250
1500
1750
0
1
2
3
4
5
6
7
2020-04-04
2020-04-18
2020-10-17
2021-07-24
2021-08-14
2021-12-11

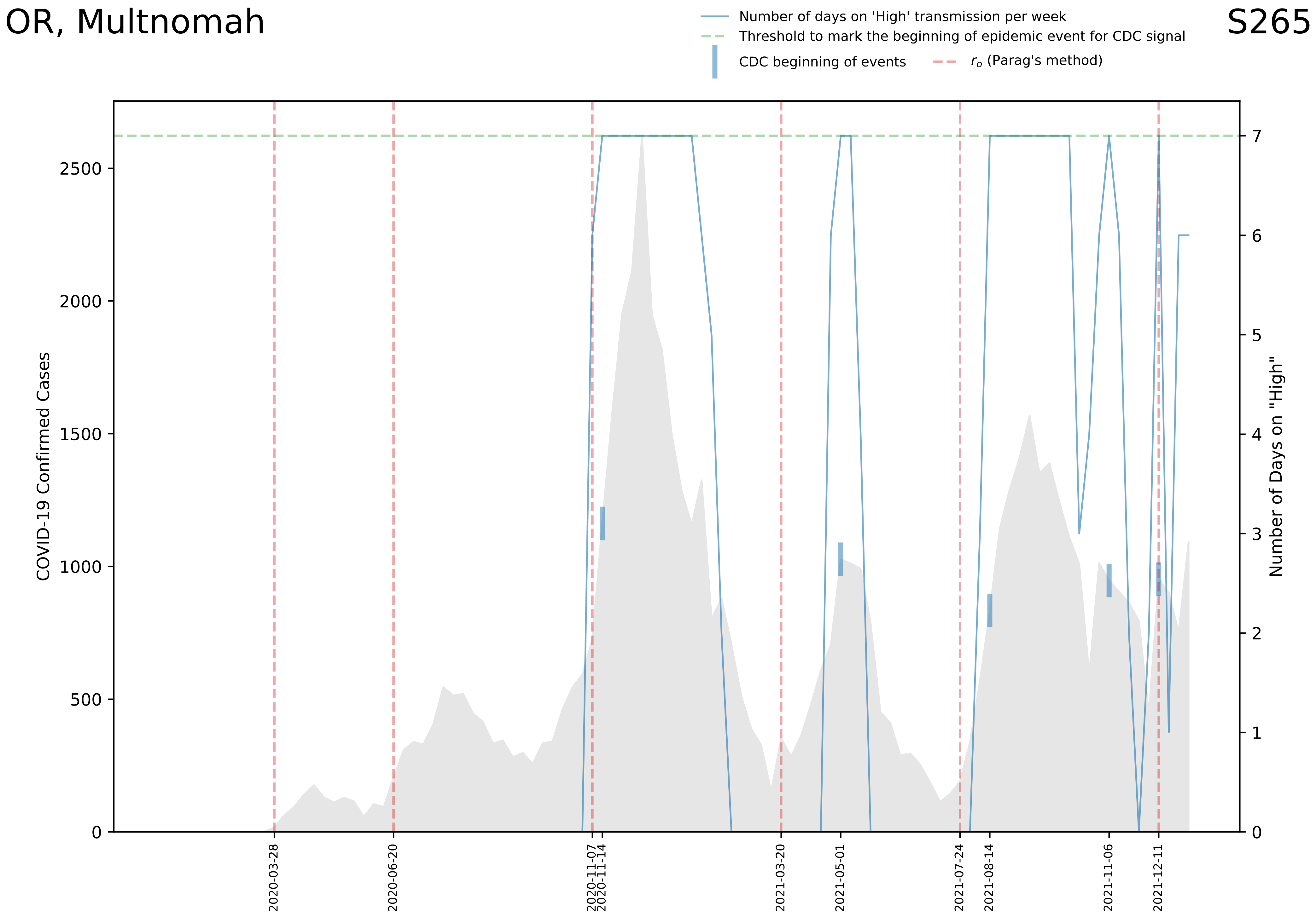

OR, Multnomah
S265
Number of days on 'High' transmission per week
Threshold to mark the beginning of epidemic event for CDC signal
CDC beginning of events
r_o (Parag's method)
COVID-19 Confirmed Cases
Number of Days on "High"
0
500
1000
1500
2000
2500
0
1
2
3
4
5
6
7
2020-03-28
2020-06-20
2020-11-07
2020-11-14
2021-03-20
2021-05-01
2021-07-24
2021-08-14
2021-11-06
2021-12-11

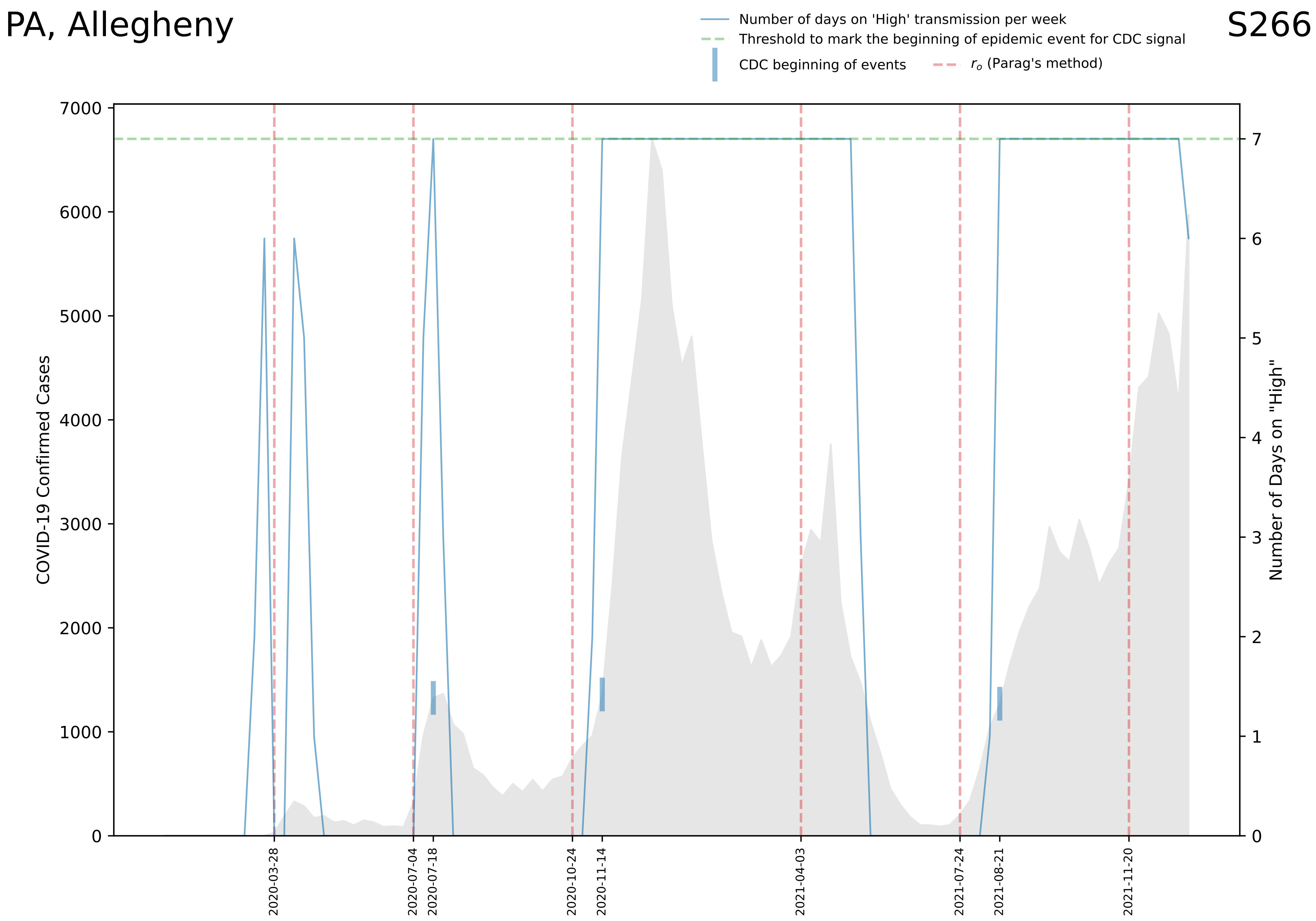
PA, Allegheny
S266
Number of days on 'High' transmission per week
Threshold to mark the beginning of epidemic event for CDC signal
CDC beginning of events
r_o (Parag's method)
COVID-19 Confirmed Cases
Number of Days on "High"
0
1000
2000
3000
4000
5000
6000
7000
0
1
2
3
4
5
6
7
2020-03-28
2020-07-04
2020-07-18
2020-10-24
2020-11-14
2021-04-03
2021-07-24
2021-08-21
2021-11-20

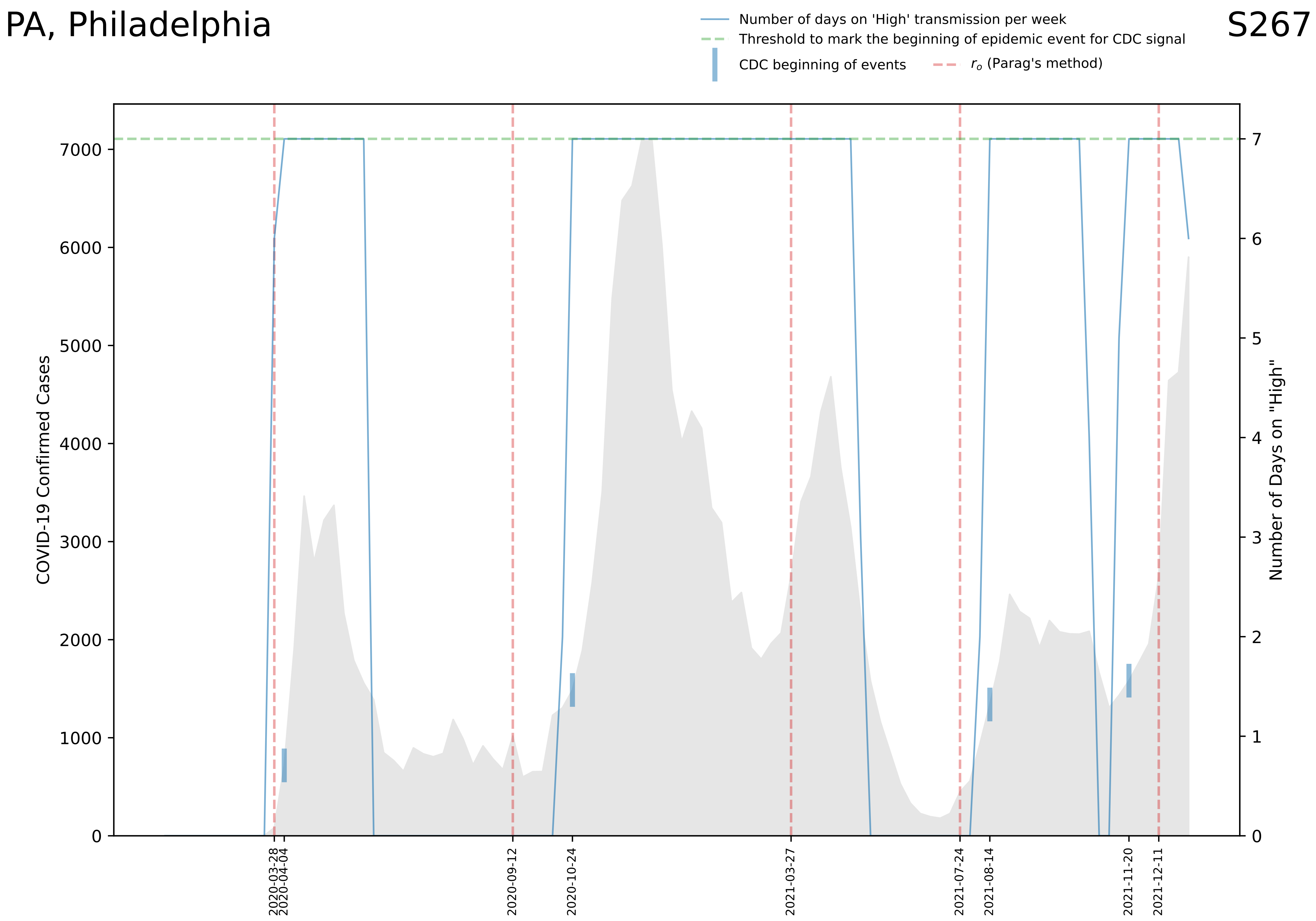

PA, Philadelphia
S267
Number of days on 'High' transmission per week
Threshold to mark the beginning of epidemic event for CDC signal
CDC beginning of events
r_o (Parag's method)
COVID-19 Confirmed Cases
Number of Days on "High"
0
1000
2000
3000
4000
5000
6000
7000
0
1
2
3
4
5
6
7
2020-03-28
2020-04-04
2020-09-12
2020-10-24
2021-03-27
2021-07-24
2021-08-14
2021-11-20
2021-12-11

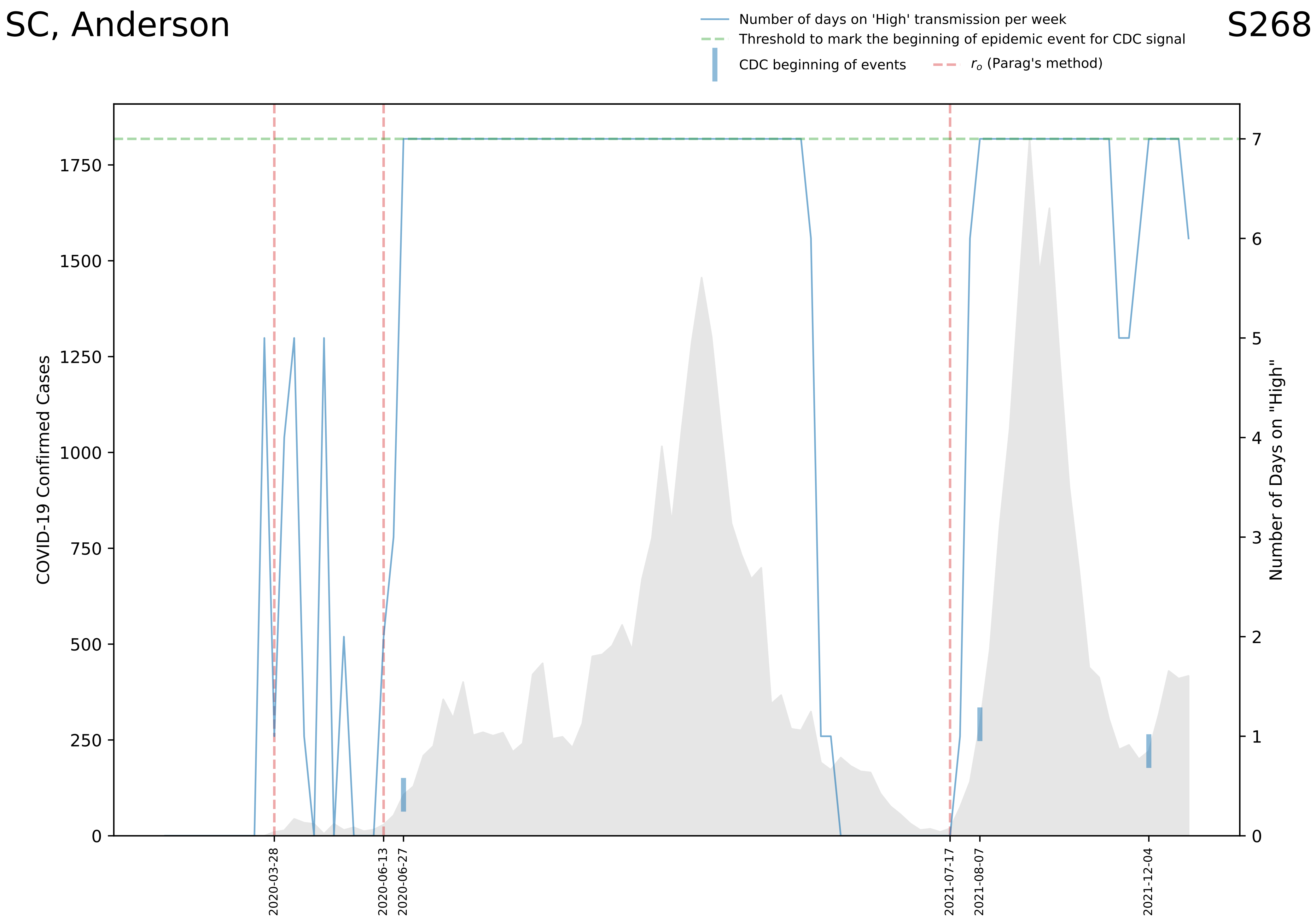

SC, Anderson
S268
Number of days on 'High' transmission per week
Threshold to mark the beginning of epidemic event for CDC signal
CDC beginning of events
r_o (Parag's method)
COVID-19 Confirmed Cases
Number of Days on "High"
0
250
500
750
1000
1250
1500
1750
0
1
2
3
4
5
6
7
2020-03-28
2020-06-13
2020-06-27
2021-07-17
2021-08-07
2021-12-04

SC, Charleston

S269

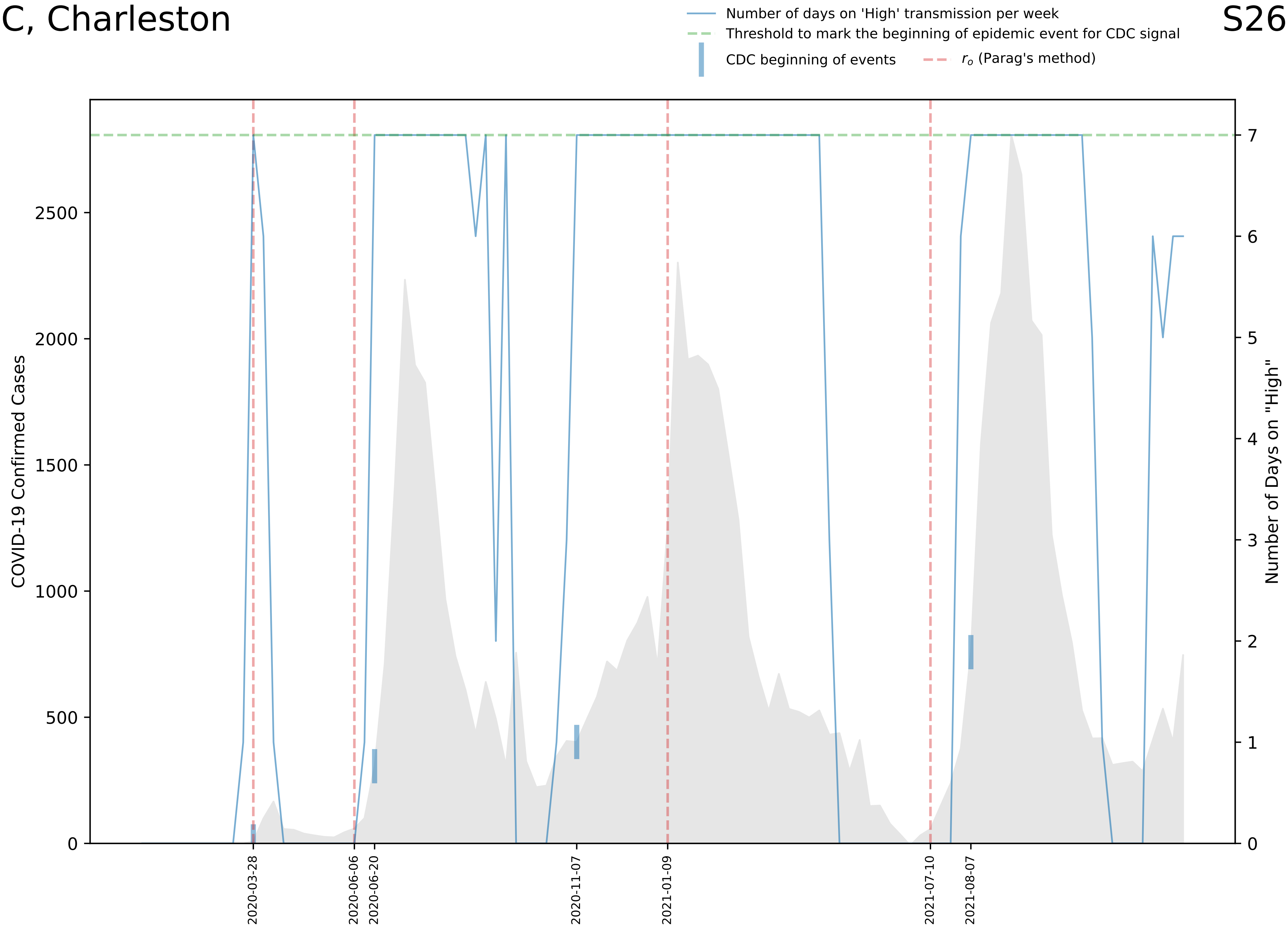

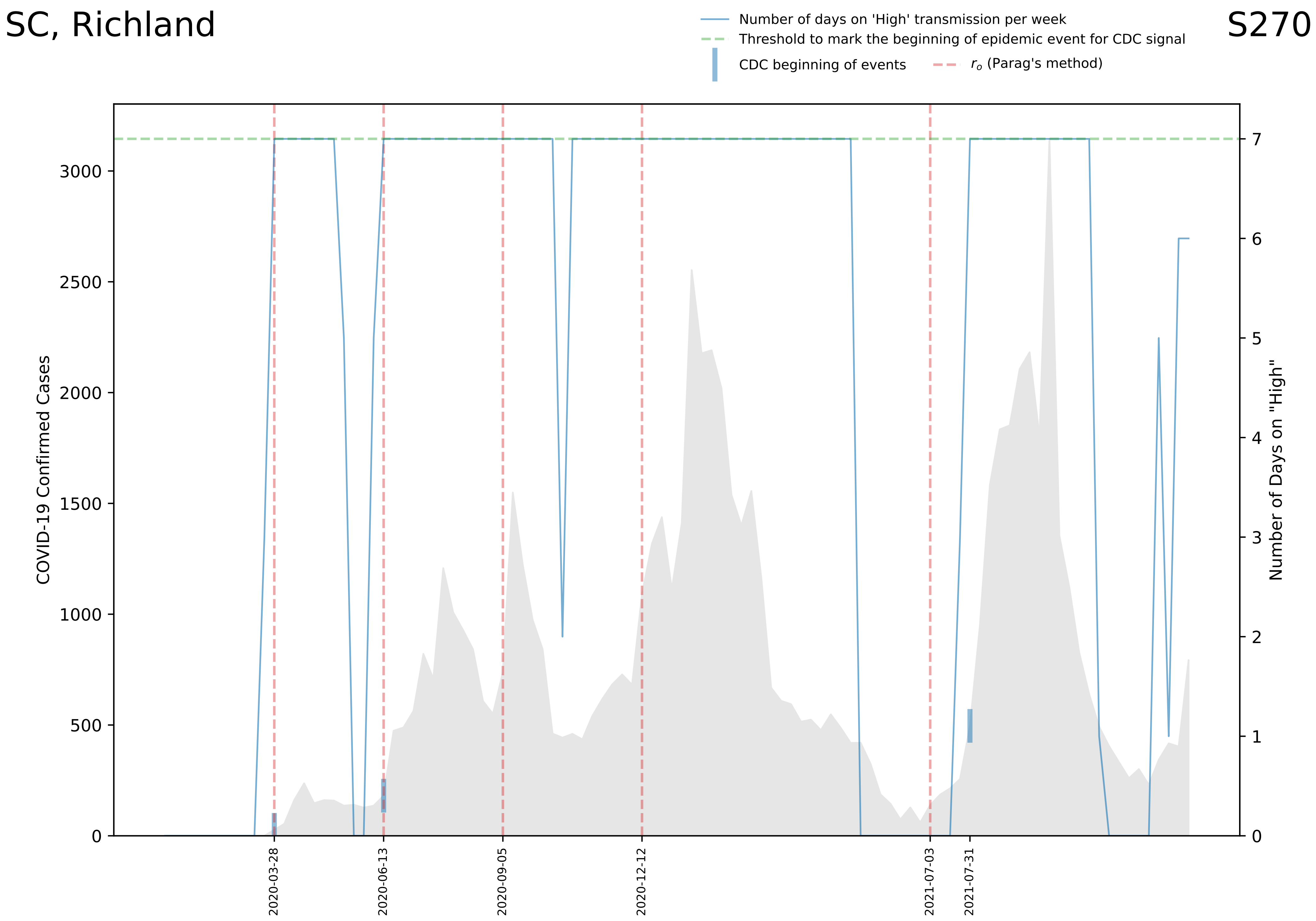

SC, Richland
S270
Number of days on 'High' transmission per week
Threshold to mark the beginning of epidemic event for CDC signal
CDC beginning of events
r_o (Parag's method)
COVID-19 Confirmed Cases
Number of Days on "High"
0
500
1000
1500
2000
2500
3000
0
1
2
3
4
5
6
7
2020-03-28
2020-06-13
2020-09-05
2020-12-12
2021-07-03
2021-07-31

TN, Blount

S271

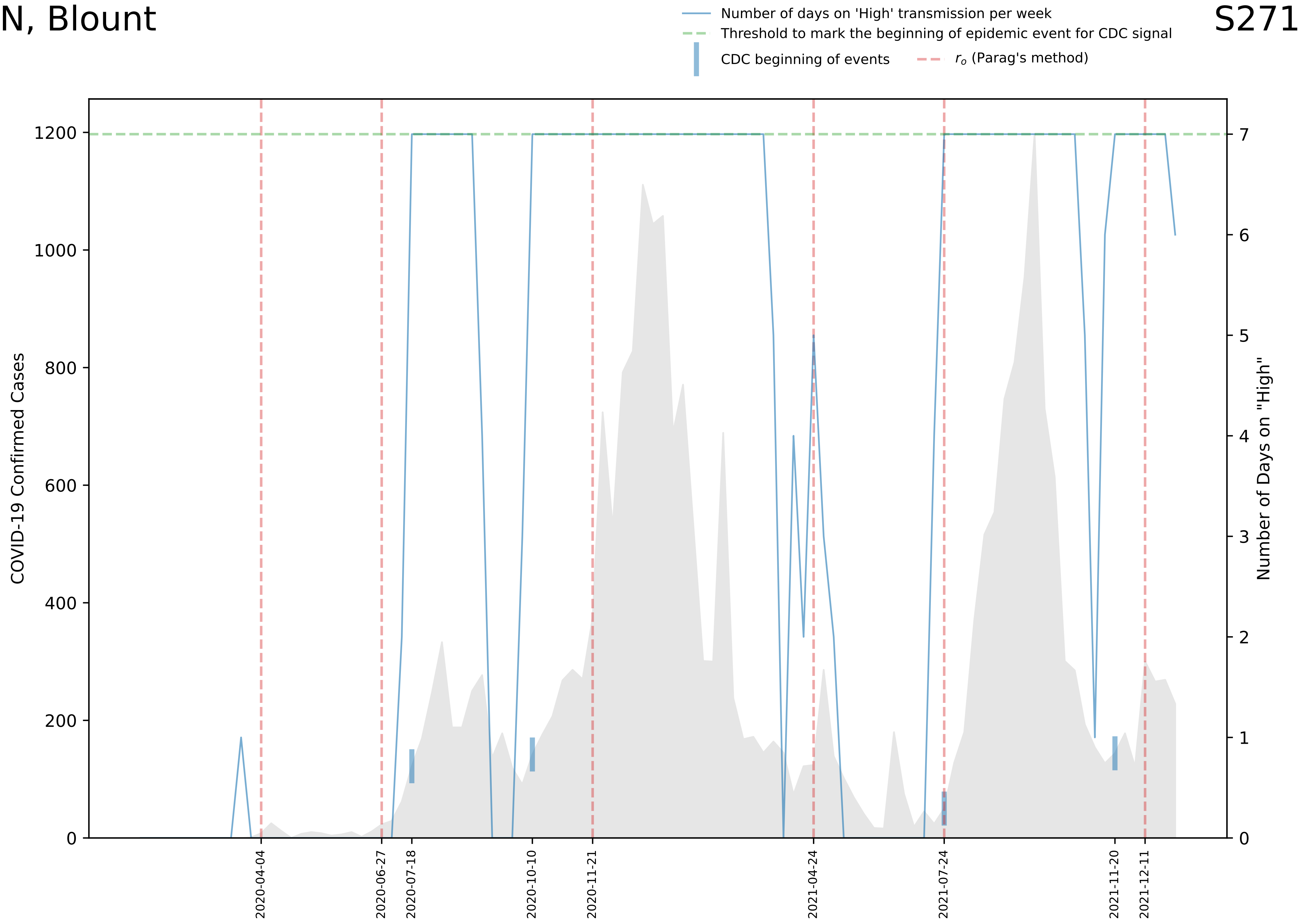

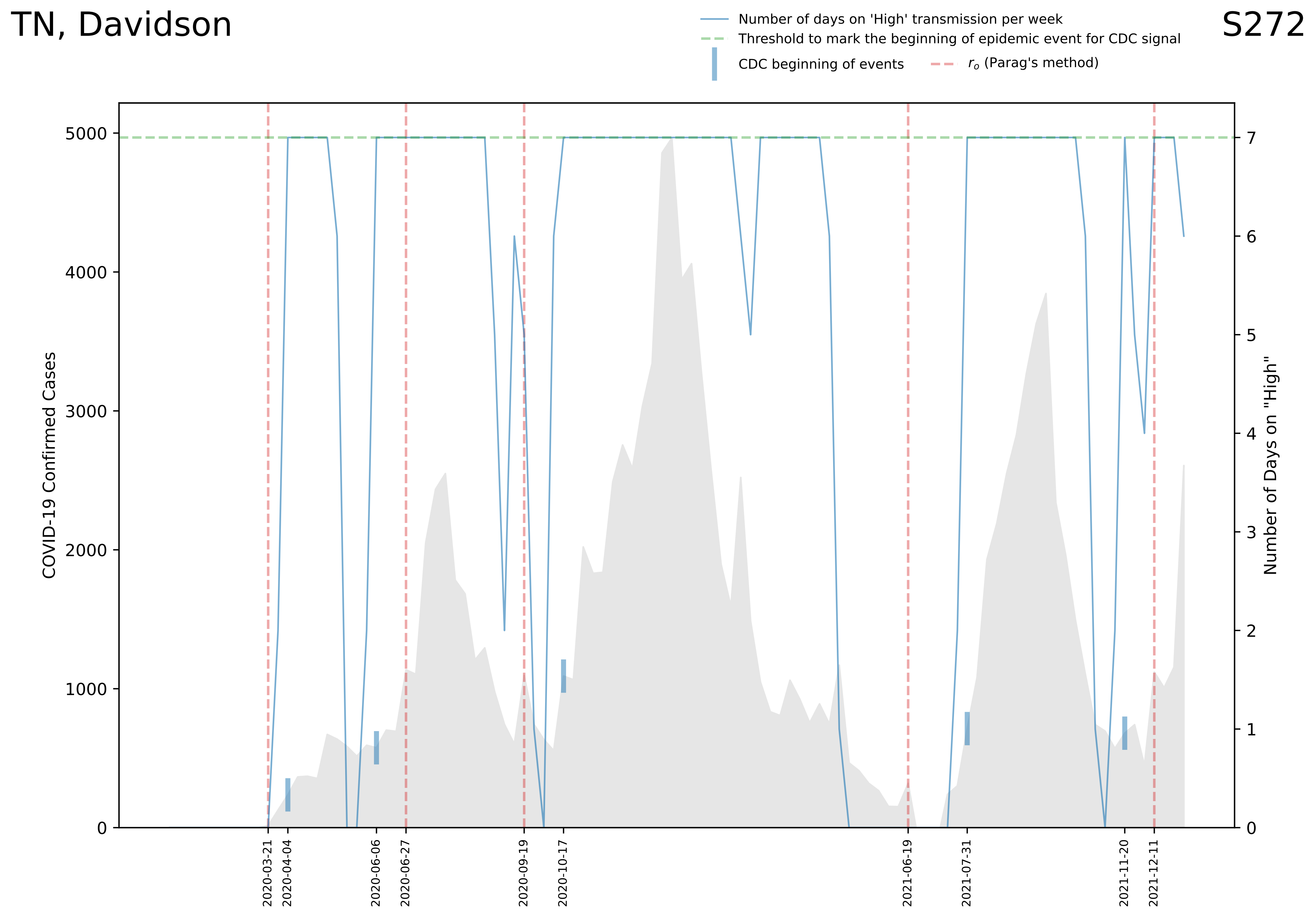

TN, Davidson
S272
Number of days on 'High' transmission per week
Threshold to mark the beginning of epidemic event for CDC signal
CDC beginning of events
r_o (Parag's method)
COVID-19 Confirmed Cases
Number of Days on "High"
0
1000
2000
3000
4000
5000
0
1
2
3
4
5
6
7
2020-03-21
2020-04-04
2020-06-06
2020-06-27
2020-09-19
2020-10-17
2021-06-19
2021-07-31
2021-11-20
2021-12-11

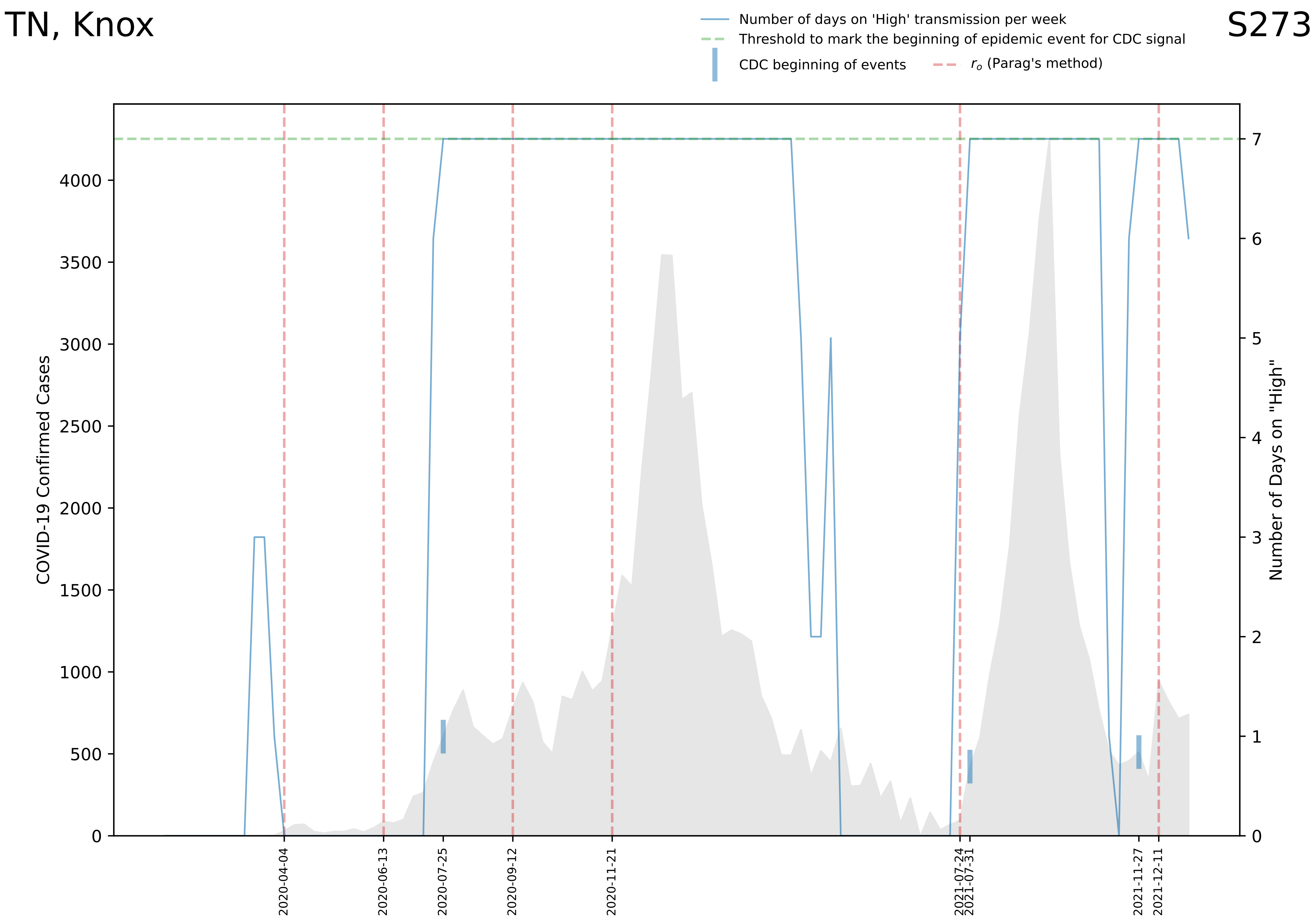

TN, Knox
S273
Number of days on 'High' transmission per week
Threshold to mark the beginning of epidemic event for CDC signal
CDC beginning of events
r_o (Parag's method)
COVID-19 Confirmed Cases
Number of Days on "High"
0
500
1000
1500
2000
2500
3000
3500
4000
0
1
2
3
4
5
6
7
2020-04-04
2020-06-13
2020-07-25
2020-09-12
2020-11-21
2021-07-24
2021-07-31
2021-11-27
2021-12-11

TN, Sevier

S274

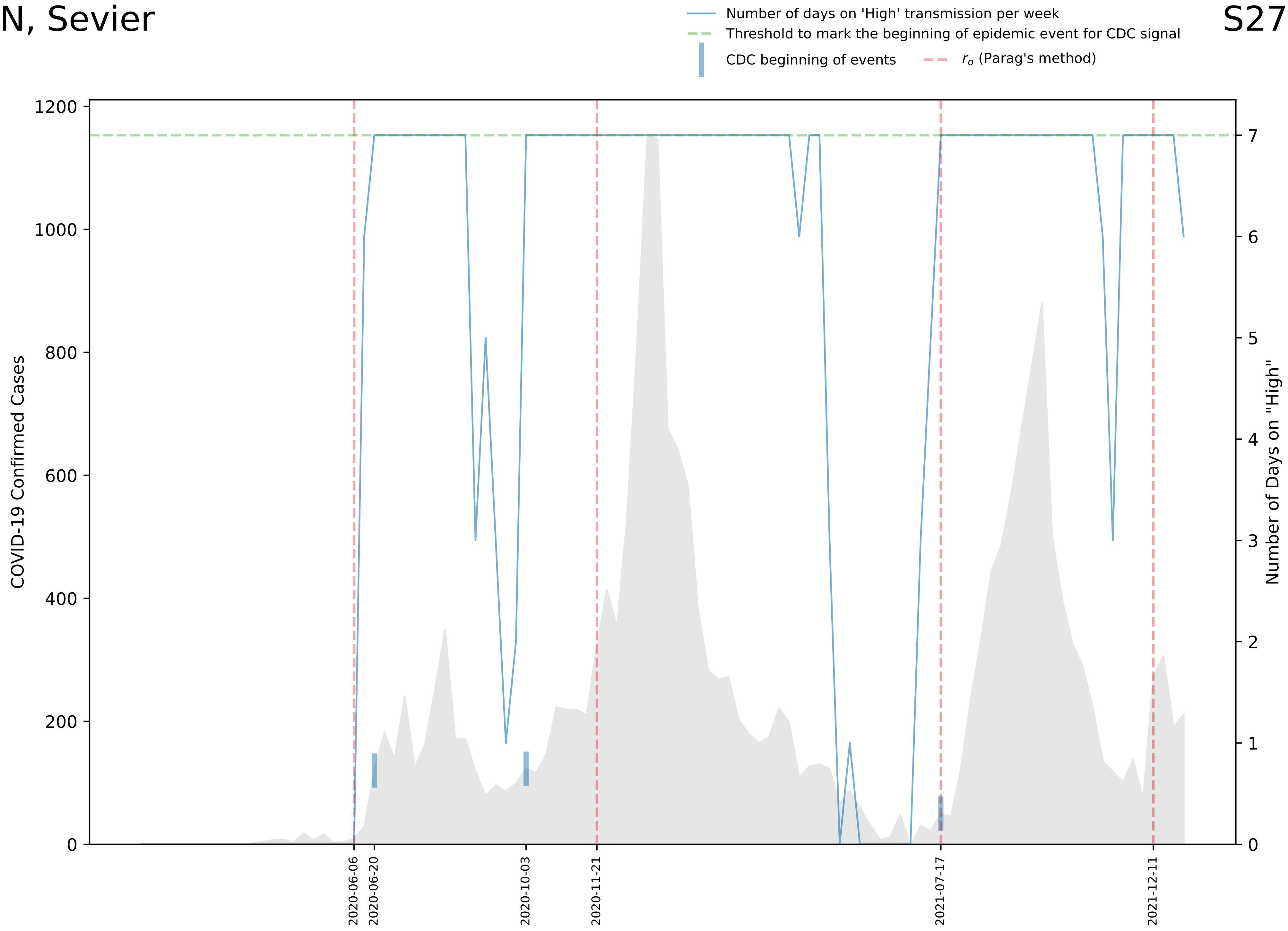

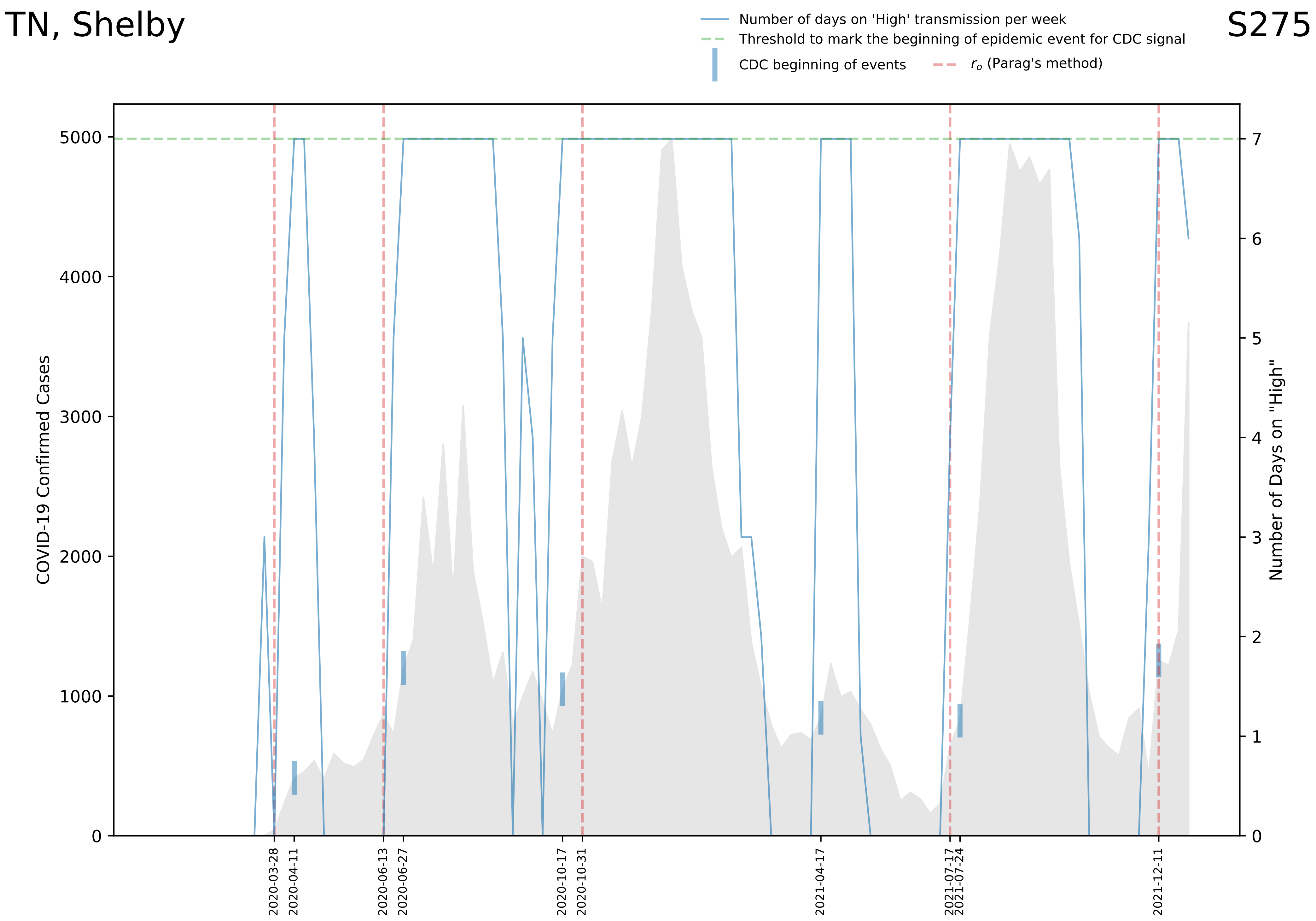

TN, Shelby
S275
Number of days on 'High' transmission per week
Threshold to mark the beginning of epidemic event for CDC signal
CDC beginning of events
r_o (Parag's method)
COVID-19 Confirmed Cases
Number of Days on "High"
0
1000
2000
3000
4000
5000
0
1
2
3
4
5
6
7
2020-03-28
2020-04-11
2020-06-13
2020-06-27
2020-10-17
2020-10-31
2021-04-17
2021-07-17
2021-07-24
2021-12-11

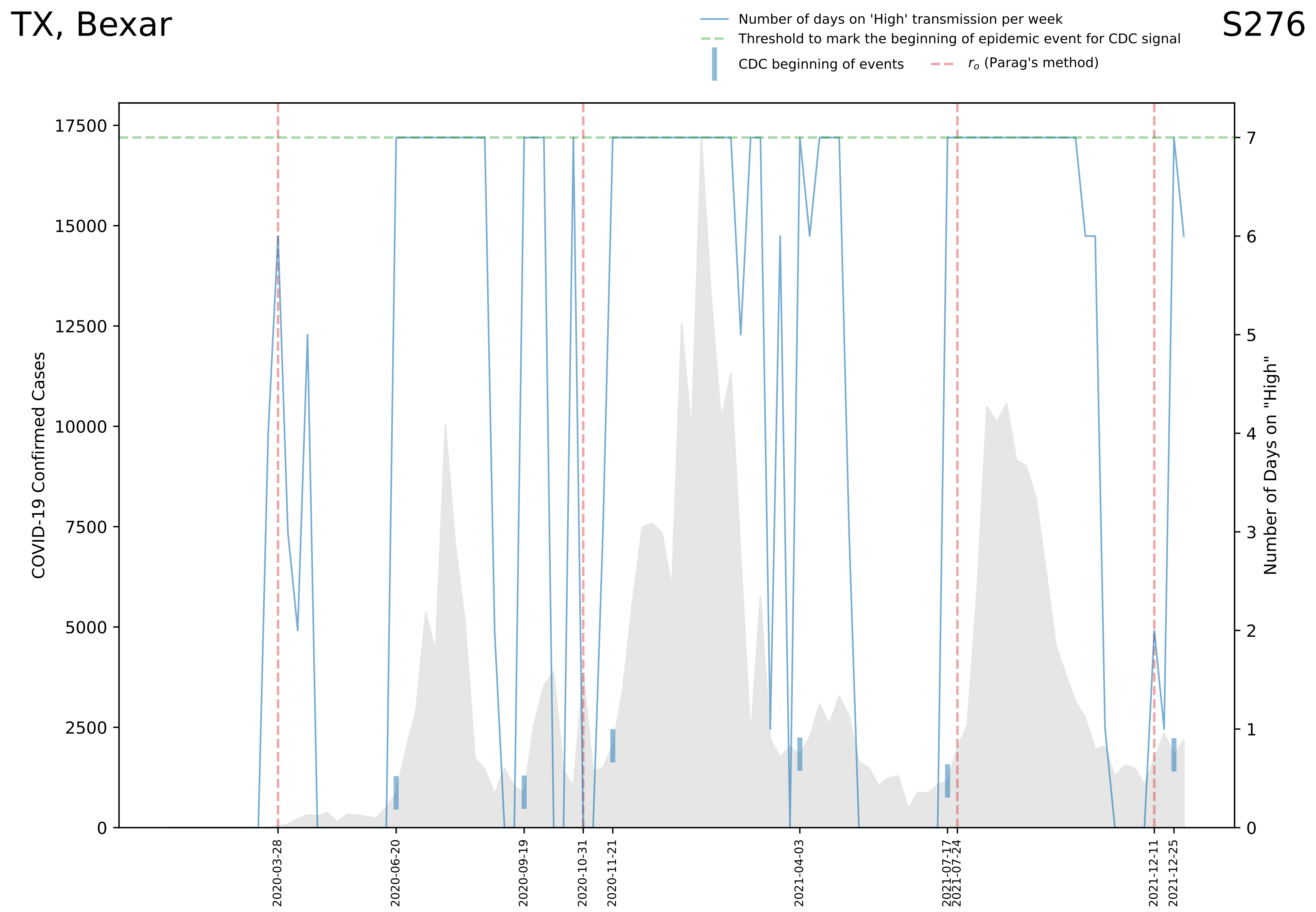

TX, Bexar
S276
Number of days on 'High' transmission per week
Threshold to mark the beginning of epidemic event for CDC signal
CDC beginning of events
r_o (Parag's method)
COVID-19 Confirmed Cases
Number of Days on "High"
17500
15000
12500
10000
7500
5000
2500
0
7
6
5
4
3
2
1
0
2020-03-28
2020-06-20
2020-09-19
2020-10-31
2020-11-21
2021-04-03
2021-07-17
2021-07-24
2021-12-11
2021-12-25

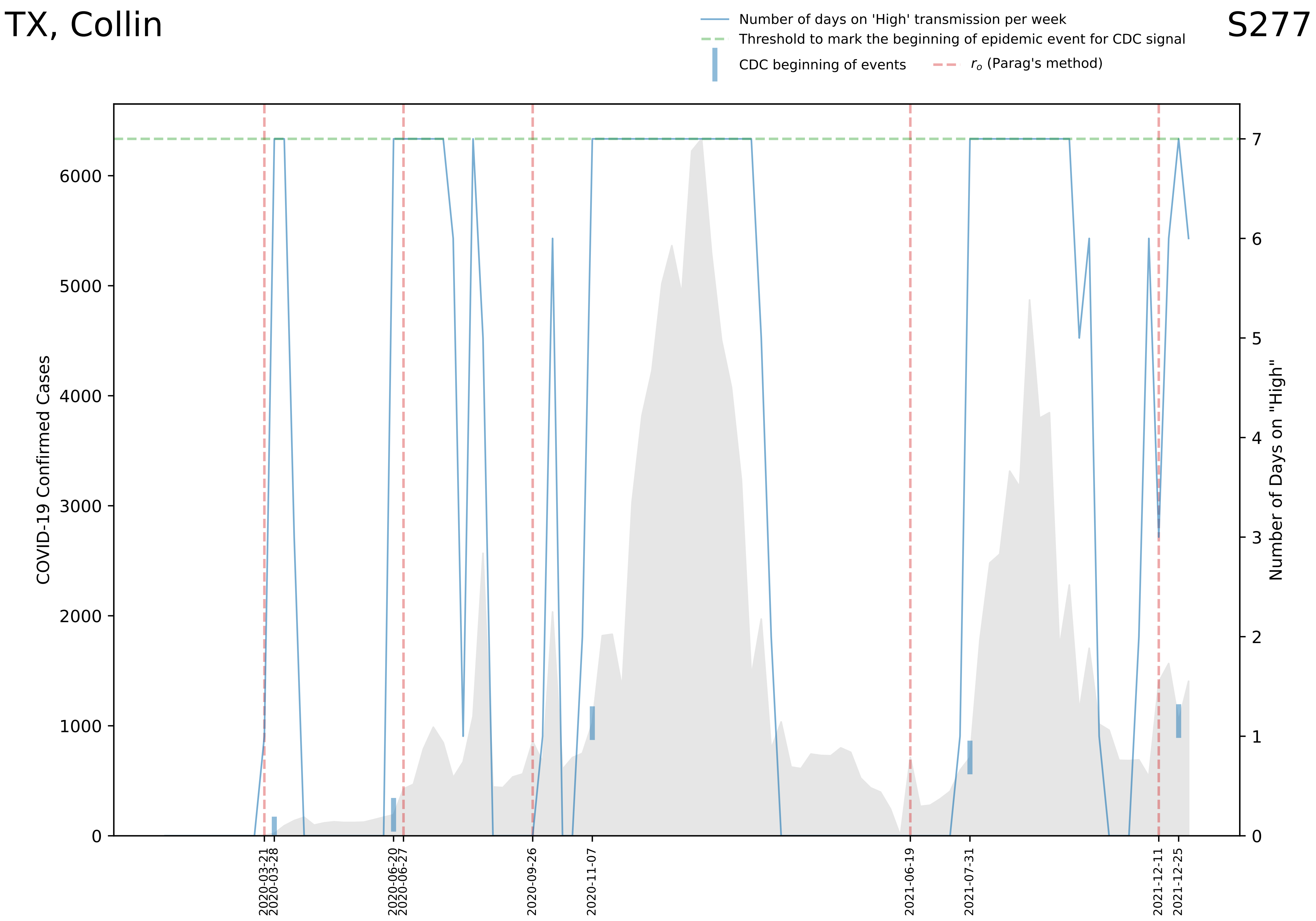
TX, Collin
S277
Number of days on 'High' transmission per week
Threshold to mark the beginning of epidemic event for CDC signal
CDC beginning of events
r_o (Parag's method)
COVID-19 Confirmed Cases
Number of Days on "High"
0
1000
2000
3000
4000
5000
6000
0
1
2
3
4
5
6
7
2020-03-21
2020-03-28
2020-06-20
2020-06-27
2020-09-26
2020-11-07
2021-06-19
2021-07-31
2021-12-11
2021-12-25

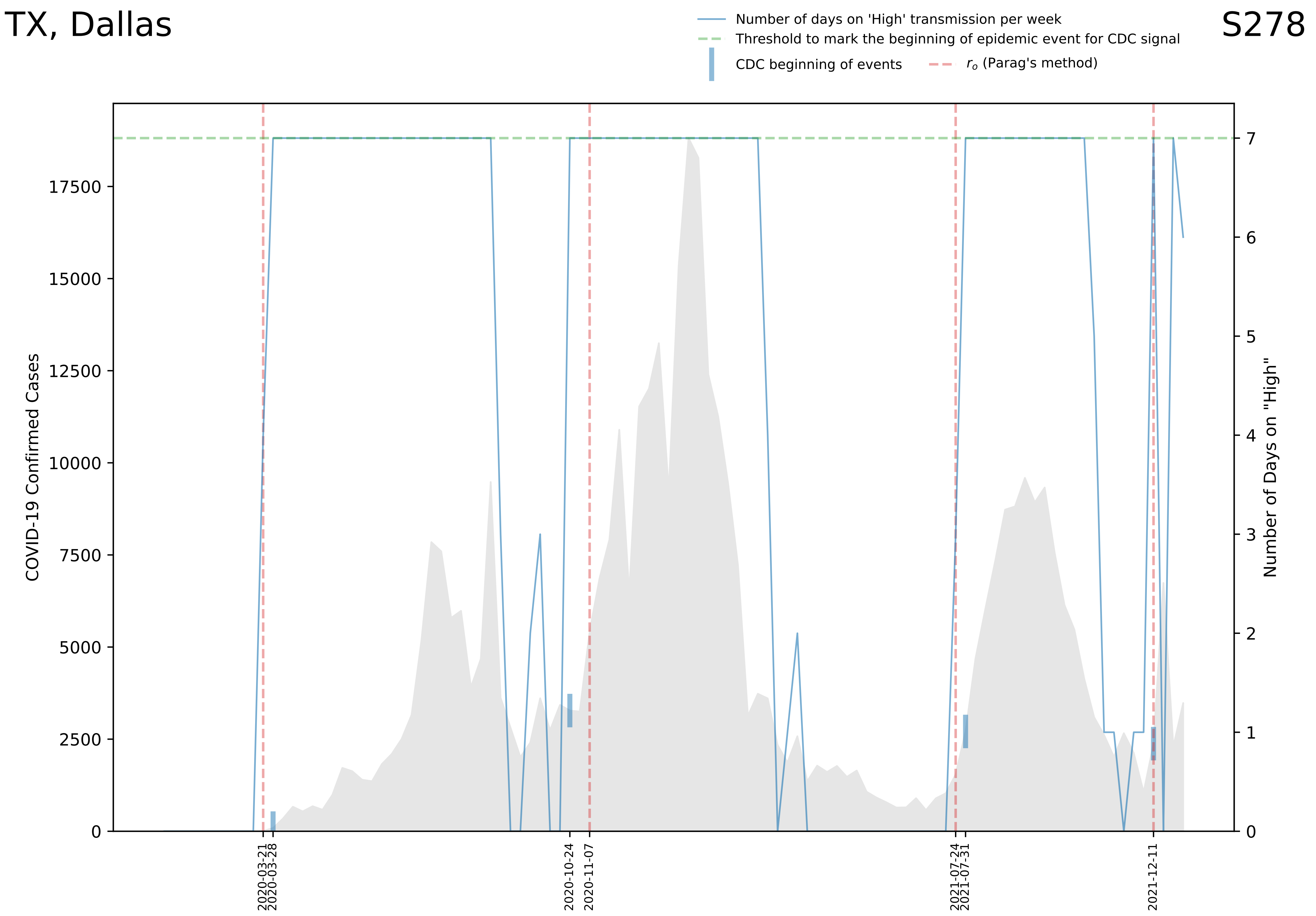

TX, Dallas
S278
Number of days on 'High' transmission per week
Threshold to mark the beginning of epidemic event for CDC signal
CDC beginning of events
r_o (Parag's method)
COVID-19 Confirmed Cases
Number of Days on "High"
0
2500
5000
7500
10000
12500
15000
17500
0
1
2
3
4
5
6
7
2020-03-21
2020-03-28
2020-10-24
2020-11-07
2021-07-24
2021-07-31
2021-12-11

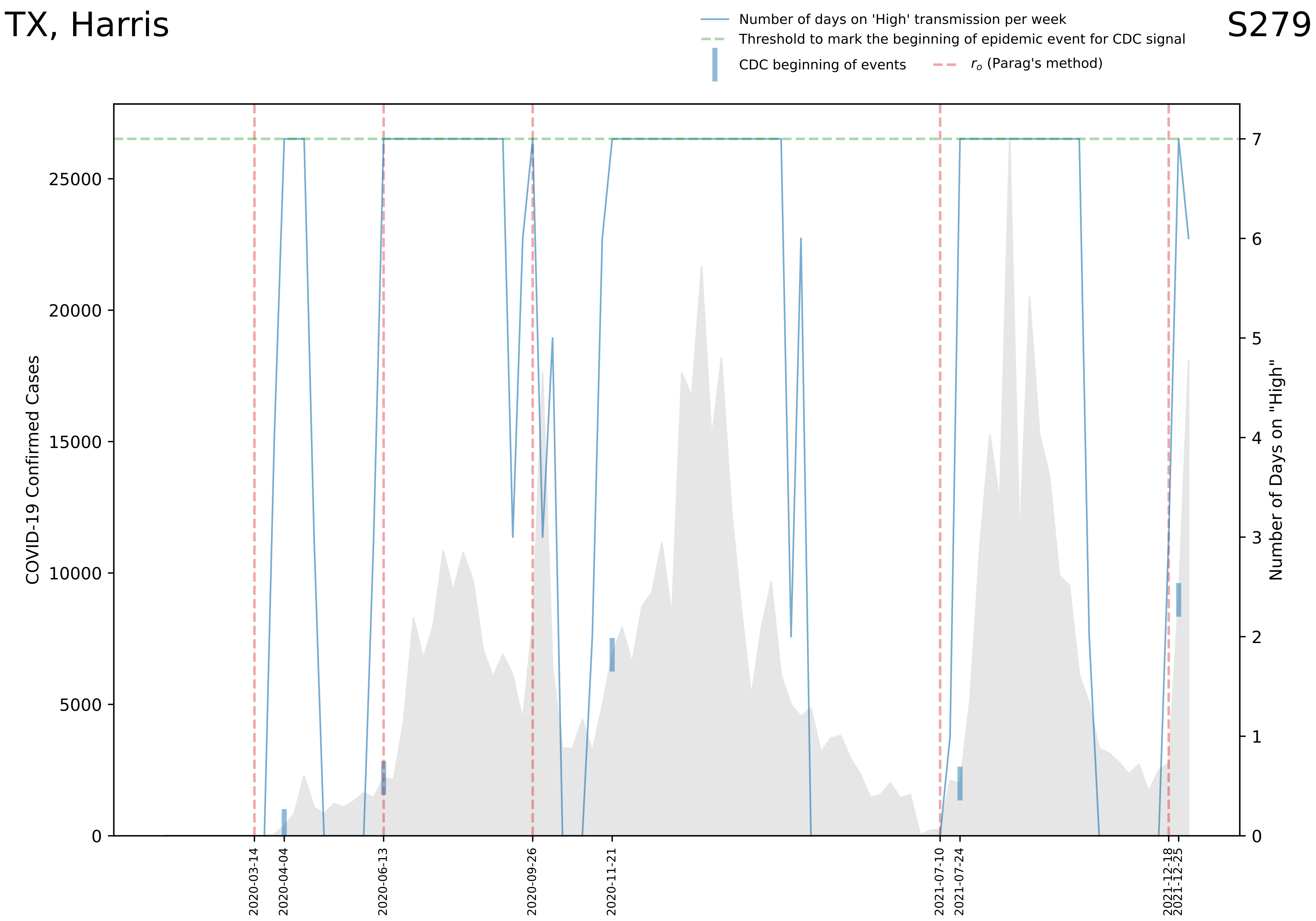

TX, Harris
S279
Number of days on 'High' transmission per week
Threshold to mark the beginning of epidemic event for CDC signal
CDC beginning of events
r_o (Parag's method)
COVID-19 Confirmed Cases
Number of Days on "High"
0
5000
10000
15000
20000
25000
0
1
2
3
4
5
6
7
2020-03-14
2020-04-04
2020-06-13
2020-09-26
2020-11-21
2021-07-10
2021-07-24
2021-12-18
2021-12-25

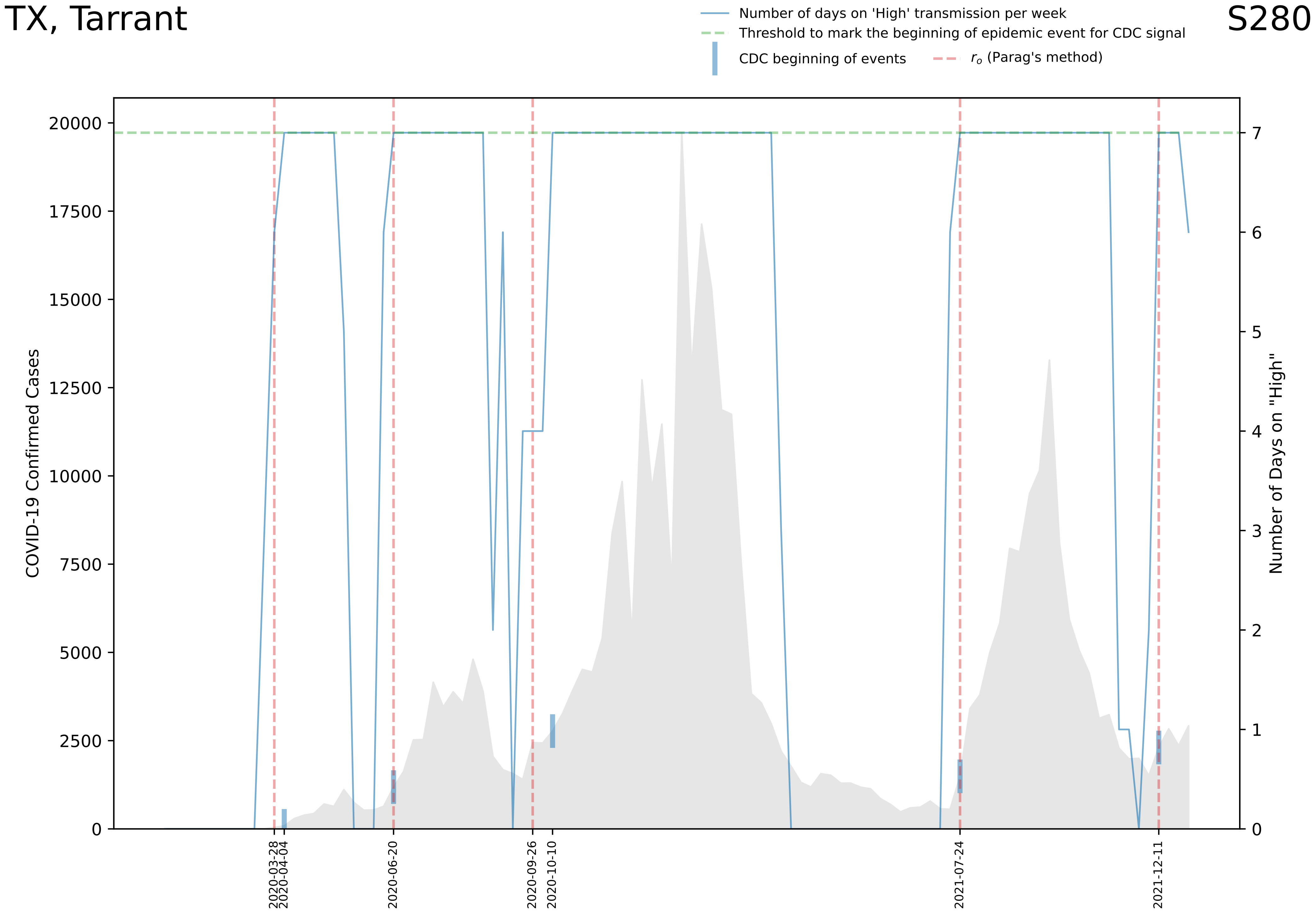

TX, Tarrant
S280
Number of days on 'High' transmission per week
Threshold to mark the beginning of epidemic event for CDC signal
CDC beginning of events
r_o (Parag's method)
COVID-19 Confirmed Cases
Number of Days on "High"
0
2500
5000
7500
10000
12500
15000
17500
20000
1
2
3
4
5
6
7
2020-03-28
2020-04-04
2020-06-20
2020-09-26
2020-10-10
2021-07-24
2021-12-11

TX, Travis

S281

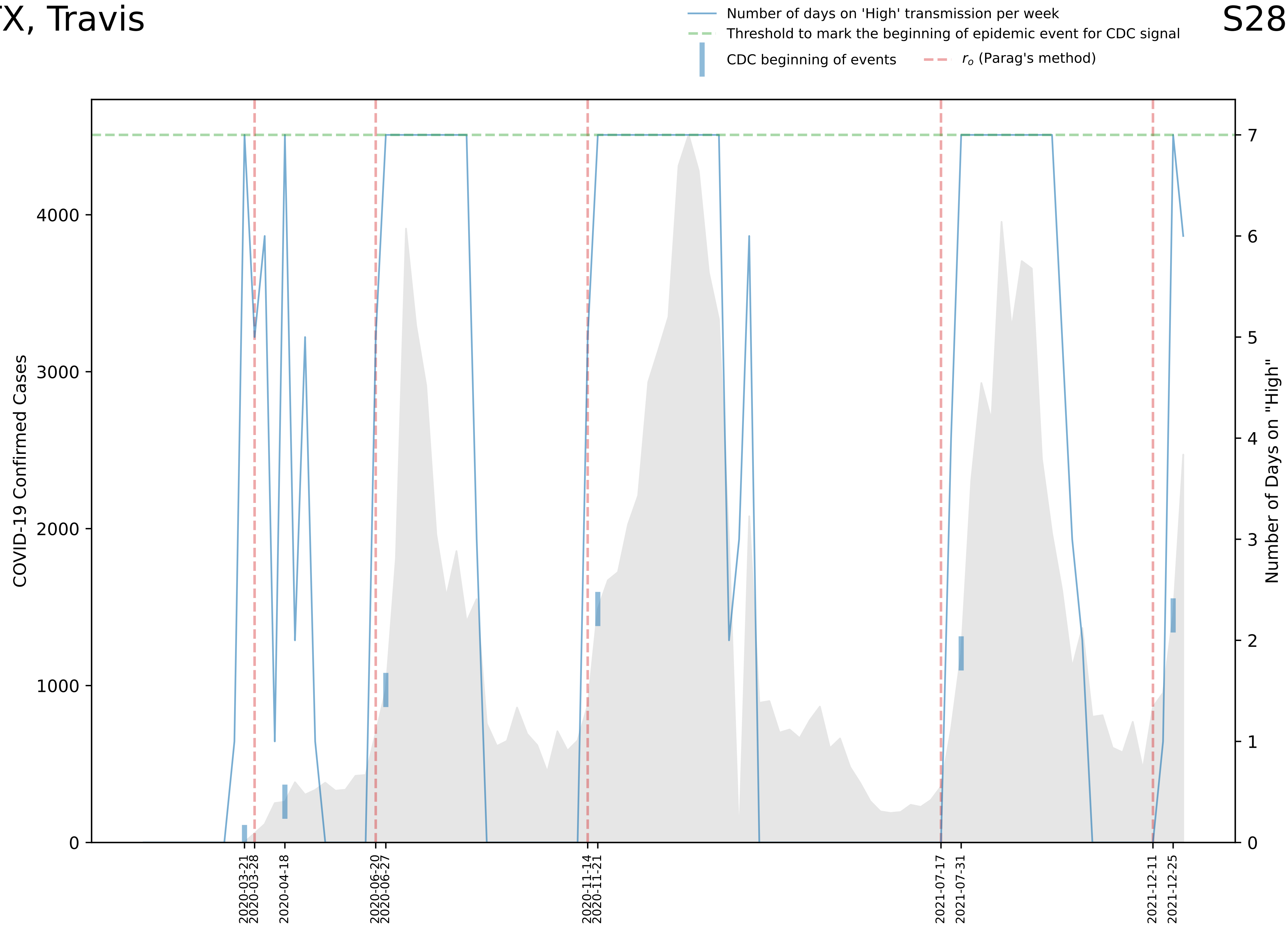

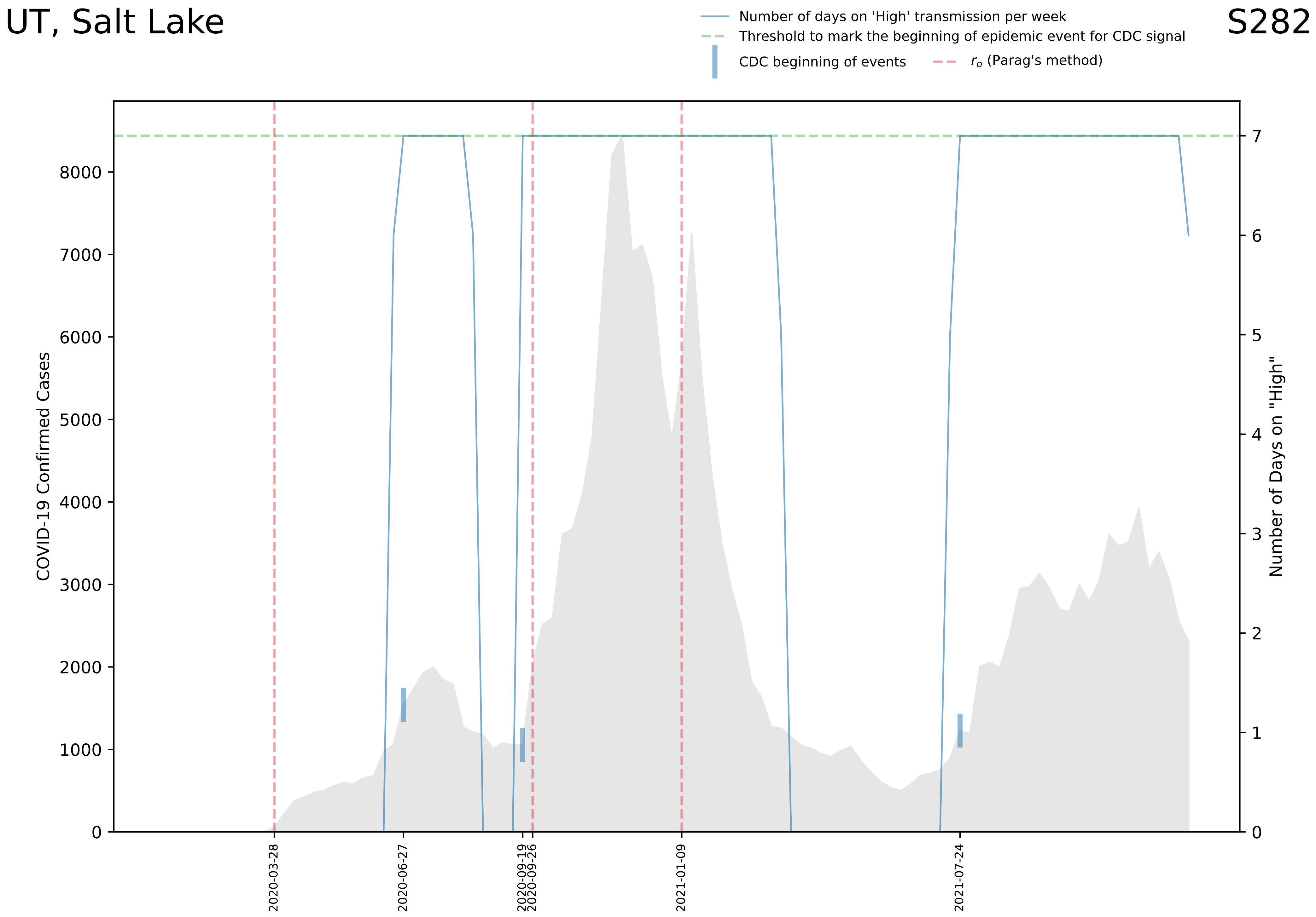

UT, Salt Lake
S282
Number of days on 'High' transmission per week
Threshold to mark the beginning of epidemic event for CDC signal
CDC beginning of events
r_o (Parag's method)
COVID-19 Confirmed Cases
Number of Days on "High"
0
1000
2000
3000
4000
5000
6000
7000
8000
0
1
2
3
4
5
6
7
2020-03-28
2020-06-27
2020-09-19
2020-09-26
2021-01-09
2021-07-24

VA, Albemarle

S283

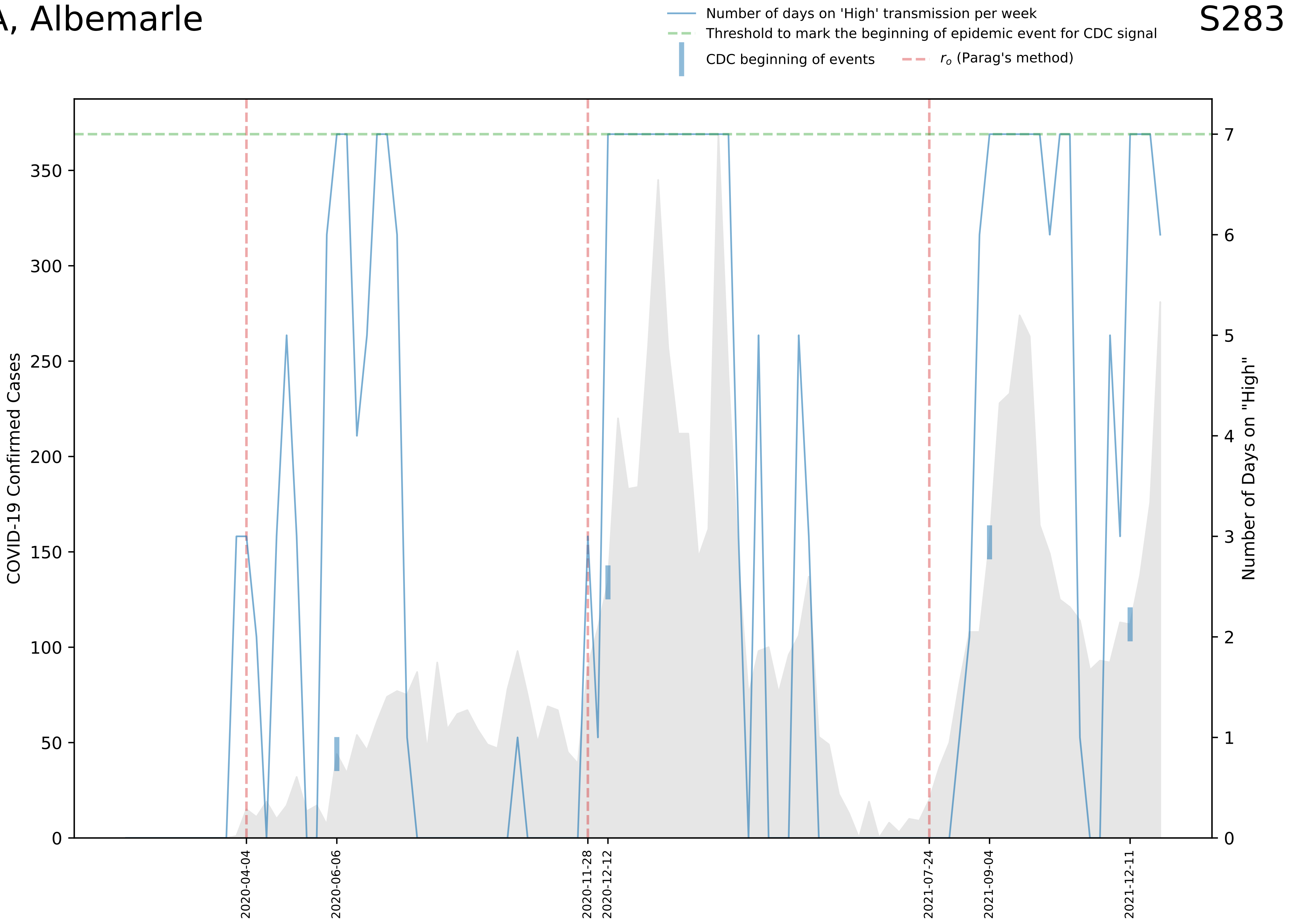

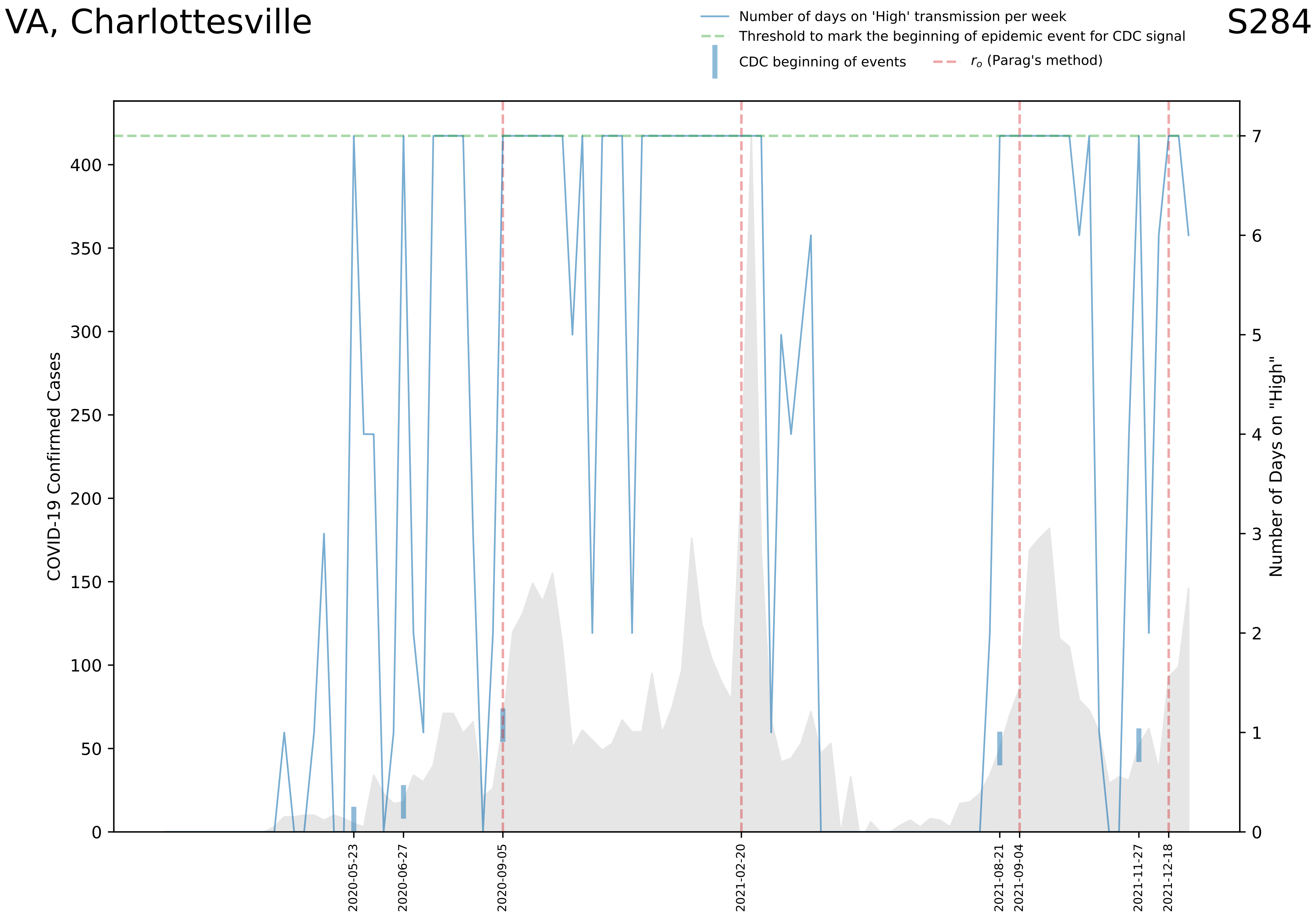

VA, Charlottesville
S284
Number of days on 'High' transmission per week
Threshold to mark the beginning of epidemic event for CDC signal
CDC beginning of events
r_o (Parag's method)
COVID-19 Confirmed Cases
Number of Days on "High"
0
50
100
150
200
250
300
350
400
0
1
2
3
4
5
6
7
2020-05-23
2020-06-27
2020-09-05
2021-02-20
2021-08-21
2021-09-04
2021-11-27
2021-12-18

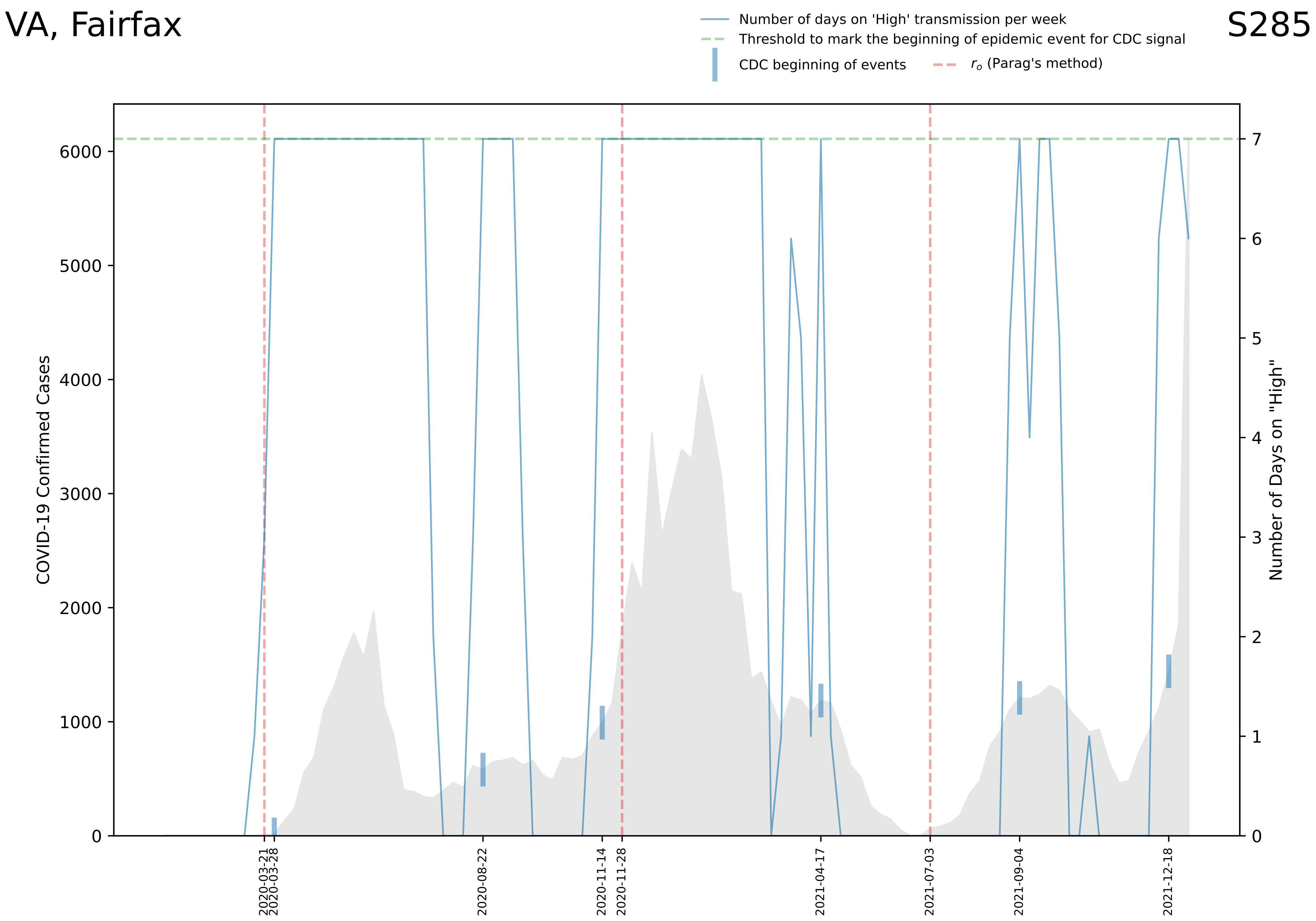
VA, Fairfax
S285
Number of days on 'High' transmission per week
Threshold to mark the beginning of epidemic event for CDC signal
CDC beginning of events
r_o (Parag's method)
COVID-19 Confirmed Cases
Number of Days on "High"
0
1000
2000
3000
4000
5000
6000
0
1
2
3
4
5
6
7
2020-03-21
2020-03-28
2020-08-22
2020-11-14
2020-11-28
2021-04-17
2021-07-03
2021-09-04
2021-12-18

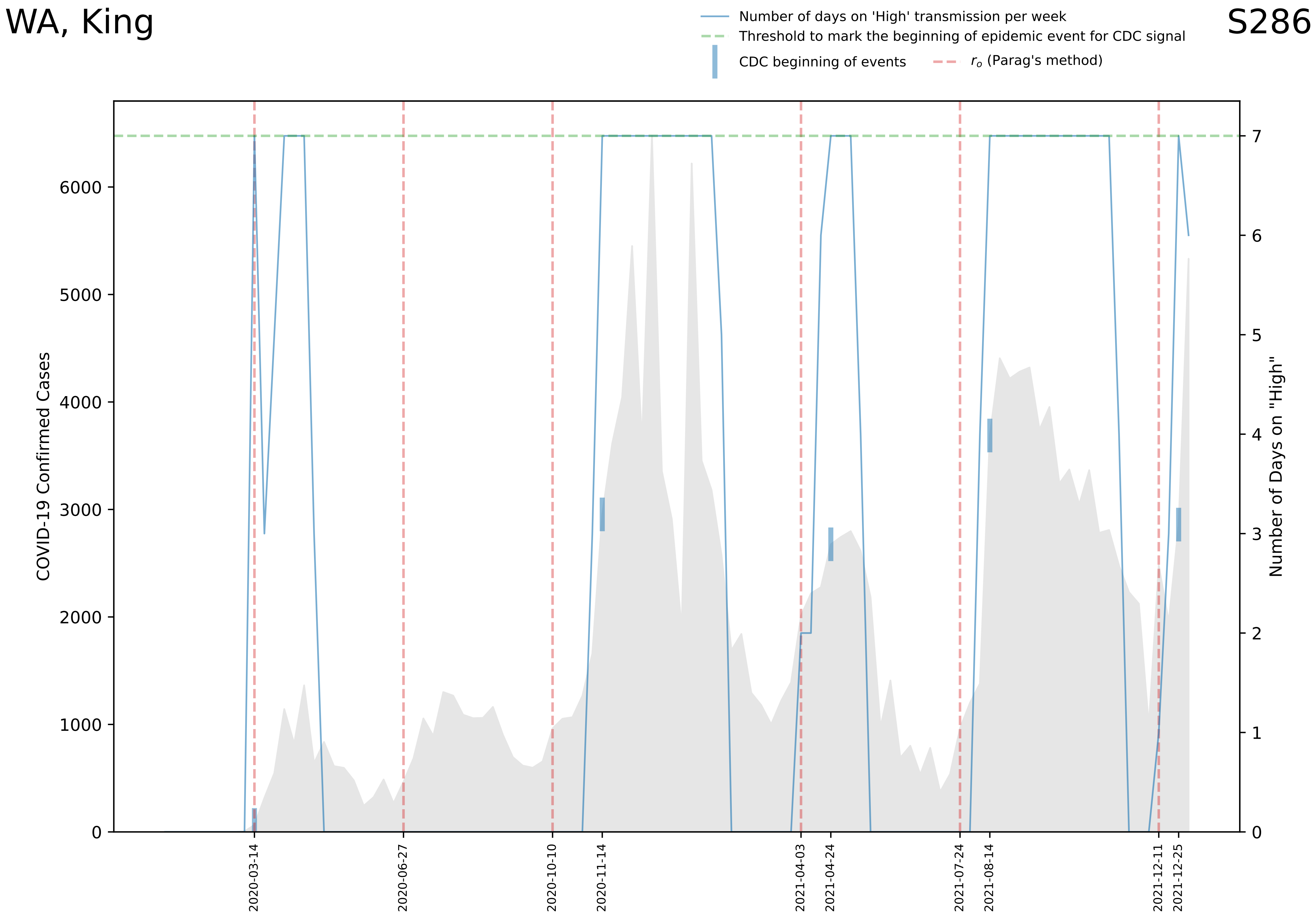

WA, King
S286
Number of days on 'High' transmission per week
Threshold to mark the beginning of epidemic event for CDC signal
CDC beginning of events
r_o (Parag's method)
COVID-19 Confirmed Cases
Number of Days on "High"
0
1000
2000
3000
4000
5000
6000
0
1
2
3
4
5
6
7
2020-03-14
2020-06-27
2020-10-10
2020-11-14
2021-04-03
2021-04-24
2021-07-24
2021-08-14
2021-12-11
2021-12-25

Model	Naive			Single Source (”How long does covid last?”)			Single Source (”side effects of vaccine”)			Multiple Sources		
Time	Before 2021-12-01	After 2021-12-01	Total	Before 2021-12-01	After 2021-12-01	Total	Before 2021-12-01	After 2021-12-01	Total	Before 2021-12-01	After 2021-12-01	Total
Total Events	305 (100%)	62 (100%)	367 (100%)	305 (100%)	62 (100%)	367 (100%)	305 (100%)	62 (100%)	367 (100%)	305 (100%)	62 (100%)	367 (100%)
Early Warning	279 (91%)	58 (94%)	337 (92%)	202 (66%)	35 (56%)	237 (65%)	184 (60%)	43 (69%)	227 (62%)	200 (66%)	54 (87%)	254 (69%)
Sync Warning	19 (6%)	4 (6%)	23 (6%)	23 (8%)	2 (3%)	25 (7%)	16 (5%)	3 (5%)	19 (5%)	30 (10%)	0 (0%)	30 (8%)
Late Warning	1 (0%)	0 (0%)	1 (0%)	26 (9%)	4 (6%)	30 (8%)	14 (5%)	1 (2%)	15 (4%)	26 (9%)	0 (0%)	26 (7%)
Soft Warning	0 (0%)	0 (0%)	0 (0%)	0 (0%)	0 (0%)	0 (0%)	0 (0%)	0 (0%)	0 (0%)	39 (13%)	8 (13%)	47 (13%)
Missed Outbreaks	6 (2%)	0 (0%)	6 (2%)	54 (18%)	21 (34%)	75 (20%)	91 (30%)	15 (24%)	106 (29%)	10 (3%)	0 (0%)	10 (3%)
Warning, increase observed	224	28	252	122	17	139	148	23	171	35	1	36
False Alarm	502	110	612	280	47	327	374	105	479	95	15	110

Table S1: **A Summary of the results at the county-level.** Here *EWS* stands for Early Warning System.

Model	Naive			Single Source (”How long does covid last?”)			Single Source (”chest pain”)			Multiple Sources		
Time	Before 2021-12-01	After 2021-12-01	Total	Before 2021-12-01	After 2021-12-01	Total	Before 2021-12-01	After 2021-12-01	Total	Before 2021-12-01	After 2021-12-01	Total
Total Events	183 (100%)	19 (100%)	202 (100%)	183 (100%)	19 (100%)	202 (100%)	183 (100%)	19 (100%)	202 (100%)	183 (100%)	19 (100%)	202 (100%)
Early Warning	160 (87%)	18 (95%)	178 (88%)	115 (63%)	13 (68%)	128 (63%)	108 (59%)	12 (63%)	120 (59%)	101 (55%)	18 (95%)	119 (59%)
Sync Warning	22 (12%)	1 (5%)	23 (11%)	11 (6%)	2 (11%)	13 (6%)	12 (7%)	0 (0%)	12 (6%)	16 (9%)	0 (0%)	16 (8%)
Late Warning	1 (1%)	0 (0%)	1 (0%)	17 (9%)	1 (5%)	18 (9%)	12 (7%)	0 (0%)	12 (6%)	20 (11%)	0 (0%)	20 (10%)
Soft Warning	0 (0%)	0 (0%)	0 (0%)	0 (0%)	0 (0%)	0 (0%)	0 (0%)	0 (0%)	0 (0%)	36 (20%)	1 (5%)	37 (18%)
Missed Outbreaks	0 (0%)	0 (0%)	0 (0%)	40 (22%)	3 (16%)	43 (21%)	51 (28%)	7 (37%)	58 (29%)	10 (5%)	0 (0%)	10 (5%)
Warning, increase observed	74	8	82	57	6	63	35	6	41	18	0	18
False Alarm	171	39	210	104	15	119	112	9	121	23	1	24

Table S2: **A Summary of the results at the state-level.** Here *EWS* stands for Early Warning System.

N = 367	Early Warning	Sync Warning	Late Warning	Missed Outbreaks	Warning, activity increases	False Alarm
(GT) how long does covid last	237 (65%)	25 (7%)	30 (8%)	74 (20%)	139	327
(GT) side effects of vaccine	227 (62%)	19 (5%)	15 (4%)	105 (29%)	171	479
(GT) chest pain	201 (55%)	19 (5%)	38 (10%)	108 (29%)	128	301
(GT) covid 19Who	169 (46%)	25 (7%)	20 (5%)	152 (41%)	87	269
COVID-19 confirmed deaths	147 (40%)	16 (4%)	31 (8%)	172 (47%)	93	249
(GT) covidSymptoms	145 (40%)	30 (8%)	50 (14%)	141 (38%)	58	122
(GT) after covid vaccine	141 (38%)	20 (5%)	29 (8%)	176 (48%)	182	463
COVID-19 confirmed cases	135 (37%)	59 (16%)	58 (16%)	114 (31%)	100	204
(GT) effects of covid vaccine	107 (29%)	14 (4%)	20 (5%)	225 (61%)	110	374
(GT) fever	85 (23%)	24 (7%)	21 (6%)	236 (64%)	40	107
(GT) covid 19	79 (22%)	22 (6%)	53 (14%)	212 (58%)	52	127
(gt) covid	51 (14%)	19 (5%)	65 (18%)	231 (63%)	10	54
Twitter	41 (11%)	14 (4%)	56 (15%)	255 (69%)	20	40
Up2Date	37 (10%)	7 (2%)	24 (7%)	298 (81%)	10	32

Table S3: **County-Level results for the Single Source Method**

N=202	Early Warning	Sync Warning	Late Warning	Missed Outbreaks	Warning, activity increases	False Alarm
(GT) how long does covid last	128 (63%)	13 (6%)	18 (9%)	42 (21%)	63	119
(GT) chest pain	120 (59%)	12 (6%)	12 (6%)	57 (28%)	41	121
(GT) covid 19Who	112 (55%)	11 (5%)	19 (9%)	59 (29%)	51	104
(GT) side effects of vaccine	96 (48%)	12 (6%)	18 (9%)	75 (37%)	56	165
(GT) covidSymptoms	88 (44%)	8 (4%)	15 (7%)	90 (45%)	28	45
COVID-19 confirmed cases	78 (39%)	22 (11%)	33 (16%)	68 (34%)	29	97
(GT) after covid vaccine	68 (34%)	8 (4%)	8 (4%)	117 (58%)	67	149
COVID-19 confirmed deaths	65 (32%)	13 (6%)	13 (6%)	110 (54%)	28	72
(GT) effects of covid vaccine	63 (31%)	6 (3%)	7 (3%)	125 (62%)	30	90
(GT) covid 19	46 (23%)	9 (4%)	30 (15%)	116 (57%)	21	54
(GT) fever	45 (22%)	5 (2%)	15 (7%)	136 (67%)	24	42
Twitter	27 (13%)	8 (4%)	28 (14%)	138 (68%)	10	11
(gt) covid	26 (13%)	5 (2%)	21 (10%)	149 (74%)	10	14
Up2Date	18 (9%)	5 (2%)	14 (7%)	164 (81%)	8	9

Table S4: **State-Level results for the Single Source Method**

Data Source(s) Used	Early Warning	Sync Warning	Late Warning	Soft Warning	Missed Outbreaks	Warning increase observed	False Alarm	Non-deployed
G, T, A	120 (59%)	13 (6%)	24 (12%)	37 (18%)	8 (4%)	16	32	0 (0%)
L, G, T	119 (59%)	16 (8%)	20 (10%)	37 (18%)	10 (5%)	18	24	0 (0%)
L, G	119 (59%)	16 (8%)	19 (9%)	39 (19%)	9 (4%)	19	24	0 (0%)
G, T	118 (58%)	12 (6%)	25 (12%)	39 (19%)	8 (4%)	16	26	0 (0%)
G, T, A, U	118 (58%)	13 (6%)	24 (12%)	39 (19%)	8 (4%)	16	32	0 (0%)
G, A	117 (58%)	15 (7%)	24 (12%)	38 (19%)	8 (4%)	17	31	0 (0%)
G,	117 (58%)	13 (6%)	24 (12%)	40 (20%)	8 (4%)	17	27	0 (0%)
G, T, U	116 (57%)	12 (6%)	27 (13%)	39 (19%)	8 (4%)	16	28	0 (0%)
G, A, U	116 (57%)	15 (7%)	23 (11%)	40 (20%)	8 (4%)	17	32	0 (0%)
L, G, U	116 (57%)	16 (8%)	20 (10%)	41 (20%)	9 (4%)	20	24	0 (0%)
L, G, T, U	116 (57%)	16 (8%)	21 (10%)	39 (19%)	10 (5%)	19	25	0 (0%)
L, G, T, A	115 (57%)	18 (9%)	20 (10%)	39 (19%)	10 (5%)	17	31	0 (0%)
G, U	115 (57%)	13 (6%)	26 (13%)	40 (20%)	8 (4%)	17	27	0 (0%)
L, G, A	114 (56%)	18 (9%)	20 (10%)	41 (20%)	9 (4%)	18	29	0 (0%)
L, G, T, A, U	114 (56%)	17 (8%)	19 (9%)	42 (21%)	10 (5%)	17	30	0 (0%)
L, G, A, U	113 (56%)	17 (8%)	19 (9%)	44 (22%)	9 (4%)	18	29	0 (0%)
G, N, T, A	110 (54%)	22 (11%)	24 (12%)	35 (17%)	11 (5%)	15	29	0 (0%)
G, N, T	109 (54%)	21 (10%)	25 (12%)	35 (17%)	12 (6%)	13	24	0 (0%)
G, N, A	109 (54%)	23 (11%)	23 (11%)	36 (18%)	11 (5%)	15	29	0 (0%)
G, N	108 (53%)	22 (11%)	24 (12%)	36 (18%)	12 (6%)	14	23	0 (0%)
G, N, T, A, U	108 (53%)	22 (11%)	24 (12%)	37 (18%)	11 (5%)	16	28	0 (0%)
G, N, T, U	107 (53%)	21 (10%)	25 (12%)	37 (18%)	12 (6%)	14	23	0 (0%)
L, G, N, T, A	107 (53%)	26 (13%)	21 (10%)	36 (18%)	12 (6%)	16	28	0 (0%)
G, N, A, U	107 (53%)	23 (11%)	23 (11%)	38 (19%)	11 (5%)	16	28	0 (0%)
L, G, N, A	106 (52%)	27 (13%)	20 (10%)	37 (18%)	12 (6%)	16	28	0 (0%)
G, N, U	106 (52%)	22 (11%)	24 (12%)	38 (19%)	12 (6%)	15	22	0 (0%)
L, G, N, T	104 (51%)	23 (11%)	24 (12%)	38 (19%)	13 (6%)	13	23	0 (0%)
L, G, N, T, A, U	104 (51%)	26 (13%)	21 (10%)	39 (19%)	12 (6%)	17	27	0 (0%)
L, G, N, A, U	103 (51%)	27 (13%)	20 (10%)	40 (20%)	12 (6%)	17	27	0 (0%)
L, G, N	103 (51%)	24 (12%)	23 (11%)	39 (19%)	13 (6%)	14	22	0 (0%)
L, G, N, T, U	101 (50%)	23 (11%)	24 (12%)	41 (20%)	13 (6%)	14	22	0 (0%)
L, G, N, U	100 (50%)	24 (12%)	23 (11%)	42 (21%)	13 (6%)	15	21	0 (0%)
L, T, A, U	86 (43%)	27 (13%)	18 (9%)	44 (22%)	21 (10%)	17	60	6 (3%)
L, A	85 (42%)	29 (14%)	8 (4%)	24 (12%)	38 (19%)	18	58	18 (9%)
L, T, A	84 (42%)	28 (14%)	17 (8%)	43 (21%)	22 (11%)	17	61	8 (4%)
L, A, U	81 (40%)	27 (13%)	8 (4%)	38 (19%)	36 (18%)	18	58	12 (6%)
T, A	80 (40%)	17 (8%)	15 (7%)	45 (22%)	32 (16%)	17	58	13 (6%)
T, A, U	80 (40%)	17 (8%)	16 (8%)	48 (24%)	30 (15%)	19	55	11 (5%)
L, N, A, U	79 (39%)	22 (11%)	21 (10%)	55 (27%)	24 (12%)	10	26	1 (0%)
N, A, U	79 (39%)	21 (10%)	17 (8%)	55 (27%)	29 (14%)	10	29	1 (0%)
N, T, A, U	79 (39%)	20 (10%)	16 (8%)	58 (29%)	28 (14%)	9	23	1 (0%)
L, T, U	79 (39%)	39 (19%)	27 (13%)	3 (1%)	26 (13%)	10	20	28 (14%)
N, T, A	77 (38%)	20 (10%)	15 (7%)	59 (29%)	28 (14%)	11	24	3 (1%)
N, A	77 (38%)	19 (9%)	16 (8%)	56 (28%)	30 (15%)	10	27	4 (2%)
L, N, A	76 (38%)	23 (11%)	21 (10%)	55 (27%)	23 (11%)	9	25	4 (2%)
L, N, T, A, U	76 (38%)	22 (11%)	21 (10%)	60 (30%)	22 (11%)	8	22	1 (0%)
A,	75 (37%)	14 (7%)	5 (2%)	21 (10%)	57 (28%)	16	56	30 (15%)
A, U	73 (36%)	14 (7%)	7 (3%)	32 (16%)	53 (26%)	18	58	23 (11%)
L, N, T, A	73 (36%)	24 (12%)	21 (10%)	58 (29%)	23 (11%)	9	23	3 (1%)
L, T	72 (36%)	40 (20%)	28 (14%)	0 (0%)	23 (11%)	8	22	39 (19%)
T, U	67 (33%)	18 (9%)	37 (18%)	0 (0%)	36 (18%)	9	21	44 (22%)
N, T, U	65 (32%)	23 (11%)	19 (9%)	50 (25%)	38 (19%)	6	16	7 (3%)
L, N, T, U	63 (31%)	24 (12%)	24 (12%)	56 (28%)	28 (14%)	5	15	7 (3%)
L, N, U	62 (31%)	26 (13%)	25 (12%)	52 (26%)	30 (15%)	7	15	7 (3%)
N, U	62 (31%)	25 (12%)	21 (10%)	47 (23%)	40 (20%)	7	17	7 (3%)
N, T	60 (30%)	27 (13%)	18 (9%)	48 (24%)	39 (19%)	6	15	10 (5%)
N,	59 (29%)	23 (11%)	19 (9%)	47 (23%)	43 (21%)	6	15	11 (5%)
L, N	58 (29%)	25 (12%)	24 (12%)	52 (26%)	32 (16%)	6	13	11 (5%)
T,	56 (28%)	17 (8%)	39 (19%)	0 (0%)	32 (16%)	7	19	58 (29%)
L, N, T	56 (28%)	29 (14%)	24 (12%)	53 (26%)	30 (15%)	5	13	10 (5%)
L, U	37 (18%)	48 (24%)	13 (6%)	0 (0%)	43 (21%)	8	9	61 (30%)
L,	23 (11%)	43 (21%)	10 (5%)	0 (0%)	21 (10%)	4	6	105 (52%)
U,	17 (8%)	6 (3%)	6 (3%)	0 (0%)	49 (24%)	5	3	124 (61%)

Table S5: **State-level performance (Jan 1st, 2020 – Jan 1st, 2022) of the Multiple Source method for different data sources used for the training step.** The source labeling is as follows: G = Google trends, T = Twitter, U = UpToDate, L = local epidemiological data, A = Human mobility from Apple, and N = Neighbor's epidemiological data. An additional column, 'Non-deployed,' indicates the number of outbreak events where our Multiple Source method did not find a reasonable set of proxies to fit a model. Thus, an early warning system was not deployed to forecast the out-of-sample period.

Data Source(s) Used	Early Warning	Sync Warning	Late Warning	Soft Warning	Missed Outbreaks	Warning increase observed	False Alarm	Non-deployed
G, T, U	258 (70%)	21 (6%)	27 (7%)	52 (14%)	9 (2%)	36	121	0 (0%)
G, U	257 (70%)	21 (6%)	27 (7%)	53 (14%)	9 (2%)	36	122	0 (0%)
G, T	257 (70%)	20 (5%)	27 (7%)	54 (15%)	9 (2%)	36	121	0 (0%)
G,	256 (70%)	20 (5%)	28 (8%)	54 (15%)	9 (2%)	36	121	0 (0%)
G, T, A, U	255 (69%)	23 (6%)	28 (8%)	55 (15%)	6 (2%)	36	121	0 (0%)
L, G, T, U	254 (69%)	30 (8%)	26 (7%)	47 (13%)	10 (3%)	36	110	0 (0%)
G, A, U	254 (69%)	23 (6%)	28 (8%)	56 (15%)	6 (2%)	36	122	0 (0%)
L, G, T, A, U	253 (69%)	30 (8%)	27 (7%)	49 (13%)	8 (2%)	36	113	0 (0%)
L, G, T	253 (69%)	29 (8%)	26 (7%)	49 (13%)	10 (3%)	36	111	0 (0%)
G, T, A	253 (69%)	23 (6%)	28 (8%)	57 (16%)	6 (2%)	36	120	0 (0%)
L, G, U	252 (69%)	30 (8%)	26 (7%)	49 (13%)	10 (3%)	36	111	0 (0%)
G, A	252 (69%)	23 (6%)	29 (8%)	57 (16%)	6 (2%)	36	120	0 (0%)
L, G	251 (68%)	29 (8%)	27 (7%)	50 (14%)	10 (3%)	36	111	0 (0%)
L, G, A, U	251 (68%)	30 (8%)	27 (7%)	51 (14%)	8 (2%)	36	114	0 (0%)
L, G, T, A	250 (68%)	30 (8%)	27 (7%)	52 (14%)	8 (2%)	36	112	0 (0%)
L, G, A	248 (68%)	30 (8%)	28 (8%)	53 (14%)	8 (2%)	36	112	0 (0%)
G, N, T, U	234 (64%)	38 (10%)	38 (10%)	44 (12%)	13 (4%)	37	94	0 (0%)
G, N, U	234 (64%)	38 (10%)	38 (10%)	44 (12%)	13 (4%)	37	95	0 (0%)
G, N, T	233 (63%)	38 (10%)	38 (10%)	45 (12%)	13 (4%)	37	93	0 (0%)
G, N	233 (63%)	38 (10%)	39 (11%)	44 (12%)	13 (4%)	37	93	0 (0%)
G, N, A, U	232 (63%)	38 (10%)	38 (10%)	47 (13%)	12 (3%)	37	96	0 (0%)
G, N, T, A, U	232 (63%)	38 (10%)	38 (10%)	47 (13%)	12 (3%)	37	95	0 (0%)
G, N, A	231 (63%)	38 (10%)	39 (11%)	47 (13%)	12 (3%)	37	94	0 (0%)
G, N, T, A	231 (63%)	38 (10%)	38 (10%)	48 (13%)	12 (3%)	37	94	0 (0%)
L, G, N, A, U	230 (63%)	47 (13%)	34 (9%)	42 (11%)	14 (4%)	37	93	0 (0%)
L, G, N, T, A, U	230 (63%)	47 (13%)	34 (9%)	42 (11%)	14 (4%)	37	92	0 (0%)
L, G, N, U	230 (63%)	47 (13%)	35 (10%)	40 (11%)	15 (4%)	37	91	0 (0%)
L, G, N, T, U	230 (63%)	47 (13%)	35 (10%)	40 (11%)	15 (4%)	37	90	0 (0%)
L, G, N, A	229 (62%)	47 (13%)	35 (10%)	42 (11%)	14 (4%)	37	91	0 (0%)
L, G, N	229 (62%)	47 (13%)	36 (10%)	40 (11%)	15 (4%)	37	89	0 (0%)
L, G, N, T, A	229 (62%)	47 (13%)	34 (9%)	43 (12%)	14 (4%)	37	91	0 (0%)
L, G, N, T	229 (62%)	47 (13%)	35 (10%)	41 (11%)	15 (4%)	37	89	0 (0%)
L, N, T, U	148 (40%)	64 (17%)	44 (12%)	47 (13%)	34 (9%)	33	67	30 (8%)
L, N, T	147 (40%)	64 (17%)	45 (12%)	47 (13%)	32 (9%)	33	67	32 (9%)
L, N, U	145 (40%)	63 (17%)	44 (12%)	46 (13%)	38 (10%)	32	65	31 (8%)
L, N, T, A, U	143 (39%)	65 (18%)	38 (10%)	62 (17%)	45 (12%)	33	69	14 (4%)
L, N, T, A	141 (38%)	64 (17%)	39 (11%)	65 (18%)	43 (12%)	33	70	15 (4%)
L, N, A, U	140 (38%)	63 (17%)	38 (10%)	64 (17%)	47 (13%)	32	68	15 (4%)
L, N	140 (38%)	62 (17%)	43 (12%)	46 (13%)	31 (8%)	31	63	45 (12%)
N, T, U	138 (38%)	59 (16%)	43 (12%)	51 (14%)	36 (10%)	34	68	40 (11%)
N, T	137 (37%)	60 (16%)	44 (12%)	50 (14%)	33 (9%)	34	68	43 (12%)
N, U	135 (37%)	59 (16%)	43 (12%)	49 (13%)	40 (11%)	33	66	41 (11%)
L, N, A	135 (37%)	63 (17%)	38 (10%)	61 (17%)	50 (14%)	31	66	20 (5%)
N, T, A, U	133 (36%)	60 (16%)	37 (10%)	66 (18%)	51 (14%)	37	69	20 (5%)
N, T, A	132 (36%)	59 (16%)	38 (10%)	68 (19%)	48 (13%)	37	70	22 (6%)
N, A, U	131 (36%)	58 (16%)	37 (10%)	67 (18%)	53 (14%)	36	68	21 (6%)
L, T, A, U	130 (35%)	40 (11%)	17 (5%)	36 (10%)	82 (22%)	17	50	62 (17%)
N,	130 (35%)	58 (16%)	42 (11%)	49 (13%)	32 (9%)	32	64	56 (15%)
N, A	126 (34%)	58 (16%)	37 (10%)	64 (17%)	55 (15%)	35	66	27 (7%)
L, T, A	125 (34%)	41 (11%)	19 (5%)	33 (9%)	84 (23%)	22	61	65 (18%)
L, T, U	125 (34%)	51 (14%)	23 (6%)	3 (1%)	49 (13%)	16	40	116 (32%)
L, T	122 (33%)	51 (14%)	22 (6%)	1 (0%)	44 (12%)	19	50	127 (35%)
L, A, U	92 (25%)	44 (12%)	21 (6%)	37 (10%)	93 (25%)	17	44	80 (22%)
L, U	85 (23%)	56 (15%)	26 (7%)	1 (0%)	62 (17%)	14	33	137 (37%)
T, A, U	84 (23%)	9 (2%)	2 (1%)	21 (6%)	105 (29%)	14	36	146 (40%)
L, A	82 (22%)	46 (13%)	21 (6%)	29 (8%)	91 (25%)	16	46	98 (27%)
T, A	80 (22%)	7 (2%)	5 (1%)	19 (5%)	104 (28%)	14	36	152 (41%)
L,	75 (20%)	54 (15%)	24 (7%)	0 (0%)	42 (11%)	11	32	172 (47%)
T, U	69 (19%)	6 (2%)	7 (2%)	0 (0%)	27 (7%)	11	19	258 (70%)
T,	62 (17%)	6 (2%)	8 (2%)	0 (0%)	10 (3%)	10	15	281 (77%)
A, U	27 (7%)	3 (1%)	1 (0%)	24 (7%)	125 (34%)	7	20	187 (51%)
A,	20 (5%)	3 (1%)	1 (0%)	15 (4%)	106 (29%)	6	21	222 (60%)
U,	9 (2%)	2 (1%)	4 (1%)	0 (0%)	49 (13%)	2	2	303 (83%)

Table S6: **County-level performance (Jan 1st, 2020 – Jan 1st, 2022) of the Multiple Sources method as a function of the data sources used as input** . The source labeling is as follows: G = Google trends, T = Twitter, U = UpToDate, L = local epidemiological data, A = Human mobility from Apple, and N = Neighbor's epidemiological data. An additional column, 'Non-deployed,' indicates the number of outbreak events where our Multiple Source method did not find a reasonable set of proxies to fit a model. Thus, an early warning system was not deployed to forecast the out-of-sample period.

	Naive	Single Source<br>“How long does covid last?”	Single Source<br>“side effects of vaccine”	Multiple Sources
Early	0.64	0.58	0.68	0.30
Early + Sync	0.63	0.56	0.66	0.28
Early + Sync + Late	0.63	0.56	0.66	0.28

Table S7: False Discovery Rates (FDR) for each methodology at the county-level. Each row shows the FDR with the inclusion of different warning signals (early, sync, or late).

	Naive	Single Source<br>“How long does covid last?”	Single Source<br>“chest pain”	Multiple Sources
Early	0.54	0.48	0.50	0.17
Early + Sync	0.51	0.46	0.48	0.15
Early + Sync + Late	0.51	0.46	0.48	0.15

Table S8: False Discovery Rates (FDR) for each methodology at the state-level. Each row shows the FDR with the inclusion of different warning signals (early, sync, or late).

Abdominal pain	Acute bronchitis	Ageusia	Anosmia	Anxiety
Asphyxia	Asthma	Bronchitis	Burning Chest Pain	Cardiac arrest
Chest pain	Chills	Chronic pain	Cough	Diarrhea
Dizziness	Dysgeusia Ear pain	Fatigue	Fever	Hyperventilation
Nasal congestion	Otitis	Phlegm	Pneumonia	Shortness of breath
Sore throat	Throat irritation	Vertigo	Viral pneumonia	Vomiting
Wheeze	after covid vaccine	anosmia	chest pain	chest tightness
covid	covid nhs	covid symptoms	covid-19	covid-19 who
effects of covid vaccine	feeling exhausted	feeling tired	fever	how long does covid last
joints aching	loss of smell	loss smell	loss taste	nose bleed
quarantine	sars-cov 2	sars-cov-2	side effects of vaccine	

Table S9: Google Search term list used in the Multiple Source method
